# Unveiling the Role of Low‐Coordinated Sites in CO_2_ Electroreduction Using Hierarchical Simulation Models

**DOI:** 10.1002/cplu.202500223

**Published:** 2025-07-08

**Authors:** Ádám Haffner, Tibor Höltzl

**Affiliations:** ^1^ Department of Inorganic and Analytical Chemistry Budapest University of Technology and Economics Műegyetem rkp. 3. H1111 Budapest Hungary; ^2^ HUN‐REN‐BME Computation Driven Chemistry Research Group Budapest University of Technology and Economics Műegyetem rkp. 3. H1111 Budapest Hungary; ^3^ Nanomaterials Science Group Furukawa Electric Institute of Technology Késmárk utca 28/A H1158 Budapest Hungary

**Keywords:** CO_2_ electroreduction, electrochemistry, metal clusters, quantum chemistry, solvent effects

## Abstract

Enhancing efficiency and product selectivity presents a significant challenge in carbon dioxide electroreduction (CO_2_RR). Recent studies have demonstrated that the solvent in the electrolyte plays a crucial role; however, its specific functions are being investigated intensively. The study utilizes hierarchically assembled models, enabling to decoupling of the various effects of the solvent. Both (sub)nanoclusters and surfaces with adatoms are utilized as model systems, which allow to study the effect of the solvent on the low‐coordinated reaction sites. It is observed that water binds to the low‐coordination active sites of the catalytic centers, thereby influencing the reaction mechanism. This binding leads to significant charge transfer between the solvent and the catalyst, altering its charge state and the potential of zero charge—both of which are known to affect product selectivity. Additionally, a solvent‐induced reorganization of the catalyst structure that can substantially influence reduction processes is observed. The solvation and solubility of the adsorbates also play a significant role, as they influence the desorption of the possible products from the catalyst surface.

Thus, the hierarchy of models presented here enables a systematic understanding of the microscopic role of solvents and paves the way for computational solvent engineering to optimize product selectivity in CO_2_RR.

## Graphical abstract

Designing an optimal catalyst for carbon dioxide electroreduction necessitates modeling various aspects of the actual catalyst. A hierarchical set of solvent models is setup, highlighting their potential applications and limitations in the electroreduction. The importance of the coordination numbers of the active sites is also demonstrated.

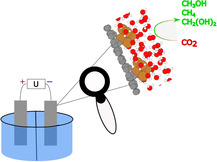

## 1 Introduction

An efficient way of reducing CO_2_ in the atmosphere would be its capture, followed by direct catalytic reduction into useful chemicals.^[^ ^1^, ^2^, ^3^, ^4^ ^]^ For the electrochemical CO_2_ reduction reaction (CO_2_RR),^[^ ^2^, ^3^, ^5^, ^6^, ^7^, ^8^, ^9^, ^10^, ^11^ ^]^ electricity, ideally from a renewable energy source, can be applied. An advantage of electroreduction is that it can be performed under ambient conditions, and it is possible to achieve relatively large conversions with only a small amount of catalyst. The products of CO_2_RR can not only be used for chemical syntheses, but also a sufficiently stable product (such as methanol) offers an alternative way for storing energy as an alternative to batteries.^[^ ^12^ ^]^ Thus, CO_2_RR is a promising Power‐to‐X technology,^[^ ^13^, ^14^ ^]^ where chemical compounds with high energy content are produced using electricity.

One of the critical aspects of electroreduction processes is the selection of the catalyst. Copper stands out as a promising option, as copper electrodes can effectively catalyze the formation of hydrocarbons from a bicarbonate solution.^[^ ^15^, ^16^, ^17^ ^]^

The solvent applied in the electrolyte strongly influences the reactivity under electrochemical conditions,^[^ ^2^, ^18^, ^19^, ^20^ ^]^ thus the inclusion of explicit water in modeling studies was reported to be of high importance.^[^ ^21^, ^22^, ^23^, ^24^, ^25^, ^26^ ^]^ The role of the solvent in CO_2_ reduction reactions (CO_2_RR) is multifaceted, as it serves several important functions: it dissolves CO_2_, acts as a proton source, maintains the necessary pH, creates a favorable microenvironment at the adsorption sites, and provides a medium for dissolving salts, and despite the intensive research it is still not understood in detail.^[^ ^4^ ^]^ The cations and anions, dissolved in the electrolyte, also play a significant role in the CO_2_RR.^[^ ^27^, ^28^ ^]^

Aprotic solvents have been reported to be effective for CO_2_ electroreduction on metal surfaces, which helps to increase the CO_2_ solubility and suppress the hydrogen evolution reaction (HER).^[^ ^4^ ^]^ Nonaqueous aprotic solvents, such as acetonitrile, attracted attention, as they lead to an outstanding selectivity toward oxalate or carbon monoxide with the co‐formation of carbonates.^[^ ^29^, ^30^ ^]^ The reduction, however, requires a higher potential bias in acetonitrile (around −2.5 to −3.0 V) than in aqueous media.^[^ ^31^ ^]^ Furthermore, aprotic solvents require sacrificial agents (e.g., sacrificial zinc anode).^[^ ^32^ ^]^

Altogether, the CO_2_RR product selectivity can be regulated by the local pH, the solvent polarity, and by the appropriate choice and concentration of the dissolved ions.^[^ ^33^ ^]^

Ikeda et al. reported the same products with different solvents (acetonitrile, dimethyl sulfoxide, and propylene carbonate) in experiments with high‐purity copper electrode.^[^ ^33^ ^]^

It has been shown by Marcandalli et al. that the reaction mechanism is needed for the detailed understanding of the various aspects of the solvent effects.^[^ ^34^ ^]^ The critical role of the solvent in the CO_2_RR modeling has been reviewed recently by Gholizadeh et al.^[^ ^35^ ^]^ The reaction paths of CO_2_ electroreduction on copper electrode surfaces have already been investigated both with and without explicit water molecules. Focusing on C_1_‐products, the work of Hussain et al.^[^ ^36^ ^]^ showed that in the absence of explicit water molecules and applied bias, methane formation is preferred; however, in the presence of explicit water molecules, carbon monoxide or methanol can be the most favored products, and the activation energy barrier toward C_2_‐products is also relatively low. They also reported that on contrary to the simulations with implicit water, formaldehyde was not present as an intermediate in the explicit water simulations. Whilst they investigated reaction paths starting with the formation of *COOH, van den Bossche et al.^[^ ^37^ ^]^ reported that *HCOO formation is thermodynamically more favored than *COOH formation in the first reduction step.

When discussing electrochemical conditions, however, it is worth noting that the formation energy difference between the *HCOO and *COOH adducts becomes smaller when applying higher potential biases compared to reversible hydrogen electrode (RHE).^[^ ^37^ ^]^ Despite this energy difference, the formation of carbon monoxide through *COOH and the formation of formic acid through *HCOO have similar onset potentials on copper surface, since the activation energy toward *COOH and *HCOO formation are nearly identical. Subsequently, Hussain et al.^[^ ^38^ ^]^ reported that the reduction mechanism on copper surfaces in the presence of two layers of explicit water may be different depending on the applied bias. When a less negative potential is used, the reaction path to form methanol goes through *CHO, while the application of a more negative potential leads to the formation through *COH. This agrees well with the experimental results of Schouten et al.^[^ ^39^ ^]^ Jiang et al. observed high selectivity toward methane formation in CO_2_ electroreduction on single‐atom copper catalyst with CeO_2_‐cluster support.^[^ ^40^ ^]^ Peterson et al. reported quantitative simulations of the voltage dependence of the main products on Cu(211) surface and the mechanisms on each reaction path using the computational hydrogen electrode (CHE) model.^[^ ^41^ ^]^ Here at small potential biases, (less negative than −0.41 V) exclusively hydrogen formation can be expected, formation of formic acid and carbon monoxide becomes favored at a somewhat more negative potential bias, whilst methane only becomes dominant at even more negative potential biases, (the onset potential is −0.74 V) agreeing with the results of Hori et al.^[^ ^42^ ^]^ In this range of potential biases, C_2_‐products such as ethylene can also be formed, which has been proven experimentally^[^ ^43^ ^]^ and the possible mechanisms have also been computed.^[^ ^44^ ^]^

However, it is important to note that the Cu(111) and Cu(100) surfaces, which are commonly used as model systems, are not catalytically active in CO_2_ reduction reactions (CO_2_RR).^[^ ^45^ ^]^ Instead, the observed reactivity can be attributed to the presence of defects and kinks on these surfaces. Moreover, nanoparticles and nanoclusters^[^ ^46^, ^47^, ^48^ ^]^ (particles composed of a countable number of atoms) were also found to be highly active catalysts for CO_2_RR. E.g., copper nanoparticles have been reported to show higher selectivity toward C_2+_ products at smaller potential biases.^[^ ^49^, ^50^ ^]^ This is because their activity and product selectivity can be tuned by their composition,^[^ ^51^ ^]^ size,^[^ ^52^, ^53^, ^54^ ^]^ shape,^[^ ^55^, ^56^ ^]^ and by the coordination symmetry of the active sites.^[^ ^57^ ^]^ Interestingly, metal cluster catalysts can spontaneously form during the CO_2_RR and can tune the electroreduction process and achieve higher selectivity for valuable products, such as hydrocarbons and alcohols.^[^ ^58^, ^59^, ^60^ ^]^ Catalytically active metal clusters also form on the copper electrode surface under operando conditions.^[^ ^61^ ^]^ Due to the presence of numerous edges and vertices, solvation in metal clusters and metal nanoparticles is significantly more complex than on bulk surfaces.

The mechanism of CO_2_ reduction on metal clusters is the subject of intense research.^[^ ^47^, ^52^, ^59^, ^62^, ^63^ ^]^ The mechanism of the free copper cluster catalyzed CO_2_RR has been investigated by Kumar et al. using an implicit solvent model.^[^ ^64^ ^]^ They showed that the thermodynamically favored reaction path leads to formic acid, but several paths toward other products, such as methane or methanol, can be observed. We recently investigated the CO_2_RR mechanism toward C_1−_ and C_2−_products on surface‐deposited copper clusters.^[^ ^65^ ^]^ We did not observe formic acid desorption; thus, the reduction could continue toward methane. Contrary to other cases where the reaction paths toward methane and methanol formation went through *CHO, we observed the formation of *CH_2_OOH instead.

In this study, we examine the role of the solvent in the electrolyte for CO_2_ reduction reactions (CO_2_RR), with a specific focus on the effects of the non‐ideal solvent‐metal interface. We chose metal clusters as model systems because they not only serve as effective catalysts but also provide an excellent framework for investigating the local reactivity of complex catalysts under clean and well‐defined conditions.^[^ ^63^, ^66^, ^67^, ^68^, ^69^ ^]^ The energetics of small metal clusters can also be computed by using high‐level methods, and upon embedding in extended structures,^[^ ^70^ ^]^ the size effects can also be investigated in subsequent studies.

## 2 Results and Discussion

**Figure 1:**
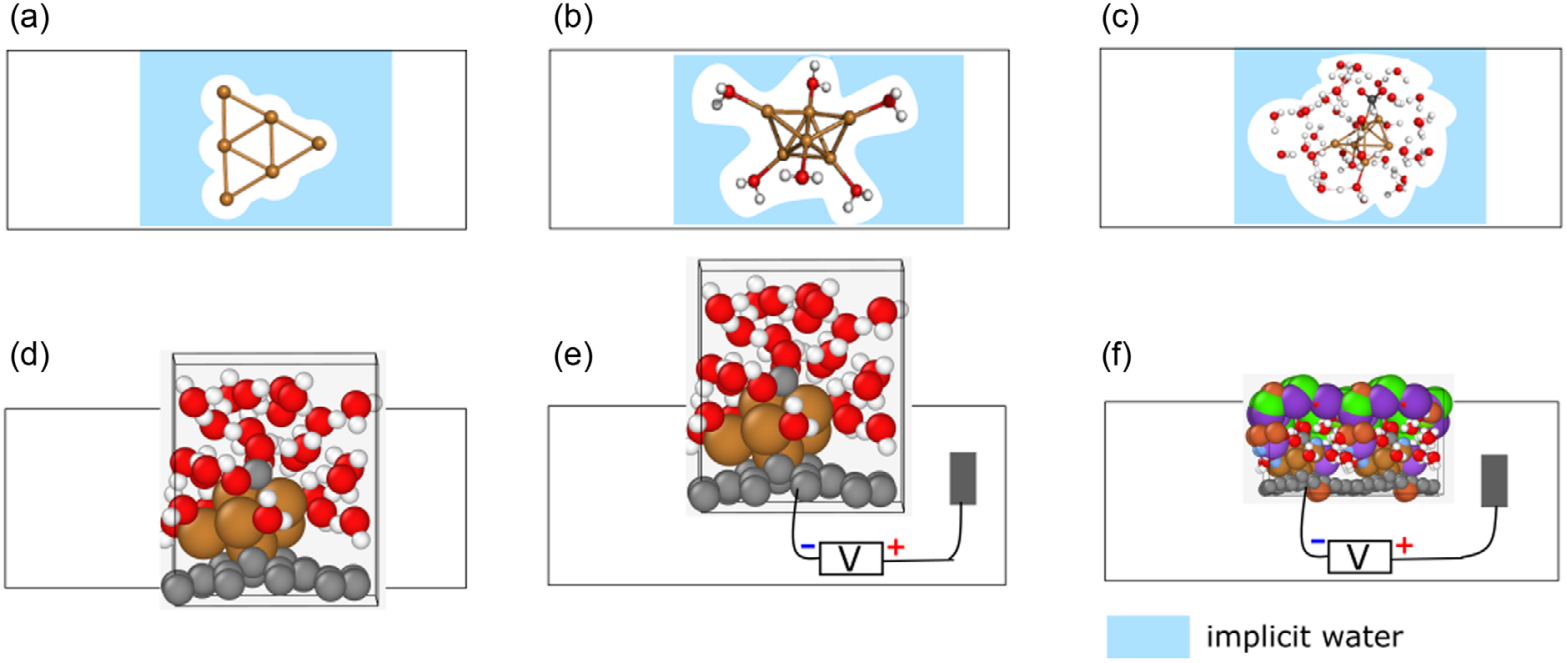
Models for metal cluster catalyzed CO_2_RR a) implicit solvent model, b) hybrid (implicit + explicit) model, c) explicit water using the ‘nanodroplet’ model, d) explicit water+cluster support, e) explicit water + cluster support including voltage bias, f) explicit water + cluster support, including water bias and the ions in the electrolyte.

Solvation can be modeled computationally using either an implicit model, where the solvent is represented as a continuous, polarizable medium with the quantum chemically treated atomic system embedded in a cavity, or an explicit model, where individual solvent molecules are explicitly included in the simulation. Implicit models are computationally more economical, but this comes at a cost of reduced accuracy. We adopt a systematic approach, beginning with an implicit solvation model and progressively enhancing it by incorporating explicit water molecules, as illustrated in **Figure** **1**. Please note that, with the exception of the surface‐supported metal cluster model, we employ a combined implicit‐explicit solvent model. In this approach, the explicitly solvated system is embedded within an implicit solvent model to reduce the edge effects.

**Figure 2:**
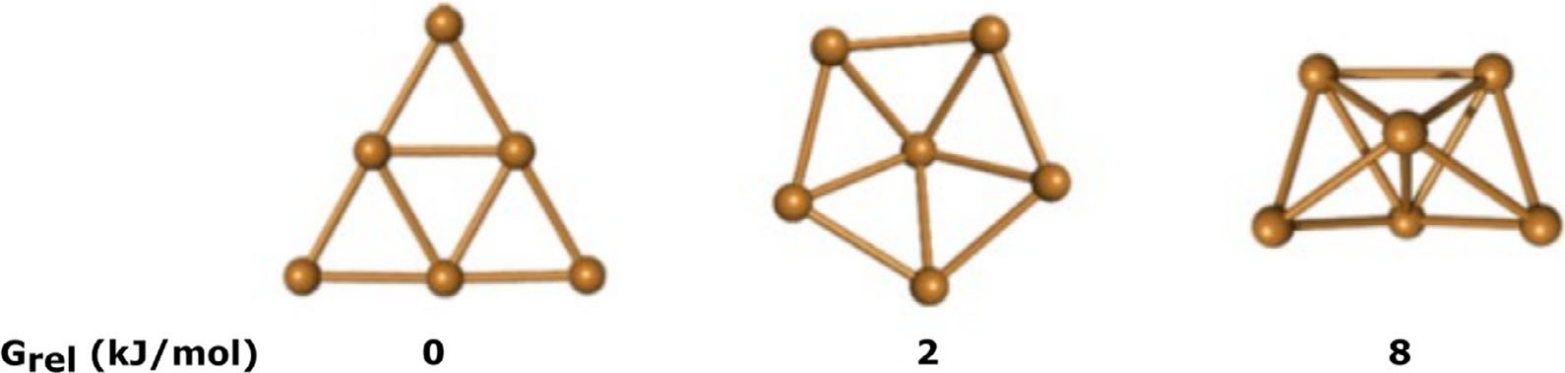
The most stable geometries of the Cu_6_ cluster and their relative free energies were computed in implicit water solvent.

Initially, we determined the most stable geometrical structure of Cu_6_ in an implicit solvent. The optimizations led to three different structures (**Figure** **2**), with the triangular‐shaped cluster being the most stable, consistent with previous works.^[^ ^71^, ^72^, ^73^ ^]^ However, the free energy differences between these structures are small, implying a potential fluxional behavior, which has been reported previously in different copper clusters.^[^ ^74^, ^75^ ^]^

We used these clusters to construct the models involving different numbers of explicit water molecules, Cu_6_[H_2_O]_x_ (x = 0,1,2,6).

**Scheme 1:**
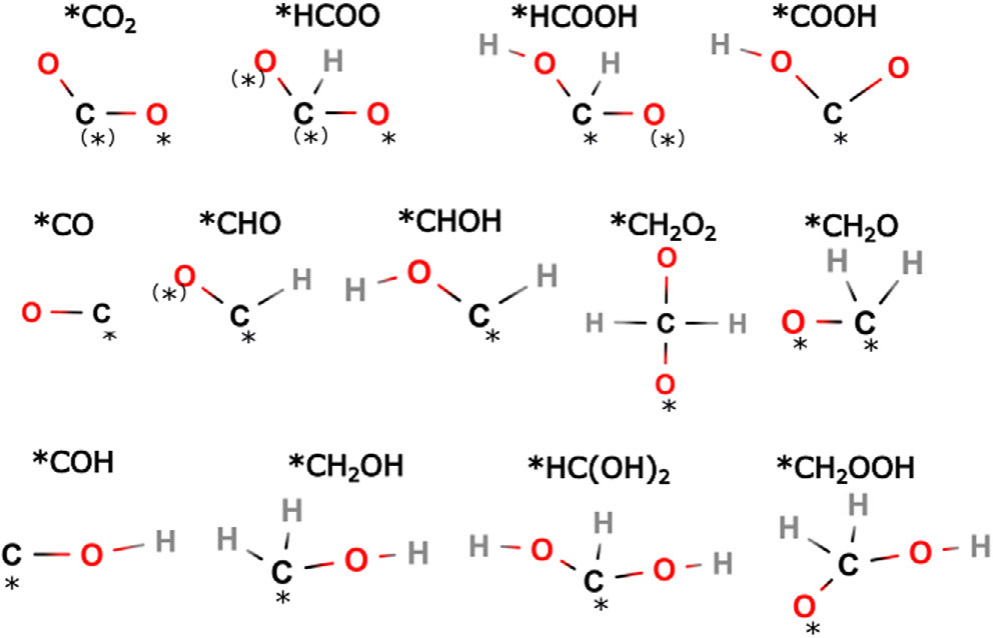
Reaction intermediates (the atom binding to the cluster catalyst is denoted by an asterisk; for certain fragments and clusters, binding in bridge position was the most preferred, which is denoted by an asterisk in brackets)

Next, we systematically applied the CHE model, i.e., unlike most of the previous studies, we attached the hydrogen atom not only to the adsorbate, but also to the cluster surface. Such a reaction could also take place in experiments, as it is an important step toward the HER, which acts as a competitive process of CO_2_RR.^[^ ^76^ ^]^ The reaction intermediates discussed in the article are schematically displayed in **Scheme** **1**.

**Figure 3:**
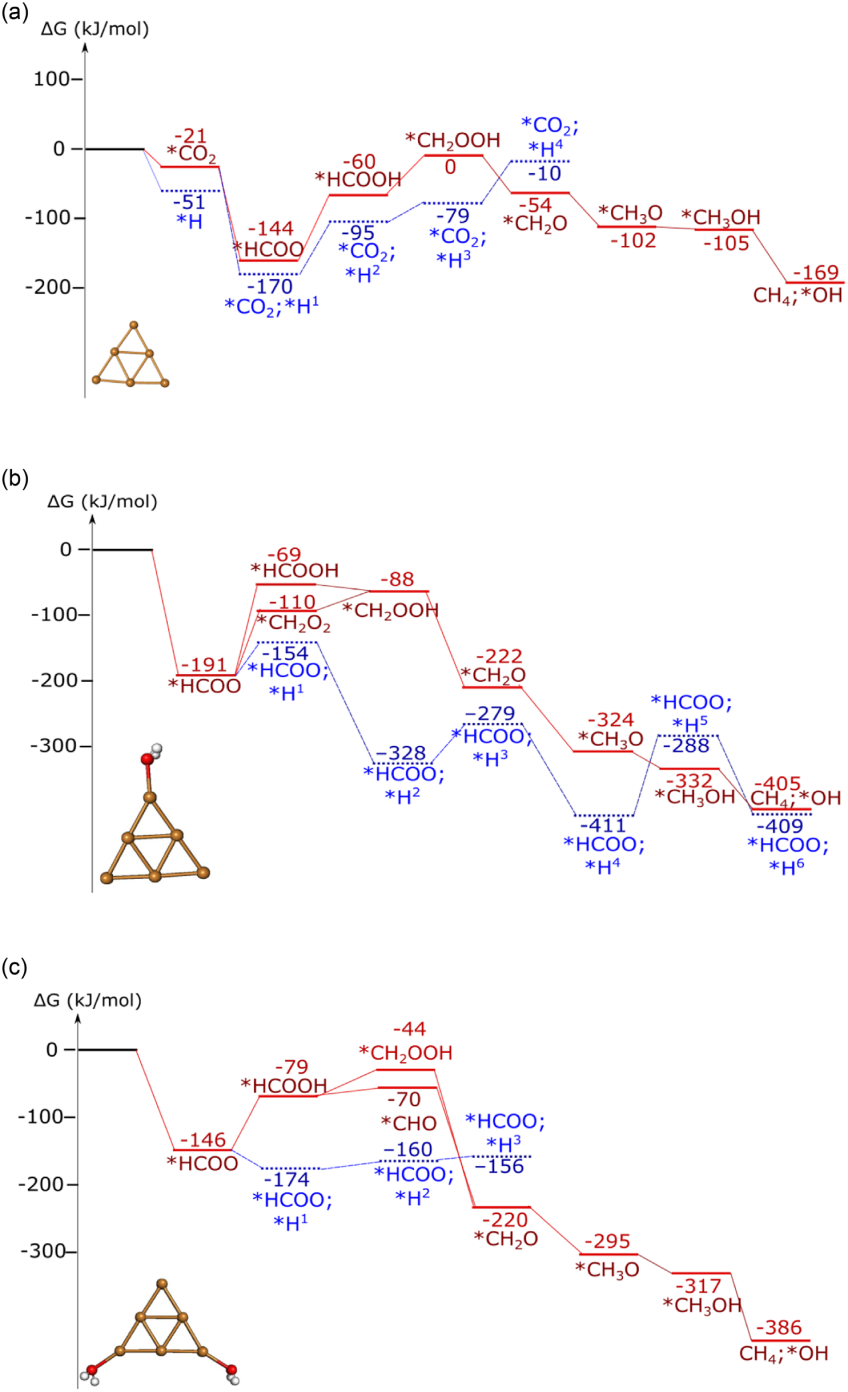
CO_2_RR mechanism on a) Cu_6_ b) Cu_6_[H_2_O]_1_ c) Cu_6_[H_2_O]_2_ Color coding: brown–copper, gray–carbon, red–oxygen, white–hydrogen. Relative free energies for each species are included in kJ mol^−1^.

Interestingly, we observed that for Cu_6_[H_2_O]_0−2_, instead of the CO_2_ reduction, copper hydrides were preferentially formed on the cluster (**Figure** **3**). In line, H^−^ in ligand‐protected copper hydride clusters^[^ ^77^, ^78^ ^]^ have been proposed to play a critical role in CO_2_RR. The occurrence of copper hydrides in nanoclusters is surprising, as they are stable only at low temperatures and their chemical synthesis requires complex techniques, such as sonochemical methods.^[^ ^79^ ^]^ The hydrogen binding free energy to Cu_6_[H_2_O]_0−2_ that we observed is significantly more negative than the −0.2 eV (−19.3 kJ mol^−1^),^[^ ^80^ ^]^ required for a good HER catalyst.

This shows stable hydride formation on Cu_6_[H_2_O]_0−2_ clusters under electrochemical conditions.

The preferred CO_2_ reduction pathways with the Cu_6_[H_2_O]_0−2_ catalyst begin with the formation of *HCOO, followed by further reduction to *HCOOH, *H_2_COOH, *CH_2_O, *CH_3_O, *CH_3_OH, and finally to methane, which spontaneously desorbs from the cluster (Figure 3). This path is similar to that we found previously for boron‐doped graphene‐anchored copper clusters.^[^ ^65^ ^]^ For Cu_6_[H_2_O]_1_, *CH_2_O_2_ was found to be more stable than formic acid. Interestingly, the formation of this intermediate was found to be unfavorable in a purely implicitly modeled solvent, as previously reported by Kumar et al.^[^ ^64^ ^]^ and according to our simulations. However, similarly to the bare Cu_6_, the reaction paths lead finally to methanol and methane. We observed important differences in the CO_2_RR paths on Cu_6_[H_2_O]_2_ cluster, as the *CHO (+H_2_O) intermediate is more stable than the *CH_2_OOH. It is important to point out that methanol did not desorb spontaneously; thus reduction could continue toward methane.

Raising the number of directly adsorbed explicit water molecules to six (i.e., an explicit water molecule is attached to each of the copper atoms of the cluster), the structure of the most stable cluster changed. Instead of the previously observed triangular‐shaped, planar structure, the cluster rearranged to a capped octahedral shape.

**Figure 4:**
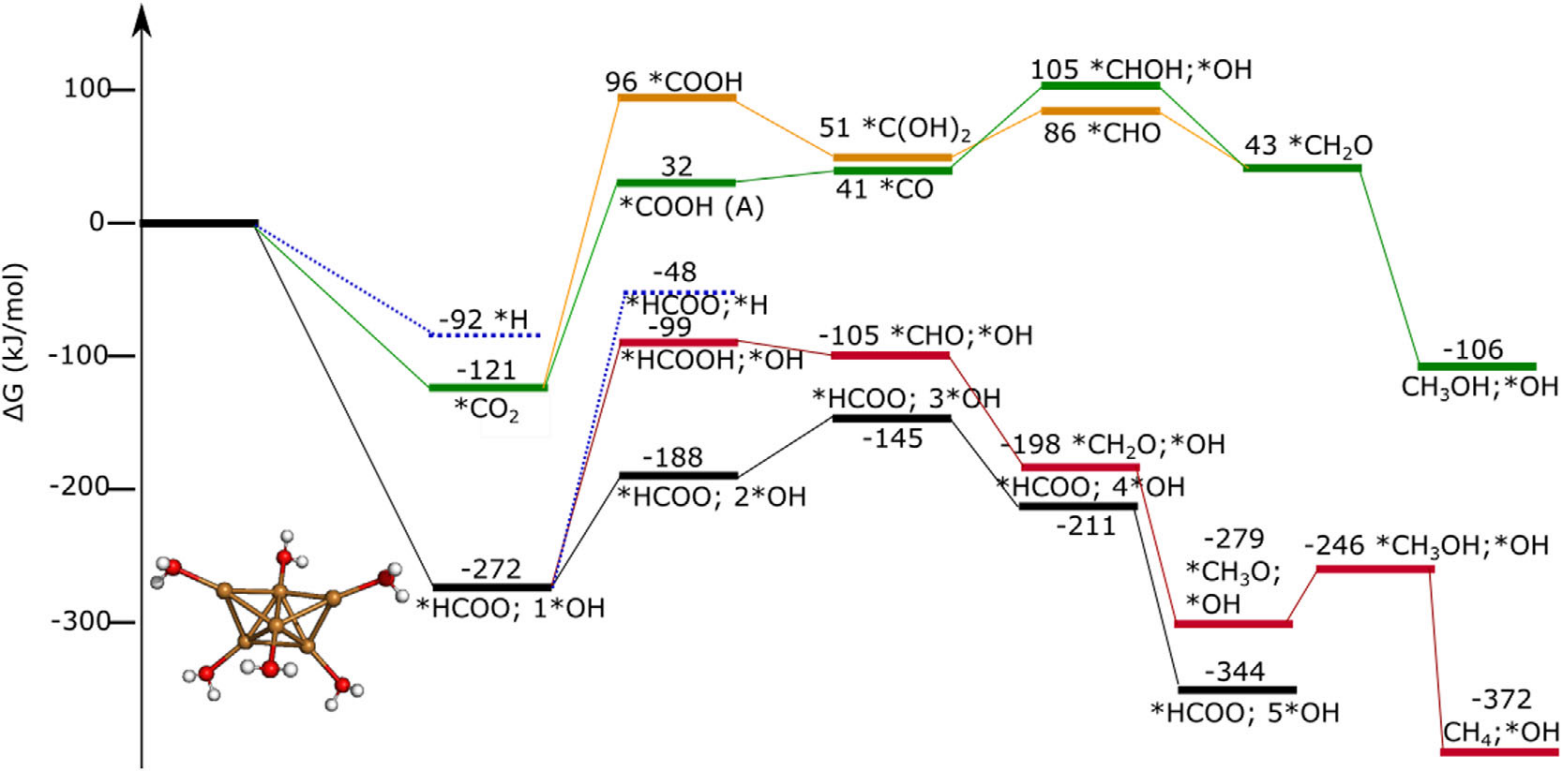
CO_2_RR mechanism on Cu_6_[H_2_O]_6_; Color coding: brown–copper, red–oxygen, white–hydrogen. Relative free energies for each species are included in kJ mol^−1^.

Starting the hydrogenation from this cluster (**Figure** **4**), the reduction of CO_2_ during the adsorption leads to the most stable intermediate of *HCOO, similarly to the previous cases. However, during the further hydrogenation, the formation of copper hydrides was less favored compared to the reduction of the adsorbed fragment. It clearly shows the necessity for an explicit solvation in CO_2_RR modeling. Direct hydride formation before CO_2_ adsorption is also presented in Figure 4.

Interestingly, in Cu_6_[H_2_O]_6_, the CO_2_ adsorption free energy is significantly more negative than in implicit solvent. This is in line with the several hydrogen bonds between the adsorbed CO_2_ and H_2_O that formed during the adsorption, and which are not present in the pure Cu_6_[H_2_O]_6_ cluster itself. In the presence of a greater number of explicit water molecules, a cavity around the adsorption site must form in the solvent to accommodate the CO_2_ molecule, where several hydrogen bonds that were present before the adsorption must break. Therefore, an additional increase in the number of water molecules is anticipated to reduce the strength of CO_2_ adsorption on the cluster.

Our computations showed that the formation of hydrides is thermodynamically unfavored on the Cu_6_[H_2_O]_6_ cluster. However, hydrogen evolution occurs through the direct reaction between the added hydrogen atom and one of the adsorbed water molecules. This process continues until a pentagonal structure with five *OH fragments in bridge positions appears. The energy decomposition analysis (EDA) showed that the charge transfer between the *OH fragments and the cluster is the main factor of the binding. Natural bond orbital analysis carried out on both the initial cluster with adsorbed water molecules and the final structure with *OH groups in bridge positions showed important differences in the natural charges of the copper atoms. Whilst in case of Cu_6_[H_2_O]_6_ the natural charges of the copper atoms are ranging between 0.1 and 0.4 with a total charge of 1.5 localized on the cluster, in case of Cu_6_*HCOO;5 *OH (pentagonal shape) the copper atoms in the pentagon have charges of 0.76–0.77 and the copper atom positioned in the middle has a charge of −0.20. These results indicate the presence of an oxidized, cationic cluster, capped by partially negative hydroxide groups. The Cu—O bond length is smaller in the case of the *OH in bridge position with 1.8–1.9 Å compared to 2.1–2.2 Å.

It is also important to note that, as water molecules are attached to each copper atom, CO_2_ adsorption must be preceded by the desorption of a single water molecule from the Cu_6_[H_2_O]_6_ cluster.

With the help of the CHE model, we investigated the stabilities of the structures with different numbers of *OH groups. The analysis showed that the structure with 5 *OH groups is the most stable in the absence of a potential bias (i.e., at 0 V compared to RHE). The reduction cannot continue on this path, as the most favored following step is the protonation of an *OH group, leading to the Cu_6_[H_2_O]_6_ *HCOO;4*OH adduct. However, by applying a −0.2 V bias, the structure with 1 *OH group became the most stable. In this case, the path marked in red can take place, leading to methane formation. These highlight that water is not only a solvent in the electrolyte, but can also partially oxidize the cluster surface, thus changing the catalyst's active state.

### 2.1 Nanodroplet Model

While the Cu_6_[H_2_O]_6_ accounted for the water binding to each copper atom of the cluster, it turned out that the hydrogen bonding network and the cavity formation play an important role; thus, a larger fraction of the solvent shell must be taken into account for a larger model. Thus, by increasing the number of explicit water molecules to form a complete water shell of 35 molecules around Cu_6_, we developed a metal cluster in a ‘nanodroplet’ model. This model is similar to that has been used before to investigate the solvation of Cu_7_ ‘nanoparticle’ by Stenlid et al.^[^ ^81^ ^]^

**Figure 5:**
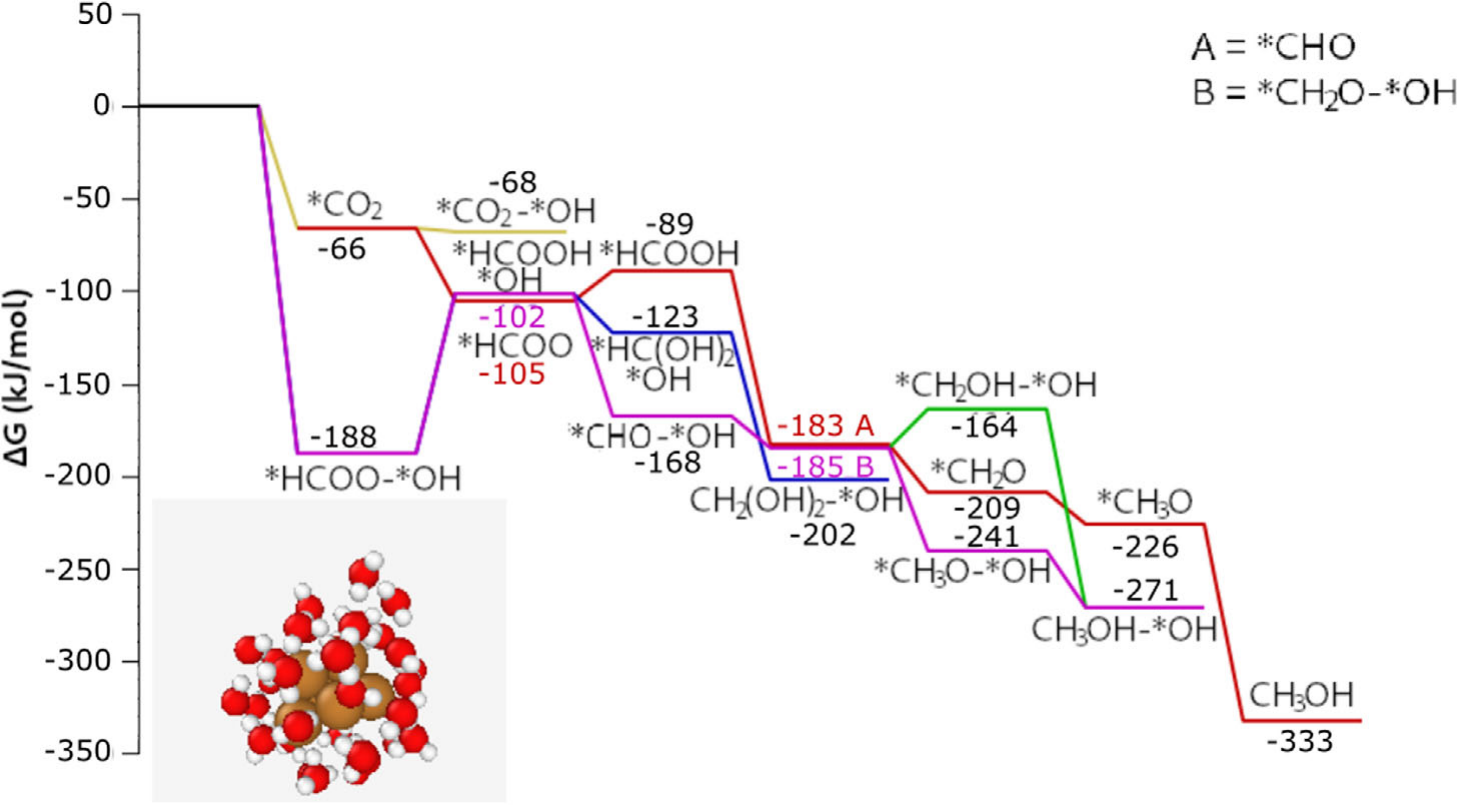
CO_2_RR mechanisms on Cu_6_[H_2_O]_35_ cluster, Color coding: brown–copper, red–oxygen, white–hydrogen. Relative free energies for each species are included in kJ mol^−1^.

The most favored reduction paths are reported in **Figure** **5**. This shows thatthe reaction path starting with the oxidation of the cluster by the water solvent and the formation of *HCOO is more favored than the reaction path starting with CO_2_ adsorption. This structure did not form spontaneously by adding CO_2_ to the system and was created with the removal of a hydrogen atom from one of the explicit water molecules and the addition of a hydrogen atom to the carbon dioxide in Cu_6_[H_2_O]_35_*CO_2_.

On the reaction path, we observed a new pathway leading to methanediol that we did not encounter with Cu_6_[H_2_O]_0–2,6_ clusters. It is important to note that methanediol is the hydrated form of formaldehyde and is the most abundant species in formaldehyde solutions. However, the preceding intermediate in the reaction path, *HC(OH)_2_ is less stable than *CHO; thus, the formation of methanediol is less likely than methanol formation. Even though methanediol was previously not considered as a product of CO_2_RR on copper clusters, Hansen et al. have shown that the reduction of formaldehyde on copper surfaces takes place through the reduction of methanediol.^[^ ^82^ ^]^ According to their computations, methanediol adsorbs on the copper surface after hydrogenation in the form of *CH_2_OH, and in the next hydrogenation step, methanol is formed as the product of this reduction process.

Formate is a possible product of the CO_2_RR; however, being an ionic species, its detachment is hindered in simulations using an implicit solvent model, while it may be possible in an explicit solvent, where the ion can be effectively solvated. Thus, we carried out molecular dynamics (MD) simulations to investigate its possible dissociation from the cluster surface.

The distance of the formate fragment and the cluster started to increase during the simulation, but it did not leave the solvent shell, and after 19‐ps simulation time, it stabilized at ≈2 Å distance from the closest copper atom. Without explicit water molecules coordinated to the cluster's surface, we observed complete desorption of formate, after which the fragment left the solvent shell. Further results related to this model are included in the Supporting Information. This emphasizes the importance of copper—water coordination, because it does not only affect the reaction free energies, but also the preferred CO_2_RR mechanism.

### 2.2 Surface‐Supported Cluster with Explicit Solvent

The nanodroplet model correctly accounted for both the cluster‐bound water and the solvation shell around the adsorbate, showing several important differences compared to the one using implicit solvent. Nevertheless, it is important to be aware of the limitations of the model, specifically the presence of a surface boundary. Small water clusters can lead to notable differences from bulk solvent, e.g., in the presence of a limited amount of explicit water molecules, HCl behaves as a weak acid.^[^ ^83^ ^]^ Thus, we constructed a periodic model that is free from artificial boundaries. This model also makes it possible to include the electrode surface in the simulations.

**Figure 6:**
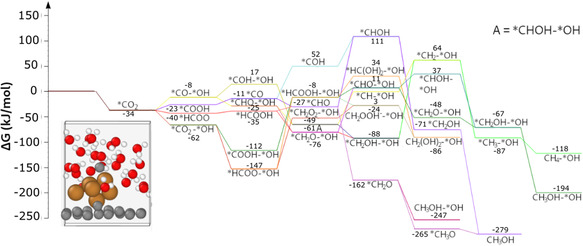
Reaction path of the surface‐deposited Cu_6_ cluster, surrounded by explicit water molecules. Color coding of the atoms: brown–copper, gray–carbon, red–oxygen, white–hydrogen. Relative free energies for each species are included in kJ mol^−1^.

We obtained the initial structure of the Cu_6_ cluster on a vacancy‐containing graphene surface from the work of García‐Rodríguez et al.^[^ ^84^ ^]^ The vacancy was created in the center of a graphene supercell of 32 atom in order to strengthen the interaction between the surface and the copper cluster. Whilst the bare Cu_6_ cluster has a symmetrical structure, the adsorption of the cluster resulted in a loss of symmetry. Thanks to the periodic boundary conditions, the graphene layer builds a formally infinite surface, and the space around the cluster is filled with 25 water molecules. This effectively removes the surface effects present in the nanodroplet model. The reaction path with the surface‐supported cluster can be seen in **Figure** **6**.

**Figure 7:**
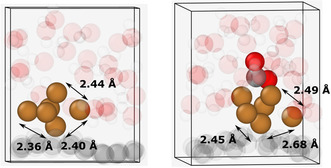
Atomic distances of the copper atoms of the cluster before and after the adsorption of CO_2_. Color coding: brown–copper, gray–carbon, red–oxygen, white–hydrogen.

The characteristic distances are changing due to the CO_2_ adsorption (**Figure** **7**). It is well visible that the distance of one of the copper atoms from the others increased significantly, thus this system can formally be regarded as a Cu_5_ cluster with a less strongly bound Cu. This agrees with the results of Lopez‐Caballero et al., who reported that the Cu_5_ cluster is outstandingly stable under electrochemical conditions.^[^ ^85^, ^86^ ^]^

**Figure 8:**
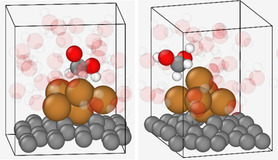
The desorbing CH_2_OOH^−^ anion is protonated during the MD simulation, leading to methanediol as the product. Color coding: brown–copper, gray–carbon, red–oxygen, white–hydrogen. The solvent molecules are rendered transparently.

**Figure 9:**
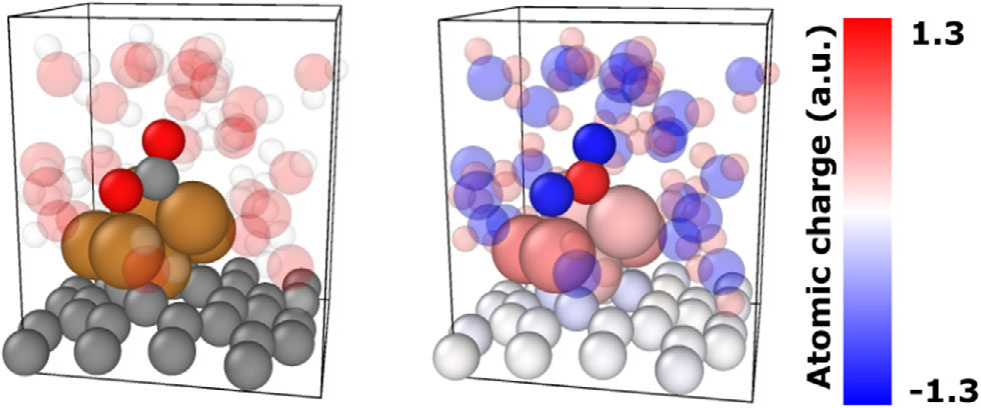
The atomic charges of the surface‐deposited cluster at its ground state. Color coding: brown–copper, gray–carbon, red–oxygen, white–hydrogen. The solvent molecules are rendered transparently.

It is important to mention that hydrogenation of CO_2_ did not occur spontaneously in the simulation, so we needed to form the formate fragment, similar to the ‘nanodroplet’ model. However, the bent *CO_2_ fragment indicates that the reduction began in this model as well, potentially forming a *COO^−^ ion. Unlike in the previous models, the direct adsorption of CO_2_ appeared to be more stable than the immediate reduction to formate. Because of this, we only discuss the reaction paths starting with direct adsorption in the main text, however, the reaction path starting with the *HCOO formation can also be seen in the Supporting Information. After the reduction of *CO_2_, the reaction paths proceed similarly to those, which we experienced in the nanodroplet model, however, the reaction free energy differences between the intermediates are generally lower. In this model, the product on the thermodynamically most favored reaction path is methanediol, instead of methanol that we observed in the nanodroplet model. While methanol formation is preferred thermodynamically, the key intermediate toward methanediol, *CH_2_O_2_;*OH has a more negative reaction free energy than that of *HCOOH;*OH, the key intermediate toward methanol. From *CH_2_O_2_;*OH, the reduction can only continue to methanediol formation according to our simulations. This reaction path proceeds through a hydrogen formation step and an *OH group binding to the cluster. This step might be less favored at higher potential bias as it can be explained as the oxidation of the cluster and the reduction of the proton added to the system. On this path, the CH_2_OOH^−^ anion already desorbs from the cluster. This anion is a formal adduct of formaldehyde and a hydroxide ion and can be considered as a deprotonated methanediol. We carried out MD simulations to investigate the effect of the solvent reorganization during the desorption of the adsorbate. **Figure** **8** shows the initial and final geometries of this 10 ps simulation. This showed that the CH_2_OOH^−^ anion is protonated by a water molecule, while the resulting OH^−^ ion binds to the partially positive cluster surface (atomic charges are displayed on **Figure** **9**).

The desorption of HCOO^−^ could not be clearly seen in this system; only a slight increase of the copper–carbon distance could be observed during the MD simulation. Thus, this reaction path can continue to methanol formation with a similar mechanism as described for the nanodroplet model.

### 2.3 Comparison of the Different Models

**Figure 10:**
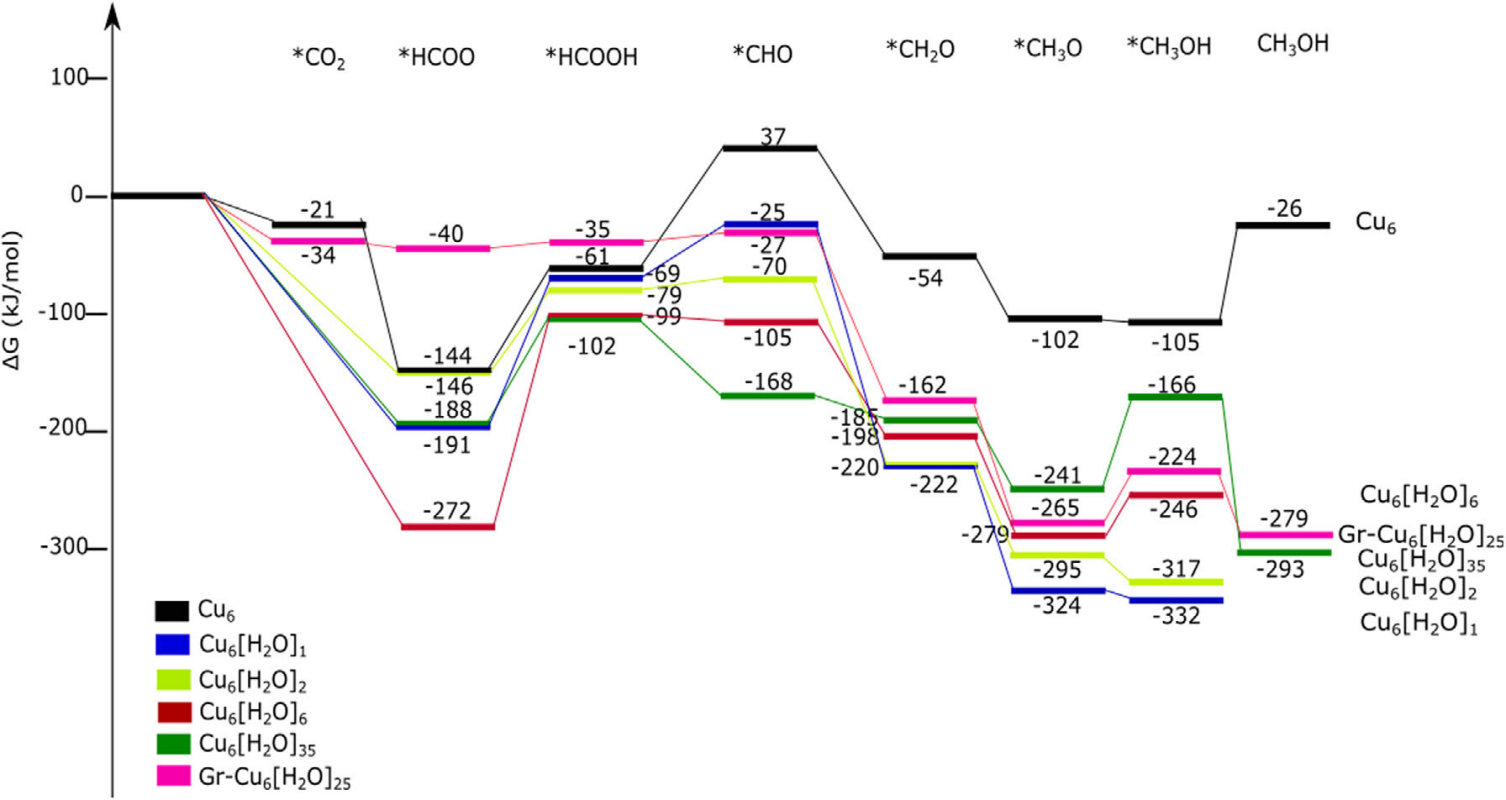
Comparing identical CO_2_RR paths, catalyzed by Cu_6_[H_2_O]_x_ (x = 0,1,2,6,35) or Gr‐Cu_6_[H_2_O]_25_ (adsorbed *OH groups are not indicated in the respective names; CH_3_OH indicates methanol desorption inside the solvent shell for periodic structures, for bare Cu_6_, the reaction free energy was computed as $G_{{\mathrm{CH}}_{3}\mathrm{OH}}+G_{{\mathrm{Cu}}_{6}*{\mathrm{CH}}_{3}\mathrm{OH};*\mathrm{OH}\left(H_{2}O\right)}‐G_{{\mathrm{Cu}}_{6}*\mathrm{OH}\left(H_{2}O\right)}$).

In **Figure** **10** we compare the reaction paths leading to methanol as obtained using the different models. The paths for the bare Cu_6_ and the surface‐deposited Gr‐Cu_6_[H_2_O]_25_ cluster start with direct CO_2_ adsorption, whilst other paths start with an immediate reduction to *HCOO, as described before. Here we exclude the hydride formation mechanisms, as hydride formation is suppressed with the inclusion of a sufficient number of explicit water molecules and methanediol formation, as it is not present in the case of Cu_6_[H_2_O]_0−2,6_ clusters. The reaction free energy values show clear trends in all cases except for the formate fragment. In the case of the surface‐supported Gr‐Cu_6_[H_2_O]_25_ cluster, *HCOO appeared to be less stable than in the other cases. The reaction free energies of *HCOOH and *CHO fragments became more negative with the increased number of explicit water molecules, whilst by *CH_2_O, *CH_3_O, and *CH_3_OH the reaction free energy became more positive. The methanol desorption was exoergic in the case of ’nanodroplet’ model and the surface‐deposited cluster, clearly demonstrating the importance of the solvation.

**Figure 11:**
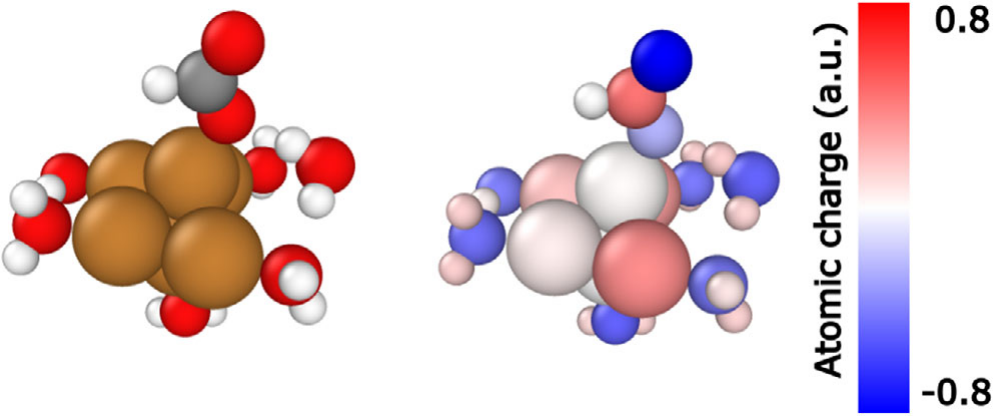
Geometry (left) and Bader atomic charges (right) of Cu_6_[H_2_O]_6_*HCOO. (Left structure is color coded according to chemical elements: brown–copper, gray–carbon, red–oxygen, white–hydrogen; on the right: the same structure color coded according to Bader atomic charges as of the scalebar).

It is important to note that the reaction free energies span a wide range in the case of *HCOO and *CHO, while this range is relatively narrow in the case of *HCOOH and *CH_2_O. This can be related to the partial ionic character of the adducts, as the increased ionic character implies a more important role of the accurate description of the solvation. However, the Bader charges for the intermediates of the Gr‐Cu_6_[H_2_O]_25_ and Cu_6_[H_2_O]_6_ catalyzed reaction paths clearly showed that the charge differences between the different fragments are small. All reduced fragments showed a negative overall charge and the charges of *CH_2_O and *HCOOH were only slightly less negative than the charge of *CHO or *HCOO. Generally, the difference of the Bader charges was less than 10% in all cases, and the negative charge of the adsorbed fragments is increasing with the number of explicit water molecules. The Bader analyses also showed that while in the ’nanodroplet’ model and on the surface‐deposited cluster all explicit water molecules were approximately charge neutral, in the case of Cu_6_[H_2_O]_6_ the waters had more negative charges, which is compensated by the cluster atoms. Another interesting difference is in the case of the formate fragment. This fragment adsorbed on the ’nanodroplet’ model or on the surface‐deposited cluster shows a symmetric charge distribution inside the fragment, the oxygen being negative with a ≈0.01 electron difference in their charges. The carbon atom is positive, and the hydrogen is approximately charge neutral. In contrast, in the case of Cu_6_[H_2_O]_6_, the formate fragment has an asymmetric charge distribution with the oxygen connecting to the cluster being less negative than the oxygen pointing to the gas phase with a difference of 0.57 electrons in their charges (**Figure** **11**). We expected that the *HCOO fragment would have symmetrical charge distribution. However, the oxygen atom binding to the cluster forms a hydrogen bond with an explicit water molecule, while the other oxygen is not stabilized by a hydrogen bond. The strong hydrogen bond can explain the outstanding stability of *HCOO with Cu_6_[H_2_O]_6_. Therefore, we think that six explicit water molecules would give less reliable results for the formate fragment, as the simulations showed more negative reaction free energy than in other cases. The charges are significantly different from those observed for Gr‐Cu_6_[H_2_O]_25_ cluster (Figure 9), because there is a larger internal charge separation within the water molecules. This is expected to be caused by the effect of the surface and the different behavior of bulk water and a small number of explicit water molecules (as part of a hybrid (explicit+implicit) solvent model).

In the case of *CH_3_OH, we observed large differences in the reaction free energies computed with different models. It may be related to the thermodynamically not favored desorption of this product in implicit water,^[^ ^64^ ^]^ whilst it desorbs spontaneously when an appropriate number of explicit water molecules (25–35 in our simulations) is present.

### 2.4 Influencing CO_2_RR Through Solvent Engineering

Our computations indicate that water molecules can interact with the active sites of the cluster catalyst through their oxygen atoms, a binding mechanism that has previously been noted in single‐atom catalysts.^[^ ^87^ ^]^ The strong binding is due to the presence of multiple low‐coordination sites within the cluster. Consequently, the results presented here indicate that the robust interaction with water should be taken into account when understanding the recently synthesized metal cluster‐based catalysts for the CO_2_RR.

**Figure 12:**
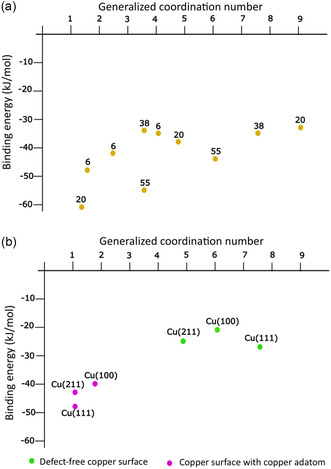
Binding energy of water with respect to the generalized coordination number of the site a) copper clusters of various sizes (the numbers denote the cluster size) and b) copper surfaces (green–perfect surface, magenta–surface with an adatom).

However, low‐coordination reaction sites are also found in nanoparticles and on defective surfaces. To evaluate the generality of the significance of these low‐coordination sites and the limitations of this interpretation, we also analyzed larger clusters and metal surfaces, including those with an adatom. (**Figure** **12**). To investigate the effect of coordination, we considered triangular geometry for Cu_6_, tetrahedral geometry for Cu_20_ (although this latter is not the global minimum),^[^ ^88^ ^]^ for Cu_38_ the truncated octahedron, and for Cu_55_ the icosahedral geometry. Binding site coordination has been quantified using the generalized coordination number using the method described by Calle‐Vallejo et al.^[^ ^89^ ^]^

It is well known that metal clusters show greatly size‐dependent properties, the addition of even a single atom can alter their reactivities,^[^ ^64^ ^]^ and apart from the coordination number, electronic effects also play an important role.^[^ ^88^, ^90^ ^]^ This is well reflected in Figure 12a, where the low‐coordinated site of the icosahedral Cu_55_ and the intermediate coordination site of Cu_20_ are outliers. A tendency of stronger water binding to low‐coordination sites is clearly observed.

Figure 12b illustrates that water binds significantly more strongly to low‐coordination adatoms on copper surfaces compared to an ideal surface. This clearly demonstrates that coordination plays an important role not only in clusters but also in larger CO_2_RR catalysts.

Our simulations showed that the binding of water to the active sites has several significant implications for reactivity. First, water can obstruct these active sites, necessitating its desorption before CO_2_ can be adsorbed. This leads to a less favored gross reaction energy for the adsorbate binding, thereby hindering the onset of the process. It is noteworthy that relatively strong binding is observed not only in the case of water molecules (−44 kJ mol^−1^), but also with acetonitrile (−67 kJ mol^−1^) computed using ωB97X–D/def2–TZVP level in Q‐Chem. Consistent with the relatively strong binding of acetonitrile molecules to the low‐coordination adsorption sites, CO_2_ adsorption was found to be the rate‐limiting step in Cu nanoparticle CO_2_RR when using this solvent, as reported recently by Oppel et al.^[^ ^91^ ^]^

In our computations, the EDA showed that the observed binding is due to the charge transfer between the solvent and the surface, leading to partially positive copper atoms at the solvent‐metal interface. Thus, the potential of zero charge, which is a crucial parameter for the product selectivity in CO_2_RR,^[^ ^92^ ^]^ can be tuned by the change of the solvent. In particular, the more electronegative binding atom of the solvent will generally lead to higher charge transfer. Thus, charge transfer is expected to be more significant in water than in acetonitrile.

Water can also dissociate on the low‐coordinated active sites, allowing the cluster to be partially oxidized at bias potentials close to 0 V (vs. RHE), while at more negative potentials the cluster is reduced gradually. Thus, the active form of the catalyst is determined by the solvent. This clearly illustrates how the solvent influences the formation of the active site. The electrolyte induced transition of the oxidation state plays an important role in determining the product selectivity in CO_2_RR.^[^ ^93^ ^]^ The surface charge of the catalyst is known to strongly influence product selectivity, and this charge can be influenced by the solvent.^[^ ^94^ ^]^ It has been shown that the Cu^+^/Cu^0^ interface improves the CO_2_ activation and the CO–CO coupling toward C_2+_ products.^[^ ^95^ ^]^

Interestingly, we observed solvent‐induced restructuring of the catalyst's active sites between the bare Cu_6_ cluster and the Cu_6_[H_2_O]_6−35_ clusters, even though the MD did not exhibit fluxional behavior. This transition occurs when an adequate number of explicit water molecules are included, highlighting that solvent binding is a crucial factor in this process. Notably, copper catalysts are well known for their dynamic behavior under electrochemical conditions, where the applied potential and the intermediates can also lead to restructuring.^[^ ^61^, ^96^ ^]^ Our results emphasize the importance of considering solvent effects, particularly in the presence of low‐coordination active sites within the catalyst.

Our computations show that the explicit water solvent clearly changes the reaction mechanisms. With an appropriate choice of water content, the required potential bias can be lowered, and thus a significant enhancement of the electroreduction can be achieved, as reported by Rudnev et al.^[^ ^97^ ^]^ This is in line with what we observed in the case of Cu_6_[H_2_O]_1–6_ cluster models, where we could observe that already with a small potential bias, there was a high selectivity toward methane formation through thermodynamically highly favorable reaction paths. It has been reported previously^[^ ^77^, ^78^ ^]^ that copper hydride clusters can effectively catalyze CO_2_ electroreduction with a lattice hydride mechanism where a hydride of the cluster is reducing CO_2_ already by the adsorption to *HCOO. This agrees with our results, as the reduced binding appeared to be more favored according to our computations. Later, the reduction continued with another hydride transport, forming HCOOH as a product, and the hydride cluster was regenerated. According to our results, this is unlikely and might be an artifact in implicit water simulations, as hydride cluster formation in explicit water is less favored than the reduction of CO_2_ through a proton–electron transfer. Hydrated copper hydride clusters might catalyze the electroreduction efficiently; however, their activity is expected to decrease significantly in time as there will be less hydride in the system.

It is also noteworthy that solvation and solubility of potential products play a significant role in the reaction mechanism. The explicit solvent affects the desorption of molecules from the cluster, facilitating the desorption of some species, such as methanol or formaldehyde in the form of methanediol, while making the desorption of others, like formic acid or formate derived from CO_2_, thermodynamically less favorable. In the presence of a sufficient number of explicit water molecules, methanol tends to desorb spontaneously from the cluster, indicating that the inclusion of explicit water not only influences the results quantitatively but also qualitatively. On a defective graphene support, we observed that methanediol is the thermodynamically favored product on the Cu_6_ cluster, whereas the formation of methanol and methane is less preferred.

Without explicit water, we observed high selectivity toward methane formation, whilst in the presence of explicit water, the product selectivity depends on the conditions, as methanediol may be formed at low potential bias and methanol at higher potential bias. This opens the possibility toward tuning product selectivity through solvent engineering as well (using, for example, non‐polar or aprotic solvent or a mixture with water).

In this article, we provided insights into how the explicit solvent can influence the CO_2_ reduction mechanism, particularly highlighting the role of low‐coordinated catalytic sites. This model can be readily extended through more elaborate simulations of the electrode potential (illustrated in Figure 1e), such as using grand canonical density functional theory (GC‐DFT) methods like the solvated jellium model,^[^ ^98^ ^]^ and by explicitly modeling the ions in the electrolyte (Figure 1f), which may influence the electroreduction processes by stabilizing the adsorbed *CO_2_ molecule on the catalyst's surface.^[^ ^99^ ^]^

## 3 Conclusions

In this study, we demonstrated the microscopic role of solvation in determining product selectivity by employing a systematic hierarchy of models. This approach enabled us to decouple the complex effects of solvents on the CO_2_ reduction reaction (CO_2_RR) mechanism. Implicit solvent models effectively captured the polarization effects of the solvent, while significant charge transfer between the solvent and the catalyst was observed when water molecules were considered explicitly.

Specifically, the solvent binds to the low‐coordinated sites of the cluster catalyst, thus changing not only its microenvironment but also leading to substantial charge transfer and even to structural reorganization. The solvent binding to the low‐coordinated sites changes the local charge and the potential of zero charge as well.

Our computations indicated binding not only with water, but also with acetonitrile; thus, these effects should occur with organic solvents as well. We believe that the reactivity of clusters may not only be influenced by well‐known quantum confinement effects, but also by the low coordination of sites as presented in this article.

The strong influence of the solvent on the simulated reaction mechanism implies the potential for solvent engineering with the electrolyte, beyond the already known CO_2_, salt solubility, and pH effect.

## 4 Computational Methods

Geometries and vibrational frequencies of clusters, with up to six explicit water molecules were computed using the ωB97X–D/def2–TZVP method and conductor‐like polarizable continuum (CPCM) solvation model,^[^ ^100^ ^]^ as implemented in the Q‐Chem 5.4. software.^[^ ^101^ ^]^ The functional and the basis set were selected by benchmarking to CCSD(T)/def2–QZVPPD (coupled cluster singles, doubles and perturbative triples and the quadruple‐zeta polarized basis set with diffuse functions) reference energies.

The reaction intermediates were optimized without constraints, and vibrational analysis was also carried out to prove that local minima are located. The reaction free energies were calculated based on the CHE model. The free energies of each species in the electrolyte were computed as

(1) $$G_{\text{solvated}}=E+H_{\text{vib}}-T\cdot S_{\text{vib}}+G_{\text{solv}},$$

where E is the internal energy of the species, $H_{\text{vib}}$ and $S_{\text{vib}}$ are the vibrational contributions to the enthalpy and the entropy, while $G_{\text{solv}}$ is the solvation free energy in the implicit solvent model computed for the given species and *T* is the absolute temperature.

For species in the gas phase, we included external translation and rotation terms as well ($H_{\text{trans}},H_{\text{rot}},S_{\text{trans}}$, and $S_{\text{rot}}$ are the translational/rotational enthalpy/entropy, respectively):

(2) $$G_{\text{gas phase}}=E+\left(H_{\text{vib}}+H_{\text{trans}}+H_{\text{rot}}\right)-T\cdot \left(S_{\text{vib}}+S_{\text{trans}}+S_{\text{rot}}\right).$$

All results refer to 298 K, 1 bar, and include zero‐point energy.

The ‘nanodroplet’ model (Figure 1c) was built using the Packmol software.^[^ ^102^ ^]^ After building the structure, a preoptimization step was performed using GFN‐force field^[^ ^103^ ^]^ method in xTB software.^[^ ^104^ ^]^ The obtained structures were then optimized using the GPAW software^[^ ^105^ ^]^ by applying the Perdew–Burke–Ernzerhof (PBE) functional in conjunction with Grimme‐D3 dispersion correction (PBE‐D3) and the triple‐zeta, polarized (TZP) basis set. For the nanodroplet model we used 1·1·1 *k*‐point grid and a 10 Å vacuum in each direction to avoid the interaction between the replicas of the cell. After the optimization, we performed single‐point energy computations in the Q‐Chem software using the ωB97X–D/def2–TZVP (range separed hybrid functional with empirical dispersion correction and the triple‐zeta, polarized basis set) level of theory in implicit solvent and vibrational analysis in GPAW using the PBE‐D3/TZP method and the harmonic approximation. The internal energies of the intermediates originated from Q‐Chem, whilst other thermochemical parameters came directly from GPAW. These results refer to 298 K and 1 bar in the gas phase.

Graphitic carbon electrode, widely used for electroreduction processes in practice^[^ ^106^ ^]^ was modeled by using a single graphene sheet, with the cluster anchored to a vacancy, as suggested by Curtiss et al. (Figure 1d,e).^[^ ^107^ ^]^ The computations were performed similarly to the ‘nanodroplet’ model, but we used a 2*⋅*2*⋅*1 *k*‐point grid and 11.75 Å cell size in the nonperiodic direction. The reaction free energies were corrected for the basis set superposition error using the counterpoise method.^[^ ^108^ ^]^ Additionally, a linear correction of the energies was applied to account for the different functionals (further details and the reaction free energies prior to the correction are included in the Supporting Information).

The MD simulations were carried out using GPAW, employing the PBE–D3/DZP (basis set of double‐zeta, polarized quality) level of theory with a time step of 0.5 fs. For keeping the temperature constant at 298 K, we used a Langevin thermostat with a friction coefficient of 10^−3^ ps^−1^.

Water molecules formed as a byproduct of the CO_2_RR were not removed from the cluster surface. The figures included in the article were created using OVITO‐Open visualizaton tool^[^ ^109^ ^]^ and Inkscape^[^ ^110^ ^]^ software.

## Conflict of Interest

The authors declare no conflict of interest.

## Supporting information

Supplementary Material

## Data Availability

The data that support the findings of this study are available in the supplementary material of this article.

## References

[1] M. D. Porosoff , B. Yan , J. G. Chen , Energy Environ. Sci. 2016, 9, 62.

[2] S. Nitopi , E. Bertheussen , S. B. Scott , X. Liu , A. K. Engstfeld , S. Horch , B. Seger , I. E. L. Stphens , K. Chan , C. Hahn , J. K. Nørskov , T. F. Jaramillo , I. Chorkendorff , Chem. Rev. 2019, 119, 7610. 31117420 10.1021/acs.chemrev.8b00705

[3] O. G. Sánchez , Y. Y. Birdja , M. Bulut , J. Vaes , T. Breugelmans , D. Pant , Curr. Opin. Green Sustainable Chem. 2019, 16, 47.

[4] D. Gao , R. M. Arán‐Ais , H. S. Jeon , B. Roldan Cuenya , Nat. Catal. 2019, 2, 198.

[5] G. Liu , P. Poths , X. Zhang , Z. Zhu , M. Marshall , M. Blankenhorn , A. N. Alexandrova , K. H. Bowen , J. Am. Chem. Soc. 2020, 142, 7930. 32250623 10.1021/jacs.0c01855

[6] A. Bagger , W. Ju , A. S. Varela , P. Strasser , J. Rossmeisl , ChemPhysChem 2017, 18, 3266. 28872756 10.1002/cphc.201700736

[7] D. Ješić , D. L. Jurković , A. Pohar , L. Suhadolnik , B. Likozar , Chem. Eng. J. 2021, 407, 126799.

[8] F. Dattila , R. R. Seemakurthi , Y. Zhou , N. López , Chem. Rev. 2022, 122, 11085. 35476402 10.1021/acs.chemrev.1c00690

[9] D. Kopač , B. Likozar , M. Huš , ACS Catal. 2020, 10, 4092. 32953235 10.1021/acscatal.9b05303 PMC7493227

[10] A. R. Woldu , Z. Huang , P. Zhao , L. Hu , D. Astruc , Coord. Chem. Rev. 2022, 454, 214340.

[11] G. M. Tomboc , S. Choi , T. Kwon , Y. J. Hwang , K. Lee , Adv. Mater. 2020, 32, 1908398. 10.1002/adma.201908398 32134526

[12] M. J. Palys , P. Daoutidis , Comput. Chem. Eng. 2022, 165, 107948.

[13] R. Daiyan , I. MacGill , R. Amal , ACS Enery Lett. 2020 5, 3843.

[14] F. Ausfelder , H. E. Dura , *DECHEMA, Gesellschaft für Chemische Technik und Biotechnologie* 2018, 1.

[15] Y. Hori , I. Takahashi , O. Koga , N. Hoshi , J. Mol. Catal. A: Chem. 2003, 199, 39.

[16] Y. Hori , K. Kikuchi , A. Murata , S. Suzuki , Chem. Lett. 1986, 15, 897.

[17] Y. Hori , A. Murata , R. Takahashi , J. Chem. Soc., Faraday Trans. 1: Physical Chemistry in Condensed Phases 1989, 85, 2309.

[18] M. König , J. Vaes , E. Klemm , D. Pant , iscience 2019, 19, 135. 31369986 10.1016/j.isci.2019.07.014 PMC6669325

[19] C. Deacon‐Price , A. H. da Silva , C. S. Santana , M. T. Koper , A. C. Garcia , J. Phys. Chem. C 2023, 127, 14518. 10.1021/acs.jpcc.3c03257 PMC10388345 37529666

[20] P. Sebastián‐Pascual , S. Mezzavilla , I. E. Stephens , M. Escudero‐Escribano , ChemCatChem 2019, 11, 3626.

[21] X. Nie , W. Luo , M. J. Janik , A. Asthagiri , J. Catal. 2014, 312, 108.

[22] J. Santatiwongchai , K. Faungnawakij , P. Hirunsit , ACS Catal. 2021, 11, 9688.

[23] Y. Du , W. An , J. Phys. Chem. C 2021, 125, 9138.

[24] M. D. Hossain , Y. Huang , T. H. Yu , W. A. Goddard III , Z. Luo , Nat. Commun. 2020, 11, 2256. 32382033 10.1038/s41467-020-16119-6 PMC7205999

[25] R. Gholizadeh , M. Pavlin , B. Likozar , M. Huš , ChemPlusChem 2024, 90, e202400346. 39561256 10.1002/cplu.202400346 PMC11826130

[26] T. Cheng , H. Xiao , W. A. Goddard III , J. Am. Chem. Soc. 2016 138, 13802. 27726392 10.1021/jacs.6b08534

[27] M. Zeng , W. Fang , Y. Cen , X. Zhang , Y. Hu , B. Y. Xia , Angew. Chem. 2024, 136, e202404574. 10.1002/anie.202404574 38638104

[28] X. Qin , R. Sechi , H. A. Hansen , Curr. Opin. Electrochem. 2024, 49, 101614.

[29] T. Mairegger , H. Li , C. Grießer , D. Winkler , J. Filser , N. G. Hörmann , K. Reuter , J. Kunze‐Liebhäuser , ACS Catal. 2023, 13, 5780. 37180961 10.1021/acscatal.3c00236 PMC10167651

[30] M. König , J. Vaes , D. Pant , E. Klemm , J. Phys. Chem. C 2023, 127, 18159.

[31] Á. Díaz‐Duque , A. P. Sandoval‐Rojas , A. F. Molina‐Osorio , J. M. Feliu , M. F. Suárez‐Herrera , Electrochem. Commun. 2015, 61, 74.

[32] O. Kuntyi , G. Zozulya , M. Shepida , J. Chem. 2022, 2022, 1306688.

[33] S. Ikeda , T. Takagi , K. Ito , Bull. Chem. Soc. Jpn. 1987, 60, 2517.

[34] G. Marcandalli , M. C. Monteiro , A. Goyal , M. T. Koper , Acc. Chem. Res. 2022, 55, 1900. 35772054 10.1021/acs.accounts.2c00080 PMC9301915

[35] R. Gholizadeh , M. Pavlin , M. Huš , B. Likozar , ChemSusChem 2025, 18, e202400898. 39022871 10.1002/cssc.202400898 PMC11696222

[36] J. Hussain , H. Jónsson , E. Skúlason , ACS Catal. 2018, 8, 5240.

[37] M. Van den Bossche , C. Rose‐Petruck , H. Jonsson , J. Phys. Chem. C 2021, 125, 13802.

[38] J. Hussain , E. Skúlason , H. Jónsson , Procedia Comput. Sci. 2015, 51, 1865.

[39] K. J. P. Schouten , Y. Kwon , C. J. M. Van Der Ham , Z. Qin , M. T. M. Koper , Chem. Sci. 2011, 2, 1902.

[40] Y. Jiang , K. Mao , J. Li , D. Duan , J. Li , X. Wang , Y. Zhong , C. Zhang , H. Liu , W. Dong , R. Long , Y. Xiong , ACS nano 2023, 17, 2620. 36715316 10.1021/acsnano.2c10534

[41] A. A. Peterson , F. Abild‐Pedersen , F. Studt , J. Rossmeisl , J. K. Nørskov , Energy Environ. Sci. 2010, 3, 1311.

[42] Y. Hori , H. Wakebe , T. Tsukamoto , O. Koga , Electrochim. Acta 1994, 39, 1833.

[43] K. P. Kuhl , E. R. Cave , D. N. Abram , T. F. Jaramillo , Energy Environ. Sci. 2012, 5, 7050.

[44] A. J. Garza , A. T. Bell , M. Head‐Gordon , ACS Catal. 2018, 8, 1490.

[45] F. Scholten , K. L. C. Nguyen , J. P. Bruce , M. Heyde , B. Roldan Cuenya , Angew. Chem. Int. Ed. 2021, 60, 19169. 10.1002/anie.202103102 PMC8457179 34019726

[46] T. Kawawaki , T. Okada , D. Hirayama , Y. Negishi , Green Chem. 2024, 26, 122.

[47] X. Cai , G. Li , W. Hu , Y. Zhu , ACS Catal. 2022, 12, 10638.

[48] Y. Zang , S. Wang , J. Sang , P. Wei , X. Zhang , Q. Wang , G. Wang , Nano Letters. 2024, 24, 7261. 10.1021/acs.nanolett.4c01239 38856118

[49] R. Kas , R. Kortlever , H. Yılmaz , M. T. Koper , G. Mul , ChemElectroChem 2015, 2, 354.

[50] D. Kim , C. S. Kley , Y. Li , P. Yang , Proc. Natl. Acad. Sci. 2017, 114, 10560. 28923930 10.1073/pnas.1711493114 PMC5635920

[51] A. G. Nabi , A. Hussain , G. A. Chass , D. Di Tommaso , Nanomaterials 2022, 13, 87. 36615997 10.3390/nano13010087 PMC9823659

[52] Y. F. Bu , M. Zhao , G. X. Zhang , X. Zhang , W. Gao , Q. Jiang , ChemElectroChem 2019, 6, 1831.

[53] W. Rong , H. Zou , W. Zang , S. Xi , S. Wei , B. Long , J. Hu , Y. Ji , L. Duan , Angew. Chem. Int. Ed. 2021, 60, 466. 10.1002/anie.202011836 32946193

[54] X. Zhang , J. X. Liu , B. Zijlstra , I. A. Filot , Z. Zhou , S. Sun , E. J. Hensen , Nano Energy 2018, 43, 200.

[55] L. J. Liu , Z. Y. Wang , Z. Y. Wang , R. Wang , S. Q. Zang , T. C. Mak , Angew. Chem. 2022, 134, e202205626. 10.1002/anie.202205626 35672885

[56] E. Sedano Varo , R. Egeberg Tankard , J. Kryger‐Baggesen , J. Jinschek , S. Helveg , I. Chorkendorff , C. D. Damsgaard , J. Kibsgaard , J. Am. Chem. Soc. 2024, 146, 2015. 38196113 10.1021/jacs.3c10610 PMC10811675

[57] Q. J. Wu , D. H. Si , P. P. Sun , Y. L. Dong , S. Zheng , Q. Chen , S.‐H. Ye , D. Sun , R. Cao , Y. B. Huang , Angew. Chem. 2023, 135, e202306822. 10.1002/anie.202306822 37468435

[58] X. Su , Z. Jiang , J. Zhou , H. Liu , D. Zhou , H. Shang , X. Ni , Z. Peng , F. Yang , W. Chen , Z. Qi , D. Wang , Y. Wang , Nat. Commun. 2022, 13, 1322. 35277523 10.1038/s41467-022-29035-8 PMC8917205

[59] H. Xu , D. Rebollar , H. He , L. Chong , Y. Liu , C. Liu , C.‐J. Sun , T. Li , J. V. Muntean , R. E. Winans , D.‐J. Liu , T. Xu , Nat. Energy 2020, 5, 623.

[60] J. Timoshenko , C. Rettenmaier , D. Hursán , M. Rüscher , E. Ortega , A. Herzog , T. Wagner , A. Bergmann , U. Hejral , A. Yoon , A. Martini , E. Liberrs , M. C.d.O. Monteiro , B. R. Cuenya , Nat. Commun. 2024, 15, 6111. 39030207 10.1038/s41467-024-50379-w PMC11271611

[61] R. Amirbeigiarab , J. Tian , A. Herzog , C. Qiu , A. Bergmann , B. Roldan Cuenya , O. M. Magnussen , Nat. Catal. 2023, 6, 837.

[62] C. Liu , B. Yang , E. Tyo , S. Seifert , J. DeBartolo , B. von Issendorff , P. Zapol , S. Vajda , L. A. Curtiss , J. Am. Chem. Soc. 2015, 137, 8676. 26115184 10.1021/jacs.5b03668

[63] M. Szalay , D. Buzsáki , J. Barabás , E. Faragó , E. Janssens , L. Nyulászi , T. Höltzl , Phys. Chem. Chem. Phys. 2021, 23, 21738. 34549207 10.1039/d1cp02220b

[64] R. K. Raju , P. Rodriguez , R. L. Johnston , J. Phys. Chem. C 2019, 123, 14591.

[65] B. Barhács , E. Janssens , T. Höltzl , Phys. Chem. Chem. Phys. 2022, 24, 21417. 36047512 10.1039/d2cp01316a

[66] L. E. Gálvez‐González , J. O. Juárez‐Sánchez , R. Pacheco‐Contreras , I. L. Garzón , L. O. Paz‐Borbón , A. Posada‐Amarillas , Phys. Chem. Chem. Phys. 2018, 20, 17071. 29896596 10.1039/c8cp00818c

[67] Megha , K. Mondal , T. K. Ghanty , A. Banerjee , J. Phys. Chem. A 2021, 125, 2558. 33728907 10.1021/acs.jpca.1c00751

[68] S. M. Lang , T. M. Bernhardt , Phys. Chem. Chem. Phys. 2012, 14, 9255. 22669249 10.1039/c2cp40660h

[69] S. M. Lang , I. Fleischer , T. M. Bernhardt , R. N. Barnett , U. Landman , ACS Catal. 2015, 5, 2275.

[70] Q. Zhao , E. A. Carter , J. Chem. Theory Comput. 2020, 16, 6528. 32816491 10.1021/acs.jctc.0c00583

[71] M. Kabir , A. Mookerjee , A. K. Bhattacharya , The European Physical Journal D‐Atomic, Molecular, Optical and Plasma Physics 2004, 31, 477.

[72] P. Jaque , A. Toro‐Labbé , J. Chem.Phys. 2002, 117, 3208.

[73] U. J. Rangel‐Pena , R. L. Camacho‐Mendoza , S. González‐Montiel , L. Feria , J. Cruz‐Borbolla , J. Cluster Sci. 2021, 32, 1155.

[74] J. Garrido‐Aldea , M. P. de Lara‐Castells , Phys. Chem. Chem. Phys. 2022, 24, 24810. 36196765 10.1039/d2cp02169b

[75] H. Guo , P. Poths , P. Sautet , A. N. Alexandrova , ACS Catal. 2021, 12, 818.

[76] D. T. Whipple , P. J. Kenis , J. Phys. Chem. Lett. 2010, 1, 3451.

[77] F. Li , Q. Tang , J. Catal. 2020, 387, 95.

[78] Q. Tang , Y. Lee , D. Y. Li , W. Choi , C. W. Liu , D. Lee , D. E. Jiang , J. Am. Chem. Soc. 2017, 139, 9728. 28640611 10.1021/jacs.7b05591

[79] N. P. Fitzsimons , W. Jones , P. J. Herley , J. Chem. Soc., Faraday Trans. 1995, 91, 713.

[80] S. Yang , D. Rao , J. Ye , S. Yang , C. Zhang , C. Gao , X. Zhou , H. Yang , X. Yan , Int. J. Hydrogen Energy 2021, 46, 3484.

[81] J. H. Stenlid , A. J. Johansson , L. Kloo , T. Brinck , J. Phys. Chem. C 2016, 120, 1977.

[82] H. A. Hansen , J. H. Montoya , Y. J. Zhang , C. Shi , A. A. Peterson , J. K. Nørskov , Catal. Lett. 2013, 143, 631.

[83] A. Vargas‐Caamal , J. L. Cabellos , F. Ortiz‐Chi , H. S. Rzepa , A. Restrepo , G. Merino , Chem. – Eur. J. 2016, 22, 2812. 26774026 10.1002/chem.201504016

[84] D. E. García‐Rodríguez , L. H. Mendoza‐Huizar , C. Díaz , Appl. Surf. Sci. 2017, 412, 146.

[85] D. Buceta , S. Huseyinova , M. Cuerva , H. Lozano , L. J. Giovanetti , J. M. Ramallo‐López , P. López‐Caballero , A. Zanchet , A. O. Mitrushchenkov , A. W. Hauser , G. Barone , C. Huck‐Iriart , C. Escudero , J. C. Hernández‐Garrido , J. J. Calvino , M. López‐Haro , M. P. de Lara‐Castells , F. G. Requejo , M. A. López‐Quintela , Chem. Eur. J. 2023, 29, e202301517. 37204268 10.1002/chem.202301517 PMC10946568

[86] P. López‐Caballero , A. W. Hauser , M. Pilar de Lara‐Castells , J. Phys. Chem. C 2019, 123, 23064. 10.1021/acs.jpcc.9b06620 PMC6777821 31598186

[87] J. Zhao , D. Liu , F. Wei , W. F. Ip , H. Pan , S. Lin , Nano Res. 2023, 16, 9091.

[88] J. Wang , G. Wang , J. Zhao , Chem. Phys. Lett. 2003, 380, 716.

[89] F. Calle‐Vallejo , Adv. Sci. 2023, 10, 2207644. 10.1002/advs.202207644 PMC10369287 37102632

[90] M. Guba , T. Höltzl , J. Phys. Chem. C 2024, 128, 4677. 10.1021/acs.jpcc.3c06475 PMC10961840 38533239

[91] N. Oppel , P. Röse , S. Heuser , M. Prokein , U. P. Apfel , U. Krewer , Electrochim. Acta 2024, 490, 144270.

[92] S. Ringe , Nat. Commun. 2023, 14, 2598. 37147278 10.1038/s41467-023-37929-4 PMC10162986

[93] H. Zhou , W. Xi , P. Yang , H. Huang , J. Tian , M. Ratova , D. Wu , J. Energy Chem. 2024, 99, 201.

[94] C. Y. J. Lim , M. Yilmaz , J. M. Arce‐Ramos , A. D. Handoko , W. J. Teh , Y. Zheng , Z. H. J. Khoo , M. Lin , M. Isaacs , T. L. D. Tam , Y. Bai , C. K. Ng , B. S. Yeo , G. Sankar , I. P. Parkin , K. Hippalgaonkar , M. B. Sullivan , J. Zhang , Y. F. Lim , Nat. Commun. 2023, 14, 335. 36670095 10.1038/s41467-023-35912-7 PMC9860078

[95] H. Xiao , W. A. Goddard III , T. Cheng , Y. Liu , Proc. Natl. Acad. Sci. 2017, 114, 6685. 28607069 10.1073/pnas.1702405114 PMC5495255

[96] A. Akhuli , A. Mahanty , D. Chakraborty , J. R. Biswal , M. Sarkar , J. Phys. Chem. C 2024, 128, 15380.

[97] A. V. Rudnev , U. E. Zhumaev , A. Kuzume , S. Vesztergom , J. Furrer , P. Broekmann , T. Wandlowski , Electrochim. Acta 2016, 189, 38.

[98] G. Kastlunger , P. Lindgren , A. A. Peterson , J. Phys. Chem. C 2018, 122, 12771.

[99] S. Chandrashekar , H. P. I. van Montfort , D. Bohra , G. Filonenko , H. Geerlings , T. Burdyny , W. A. Smith , Nanoscale 2022, 14, 14185. 36124967 10.1039/d2nr03438g

[100] A. Klamt , G. J. G. J. Schüürmann , J. Chem. Soc., Perkin Trans. 1993, 2, 799.

[101] E. Epifanovsky , A. T. Gilbert , X. Feng , J. Lee , Y. Mao , N. Mardirossian , P. Pokhilko , A. F. White , M. P. Coons , A. L. Dempwolff , Z. Gan , D. Hait , P. R. Horn , L. D. Jacobson , I. Kaliman , J. Kussmann , A. W. Lange , K. U. Lao , D. S. Levine , J. Liu , S. C. McKenzie , A. F. Morrison , K. D. Nanda , F. Plasser , D. R. Rehn , M. L. Vidal , Z.‐Q. You , Y. Zhu , B. Alam , B. J. Albrecht , et al., J. Chem. Phys., 2021, 155, 084801.34470363

[102] L. Martínez , R. Andrade , E. G. Birgin , J. M. Martínez , J. Comput. Chem. 2009, 30, 2157. 19229944 10.1002/jcc.21224

[103] S. Spicher , S. Grimme , Angew. Chem. Int. Ed. 2020, 59, 15665. 10.1002/anie.202004239 PMC7267649 32343883

[104] C. Bannwarth , E. Caldeweyher , S. Ehlert , A. Hansen , P. Pracht , J. Seibert , S. Spicher , S. Grimme , Wiley Interdiscip. Rev.:Comput. Mol. Sci. 2021, 11, e1493.

[105] J. J. Mortensen , A. H. Larsen , M. Kuisma , A. V. Ivanov , A. Taghizadeh , A. Peterson , K. S. Thygesen , A. O. Dohn , C. Schäfer , E. Ö. Jónsson , E. D. Hermes , F. A. Nilsson , G. Kastlunger , G. Levi , H. Jónsson , H. Häkkinen , J. Fojt , J. Kangsabanik , J. Sødequist , J. Lehtomäki , J. Heske , J. Enkovaara , K. T. Winther , M. Dulak , M. M. Melander , M. Ovesen , M. Louhivuori , M. Walter , M. Gjerding , O. Lopez‐Acevedo , et al., J. Chem. Phys. 2024, 160, 092503.38450733

[106] H. Shen , Z. Gu , G. Zheng , Sci. Bull. 2019, 64, 1805. 10.1016/j.scib.2019.08.027 36659577

[107] C. Liu , H. He , P. Zapol , L. A. Curtiss , Phys. Chem. Chem. Phys. 2014, 16, 26584. 25158148 10.1039/c4cp02690j

[108] F. B. Van Duijneveldt , J. G. van Duijneveldt‐van de Rijdt , J. H. van Lenthe , Chem. Rev. 1994, 94, 1873.

[109] A. Stukowski , Modell. Simul. Mater. Sci. Eng. 2009, 18, 015012.

[110] T. Bah , Inkscape: Guide to a Vector Drawing Program, Prentice Hall pPress, Englewood Cliffs, NJ 2011

