# Supplementary material for: Unveiling the Role of Low‐Coordinated Sites in CO2 Electroreduction Using Hierarchical Simulation Models
Source: Chempluschem. 2025 Jul 8;90(8):e202500223. doi: 10.1002/cplu.202500223 (PMC12352728; doi:10.1002/cplu.202500223)
Supplement: Supplementary file 1 — Supplementary Material [file CPLU-90-e202500223-s001.pdf]

UNVEILING THE ROLE OF LOW-COORDINATED SITES IN CO_2 ELECTROREDUCTION USING HIERARCHICAL SIMULATION MODELS

Supporting Information

Ádám Haffner, Tibor Höltzl

Contents

Method testing in Q-Chem 2

Model testing in GPAW 7

Building of the 'nanodroplet' model 8

Building of the surface deposited cluster model 10

Investigation of the CO_2 reduction mechanism 12

Investigation of CO_2 adsorption mechanism on $Cu_6[H_2O]_{0-6}$ 13

Energy Decomposition Analysis in $Cu_6[H_2O]_0$*CO_2 and $Cu_6[H_2O]_0$*H_2O 14

EDA analysis investigating different binding modes of H_2O on $Cu_6[H_2O]_6$ 15

Analysis of non-covalent interactions and voltage dependency of the product of CO_2 reduction on $Cu_6[H_2O]_6$ 15

Investigation of $Cu_6[H_2O]_{35}$ without directly attached explicit water molecules 17

Additional analysis for the surface deposited cluster 22

Reaction path of the surface deposited cluster starting with the adsorption of CO_2 in a reduced form of *HCOO 24

Linear correction of the reaction free energies obtained by using different DFT methods in case of the surface deposited cluster 24

Tendencies in the effect of copper coordination 26

XYZ-coordinates of all structures presented in this article (in Å) 27

Reaction paths of $Cu_6[H_2O]_0$ 27

Reaction paths of $Cu_6[H_2O]_1$ 34

Reaction paths of $Cu_6[H_2O]_2$ 43

Reaction paths of $Cu_6[H_2O]_6$ 51

Structures with explicite water molecules attached to the optimized structures 73

$Cu_6[H_2O]_{35}$ without directly attached explicit water molecules 80

$Cu_6[H_2O]_{35}$ (with directly attached explicit water molecules) 107

Gr-$Cu_6[H_2O]_{25}$ 158

Structures for investigating low-coordination sites 285

Method testing in Q-Chem

We selected five model systems for method testing that represent different cluster-adsorbate configurations, including various structures of the Cu_6 cluster and the water-water dimer (Figure S1).

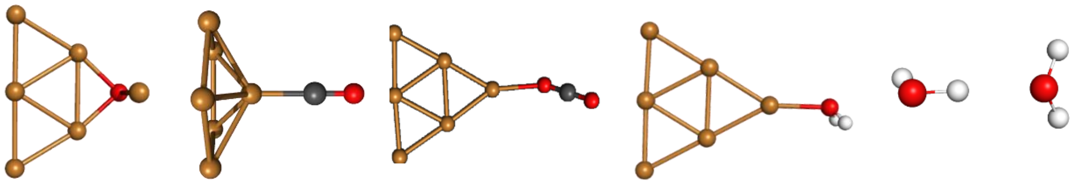

Figure S1: The model systems used for benchmarking the computational method

Basis set selection

We computed the reaction energies with different basis sets using the B3LYP functional and compared to those obtained using def2-QZVPPD, what should provide reaction energies close to the basis set limit. For calculating the reaction energies, we used the Computational Hydrogen Electrode (CHE) method with the following equations.

$$\Delta E(\ ^{*}O) = E(Cu_6\ ^{*}O) + E(H_2O) + E(CH_4) - E(Cu_6) - E(CO_2) - 6 \cdot \frac{1}{2} \cdot E(H_2) \quad (1)$$

$$\Delta E(\ ^{*}CO) = E(Cu_6\ ^{*}CO) + E(H_2O) - E(Cu_6) - E(CO_2) - 2 \cdot \frac{1}{2} \cdot E(H_2) \quad (2)$$

$$\Delta E(\ ^{*}CO_2) = E(Cu_6\ ^{*}CO_2) - E(Cu_6) - E(CO_2) \quad (3)$$

$$\Delta E(\ ^{*}H_2O) = E(Cu_6\ ^{*}H_2O) - E(Cu_6) - E(H_2O) \quad (4)$$

$$\Delta E(H_2O - H_2O) = E(H_2O - H_2O) - 2 \cdot E(H_2O) \quad (5)$$

We ranked the different basis sets according to the absolute differences of the computed reaction energies compared to the reference (Table S1 and Figure S2).

Table S1: Difference in absolute reaction energies in case of the different basis sets in kJ/mol from those obtained using def2-QZVPPD. Functional: B3LYP

	LANL2DZ	LANL2TZ (f)	def2-SVP	def2-TZVP	def2-QZVP	def2-TZVPD	def2-TZVPP	def2-QZVPPD
Absolute difference (kJ/mol) $(\sqrt{\sum(\Delta(\Delta E))^2})$	23	18	18	12	5	12	11	0

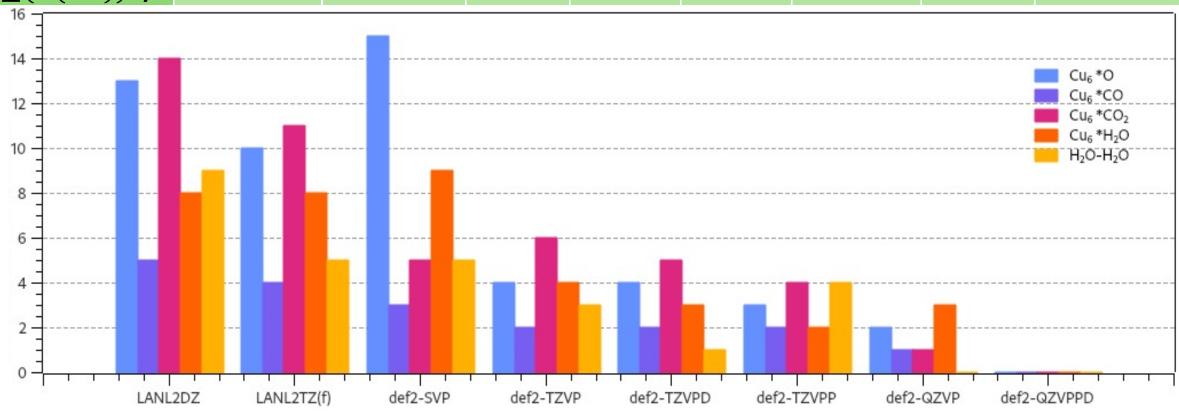

Figure S2: Difference in absolute reaction energies in case of the different basis sets in kJ/mol from those obtained using def2-QZVPPD. Functional: B3LYP

As expected, def2-QZVP showed the best agreement, however, taking the computation times into account we finally selected the def2-TZVP basis set, which provides a reasonable accuracy with modest computational time. In general, the different functionals show similar basis set dependence, thus we applied this basis set for all Q-Chem computations.

Table S3: Reaction energies computed using the CCSD(T)/def2-QZVPPD//ωB97X-D/def2-TZVP method

	Cu_6*O	Cu_6*CO	Cu_6*CO_2	Cu_6*H_2O	H_2O-H_2O
ΔE (kJ/mol)	-58	-8	-17	-57	-19

Functional selection

To assess the accuracy of the various functionals, we compared the reaction energies obtained from optimizations with those derived from the CCSD(T)/def2-QZVPPD method at the ωB97X-D/def2-TZVP geometries (Table S4 and Figure S3).

Table S4: Difference in absolute reaction energies in case of the different functionals in kJ/mol from those obtained using CCSD(T)/def2-QZVPPD// ωB97X-D/def2-TZVP. Basis set: def2-TZVP

	B3LYP	ωB97X-D	ωB97X-V	BP86	BP86-D3	BP86-D4
Absolute difference (kJ/mol) ($\sqrt{\sum(\Delta(\Delta E))^2}$)	13	5	7	9	7	7
	**CAM-B3LYP**	**TPSSh**	**HSE-HJS**	**VV10**	**ωB97M-V**	**CCSD(T)**
Absolute difference (kJ/mol) ($\sqrt{\sum(\Delta(\Delta E))^2}$)	19	6	11	13	10	0

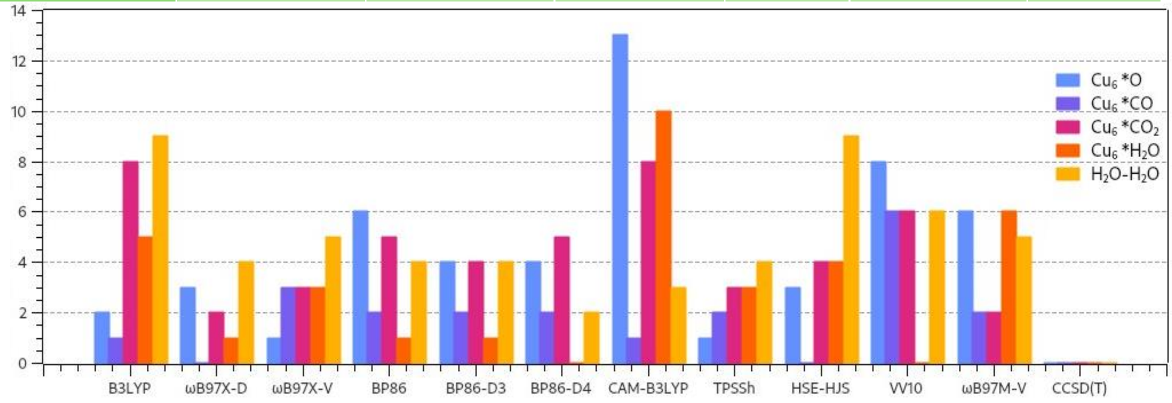

Figure S3: Absolute differences of reaction energies obtained with different functionals (in kJ/mol) compared to CCSD(T)/def2-QZVPPD// ωB97X-D/def2-TZVP.

The best agreement was observed in the ωB97X-D functional, thus we selected this functional to use in the computations. The same functional was selected by Raju Rajesh Kumar et al. [63]

Thus, we performed the Q-Chem simulations using the ωB97X-D/def2-TZVP level of theory.

Implicit solvent models

We also benchmarked the various implicit solvent models on the different model systems except for the water-water dimer. The reaction energies are listed in Table S4.

Table S4: Reaction energies with different implicit solvent models (ωB97X-D/def2-TZVP)

		SMD	SM12	CPCM	COSMO
Cu_6*O	(ΔE) (kJ/mol)	-55	-57	-57	-53
Cu_6*CO	(ΔE) (kJ/mol)	-12	-10	-11	-10
Cu_6*CO_2	(ΔE) (kJ/mol)	-22	-20	-21	-22
Cu_6*H_2O	(ΔE) (kJ/mol)	-42	-41	-43	-43

There are small differences between the different solvent models, thus we selected the CPCM, since analytical second derivatives of the energies with respect to the nuclear coordinates were available in Q-Chem for this implicit solvent model.

In solvent phase free external translation and rotation of the molecules are not allowed, thus using the same statistical thermodynamical model as in gas phase would lead to a systematic error. Thus, according to the suggestion of Ohnishi et al. we excluded the external translational and rotational contributions in the thermochemical computations. [109] Simulating a single water molecule in implicit solvent, the solvation free energy without any translational and rotational adjunct gives -29 kJ/mol using the CPCM solvent model, whilst the reference value is -26 kJ/mol. [110] It is also important to point out that we can expect an error compensation when comparing different reaction paths. We considered carbon dioxide, methane and hydrogen in gas phase, so this error might influence most the reactant adsorption and the product desorption.

Attaching explicit water molecules to optimized adducts

In the work of Kumar et al. [63] several CO_2 reaction paths were presented in implicit solvent. We selected one of these reaction paths that was leading to the formation of methane. This path was labelled as one of the most important reaction path in the CO_2RR on Cu_6 in ref. 56. We reoptimized the structures of the path with our tested method, (black path on Figure S4) afterwards we systematically attached explicit water molecules onto all copper atoms of the reoptimized structures and selected the thermodynamically most stable structures of them. Reaction free energies of these structures can be compared on Figure S4 by one and two explicit water molecules with the structure without any explicit water molecule attached.

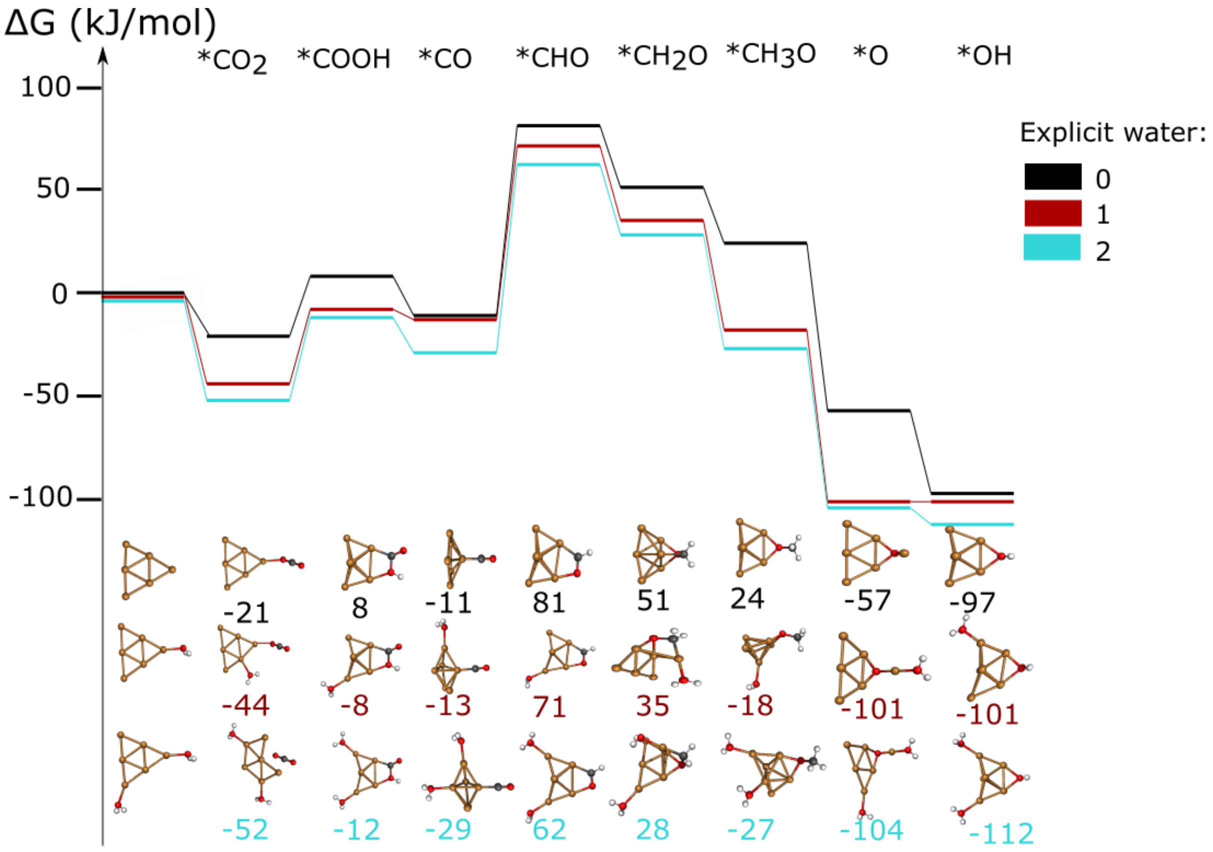

Figure S4: The effect of attaching explicit water molecules to different Cu_6-adducts

These reaction paths showed similar patterns with the *CO-*CHO reduction step having the largest positive change in reaction free energy. The results also showed that whilst the first explicit water molecule always adsorbed to the copper atom having the most positive charge prior to the adsorption, (except the cases where an *OH group or a carbon-containing fragment was already bound to the most positive copper atom) by the second water molecule it was not always the case. The triangular shaped cluster had three of such atoms, whilst the other common cluster type had only two. This is in line with the solvent induced re-organization of the preferred cluster shape.

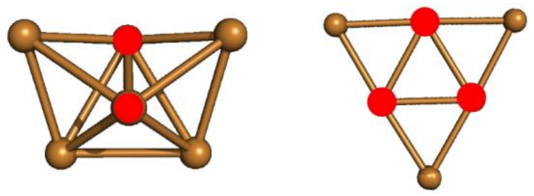

Figure S5: The copper atoms of Cu_6 (highlighted as red) which are less preferentially form bonds with the adsorbate

GPAW model benchmarks

Functional and basis set

We applied the PBE functional in conjunction with the Grimme-D3 dispersion correction and the TZP basis set for geometry optimizations and PBE-D3/DZP for molecular dynamics simulations using the GPAW code. We investigated the accuracy of the method on the same systems that we used for Q-Chem benchmarks (Table S5).

Table S5: Reaction energies as computed using equations 1-5, computed using GPAW (in kJ/mol)

Structure	a*	b*	c*	d*	e*
Cu_6*CO_2	-16	-12	-19	-18	-21
Cu_6*CO	-40	-39	-8	-11	-30
Cu_6*H_2O	-65	-74	-56	-58	-61
Cu_6*O	-45	-41	-55	-48	-46
H_2O-H_2O	-20	-20	-23	-22	-20

*a. GPAW (PBE-D3 /TZP), b. GPAW (PBE-D3/DZP), c. Q-Chem (ωB97X-D /def2-TZVP), d. GPAW (PBE-D3/TZP), followed by single point in Q-Chem (ωB97X-D/def2-TZVP), e. GPAW (PW (cutoff: 1000 eV [111])

Time step and friction coefficient in molecular dynamics computation

We also selected the suitable time step for the molecular dynamics simulations in GPAW using the Velocity Verlet-algorithm for time-integration and without a thermostat (i.e. microcanonical ensemble). For the testing we used graphene surface deposited Cu_6 nanocluster with CO_2 adsorbed, surrounded by explicit water molecules (Table S6 and Figure S6). As a result, we selected a time step of 0.5 ps.

Apart from the time step, we also specified the friction coefficient used for the Langevin thermostat as 0.001 ps^{-1}, which performed well in keeping an approximately constant temperature of 298 K.

Table S6: The energy span during the 1 ps MD simulations by different time steps using NVE ensemble (Velocity Verlet)

Time step (fs)	1	0.9	0.8	0.7	0.6	0.5	0.4
Energy range (kJ/mol)	29	32	19	44	45	18	15

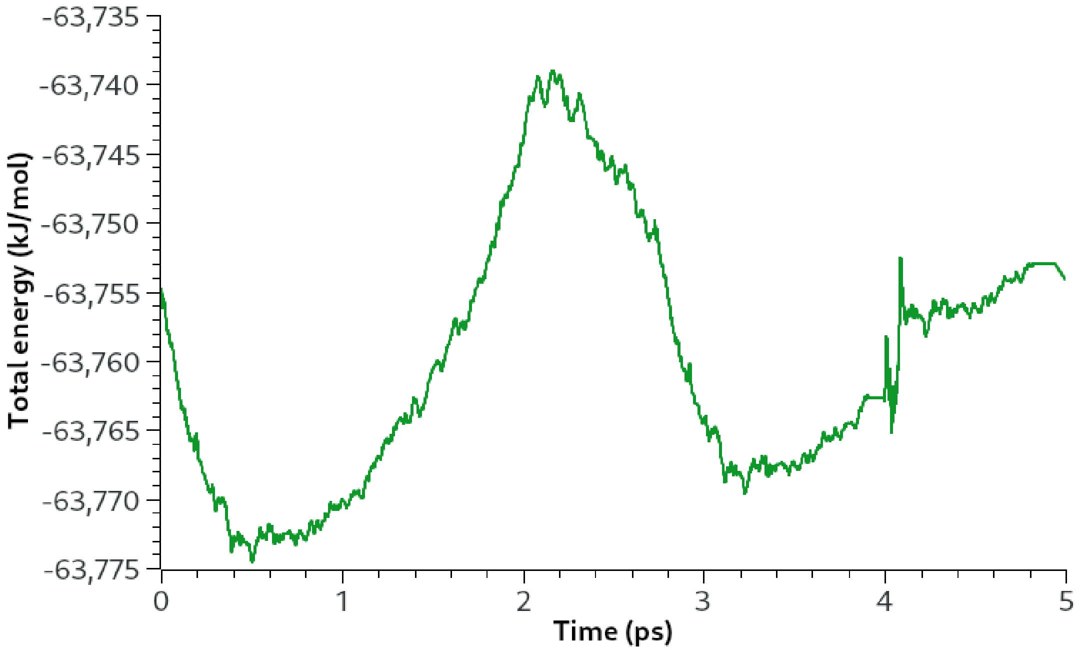

Figure S6: Total energy as a function of time using 0.5 ps time step

Building of the ’nanodroplet’ model

The ’nanodroplet’ model system was built using the Packmol software. [100] The most stable cluster, with six directly attached explicit water molecules was positioned in the centre of the cell and water molecules were added inside a sphere of 4.5 Å from the centre of the cluster. After generating the structure with the molecules, we pre-optimized using the GFN-FF force field [101] as implemented in the xTB program. [102] By choosing a total of 35 water molecules, we reached a system, in which the water molecules were densely arranged in the solvent shell, forming a compact structure. When an additional water molecule was added, it visibly started to build the second solvent shell, thus we applied a model system with 35 water molecules for the simulations.

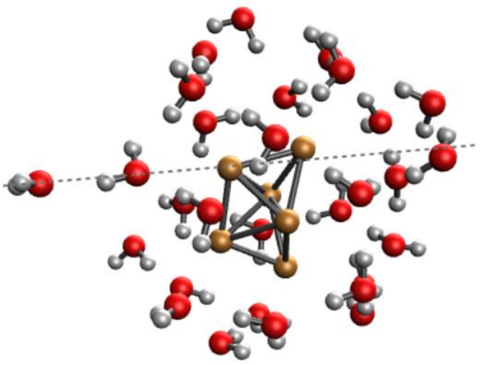

Figure S7: Structure of the cluster in the ’nanodroplet’ model with 36 explicit water molecules

We also set up a 'nanodroplet' model, where we solvated the $Cu_6[H_2O]_6$ cluster (with bound H_2O) with 29 water molecules around the cluster (Figure S8). The final structure had five directly attached water molecules, whilst one of the water molecules desorbed. This model yielded an energetically more stable structure compared to the one without bound water molecules. The free copper appeared to be the active site of the cluster for CO_2 adsorption. The main text focuses solely on the more stable form; however, our investigation of $Cu_6[H_2O]_{35}$, which does not include directly attached explicit water molecules, yielded interesting results that we present in the section with the title 'Investigation of $Cu_6[H_2O]_{35}$ without directly attached explicit water molecules' in the Supporting Information.

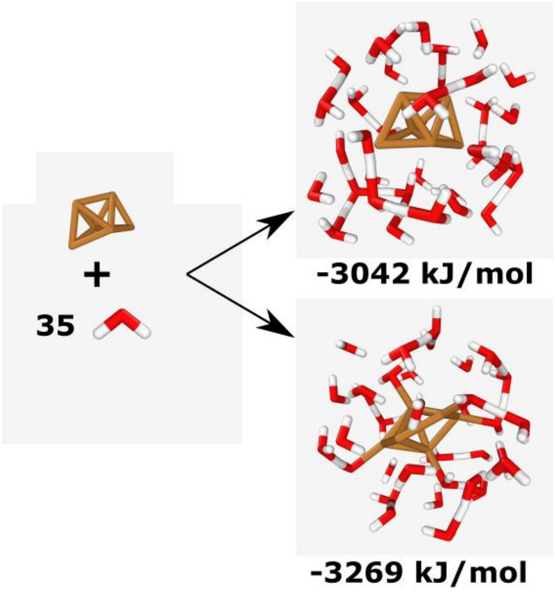

Figure S8: The $Cu_6[H_2O]_{35}$ cluster is more stable in the presence of directly adsorbed explicit water molecules

Construction of the surface deposited cluster model

For building the systems on the surface we started with the orthogonal cell of graphene.

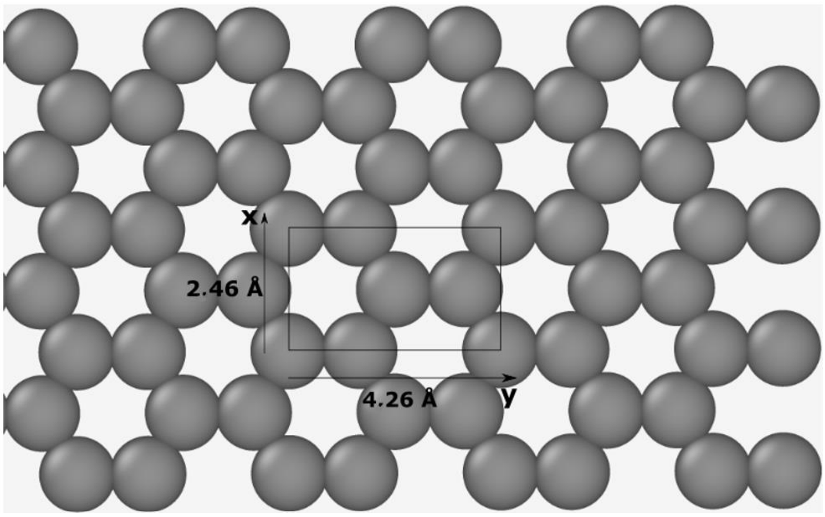

Figure S9: The orthogonal cell of graphene

We repeated the orthogonal cell four-times along the x-axis and twice along the y-axis to prepare a graphene supercell. From this supercell, we removed a carbon atom from the middle of the structure, creating a vacancy in the graphene layer, which provides a stronger interaction between the copper cluster and the graphene compared to perfect graphene layer.

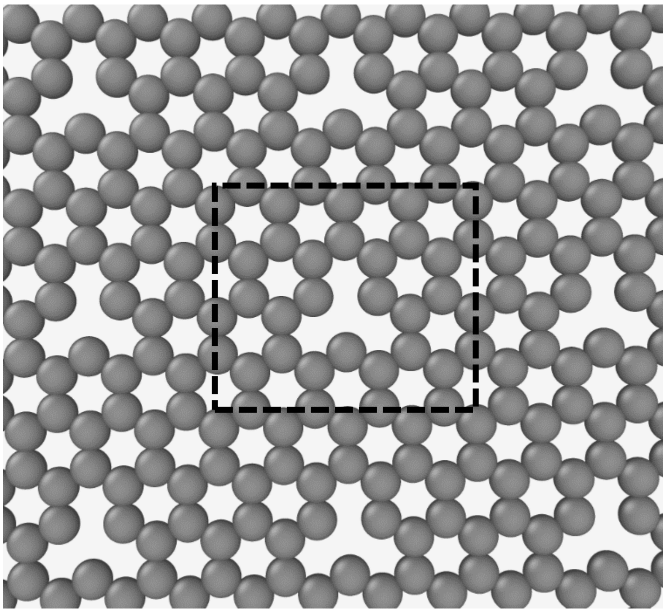

Figure S10: The graphene supercell used in simulations

In the next step, the cluster was positioned above the vacancy using the Atomic Simulation Environment. After this point, we added 25 explicit water molecules with Packmol to the system and performed pre-optimization with GFN-force field (GFN-FF) method in xTB software. The surface deposited system contained both directly bound water molecules and surrounding water molecules, forming an explicit solvent layer around the cluster. We

investigated mainly the structures where the hydrogen atom was added directly to an atom of the adsorbate, but we also investigated several cases where the hydrogen was added to one of the explicit water molecules in each step.

The solvent structure was investigated using NCI-analysis [112] on three neighbouring cells. The result showed that the interactions are dominated by the primary bonds between the metal atoms, however, when we carried out the simulation removing the surface and the cluster atoms, we could see that the hydrogen bonding network is spreading throughout the cells, thus with this model we could reach a coherent explicit water layer. Also, we can see that the hydrogen bonds and the explicit water structure have a wavy structure consistent with wavy geometry of the graphene surface in the model.

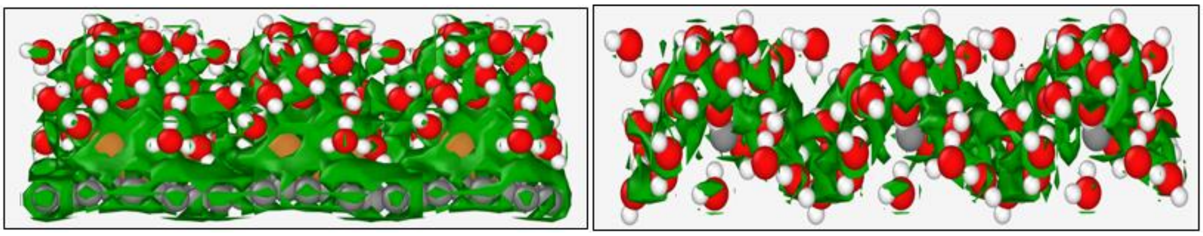

Figure S11: NCI-analysis of the surface deposited cluster and its explicit solvent shell with CO_2

We optimized the cell with various cell sizes in the non-periodic direction and we selected the one yielding to the lowest energy (Figure S12).

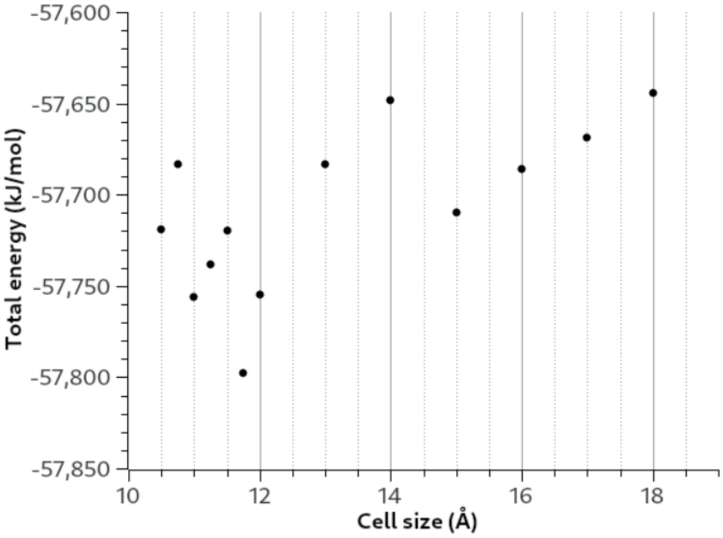

Figure S12: The dependence of the total energy of the system from the cell size by the surface deposited cluster model

As we can see the energy shows a minimum at 11.75 Å, however, there is a local minimum around 15 Å, which is due to a rearrangement of the water molecules at this cell size. According to these results, we used a cell size of 11.75 Å for the geometry optimizations. We investigated

the interaction energy between the neighbouring graphene layers without any substrate. This value appeared to be 7 kJ/mol. It is important to point out that these errors occur for all the investigated structures, thus, when calculating reaction free energies, an error compensation can be expected. Based on this concept, further measures have not been taken in order to prevent interaction between the neighbouring cells. The reported energies and free energies do not include dipole corrections. We investigated the range of dipole corrections and it appeared to be generally between 7-8 kJ/mol for the selected investigated cases (7 kJ/mol for * and *HCOOH, 8 kJ/mol for $*CO_2$, *HCOO, $*CH_2O$, CH_2OOH^-). This means that a similar error compensation can be expected as for the interaction of the neighbouring layers.

Afterwards we used the optimized geometry to determine the optimal number of *k*-points in the periodic axes (Table S8), while in the non-periodic direction we applied on *k*-point.

Table S8: Energies by different *k*-point grids

k_x	k_y	E (kJ/mol)	Difference[a] (kJ/mol)	k_x	k_y	E (kJ/mol)	Difference [a] (kJ/mol)
1	1	-63188	60	3	4	-63248	0
1	2	-63285	37	3	5	-63249	1
1	3	-63279	31	4	1	-63155	93
1	4	-63278	30	4	2	-63252	4
1	5	-63280	32	4	3	-63248	0
2	1	-63162	86	4	4	-63248	0
2	2	-63255	7	4	5	-63249	1
2	3	-63249	1	5	1	-63156	92
2	4	-63250	2	5	2	-63252	4
2	5	-63250	2	5	3	-63248	0
3	1	-63155	93	5	4	-63249	1
3	2	-63252	4	5	5	-63248	0
3	3	-63248	0	Reference (5·5·1)		-63248	0

[a] from the results with 5·5·1 k points.

Finally, we selected a *k*-point grid of 2·2·1, which led energies close to that using the 5·5·1 grid, but with a significantly reduced computational cost.

CO_2 reduction mechanism

We first bound the CO_2 to the small $Cu_6[H_2O]_x$ (x=0-6) cluster models, followed by the systematic, stepwise addition of hydrogen atoms to the carbon and oxygen atoms of the adsorbates, as well as to the copper atoms in the binding modes illustrated in Figure S13.

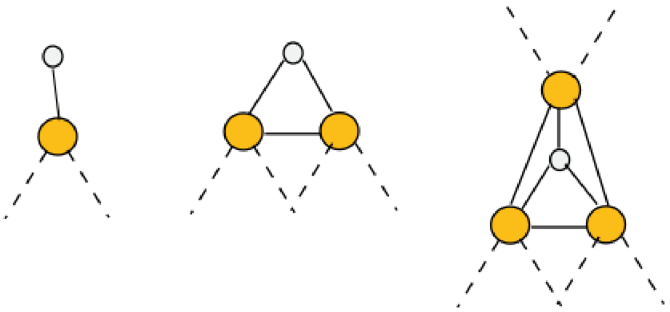

Figure S13: Schematic picture showing the investigated binding modes (yellow: copper, white: hydrogen)

As hydride formation on the cluster became unfavored with the increased number of water molecules, we bound hydrogens only to the adsorbate atoms of the 'nanodroplet' and the surface supported cluster models. For the nanodroplet models, we also constructed initial geometries in which hydrogen atom was attached to one of the water molecules on the droplet surface, and we subsequently followed the hydrogen transfer to the adsorbate via the Grotthuss mechanism.

Investigation of CO_2 adsorption mechanism on $Cu_6[H_2O]_{0-6}$

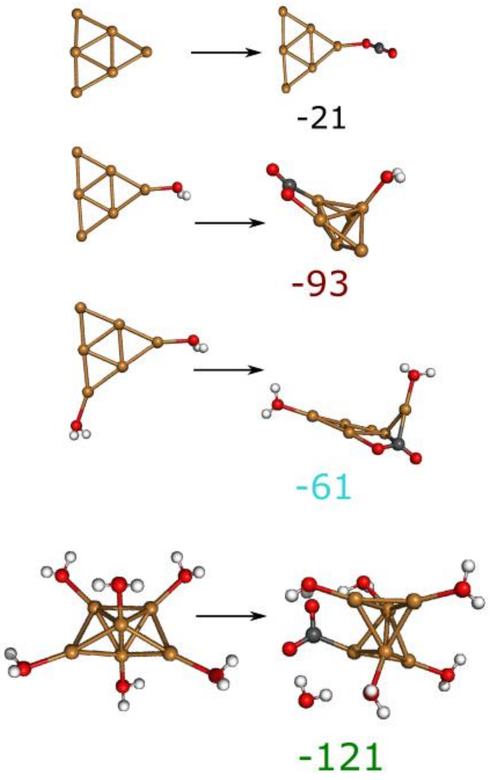

Figure S14: Formation free energy of $Cu_6[H_2O]_n$*CO_2 adducts from $Cu_6[H_2O]_n$ clusters

It is important to note that the reaction of the adsorbed CO_2 on the $Cu_6[H_2O]_{1-6}$ with one of the water molecules, leading to *HCOO and *OH is energetically favored. Therefore, the cluster facilitates the reduction of the adsorbed CO_2 (Figure S15).

a)

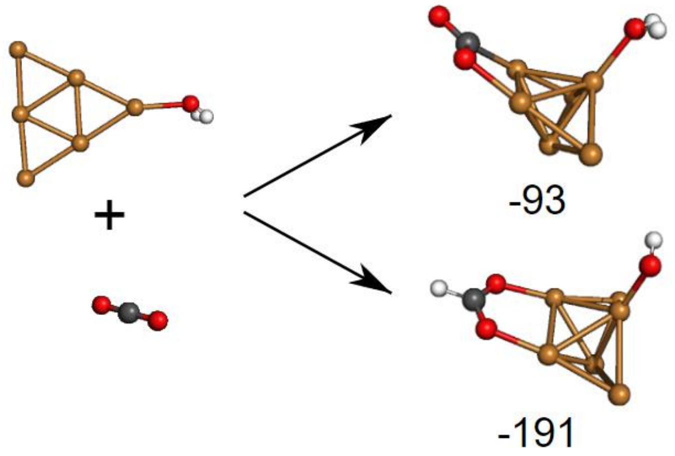

b)

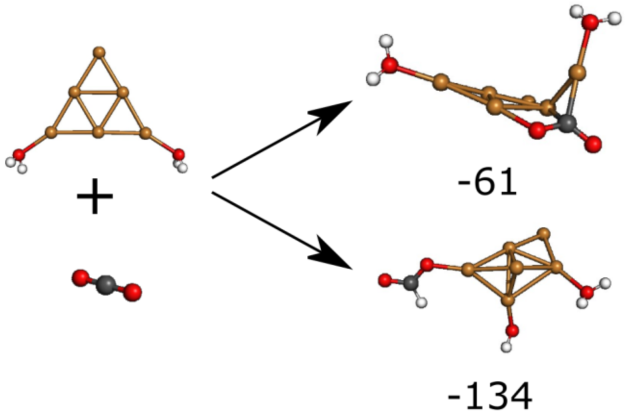

Figure S15 Spontaneous reduction of the adsorbed CO_2 with $Cu_6[H_2O]$ (a) and $Cu_6[H_2O]_2$ clusters

Energy Decomposition Analysis (EDA) in $Cu_6[H_2O]_0$*CO_2 and $Cu_6[H_2O]_0$*H_2O

Water binds more strongly to the cluster than CO_2, with binding free energies of -44 kJ/mol and -20 kJ/mol, respectively. The EDA-analysis showed that the most dominant term is the polarization, which is in line with the order of the interaction free energies.

Table S9: Results of EDA-analyses of Cu_6*CO_2 and Cu_6*H_2O adducts

	$*CO_2$	$*H_2O$
Interaction free energy (kJ/mol)	-20	-44
Polarization term (kJ/mol)	-14	-40
Charge transfer term (kJ/mol)	-9	-13
Electrostatic term (kJ/mol)	-65	-184
Dispersion term (kJ/mol)	-10	-16
Pauli repulsion (kJ/mol)	86	208
Solvation term (kJ/mol)	-3	3
Vibration term (kJ/mol)	-8	-5
Relaxation term (kJ/mol)	4	11
Wiberg bond index within the fragment	1.94	0.75
Wiberg bond index with the cluster	0.03	0.07

EDA analysis investigating different binding modes of H_2O on $Cu_6[H_2O]_6$

Table S9: EDA-analyses of different adducts of $Cu_6[H_2O]_6$

	$*OH_2$	*OH (regular)	*OH (bridge position)
Largest copper-oxygen bond index	0.08	0.33	0.24
Sum of copper-oxygen bond indices	0.12	0.44	0.49
Oxygen-hydrogen bond index	0.75	0.80	0.77
Charge of oxygen (a.u.)	-0.96	-1.17	-1.2
Interaction free energy (kJ/mol)	-55	-131	-406
Polarization term (kJ/mol)	-42	-69	-202
Charge transfer term (kJ/mol)	-13	-158	-649
Copper-oxygen distance (Å)	2.05	1.85	1.88

Analysis of non-covalent interactions and voltage dependence of the product of CO_2 reduction on $Cu_6[H_2O]_6$

We carried out a non-covalent interaction index (NCI) analysis on the $Cu_6[H_2O]_6*CO_2$ structure to unveil the non-covalent interactions formed due to the inclusion of explicit water molecules. The results are presented on Figure S16.

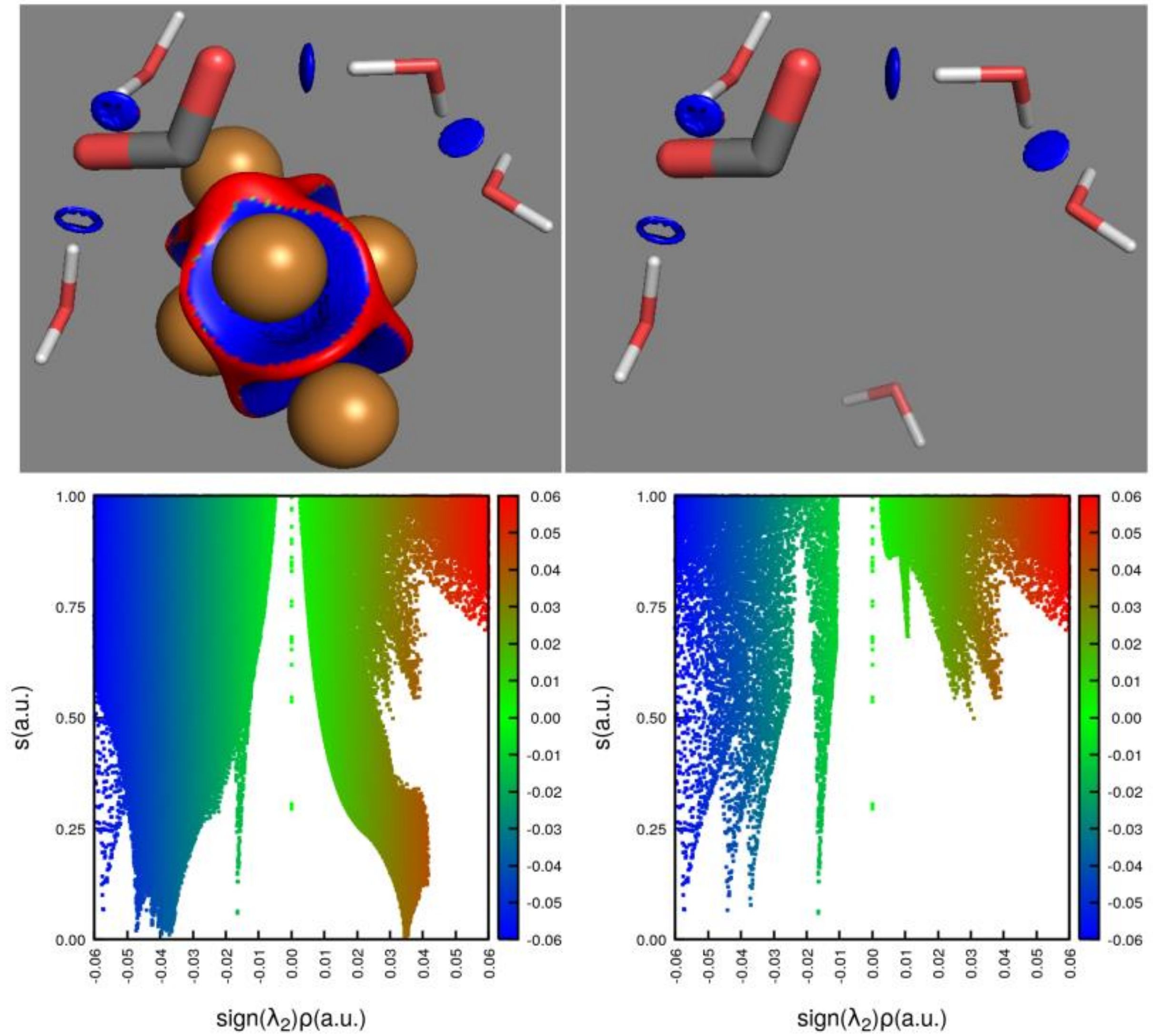

Figure S16: Non-covalent interaction index analysis carried out on $Cu_6[H_2O]_6$ *CO_2 (left) and on a system that does not contain the carbon atoms, only the water molecules and carbon dioxide (right)

The CHE model gives the opportunity to investigate the stability of $Cu_6[H_2O]_6$ *HCOO; n *OH adducts (n=1-5). For computing their reaction free energy as a function of the applied potential bias we applied CHE model according to the following equation:

$$\Delta G = G_{adduct} - G_{Cu_6 \cdot 6H_2O} - n \cdot \left(\frac{1}{2} G_{H_2} + F \cdot U\right),$$

where F is the Faraday constant and U is the voltage bias. Since U is negative under the conditions of electroreduction, the reaction free energy raises with increasing U.

At around -0.2 V bias the structure $Cu_6[H_2O]_6$ *HCOO;1*OH becomes more stable than the structure with 5 *OH groups and remains the most stable on any higher potential bias as well. On this path the reduction of CO_2 can continue to methanol formation which path already becomes the most favored by a relatively small potential bias.

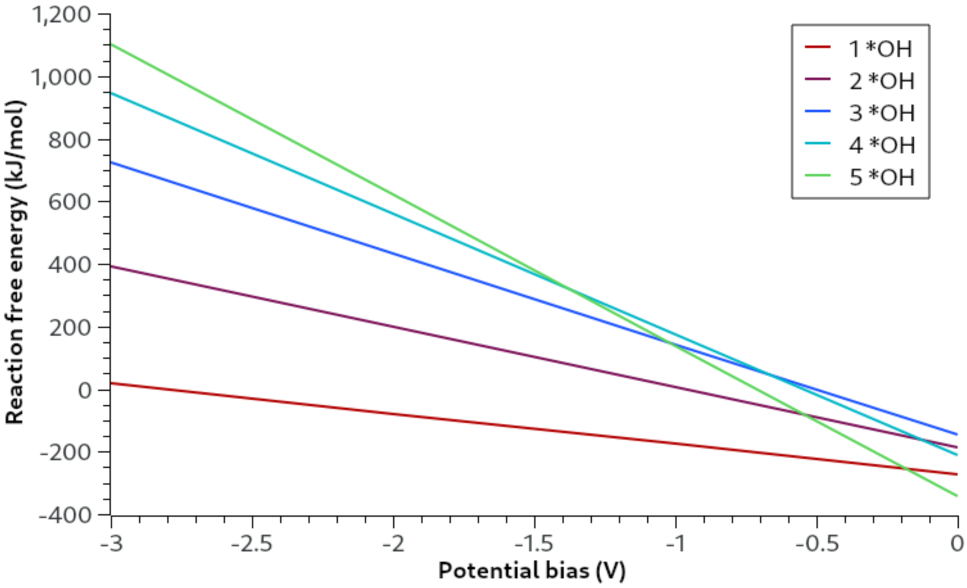

Figure S17: Dependence of the stability of different $Cu_6[H_2O]_6$ *HCOO; n *OH adducts on the applied potential bias

Investigation of $Cu_6[H_2O]_{35}$ without directly attached explicit water molecules

In this case, all 35 water molecules are part of the solvent shell and no water molecule is bound to the cluster. As for initial structure of the copper cluster we used the cluster geometry with the capped octahedral shape. The most important reaction paths we reached by the hydrogenation of the cluster can be seen on Figure S18.

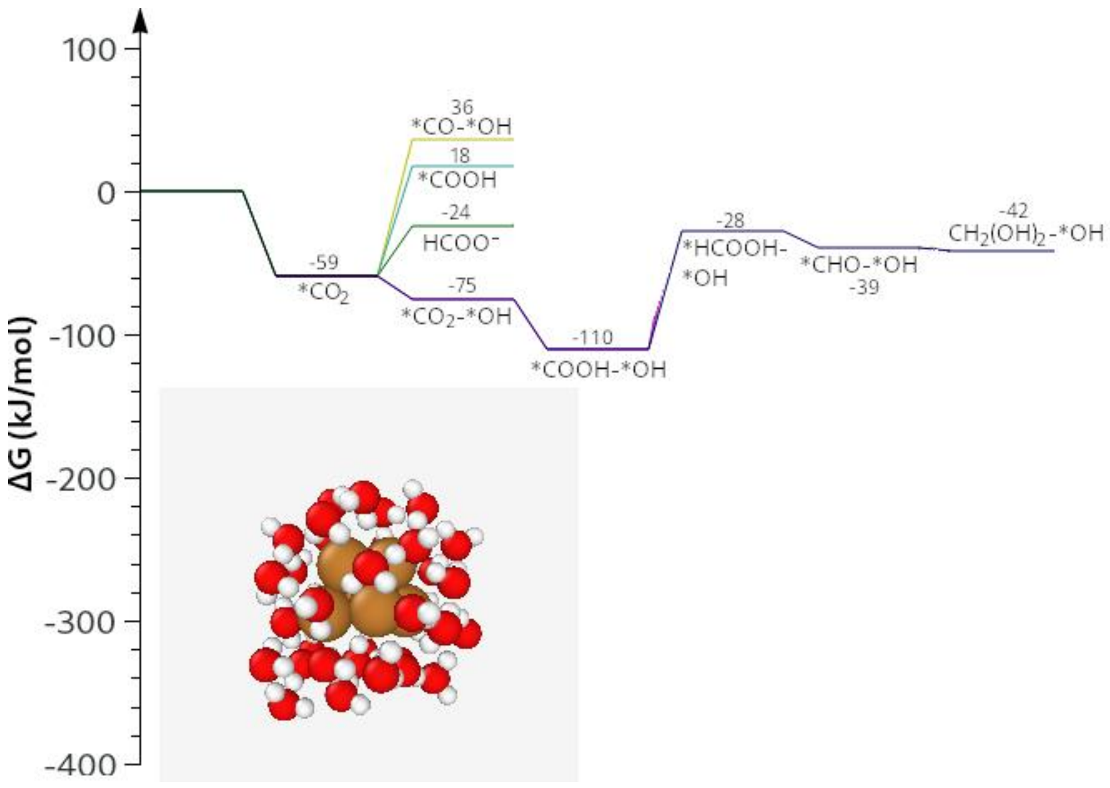

Figure S18: The most important CO_2 reduction mechanisms on $Cu_6[H_2O]_{35}$

Without external potential bias, the lowest reaction free energy paths were those which led to the oxidation of the cluster by water dissociation. The energetically most favored reaction path led to the formation methanediol, which was not reported as a product in implicit solvent computations before. It is important to observe that methanediol as a product appeared only if a hydrogen evolution step coupled with an adsorption of a *OH group was present on the path.

The green path shows the desorption of *HCOO from the cluster. The Bader atomic charges confirmed that the desorbed product is a formate anion ($HCOO^-$). *HCOO is considered to be the most stable intermediate after the first step of the reduction in implicit solvent, however, in explicit water this fragment shows low stability according to previous studies. [113]

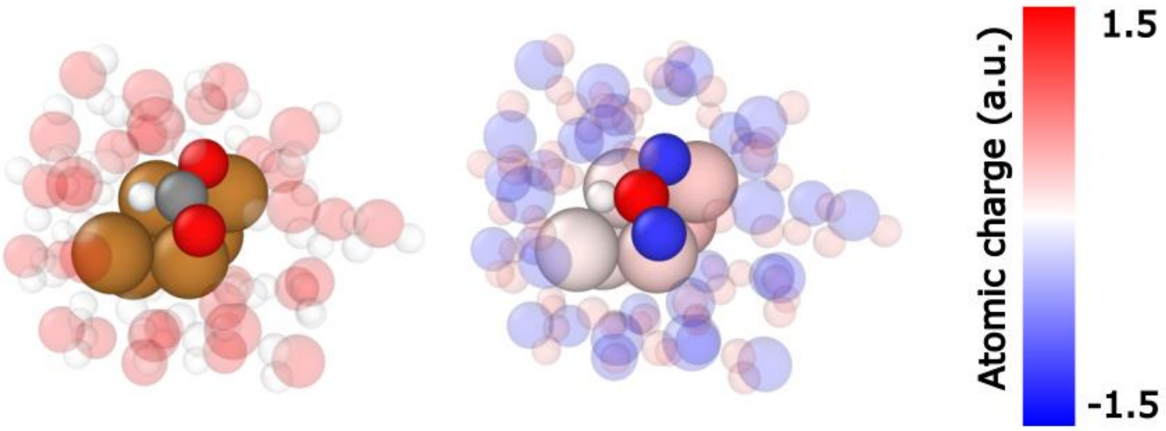

Figure S19: Bader atomic charges of the formate fragment bound to $Cu_6[H_2O]_{35}$ cluster without directly adsorbed explicit water molecules

EDA analysis of the systems with formate and carboxyl fragments pointed out that the interaction between the formate fragment and the cluster is weaker than the interaction between the fragment and the solvent shell. It explained why the fragment shows less affinity to adsorb on the cluster.

Table S11: Comparison of interaction energies by formate and carboxyl fragments in the 'nanodroplet' model

	Interaction free energies		
	between the cluster and the adsorbated explicit water molecules as well	between the cluster and the adsorbate only (percentage from total interaction free energy)	
	(kJ/mol)	(kJ/mol)	(%)
**$HCOO^-$**	-421	-153	36.3
***COOH**	-348	-245	70.4

We carried out a 20 ps molecular dynamics simulation on the HCOO-adduct. Analyzing the changes in the distance of the fragment and the cluster we can see that the initial 2.6 Å distance stabilized between 3 Å and 3.5 Å.

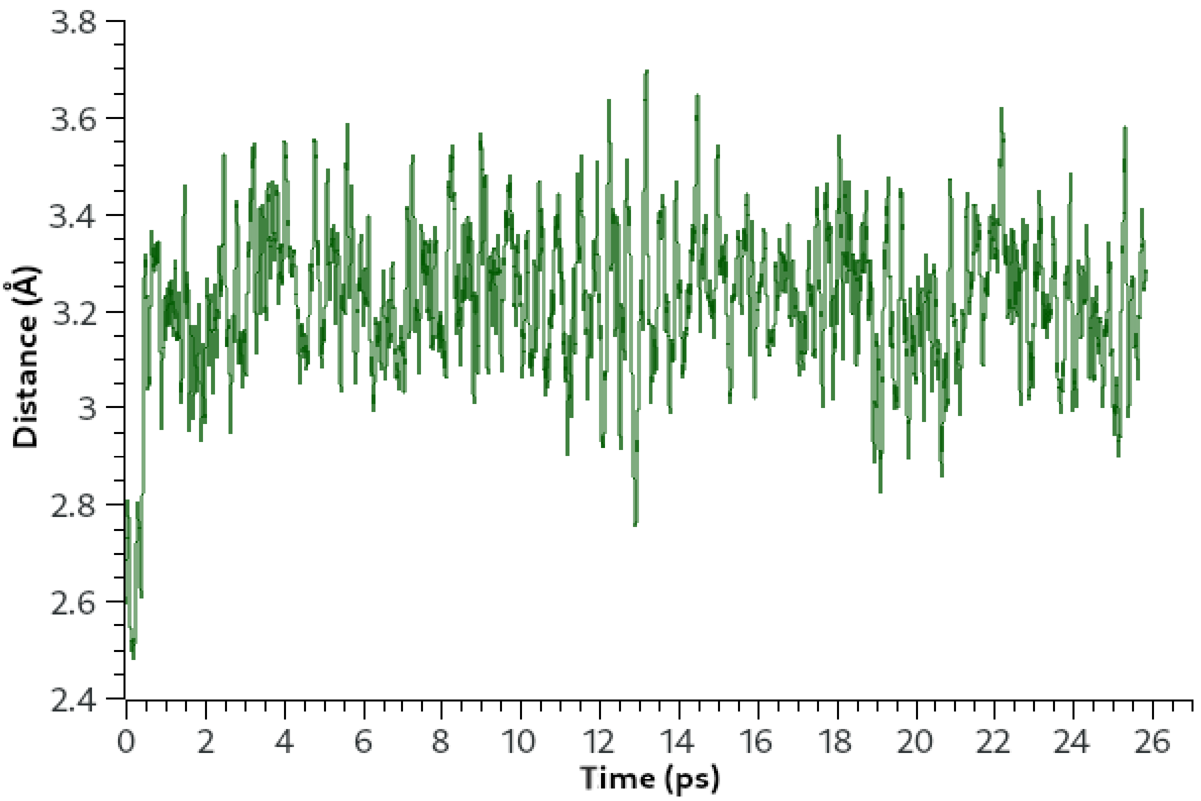

Figure S20: The distance between the oxygen of the formate fragment closest to the cluster and the nearest copper atom (determined at the last timestep) on $Cu_6[H_2O]_{35}$ cluster without directly attached explicit water molecules

The formate fragment migrated towards the edge of the investigated system, thus it was only partially solvated already shortly after the start of the MD simulation. This highlights that formate is a stable product of CO_2RR in this model.

For comparison, when directly attached explicit water molecules are present, the formate fragment did not desorb from the cluster, after an initial period its distance stabilized at 2 Å.

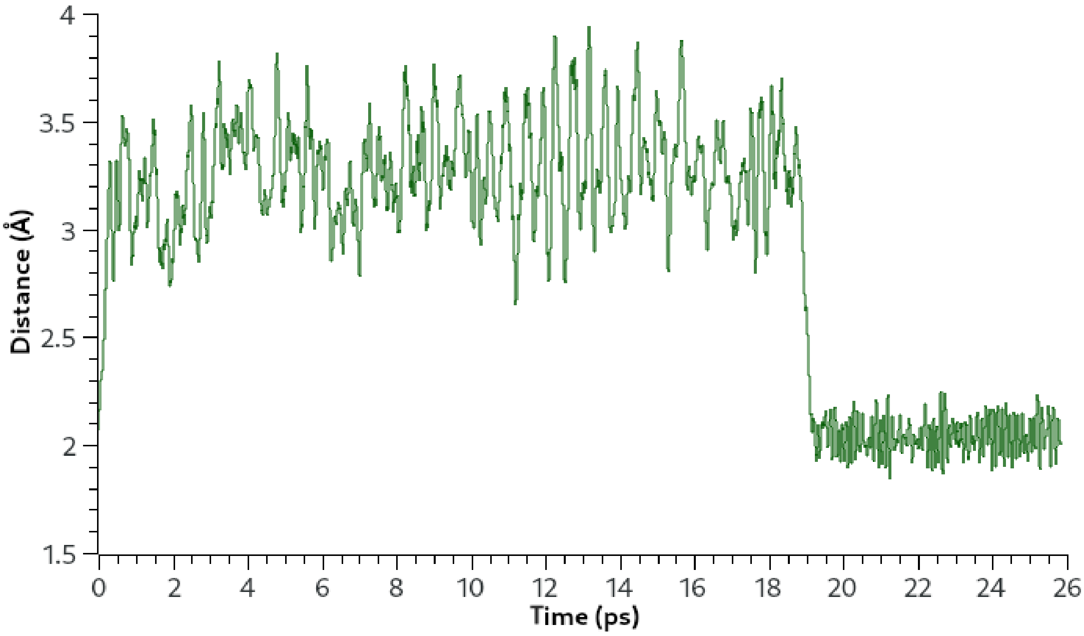

Figure S21: The distance between the oxygen of the formate fragment closest to the cluster and the nearest copper atom (determined at the last timestep) on $Cu_6[H_2O]_{35}$ cluster with directly attached explicit water molecules

For analyzing the stability of methanediol as a product we also carried out a molecular dynamics simulation on the final structure of the reduction. Methanediol remained stable during the total of 20 ps simulation time, the distance of the cluster and the product was stabilized between 3 Å and 4 Å.

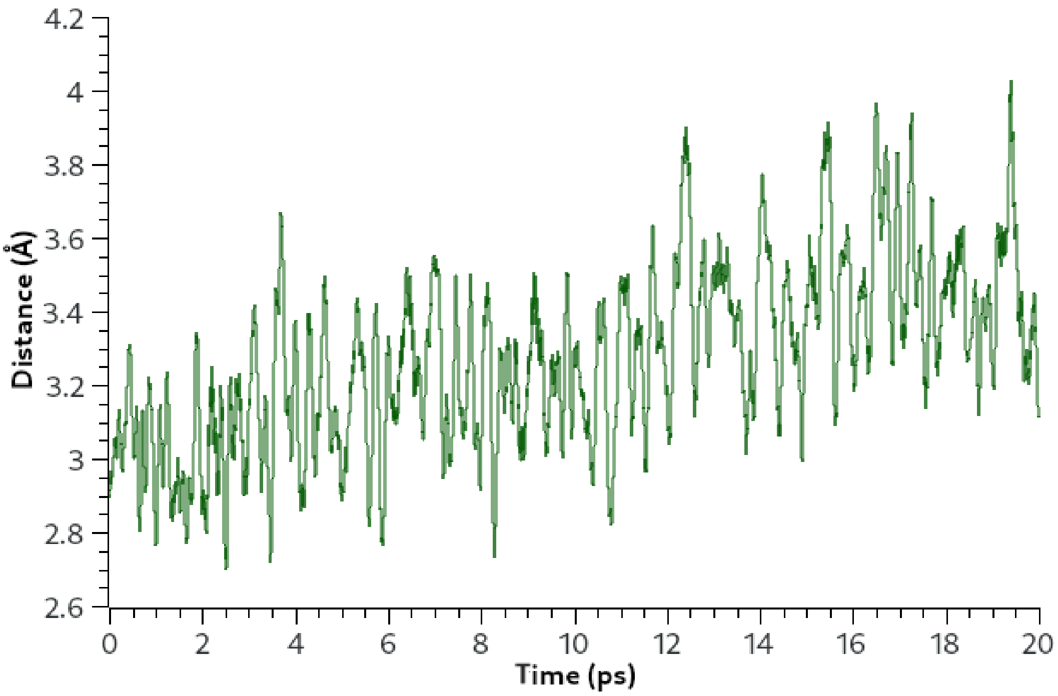

Figure S22: Distance of the carbon atom of methanediol and the nearest copper atom (determined at the last timestep) of $Cu_6[H_2O]_{35}$ cluster during MD simulation without explicit water molecules present

Additional analysis for the surface deposited cluster

Figure S23 shows that the graphene surface lost its two-dimensional, planar structure due to the adsorption of the cluster and became wavy.

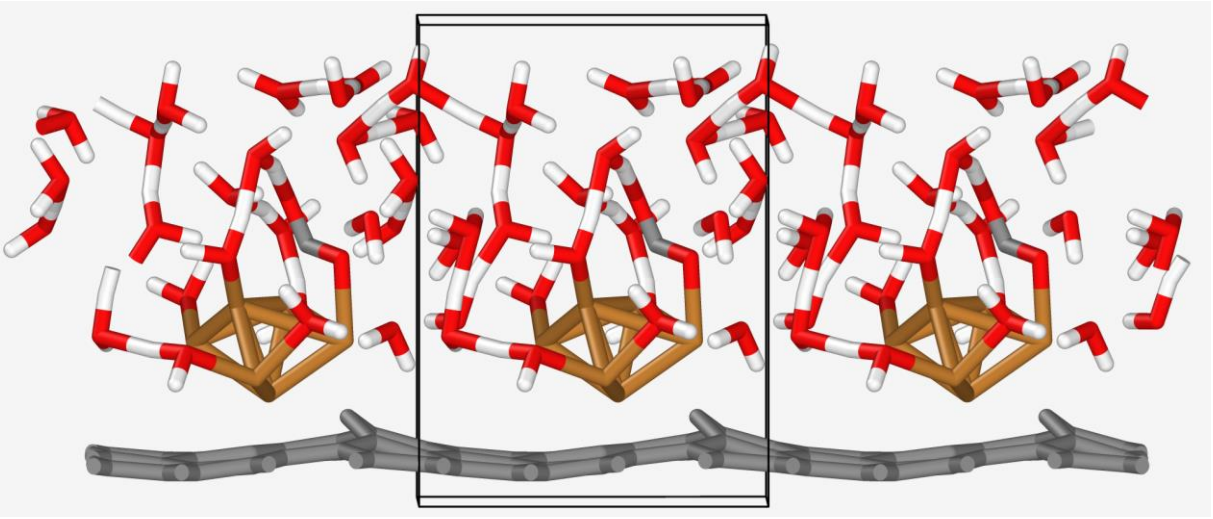

Figure S23: Roughening of the graphene surface after adsorbing the cluster

The changes in the distance of the cluster and the formate fragment are visible on the Figure.

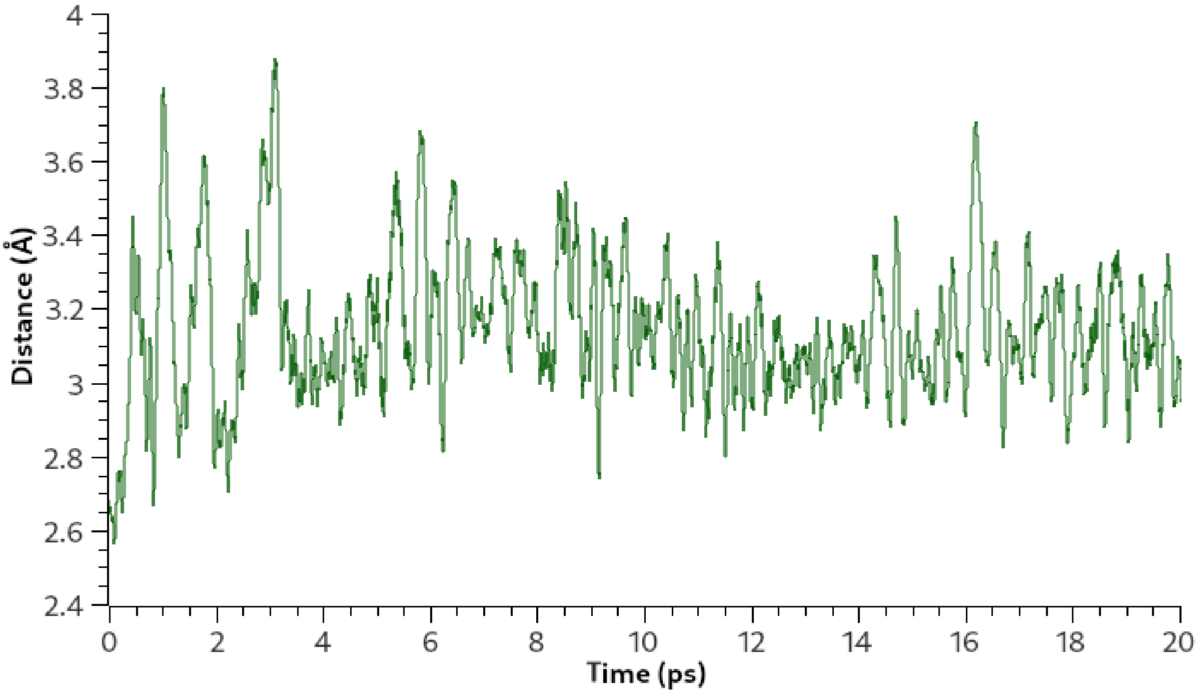

Figure S24: The distance of the oxygen atom of the formate fragment and closest copper atom (determined at the last timestep) of the surface deposited cluster during MD simulation

The molecular dynamics simulations show that without applying an external potential bias, we could not observe any significant structural change related to the dynamical behaviour of the system during the 20 ps simulation time.

Reaction path of the surface deposited cluster starting with the adsorption of CO_2 in a reduced form of *HCOO

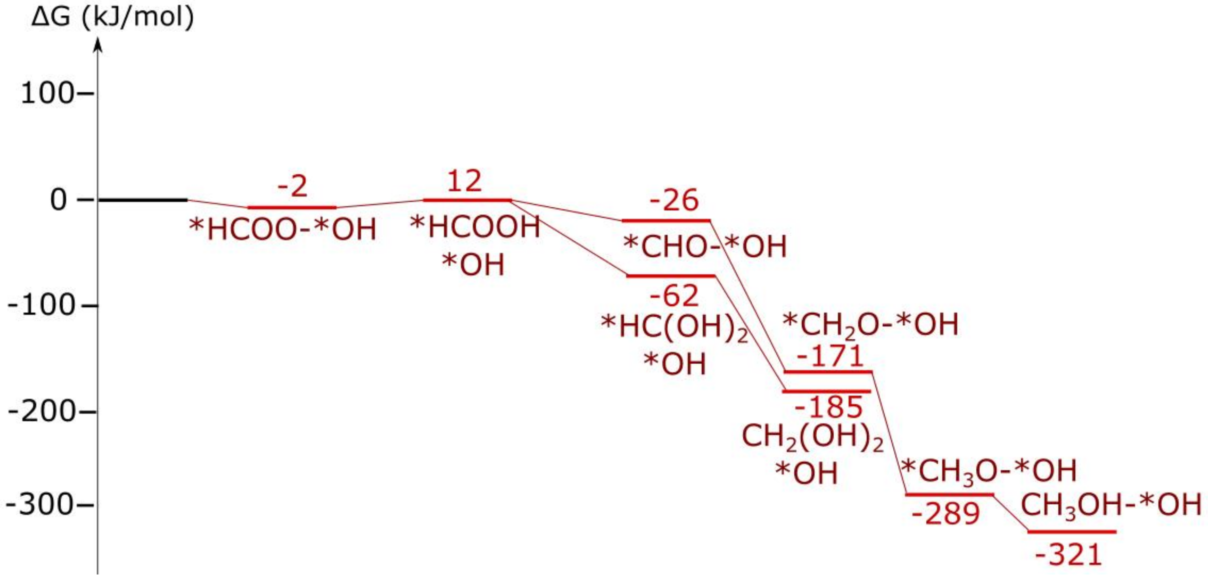

Figure S25: Reaction path of the surface deposited cluster starting with the adsorption of CO_2 in a reduced form of *HCOO (*OH groups are not indicated)

Linear correction of the reaction free energies obtained by using different DFT methods in the case of the surface deposited cluster

The final reaction free energies were calculated from the energy values obtained by using Q-Chem in all free-cluster models including the 'nanodroplet' where single point ωB97X-D/def2-TZVP energy computation was carried out in Q-Chem after the geometry optimization in GPAW. However, in case of the surface deposited cluster periodic boundary conditions were important thus we could not compute single point ωB97X-D/def2-TZVP energies using Q-Chem. However, we observed a linear correction based on the results of the 'nanodroplet' model. We listed the calculated reaction free energy values from Q-Chem (ωB97X-D functional def2-TZVP basis set) and GPAW (PBE-D3 functional and TZP basis set). The reaction free energies of the surface deposited cluster on the comparison figure (Figure 10 in the article) were corrected according to the following equation (represented as a trendline on Figure S26):

$$\Delta G_{corrected} = 0.989\ \Delta G_{PBE-D3/TZP,GPAW} - 15.917$$

Table S12: Reaction free energies originating from different methods in case of $Cu_6[H_2O]_{35}$ cluster (kJ/mol)

	ωB97X-D/def2-TZVP	PBE-D3/TZP
*CO_2	-66	-72
*CO_2;*OH	-68	-79
*HCOO;*OH	-188	-134
*HCOO	-105	-88
*HCOOH	-89	-71
*HCOOH;*OH	-102	-61
*CHO	-183	-169
*CHO;*OH	-168	-148
*CH_2O	-209	-201
*CH_2O;*OH	-185	-182
*CH_3O	-226	-221
*CH_3O;*OH	-251	-216
CH_3OH	-333	-302
CH_3OH;*OH	-271	-271
*CH_2OH;*OH	-164	-126
*$HC(OH)_2$;*OH	-123	-141
$CH_2(OH)_2$;*OH	-202	-211

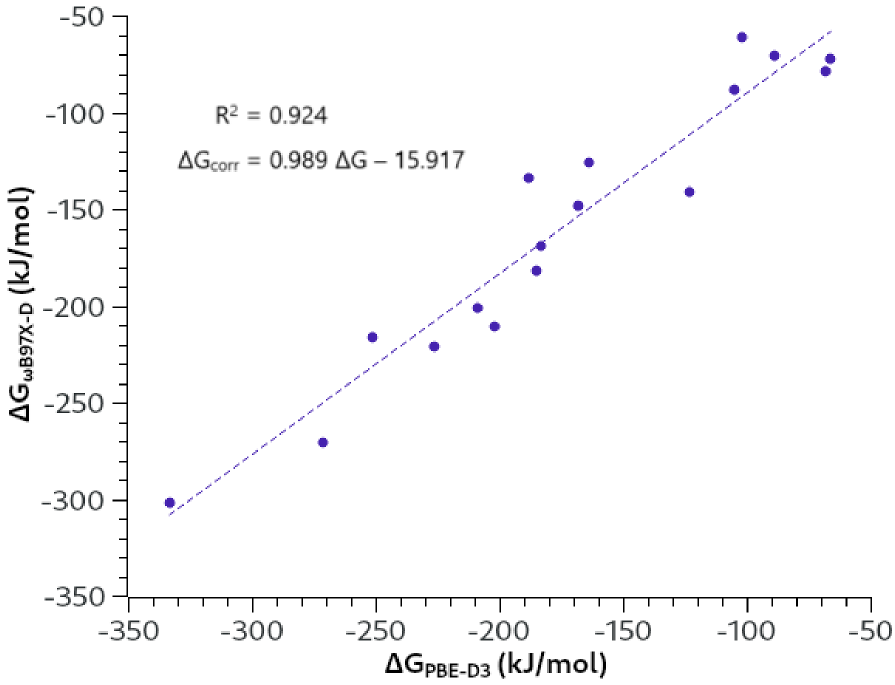

Figure S26: Linear correlation between the reaction free energies computed with different functionals

Tendencies in the effect of copper coordination

To investigate the relationship between the coordination number of the copper site and the water molecule binding strength, we simulated several structures using the PBE-D3/TZP method in GPAW software in gas phase using 8 Å vacuum in each of the non-periodic direction. Linear correction, as presented above, has also been applied. The results are visually reported on Figure 12 in the main text. Our results on different copper surfaces are in good agreement with previously reported results. [114] The conventional and generalized coordination numbers [88] are calculated according to the following equation: $\overline{CN}(i) = \frac{\sum_{j=1}^{n} cn\,(j)}{cn_{max}}$, where $\overline{CN}(i)$ is the generalized coordination number of a given atom, computed so that the coordination numbers of the neighbouring atoms are summarized and divided by the maximal coordination number present in the structure. (of an atom located in the middle of the investigated structure) The results that are shown are listed in Table S13.

Table S13. Binding energies on different coordination sites

	Cu(100)	Cu(111)	Cu(211)	Cu(100)*Cu	Cu(111)*Cu	Cu(211)*Cu
**Binding energy (kJ/mol)**	-20	-26	-24	-39	-47	-42
**Conventional coord. number**	8	9	7	4	3	3
**Generalized coord. number**	6	7.5	4.7	1.7	1	1
	Cu_{20}	Cu_{20} (a)	Cu_{20} (b)	Cu_{38}		Cu_{38} (a)
**Binding energy (kJ/mol)**	-60	-32	-37	-33		-34
**Conventional coord. number**	3	9	6	6		9
**Generalized coord. number**	1.3	9	4.7	3.5		7.5
	Cu_{6}	Cu_{6} (a)	Cu_{6} (b)	Cu_{55}		Cu_{55} (a)
**Binding energy (kJ/mol)**	-47	-34	-41	-54		-43
**Conventional coord. number**	2	4	3	6		8
**Generalized coord. number**	1.5	4	2.4	3.5		6

XYZ-coordinates of all structures presented in this article (in Å)

Reaction paths of $Cu_6[H_2O]_0$

*

6

Cu -0.7419262 -2.6974785 0.0001911

Cu -1.3832485 -0.3610423 -0.0000384

Cu 1.0084463 -1.0219888 -0.0002891

Cu 0.3789808 1.3819949 0.0006876

Cu 2.7058443 0.7077551 -0.0002067

Cu -1.9680967 1.9907596 -0.0003444

*CO_2

9

Cu 1.4652283 -2.6540374 0.0311124

Cu -0.3167684 -1.0460317 -0.2050019

Cu 2.0759180 -0.3370844 0.1287818

Cu 0.2292822 1.3598414 -0.1107710

Cu 2.5181829 2.0165074 0.2299663

Cu -2.0527893 0.6356411 -0.3434098

C -5.1753487 0.0362692 0.3535050

O -4.3264739 0.6873697 -0.1759735

O -5.9985842 -0.6233536 0.8871377

*HCOO

10

Cu 0.8159730 0.0125265 -1.2784067

Cu -0.9001281 -1.4410631 -0.0172133

Cu -0.9487261 1.4107662 -0.0356951

Cu 0.7831357 0.0155485 1.2948509

Cu 1.3093454 2.1189430 0.0158174

Cu 1.3783260 -2.0779942 0.0058827

C -3.4293880 -0.0550576 0.0288978

O -2.8700311 1.1018156 0.0135763

O -2.8300612 -1.1916147 0.0106467

H -4.5227848 -0.0743396 0.0609886

*HCOOH

11

Cu	-0.3966334	-0.9396421	2.0414889
Cu	-0.2896394	-0.1817294	-0.3688581
Cu	1.5383503	-1.5581127	0.6680142
Cu	1.1134305	0.9002621	1.1584100
Cu	2.0566555	0.3309845	-1.0143522
Cu	-1.2802422	1.3658959	1.3497308
O	-3.2624842	-1.2083417	-0.1608762
C	-2.7447402	-1.3264822	-1.3514637
O	-1.6115696	-1.0065740	-1.6647543
H	-2.6038617	-0.8086214	0.4544315
H	-3.4441975	-1.7532973	-2.0729674

*CH_2OOH

12

Cu	0.6195480	0.0425506	0.0484465
Cu	-1.3978872	-1.4595017	0.0491505
Cu	-1.4331131	0.8720324	-1.2897240
Cu	-3.5181327	0.0039096	-0.4818047
C	2.9222283	-1.8626132	0.1601480
O	2.0050625	-2.8087702	-0.1127663
O	2.5648254	-0.6117439	-0.3267818
H	1.1120755	-2.4612953	0.0934530

Cu	-1.5883029	0.7202987	1.0483420
Cu	-0.1494547	2.4626838	0.1820056
H	3.2016834	-2.2075001	0.9664694
H	3.2154523	-2.0748697	-0.7997106

*CHO

12

Cu	0.6084192	0.3673631	-0.1649351
Cu	-1.7841542	-1.0446913	0.0784345
Cu	-1.5079117	1.0216855	-1.3538405
Cu	-3.5469985	0.5410904	0.0390846
O	1.6069611	-2.3524333	0.1482500
C	1.8196608	-1.1389953	0.0172744
Cu	-1.3896455	1.2448208	1.0945080
Cu	0.0052257	2.7169870	-0.3455389
O	-0.9262588	-2.8410154	0.1324873
H	-1.1563608	-3.3499181	0.9146308
H	2.9166352	-0.9018899	0.0075443
H	0.0723852	-2.7002599	0.1596544

*CH_2O

13

Cu	0.5860545	0.3815378	-0.3250228
Cu	-1.7606318	-1.1081737	-0.1060487
Cu	-1.5403666	1.0373652	-1.3329853
Cu	-3.5880490	0.4806998	0.0218837
C	1.9438412	-2.1306651	0.2235849
O	2.0664754	-0.9434477	-0.0171978
Cu	-1.4740224	1.1525546	1.0879852
Cu	-0.0026239	2.7047992	-0.1053084
H	2.8474070	-2.7394915	0.3621853
O	-1.1015826	-2.8353214	0.2322087

H	-1.4103046	-3.1302301	1.0915663
H	0.9439567	-2.5924620	0.2997378
H	-0.5015826	-3.5353214	-0.2677913

*CH_3O

14

Cu	0.7859142	0.4987242	0.0510697
Cu	-1.5128252	-1.0502320	0.4428664
Cu	-1.3312941	0.8924103	-1.3351069
Cu	-3.3939858	0.3371954	-0.1662229
C	2.2729872	-1.9938625	0.1345362
O	2.2702578	-0.6308170	-0.1048625
Cu	-1.3204850	1.3045301	1.0437402
Cu	0.0451125	2.7506616	-0.5116077
H	3.2961527	-2.4032151	0.1144309
O	-0.9314465	-3.0051080	0.4410820
H	-1.1783782	-3.4805684	1.2411048
H	0.0282156	-3.0859196	0.3737945
H	1.7104118	-2.5658166	-0.6269575
H	1.8577667	-2.2693833	1.1211076

*CH_3OH

15

Cu	0.8025280	0.3928087	0.0163838
Cu	-1.2675306	-1.1617952	0.6832005
Cu	-2.8198509	2.4528085	-1.0610672
Cu	-3.5392861	0.2376145	0.0222446
C	2.8351030	-1.8335145	-0.1060345
O	2.6762913	-0.4338703	-0.3572455
Cu	-1.3934350	1.1558275	0.3264299
Cu	-0.2551328	2.6586781	-1.0856505
H	3.7823439	-2.1855875	-0.5165799

O -1.1981752 -3.2360917 0.8034597
H -1.9234686 -3.6096269 1.3142163
H -0.3927677 -3.5691215 1.2127324
H 2.0124722 -2.3391791 -0.6067965
H 2.7910876 -2.0465822 0.9632855
H 3.4062698 0.0460030 0.0436363

CH_4;*OH
16

Cu	0.8686956	0.2877713	-0.8484647
Cu	-0.6249591	-1.2527512	0.2780167
Cu	-2.8215390	2.1151676	-0.7855884
Cu	-1.5146824	0.1352769	-1.5987029
C	2.6758754	-2.7056223	0.0990630
O	2.5262631	0.8605324	-1.4865404
Cu	-0.9895347	1.1778704	0.5370850
Cu	-2.2185290	3.0604979	1.3905166
H	3.6587963	-2.2527871	-0.0293629
O	-1.1117027	-2.9888602	1.1871122
H	-1.8185017	-2.9062060	1.8358344
H	-0.3763495	-3.4077397	1.6468670
H	2.1551014	-2.7217273	-0.8581571
H	2.0948750	-2.1126923	0.8066188
H	3.1911944	0.1991794	-1.2841080
H	2.7835957	-3.7227730	0.4755747

*H[1]
11

Cu -1.2208424 1.3540424 -0.0055455
Cu 0.0028157 -0.5379166 1.2912615
Cu -2.1348402 -0.8683606 0.0033414
Cu 2.1452158 -0.8549890 -0.0035242

Cu 0.0020820 -0.5371779 -1.2914718
Cu 1.2058666 1.3573482 0.0057866
H -0.0086247 2.5245533 0.0044085
C 3.6600458 0.0507445 -0.3614263
O 4.9578306 0.0424020 -0.0892591
O 3.3776863 -0.5108030 -1.4575777
H 5.4517831 -0.4263573 -0.7493988

*H[2]

12

Cu 2.2965477 0.9195801 0.7143082
Cu 0.0077524 1.7513343 0.5273023
Cu 1.4123412 -0.7829079 -1.5563866
Cu -0.8083640 0.3356014 -1.2732659
Cu -0.6119199 -2.0403159 -1.6513320
Cu 0.2128181 -0.9376075 0.6210163
C 3.6600458 0.0507445 -0.3726193
O 4.9578306 0.0424020 -0.0532591
O 3.3776863 -0.5108030 -1.4575777
H 1.3324254 2.0407981 1.4302922
H 5.4517831 -0.4263573 -0.7493988
H -1.1977785 1.7979512 -0.5985256

*H[3]

13

Cu 2.6877974 0.4721633 0.5016905
Cu 0.4621877 1.7141058 0.5594080
Cu 1.9914298 -1.4473011 -1.4316349
Cu -1.1395003 1.3452499 -1.3223553
Cu -0.2147908 -0.6991016 -2.2007422
Cu 0.3042556 -0.8872987 0.1505120
C 4.1726357 -0.4559109 -0.3938459

O 5.4762479 -0.2361525 -0.1366659
O 3.9979333 -1.3222435 -1.2723463
H 1.7354972 1.3576285 1.4676219
H 5.5439329 0.4411751 0.5447917
H -0.9205680 2.3138727 -0.0531865
H -1.3930004 0.4261880 -2.6589653

*H^4
14

Cu 2.7642474 0.7180589 0.7608973
Cu 0.4285585 1.6349355 0.9357170
Cu 2.4533695 -0.7496634 -2.2707340
Cu -1.0904126 1.1116921 -0.9463798
Cu 0.0912054 -0.1602845 -2.7067756
Cu 0.7390760 -0.6196053 -0.2689809
C 4.2009227 -0.2349047 -0.1570201
O 5.4299470 -0.3778991 0.3658239
O 4.1117139 -0.7712140 -1.2795868
H 1.8407015 1.6555304 1.7073859
H 5.4534547 0.0551244 1.2262954
H -1.0922219 1.9479787 0.4454863
H -1.3621811 0.5223426 -2.4378772
H 1.3694010 -0.8496055 -3.4281013

*O
7

Cu 2.5187039 -0.0709661 0.7408949
Cu 0.5774293 1.2691513 -0.5263420
Cu 0.4895675 -1.2961484 -0.5233572
Cu -1.3974864 0.0403165 0.3091100
Cu -1.4604439 -2.4132187 0.2817330
Cu -1.3224220 2.4892665 0.2582168

O 1.8915880 -0.0661525 -0.9869362

*OH

8

Cu -0.6357983 0.3278736 1.2583961

Cu 1.4163624 0.6020903 -0.2152248

Cu 0.1607778 -1.5822838 -0.2205627

Cu -0.9071827 0.4786161 -1.2102745

Cu -2.1866551 -1.2785728 0.0769075

Cu -0.0317970 2.4738781 0.0757410

O 2.0998692 -1.2374969 -0.3938108

H 2.5743253 -1.5139263 0.3931523

Reaction paths of $Cu_6[H_2O]_1$

*

9

Cu 0.4884939 -0.4660451 0.9461114

Cu -1.1972186 -0.2777614 -0.7333015

Cu 0.5405590 1.3298544 -0.6319276

Cu -1.1343690 1.4985707 -2.3732542

Cu 0.6142543 3.0677705 -2.2549339

Cu -2.8451328 -0.1171256 -2.4379163

H 1.6341606 -2.3773168 2.2374464

O 1.3530662 -1.4851590 2.4875625

H 0.7729924 -1.6202987 3.2507722

*CO_2

12

Cu -1.2618159 -0.7606962 -1.0746248

Cu 1.1205684 -0.8554759 -1.9225098

Cu 0.3609916 1.3358961 -0.8976614
Cu 0.8121052 -0.8121060 0.4131464
Cu 2.6707286 0.5920651 -0.6449070
Cu -0.0535597 -2.8183239 -0.9144216
H 0.3845936 -1.6333996 2.8222482
O 0.7205396 -0.8245591 2.4232317
H 1.5398357 -0.6195759 2.8847667
C -1.5607438 1.8602668 -0.9994341
O -2.0249726 3.0152411 -0.9645921
O -2.3905206 0.8451677 -1.1181883

*HCOO(;*OH-not indicated in further cases)
12

Cu -1.0755661 -0.9550939 -0.5967779
Cu 0.8408193 -0.5764816 -2.1641749
Cu 0.5115350 1.1990174 -0.4332702
Cu 1.3662863 -1.1421040 0.0880267
Cu 2.7322771 0.6551386 -1.1208522
Cu 0.1502612 -2.8403217 -1.3980512
H -3.0324658 2.0370885 0.8468847
O 1.9713662 -1.7378753 1.7926369
H 2.5988480 -1.0894467 2.1179902
C -2.1980453 1.4557849 0.4319148
O -1.1173092 2.0689557 0.2640922
O -2.4302565 0.2498380 0.1786338

*H[1]
13

Cu -0.8622825 -0.6659127 -0.0909300
Cu 0.9378835 -0.3314259 -2.1781735
Cu 0.6001613 1.4736577 -0.4694964
Cu 1.6579461 -0.8306300 0.1685834

Cu 2.8233200 0.9204152 -1.1848847
Cu 0.3307551 -2.5107259 -1.2318401
H -3.0859096 2.4329080 0.0058997
O 3.1094212 -1.4030091 1.2600384
H 2.8296644 -2.1829851 1.7426319
C -2.1774209 1.8181030 -0.0645308
O -1.0855582 2.4282326 -0.0910213
O -2.3542275 0.5760255 -0.1106982
H 0.1618482 -1.8266662 0.4186125

*H^2
14

Cu -0.7604646 -0.6996467 -1.1109486
Cu 1.3290511 -0.0009850 -2.2053898
Cu 1.0180399 1.4168483 -0.2228259
Cu 2.7391089 -0.3469227 -0.1544206
Cu 3.0972798 1.5958986 -1.5268205
Cu 0.7428501 -1.9544613 0.4409494
H -2.5943477 2.3313364 0.6805171
O 2.5932764 -1.9504183 0.9152258
H 2.7248810 -1.7700766 1.8491468
C -1.7713407 1.7383386 0.2507806
O -0.6120247 2.1586552 0.5127434
O -2.0852297 0.7439989 -0.4259590
H -0.7712071 -1.9473900 0.0411046
H -0.1305487 -0.5570775 -2.6780463

*H^3
15

Cu -0.7408028 -0.4895581 -1.0205877
Cu 1.4276472 -0.1163028 -2.1694697
Cu 0.7818347 1.5343995 -0.1868060

Cu 3.0783959 -0.7237061 -0.4566057

Cu 2.9773215 1.6029968 -1.2884689

Cu 0.8125171 -1.5518480 0.6577220

H -2.9045608 2.3996060 0.5415699

O 2.6521125 -1.6028438 1.1785965

H 3.0016168 -2.4970441 1.2030101

C -2.0478923 1.8161243 0.1663650

O -0.9120681 2.2340694 0.5170366

O -2.3022086 0.8281147 -0.5460475

H -0.7146156 -1.4888565 0.3252798

H -0.1337921 -0.2505428 -2.5584974

H 3.1653036 -0.1082654 -1.9616540

*H[4]

16

Cu -0.5864243 -0.2587270 -1.2140621

Cu 1.5266881 -0.3746641 -2.5008836

Cu 0.5552648 1.2145660 0.5226107

Cu 3.3513731 -1.2885703 -1.0736797

Cu 2.6499112 1.0826475 -0.7843376

Cu 1.1682049 -1.3339029 0.3924055

H -3.1738440 2.3276819 0.1681239

O 2.6970796 -2.5034680 0.2573932

H 3.2347869 -2.5090554 1.0525560

C -2.2163680 1.8128543 -0.0168307

O -1.2379405 2.1927987 0.6661739

O -2.2300270 0.9150608 -0.8901411

H -0.1446908 -0.4367647 0.5094726

H -0.0591989 -0.5566151 -2.7582734

H 3.1926528 0.0123245 -2.0774863

H 1.9586392 2.0696668 0.2956677

*H[5]

17

Cu -1.1117254 -0.0582832 -1.1181490
Cu 1.1267638 -0.5849458 -2.0608305
Cu -0.0145731 1.5883408 0.4922148
Cu 2.9482842 -1.3618293 -0.5180243
Cu 2.1873998 1.0349236 -0.5147982
Cu 0.6977964 -0.8779805 0.5024036
H -3.4467458 3.0424856 -0.6889287
O 4.5622264 -2.5810265 -0.3427392
H 5.4033003 -2.2020720 -0.6183396
C -2.5675546 2.3819762 -0.6110601
O -1.6427957 2.7752454 0.1366068
O -2.6076431 1.3258357 -1.2837231
H -0.7693191 -0.0113837 0.6251655
H -0.4387508 -0.7630871 -2.4697173
H 2.7068072 -0.0216246 -1.7519948
H 1.5321989 2.2040548 0.4095351
H 4.7021047 -2.9318955 0.5426541

*H^6
18

Cu -1.0781342 -0.0185854 -1.0971236
Cu 1.1136636 -0.5815108 -2.0951833
Cu 0.0487218 1.8185142 0.2733913
Cu 2.9182478 -1.2379667 -0.3960262
Cu 2.2170321 1.2068266 -0.7536060
Cu 0.7479293 -0.6502163 0.6457762
H -3.5174323 3.0098244 -0.7728778
O 4.6368559 -2.2925548 -0.8211318
H 5.2942677 -1.8161182 -1.3364958
C -2.6003748 2.4010436 -0.7119629
O -1.6401012 2.9055083 -0.0855031

O -2.6369131 1.2848520 -1.2791682
H -0.6586663 0.2210885 0.6164748
H -0.4557697 -0.8380665 -2.4045989
H 2.7196202 -0.1039765 -1.7374670
H 1.5696666 2.4557353 0.0541469
H 5.1029160 -2.6550843 -0.0620771
H 2.0271567 -1.6293224 0.9400115

*HCOOH
13

Cu -0.8966573 -1.0664487 -0.647706277
Cu 1.0442315 -1.0829129 -2.228423745
Cu 0.9018449 0.9374040 -0.767246771
Cu 1.5346756 -1.4107635 0.090282961
Cu 3.0501962 0.0994099 -1.346647089
Cu 0.1432833 -3.1472753 -1.202746937
H -2.6992276 2.2211370 0.186054337
O 2.1457162 -1.9557238 1.780780001
H 2.8589052 -1.3732534 2.047585494
C -1.8948743 1.4840765 0.312884974
O -0.6647574 2.0330962 -0.202520560
O -2.1295170 0.2652598 -0.046494281
H -0.5082184 2.8939803 0.198387893

*CH_2O_2
13

Cu -0.6777676 -0.8580418 -0.9151423
Cu 1.4351089 -0.9604974 -2.2656942
Cu 1.0431134 0.9997345 -0.7423983
Cu 1.5814503 -1.3317178 0.1783314
Cu 3.2949987 0.0877253 -1.0068714
Cu 0.4149080 -2.9985932 -1.3101039

H -1.6377648 1.5549190 1.0989824
O 2.1377746 -1.8638374 1.8646333
H 1.7071543 -1.3317627 2.5371725
C -1.6235895 1.6409827 -0.0097360
O -0.3945603 2.1487105 -0.4097253
O -1.9955945 0.4233839 -0.5646008
H -2.3996315 2.3869815 -0.2806565

*CH_2OOH
14

Cu -0.6889614 -0.8338515 -1.0124081
Cu 1.4880768 -0.8108100 -2.2461018
Cu 1.2177086 1.1655578 -0.7295646
Cu 1.6263191 -1.2392161 0.0961348
Cu 3.3953929 0.2291352 -1.0568486
Cu 0.4047267 -2.9017977 -1.4522025
H -1.2063518 1.2588103 1.2085019
O 2.0251539 -1.8120101 1.8395613
H 1.4413537 -2.5374975 2.0675665
C -1.3621578 1.7560518 0.2411113
O -0.2604290 2.2823021 -0.3040616
O -1.9533400 0.6599353 -0.5650764
H -2.1687993 2.4967812 0.3367190
H -2.3055531 1.0489567 -1.3730014

*CHO
14

Cu   -0.6608324   -0.9396536   -0.7548611
Cu   1.2131207   -1.2341602   -2.3820491
Cu   1.2015486   0.9409775   -1.0817343
Cu   1.7652286   -1.3856105   -0.0705802
Cu   3.2836082   -0.0810807   -1.6894658

Cu	0.2811163	-3.1612741	-1.1542807
O	2.3997912	-1.5438007	1.6888161
H	1.9703186	-2.2910124	2.1087242
H	-1.8627168	1.0411808	0.6435195
O	0.5205644	2.7758008	-0.4724507
C	-1.8746873	0.5409233	-0.3693287
H	1.2281588	3.4042558	-0.2969868
O	-2.7236671	0.9853687	-1.1346223
H	-0.0194125	2.7613865	0.3247887

*CH_2O

15

Cu -0.6706504 -0.7442954 -0.6074516

Cu 1.3288370 -0.8828015 -2.1146092

Cu 1.2241422 1.0390018 -0.4829621

Cu 1.6127650 -1.3195715 0.2315854

Cu 3.3688547 0.0099216 -1.1704166

Cu 0.2423569 -2.9080387 -1.3558879

H -1.7434713 1.8364289 0.5430900

O 2.4221245 -2.2838019 1.6440219

H 1.8982688 -3.0683503 1.8141407

C -2.6115126 1.3078325 0.1275049

O 0.5506346 2.9982383 -0.4526563

O -2.4953908 0.2247128 -0.4038689

H -3.5976343 1.7850324 0.1977578

H 0.8484612 3.5010957 0.3122614

H 0.8303102 3.5028983 -1.2231752

*CH_3O

16

Cu -0.6391917 -0.4181211 -1.0160645

Cu 1.3828735 -0.9249531 -2.4012452

Cu 1.7560191 1.2276966 -1.1779440
Cu 1.6573330 -1.1026350 -0.0450168
Cu 3.5997732 -0.2398558 -1.4985942
Cu 0.0521326 -2.6892155 -1.2776564
H -3.0735276 0.6431692 0.5682388
O 2.1534506 -1.5667963 1.7079106
H 1.4996848 -2.1715769 2.0627627
C -2.3110388 1.4235325 0.4573580
O 0.4610224 2.6921206 -0.8819028
O -1.5445604 1.2440100 -0.7021576
H -2.8335853 2.3893799 0.4286548
H -0.4415892 2.1503333 -0.7625894
H 0.6170017 3.1898941 -0.0754470
H -1.6984752 1.4111303 1.3706934

*CH_3OH
17

Cu -0.6668938 -0.6176687 -0.8242639
Cu 1.3214828 -0.9968240 -2.2811008
Cu 1.3296286 1.0807298 -0.8877601
Cu 1.5975140 -1.2029434 0.0767132
Cu 3.4262345 -0.1314084 -1.4421252
Cu 0.1570631 -2.9007948 -1.3342792
H -2.4811723 1.0422835 1.3747790
O 2.2129810 -1.8556579 1.7389357
H 1.6430617 -2.5777846 2.0086039
C -2.6734351 1.3234561 0.3382122
O 0.9261405 3.1114885 -0.8424339
O -2.5441333 0.1941519 -0.5315280
H -3.6710637 1.7548109 0.2483762
H 0.2843261 3.3563724 -0.1681582
H 1.7043269 3.6521789 -0.6725922
H -3.2126090 -0.4586863 -0.3058515

H -1.9320565 2.0527671 0.0206989

CH_4;OH

18

Cu	-0.6668938	-0.6176687	-0.8242639
Cu	1.3214828	-0.9968240	-2.2811008
Cu	1.3296286	1.0807298	-0.8877601
Cu	1.5975140	-1.2029434	0.0767132
Cu	3.4262345	-0.1314084	-1.4421252
Cu	0.1570631	-2.9007948	-1.3342792
H	-5.4811723	1.0422835	1.3747790
O	2.2129810	-1.8556579	1.7389357
H	1.6430617	-2.5777846	2.0086039
C	-5.6734351	1.3234561	0.3382122
O	0.9261405	3.1114885	-0.8424339
O	-2.5441333	0.1941519	-0.5315280
H	-6.6710637	1.7548109	0.2483762
H	0.2843261	3.3563724	-0.1681582
H	1.7043268	3.6521789	-0.6725922
H	-3.2126090	-0.4586863	-0.3058515
H	-4.9320565	2.0527671	0.0206989
H	-6.0475457	0.2645009	0.4287797

Reaction paths of $Cu_6[H_2O]_2$

*

12

Cu 1.3887118 -2.0258106 -0.4403780

Cu 0.1237686 0.0355979 0.0154669

Cu 1.0552134 -0.3670037 -2.1948418

Cu -0.3316938 1.6699116 -1.7286494

Cu 0.6062668 1.2679312 -3.9449380

Cu -1.2627432 2.0149566 0.4958951
H 1.3371662 -4.4833045 0.4270791
O 2.0563591 -3.8757125 0.2250514
H 2.5965184 -3.8356898 1.0211043
H -1.8917606 3.5182645 2.5516946
O -2.4075979 2.9959050 1.9287588
H -2.9524579 2.4094542 2.4633679

*CO_2
15
None
Cu 0.7814898 -2.4159151 -0.2262244
Cu -1.3342498 0.1876741 -0.1030747
Cu 0.5338777 -0.7215931 -1.9229824
Cu 0.3434197 1.5965967 -1.0935141
Cu 1.8172527 1.0187969 -2.9546923
Cu -1.0266166 2.4018870 0.7720573
H 2.8417332 -3.5143249 0.7348067
O 2.2943596 -2.7602829 0.9792218
H 2.8929973 -2.0112772 1.0693620
H -1.0360867 3.9996589 2.8334477
O -1.4368273 4.0279781 1.9584043
H -2.3762125 4.1819934 2.1029384
C -0.8881066 -2.2744795 -1.2799781
O -1.1202992 -3.1926596 -2.0804800
O -1.8473344 -1.5886557 -0.7320982

*HCOO(;*OH-not indicated in further cases)
15

Cu -0.1164053 -0.8261218 0.1715817
Cu 1.8950819 0.5095308 -0.0726630
Cu 1.1688680 -1.1782732 -2.2844924
Cu 0.0457255 0.8490904 -1.6697218

Cu -0.1564640 -0.0188834 -3.9188780
Cu -0.1288820 1.5852298 0.7240575
H 3.1006859 -2.8555243 -2.4919553
O 2.3798210 -2.7287062 -1.8666609
H 2.7867682 -2.6777662 -0.9955849
H -2.3609983 1.6398479 2.5812323
O -1.1002256 -2.3558270 0.5420472
H -0.9689149 -2.6087133 1.4582929
C -2.1027795 2.7082098 2.4374679
O -1.1219113 2.9429966 1.6533275
O -2.7593305 3.5612041 3.0267299

*H[1]

16

Cu -1.1249998 -0.7978911 -0.7404050
Cu 1.8626387 2.0905801 -2.0864034
Cu 1.8332548 -0.2494934 -1.4425643
Cu -0.2174033 0.8030781 -2.3409966
Cu 1.6856211 0.3542091 -3.8696160
Cu 0.6013506 1.4699503 -0.0182967
H 2.6280051 -1.8187857 0.3644309
O 2.0151945 -1.9039551 -0.3707405
H 1.0652216 -2.0384980 0.0232057
H -1.7226569 0.8735709 1.5794866
O -0.3664788 -2.0421889 0.4511784
H -0.7783677 -2.9052165 0.3755526
C -1.1206551 1.5312433 2.2399482
O -0.0185557 1.9493795 1.7494714
O -1.5483502 1.7984373 3.3591077
H -1.6874808 0.2113651 -1.8313119

*H[2]

17

None

Cu -1.3265517 -1.3401281 -1.2618389

Cu 0.9353994 2.0971382 -0.4218676

Cu 2.0488220 0.1816505 -1.5207880

Cu -0.3148647 0.7286190 -2.0630369

Cu 1.5442854 2.2000793 -2.7630520

Cu 1.1155817 -2.2292907 -0.6388834

H 3.3606182 -2.4064223 1.4488748

O 2.8109025 -1.5097661 -0.9901859

H 3.2395050 -1.9593625 -1.7224238

H -0.9169437 3.5148045 2.5474005

O -0.633350920 -2.787210107 -0.228107336

H -0.8211457 -3.6419001 -0.6242059

C -0.6934838 3.2338167 1.5004387

O 0.4654840 2.7290580 1.3383079

O -1.5467673 3.4277052 0.6362733

H -1.7753440 -0.0688629 -2.0807740

H 3.5285417 -2.5634676 2.1593625

*H^3 (hydrogen removed after previous step)

16

Cu -1.2417561 -1.2804171 -1.0270148

Cu 1.4692948 1.8675973 0.1022177

Cu 2.7193774 -0.0964167 -0.7429078

Cu -1.4417816 1.0405519 -0.1800138

Cu 0.4448453 0.5295698 -1.8163699

Cu 1.2748819 -2.1474864 -1.9352866

O 3.0673648 -1.5937057 -1.8840739

H 3.5979230 -2.2558992 -1.4329275

H -1.0040889 4.7525990 0.7877268

O -0.5035906 -2.7348731 -2.0242366

H -0.5970330 -3.5641805 -1.5477958

C -0.6896716 3.7259900 0.5498321

O 0.5426662 3.5142622 0.5379808

O -1.6054869 2.9051733 0.3234046
H -2.2034175 -0.3736066 -0.1702382
H 2.8472988 1.0666846 0.3142268

*HCOOH
16

Cu 0.0684512 -1.3196005 0.2477952
Cu 1.8847840 0.1791815 -0.3967523
Cu 1.4241499 -1.4833547 -2.2543702
Cu -0.2445309 0.1931103 -1.6545851
Cu -0.1850758 -0.6146480 -3.9077030
Cu -0.2369176 1.0992551 0.6291078
H 2.8741645 -3.4597884 -3.2019589
O 2.5399322 -3.2347947 -2.3276732
H 3.3106694 -3.1964498 -1.7524271
H -2.5058756 3.1308554 1.1111869
O -0.4574493 -3.0232422 0.8064223
H 0.2099201 -3.6672615 0.5622210
C -1.6997244 3.3140698 1.8245199
O -0.6653228 2.6746202 1.8557986
O -1.9713237 4.2976020 2.6390414
H -1.2395122 4.4362303 3.2614228

*CH_2OOH
17

Cu 0.0866157 -0.8160433 0.1654997
Cu 1.9961622 0.4149100 -0.5574765
Cu 1.1680913 -1.4145172 -2.1281327
Cu -0.1360308 0.5725202 -1.9879390
Cu -0.3579211 -0.8064649 -3.9582617
Cu -0.1387778 1.8455514 0.0209602
H 2.6156167 -3.3961288 -3.0118162

O	2.3366953	-3.1055611	-2.1380237
H	3.1397762	-3.0333170	-1.6131805
H	-0.5212408	3.5593755	3.5317222
O	-1.2256525	-1.7947813	1.0654453
H	-0.8143399	-2.2745541	1.7873716
C	-0.7655430	3.0390898	2.6010759
O	0.0777748	2.8116231	1.7582057
O	-2.0112463	2.6737912	2.4416119
H	-2.5517975	2.9325302	3.2003062
H	-0.2455430	2.4290898	3.1010759

*CHO

17

Cu	-0.2878513	-1.3691429	0.1363327
Cu	1.8606894	-0.0610621	0.1048698
Cu	0.7041252	-0.8562777	-2.0768661
Cu	-0.3406220	0.8885474	-0.7281520
Cu	-1.7129595	-0.8224873	-1.9080715
Cu	0.2123604	0.5809747	1.7864649
H	2.4511480	-1.1843942	-4.0072201
O	2.2003560	-1.6268708	-3.1857810
H	3.0183494	-1.7714148	-2.7018358
O	-0.9638914	-2.7738639	1.1833678
H	-0.4514052	-2.8364903	1.9922023
C	-0.4658246	1.6173559	3.2988401
O	0.0349305	2.6636293	3.7029506
O	-1.1181330	2.7746850	-0.5310309
H	-0.8924743	3.3862033	-1.2419062
H	-0.8052654	3.1846680	0.2816453
H	-1.3910734	1.2995257	3.8708591

*CH_2O

18

Cu 0.3337405 -1.4467691 0.0052316
Cu 2.0959272 0.0451096 -0.7656614
Cu 1.3205318 -1.9405212 -2.3437362
Cu -0.0643970 0.0216682 -2.0355572
Cu -0.3775895 -1.4585724 -3.9648833
Cu 0.1733705 1.0014564 0.3417063
H 3.4533937 -3.3347667 -2.7993559
O 2.8369344 -3.2706966 -2.0630917
H 3.3699823 -3.0846925 -1.2837152
H -1.8199919 3.3311611 0.0741344
O -0.2948312 -3.0398828 0.7360157
H -0.7640568 -3.5393193 0.0652380
C -1.5962889 3.1349676 1.1246819
O -0.6248707 2.2971765 1.4581638
H -1.8736617 3.8995472 1.8536452
O -1.2688922 1.7001125 -2.4269009
H -0.8488437 2.5026106 -2.0992480
H -2.1112417 1.6484932 -1.9622357

*CH_3O
19

Cu 0.3710540 -1.4551127 0.1023663
Cu 2.1939626 -0.0173126 -0.6553420
Cu 1.3067263 -1.8897121 -2.2766209
Cu -0.0161478 0.0966207 -1.8453269
Cu -0.5567338 -1.4069056 -3.7070524
Cu 0.3531593 0.9830580 0.5480468
H 3.1971326 -3.5038864 -2.9945879
O 2.8470225 -3.2119886 -2.1469321
H 3.5997848 -2.8827960 -1.6459156
H -0.9384414 3.8241781 0.3682135
O -0.4063590 -2.8853547 1.0060449

H -0.8671712 -3.4543028 0.3867284
C -1.3596623 3.1767170 1.1572731
O -0.4485773 2.2526277 1.6548471
H -2.2652638 2.7100677 0.7305627
O -1.1625853 1.7911781 -2.2202387
H -1.3819051 2.3107373 -1.4392461
H -1.9993575 1.5910999 -2.6509437
H -1.7111244 3.8546605 1.9519840

*CH_3OH
20

Cu 0.5289253 -1.4989339 0.5127692
Cu 1.7129594 0.0446100 -0.9720068
Cu 1.0550745 -1.9556412 -2.3743498
Cu -0.6541147 -0.5158144 -1.3972590
Cu -1.1493895 -1.6501414 -3.4425360
Cu -0.1333355 0.8698948 0.5597289
H 2.3438994 -3.9375162 -3.5186269
O 2.3687379 -3.5441552 -2.6404654
H 3.2874708 -3.3030781 -2.4843791
H -1.5993301 3.8123193 0.2870362
O 0.6261374 -3.0547444 1.5398409
H 1.1627170 -3.7122206 1.0931700
C -1.7407349 3.3198718 1.2500141
O -0.5793103 2.5614854 1.6105024
H -2.5672859 2.6168968 1.1786500
O -0.5605529 2.7314807 -2.3424850
H 0.1058095 2.2242699 -1.8639633
H -1.3828923 2.2835674 -2.1212703
H -1.9546970 4.0597209 2.0214357
H 0.1669118 3.1567706 1.7252202

CH_4;*OH

21

Cu	1.1693854	-0.5894963	-0.8580193
Cu	0.4673948	0.7756643	-2.7553493
Cu	0.2095455	-3.5529774	-1.6356995
Cu	-0.6969572	-1.3643963	-2.2656308
Cu	-1.7226410	-3.4263269	-3.0026147
Cu	-0.9759002	0.6164236	-0.8058372
H	2.4502207	-4.4280992	-0.8261374
O	1.7404680	-3.9085115	-0.4409398
H	2.1251654	-2.9733784	-0.1928493
H	-1.1785384	3.4847664	3.7300733
O	2.5252168	-1.5725645	0.0380264
H	2.5971641	-1.3449377	0.9667618
C	-1.7174934	4.0091713	2.9415775
O	-2.3119693	1.4140074	0.2151433
H	-1.0788015	4.7850446	2.5205870
O	1.0527346	1.8624077	-4.3361564
H	1.2115141	1.3501377	-5.1357274
H	0.4424343	2.5644097	-4.5848705
H	-2.2669352	1.1197317	1.1272002
H	-2.6151846	4.4643405	3.3586522
H	-1.9951492	3.3022476	2.1597866

Reaction paths of $Cu_6[H_2O]_6$

*

24

Cu	2.3369506	0.2153753	-0.0662694
Cu	0.0475229	0.9033295	0.6316385
Cu	0.2242887	-0.1725502	-1.3910895
Cu	-1.7762983	-0.7644972	-0.0713908
Cu	0.5369021	-1.6033554	0.6055600

Cu	-1.5085669	1.6361498	-1.1796211
O	4.5073262	0.0455813	-0.2347618
O	-0.3396687	1.5488056	2.6014647
O	-2.9837384	2.8900361	-2.1686580
O	-3.8582891	-1.1802721	0.5315232
O	1.3055197	-3.0331417	2.1113062
O	0.5788646	-0.8466460	-3.3459155
H	4.9577583	0.8632556	0.0002133
H	4.8517687	-0.6191828	0.3703440
H	0.4669062	1.8549082	3.0267638
H	-0.9340665	2.3049427	2.6031199
H	-2.9781816	3.7914332	-1.8299733
H	-3.8734165	2.5604700	-2.0046160
H	-4.3062923	-0.3746632	0.8081583
H	-3.8929997	-1.7669672	1.2935975
H	0.5919661	-3.3696734	2.6623582
H	1.9133869	-2.5981316	2.7173079
H	1.1485224	-1.6214417	-3.3379994
H	1.0514452	-0.1867481	-3.8615447

*HCOO;*OH (oxidated)

27

Properties=species:S:1:pos:R:3:initial_charges:R:1 pbc="F F F"

Cu	1.91831560	-0.17313220	0.26361880	0.354575000
Cu	-0.61198540	0.08504980	0.53949770	0.013250000
Cu	0.21963950	-0.46066560	-1.60845210	0.003650000
Cu	-2.03225810	-1.29680770	-0.98324560	0.183725000
Cu	0.58475940	-2.14493100	0.21936510	0.053275000
Cu	-1.79978950	1.06955700	-1.44732730	0.294950000
O	3.20768510	1.38088920	0.67785410	-0.41067500
O	0.89494120	3.12652370	1.52188360	-0.48080000
O	-2.26218250	2.98029930	-2.04092140	-0.40975000
O	-2.74672590	-3.02737510	-0.94951110	-0.39002500
O	-0.06538880	-4.04838170	0.59306390	-0.43990000

O	1.04813020	-0.57161890	-3.50236910	-0.48662500
H	3.82249590	1.22480510	1.40282150	0.07687500
H	3.73876860	1.69035150	-0.06341460	0.13275000
H	1.65543370	2.60333380	1.24181660	0.12600000
H	0.25305290	2.46928720	1.84472550	0.14565000
H	-3.02812830	3.35910190	-1.59734750	0.13420000
H	-2.42554040	3.08087920	-2.98422110	0.14100000
H	-1.23337520	-0.54839810	3.38345150	-0.00070000
H	-2.09303280	-3.59478410	-0.53350340	0.03932500
H	0.51824160	-4.74673270	0.27978700	0.14200000
H	-0.21327000	-4.22467670	1.52810400	0.13800000
H	0.54204160	-1.16989150	-4.06169080	0.11547500
H	1.01924710	0.28041360	-3.94983020	0.13350000
C	-1.03048000	0.54080450	3.38055450	0.43067500
O	-0.87998330	1.06478940	2.22591990	-0.23285000
O	-0.97584610	1.14111310	4.44916050	-0.80157500

*CO_2

27

Cu 2.5167330 0.3796833 -0.8123885
Cu 0.6291230 1.1562262 0.6969348
Cu 0.0978312 -0.1592380 -1.1819915
Cu -1.6724741 0.2485541 0.5601089
Cu 1.5044925 -1.2132185 0.6580770
Cu -1.0713929 2.0522826 -1.0022139
O 3.9352149 1.4143301 -1.8512172
O 0.4238697 1.3015229 2.7550998
O -1.2532653 3.8010146 -2.1134371
O -2.7645927 -2.7473588 -0.6823172
O 1.0483095 -2.3635720 2.2679967
O -0.6238406 -1.5546108 -2.5227399
H 3.5853006 2.1585689 -2.3516178
H 4.6392670 1.7737014 -1.3017240

H -0.1657982 0.4865203 2.9154998
H -0.1032806 2.0642003 3.0077533
H -0.6315200 4.4866245 -1.8484642
H -2.1251614 4.2068668 -2.0659173
H -2.0363158 -2.3289033 -1.1549741
H -2.6447865 -2.5006430 0.2636028
H 0.7514485 -3.2517060 2.0481347
H 0.2626996 -1.8962083 2.6657818
H 0.0280831 -2.1589084 -2.8905920
H -1.1024431 -1.1829660 -3.2702826
C -1.7967521 -1.0643720 2.0321433
O -2.4021406 -2.1658038 1.9629547
O -1.0038416 -0.8527812 3.0455800

*H
25

Cu 0.6889184 2.1982099 -0.4241111
Cu 0.4110293 0.1812636 1.2417420
Cu -0.9706638 0.4201897 -0.8897497
Cu -0.2384645 -1.8649119 -0.0799346
Cu 1.4339255 -0.0604215 -1.1065853
Cu -1.8962478 -0.7231161 1.3541256
O 1.4319055 3.1655607 1.2630105
O 3.4898371 -0.8017506 1.5627238
O -4.3855563 0.8958572 0.0603444
O 0.3865694 -3.6731628 -0.7912546
O 3.3293961 -0.7959563 -0.9955413
O -2.6961813 0.7784168 -1.8979099
H 1.5223782 2.3685701 1.8618288
H 1.0367405 3.9270671 1.7276741
H 2.7453880 -0.2067149 1.8807304
H 3.5301171 -1.6031238 2.1135404
H -3.7089155 0.8185799 0.7827339

H -5.0752888 0.2185287 0.1987238

H 0.0200498 -4.5610833 -0.6174531

H 1.1772801 -3.6986204 -1.3622538

H 4.0591549 -0.3680815 -1.4761939

H 3.5112501 -0.8628426 0.0104073

H -2.6916906 1.4636717 -2.5860204

H -3.4789690 0.8491950 -1.2643136

H 1.4783227 0.7179374 2.3504920

*HCOO;*OH;*H (oxidated with hydride formation)

25

Cu	1.9726212	0.1686561	0.0479924
Cu	-0.4549940	0.1894530	0.4894273
Cu	0.3840770	-0.4670026	-1.7390751
Cu	-1.7724551	-1.5289099	-1.2718685
Cu	0.7582117	-1.9639157	0.2031516
Cu	-1.6093569	1.0805126	-1.5399069
O	3.6121415	1.3225076	0.3888227
O	-2.8197929	2.4845326	-2.4100206
O	-3.2009401	-1.8703572	-0.1302977
O	0.6397957	-3.8906865	0.8238989
O	1.6078110	0.1825573	-3.3400950
H	4.3918765	0.8194676	0.6458079
H	3.8777618	1.8514040	-0.3701375
H	-3.4258150	2.8965183	-1.7856100
H	-2.3363589	3.2075129	-2.8244316
H	-0.3311826	-0.7303708	3.2203789
H	-3.1053721	-1.3150222	0.6464516
H	0.7156996	-3.9983468	1.7781368
H	-0.1918990	-4.3081051	0.5768812
H	2.5231682	0.3069800	-3.0707265
H	1.6333754	-0.4356449	-4.0779941
C	-1.2243653	-0.0698013	3.2309035

O	-1.4999112	0.5015616	2.1266080
O	-1.8546482	0.0524625	4.2794807
H	-0.7078112	-1.5640291	-2.4654419

*HCOOH;*OH (oxidated)

28

Properties=species:S:1:pos:R:3:initial_charges:R:1 pbc="F F F"

Cu	2.19718540	-0.58168990	-0.11649480	0.38560000
Cu	-0.22138160	-0.06296630	0.28489390	0.39457500
Cu	0.40272410	-0.71342920	-1.87541450	0.49340000
Cu	-1.87392830	-1.43124490	-1.01974000	0.20712500
Cu	0.49892850	-2.39372640	0.03285800	0.34197500
Cu	-1.43565820	0.95078650	-1.63810700	0.23420000
O	4.11587130	0.00092650	0.51151170	-0.41630000
O	-2.30104620	2.92964160	-1.67217190	-0.42762500
O	-3.04293990	-2.90592310	-1.28189220	-0.52117500
O	0.33216280	-4.48677690	-0.06448290	-0.40340000
O	0.81351150	-1.86966810	-3.58273720	-0.38432500
H	4.21496070	0.08610270	1.46527420	0.13600000
H	4.38808720	0.84761960	0.14310210	0.16325000
H	-3.26147740	2.92725680	-1.59257840	0.17740000
H	-2.10398150	3.43534660	-2.46798100	0.14620000
H	-0.16423490	2.22831860	3.60517330	0.02147500
H	-2.86489650	-3.58454230	-0.62774610	0.14450000
H	0.38120260	-4.92566300	0.79073310	0.15750000
H	-0.51936820	-4.73335900	-0.44007050	0.07270000
H	1.75349880	-1.87887950	-3.78766620	0.12700000
H	0.37794640	-1.53311390	-4.37233560	0.14560000
C	-0.14128690	1.26689910	3.07750620	0.42282500
O	-0.48328600	1.35215040	1.79815630	-0.47250000
O	0.16378450	0.22775480	3.59322490	-0.62192500
H	-0.69241980	2.28841410	1.47327580	0.14125000
O	-0.95306340	3.64004320	0.74290820	-0.81030000
H	-1.44555860	3.48890230	-0.08391200	0.40480000

H	-1.47173540	4.26481880	1.25478640	0.40510000

*CHO;*OH (oxidated)

29

Properties=species:S:1:pos:R:3:initial_charges:R:1 pbc="F F F"

Cu	1.63290450	-0.09639790	-0.13342880	0.27587500
Cu	-0.89771870	-0.34758930	-0.04849140	0.51052500
Cu	0.30096930	-1.27548910	-1.92226580	0.42567500
Cu	-2.02136890	-2.09844780	-1.50555670	0.81025000
Cu	0.72592480	-2.27601570	0.34397500	0.37240000
Cu	-1.77987450	0.11466450	-2.37030510	0.08335000
O	2.81137850	1.58336030	-0.16592960	-0.59317500
O	-0.57263200	3.10424380	-0.64731860	-0.54922500
O	-2.51062400	-3.82925740	-0.85397230	-0.79347500
O	-0.32288690	-4.08217050	0.45936680	-0.42450000
O	1.38564540	-1.83968920	-3.60081820	-0.49355000
H	2.46679650	2.30063000	0.37592190	0.14075000
H	3.71564360	1.43869400	0.13129900	0.13925000
H	-0.98729560	2.74888280	-1.44186670	0.14000000
H	-2.58782660	-4.46909720	-1.56408160	0.05402500
H	-0.52686380	-4.35832260	1.35611330	0.03447500
H	-1.20707290	-4.04062560	-0.05024320	0.14100000
H	2.29389640	-1.52262690	-3.59069370	0.05647500
H	1.00651710	-1.52907210	-4.42857320	0.13900000
H	-1.03223020	2.66781920	0.10251780	0.08342500
C	-1.74727550	0.66660270	1.37477740	0.54875000
O	-1.80797750	1.90049260	1.49084600	-1.21620000
O	-1.90045770	2.03002200	-3.14350710	-0.41777500
H	-2.24576810	0.13492800	2.23459640	0.11147500
H	-1.42225910	2.17791670	-3.96612370	0.09542500
H	-2.78756020	2.37988500	-3.27790750	0.03755000
O	2.02870650	-3.12285900	1.98007380	-0.51337500
H	2.92749160	-2.78382360	1.94052530	0.13245000
H	2.11371330	-4.07922020	1.92390080	0.13850000

*CH_2O;*OH (oxidated)

30

Properties=species:S:1:pos:R:3:initial_charges:R:1 pbc="F F F"

Cu	1.94430490	-0.26856130	-0.31623120	0.31667500
Cu	-0.58471720	-0.32424000	-0.10842690	0.37670000
Cu	0.41051120	-1.29041130	-2.04601950	0.45105000
Cu	-1.95370880	-1.90567210	-1.53054180	0.28430000
Cu	0.89337260	-2.37805510	0.18270570	0.31380000
Cu	-1.55733910	0.29006150	-2.37699100	0.18150000
O	3.18386800	1.36745830	-0.28879850	-0.48827500
O	-2.23522920	3.23442070	-0.34769050	-0.67110000
O	-2.49385070	-3.62456460	-0.89817540	-0.42742500
O	-0.26932770	-4.09379920	0.29196000	-0.49180000
O	1.27795630	-1.93472960	-3.80774240	-0.62767500
H	2.97621010	1.99022170	0.41536660	0.13820000
H	4.11824770	1.15794390	-0.18797540	0.14360000
H	-2.01355680	2.89160660	-1.22040040	0.28190000
H	-2.66010940	-4.23470790	-1.61926820	0.13645000
H	-0.44329920	-4.40814840	1.18243240	0.12390000
H	-1.17174670	-3.96388230	-0.16515510	0.14110000
H	2.23298990	-1.82417670	-3.83630240	0.14250000
H	0.93581560	-1.49854880	-4.59373630	0.14275000
H	-2.31295230	2.43961860	0.23581870	0.28025000
C	-1.20013280	0.73247180	1.57741820	0.36295000
O	-2.42129060	1.11339050	1.30522930	-0.92416000
O	-1.67136980	2.20982620	-3.14382660	-0.32590000
H	-0.86789860	2.52400750	-3.57162290	0.20235000
H	-2.38919530	2.37160960	-3.76497560	0.17452500
H	-1.06209660	-0.01903040	2.37170430	0.00915000
H	-0.41027560	1.50522540	1.57419740	0.02087500
O	2.32305550	-3.30275070	1.67963990	-0.35005000
H	1.89509180	-3.90035530	2.30016970	0.12020000
H	2.71013610	-2.60829780	2.22034680	0.12515000

*CH_3O;*OH (oxidated)

31

Cu	1.6266546	-0.8711252	0.2809881
Cu	-0.8263702	-0.3653344	-0.0633559
Cu	0.3224730	-1.4272828	-1.8264649
Cu	-2.1139887	-1.8872789	-1.6383759
Cu	0.0242872	-2.6767579	0.3356033
Cu	-1.4506804	0.2808606	-2.4375718
O	3.1725274	0.2135496	0.5226844
O	-2.2884471	3.1002509	-0.3845112
O	-2.9648873	-3.5139883	-1.0849837
O	-1.1163456	-4.3249204	0.4398649
O	1.5680468	-2.0582224	-3.3204796
H	-2.0234516	2.8860767	-1.2927865
H	-3.1841690	-4.0881938	-1.8208049
H	-1.4652953	-4.5246551	1.3122703
H	-1.9156054	-4.0790288	-0.1696065
H	2.4925318	-2.0900800	-3.0550087
H	1.5377321	-1.5118355	-4.1116352
H	-3.2293484	3.2903965	-0.4302115
O	-1.4633941	2.2679116	-3.0564229
H	-0.6090876	2.6119123	-3.3397858
H	-2.0871284	2.4844693	-3.7580715
O	-1.6966796	0.9768426	1.1711483
H	-1.1271448	1.2657917	1.8890241
H	-1.9309081	1.7810111	0.6500605
C	3.9480730	0.6002471	-0.5714576
H	3.4787107	1.4126432	-1.1469902
H	4.1390920	-0.2196905	-1.2813959
H	4.9315753	0.9698613	-0.2424450
O	4.5895665	-0.5801220	2.5788165
H	4.0567338	-0.2856382	1.7850641

H 4.6739711 -1.5304587 2.4823647

*CH_3OH;*OH (oxidated)

32

Cu	1.7448568	-0.2286884	0.2570057
Cu	-0.7220721	0.0566949	-0.0278525
Cu	0.4962834	-0.9397049	-1.7899767
Cu	-1.8628400	-1.7983674	-1.2861068
Cu	0.2402654	-2.1756307	0.4468449
Cu	-1.5345482	0.3990123	-2.3514608
O	3.3882472	0.8779967	0.9307881
O	-2.6401511	3.3417141	-0.6534773
O	-2.3980246	-3.6504097	-1.0306315
O	-0.3614482	-4.1481942	0.4144980
O	1.3391380	-2.3498439	-3.1316954
H	3.6391773	0.6657583	1.8344326
H	-2.5657195	2.9787094	-1.5508944
H	-2.4492493	-4.1386537	-1.8547278
H	-0.6354898	-4.4930547	1.2681989
H	-1.2084505	-4.0528929	-0.1641153
H	2.2963462	-2.2688714	-3.1837078
H	1.0185339	-2.2228645	-4.0300137
H	-3.5830305	3.3871693	-0.4738157
O	-2.3259586	2.1437897	-3.2661311
H	-1.7162418	2.5989448	-3.8566220
H	-3.1394280	2.0151025	-3.7659061
O	-1.3733863	1.6778120	1.0778441
H	-0.6482702	2.1531671	1.4915177
H	-1.8211929	2.3154444	0.4765266
C	4.5610438	0.9111336	0.1125436
H	4.2363008	1.1575256	-0.8962642
H	5.0590877	-0.0600589	0.1047380
H	5.2499150	1.6775215	0.4701611

O	3.5399903	-2.7224125	1.6854535
H	4.1489528	-1.9793802	1.7049519
H	2.7355842	-2.3554981	1.2878512

CH_4 (desorbed);*OH (oxidated)

33

Cu	1.4841029	-0.6474371	1.4425362
Cu	-0.6730853	-0.2409309	0.1863940
Cu	1.0021056	-1.3911766	-0.9930526
Cu	-1.3604201	-1.9470267	-1.5602097
Cu	-0.0062009	-2.4991030	1.0294043
Cu	-0.5002725	0.1622812	-2.3220418
O	2.8071178	0.3978060	2.2862916
O	-2.0669115	3.0773520	-0.9793909
O	-2.4032639	-3.5041415	-1.1598605
O	-1.1231325	-4.1650160	0.9194534
O	2.7214062	-2.0676807	-1.8587105
H	3.5621076	0.4897139	1.7010937
H	-1.5437870	2.7937113	-1.7454876
H	-2.4065539	-4.1449871	-1.8729539
H	-1.7191524	-4.3030816	1.6596539
H	-1.6988800	-3.9846834	0.0793325
H	3.3369322	-2.2562888	-1.1182248
H	3.1453850	-1.4107327	-2.4179270
H	-2.9602499	3.1994270	-1.3120409
O	-0.4900385	2.0605274	-3.2129261
H	0.3772183	2.4661749	-3.3197899
H	-0.9241835	2.1183432	-4.0710257
O	-1.8796867	1.1951334	0.9391360
H	-1.5448974	1.5975060	1.7449351
H	-1.9760539	1.9187649	0.2755619
C	2.6771940	1.6617931	-1.3615090
H	1.6700064	1.7529231	-0.9527660

H 3.1721059 0.8140749 -0.8877819

H 3.2407781 2.5738542 -1.1657576

O 3.9296717 -2.6893003 0.5031946

H 4.4479297 -1.9656571 0.8695260

H 3.0609852 -2.5925498 0.9230279

H 2.6167003 1.4901208 -2.4363307

*HCOO;2*OH (oxidated; hydrogens removed)

26

Cu 2.1967913 -0.9561261 -0.802187331

Cu 0.7694701 0.7029727 0.567716446

Cu -0.2507584 -0.5056835 -1.222573016

Cu -1.5662026 -0.3785811 1.078949784

Cu 0.5082640 -1.8109922 0.8015364

Cu -1.5202277 1.5356469 -0.3096655

O -2.4109929 -1.6414054 2.6128076

O -0.4207780 -3.4070610 1.8163461

O -1.1653572 -1.0337839 -2.9627243

O 3.6121415 4.3225076 -1.7888227

H 3.4238156 1.8633632 0.4446362

H -2.3213847 -1.2407864 3.4843224

H 0.1376779 -3.7919067 2.4979934

H -1.1807314 -3.0075332 2.2722205

H -0.6007070 -0.9682402 -3.7391816

H -1.9559619 -0.5228883 -3.1632686

C 2.9378468 2.4169404 1.2748201

O 1.7350174 2.0783318 1.5338836

O 3.5751986 3.2876735 1.8610619

O -3.0405469 1.1622321 0.8829625

O 2.4674265 -2.4417254 0.4788844

H -3.3538477 -1.7914038 2.4849353

H -3.8053574 0.8414591 0.3992904

H 2.4589374 -3.3020446 0.0516143

H 4.3918765 4.8194676 -1.4458079

H 3.8777618 4.8514040 -2.3701376

*HCOO;3*OH (oxidated; hydrogens removed)

25

Cu 1.8520480 -0.2521005 -1.1493050

Cu 0.3295992 0.7440887 0.5085414

Cu -0.5528716 -0.4573694 -1.6212688

Cu -1.6965568 -0.8415672 0.6947034

Cu 0.5025359 -1.7605358 0.2729085

Cu -1.7533855 1.3965449 -0.6062260

O -1.0595926 -1.1522239 -3.4405717

H 3.1186400 0.8602675 1.3956750

H -0.3254209 -1.5228855 -3.9419588

H -1.4727843 -0.4977649 -4.0136086

C 2.5795588 1.5510470 2.0739618

O 1.3261201 1.6782299 1.8535518

O 3.2035510 2.1277905 2.9572239

O -0.9055645 -2.6634848 1.2501551

H -0.7435284 -2.6884994 2.1956300

O 3.5352493 -0.7040725 -1.7163801

O 3.5399903 -2.7224125 1.6854535

O -2.9053515 2.8138387 -0.8317038

O -3.5307137 -0.3038752 1.4135656

H -3.8975004 0.5034418 1.0370955

H -4.2020132 -0.9831106 1.2912892

H -3.8105458 2.4947170 -0.8105727

H 3.4802931 -1.4639996 -2.2995170

H 4.1489528 -1.9793802 1.7049519

H 2.7355842 -2.3554981 1.2878512

*HCOO;4*OH (oxidated;hydrogens removed)

24

Cu 2.1765532 -0.1810768 1.3265683

Cu -0.3089680 -0.1040063 1.3900358

Cu 0.9862406 0.5187089 -0.7836467

Cu -1.3013100 -3.1754070 -0.5341358

Cu 0.8451197 -1.9725201 0.3291242

Cu -1.8261184 -0.6219013 -1.0623970

O 2.7933825 0.8650751 -0.2306643

O -0.8062938 0.9203397 -1.3494302

O -2.8643514 -2.1795254 -0.8314313

O 0.3927039 -3.8238014 -0.0616470

O 1.4482153 -0.4291686 -2.8502462

H 3.4037154 0.3519388 -0.7681669

H -1.1693600 1.6443772 -0.8321817

H -0.9192563 2.0818115 3.2174979

H -3.3096920 -2.4314040 -1.6440137

H 0.8635055 -4.1536266 -0.8312117

H 2.0036341 0.1328241 -3.3985248

C -1.8380092 1.4622658 3.2192019

O -1.8039621 0.4161644 2.4878945

O -2.7910759 1.8178055 3.9062706

H 0.6035740 -0.4757316 -3.3088591

O -3.1953896 0.5245661 -5.0317247

H -3.6939923 -0.1736554 -5.4679283

H -2.6180653 0.8919248 -5.7089186

*HCOO;5*OH (oxidated;hydrogens removed)

23

Cu 1.8561325 0.3101453 -0.9118769

Cu -0.6070296 -0.3224925 0.0650858

Cu -0.7171888 1.0955310 -2.2084670

Cu -1.2848915 -2.8715064 0.0736301

Cu 1.4110008 -1.9805452 0.7473731

Cu -2.6333198 -0.8517513 -1.5996963
O 2.7152338 -0.6878937 0.4136419
O -2.5757224 0.9117106 -2.2092923
O -2.7575220 -2.6201706 -1.0349624
O 0.1810683 -3.3259794 1.1312689
O 1.1242615 1.3568590 -2.2680339
O -3.1953896 0.5245661 -5.0317247
H 3.4938743 -1.1103429 0.0393519
H -2.8968753 0.9345668 -3.1162455
H 0.4557217 -0.1385290 2.7414958
H -3.5597943 -2.7317917 -0.5159033
H -0.0631678 -3.2744856 2.0601612
H 1.3179086 2.2829598 -2.0959832
H -3.6939923 -0.1736554 -5.4679283
H -2.6180653 0.8919248 -5.7089186
C -0.2366416 0.7270273 2.6866983
O -0.8936909 0.8402630 1.5986973
O -0.3176994 1.4843365 3.6494524

*COOH
28

Cu 2.6020191 0.1400395 -0.7504445
Cu 0.6786960 1.1044387 0.5153582
Cu 0.1735298 -0.1922715 -1.4163150
Cu -1.4081032 -0.3063658 0.4955434
Cu 1.1509596 -1.3937364 0.5053183
Cu -1.2864761 1.7608947 -0.9287416
O 4.3255457 0.5466033 -1.8608774
O 0.5815493 1.4469870 2.6485629
O -2.3535025 3.3006683 -1.8864980
O 0.9553173 -2.4624398 2.3057280
O 0.2774308 -0.5476461 -3.4938266
H 4.2706258 1.3801190 -2.3395537

H 5.1175853 0.6107875 -1.3171371
H 0.0192561 0.7158364 2.9630397
H 0.0776225 2.2471942 2.8237428
H -2.0896323 4.1805092 -1.5979959
H -3.3045228 3.2540281 -1.7446271
H 0.6101366 -3.3498277 2.1656973
H 0.2408037 -1.9683984 2.7516679
H 1.1824200 -0.5963531 -3.8167264
H -0.1396552 0.1592622 -3.9953540
C -2.1229410 -0.8591323 2.2527142
O -3.2160910 -1.2020575 2.6518155
O -1.1211426 -0.8042833 3.2666932
H -1.5347320 -1.0326797 4.1158772
O 5.3482223 -1.8205791 -1.1161954
H 5.1134240 -2.2585678 -0.3342968
H 5.6833123 -2.6424850 -0.7342389

*COOH(A)
28

Cu 2.6125549 0.1459766 -0.7656388
Cu 0.6964035 1.1102161 0.5088837
Cu 0.1807830 -0.1921416 -1.4167135
Cu -1.3921855 -0.3042837 0.4993214
Cu 1.1685583 -1.3906811 0.4994103
Cu -1.2757876 1.7622068 -0.9263321
O 4.3325686 0.5495547 -1.8883783
O 0.6144614 1.4625172 2.6449977
O -2.3486850 3.3001664 -1.8836504
O 1.0051435 -2.4622603 2.3064265
O 0.2776195 -0.5472112 -3.4974858
H 4.2738413 1.3823454 -2.3675958
H 5.1281933 0.6149656 -1.3501599

H 0.0584240 0.7383735 2.9712794

H 0.1107446 2.2638790 2.8171550

H -2.0831488 4.1797696 -1.5966698

H -3.2992876 3.2543701 -1.7387929

H 0.6601900 -3.3495642 2.1680541

H 0.3008023 -1.9764896 2.7648833

H 1.1812876 -0.5947706 -3.8243634

H -0.1412757 0.1590179 -3.9985885

C -2.1180840 -0.8381990 2.2495271

O -3.2839012 -1.2227463 2.6933610

O -1.2258131 -0.8387092 3.3172836

H -4.0845267 -0.6877737 2.7247814

O 5.3732223 -1.8205791 -1.1161954

H 4.7234240 -2.2585678 -0.3342968

H 5.2933123 -2.6424850 -0.7342389

*CO

29

Cu 2.2589509 0.5309331 -0.3829373

Cu 0.1797606 1.2746224 0.6076276

Cu -0.0597048 0.0245126 -1.3903648

Cu -1.7397617 -0.3798213 0.2899899

Cu 0.5512448 -1.4156571 0.5089008

Cu -1.8234327 1.7775726 -0.9855285

O 4.1845740 -0.1266802 -1.0042920

O -0.3708937 1.9219505 2.5496188

O -2.5358686 3.4727433 -2.0818960

O 1.9910375 -2.6583288 1.5206183

O 0.3753144 -0.3903078 -3.4029833

O -4.3482223 -1.8205791 -1.1161954

H 4.8758571 0.5305914 -0.8750257

H 4.4742455 -0.9061491 -0.5191120

H 0.0834335 1.4214044 3.2341287

H -1.3101324 1.7623969 2.6907564

H -2.1009101 4.2866799 -1.8075371

H -3.4785022 3.6340303 -1.9703000

H 1.5934152 -3.2986110 2.1189732

H 2.5870020 -2.1319014 2.0627968

H 1.1219422 0.1308287 -3.7123308

H -0.3603492 -0.1526325 -3.9750485

H -4.1134240 -2.2585678 -0.3342968

H -4.6833123 -2.6424850 -0.7342389

O -5.3732223 4.2205791 -1.1161954

H -4.7234240 4.8585678 -0.3342968

H -5.2933123 4.4424850 -0.7342389

C -2.9659714 -0.8058421 1.6169213

O -3.6847269 -1.1928685 2.4079932

*$C(OH)_2$

29

Cu	2.4772096	0.4020255	-0.6772081
Cu	0.6106060	1.2072514	0.5965332
Cu	0.0012863	-0.0866368	-1.3499853
Cu	-1.3991873	-0.4139605	0.5571980
Cu	0.9251884	-1.4699176	0.4248599
Cu	-1.5350333	1.7855652	-0.7285956
O	4.1438449	-0.1792984	-1.8695709
O	0.2676782	1.7248159	2.7013080
O	-2.6772453	3.4880053	-1.4155738
O	2.2102327	-2.7153318	1.6956634
O	0.3403752	-0.2778809	-3.4410567
H	4.7432300	0.5476181	-2.0679038
H	4.6846272	-0.8510752	-1.4416518
H	-0.4541628	1.1663600	3.0233265
H	-0.0798483	2.6217361	2.7273226
H	-2.4803981	4.2775460	-0.9009894

H	-3.6268385	3.3551158	-1.3277274
H	1.6983919	-3.2557590	2.3054099
H	2.7681340	-2.1604539	2.2495996
H	1.1980280	-0.6845539	-3.5994024
H	0.3887143	0.5875587	-3.8579759
C	-2.4283128	-0.5800774	2.1826331
O	-3.5839169	-1.1750228	2.3593083
O	-2.0110729	-0.0802678	3.3240699
H	-2.6280361	-0.2456159	4.0586651
H	-3.8909686	-1.5486811	1.5236581
H	-5.3732223	4.2205791	-1.1161954
H	-4.7234240	4.8585678	-0.3342968
O	-5.2933123	4.4424850	-0.7342389

*CHO

30

Cu	2.3994663	0.2825936	-0.5892920
Cu	0.3608372	1.2769692	0.4612252
Cu	0.0363214	0.0079298	-1.4910339
Cu	-1.7027867	-0.1807809	0.2841877
Cu	0.7729005	-1.2442575	0.5031675
Cu	-1.5105027	1.9392274	-1.0803646
O	4.3497809	0.3757900	-1.3776743
O	0.3411924	1.9619884	2.4580868
O	-2.5997086	3.4345537	-2.1014321
O	0.7597143	-2.5228605	2.1872772
O	0.1833899	-0.4413143	-3.5297621
H	-5.3732223	4.8205791	-1.1161954
O	3.1482223	-1.8205791	-1.1161954
H	2.7134240	-2.2585678	-0.3342968
H	3.3833123	-2.6424850	-0.7342389
H	-4.7234240	4.2585678	-0.3342968
O	-5.2933123	4.6424850	-0.7342389
H	4.8162957	1.1848858	-1.1439825

H	4.9098451	-0.3445436	-1.0705378
H	0.8314423	1.3652484	3.0320083
H	-0.5558136	1.9787224	2.8074893
H	-2.4076203	4.3204900	-1.7769432
H	-3.5532245	3.3297534	-2.0191018
H	-0.1299371	-2.6080042	2.5469793
H	1.2997060	-2.1857764	2.9093360
H	1.0932493	-0.5609871	-3.8185155
H	-0.1759021	0.2581238	-4.0836622
C	-2.7963716	-1.0874396	1.6349206
O	-3.8950422	-1.6345684	1.5146098
H	-2.3632695	-1.1907049	2.6834339

*CHOH;*OH

30

Cu	0.4167287	-1.0471894	-0.4588791
Cu	0.9248565	-0.4247593	-2.6618829
Cu	-0.5689433	0.8304187	-1.2036809
Cu	-2.5275559	1.6856529	-0.1049401
Cu	-2.2099083	-0.5682017	-0.0654877
Cu	0.0804670	0.0881481	1.5729875
O	0.2709050	-2.6821098	0.7809691
C	0.5578815	-1.7271192	1.8903520
O	0.1959275	2.0958864	1.5309249
O	3.1812223	-1.8205791	-1.1161954
H	2.7454240	-2.2585678	-0.3342968
H	3.6133123	-2.6424850	-0.7342389
H	-0.5462412	2.6231443	1.0415001
H	1.0633275	2.2683134	1.0765735
O	-3.4548418	3.7944963	-1.2331062
H	-4.2162231	4.3402147	-1.1324863
H	-3.4435896	3.6482846	-2.2019043
O	2.2675420	2.0511069	-0.1784438

O	2.1567059	-1.1446498	-4.1678347
O	-2.4110408	-2.4041297	0.3595147
H	-3.1013905	-2.2861522	1.0749064
H	-0.7089302	-2.6638217	0.6966307
H	2.0781167	2.8414052	-0.7200297
H	1.7739096	1.2507060	-0.5566958
H	1.9037985	-1.7040346	-4.8963083
H	2.5591060	-0.3380755	-4.5813120
H	0.3875570	-1.2369741	2.7445681
H	-5.3732223	4.2205791	-1.1161954
H	-4.7234240	4.8585678	-0.3342968
O	-5.2933123	4.4424850	-0.7342389

*CH_2O

31

Cu	0.0645708	-1.5487009	-0.7727132
Cu	1.3378873	-0.4284018	-2.5768885
Cu	-0.5535736	0.6555417	-1.6120172
Cu	-2.3873836	2.0844354	-0.9605260
Cu	-2.1559481	-0.1695884	0.0042638
Cu	0.4250496	-0.0861635	1.4418909
O	-0.1850493	-2.8005755	0.9578129
C	0.5518133	-1.9596888	1.9296243
O	2.9812486	-1.8205791	-1.1161954
H	2.5454420	-2.2585678	-0.3342968
H	3.4162598	-2.6448500	-0.7342389
H	0.1293330	-2.1732596	2.9178242
H	1.5881573	-2.3112612	1.9381670
O	0.3488932	1.8713545	1.1942288
H	-0.4688046	1.9450141	0.6581839
H	1.0541843	2.1647761	0.5710026
H	-5.3732223	4.2205791	-1.1161954
H	-4.7234240	4.8585678	-0.3342968

O	-5.2933123	4.4424850	-0.7342389
O	-3.6159087	3.7705788	-0.9697025
H	-4.1983455	3.8123940	-0.2042553
H	-4.1939145	3.8157859	-1.7384905
O	2.0804631	2.5238177	-0.8183851
O	2.9881258	-0.9411838	-3.7503048
O	-2.7506941	-1.8017769	1.1778781
H	-2.9514009	-1.5682937	2.0896210
H	-1.9109609	-2.3075205	1.2028259
H	1.8870344	3.4081356	-1.1466413
H	1.5992977	1.9236624	-1.4302474
H	2.8599868	-1.7489201	-4.2581990
H	3.2259832	-0.2652248	-4.3930506

CH_3OH;*OH

32

Cu	0.4167287	-1.0471894	-0.4588791
Cu	0.9248565	-0.4247593	-2.6618829
Cu	-0.5689433	0.8304187	-1.2036809
Cu	-2.5275559	1.6856529	-0.1049401
Cu	-2.2099083	-0.5682017	-0.0654877
Cu	0.0804670	0.0881481	1.5729875
O	0.2709050	-2.68210980	0.7809691
O	0.1959275	2.09588640	1.5309249
H	-0.5462412	2.62314430	1.0415001
H	1.0633275	2.26831340	1.0765735
O	-3.4548418	3.79449630	-1.2331062
H	-5.3732223	4.2205791	-1.1161954
H	-4.7234240	4.8585678	-0.3342968
O	-5.2933123	4.4424850	-0.7342389
H	-4.2162231	4.3402147	-1.1324863
H	-3.4435896	3.6482846	-2.2019043
O	2.2675420	2.0511069	-0.1784438

O 2.1567059 -1.1446498 -4.1678347

O -2.4110408 -2.4041297 0.3595147

O -1.6159086 3.7705788 -0.9697025

H -2.1983455 3.8123940 -0.2042553

H -2.1939145 3.8157859 -1.7384906

H -3.1013905 -2.2861522 1.0749064

H -0.7089302 -2.6638217 0.6966307

C 2.0781167 2.8414052 -0.7200297

H 1.7739096 1.2507060 -0.5566958

H 1.9037985 -1.7040346 -4.8963083

H 2.5591060 -0.3380755 -4.5813120

H 2.5781167 3.3414052 -0.2200297

H 1.4781167 3.4414052 -0.7200297

H 2.5781167 3.3414052 -1.3200297

H 0.8709050 -2.1821098 0.7809691

Structures with explicite water molecules attached to the optimized structures

COOH;H_2O

13

Cu -0.5186684 -0.0708939 1.3410407

Cu 1.4018969 0.7900135 -0.0532816

Cu 0.7718099 -1.8697102 0.1690000

Cu -0.6283055 -0.2421869 -1.1198400

Cu -1.5979086 -2.1049860 0.2908205

Cu -0.6780843 2.0185282 -0.0292871

C 3.1042006 -0.1921008 -0.1035285

O 2.8197224 -1.5783092 0.0907571

O 4.2692906 0.0686766 -0.2742509

H 3.6279341 -2.1132803 0.0277511

O -3.5057585 -2.8208294 0.3793238

H -4.1805285 -2.1376244 0.3119779

H -3.7129079 -3.4650452 -0.3053899

*COOH;2 H_2O

16

Cu -0.4162650 -0.1501135 1.3288002

Cu 1.4801838 0.6919340 -0.0412587

Cu 0.8296385 -1.9878494 0.1592488

Cu -0.5305717 -0.3050823 -1.1091529

Cu -1.5380950 -2.1828301 0.2766347

Cu -0.6008495 1.9692964 -0.0155716

C 3.1826885 -0.3039530 -0.0965624

O 2.8753493 -1.6908029 0.1015506

O 4.3523116 -0.0690860 -0.2746624

H 3.6752168 -2.2367999 0.0279249

O -3.4814906 -2.8302324 0.3817272

H -4.1294114 -2.1201521 0.3303039

H -3.7185194 -3.4537638 -0.3123753

O -1.8483578 3.6030834 -0.0501023

H -1.5742596 4.5222949 -0.0039117

H -2.8062603 3.5857629 -0.1103282

CO;H_2O

11

Cu -0.8554805 1.5129574 -0.7047805

Cu 0.1931656 0.0158730 0.9146937

Cu 1.6135380 1.5021760 -0.4357906

Cu 0.3626103 -0.6372343 -1.3158561

Cu 2.3743223 -0.8658660 0.0619589

Cu -1.8596823 -0.8333127 -0.2884163

C 0.0637423 0.1447996 2.7201044

O -0.0175250 0.2185490 3.8495512

O -3.8355348 -1.4560485 -0.2192307

H -3.9569616 -2.4086855 -0.2854986

H -4.3889045 -1.0724267 -0.9068684

*CO;2 *H_2O

14

Cu -0.9574093 1.5768410 -0.3984232

Cu 0.1295946 -0.0569161 1.0702561

Cu 1.5298413 1.5013036 -0.2431823

Cu 0.2200505 -0.4881825 -1.2434153

Cu 2.2336527 -0.9210181 0.1346586

Cu -1.9419628 -0.8154452 -0.0630758

C 0.0768246 0.2325573 2.8686150

O 0.0345904 0.3989183 3.9910925

O -3.9032045 -1.5022157 -0.0909154

H -3.9828570 -2.4567294 -0.1866068

H -4.4323097 -1.1231541 -0.8000704

O 0.3419965 -0.6643027 -3.3095188

H 0.4720762 -1.5757049 -3.5899760

H 1.0911066 -0.1755614 -3.6644991

CHO;H_2O

12

Cu -0.4694153 0.2195466 1.3135364

Cu 1.3780853 1.0011675 -0.2082239

Cu 0.7301867 -1.6109249 0.0766255

Cu -0.7521579 0.0362580 -1.1287917

Cu -1.6338908 -1.8141224 0.3580776

Cu -0.6596464 2.3074757 -0.0644555

C 2.9260482 -0.1820972 -0.3066436

O 2.7605971 -1.4156752 -0.1677495

H 3.9993966 0.0760174 -0.4783298

O -3.5540705 -2.4667833 0.5902189

H -3.6676398 -3.1359867 1.2729028

H -3.9474300 -2.8338892 -0.2079059

*CHO;2 *H_2O

15

Cu -0.3349896 -0.0454093 1.4215031

Cu 1.4582666 0.8700879 -0.0385394

Cu 0.7694714 -1.7635100 -0.0559783

Cu -0.6952392 0.0713931 -0.9900204

Cu -1.5806020 -1.9528135 0.2845494

Cu -0.5496254 2.2206835 0.3218310

C 2.9877007 -0.3145070 -0.3525045

O 2.7860193 -1.5523422 -0.3742786

H 4.0649054 -0.0714356 -0.5319278

O -3.5199870 -2.5836868 0.5271187

H -3.6565422 -3.1440548 1.2979065

H -3.8702287 -3.0759167 -0.2223097

O -1.8062324 3.8552246 0.6331962

H -1.5387383 4.6416226 0.1464266

H -2.7146859 3.6813340 0.3652949

CH_2O;H_2O

13

Cu 1.2335619 -1.0487346 -0.5513365

Cu 2.4770032 0.7181748 -1.5439363

Cu 0.0534315 0.9720290 -1.2955290

Cu -2.3254947 1.2920502 -0.8304607

Cu -1.2954404 -0.7571430 -0.1957924

Cu 0.1529258 0.1320158 1.8701239

O -0.0728846 -2.2164047 0.3557627

C 0.1254353 -1.8107286 1.7433696

H -0.6841176 -2.2388099 2.3516879

H 1.0508794 -2.2688596 2.1204630
O 0.1049305 2.1196328 1.9171447
H -0.3201541 2.4912075 2.6977180
H -0.4437632 2.3820658 1.1646373

*CH_2O;2 *H_2O
16

Cu 1.5827948 -0.8559979 -0.1331973
Cu 2.3915666 1.2344090 -0.9365668
Cu -0.0092981 0.7380360 -1.1596036
Cu -2.4391467 0.3043953 -1.2462436
Cu -0.9426693 -1.2741199 -0.2455110
Cu 0.2708481 0.0533545 2.0098560
O 0.4904027 -2.3520323 0.5942444
C 0.4025173 -1.8897428 1.9720501
H -0.4605169 -2.3693935 2.4556466
H 1.2863616 -2.2473609 2.5203561
O 0.2573916 2.0456893 1.9854792
H -0.6261381 2.4291928 1.9509348
H 0.6834175 2.2867037 1.1498850
O -4.2867916 1.0051874 -1.8407411
H -4.4142769 1.9529607 -1.7279651
H -4.5068503 0.8171376 -2.7592522

CH_3O;H_2O
14

Cu -0.4325861 0.1021689 1.3038263
Cu 1.5091712 0.5622616 -0.2415239
Cu 0.3641035 -1.6540225 -0.3117329
Cu -1.0017422 0.4830184 -1.0470312
Cu -1.9909842 -1.5006841 0.0622507
Cu 0.0094964 2.3696477 0.2020296

O 2.1732848 -1.1670820 -0.9510881
C 3.3294639 -1.7763667 -0.4410853
H 4.2309793 -1.2451571 -0.7731486
H 3.4127471 -2.8138630 -0.7904906
H 3.3484444 -1.7989962 0.6574451
O -1.1431138 0.3751055 3.2386404
H -1.3573512 1.2911956 3.4412541
H -1.9435202 -0.1285230 3.4166050

$*CH_3O;2\ *H_2O$
17

Cu -0.3312391 -0.0084314 1.2050149
Cu 1.6000268 0.6117608 -0.2826742
Cu 0.5703582 -1.6604522 -0.4497233
Cu -0.8881331 0.2943402 -1.1577598
Cu -1.7864592 -1.7601930 -0.0085204
Cu -0.0583744 2.2835652 0.1066990
O 2.3939608 -1.0743557 -0.9863574
C 3.5466393 -1.6158250 -0.3995880
H 4.4297896 -1.0044353 -0.6281455
H 3.7419619 -2.6284262 -0.7771759
H 3.4727827 -1.6857163 0.6950984
O -1.1949566 0.1099210 3.1026192
H -1.5225271 0.9867719 3.3260915
H -1.9485742 -0.4823941 3.1896089
O -3.6822285 -2.4192425 0.4925089
H -4.3671060 -1.7732545 0.2914484
H -3.9451685 -3.2269797 0.0391311

$*O;*H_2O$
10

Cu 2.4612757 -0.2670902 0.6930871

Cu 0.5657052 1.2009238 -0.5694935
Cu 0.2614617 -1.3503224 -0.5282650
Cu -1.5632350 0.1621901 0.1211574
Cu -1.8316876 -2.2776033 0.1543142
Cu -1.2614751 2.5942966 0.0928890
O 1.7635688 -0.2486570 -0.9836498
O 3.1718176 -0.2888221 2.4912726
H 3.4751811 0.5729282 2.7972523
H 3.9153765 -0.8903537 2.6075260

*O;2 *H_2O

13

Cu 2.5235903 -0.1286944 0.6878535
Cu 0.7006304 1.3987906 -0.6111419
Cu 0.3436346 -1.1372666 -0.6021688
Cu -1.4558778 0.3343963 0.0031532
Cu -1.7658924 -2.1226020 0.0213662
Cu -1.1353949 2.7817913 0.0395627
O 1.8908451 -0.0634702 -1.0131819
O 3.1647331 -0.1789424 2.5112334
H 3.8813253 0.4394372 2.6901299
H 3.4815835 -1.0445276 2.7915795
O -3.1107637 -3.6751042 0.3715243
H -2.6792902 -4.4957704 0.6311079
H -3.7445236 -3.4825757 1.0703768

OH;H_2O

11

Cu -0.3688798 0.3227868 1.1785047
Cu 1.6536873 0.1055828 -0.2711416
Cu 0.0101145 -1.8009892 -0.1682413
Cu -0.6158407 0.3764478 -1.2858322

Cu -2.2293415 -1.0088810 0.0979634
Cu 0.6184798 2.2679612 -0.1275500
O 1.9739974 -1.8609828 -0.3548477
H 2.3877277 -2.1846597 0.4478715
O 0.1064818 4.2691190 -0.0861369
H -0.5756620 4.4588011 0.5660855
H 0.8390213 4.8581953 0.1225819

*OH;2 *H_2O
14

Cu -0.1773178 0.1881335 1.2548552
Cu 1.7781652 0.1617296 -0.3113525
Cu 0.2963685 -1.8642579 -0.0725217
Cu -0.5586942 0.2127015 -1.1815435
Cu -2.0136048 -1.2912693 0.2648968
Cu 0.5635983 2.2267721 -0.0881892
O 2.2543378 -1.7722953 -0.4425585
H 2.7713939 -2.0630942 0.3112363
O -0.0993397 4.1880625 0.0222508
H -0.3084263 4.4715483 0.9183233
H 0.5276248 4.8346744 -0.3174919
O -4.0621773 -1.2431779 0.6216843
H -4.5254120 -2.0247872 0.3029571
H -4.4933804 -0.4955304 0.1948470

$Cu_6[H_2O]_{35}$ without directly attached explicit water molecules

*
111
Lattice="30.020263 0.0 0.0 0.0 30.8680216 0.0 0.0 0.0 32.2339827" Properties=species:S:1:pos:R:3:forces:R:3 I
Cu 16.76930918 16.03089400 17.18543395 -0.07701658 -0.01515432 -0.06880893
Cu 14.40400842 15.44596492 17.41376034 0.01326531 -0.03028996 0.00470561
Cu 15.34289917 15.94522783 15.30101776 0.02189371 -0.01645098 -0.06790948
Cu 13.89033679 13.99129242 15.43711395 0.00979629 0.04594502 0.01766472
Cu 15.94499441 13.86439038 16.57867082 -0.02257121 0.03268337 0.03338689

Cu	13.01897670	16.21564680	15.47891511	0.05496465	-0.00200643	-0.01890035
H	11.70318433	13.51537591	18.69612569	0.01487986	0.01470507	-0.02243305
O	11.22676757	14.22288592	18.14470490	0.01552529	-0.00289901	-0.01443309
H	11.30031457	13.80811375	17.22882249	0.03644056	0.01317131	-0.00891735
H	19.05950845	14.11445510	15.86101738	-0.03509853	0.01364348	0.00248504
O	18.79735521	14.71051080	15.10391050	-0.01362892	-0.00588538	0.00232187
H	17.95634581	14.28308301	14.76516710	-0.02147453	0.01902415	0.01901208
H	14.38013260	14.96219923	21.62368844	0.00153615	-0.00050017	-0.01687098
O	14.75395606	15.24581869	20.76269822	0.00701736	-0.00378222	-0.02061825
H	15.18945767	14.41305645	20.39489151	0.00178302	0.01322947	-0.03255850
H	14.85940051	11.97168632	14.96337006	0.00619916	0.00004982	-0.00257925
O	14.68432200	11.05660180	14.57678441	0.01216286	0.01835162	0.00370628
H	14.08813795	11.31962568	13.80106888	0.01235412	0.02091195	0.01919180
H	14.09195840	18.37785104	12.35086041	-0.00247016	-0.01302499	0.02320662
O	13.24039172	18.25798868	12.92179643	0.00996396	-0.00437113	0.01914221
H	13.51028065	18.66570701	13.80929338	0.00457831	-0.02857602	0.01942522
H	11.47303345	18.43003930	13.39376161	0.00778468	-0.01513052	0.01593972
O	10.64174827	18.41991575	13.94103591	0.02256225	-0.01973375	0.01158036
H	10.81275609	17.71097260	14.60534242	0.01987118	-0.00215719	-0.00234760
H	15.72227425	12.26764958	19.03289170	-0.00235263	0.02556409	-0.02187890
O	15.75846479	12.88228770	19.90875104	-0.00172331	0.01248940	-0.01612523
H	15.80052996	12.22980392	20.64207215	-0.00523881	0.00474720	-0.01417253
H	14.70564133	20.16570206	14.67237438	-0.00266287	-0.01874215	0.00577310
O	13.86566071	19.80727432	15.03630062	0.00381565	-0.02831808	0.00107206
H	14.17145083	19.35013270	15.90292943	0.00056896	-0.03093445	-0.00194562
H	13.03686489	18.53772161	20.38689029	0.00214165	-0.00895000	-0.01591739
O	12.08458895	18.35828644	20.11301095	0.01620385	-0.01637860	-0.01506286
H	12.19347888	17.42826355	19.78823441	0.01142953	-0.00298942	-0.01577407
H	14.94127803	17.26886542	20.79691921	0.00281626	-0.00128536	-0.02529094
O	14.77710631	18.23899019	20.86942008	0.00147622	-0.01866309	-0.02426386
H	15.48772841	18.61904167	20.29062668	-0.00847228	-0.01495141	-0.01153804
H	15.65465634	14.81845588	11.81483768	-0.01277261	0.00521312	0.02627622
O	15.04461757	14.22745345	12.36548838	-0.00029628	0.01020504	0.01406991
H	14.30854199	14.86243802	12.64331337	0.00935618	0.00855895	0.04305129
H	13.71303218	12.84675068	19.39951846	-0.00011807	0.01908553	-0.03050730
O	12.81932509	12.42902855	19.33673235	0.02531024	0.01606848	-0.01621888
H	12.93786108	11.79438367	18.58266008	0.01306856	0.02126434	-0.00539077
H	11.61078693	15.06239133	13.41695595	0.01599553	-0.00251285	0.01869803
O	10.82253609	14.66338977	13.91628092	0.01314354	0.00703005	0.00324634
H	10.53460686	15.41475046	14.48404232	0.02039588	-0.00409518	-0.00000815
H	12.53376475	15.85087595	11.58488844	0.00870423	0.00210289	0.01860873
O	12.85078908	15.77563368	12.51282106	0.00984086	-0.00185492	0.00968736
H	13.04502054	16.77109206	12.79257349	0.01407352	-0.01071347	0.01661771
H	17.36787509	16.18851304	12.12748560	-0.01281414	-0.00656182	0.01870874
O	16.72075769	16.00541401	11.31768810	-0.00882423	-0.00370053	0.01992100

H	17.29410247	15.97831967	10.52428342	-0.00270787	0.00023279	0.01193572
H	17.16724125	18.92671264	15.13887061	-0.02264083	-0.02608087	0.00800221
O	16.59402509	18.59777531	14.36618179	-0.01376190	-0.01135613	0.01194145
H	16.00631868	17.90054768	14.82426126	-0.00953102	-0.01058610	0.01555597
H	17.33819544	13.63798505	19.73997574	-0.00719084	0.01320602	-0.02915122
O	18.24828217	14.03767927	19.62745528	-0.01683049	-0.00455886	-0.01712974
H	18.55893210	13.61630787	18.78033654	-0.02675408	0.01124754	-0.01482097
H	17.56754574	17.16369650	19.08030564	-0.00500030	-0.00377651	0.00911150
O	18.14021915	16.42832528	18.69583333	-0.02153218	-0.00405842	-0.01453177
H	18.12243528	15.54035959	19.23004130	-0.00527308	-0.00120227	0.00234640
H	15.90083293	17.56211984	11.48073564	-0.01002686	-0.00441207	0.02079350
O	15.55286460	18.47322533	11.71214002	0.00242767	-0.01688750	0.02155244
H	16.05004967	18.68595798	12.54492089	-0.00583122	-0.01365633	0.00756259
H	14.71007486	11.34466343	17.52143059	0.01173799	0.03184825	-0.01381878
O	15.61930520	11.26261590	17.96952117	-0.00075775	0.01095255	-0.01345382
H	16.25284120	11.45609563	17.22230759	-0.01099420	-0.00002363	0.00714865
H	15.74839975	18.54286579	18.28909918	-0.00115721	-0.02987011	-0.01838426
O	16.61819332	18.61639794	18.80221793	-0.00533195	-0.00487093	-0.01449481
H	17.21429130	19.02006653	18.10868598	-0.01470857	-0.02247656	-0.00823020
H	11.15431994	13.58785064	14.99659139	0.02370539	0.01007022	0.01033418
O	11.28102686	12.88659630	15.74899222	0.01837790	0.00747083	0.00178851
H	10.49164628	12.30386884	15.68953998	0.01776007	0.01122368	-0.00139912
H	11.38524800	17.51292419	16.90874055	0.00662487	-0.00299266	0.00714199
O	11.17150489	16.66595001	16.29632067	0.03141942	-0.00631795	0.00427493
H	10.97032655	15.94069747	16.93741277	0.00571868	0.00504589	0.00018591
H	11.51350134	19.52641007	16.96113892	0.01060216	-0.01637250	-0.00349034
O	11.85812319	18.73310903	17.55881436	0.01337666	-0.01273139	-0.00840522
H	11.77106363	18.83040292	18.57653481	0.00896121	-0.00966491	-0.01623178
H	19.25869084	16.76922067	17.43179092	0.00312487	-0.00153892	-0.00749674
O	19.57320260	17.03481232	16.51716716	-0.01316234	-0.00797272	-0.00945152
H	19.20369440	16.29977111	15.95168025	-0.01936464	0.00225839	0.00481628
H	18.76413797	19.92181481	16.40519504	-0.00662409	-0.01377669	0.00186011
O	18.13313249	19.17759150	16.50296515	-0.01679495	-0.01715481	-0.00081557
H	18.73061081	18.32102367	16.44932147	-0.02175001	-0.01788196	0.00061015
H	12.68975898	11.84913894	16.42626680	0.01351661	0.00927709	0.00374051
O	13.19849087	11.07305999	16.79496789	0.00010291	0.02179518	-0.00382890
H	13.69535739	10.79508570	15.95403751	0.00377954	0.02698393	0.00448661
H	17.45158853	12.89331985	13.04799428	-0.01487363	0.01337898	0.01284972
O	16.93619766	13.20816082	13.82347229	-0.01216973	0.00975819	0.00229772
H	16.08147229	13.61459035	13.39987411	-0.01559817	0.01486449	0.03996400
H	12.67495013	12.78244113	13.44368547	0.00080064	0.01153093	0.00350630
O	13.07446850	12.24688583	12.71533711	0.00098395	0.02165757	0.01652658
H	13.76829590	12.87057772	12.36662851	0.00041840	0.01238729	0.02146426
H	12.18926144	20.49183744	15.45032923	0.00003764	-0.01751951	0.00034785
O	11.24294286	20.40553121	15.74772247	0.01412239	-0.01769549	-0.00305546

H	10.89272407	19.75326010	15.05995541	0.01444338	-0.00756374	0.00728664
H	14.58683468	17.68718250	16.85953416	0.00120814	0.03980708	0.01926727
O	14.44579754	18.60168171	17.27524397	-0.00073148	-0.00520649	0.00264721
H	13.44482337	18.60444161	17.51144590	0.01311796	-0.03484610	-0.01147860
H	20.01400974	12.42710781	17.22354794	-0.01475783	0.00590870	-0.00270761
O	19.13753855	12.86208488	17.14785134	-0.02148768	0.01059905	-0.00079285
H	18.55339614	12.16970367	16.71517915	-0.02177445	0.01719612	0.00471072
H	16.90794465	10.51446057	15.45597688	-0.00720285	0.02076439	0.00264522
O	17.37655078	11.33904433	15.71716382	-0.01023983	0.01155932	-0.00461712
H	17.08210040	11.99286304	14.99995682	-0.01014388	0.00820295	0.00562727
H	18.50086007	15.99887198	13.94472946	-0.02863442	0.00162849	0.01472406
O	18.19685487	16.66054047	13.26931951	-0.01805774	-0.00474909	0.02147247
H	17.65551016	17.34997394	13.76798366	-0.01253895	-0.02016742	0.01968163
H	13.55674026	15.56223464	19.64930886	-0.00523788	0.00301801	-0.00604191
O	13.06854321	15.89627027	18.82818338	0.01779518	0.00542793	-0.01836996
H	12.32217453	15.22189578	18.57364532	0.00362702	0.00523585	-0.00503779

*CO_2

114

Lattice="29.5223634 0.0 0.0 0.0 29.977376800000002 0.0 0.0 0.0 31.099404999999997" Properties=species:S:1:pos:R:3:forces:R:3

Cu	16.43719877	14.76747143	15.96264431	-0.03663322	0.02152832	0.00914855
Cu	14.15346457	15.04154467	16.61874066	0.00018915	-0.01958326	-0.00594922
Cu	14.43186177	14.83346648	14.31338186	-0.00959798	0.03083063	0.01113079
Cu	12.79046647	13.34788863	15.27869080	0.06105049	0.03579932	-0.01189136
Cu	14.97382873	12.85689362	15.90670110	0.00150300	0.07481386	0.01970945
Cu	12.39129193	15.77153157	15.13769675	0.05814580	-0.00029471	-0.03578358
H	11.23075829	12.83609134	18.08729972	0.02049441	0.01498164	-0.02434218
O	10.77273602	13.57689626	17.56013402	0.01128284	0.00588495	-0.01325449
H	10.78066917	13.23637685	16.62904901	0.00455393	0.01456636	0.01023825
H	18.44370489	13.49801030	15.33490509	-0.00578923	-0.00123606	-0.00610470
O	17.96368318	14.18541982	14.74899505	-0.02630163	0.00415386	0.00962586
H	17.47082434	13.60265771	14.08204155	-0.01479040	-0.00344053	-0.00166681
H	13.10537217	13.92190136	20.83176472	0.00324417	0.00068908	-0.01950866
O	13.74514891	14.50486746	20.37044932	0.00383130	0.00421427	-0.02112846
H	14.40005485	13.86256189	19.97354037	-0.00083357	0.01232960	-0.02853120
H	14.37585452	11.21867912	14.56095443	0.00061677	0.01217817	-0.01063492
O	14.24397786	10.33913401	14.07935912	0.00529610	0.02099414	0.00198311
H	13.71988738	10.67287397	13.28601947	0.01182015	0.02037562	0.02482821
H	13.58652889	17.76256638	11.82954182	-0.00219663	-0.01640799	0.02192026
O	12.70904529	17.57933807	12.33797220	0.00908336	-0.00953028	0.02046555
H	12.91002615	18.09818526	13.19444034	0.00277728	-0.02889761	0.02166880
H	10.77268237	17.94462081	12.82010560	0.00547410	-0.01569206	0.01640399
O	9.95712595	18.06402026	13.36371154	0.02386079	-0.01826175	0.00793646
H	10.03788671	17.36392763	14.07362857	0.02641153	-0.00800824	0.00435808

H	15.15145260	11.66091806	18.54390780	-0.00364150	0.02852494	-0.02099798
O	14.88629255	12.26382248	19.33640181	-0.00039581	0.01749966	-0.02519366
H	15.38386018	11.85376545	20.07691354	-0.00197781	0.00674779	-0.01739753
H	14.20288746	19.68535530	13.85433896	0.00056009	-0.01780776	0.00721083
O	13.35745203	19.31907240	14.19145440	0.00332268	-0.02674385	0.00552845
H	13.60921064	18.91094826	15.10577554	0.00134296	-0.02690354	-0.00034873
H	13.34432321	17.62224527	19.69493310	0.01217503	-0.01382334	-0.02088381
O	11.95359830	17.42214465	19.34323328	0.01062637	-0.01066023	-0.02261529
H	12.09905491	16.56225124	18.83761114	0.01689957	-0.00378055	-0.02561255
H	14.78718799	16.78124306	19.72835048	-0.00816611	-0.00777758	-0.02119339
O	14.37948013	17.66362321	19.98874454	-0.00094166	-0.01781694	-0.02141050
H	15.19562839	14.70016691	11.72233449	-0.00870342	0.00614718	0.01643725
O	14.55616879	14.16511389	12.33728163	0.00290797	0.01311271	0.02618261
H	13.60090712	14.53135367	12.12166361	-0.00146260	0.00005191	0.00860983
H	13.10619215	11.93991371	19.09750733	0.00240989	0.01599168	-0.02552159
O	12.14827444	11.71074417	18.91915770	0.02450790	0.01696375	-0.01513948
H	12.22285261	10.99314430	18.24343558	0.01304134	0.01710888	-0.00588426
H	10.76018857	14.63858708	12.81763733	0.02333469	0.00227798	0.03122771
O	9.97250218	14.39752970	13.39662405	0.01523829	0.00604503	-0.00164345
H	10.04093254	15.13320411	14.11233824	0.01117907	-0.01024602	-0.00431320
H	11.92220165	15.20874614	11.02197426	0.00919809	0.00161914	0.01931317
O	12.19863983	15.16377699	11.96407732	-0.00161033	-0.00488454	0.01041671
H	12.42006515	16.19785433	12.22182290	0.01003763	-0.01184159	0.03384975
H	16.95401949	15.79116527	11.71510075	-0.01336739	-0.00847715	0.01986427
O	16.19871324	15.57957185	10.97801980	-0.00930667	-0.00542703	0.01897492
H	16.65997858	15.28104973	10.16712382	-0.00312556	0.00058996	0.01368267
H	16.80661877	18.28195554	14.76518496	-0.02114928	-0.02435196	0.00957625
O	16.16027936	17.79307138	14.08090268	-0.02592076	-0.01844563	0.01284534
H	15.64213740	17.17793252	14.66485894	-0.01122021	-0.01049423	0.01197434
H	16.85113094	13.79013860	18.79890058	-0.01809478	-0.00436778	-0.03153647
O	17.75603852	13.39883764	18.90835153	-0.02279054	-0.00118204	-0.02161764
H	17.92564708	12.95353070	18.04195034	-0.03233064	0.01364810	-0.01680962
H	17.85792739	16.37732877	18.89387219	-0.00370304	-0.00052184	-0.01120182
O	18.75439386	15.98902540	18.75155953	-0.02315367	-0.00381540	-0.01201716
H	18.52918704	15.00909770	18.80383301	-0.01316097	0.00658277	-0.01747396
H	15.48807458	17.22634209	11.06011323	-0.00844446	-0.00519494	0.01910180
O	15.11194546	18.07517153	11.42209268	0.00232536	-0.01745010	0.02234555
H	15.50010598	18.08982972	12.34115450	-0.00828208	-0.01538239	0.00910696
H	14.44416455	10.31824591	17.16371786	0.00565227	0.02298128	-0.00873019
O	15.36560634	10.42043118	17.55788675	-0.00149205	0.01257071	-0.01388467
H	15.90377772	10.71038573	16.77481330	-0.00096267	0.00119211	0.00867658
H	14.81427072	19.03330707	17.64350610	0.00026705	-0.01891925	-0.00617005
O	15.52953307	19.09856736	18.35186782	-0.00280437	-0.01492267	-0.01423739
H	16.14648909	18.39551868	17.95896501	-0.00344701	-0.01473842	-0.01213005
H	10.42170009	13.35685989	14.08106657	0.01165810	0.01339862	0.00278457

O	11.00095798	12.61690886	14.71787244	0.02749965	0.00727018	0.01155185
H	10.62359481	11.71400802	14.77801593	0.01587546	0.00832204	0.00306641
H	10.87433275	17.57401612	16.39017413	0.01142926	0.00301439	-0.00125154
O	10.49907800	16.20930757	15.31212425	0.03265959	-0.00429035	0.00520299
H	10.09939253	15.70095544	16.05558884	0.00288516	-0.01090302	0.00957121
H	10.95801842	19.15002775	16.22799937	0.01100591	-0.02069303	-0.00360053
O	11.18825740	18.38356184	16.90421558	0.00896865	-0.01530185	-0.00982600
H	11.58713412	18.00544644	18.61882158	0.01398891	-0.01271641	-0.00780463
H	19.09924625	16.48097062	17.26679200	-0.02081087	-0.00801945	-0.01274201
O	19.32039022	16.81229174	16.31683810	-0.02421157	-0.01352228	-0.00957653
H	18.87339626	16.14327138	15.75493046	-0.02246368	-0.00200730	0.00270439
H	18.41541796	19.46075002	15.25365754	-0.00639588	-0.01323190	0.00244004
O	17.78469557	18.87328567	15.72060190	-0.01469365	-0.01323086	0.00678950
H	18.37246766	18.07719932	16.02147148	-0.01807383	-0.01219228	0.00098702
H	13.06624207	11.43195967	16.25028835	-0.01260676	-0.00084564	0.00722971
O	12.81572482	10.46020498	16.42520027	-0.00114925	0.01303881	-0.00322089
H	13.11473338	10.07360157	15.54888349	0.00164261	0.02823219	0.00375682
H	16.90397185	12.23273911	12.28159701	-0.01519989	0.01363822	0.01567248
O	16.56393231	12.55652930	13.14444609	-0.01047116	0.00732414	0.00238347
H	15.68783260	13.00978359	12.92905184	-0.00513372	-0.00587959	0.02505318
H	12.20464365	12.11138800	12.97012122	0.01662839	0.01745308	-0.00019652
O	12.77078869	11.76389100	12.23458827	0.00359016	0.02627346	0.01952899
H	13.42604455	12.49502845	12.12155532	-0.00076344	0.01344307	0.02970418
H	11.69710184	20.10690114	14.66501322	-0.00130706	-0.01517539	0.00044693
O	10.76137159	20.06824271	14.99301339	0.01464786	-0.01587246	-0.00377329
H	10.35760880	19.40179753	14.34311251	0.01426246	-0.00761275	0.00637263
H	13.96374382	17.34683770	16.38759679	0.00938658	0.01246268	0.00611203
O	13.71995648	18.30666104	16.52939565	0.00879932	-0.01723575	0.00477984
H	12.68519181	18.28660373	16.72734623	0.01383681	-0.03202537	-0.01051268
H	19.63980104	11.89518092	16.48717992	-0.01504933	0.00609902	-0.00087157
O	18.73485881	12.24890765	16.35564880	-0.01818041	0.00545788	0.00050976
H	18.19160105	11.49244366	15.97417869	-0.02138992	0.01621608	0.00398170
H	16.46723584	9.86964462	14.90027688	-0.00643075	0.02021061	0.00314939
O	16.94836626	10.69577616	15.13579902	-0.01073122	0.01393954	-0.00584673
H	16.62135036	11.33377142	14.43511883	-0.00591053	0.01081981	0.00791337
H	18.08330486	15.52549380	13.41149792	-0.02680973	0.00165162	0.01611559
O	17.80819144	16.23521461	12.78008455	-0.01475480	-0.00752802	0.02218070
H	17.19040059	16.84235446	13.34532769	-0.01196074	-0.02080718	0.01725843
H	13.11599406	14.84710423	19.00573803	0.00209111	-0.00282500	-0.01388242
O	12.74553684	15.19313785	18.10556041	0.02051256	0.00504497	-0.01097442
H	11.95996096	14.57691541	17.83935610	0.00679287	-0.00085719	-0.01785842
C	15.82658450	15.89993104	17.51516104	-0.00430394	0.00097128	-0.00839435
O	15.76683557	15.59139339	18.75722658	-0.02285360	0.00158220	-0.00309206
O	16.34348776	16.98126910	17.03827482	0.00125913	-0.00132625	-0.01095038
H	10.49907800	16.22930757	14.66212425	-0.02680973	0.00165162	0.01611559

*CO;*OH

115

Lattice="29.682674999999996 0.0 0.0 0.0 30.2372565 0.0 0.0 0.0 30.664640900000002"

Properties=species:S:1:pos:R:3:forces:R:3

Cu	16.33008133	15.05216015	16.14942800	-0.01423899	-0.01321101	-0.03222804
Cu	13.84543887	14.86223228	16.71542431	-0.01439771	-0.02508552	0.02258478
Cu	14.41884281	15.02994420	14.38360774	0.01695215	0.03806350	-0.00675015
Cu	12.80053592	13.36135956	15.14448721	0.06513171	0.04406965	-0.00336783
Cu	15.08511647	13.02224893	15.78559658	0.01467272	0.05504530	-0.00209980
Cu	12.28634497	15.79219936	15.17583648	0.07240710	-0.00970793	-0.03216354
H	11.17027283	12.82207576	17.89657916	0.02256355	0.01727391	-0.02254812
O	10.63594921	13.51423457	17.38255355	0.01227790	0.00222050	-0.01302757
H	11.19603079	13.60199561	16.55897441	0.01896992	0.01198581	0.00008805
H	18.50971026	13.78298956	15.19918222	-0.01820585	0.00858424	-0.00025829
O	17.98974276	14.47499665	14.68314245	-0.02604343	-0.00061752	0.01021175
H	17.44843485	13.89542600	14.06546690	-0.02054319	-0.00006852	0.00648457
H	12.86054512	13.62725509	20.82308897	0.00432696	-0.00053485	-0.01850476
O	13.47352336	14.26381527	20.39685823	0.00649479	0.00381088	-0.02496289
H	14.13396135	13.67298070	19.93402324	-0.00088784	0.01227607	-0.02320725
H	14.48228824	11.38511122	14.41617906	0.00164089	0.00801455	-0.01152420
O	14.27132715	10.52149786	13.94696836	0.00523026	0.01841224	0.00238774
H	13.75042265	10.88737319	13.15298120	0.00987096	0.02225338	0.02501350
H	13.55664964	17.87976393	11.70296488	-0.00338866	-0.01420854	0.01944590
O	12.64094520	17.72813215	12.16660263	0.00574477	-0.01128583	0.02513955
H	12.84858220	18.21158968	13.03520218	0.00107968	-0.02631828	0.02019830
H	10.77880173	18.18195375	12.64010248	0.00575567	-0.01511585	0.01551277
O	9.99856710	18.33286262	13.22882937	0.02226460	-0.01896902	0.00857230
H	10.04774364	17.58338243	13.88858173	0.02446429	-0.00835861	0.00489366
H	15.25847265	11.69114611	18.36609200	-0.00585242	0.02940559	-0.02151803
O	15.05679885	12.32615346	19.16976740	-0.00949745	0.01473569	-0.02068375
H	15.33681443	11.77502020	19.93315565	-0.00246702	0.00841148	-0.01767548
H	14.28009091	19.76221686	13.66933006	0.00079940	-0.01658133	0.00855316
O	13.42573723	19.46172450	14.04950924	0.00292943	-0.02514571	0.00571845
H	13.70143798	18.97768967	14.90783271	0.00160527	-0.02522649	0.00007797
O	12.04255115	17.72460358	19.16076484	0.01037029	-0.00974458	-0.01915721
H	12.00384787	16.71931400	18.84184824	0.01810897	-0.00518170	-0.02484366
H	14.78208471	17.00108195	19.80838170	-0.01002182	-0.00269860	-0.01892186
O	14.42842276	17.91918181	19.76200920	0.00147335	-0.02121626	-0.02355504
H	15.04860692	18.39804331	19.07078279	-0.00685825	-0.01920955	-0.01279507
H	15.24867728	14.89165357	11.74790495	-0.01088939	0.00520452	0.02223978
O	14.58826085	14.37778446	12.36271087	0.00217035	0.01309397	0.02490970
H	13.64972936	14.70316388	12.06671353	-0.00252028	-0.00225020	0.00744970
H	13.16273298	12.03165539	18.84591366	-0.00083185	0.01948636	-0.03114583
O	12.21472050	11.74104683	18.75652381	0.02502745	0.01966932	-0.01317936

H	12.28563681	11.01392740	18.08889916	0.01374409	0.01899243	-0.00517993
H	10.76833548	14.84979987	12.59808527	0.02023910	0.00059741	0.03129386
O	10.02899682	14.55836838	13.21836970	0.01388835	0.00510120	-0.00168980
H	10.08374771	15.28103405	13.95526430	0.00684095	-0.00997754	-0.00815715
H	12.03540229	15.31295213	10.79921765	0.00802828	0.00100903	0.01714646
O	12.22989939	15.29928661	11.76130490	0.00328886	-0.00476180	0.01847722
H	12.39770866	16.33910327	12.01925144	0.00831589	-0.00935687	0.03127764
H	17.06428837	15.94157087	11.65832094	-0.01066758	-0.00746120	0.01973279
O	16.26932420	15.69628298	10.94174745	-0.00818068	-0.00671640	0.01763523
H	16.69754415	15.35339828	10.13057379	-0.00305932	-0.00002884	0.01413647
H	17.16894800	18.68185063	14.29403036	-0.01818847	-0.01912737	0.00605120
O	16.42095554	18.07859346	13.90230424	-0.02540507	-0.01778723	0.01019330
H	16.28601025	17.55354427	14.78057241	-0.01578383	-0.02454208	0.00590919
H	17.10229606	13.28297347	18.74694625	-0.00915650	0.00713403	-0.02999715
O	18.04459482	13.49162314	18.54494158	-0.02001038	-0.00321286	-0.01747564
H	18.23727873	12.98489133	17.71320602	-0.03258789	0.01466684	-0.01248747
H	17.77186977	16.53114731	17.81852664	-0.00450844	0.00087715	0.00251285
O	18.62277880	16.17720545	18.18950107	-0.02078941	-0.00169367	-0.00801527
H	18.41346910	15.20910525	18.34886330	-0.02088828	0.00532392	-0.02206966
H	15.48534239	17.29496167	11.00840115	-0.00820906	-0.00436322	0.01856841
O	15.07773950	18.12596500	11.38313306	0.00326301	-0.01716942	0.02209636
H	15.50421180	18.16832976	12.28727942	-0.00681166	-0.01770576	0.01073400
H	14.55775557	10.41089051	16.97361565	0.00350846	0.02629212	-0.00945954
O	15.48459466	10.53122802	17.37241165	0.00102342	0.01389657	-0.01241564
H	16.03681747	10.79518666	16.58936838	-0.00164562	0.00322079	0.00882159
H	15.08052500	19.05494881	17.28258138	-0.00292972	-0.01756318	-0.00811916
O	15.79418802	19.17728622	17.96842086	-0.00239983	-0.01864522	-0.01639604
H	16.53923682	18.67604669	17.54273977	-0.00178000	-0.01569896	-0.00955276
H	10.58118379	13.51853366	13.74366515	0.01051408	0.01699383	0.00290392
O	11.23072869	12.64049695	14.12234955	0.02836536	0.00925775	0.01771403
H	10.68051005	11.99645665	14.61946275	0.00176432	0.00152128	0.00349714
H	10.89825517	17.73634858	16.23337839	0.00770860	0.00830759	0.00081977
O	10.42390077	16.34866983	15.11506803	0.03302864	-0.00274388	0.00596009
H	9.88731856	15.92448608	15.82333067	-0.00101941	-0.00718365	0.00728386
H	11.05964442	19.32136382	16.06793935	0.00951771	-0.02053412	-0.00422834
O	11.26664554	18.52208588	16.74037507	0.00725392	-0.01529792	-0.00933379
H	11.74119890	18.21544708	18.32335401	0.01457549	-0.01469244	-0.00781477
H	19.32384929	16.60720607	16.77636667	-0.02073099	-0.00756115	-0.01159624
O	19.56682027	16.90583919	15.82883586	-0.02717384	-0.01280229	-0.01029020
H	19.01255318	16.29059161	15.30041171	-0.02512666	0.00203150	0.00940666
H	19.02614857	19.62198162	14.72600931	-0.00572254	-0.01240177	0.00211120
O	18.32054788	19.18506053	15.24678120	-0.01486314	-0.01725201	0.00922602
H	18.76744834	18.30615491	15.54555709	-0.01758420	-0.01004503	0.00198889
H	13.15961695	11.53020622	16.21125350	-0.01191407	0.00120631	0.00987435
O	13.01781352	10.52798850	16.34255936	-0.00207288	0.01323889	-0.00345220

H	13.25636196	10.22830357	15.40908404	0.00166346	0.03051399	0.00324246
H	17.01057307	12.58340492	12.08767168	-0.01345056	0.01420637	0.01553412
O	16.63454540	12.74399558	12.98059099	-0.01142740	0.01061579	0.00552924
H	15.74311642	13.18236726	12.80964357	-0.01282588	0.00314840	0.03113593
H	12.17742648	12.12251785	12.74925722	0.01426233	0.01936337	0.00898535
O	12.87716602	11.87005360	12.07766929	0.00629471	0.02500893	0.01385817
H	13.46033854	12.66537582	12.05920507	-0.00252261	0.01229765	0.03038199
H	11.78675598	20.30512310	14.56328900	-0.00183154	-0.01470804	0.00063067
O	10.85673174	20.25847680	14.91010706	0.01398868	-0.01596781	-0.00430927
H	10.43973435	19.62004388	14.23439194	0.01347390	-0.00750088	0.00646338
H	13.84415870	17.24386856	15.97085930	0.01787521	0.02027206	0.00377743
O	13.81162641	18.20680225	16.28807677	0.00098691	-0.01115965	0.00434064
H	12.80367714	18.31778783	16.51704688	0.01320734	-0.03012942	-0.00819614
H	19.75800957	11.97092786	16.18616611	-0.01351861	0.00530512	-0.00212878
O	18.84864213	12.33174000	16.12181673	-0.02182530	0.00776198	-0.00056803
H	18.29252686	11.59445235	15.72879474	-0.02038514	0.01425544	0.00430132
H	16.48960671	9.99630211	14.62299930	-0.00439436	0.01860735	0.00463159
O	17.02357104	10.77793925	14.89446500	-0.01136519	0.01515446	-0.00437571
H	16.75906880	11.45654024	14.20372966	-0.01166302	0.01444519	0.01290662
H	18.14599880	15.68197113	13.30808687	-0.02529911	0.00180284	0.01204116
O	17.94162997	16.38763383	12.64028670	-0.01284357	-0.00531551	0.02212768
H	17.35744546	17.06814932	13.17537411	-0.01099262	-0.01843553	0.01780260
H	12.65156899	14.86758277	19.16510372	0.00588852	0.00122418	-0.02107475
O	12.19783607	15.33435052	18.37623524	0.02114810	0.00018540	-0.01858923
H	11.44728447	14.70209087	18.06539338	0.02001060	0.00358089	-0.01971870
C	15.23528599	15.16129237	17.92032545	-0.01928443	0.00321900	0.00219723
O	15.81073585	15.35235380	18.94875150	-0.00560044	-0.00075212	-0.00975443
O	16.61080310	17.01165442	16.31006379	-0.00324569	-0.00808385	-0.01421440
H	15.72089721	17.21228637	16.67686232	-0.01113509	-0.00969031	-0.00705003
H	16.61080310	17.01165442	15.71006379	0.00125862	-0.00075210	-0.01395216

*COOH

115

Lattice="29.682674999999996 0.0 0.0 0.0 30.2372565 0.0 0.0 0.0 30.664640900000002" Properties=species:S:1:pos:R:3:forces:R:3

Cu	16.74106906	15.03925816	15.34748102	-0.05255854	-0.03140767	0.01785213
Cu	14.52591504	15.34686404	16.42396230	-0.02336440	-0.02140354	0.01749097
Cu	14.64659722	14.89989722	14.08335615	-0.02151007	0.01568373	0.01641437
Cu	13.06988131	13.53412278	15.29311868	0.04576209	0.02539582	-0.02759077
Cu	15.34602538	13.14259033	15.75548144	-0.00261620	0.04579661	0.05113765
Cu	12.66166000	15.92890047	15.00007535	0.05150035	-0.00190044	-0.04584531
H	11.30548656	12.84857959	17.87173824	0.01899374	0.01411831	-0.02109518
O	10.79791706	13.54736730	17.34053496	0.01240711	0.00456298	-0.01194194
H	10.97956589	13.29303006	16.40131350	0.00093448	0.01392606	0.01257870
H	18.87402762	13.49652646	14.77542425	-0.01728469	0.00510271	-0.00137672

O	18.83825963	14.03033382	13.92704260	-0.01877510	-0.00830439	0.00495148
H	18.21396684	13.49227434	13.37110531	-0.02152271	0.01053248	0.02068993
H	12.78155008	13.59735649	20.42476347	0.00382693	0.00336054	-0.01441880
O	13.42222510	14.31462635	20.22655910	0.00431442	0.00353778	-0.02172538
H	14.20973942	13.82703170	19.86581296	-0.00158239	0.01341009	-0.02283659
H	14.52445140	11.41115550	14.38934981	-0.00303923	0.00534822	-0.01351675
O	14.27225608	10.54953223	13.94403019	0.01083122	0.02239056	0.00642094
H	13.73074570	10.92259352	13.16495132	0.01157730	0.02329797	0.02347851
H	13.51046865	17.85294162	11.64305008	-0.00166590	-0.01557092	0.02247706
O	12.60201254	17.72240783	12.11950956	0.00941355	-0.00997810	0.02191022
H	12.81003688	18.20730694	12.98487679	0.00402402	-0.02834951	0.02239747
H	10.73504473	18.20437026	12.67587984	0.00671739	-0.01412536	0.01514706
O	9.99220231	18.31413383	13.31739435	0.02301941	-0.01843537	0.00677188
H	10.14492140	17.57475238	13.97530540	0.02400365	-0.00766673	0.00148387
H	15.28940435	11.68161447	18.36591956	-0.00528411	0.02461101	-0.02147465
O	15.05054472	12.33612839	19.14009507	-0.00932892	0.01302429	-0.02220249
H	15.39110816	11.85667634	19.92667259	-0.00207956	0.00640541	-0.01622768
H	14.18175164	19.79872962	13.55967947	-0.00079880	-0.01702486	0.00860339
O	13.36322551	19.47111272	13.98858792	0.00285298	-0.02838495	0.00630969
H	13.68438206	19.08338270	14.88108242	0.00029944	-0.02669450	-0.00092281
O	11.99256423	17.49883687	19.14650129	0.01460751	-0.00812803	-0.02131083
H	12.16395056	16.59833050	18.63419868	0.01998740	-0.00318333	-0.02461853
H	14.74658020	17.12283918	19.85049995	-0.00807685	-0.00435867	-0.01824168
O	14.22309622	17.95693597	20.02680191	0.00066656	-0.01594574	-0.02117270
H	14.66760700	18.67517205	19.40917408	-0.00520935	-0.01736613	-0.01349288
H	15.14938767	14.78918089	11.55097384	-0.00646733	0.00212010	0.00891245
O	14.43867360	14.30355565	12.10249393	0.00325243	0.01149135	0.02845366
H	13.53911025	14.72937890	11.85836272	-0.00137796	0.00216072	0.00930728
H	13.20813697	11.99484769	18.85926278	-0.00022489	0.01120998	-0.02877912
O	12.24434181	11.74837595	18.76236947	0.02419251	0.01695074	-0.01480239
H	12.28704405	11.00308128	18.11400737	0.01166427	0.01591299	-0.00266141
H	10.74906926	14.76908890	12.65614823	0.02219752	0.00107267	0.02792575
O	10.08307007	14.50620730	13.37139756	0.01598899	0.00451124	0.00133607
H	10.23777271	15.27222759	14.05518515	0.01457755	-0.00800134	-0.00215528
H	11.75955597	15.36286197	10.77061802	0.00845163	0.00205664	0.01609026
O	12.04834693	15.31344140	11.70729829	0.00690007	-0.00352008	0.01947463
H	12.26224542	16.33142382	11.97102557	0.01142130	-0.00928467	0.03225145
H	17.04304175	15.90201246	11.59693671	-0.01270095	-0.00924880	0.02513700
O	16.28061780	15.69739378	10.89162809	-0.00774519	-0.00612921	0.02189909
H	16.74147420	15.40679449	10.07718983	-0.00293328	0.00071830	0.01478992
H	16.59595446	18.35835731	14.56867960	-0.02391213	-0.02722701	0.00537279
O	16.03264272	17.88849787	13.86216241	-0.02095869	-0.00858064	0.00976941
H	15.51698946	17.16924704	14.34479613	-0.01717939	0.01327885	0.01192400
H	16.86164606	13.51635944	18.64079212	-0.01175141	0.01600019	-0.03430605
O	17.84768076	13.50639178	18.55356696	-0.02494544	-0.00075241	-0.02174293

H	18.02224108	13.05982985	17.68007515	-0.03155438	0.01490693	-0.01653065
H	18.13393778	16.49066037	18.58910971	-0.00855012	-0.00845641	-0.01241310
O	18.86109644	15.89790538	18.28141012	-0.01783823	-0.00015608	-0.00919473
H	18.45357135	14.96325433	18.44440646	-0.01598491	0.00720327	-0.01948078
H	15.45044562	17.27214981	10.94050314	-0.00879621	-0.00609140	0.02101575
O	15.03160278	18.11166066	11.28397879	0.00237006	-0.01841850	0.02182537
H	15.41194793	18.14921357	12.20556350	-0.00457978	-0.01929604	0.01071929
H	14.57677585	10.41847762	16.97789077	0.00502580	0.02650006	-0.00997600
O	15.50770562	10.52870516	17.36487589	0.00079607	0.01250897	-0.01285947
H	16.04007008	10.82322187	16.57849305	-0.00460472	0.00231432	0.00682175
H	14.70567763	19.33942667	17.59506914	-0.00015915	-0.01860105	-0.00494164
O	15.24487378	19.67451388	18.37905017	-0.00386631	-0.02017277	-0.01614565
H	16.09647583	19.20908191	18.20687165	-0.00474962	-0.00927087	-0.00877787
H	10.67924763	13.54567367	13.92543396	0.02076223	0.01571426	0.00619153
O	11.39817453	12.73713727	14.41432189	0.03165217	0.00658669	0.00662672
H	10.93391707	11.91436258	14.68501000	0.01416453	0.00593483	0.00302962
H	11.04945940	17.73902606	16.18234026	0.01251829	0.00130244	-0.00227777
O	10.73505591	16.35914780	15.11618871	0.03285449	-0.00140088	0.00485878
H	10.31708265	15.90255583	15.88349524	0.00221522	-0.00910666	0.00963304
H	11.04815845	19.33602304	16.09989671	0.01116768	-0.01992117	-0.00367084
O	11.30734775	18.53717132	16.74290683	0.01021066	-0.01577097	-0.00883651
H	11.68943704	18.10021821	18.39909085	0.01628328	-0.01228065	-0.00703615
H	18.67489321	16.06228218	16.91866234	-0.01377949	-0.00574594	-0.00358952
O	18.42948545	16.24917666	15.85148618	-0.02627814	-0.01623632	-0.01428327
H	19.17831252	15.90240526	15.31673220	0.00236623	0.00821563	0.00903663
H	18.35900794	19.43050131	15.16110526	-0.00735386	-0.01497329	0.00189701
O	17.73549500	18.85482634	15.65143695	-0.01268102	-0.01474105	0.00589176
H	18.20268106	17.96012684	15.71416056	-0.01605644	-0.01463062	-0.00040004
H	13.20140110	11.55010176	16.21642494	0.00014194	0.00571432	0.00510499
O	13.03037810	10.55604881	16.32636662	0.00157553	0.01779165	-0.00565195
H	13.29456253	10.26893665	15.39126018	0.00318072	0.02842614	0.00380003
H	16.00323035	12.42763987	12.26869892	-0.01234212	0.01810417	0.02613790
O	16.67546032	12.55734121	12.97561169	-0.01397753	0.00897959	-0.00205133
H	16.19707802	13.18629538	13.62955899	0.00033972	-0.01884253	-0.02390969
H	12.27617515	12.23914311	12.88573382	0.02012831	0.02362867	0.01284143
O	12.85682050	11.91999448	12.13698143	0.00491598	0.02556342	0.01783885
H	13.47931406	12.68459031	12.02151618	0.00272155	0.01639775	0.03168196
H	11.74704630	20.33820212	14.57527840	-0.00141770	-0.01410648	0.00111114
O	10.82433537	20.29144656	14.93836246	0.01470499	-0.01483920	-0.00429567
H	10.40817684	19.63557895	14.28068418	0.01402004	-0.00673586	0.00602259
H	14.03691787	17.54322432	16.28104030	0.00761212	0.01436812	0.00917203
O	13.83876503	18.52825388	16.36371310	0.01098765	-0.01584190	0.00523339
H	12.80217881	18.52499960	16.55714301	0.01221491	-0.03102193	-0.01054214
H	19.65374065	11.88724033	16.37934055	-0.01479040	0.00707628	-0.00388646
O	18.81225398	12.33890863	16.15228554	-0.02242662	0.01358965	-0.00198916

H	18.23117940	11.61116697	15.76925527	-0.02189942	0.01895480	0.00108660
H	16.54627532	9.92173341	14.71869571	-0.00843902	0.02117665	0.00245621
O	17.05125708	10.74102324	14.91773788	-0.01053863	0.01258156	-0.00394531
H	16.83474863	11.33745838	14.12805142	-0.01084717	0.01850140	0.01488615
H	18.25503886	15.58523540	13.23457999	-0.01890728	0.00575357	-0.00124027
O	17.99584724	16.35969648	12.66013257	-0.01573277	-0.00020983	0.01631518
H	17.33666469	16.88427096	13.21258741	-0.01218742	-0.02227100	0.01463036
H	12.97371317	14.89392758	18.81806567	0.01422088	0.00223001	-0.03652801
O	12.62628026	15.33707923	17.96061474	0.01677085	0.00657731	-0.00622405
H	11.88156515	14.70583954	17.65472690	0.03447054	0.00809129	-0.02882531
C	15.57889982	16.05580229	17.88565888	-0.02010160	-0.00630221	-0.00852185
O	15.60500438	15.71006388	19.08040539	-0.00229741	-0.00497256	-0.02020472
O	16.40924443	17.18365611	17.63305338	-0.01377830	0.00175241	-0.01653229
H	16.33706958	17.33682410	16.65990989	-0.01294550	-0.00197829	0.00661755
H	10.73505591	16.95914780	15.11618871	-0.01845728	0.00575357	-0.01258631

*HCOO

115

Lattice="29.682674999999996 0.0 0.0 0.0 30.2372565 0.0 0.0 0.0 30.664640900000002" Properties=species:S:1:pos:R:3:forces:R:3:initial_charges:R:1

Cu	16.35367976	15.01082188	15.40260974	-0.04242164	-0.01055410	-0.01517190	0.20110000
Cu	14.09713143	15.20411632	16.33982961	-0.00858499	-0.01811867	-0.01655121	0.25230000
Cu	14.39677374	14.98834752	14.03744726	-0.01195579	0.03442933	0.02307223	0.11800000
Cu	12.83774922	13.43126601	15.07161422	0.04980792	0.04548138	-0.00243694	0.16350000
Cu	15.04214642	13.05225834	15.70681794	0.01642704	0.06953798	0.02891693	-0.12480000
Cu	12.34108106	15.89188888	14.89571484	0.07966945	0.01480416	-0.02395908	0.20160000
H	11.29213016	12.95355208	17.90138553	0.01979903	0.01517312	-0.02388824	0.62280000
O	10.85678302	13.71036186	17.38567775	0.01201633	0.00492432	-0.01178642	-1.21260000
H	10.88008124	13.39119219	16.44591232	0.00583500	0.01636392	0.00976162	0.57610000
H	18.55338354	13.65934785	15.18718844	-0.00922414	0.00245032	-0.00070050	0.62830000
O	18.21561131	14.34371483	14.51922371	-0.02893287	-0.00274372	0.00912097	-1.23440000
H	17.73671162	13.77014706	13.84656696	-0.00793897	-0.00739431	-0.00081956	0.62330000
H	12.97569184	13.90472326	20.57599269	0.00323756	0.00024820	-0.01781082	0.58380000
O	13.66675332	14.49530779	20.20575269	0.00589306	0.00399721	-0.02130618	-1.16310000
H	14.36139482	13.86109144	19.87485166	0.00151009	0.01535760	-0.02598844	0.62940000
H	14.40096625	11.39046864	14.42691763	0.00720415	0.00494431	-0.01324329	0.49410000
O	14.27059666	10.51107683	13.94506958	0.00431550	0.01954038	0.00039009	-1.22060000
H	13.74360350	10.84979537	13.15189842	0.01132544	0.02174970	0.02469939	0.62090000
H	13.54753899	17.86610598	11.65483038	-0.00282383	-0.01708150	0.02242616	0.62710000
O	12.64388469	17.72057993	12.13768988	0.00781763	-0.00881212	0.02019906	-1.26380000
H	12.86876049	18.22293746	12.99756696	0.00270583	-0.02949843	0.02171959	0.62200000
H	10.79079980	18.14646673	12.60770755	0.00651608	-0.01581535	0.01606695	0.59800000
O	9.98562455	18.29090888	13.16254831	0.02300320	-0.01912347	0.00887703	-1.20280000
H	10.03697632	17.56918910	13.85066108	0.02569315	-0.00908219	0.00433213	0.61010000
H	15.26336275	11.71316519	18.36239028	-0.00478910	0.02511250	-0.02172150	0.62770000

O	15.05420167	12.34680133	19.16395821	-0.00780196	0.01503286	-0.02370933	-1.22470000
H	15.39173869	11.82553576	19.92507503	-0.00260036	0.00550359	-0.01542717	0.58540000
H	14.26803467	19.78507612	13.65090682	-0.00023090	-0.01686976	0.00814017	0.58020000
O	13.40588968	19.45695260	13.98426524	0.00311809	-0.02682484	0.00532692	-1.20360000
H	13.62874896	19.06012587	14.91090347	0.00139140	-0.02687949	-0.00120938	0.62500000
H	13.43775790	17.81297962	19.57039850	0.01236571	-0.01248194	-0.01964687	0.63790000
O	12.01917744	17.53606327	19.17242489	0.01089297	-0.00983223	-0.02361008	-1.21260000
H	12.20630416	16.69602778	18.65352177	0.01578514	-0.00321991	-0.02497012	0.61100000
H	14.94950662	17.06448384	19.56442257	-0.01047421	-0.00760458	-0.01936666	0.63840000
O	14.44791063	17.92533300	19.86460841	-0.00420971	-0.01283701	-0.01833306	-1.23280000
H	15.16576045	14.82274376	11.54553225	-0.00659564	0.00499036	0.00906856	0.64150000
O	14.46536520	14.29857214	12.09268073	0.00196011	0.01201007	0.02437107	-1.27480000
H	13.53548071	14.69445980	11.83316526	-0.00137048	0.00055431	0.00814252	0.63910000
H	13.17454074	12.02790210	18.89145282	-0.00060609	0.01405806	-0.02623325	0.59520000
O	12.21926936	11.77444384	18.76123695	0.02506747	0.01795800	-0.01473245	-1.19970000
H	12.28419379	11.05514770	18.08521533	0.01274964	0.01633758	-0.00527823	0.60450000
H	10.75506875	14.77431392	12.62594353	0.02423101	0.00188998	0.03112163	0.62100000
O	9.99246726	14.52486209	13.23464686	0.01411691	0.00346656	-0.00365834	-1.22260000
H	10.05516921	15.27449839	13.95076843	0.00491717	-0.01126520	-0.00711853	0.62270000
H	11.86135776	15.39202059	10.76385285	0.00899638	0.00203292	0.01914860	0.58840000
O	12.14884096	15.31483733	11.70020769	-0.00151718	-0.00407693	0.01081622	-1.21870000
H	12.36188616	16.34008619	11.99331018	0.01091072	-0.01186396	0.03341835	0.63690000
H	17.03700653	15.92709592	11.60736792	-0.01398088	-0.00961004	0.02323365	0.63160000
O	16.26554097	15.69761798	10.89482693	-0.00877710	-0.00660859	0.02119151	-1.22210000
H	16.71579455	15.42955825	10.06724616	-0.00314950	0.00000684	0.01446137	0.59340000
H	16.82429558	18.43012058	14.59223231	-0.02106395	-0.02469094	0.00878884	0.63150000
O	16.21379498	17.92457646	13.89665782	-0.02487678	-0.01235453	0.00963927	-1.22480000
H	15.69773408	17.26305234	14.43840038	-0.01747849	0.01470786	0.00720567	0.54740000
H	16.90038615	13.49689230	18.87965270	-0.00868086	0.01150392	-0.02462173	0.57850000
O	17.88131108	13.50701868	18.76121854	-0.02130263	-0.00112552	-0.01932404	-1.18420000
H	18.02788688	13.03572293	17.90127596	-0.02794758	0.01067478	-0.01385688	0.60450000
H	18.30868692	16.58043841	19.11793216	-0.01159394	-0.00244563	-0.00833822	0.58680000
O	18.98933207	16.03801633	18.66327388	-0.01635984	-0.00581676	-0.01009171	-1.18890000
H	18.59123274	15.11366268	18.71906257	-0.00487533	0.00513338	-0.01103038	0.61770000
H	15.48825713	17.30405850	10.96199868	-0.00951314	-0.00578927	0.02075638	0.61500000
O	15.06878881	18.13624980	11.31766788	0.00237857	-0.01840998	0.02215859	-1.21330000
H	15.47235010	18.18526003	12.23053409	-0.00704057	-0.01797065	0.00900781	0.60290000
H	14.52475033	10.44124980	16.98334411	0.00495069	0.02540630	-0.01008323	0.61410000
O	15.45715807	10.54069435	17.36735877	0.00084251	0.01224225	-0.01305119	-1.22160000
H	15.98547611	10.84187247	16.58217322	-0.00367131	0.00188771	0.00935691	0.58740000
H	14.73852140	19.30183969	17.46627018	0.00015911	-0.01765301	-0.00472670	0.61130000
O	15.47667129	19.39028010	18.14394763	-0.00265505	-0.01425198	-0.01454892	-1.19490000
H	16.06006892	18.65478380	17.77057875	-0.00463107	-0.01381518	-0.01204082	0.61730000
H	10.44863163	13.51760766	13.88200380	0.01390895	0.01570224	0.00318593	0.63120000
O	11.03975059	12.74020304	14.51039754	0.02728896	0.00623884	0.00964807	-1.21640000

H	10.62845932	11.85403865	14.59816024	0.01579741	0.00796681	0.00376370	0.59060000
H	10.86778008	17.72385353	16.18537085	0.01329973	0.00319464	-0.00090284	0.61090000
O	10.44950056	16.33845181	15.08518811	0.03240173	-0.00274033	0.00504948	-1.18510000
H	10.05489468	15.85962776	15.85035522	0.00236206	-0.01004227	0.00914336	0.57280000
H	10.97977352	19.30169066	16.03849381	0.01108684	-0.02076119	-0.00398506	0.63010000
O	11.18350277	18.52062012	16.70944378	0.00835335	-0.01615868	-0.01083894	-1.25330000
H	11.63198639	18.11952874	18.46375845	0.01384720	-0.01224091	-0.00768742	0.61490000
H	19.12379582	16.60335502	17.08988757	-0.02056887	-0.00778123	-0.01461727	0.63170000
O	19.25885811	16.91576323	16.13048241	-0.02674319	-0.01624699	-0.00763775	-1.20390000
H	18.78686960	16.22798787	15.61171017	-0.01422678	0.00471428	0.00620485	0.57000000
H	18.48844164	19.56670065	15.01718102	-0.00591676	-0.01305020	0.00165600	0.57060000
O	17.83181435	19.05732028	15.53702526	-0.01636166	-0.01600802	0.00795620	-1.18390000
H	18.36785972	18.23444257	15.84936420	-0.01951395	-0.01263171	0.00083007	0.63570000
H	13.11142174	11.52366415	16.14027482	-0.01214744	-0.00291560	0.00923702	0.48140000
O	12.94447695	10.53714590	16.33029151	-0.00104569	0.01424843	-0.00411962	-1.18550000
H	13.19654386	10.17971068	15.42558649	0.00138054	0.02783035	0.00311203	0.61220000
H	16.84210167	12.36662498	12.01869170	-0.01546201	0.01523711	0.01780099	0.59600000
O	16.67052806	12.71560983	12.92069722	-0.01418896	0.00928214	0.00209457	-1.20730000
H	15.78015669	13.16493395	12.85650579	-0.00192092	-0.00705855	0.02670733	0.62810000
H	12.22194039	12.23866711	12.83004754	0.01489258	0.01494350	-0.00697281	0.58560000
O	12.82869838	11.91737168	12.11385165	0.00270274	0.02467825	0.01591729	-1.18160000
H	13.46539112	12.67191744	12.02642849	0.00148547	0.01744638	0.03360024	0.59020000
H	11.75409240	20.29090204	14.49693018	-0.00200368	-0.01447506	0.00036995	0.60360000
O	10.82032162	20.25602349	14.83238010	0.01448626	-0.01596695	-0.00430415	-1.21260000
H	10.40884568	19.60936239	14.16676167	0.01413690	-0.00761410	0.00642896	0.61880000
H	13.99439890	17.56056181	16.25289179	0.00716061	0.00221479	0.00088166	0.55050000
O	13.69291021	18.50587787	16.35025039	0.00899480	-0.02034144	0.00419879	-1.20990000
H	12.65653455	18.44548764	16.53945104	0.01295913	-0.03075639	-0.01097599	0.61700000
H	19.60758791	11.97835707	16.35768232	-0.01391554	0.00523630	-0.00186866	0.60060000
O	18.70559337	12.35055897	16.25406912	-0.02348357	0.01036099	0.00080450	-1.20370000
H	18.16363730	11.61584509	15.83143689	-0.02027030	0.01633927	0.00319490	0.63630000
H	16.43580966	10.01166552	14.67465319	-0.00333288	0.01922237	0.00447841	0.60160000
O	17.02412795	10.77148196	14.89115089	-0.01061228	0.01495691	-0.00669935	-1.21240000
H	16.73843854	11.45022404	14.21154151	-0.01117164	0.01565705	0.01277154	0.62010000
H	18.26349657	15.73910401	13.23539525	-0.02644799	0.00269917	0.01459667	0.60810000
O	17.94920508	16.43337239	12.60513176	-0.01381668	-0.00558025	0.02053093	-1.21490000
H	10.44950056	16.49845181	14.47518811	-0.01450129	0.00852697	-0.01502506	0.58011238
H	17.30898206	17.00781823	13.17153945	-0.01245152	-0.02159875	0.01688840	0.61230000
H	13.19591196	14.94268570	18.81851113	0.00559683	-0.00230838	-0.01591697	0.62860000
O	12.84804061	15.32159872	17.91903374	0.02202321	0.00569449	-0.01189673	-1.23200000
H	12.05025214	14.71716070	17.65367064	0.00275636	-0.00288069	-0.01601979	0.63140000
C	16.27724655	16.04973366	17.79344302	-0.01949879	0.00630150	-0.00264484	1.44530000
O	15.82974567	15.91620137	18.97544600	-0.00664400	-0.01294472	-0.00930224	-1.12490000
O	16.33566995	17.09056679	17.05212207	0.00325178	0.00931157	-0.01472558	-1.13440000
H	16.72704507	15.09284279	17.35862434	-0.00973100	-0.01808078	-0.02227112	0.01570000

*CO_2;*OH

115

Lattice="29.682674999999996 0.0 0.0 0.0 30.2372565 0.0 0.0 0.0 30.664640900000002"
Properties=species:S:1:pos:R:3:forces:R:3

Cu	16.43466316	14.84883092	15.71464791	-0.04766688	0.01033105	0.00012543
Cu	14.19166899	15.07700290	16.56125376	-0.02411694	-0.02206305	0.00757608
Cu	14.40188765	14.53489646	14.24493882	-0.01342883	0.02025243	0.02193300
Cu	12.78948494	13.28676612	15.46441427	0.07200675	0.00663588	-0.06333206
Cu	15.11132907	12.88636103	16.00391480	0.00781034	0.06329543	0.01268697
Cu	12.39993769	15.65662407	15.02107901	0.09664253	0.03346961	-0.02465813
H	11.44935153	12.86455199	17.42810706	0.01155148	0.00511073	0.00611511
O	10.64106321	14.12279863	17.36710977	-0.00110538	0.00980023	-0.00607446
H	10.65087041	14.22381496	16.38643811	-0.00540103	0.00315136	0.00676934
H	18.51164443	13.63992039	15.11403909	-0.00646050	-0.00121960	-0.00459983
O	18.03763482	14.33461612	14.53444562	-0.02952081	-0.00015315	0.00923716
H	17.56852651	13.76704285	13.83695671	-0.01259998	-0.00493829	-0.00147740
H	13.16487564	14.07403372	20.77789611	0.00384049	0.00186635	-0.01790546
O	13.84806745	14.43606887	20.17668017	0.00636077	0.00910817	-0.02830094
H	14.39808300	13.62759495	19.90756151	-0.00028249	0.01251774	-0.02895063
H	14.50677156	11.63292818	14.37464288	0.00566169	0.01306739	-0.01167418
O	14.42978890	10.74610039	13.89675378	0.00754719	0.01700899	0.00161305
H	13.82669815	11.03303661	13.12530720	0.01123553	0.02571598	0.02615678
H	13.52992777	17.86741599	11.62871761	-0.00449744	-0.01248139	0.01833447
O	12.63277569	17.71995996	12.11207758	0.00596196	-0.01055101	0.02384222
H	12.84746458	18.23677178	12.96619010	0.00067057	-0.02335797	0.01881668
H	10.77334144	18.17895086	12.53750130	0.00548965	-0.01411251	0.01461046
O	9.99160274	18.30667091	13.12770137	0.02168995	-0.01815366	0.00908586
H	10.10850550	17.59055723	13.81741135	0.02265622	-0.00825419	0.00300134
H	15.27426064	11.79048901	18.65707485	-0.00854207	0.02109143	-0.02228247
O	15.29254946	12.28944383	19.51684092	-0.00625602	0.01104185	-0.02867367
H	16.21191174	12.67281573	19.46396824	-0.01312705	0.00741452	-0.02063272
H	14.26559160	19.78861445	13.60995792	-0.00005327	-0.01475254	0.00819644
O	13.40842232	19.45254861	13.94748873	0.00246065	-0.02541230	0.00623327
H	13.63287995	19.05695283	14.87376344	0.00157731	-0.02364179	-0.00052415
O	12.13555284	17.47223249	19.08057953	0.00926001	-0.00752650	-0.02535494
H	12.33557329	16.59426854	18.52547413	0.01858967	-0.00719756	-0.02886543
H	14.89406112	16.86173088	19.55800962	-0.00972803	-0.00944171	-0.02008928
O	14.46409154	17.72842693	19.80847695	-0.00001100	-0.01886848	-0.02189618
H	14.93588010	18.44058318	19.13708780	-0.00613180	-0.01790504	-0.01371026
H	15.33681979	14.89452141	11.68516790	-0.00934633	0.00643966	0.02152812
O	14.63196604	14.34347355	12.20463937	0.00230558	0.01144826	0.02732302
H	13.69759213	14.72843569	11.93011424	-0.00034163	0.00484066	0.00418872
H	12.91148955	12.47518356	17.94532307	0.02397063	0.01806395	-0.00111001
O	12.22046557	12.13661046	17.32932336	0.01735665	0.01398882	-0.00517847

H	11.35369222	10.88285882	19.26858037	0.01433768	0.01580863	-0.02340543
H	10.84213910	14.75867021	12.63169181	0.02179042	0.00065371	0.03156646
O	10.10036812	14.49396926	13.25441146	0.01251343	0.00627086	-0.00192803
H	10.15633853	15.25007003	13.94423040	0.00237858	-0.00897167	-0.00780844
H	12.01376058	15.31462006	10.78413529	0.00756275	0.00140566	0.01738904
O	12.27920256	15.28753189	11.72938118	0.00107720	-0.00403735	0.01438792
H	12.41689760	16.33385109	11.99650627	0.00795387	-0.00986234	0.03145744
H	17.19515895	15.99645980	11.66024813	-0.01105781	-0.00879063	0.01864393
O	16.43248576	15.71437638	10.95553810	-0.00746735	-0.00772680	0.01925092
H	16.88457254	15.39834809	10.14645533	-0.00355835	-0.00012336	0.01433539
H	16.88469976	18.48750303	14.53547584	-0.01857870	-0.02133619	0.00758390
O	16.23325322	17.91050925	13.93210406	-0.02567089	-0.02152524	0.01191677
H	15.80189028	17.36657720	14.64424480	-0.02147798	-0.02510815	-0.00054250
H	16.93154736	14.23480640	18.44067760	-0.02171661	-0.00432303	-0.02745106
O	17.64336523	13.53771822	18.59599227	-0.01098764	-0.00227072	-0.02371134
H	17.78758903	13.10946206	17.71677746	-0.03297478	0.01695107	-0.02123102
H	17.91610585	16.39900629	18.73835696	-0.00811908	0.00098764	-0.01362361
O	18.85842398	16.12209956	18.58212641	-0.01998941	-0.00657448	-0.01081809
H	18.73751017	15.13442818	18.61603097	-0.01132722	0.00386578	-0.01535751
H	15.53247992	17.31347623	10.96021777	-0.00827687	-0.00417922	0.01724753
O	15.07977306	18.11998365	11.32933318	0.00350825	-0.01877614	0.02220543
H	15.44912970	18.14151578	12.25720445	-0.00493851	-0.01647614	0.00858419
H	14.60483841	10.52094388	16.67902486	-0.00361510	0.00095268	-0.00185814
O	15.45549896	11.13412884	16.95835117	0.00472382	0.02974236	-0.01693814
H	16.20973473	10.80513733	16.40385798	-0.00231065	0.00197363	0.01076384
H	14.73479030	19.23959399	17.48051130	-0.00046953	-0.01777419	-0.00501767
O	15.47526885	19.32100660	18.15736402	-0.00268018	-0.01546065	-0.01503266
H	16.12687811	18.70407143	17.71588982	-0.00261946	-0.01516210	-0.01095827
H	10.65968551	13.39395909	13.86885699	0.01658942	0.01728628	0.00628080
O	11.22829097	12.54635098	14.30533162	0.03014620	0.00650132	0.01967572
H	10.58432808	11.90635780	14.67914036	0.01375210	0.01041997	0.00499319
H	10.93360050	17.77351935	16.08967338	0.01861968	-0.00720871	-0.00429592
O	10.59245129	16.40719970	15.04580813	0.03222042	-0.00180846	0.00428568
H	10.05871806	16.08520634	15.81083448	-0.00114787	0.00125250	0.00843212
H	10.96661131	19.35390783	15.98800215	0.01019915	-0.01921664	-0.00289341
O	11.16755201	18.57300245	16.65633542	0.00870608	-0.01856201	-0.01056801
H	11.76989651	18.09870049	18.40161630	0.01504842	-0.01451515	-0.00751639
H	19.10503282	16.61683046	17.08099461	-0.02221025	-0.00863968	-0.01308673
O	19.25557553	16.93086930	16.11322005	-0.02561323	-0.01441749	-0.01090477
H	18.75510071	16.26443245	15.59591391	-0.01398170	0.00424306	0.00655124
H	18.54519296	19.60734123	15.01367647	-0.00614874	-0.01332002	0.00164766
O	17.86948872	19.08947562	15.49991550	-0.01451149	-0.01444822	0.00740674
H	18.39571855	18.26030525	15.82195167	-0.01790583	-0.01208761	0.00107238
H	12.77636747	10.60286140	16.54281241	0.01171826	0.02596466	-0.01370311
O	13.40636706	9.90584932	16.20682995	-0.00358360	0.01700324	-0.00406001

H	13.57325873	10.14745936	15.23751120	0.00204723	0.03284980	0.00674557
H	17.05082380	12.43278724	12.02789598	-0.01449150	0.01309034	0.01517465
O	16.68223668	12.71575638	12.89322320	-0.01182075	0.00557026	0.00890580
H	15.79178943	13.12946777	12.67089444	0.00279122	-0.00832429	0.01474194
H	12.16326630	12.10694366	12.84457181	0.01982222	0.02569669	0.01914555
O	12.82040511	11.89758215	12.11737207	0.00511016	0.02745585	0.01771621
H	13.35167187	12.72427828	12.05543214	-0.00135454	0.01433180	0.03294920
H	11.75949376	20.31493450	14.42753777	-0.00216843	-0.01325179	0.00026433
O	10.82193916	20.29434941	14.75020755	0.01444688	-0.01543320	-0.00383924
H	10.41179880	19.63757413	14.09264252	0.01318005	-0.00640038	0.00625001
H	13.95394132	17.57061844	16.33021261	-0.01096411	0.00066870	-0.00572350
O	13.66917744	18.52002310	16.32977515	0.00593843	-0.02673569	0.00160483
H	12.62264326	18.50565599	16.50753171	0.01177045	-0.02880106	-0.00879541
H	19.74467058	12.09185131	16.30440411	-0.01621478	0.00603823	-0.00071418
O	18.82195526	12.36539346	16.11775068	-0.01907886	0.00602633	0.00016279
H	18.38845634	11.58771875	15.65375756	-0.02051027	0.01647173	0.00428893
H	16.78925058	9.88947349	14.48046281	-0.01260142	0.02067620	0.00314441
O	17.23727378	10.72665638	14.73166504	-0.01331954	0.01717112	-0.00574020
H	16.79957448	11.38767509	14.11876347	-0.01633723	0.02375672	0.02012167
H	18.31315921	15.76650282	13.31141048	-0.02318317	0.00242739	0.01227076
O	18.05163645	16.49994456	12.70171790	-0.01492419	-0.00573142	0.02149337
H	17.35963071	17.03468993	13.25099263	-0.01029180	-0.01909689	0.01484471
H	13.18980318	14.93995034	18.81007561	0.00027475	-0.00110130	-0.01133570
O	12.80185631	15.31779287	17.94274000	0.02594777	0.00896947	-0.00720588
H	11.52282516	14.68476269	17.63620281	0.02535320	0.00339740	-0.02938154
H	12.20185631	15.40779287	17.94274000	0.00153802	-0.00154289	0.00125874
C	15.86861292	16.03921207	17.27414658	-0.00637338	0.00632125	-0.00779745
O	16.06880323	15.75335376	18.51670870	-0.02091900	-0.00297919	0.00013366
O	16.21367022	17.14514027	16.71343560	0.01238512	-0.00334497	0.00149995
H	11.06812005	10.54502373	19.89475432	-0.00065551	-0.00065368	0.00581334

*COOH;*OH

114

Lattice="29.753067899999998 0.0 0.0 0.0 30.425461 0.0 0.0 0.0 30.6314408" Properties=species:S:1:pos:R:3:forces:R:3

Cu	16.45044266	14.90513143	15.31712217	-0.04138994	0.02454911	0.01548621
Cu	14.20391249	15.27172806	16.25574827	-0.02055202	-0.02214720	0.01397620
Cu	14.36493218	14.57478527	13.98379131	-0.02202592	0.01381992	0.01470122
Cu	12.80324887	13.44925657	15.32843181	0.07897548	-0.00640576	-0.07413456
Cu	15.19538773	12.97023378	15.75294639	0.00386768	0.05056093	0.04583980
Cu	12.34891869	15.76635318	14.78728536	0.09944797	0.03209740	-0.02642683
H	11.44798825	12.88867784	17.19545094	0.01039607	0.00473196	0.00351444
O	10.60955680	14.18480872	17.15865431	0.00025727	0.01363694	-0.00932590
H	10.74379423	14.40608643	16.20207950	-0.01045382	0.00025491	0.01219208
H	18.62921354	13.72255003	14.92483147	-0.00428333	0.00069924	-0.00212295

O	18.28000656	14.45381509	14.30760536	-0.02800544	-0.00050743	0.01176271
H	17.84929616	13.91600895	13.57090617	-0.00800252	-0.00792552	-0.00420880
H	13.46600201	13.87013252	20.64700887	0.00339897	0.00198637	-0.01503549
O	14.06943595	14.19856223	19.94960111	0.00548308	0.01021288	-0.02621835
H	14.71284747	13.41543650	19.75550041	-0.00067949	0.01197935	-0.02481642
H	14.59576254	11.70365592	14.15099294	-0.00563286	0.01884389	-0.00333601
O	14.45470515	10.81226653	13.70022907	0.00173090	0.02298052	0.00131606
H	13.85198776	11.11141643	12.92719943	0.01073310	0.02624137	0.02603441
H	13.38231334	18.08005953	11.41237556	-0.00306589	-0.01211766	0.01523671
O	12.55248592	17.93118752	11.93935832	0.01142844	-0.01025701	0.02100123
H	12.83143152	18.42330864	12.80267242	0.00038017	-0.02220456	0.01762793
H	10.59372732	18.34587315	12.44763386	0.00450113	-0.01445891	0.01410824
O	9.84825444	18.45570005	13.08255900	0.02203234	-0.01633814	0.00656165
H	10.00766756	17.71374453	13.74688795	0.02317060	-0.00808286	0.00003919
H	15.50198551	11.79657898	18.60871682	-0.00700779	0.01884563	-0.02154210
O	15.72962522	12.25904199	19.46173628	-0.00497059	0.00754251	-0.02491326
H	16.54840886	12.77104843	19.19084471	-0.01237375	0.00496281	-0.01792614
H	14.22274861	19.90744354	13.55056627	0.00192965	-0.01600165	0.00704862
O	13.39130244	19.47890448	13.84192413	0.00293254	-0.02486854	0.00539819
H	13.61716577	19.06570482	14.78808360	0.00249250	-0.02571139	-0.00041182
H	12.98574199	17.72783805	19.23948286	0.00176698	-0.00819247	-0.02336308
O	12.06392758	17.38613049	18.92841840	0.00781894	-0.00401549	-0.02418345
H	12.53509371	16.19332807	18.29380152	0.01387232	-0.00827464	-0.02835631
H	14.90132396	16.99358263	19.49607556	-0.00615397	-0.00929678	-0.02054539
O	14.49003923	17.85580042	19.73773427	0.00466466	-0.01742556	-0.02503266
H	14.97686937	18.49833171	19.12021863	-0.00475445	-0.01699519	-0.01193219
H	15.22936109	14.96415072	11.46849011	-0.00442228	0.00257541	0.01845549
O	14.49455767	14.44116782	11.92304795	0.00276969	0.00815859	0.02820651
H	13.61039380	14.88945815	11.64445095	0.00073579	0.00608304	0.00430282
H	12.89518976	12.53165261	17.75419916	0.02516196	0.01487794	-0.00067670
O	12.24333032	12.21285352	17.08836031	0.01850238	0.01654094	-0.00851509
H	10.76623739	14.85796253	12.43985650	0.02165821	0.00048813	0.03077572
O	10.05634207	14.57353472	13.08437246	0.01188494	0.00663591	-0.00170361
H	10.10384394	15.33654533	13.76229456	0.00109302	-0.00819724	-0.00797209
H	11.84890331	15.52379981	10.55000241	0.00681295	0.00121933	0.01630025
O	12.16057578	15.46456016	11.47890521	0.00246394	-0.00427554	0.01648395
H	12.29917574	16.46855905	11.77338751	0.00684560	-0.01030714	0.03187604
H	17.80262567	16.25255077	11.92414174	-0.01085182	-0.00735613	0.02186358
O	16.42632469	15.95250601	10.74029701	-0.00646829	-0.00502533	0.02212226
H	16.58650757	15.83117952	9.78086989	-0.00427487	0.00285984	0.01310277
H	17.44952823	19.08037125	14.66402193	-0.00906188	-0.01035797	0.00611696
O	16.30498125	18.04386531	13.66907308	-0.01832208	-0.01643543	0.01005499
H	16.07221574	17.63056139	14.57792261	-0.01555506	-0.02435702	0.00240281
H	16.96919806	14.40613069	18.04417430	-0.02221345	-0.00897274	-0.02786028
O	17.70535261	13.78590254	18.30993921	-0.01509151	-0.00026947	-0.02477488

H	17.93659704	13.29892162	17.47383298	-0.03033677	0.01864565	-0.02157055
H	17.22242052	16.45223215	18.20124418	-0.01405402	-0.00322289	-0.01298196
O	18.60497374	16.50360819	18.39632047	-0.01212040	-0.00334636	-0.01164843
H	18.73288219	15.52361934	18.48181637	-0.00762506	0.00620142	-0.01590442
H	15.97046154	16.87006981	10.83935173	-0.00784779	-0.00786717	0.01378732
O	15.24667618	18.18814589	11.34817819	-0.00484655	-0.01409711	0.01687940
H	15.55677255	18.13851025	12.35386892	-0.00771142	-0.01620071	0.01252462
H	14.51786544	10.59979333	16.55373377	-0.00310262	0.00634008	-0.00155773
O	15.32436815	11.24448310	16.90912479	0.00552438	0.03074531	-0.01896655
H	16.15579147	10.83782337	16.57278464	0.00004237	-0.00078447	0.00767213
H	14.88516269	19.29126919	17.26487384	0.00031503	-0.01817838	-0.00725889
O	15.61651369	19.50231978	17.91392387	-0.00144358	-0.02378472	-0.01810154
H	16.37341143	19.03275131	17.49397596	0.00025689	-0.01368525	-0.00828243
H	10.69308624	13.44939913	13.69829417	0.01744059	0.01693008	0.00649179
O	11.27161724	12.61929590	14.09714713	0.02997787	0.00545301	0.01882407
H	10.63228874	11.96285674	14.44962616	0.01826187	0.01367170	0.00643202
H	10.97791818	17.85099210	15.92967390	0.01662067	-0.00428047	-0.00286614
O	10.52162431	16.54080123	14.85245483	0.03086044	-0.00138007	0.00406124
H	9.94298788	16.23922611	15.59153040	-0.00014341	0.00036692	0.00823789
H	10.99792829	19.43319808	15.89944848	0.00961636	-0.01929586	-0.00390785
O	11.22317794	18.62733461	16.52508532	0.00805245	-0.01874048	-0.00971803
H	11.73341402	18.00411543	18.22677294	0.01507589	-0.01361767	-0.00739111
H	18.95393972	16.72611169	17.45333427	-0.02005245	-0.00914769	-0.00617479
O	19.33134867	17.01347243	15.91460335	-0.02775765	-0.01192467	-0.01263168
H	18.69724855	16.38950651	15.49774717	-0.01210386	0.00763725	0.00528202
H	18.83867778	19.77105780	14.62348733	-0.00924474	-0.01194265	-0.00137604
O	18.18873070	19.39878493	15.25612358	-0.01867201	-0.01822482	0.00357142
H	18.88528501	17.90528548	15.72822771	-0.01877417	-0.02084285	-0.00035645
H	12.74530389	10.66857930	16.29109403	0.01291135	0.02703925	-0.01625770
O	13.39176284	9.97586908	15.98024592	-0.00107862	0.01727345	-0.00797716
H	13.60810544	10.23283476	15.01965204	0.00165338	0.03220359	0.00658271
H	16.21222457	12.74454078	12.06766703	-0.01416866	0.01286919	0.02739065
O	16.73816929	12.81743175	12.89558827	-0.01092899	-0.00007023	-0.00685017
H	16.04097078	13.16474957	13.59107879	0.00099436	-0.01650708	-0.02616307
H	12.21515194	12.20606835	12.63851744	0.01983613	0.02661671	0.02037738
O	12.87124712	11.98047906	11.91483708	0.00518994	0.02643657	0.01825267
H	13.41997355	12.80005452	11.84726528	0.00013476	0.01380519	0.03320829
H	11.76133310	20.37214670	14.34485716	-0.00230114	-0.01365122	-0.00014761
O	10.83301558	20.41087083	14.69250259	0.01447079	-0.01601840	-0.00505429
H	10.36551725	19.77398170	14.05313171	0.01371046	-0.00660540	0.00589739
H	13.93297693	17.60484385	16.14050304	-0.00330945	0.00180141	-0.00334131
O	13.71017674	18.58025736	16.16160796	0.00665475	-0.02347897	0.00381855
H	12.66672352	18.59233924	16.37849087	0.01240640	-0.02650770	-0.01056714
H	19.56795100	12.02141140	16.12444281	-0.01636171	0.00573606	-0.00252482
O	18.68813699	12.42476177	15.96067495	-0.02108173	0.00556715	0.00123585

H	18.13797014	11.69484528	15.51039590	-0.02513499	0.02065953	0.00314219
H	16.50616980	10.15214484	14.44128007	-0.00415881	0.01970721	0.00646440
O	17.30038237	10.73148812	14.49698553	-0.01498672	0.01517942	-0.00226718
H	17.07440753	11.42117217	13.79782435	-0.01729085	0.01884352	0.01991245
H	18.58127607	15.92648516	13.24918697	-0.02220848	0.00139402	0.00988181
O	18.31532696	16.68044826	12.66265929	-0.01859725	-0.00271845	0.01699120
H	17.10760888	17.48164611	13.35906473	-0.01570258	-0.01838066	0.01684588
H	13.32327496	14.80289426	18.75041757	0.00493462	0.00255485	-0.02446347
O	12.85999325	15.22598469	17.93160451	0.02301576	0.00770374	-0.00954464
H	11.43087002	14.67331879	17.55991901	0.02493940	0.00743885	-0.03212124
C	15.76100354	16.33744174	16.77591640	-0.00997814	0.01368143	0.01066898
O	16.18073160	16.08123233	18.04324979	-0.02108408	-0.00129134	-0.00358594
O	16.42701548	17.22064925	16.11493076	0.00080676	0.00480319	-0.00447651
H	15.69280254	18.98977252	11.00154854	-0.00309125	-0.00811612	0.01302782

*HCOOH;*OH

115

Lattice="29.7196966 0.0 0.0 0.0 30.4350017 0.0 0.0 0.0 30.866138999999997" Properties=species:S:1:pos:R:3:forces:R:3

Cu	16.66955458	14.81116332	15.50283994	-0.03604375	0.04921061	0.03287455
Cu	14.47146250	15.17602552	16.45011925	-0.00071883	0.01705800	0.01257361
Cu	14.54405440	14.51416088	14.17412177	-0.01768882	0.01470072	0.02047812
Cu	12.96071999	13.38413731	15.50717610	0.06796447	0.00122287	-0.07077776
Cu	15.34460451	12.91285735	15.91841305	0.00617092	0.05599974	0.04547192
Cu	12.52477170	15.68189851	15.03442856	0.10192848	0.04599848	-0.02046754
H	11.58424993	12.93981317	17.38472092	0.01053582	0.00545834	0.00214075
O	10.85129641	14.24858880	17.44037569	0.00031173	0.01422555	-0.00491115
H	10.89881672	14.47872323	16.47704151	-0.01050895	-0.00096745	0.01386532
H	18.81430085	13.68530066	15.08835062	-0.00314268	0.00017064	-0.00337564
O	18.46702775	14.40410203	14.45463939	-0.02751052	0.00370741	0.01429982
H	18.05777302	13.86173079	13.70812965	-0.00778317	-0.00678945	-0.00401301
H	13.64997427	13.90948922	20.84518718	0.00328830	0.00192394	-0.01510722
O	14.26126078	14.20293340	20.13947831	0.00522432	0.01035616	-0.02698071
H	14.87479880	13.39927132	19.95187942	-0.00054031	0.01191877	-0.02424349
H	14.67184301	11.69054861	14.35851266	0.00112350	0.01081053	-0.00654924
O	14.54110113	10.79041788	13.91438560	0.00178345	0.02107366	0.00048260
H	13.98414793	11.09484417	13.10762279	0.01062105	0.02627784	0.02654220
H	13.52352986	18.15204352	11.59837747	-0.00249290	-0.01159998	0.01458205
O	12.66957984	18.01849375	12.08918822	0.01129039	-0.00995777	0.01992570
H	12.94866255	18.47053881	12.98466098	0.00141281	-0.02088686	0.01632479
H	10.75801810	18.39133630	12.59904178	0.00397382	-0.01376624	0.01286819
O	10.01358948	18.45602955	13.24091469	0.02181869	-0.01689744	0.00763456
H	10.21665739	17.71952191	13.89415971	0.02257193	-0.00741354	0.00049360
H	15.64313707	11.78266226	18.75362458	-0.00698999	0.02039683	-0.02215153
O	15.88017424	12.21815694	19.61622806	-0.00532542	0.00753077	-0.02509127

H	16.69835267	12.74205184	19.36076148	-0.01215396	0.00489818	-0.01904516
H	14.32578385	19.88487092	13.75695334	0.00228878	-0.01551257	0.00789836
O	13.49655690	19.44531752	14.03924304	0.00437664	-0.02604376	0.00553976
H	13.66983970	19.16885251	15.06854175	0.00261676	-0.02550124	-0.00041638
H	13.19686071	17.75608386	19.48177197	0.00278644	-0.00725142	-0.02232124
O	12.27866918	17.38079735	19.15126175	0.00674816	-0.00304482	-0.02380339
H	12.79864852	16.30258489	18.47929781	0.01785309	-0.00843620	-0.03153198
H	15.08628024	17.10104661	19.54691938	-0.00626944	-0.00989276	-0.02321679
O	14.65975045	17.90026875	19.94827869	0.00517833	-0.01703926	-0.02569889
H	15.07976488	18.63293607	19.38048950	-0.00415191	-0.01688392	-0.01213808
H	15.36195430	15.00342819	11.70482062	-0.00298015	0.00322137	0.01497203
O	14.61888755	14.45575829	12.12906553	0.00134396	0.00555565	0.02927474
H	13.73761588	14.92936772	11.87316158	-0.00038424	0.00615571	0.00491580
H	13.00519270	12.44659575	17.90996692	0.02583698	0.01614098	0.00218537
O	12.33528233	12.21040748	17.22706019	0.01890437	0.01629232	-0.01043541
H	10.94735924	14.88623763	12.60829218	0.02179011	-0.00022712	0.03062272
O	10.23640717	14.58287805	13.24575855	0.01134909	0.00669054	-0.00201205
H	10.29148189	15.33433006	13.93621370	0.00090751	-0.00735229	-0.00892250
H	12.00008515	15.58543377	10.75895847	0.00671831	0.00141137	0.01581141
O	12.30935058	15.55870862	11.69018233	0.00389910	-0.00387937	0.01791977
H	12.43842149	16.58340356	11.95282124	0.00682575	-0.01046922	0.02943420
H	17.93178695	16.27999206	12.13009031	-0.00998335	-0.00760316	0.02183958
O	16.51339864	15.96648496	11.06067126	-0.00539753	-0.00612052	0.02205882
H	16.66569641	15.82965342	10.10141256	-0.00429629	0.00253023	0.01272147
H	17.56159342	19.05058354	14.84747249	-0.01024717	-0.01266752	0.00612675
O	16.46279165	18.02846991	13.92295291	-0.01365804	-0.01879582	0.01004679
H	16.11915189	17.67177765	14.86170649	-0.01043074	-0.03003839	-0.00150889
H	17.12360759	14.48190321	18.36016227	-0.02319646	-0.00973851	-0.02876158
O	17.84211571	13.81525344	18.57314223	-0.01600066	0.00245802	-0.02551281
H	18.04596976	13.36994058	17.70845978	-0.03000328	0.01834374	-0.02269274
H	17.25779959	16.44836290	18.56670975	-0.02026542	-0.00251948	-0.01780367
O	18.79277195	16.49111814	18.78542956	-0.01353431	-0.00380268	-0.01684287
H	18.88349472	15.50525119	18.81975216	-0.00540487	0.00509666	-0.01461646
H	16.06251153	16.89841687	11.15841501	-0.00894792	-0.00817692	0.01472941
O	15.40218804	18.20414987	11.64241594	-0.00570346	-0.01381834	0.01688908
H	15.74999000	18.16521502	12.65354735	-0.00896926	-0.01613072	0.01075676
H	14.60683160	10.57016351	16.72227414	-0.00410510	0.00624303	-0.00152500
O	15.44917121	11.21790168	17.04731887	0.00561537	0.03054331	-0.01886775
H	16.28173602	10.74618920	16.82157735	0.00221473	-0.00017082	0.00301348
H	14.90460227	19.63591347	17.61839702	-0.00100553	-0.01594665	-0.00582701
O	15.69346787	19.68545016	18.22712202	-0.00166568	-0.02220359	-0.01664760
H	16.31135486	19.09435076	17.73427341	0.00363885	-0.01303057	-0.00779244
H	10.85927993	13.45877784	13.83728770	0.01939612	0.01650576	0.00750275
O	11.43040444	12.61511106	14.23260365	0.02924529	0.00392865	0.01933801
H	10.77902867	11.95129883	14.54693935	0.01912419	0.01382710	0.00758409

H	11.08463698	17.85199961	16.16772645	0.02041059	-0.00979571	-0.00638331
O	10.74121932	16.52166144	15.02251957	0.03059664	0.00055606	0.00234176
H	10.11471979	16.26832597	15.74017558	-0.00180486	0.00203604	0.00715643
H	11.08961966	19.41829752	16.16939279	0.00967921	-0.01820679	-0.00270746
O	11.24949663	18.61352212	16.80003328	0.01025783	-0.01959832	-0.01163929
H	11.90119903	18.00354719	18.47718163	0.01574131	-0.01251976	-0.00716653
H	19.13190264	16.72802869	17.86391759	-0.01471770	-0.00822006	-0.00174516
O	19.51528990	17.11709045	16.23550624	-0.02347775	-0.01100789	-0.01194389
H	19.25480606	16.44106446	15.57528909	-0.02688522	-0.00453038	-0.00044598
H	18.94392742	19.77376529	14.84157949	-0.00858159	-0.01144932	-0.00165908
O	18.28914784	19.37969703	15.45497518	-0.01971700	-0.01931521	0.00479676
H	19.02351804	17.95884018	15.94075695	-0.01898394	-0.01950589	-0.00049276
H	12.82367850	10.66554963	16.50321915	0.01321784	0.02438279	-0.01657293
O	13.47876056	9.97221049	16.20701693	-0.00052132	0.01531539	-0.00785275
H	13.68402229	10.20746547	15.23611578	0.00189407	0.03273459	0.00622582
H	16.36832695	12.77361540	12.21580075	-0.01481714	0.01282124	0.02888958
O	16.90740789	12.80039114	13.03849674	-0.01188033	0.00116330	-0.00668510
H	16.22506978	13.12690941	13.75293369	0.00074469	-0.01458608	-0.02729950
H	12.38624305	12.20811021	12.80426985	0.02034642	0.02609686	0.02101885
O	13.05299915	11.98304760	12.08800437	0.00529086	0.02609518	0.01769417
H	13.60132354	12.80436239	12.03053575	0.00020717	0.01282949	0.03258208
H	11.93864031	20.35267145	14.53683067	-0.00229131	-0.01260616	-0.00125421
O	11.00723371	20.41035226	14.87462184	0.01356647	-0.01596291	-0.00449527
H	10.53665462	19.78360821	14.23513059	0.01348090	-0.00591480	0.00567451
H	14.39195821	18.07851307	16.49916477	-0.01371597	-0.03195739	-0.00089757
O	13.76985703	18.88522296	16.44403223	-0.00340419	-0.02642732	0.00333388
H	12.79133488	18.64853675	16.67870840	0.01535338	-0.02516971	-0.00933776
H	19.67389460	11.98446488	16.37910474	-0.01739818	0.00665095	-0.00272038
O	18.81591306	12.42733652	16.19873923	-0.01960428	0.00326554	0.00183998
H	18.25234091	11.72141250	15.72225880	-0.02704529	0.02175859	0.00395730
H	16.58823604	10.27301602	14.64151053	-0.00361924	0.02159298	0.00773991
O	17.46979905	10.71932670	14.65117062	-0.01587643	0.01570514	0.00083526
H	17.31281835	11.39270094	13.92131117	-0.01717002	0.01816632	0.01910552
H	18.74428848	15.87789991	13.40151091	-0.02313076	0.00081543	0.01099214
O	18.48529169	16.66719454	12.86340903	-0.01871811	-0.00225517	0.01874582
H	17.24517736	17.43817912	13.64183702	-0.01640182	-0.01933905	0.01833670
H	13.60753358	14.88787274	18.89362166	0.00239577	0.00103684	-0.01747844
O	13.18789054	15.34353727	18.06907945	0.02410034	0.00806578	-0.00960888
H	11.70504650	14.73401858	17.76441196	0.02528034	0.00509464	-0.03348308
C	16.28390271	16.29375783	16.85107536	-0.00696243	-0.00329239	-0.00559720
O	16.28981317	16.16343631	18.26928051	-0.02099050	0.00027639	0.00421713
O	15.83266605	17.45564492	16.33124553	-0.00360239	0.00215549	0.00551779
H	15.86603928	18.97343471	11.24872149	-0.00302366	-0.00848890	0.01289736
H	17.39195612	16.15877897	16.54632606	-0.01939989	-0.01577295	-0.01656190

*CHO;*OH
116
Lattice="29.682498000000002 0.0 0.0 0.0 30.4228166 0.0 0.0 0.0 30.5543331"
Properties=species:S:1:pos:R:3:forces:R:3
Cu 16.70983813 15.21371843 15.67726270 -0.05700701 -0.02167928 0.00705912
Cu 14.48375229 15.42639994 16.30833044 -0.00854256 0.00392698 -0.01620787
Cu 14.62872208 14.94300450 14.02541931 -0.02699242 0.00404931 0.00530565
Cu 13.07604716 13.61992154 15.23205044 0.06528354 0.04583157 0.00138165
Cu 15.36451914 13.21336212 15.71832805 -0.00524943 0.02835883 0.05324224
Cu 12.66295317 16.04168468 14.95147159 0.06993625 0.00677116 -0.02780947
H 11.27600192 12.81475807 17.76520732 0.01945718 0.01464995 -0.01950670
O 10.62294071 13.41608908 17.30081871 0.01762202 0.00296028 -0.01075672
H 10.87476425 13.31387512 16.34401110 0.00057388 0.01247639 0.00948783
H 18.99030258 13.52867539 14.56434667 -0.03221897 0.01365751 0.00422337
O 19.05863298 13.98346239 13.67571494 -0.01865787 -0.01045755 0.00559114
H 18.39491527 13.46321814 13.15190925 -0.01647795 0.00926515 0.01866112
H 12.58798959 13.13438676 20.33895370 0.00527232 0.00818883 -0.00618994
O 13.01983163 14.00453607 20.51390840 0.01169405 0.00205753 -0.02786080
H 13.90023910 13.88789181 20.08923491 0.00104465 0.00741772 -0.01386415
H 14.64966851 11.48112628 14.36526651 -0.00805155 0.01010324 -0.00914418
O 14.41184510 10.62433244 13.89798988 0.00724544 0.02126888 0.00609410
H 13.79460371 11.00405258 13.17999851 0.01223069 0.02411090 0.02231597
H 13.51843029 17.95510276 11.59789256 -0.00206287 -0.01592273 0.02254250
O 12.61339471 17.81277803 12.08189807 0.00930323 -0.01020722 0.02115384
H 12.83440191 18.24610856 12.97480356 0.00337632 -0.02790092 0.02148147
H 10.75980417 18.32261791 12.62603200 0.00679107 -0.01426421 0.01528894
O 10.00604042 18.42750780 13.25677145 0.02295962 -0.01889925 0.00775259
H 10.15658015 17.69860052 13.92107670 0.02388597 -0.00802078 0.00153847
H 15.43621090 11.54054263 18.33417773 -0.00658751 0.02149811 -0.01970142
O 15.24585325 12.24841771 19.07877442 -0.00859226 0.01196683 -0.01944705
H 15.39601395 11.74813875 19.91043771 -0.00543073 0.00842581 -0.01383766
H 14.23877407 19.70299532 13.64623730 -0.00351788 -0.01678313 0.00837046
O 13.39408655 19.41862231 14.05684376 0.00108083 -0.02818840 0.00490256
H 13.67988873 19.07139941 14.97972261 -0.00273408 -0.02760965 -0.00304826
H 12.75114590 18.11865316 19.60438965 0.01229049 -0.00911017 -0.01887508
O 11.43920669 17.89607598 19.29472627 0.01094164 -0.00732269 -0.01935792
H 11.46201012 16.91158095 19.01675568 0.01492223 0.00138406 -0.01701225
H 14.35461577 17.39130033 19.52950818 -0.01476737 0.00072260 -0.01666883
O 13.81428580 18.21165366 19.93266507 -0.00011343 -0.01278819 -0.02065069
H 15.17258814 14.86780999 11.46330632 -0.00678996 0.00361401 0.01094364
O 14.47352652 14.40405073 12.06761637 0.00320440 0.01394409 0.02980823
H 13.56819198 14.81439659 11.82001138 -0.00108493 0.00345411 0.00830216
H 13.41480462 12.06407682 18.69598376 -0.00084866 0.01785682 -0.03289663
O 12.44303185 11.82743524 18.69254271 0.01965807 0.01380367 -0.02407986
H 12.42394457 10.98304302 18.18406680 0.01057751 0.01524344 0.00024110

H	10.89059125	14.83658959	12.71217549	0.02595282	0.00122633	0.03034632
O	10.20563268	14.59698849	13.42820839	0.01216925	0.00058766	-0.00167966
H	10.35540199	15.41360594	14.14928401	0.00294895	-0.01276272	-0.00666765
H	11.72382773	15.41656787	10.76539541	0.00857177	0.00248588	0.01613044
O	12.06604149	15.40083379	11.68519298	0.00688082	-0.00297396	0.01795073
H	12.28078923	16.43338735	11.92130305	0.01177914	-0.00831232	0.03223749
H	16.99250577	15.86604841	11.48836897	-0.01251961	-0.00795191	0.02224789
O	16.24677879	15.72154016	10.74230746	-0.00702937	-0.00433046	0.01963459
H	16.70376419	15.35715300	9.95631070	-0.00325989	0.00169808	0.01465801
H	16.58049151	18.19035374	14.48291648	-0.02517468	-0.02911121	0.00792222
O	15.92543487	17.81418275	13.79268841	-0.01923391	-0.00676410	0.00613479
H	15.31475875	17.22224646	14.32463907	-0.00984309	0.00543552	0.01054626
H	16.95206432	13.14495879	18.73915635	-0.00591410	0.01472389	-0.03005570
O	17.87106592	13.44069269	18.49685369	-0.02278481	-0.00018081	-0.01922085
H	18.05235487	13.03167749	17.60527183	-0.03181447	0.01454451	-0.01626816
H	18.22413916	16.67435761	18.29163637	-0.01603473	-0.01229514	-0.01735416
O	18.85981223	15.82561013	18.10438479	-0.01343927	-0.00411484	-0.00622574
H	18.35190336	14.95914582	18.34925476	-0.01993259	0.00964616	-0.02256802
H	15.43578164	17.37959347	10.81657095	-0.00794499	-0.00669444	0.01976242
O	15.03623250	18.19718123	11.22336192	0.00215051	-0.02021456	0.02072917
H	15.42492693	18.16359088	12.14377220	-0.00502936	-0.01997388	0.01062865
H	14.61563454	10.42686859	16.88460003	0.00566093	0.02644425	-0.00922038
O	15.55650283	10.46431325	17.26417237	0.00015923	0.01432751	-0.01207933
H	16.09366954	10.76818918	16.48199183	-0.00598345	0.01044262	0.00383580
H	14.80516059	19.47835880	17.67233391	0.00192141	-0.01748361	-0.00311022
O	15.22535514	19.84965547	18.50410939	-0.00051969	-0.01981755	-0.01383986
H	16.07891968	19.34641448	18.56227128	-0.01080110	-0.00561194	-0.00693584
H	10.63612865	13.72695455	13.93710504	0.01939135	0.01957211	0.00241157
O	11.40390515	12.79177610	14.60733120	0.03238162	0.00360505	0.00923271
H	11.08965349	11.89723412	14.86818977	0.00609984	-0.00309141	0.00159852
H	11.04947637	17.87412664	16.12837645	0.01265130	0.00139192	-0.00121193
O	10.71402611	16.42496784	15.05408656	0.03267778	0.00115169	0.00715834
H	10.34574701	16.04397042	15.88519052	0.00093624	-0.00846283	0.01039454
H	11.04772687	19.46074721	16.03274374	0.00965608	-0.01967272	-0.00310657
O	11.28501752	18.66737542	16.68644362	0.01065505	-0.01807176	-0.00802312
H	11.25649302	18.34241960	18.41800427	0.01334533	-0.01021211	-0.00546340
H	18.84069377	15.83677999	17.01238295	-0.00205683	-0.00271122	0.00989129
O	18.50796684	16.01774505	15.66079580	-0.02796848	-0.01599457	-0.00958615
H	19.10494882	15.60039086	14.99794716	0.00695596	0.00801807	0.00399403
H	18.43073522	19.10493377	15.34548984	-0.00775302	-0.01592137	0.00330425
O	17.75539179	18.42288205	15.54360515	-0.01576412	-0.01154197	0.00753267
H	18.22724858	17.48765874	15.48162276	-0.01153068	-0.00606841	-0.00166514
H	13.16633237	11.57838596	16.13450675	0.00633122	0.00634479	0.00456236
O	13.06590239	10.58263183	16.21433435	0.00164002	0.01903531	-0.00779133
H	13.41077510	10.32879543	15.29322344	0.00331388	0.02839239	0.00518677

H	15.98751804	12.55992869	12.37376833	-0.01223082	0.01827327	0.02632318
O	16.71783905	12.68407898	13.02271507	-0.01279631	0.01177350	-0.00021631
H	16.27214649	13.28037992	13.72427804	0.00523775	-0.00217008	-0.00514914
H	12.27916285	12.31470690	13.04980219	0.01633945	0.01620297	-0.00175633
O	12.82369263	12.00440278	12.26742131	0.00489340	0.02529685	0.01608676
H	13.43515903	12.76934284	12.11890207	0.00302709	0.01763879	0.03490414
H	11.77652303	20.41777389	14.52687288	-0.00152966	-0.01390399	0.00119350
O	10.84438598	20.41769988	14.86668292	0.01471054	-0.01528768	-0.00361867
H	10.41789395	19.76590908	14.21525541	0.01341900	-0.00653694	0.00618048
H	14.10355169	17.64176765	16.44925812	0.01334844	0.00757499	0.01165201
O	13.86130127	18.61590976	16.48753871	0.01109012	-0.01724754	0.01002806
H	12.82947565	18.60692272	16.65815786	0.01069660	-0.02965430	-0.00576522
H	19.70067682	12.04776219	16.28798859	-0.01467676	0.00768625	-0.00397152
O	18.83067503	12.43758536	16.05503497	-0.02335330	0.01688915	-0.00255360
H	18.30129420	11.66874064	15.67819920	-0.02154502	0.01824002	0.00162407
H	16.67071859	9.94732660	14.61842637	-0.00783251	0.02038722	0.00294063
O	17.08815072	10.79447810	14.88884548	-0.00908467	0.01256771	-0.00240254
H	16.86309736	11.41477696	14.12100748	-0.00857265	0.01589648	0.01379076
H	18.19549215	15.45467669	13.15617374	-0.03325286	0.00172426	0.01813822
O	17.83815106	16.22715505	12.62860552	-0.01830572	-0.00517330	0.02162631
H	17.16349729	16.69115340	13.21129289	-0.01526877	-0.02595465	0.01637707
H	12.19581597	14.94980922	19.30157934	0.00873552	0.00550453	-0.02404231
O	11.83245491	15.44957621	18.50780238	0.01682936	0.00319104	-0.02284874
H	11.28822393	14.75525306	18.00946298	0.02990650	0.00787336	-0.01984093
C	15.05332878	15.44320396	18.18404212	-0.01314340	0.00248327	-0.00516951
H	16.45736047	17.27193763	18.47884348	0.00198538	-0.00635556	-0.03076132
O	15.35382457	16.48042835	18.97521382	-0.00306996	-0.01019217	-0.01092478
O	17.32347962	17.83916499	18.26749228	-0.01513390	-0.00762142	-0.01967486
H	17.31246667	18.07451154	17.29788635	-0.02130324	-0.02415810	-0.00694088
H	15.61421644	14.51235108	18.38746151	0.00965260	-0.01326321	-0.01657210

$CH_2(OH)_2$;*OH

117

Lattice="29.6475516 0.0 0.0 0.0 30.2890283 0.0 0.0 0.0 30.3817086" Properties=species:S:1:pos:R:3:forces:R:3

Cu	16.79647460	14.59249140	15.04884840	-0.01279631	0.01177350	-0.00021631
Cu	13.72764580	15.00056580	16.68545840	0.01069660	-0.02965430	-0.00576522
Cu	14.48264340	14.70174210	14.42783880	0.01069660	-0.02965430	-0.00576522
Cu	12.72282780	13.32336180	15.30792320	0.01069660	-0.02965430	-0.00576522
Cu	15.27610400	13.18417460	16.09572260	0.01069660	-0.02965430	-0.00576522
Cu	12.27397730	15.63967060	14.91532210	0.01069660	-0.02965430	-0.00576522
H	11.44193490	12.89212280	17.35510830	-0.00872631	0.01177350	-0.00021631
O	10.62705430	14.19463540	17.44182130	0.01069660	-0.02965430	-0.00576522
H	10.89324500	14.50617220	16.53474590	0.01069660	-0.02965430	-0.00576522
H	19.04549360	13.67296410	15.05136720	0.01069660	-0.02965430	-0.00576522
O	18.81942370	14.39410180	14.37250070	0.01069660	-0.02965430	-0.00576522

H	18.50789490	13.86786990	13.58266000	0.01069660	-0.02965430	-0.00576522
H	13.80215170	13.87171840	20.99413760	-0.01279631	0.01177350	-0.00021631
O	14.30943020	14.17381410	20.21339610	0.01069660	-0.02965430	-0.00576522
H	14.91502170	13.37328850	19.96676300	0.01069660	-0.02965430	-0.00576522
H	14.75278630	11.84064190	14.45048180	0.01069660	-0.02965430	-0.00576522
O	14.53508850	10.95884110	14.03143740	0.01069660	-0.02965430	-0.00576522
H	14.02136270	11.27667180	13.18639780	0.01069660	-0.02965430	-0.00576522
H	13.62630350	18.05836440	11.66660060	-0.01279631	0.01177350	-0.00021631
O	12.69104870	18.02612550	12.05616370	-0.01238660	-0.02965430	-0.01258964
H	12.90504130	18.45659100	12.95638600	0.01069660	-0.02965430	-0.00576522
H	10.75677870	18.55235500	12.60265520	0.01069660	-0.02965430	-0.00576522
O	10.02947250	18.63681130	13.26161600	0.01069660	-0.02965430	-0.00576522
H	10.17821620	17.85202770	13.87277730	0.01069660	-0.02965430	-0.00576522
H	15.62407610	11.80790940	18.74656320	-0.01279631	0.01177350	-0.00021631
O	15.89184140	12.21253950	19.61506210	0.01069660	-0.02965430	-0.00576522
H	16.71308060	12.72765600	19.35114900	0.00269660	-0.02965430	-0.00576522
H	14.28594840	19.93321350	13.73214410	-0.02169660	-0.02965430	-0.00576522
O	13.48302040	19.48259130	14.06814870	0.01069660	-0.02965430	-0.00576522
H	13.75896360	19.08019110	14.98613500	0.01069660	-0.02965430	-0.00576522
H	13.40086120	17.79603330	19.42060760	-0.01279631	0.01177350	-0.00021631
O	12.39786880	17.45961170	19.21243910	0.01069660	-0.02965430	-0.00576522
H	12.62046730	16.34839840	18.76875130	0.01069660	-0.02965430	-0.00576522
H	15.35654330	17.30798330	19.48172570	-0.01069660	-0.02965430	-0.00576522
O	14.76245620	18.03916670	19.80142730	0.01069660	-0.02965430	-0.00576522
H	15.11300350	18.84230150	19.27109760	0.01069660	-0.02965430	-0.00576522
H	15.36602150	15.06990200	11.97308260	-0.01279631	0.01177350	-0.00021631
O	14.51318440	14.53701850	12.42334010	0.01069660	-0.02965430	-0.00576522
H	13.66686190	14.98320550	12.07939780	0.00869660	0.02965430	-0.01145522
H	12.92538750	12.54480200	17.77231300	0.00863260	-0.01965430	-0.01126987
O	12.21518440	12.19949220	17.18195970	0.00899660	-0.02965430	-0.00576522
H	10.83069270	14.96570170	12.47132660	0.01069660	-0.02965430	-0.00576522
O	10.09010560	14.62713580	13.07092670	-0.01279631	0.01177350	-0.00021631
H	10.08939800	15.35309720	13.76907550	0.01069660	-0.02965430	-0.00576522
H	12.04687160	15.58765420	10.72331040	0.01069660	-0.02965430	-0.00576522
O	12.21088200	15.59269820	11.69061130	0.01069660	-0.02965430	-0.00576522
H	12.37245140	16.62356590	11.92541670	0.01069660	-0.02965430	-0.00576522
H	17.77932460	16.29765860	12.24849890	0.01069660	-0.02965430	-0.00576522
O	16.43144900	15.80889460	11.47807610	-0.01279631	0.01177350	-0.00021631
H	16.58665180	15.55755810	10.54317410	0.01069660	-0.02965430	-0.00576522
H	17.83899920	19.16630100	14.72254580	0.01069660	-0.02965430	-0.00576522
O	16.53922630	18.03262610	14.08174070	0.01069660	-0.02965430	-0.00576522
H	16.47998840	17.88347830	15.68312590	0.01069660	-0.02965430	-0.00576522
H	17.22154950	14.57915300	18.49309620	0.01069660	-0.02965430	-0.00576522
O	17.86420740	13.81913130	18.58550290	-0.01279631	0.01177350	-0.00021631
H	17.97732930	13.45383910	17.66829200	0.01069660	-0.02965430	-0.00576522

H	17.69244280	16.54541510	18.61258700	0.01069660	-0.02965430	-0.00576522
O	19.14622980	16.44634450	18.61880160	0.01069660	-0.02965430	-0.00576522
H	19.18388460	15.45944450	18.65735560	0.01069660	-0.02965430	-0.00576522
H	15.79627420	17.07198710	11.48438810	0.01069660	-0.02965430	-0.00576522
O	15.30178160	18.03170370	11.66005100	-0.01279631	0.01177350	-0.00021631
H	15.92017450	18.12190890	13.29290520	0.01069660	-0.02965430	-0.00576522
H	14.54432660	10.70836850	16.72249620	0.01069660	-0.02965430	-0.00576522
O	15.36553370	11.39902910	17.02331940	0.01069660	-0.02965430	-0.00576522
H	16.19104550	11.00996680	16.65125250	0.01069660	-0.02965430	-0.00576522
H	14.87730750	19.70116590	17.54998100	0.01069660	-0.02965430	-0.00576522
O	15.60125080	19.95825250	18.18329390	-0.01279631	0.01177350	-0.00021631
H	16.32580530	19.41018650	17.78911260	0.01069660	-0.02965430	-0.00576522
H	10.70069510	13.46804590	13.65275490	0.01069660	-0.02965430	-0.00576522
O	11.25353400	12.61018090	14.02676930	-0.01279631	0.01177350	-0.00021631
H	10.59252440	11.95783920	14.34472210	0.00863260	-0.01965430	-0.01126987
H	11.11214980	17.79451000	16.17717640	0.00863260	-0.01965430	-0.01126987
O	10.61334800	16.62801520	15.00031490	0.00863260	-0.01965430	-0.01126987
H	9.99956210	16.28776690	15.66388160	0.00863260	-0.01965430	-0.01126987
H	11.16440880	19.37644820	16.21164210	0.00863260	-0.01965430	-0.01126987
O	11.38322830	18.53963100	16.80564020	-0.01279631	0.01177350	-0.00021631
H	12.00439470	17.99389270	18.47010330	0.00863260	-0.01965430	-0.01126987
H	19.36780160	16.66696950	17.64604830	0.00863260	-0.01965430	-0.01126987
O	19.56012600	16.99423850	16.05095460	0.00863260	-0.01965430	-0.01126987
H	19.04860210	16.34865920	15.51781240	0.00863260	-0.01965430	-0.01126987
H	19.32724690	19.47271980	14.45198060	0.00863260	-0.01965430	-0.01126987
O	18.68621450	19.40690000	15.19247260	-0.01279631	0.01177350	-0.00021631
H	19.14935250	17.89164200	15.81308110	0.01069660	-0.02965430	-0.00576522
H	12.76253220	10.64017520	16.54463280	0.01069660	-0.02965430	-0.00576522
O	13.46847550	10.00481440	16.23547030	0.01069660	-0.02965430	-0.00576522
H	13.66100700	10.28773450	15.27543140	0.01069660	-0.02965430	-0.00576522
H	16.55961910	12.79610710	12.03732450	0.01069660	-0.02965430	-0.00576522
O	17.08286250	12.78679400	12.86983420	-0.01279631	0.01177350	-0.00021631
H	16.47443160	13.28198350	13.53362880	0.01069660	-0.02965430	-0.00576522
H	12.40455210	12.21172940	12.65277500	0.01069660	-0.02965430	-0.00576522
O	13.21084640	12.04094090	12.08832330	0.01069660	-0.02965430	-0.00576522
H	13.69367850	12.91677610	12.12824740	0.01069660	-0.02965430	-0.00576522
H	11.93867750	20.38620720	14.69661130	0.01069660	-0.02965430	-0.00576522
O	11.01427490	20.41262330	15.05754880	-0.01279631	0.01177350	-0.00021631
H	10.53890860	19.84506000	14.36229080	0.01069660	-0.02965430	-0.00576522
H	14.22152090	17.70066640	16.46877690	-0.00058951	-0.00985350	-0.00021631
O	13.86858390	18.61769620	16.42656650	-0.01270001	0.01412350	-0.00021631
H	12.82695770	18.52917940	16.62197000	-0.00759631	0.01258350	-0.00021631
H	19.62847880	11.94995710	16.51952980	0.01069660	-0.02965430	-0.00576522
O	18.83660520	12.43965440	16.20909170	0.01069660	-0.02965430	-0.00576522
H	18.27190230	11.74936240	15.72078910	0.01069660	-0.02965430	-0.00576522

H	16.57604050	10.30588630	14.59202780	-0.01279631	0.01177350	-0.00021631
O	17.43873100	10.78337470	14.65137610	0.01469660	-0.02965430	-0.00576522
H	17.31818280	11.44040160	13.89700880	-0.01033258	-0.02965430	-0.01569875
H	18.93057800	16.10072550	13.32282930	0.01069660	-0.02965430	-0.00576522
O	18.45919930	16.80547160	12.82336470	0.02129660	-0.02965430	-0.01574692
H	17.32026960	17.49764360	13.67479770	0.01069660	-0.02965430	-0.00576522
H	13.42316930	14.78342140	19.14871240	-0.01279631	0.01177350	-0.00021631
O	12.86340050	15.22282340	18.39469680	0.00863260	-0.01965430	-0.01126987
H	11.37879250	14.64434950	17.97718480	0.00863260	-0.01965430	-0.01126987
C	16.40555760	16.53878740	17.11518930	0.00863260	-0.01965430	-0.01126987
O	16.64010700	16.38288490	18.47633120	-0.00856980	-0.02421530	-0.00325896
O	16.65123740	17.84374740	16.68482220	0.01069660	-0.02965430	-0.00576522
H	15.80469920	18.67985210	11.12298270	-0.01279631	0.01177350	-0.00021631
H	15.34168710	16.19243610	16.91398130	-0.02569660	-0.01465430	-0.01569875
H	17.05881160	15.80905640	16.51923480	-0.01279631	0.01387550	-0.01258631
H	16.43144900	16.60889460	11.47807610	0.00120569	-0.01458963	0.00985214

$Cu_6[H_2O]_{35}$ (with directly attached explicit water molecules)

*

111

Lattice="30.00023399524536 0.0 0.0 0.0 32.53004721085648 0.0 0.0 0.0 31.597066267600972"
Properties=species:S:1:pos:R:3:forces:R:3

Cu	17.06074347	18.05143159	15.96638846	-0.07320390	0.00506508	-0.01003367
Cu	14.72706092	18.48994796	16.40182312	0.01893995	0.02219509	-0.02040123
Cu	15.35300565	17.84750288	14.27755650	0.00641476	0.02069664	0.00434915
Cu	13.48271160	16.64287143	15.16412805	0.02935892	0.01963531	-0.02376167
Cu	15.54808012	16.18680639	16.32446667	-0.00625414	0.00819481	-0.01260555
Cu	13.27674916	19.02618957	14.51913870	0.04269827	-0.00026582	0.09633713
O	18.91892089	17.08701367	15.59352791	-0.02653281	0.01155181	-0.00897981
O	13.78683091	19.20898869	18.12705750	0.00323114	-0.01634494	-0.01816534
O	11.58792676	19.41935178	13.18525108	0.02283982	-0.00121667	0.01569100
O	11.82720306	16.84643579	17.51956572	0.01151283	0.01501086	-0.00498410
O	16.20399798	14.43303479	17.16307109	-0.01329470	0.02066416	-0.02035070
O	15.80081811	16.47849637	12.77477994	-0.00552256	0.00214691	0.01827404
H	19.43318677	17.72662749	15.04611066	0.00122952	-0.00523357	-0.00167732
H	19.29713364	17.11979905	16.62806088	-0.00114506	-0.00715095	-0.00068033
H	14.59109519	19.36881396	18.71745687	-0.00901340	-0.00654084	-0.00066865
H	13.47540012	20.11559654	17.79782300	-0.00173792	-0.00538845	0.00056893
H	11.20798744	20.32524181	13.21558209	0.01403110	-0.00704427	0.00869285
H	10.89535889	18.80969809	13.72962975	0.00185896	-0.00905478	-0.00048820
H	11.52909088	17.77722577	17.27043215	0.03604094	-0.00578610	-0.02309464
H	12.71590225	16.81771557	17.00290381	0.01116386	0.00776813	-0.00131010
H	15.34843334	13.91263473	17.19671911	0.00678775	-0.00788328	-0.00451893
H	16.92688240	13.80180256	16.72024393	-0.01316747	0.01496905	-0.01177371

H	15.75230889	15.70327993	13.38467811	-0.01061652	0.00949484	0.01923441
H	16.81080298	16.62790226	12.59168627	-0.00599044	-0.00747814	0.01096004
H	17.39522582	12.20100218	15.73903433	-0.00266019	0.01845380	0.00074442
O	17.98241796	12.91083510	16.16526281	-0.00574290	0.01586334	-0.00739323
H	18.29708045	13.45009082	15.39414411	-0.01378243	0.00563077	0.00434687
H	17.22501510	15.14050037	18.39520804	-0.01158491	0.01861558	-0.01563511
O	17.74640620	15.65216228	19.07884497	-0.01920074	0.01156323	-0.01420143
H	17.01002517	16.13753246	19.53764037	-0.00459616	0.00917309	-0.02806482
H	13.22706654	12.78472888	17.12213357	0.00682485	0.01769649	-0.00925696
O	13.61686712	13.68469909	17.07695877	-0.00423034	0.01505687	-0.01281764
H	13.40294239	13.99553051	16.14135979	0.01161618	0.02965569	0.00022072
H	14.14796485	20.32118024	13.21083728	0.00846682	-0.02197312	0.00999202
O	14.28099347	21.24218909	12.76210820	0.00091104	-0.01984356	0.00285109
H	13.81479725	21.15992229	11.90033338	0.00442829	-0.02308985	0.01809310
H	16.10851070	21.02638699	12.97635633	-0.00042693	-0.01847942	0.02211983
O	16.80400322	20.36197548	13.23756618	-0.00804951	-0.00534779	0.01590576
H	16.31029151	19.79052240	13.92843180	-0.01105656	-0.00092524	0.01848154
H	18.58100559	15.13511856	13.31683079	-0.01524393	0.00848582	0.01265115
O	18.15328912	14.88719480	14.16759319	-0.02451214	0.01901861	0.00183788
H	18.34619961	15.69542852	14.75178912	-0.03768616	0.01653600	0.00868601
H	18.79815016	20.28298251	19.03756224	-0.01370236	-0.00571588	-0.00793768
O	18.33421250	19.64224221	18.45631890	-0.01764453	-0.00529097	-0.00677106
H	17.45271973	19.45940949	18.92069759	-0.00870238	-0.01352598	-0.02732413
H	11.02884637	17.98767010	19.61239522	0.01287660	-0.00368181	-0.00895840
O	11.32407983	18.92385667	19.58375937	0.02139331	-0.00697072	-0.01552152
H	12.24573295	18.84473616	19.21138382	0.01291067	-0.00880412	-0.01116482
H	14.18447002	13.82394615	14.22996754	0.00636293	0.03201999	0.01246529
O	13.19570644	13.91363223	14.38415364	0.02372286	0.00809144	-0.00731618
H	12.93505093	14.48823555	13.60530212	0.01945580	0.00825747	0.00712805
H	13.94195243	22.12174832	14.25923584	0.00461972	-0.02378381	0.01232671
O	14.02609783	22.57771530	15.14298771	0.00791812	-0.02403608	-0.00078211
H	15.04688414	22.54343771	15.32191399	-0.00572111	-0.02547764	-0.00252444
H	11.02223751	15.87040708	16.26505830	0.01830541	0.00153993	-0.00458553
O	10.64468962	15.50762686	15.40757356	0.01810406	0.00190848	0.00078802
H	11.40448908	14.99405952	15.04597808	-0.00274386	0.00838225	0.00597259
H	10.78112135	19.38705004	17.96511383	0.01457490	-0.00075822	-0.01913367
O	10.83215851	19.39662633	16.95830869	0.02373916	-0.00473240	-0.00979548
H	11.56235348	20.05151488	16.77399383	0.02072823	-0.02539633	-0.01906362
H	15.81116997	19.84250982	20.40122122	-0.00295060	-0.00478825	-0.01334618
O	15.97220318	19.22831123	19.65427514	-0.00120689	-0.00749251	-0.01851943
H	15.74285925	18.27292375	19.97989066	0.00066584	0.00115605	-0.02869046
H	18.86237653	16.58786372	18.41746097	-0.01519093	0.01245348	-0.01842094
O	19.55419097	17.22219786	17.99270526	-0.01209452	0.00649027	0.00023529
H	19.17537427	18.11984356	18.21744849	-0.01913490	-0.00773799	-0.01814946
H	17.59284242	17.70141401	11.38995734	-0.00452291	-0.00311275	0.02393584

O	18.16335503	17.07595084	11.95813405	-0.00857636	0.00648750	0.02274929
H	18.59237843	17.73079082	12.57679248	-0.02419581	-0.00623172	0.01914501
H	16.46470398	19.38469314	11.76544525	-0.01361561	-0.01696261	0.01924282
O	16.39042143	18.82763217	10.93482930	-0.01421194	-0.00084952	0.03001762
H	15.51261160	18.39130835	11.05176097	0.00261148	0.00013712	0.02866898
H	15.33794830	16.50146968	21.16097629	-0.00241984	0.00097216	-0.01199118
O	15.41095267	16.78921211	20.22682366	-0.00268417	0.00547614	-0.02346473
H	14.62459875	16.27861281	19.74111705	0.00613202	0.01179054	-0.02892747
H	14.68052886	21.74822929	17.82836499	0.01197401	-0.02033386	-0.00451487
O	15.60944603	21.65856920	18.16578244	-0.00512957	-0.02706932	-0.01171424
H	15.89795978	20.83095122	17.70548485	-0.00299198	-0.02461696	-0.02461620
H	12.96030056	16.30161257	11.89437100	0.01595486	0.00640558	0.03168715
O	12.76841554	15.29354010	12.02583407	0.01142744	0.01100088	0.02160036
H	11.92835784	15.12846013	11.54747687	0.00899158	0.00895230	0.01302278
H	14.14266019	14.47649326	11.38193662	0.00926015	0.00789732	0.01476555
O	15.03644834	14.08425651	11.13059878	-0.00192722	0.01785770	0.01945812
H	15.55684835	14.90679479	10.98558176	-0.00257091	0.01232083	0.01326358
H	12.77487303	16.11138867	18.81938843	0.01561582	0.01272654	-0.02290118
O	13.42716830	15.49082720	19.25450209	0.00598527	0.01180343	-0.02587979
H	13.57639517	14.83027105	18.52129070	0.00961253	0.02170961	-0.01970839
H	17.27080221	21.94206271	15.62825620	-0.01661208	-0.01791582	-0.00276970
O	16.56580143	22.64335473	15.77032968	-0.00463838	-0.02946102	-0.00422700
H	16.38339593	22.54142466	16.74358668	0.00106458	-0.01297823	-0.00910345
H	18.32027098	19.77367792	13.35723187	-0.01745532	-0.01489681	0.01348197
O	19.14919961	19.22454798	13.56500137	-0.01868376	-0.00546440	-0.00145908
H	19.87629008	19.70827563	13.11735567	-0.01606788	-0.00570884	0.00667961
H	13.86130582	17.92972421	12.59581293	-0.00231946	-0.00993183	-0.00656489
O	13.39281764	17.83161999	11.69515438	0.00669173	-0.00137006	0.00835170
H	12.59801333	18.40246863	11.88340061	0.01739162	-0.00516380	0.01862836
H	13.37084010	21.88907256	16.11743625	0.00839204	-0.03046661	-0.00764700
O	12.86901977	21.41301126	16.99652787	0.00421043	-0.01818588	-0.00233368
H	12.34592289	22.12477168	17.42377508	0.00841301	-0.01198061	-0.00518671
H	18.82156686	20.16837093	15.27539291	-0.02037964	-0.01315175	0.01408885
O	18.69717130	20.85709680	15.97273058	-0.01349881	-0.01841483	-0.00229351
H	18.45000614	20.33823727	16.78525685	-0.01609911	-0.01605211	-0.01315427
H	10.32752357	17.09590374	14.76619979	0.03016152	0.01141859	0.00282958
O	10.03244620	18.05098802	14.60954876	0.01746302	-0.00632499	0.01330758
H	10.20749350	18.49311451	15.48589106	0.02626523	-0.00829066	-0.00400396
H	16.57434202	10.57884048	14.48079981	-0.00121304	0.01056381	-0.00091842
O	16.18626353	11.30707253	15.00860554	-0.00956705	0.02072115	0.00276254
H	15.97798144	12.02278878	14.32995299	-0.00548407	0.01295585	0.00741591
H	16.64012901	14.02537423	13.80093771	-0.01919879	0.01953680	0.01330111
O	15.79576080	13.52662605	13.58667929	0.00417466	0.01843264	0.01967712
H	15.59186901	13.67332878	12.58655344	0.00288203	0.00956667	0.01521267

$^*CO_2$

114

Lattice="29.843843899999996 0.0 0.0 0.0 32.0645142 0.0 0.0 0.0 30.226147"

Properties=species:S:1:pos:R:3:forces:R:3

Cu	16.96200476	17.26206254	15.02396490	-0.04300041	0.02641255	-0.01530326
Cu	14.70084567	17.76803887	15.83049382	-0.01270579	-0.03570130	0.00140037
Cu	15.54393464	18.89517532	13.86199841	0.01390657	-0.03553358	0.06687522
Cu	14.81132803	16.46050711	13.95195871	0.00162499	-0.00267344	0.01496147
Cu	15.69821278	15.52161470	16.02318109	-0.00978512	0.06918658	-0.02559718
Cu	13.22344033	18.34885325	13.71361187	0.04254863	0.00829116	0.09288019
O	18.88243847	16.61308440	14.70264558	-0.02748558	0.01234500	-0.00794817
O	13.71728995	18.66261760	17.39322805	0.00555125	-0.01702797	-0.02258160
O	11.54357596	18.64108154	12.56828418	0.02514950	-0.00070491	0.01618275
O	11.50246690	16.24459563	16.90606630	0.01848202	0.02076912	-0.01688178
O	16.17145445	13.65266711	16.52412188	-0.01194903	0.02181753	-0.01544567
O	15.50049622	15.69639528	12.14129836	-0.00437112	0.00101142	0.01346158
H	19.40607867	17.26710331	14.18552252	0.00210499	-0.00462988	0.00093618
H	19.24992050	16.58734761	15.75475868	-0.00152423	-0.00429509	0.00142256
H	14.50680736	18.84778542	18.00891364	-0.00514626	-0.00003955	-0.00261455
H	13.46060275	19.53744209	16.97034786	-0.00048222	-0.00222543	-0.00496364
H	11.25714447	19.57642385	12.48317334	0.00391353	-0.00019202	0.00215367
H	10.69087023	18.13214255	13.12807805	0.00283241	-0.01130946	0.00350631
H	11.49269992	17.16160590	16.52400777	0.03099779	-0.00803705	-0.02747113
H	11.94353953	15.71299913	16.14392947	0.02833172	0.02483288	-0.00527298
H	15.26230113	13.18882321	16.38019057	0.00529565	-0.00756071	-0.00892479
H	16.87738789	13.18688210	15.90785915	-0.00600646	0.00204740	-0.01241928
H	15.55832426	14.72648085	12.39909483	-0.00027929	0.00153774	0.00052828
H	16.45118871	15.97322919	11.92898109	-0.01044766	0.00631617	0.01563387
H	17.37669361	11.71488948	14.74169994	-0.00319400	0.01871736	0.00064210
O	17.96809463	12.43160715	15.14756503	-0.00703094	0.01869972	-0.00747175
H	18.26210571	12.97544273	14.37657092	-0.01408501	0.00552951	0.00689433
H	17.35378582	14.32894520	17.73564675	0.00026643	0.00836098	-0.00002402
O	17.82046701	14.99055824	18.31649796	-0.01197512	0.00865121	-0.00746920
H	17.03982202	15.49802140	18.67386138	-0.00777214	0.00981063	-0.03125404
H	13.50227149	12.13241258	15.76296879	0.00889252	0.01680015	-0.01124407
O	13.71229656	12.94969107	16.26758001	0.00052346	0.00578987	-0.01501604
H	13.32426045	13.69180706	15.65549490	0.01653822	0.02170270	-0.00929594
H	14.16144538	19.79939892	12.33445982	-0.00282170	-0.00355549	-0.00800950
O	14.26861777	20.63204110	11.72761060	-0.00437060	-0.01321160	-0.00315244
H	13.89922653	20.34735969	10.86122081	0.00353119	-0.02091238	0.02375741
H	16.05850891	20.58426924	11.96966477	0.00765428	0.00415646	0.00250358
O	16.81554184	20.32023656	12.57009931	-0.01131225	-0.01529706	0.01560730
H	16.81031977	21.01213809	13.33353921	0.00411036	-0.00181375	-0.00178753
H	18.44577024	14.86135082	12.24030235	-0.01137489	0.00254954	0.01699546
O	18.33534028	14.39909250	13.10604845	-0.02293378	0.01797204	0.00545000

H	18.44538675	15.15268438	13.76195372	-0.03482243	0.01149618	0.00830323
H	18.63881276	19.68922592	18.05067068	-0.01485817	-0.00610309	-0.00918186
O	18.16453935	19.02734468	17.50222283	-0.02151533	-0.00662713	-0.01167017
H	17.28846239	18.88154211	17.98897227	-0.01137937	-0.01599851	-0.03188930
H	10.94346455	17.70097910	18.72420433	0.01046200	-0.00035985	-0.00515486
O	11.27783660	18.62578208	18.69645583	0.01744391	-0.00723671	-0.01375992
H	12.20925194	18.49065565	18.36527844	0.01134169	-0.00909231	-0.01257196
H	14.07271463	12.83063275	13.13686199	-0.00229721	0.02713688	0.01181756
O	13.12286317	12.57555365	13.14128871	0.01975892	0.02858199	0.00452158
H	12.72494204	13.32039048	12.63355379	0.01632979	0.01450150	0.01508825
H	13.96181562	21.63927124	13.19403696	0.01013181	-0.02545775	0.01284760
O	14.08260041	22.07411484	14.08373755	0.01577596	-0.02356198	0.00059493
H	15.06774707	22.07835669	14.19551217	-0.00672658	-0.02640580	-0.00121265
H	9.92822571	15.42071726	15.65207628	0.00734192	0.00368554	-0.00821559
O	9.73753318	15.21930353	14.70323136	0.02467725	0.00742948	0.00373271
H	10.64348363	14.91304272	14.41463162	-0.00006373	0.00273962	-0.00015675
H	10.77104673	18.98727650	17.08974090	0.01332056	-0.00128438	-0.01830034
O	10.73138960	18.99703197	16.07949866	0.02368856	-0.00563868	-0.01074156
H	11.49265673	19.57431557	15.82079958	0.01848446	-0.02689978	-0.01677847
H	15.84113108	19.18367850	19.71556496	-0.00227450	-0.00566202	-0.01506410
O	15.87476320	18.67045668	18.88066384	0.00006981	-0.00376719	-0.01850060
H	15.72426760	17.66892345	19.13628252	0.00004560	-0.00220488	-0.03146575
H	18.85678428	15.93737966	17.53289178	-0.01911116	0.01190553	-0.01916447
O	19.49486447	16.62087748	17.09885725	-0.01400752	0.00767021	-0.00022777
H	19.06158867	17.48869286	17.34191322	-0.02170806	-0.00896398	-0.01871717
H	17.23646117	17.04048678	10.59182430	-0.00464668	-0.00447675	0.02249322
O	17.86411329	16.48860933	11.15298490	-0.01379979	0.00672529	0.01819016
H	18.31422377	17.19999537	11.69149515	-0.02261804	-0.00342565	0.02166015
H	15.89008478	18.47706089	11.30537216	-0.00904487	-0.01977085	0.01161990
O	15.91591171	18.24013233	10.34514317	-0.00890003	0.00183139	0.03171093
H	14.99978489	17.84958214	10.25033138	0.00310304	0.00041820	0.03033816
H	15.41860456	15.87226088	20.22620687	-0.00271111	0.00114358	-0.01478176
O	15.51072130	16.19062050	19.30238130	-0.00068299	0.00587097	-0.01853765
H	14.69350627	15.73138173	18.79556095	0.00896770	0.01141239	-0.03281939
H	14.75814708	21.06450527	16.66228194	0.01193082	-0.02751514	-0.01246362
O	15.72484467	20.90709309	16.83241325	-0.00285168	-0.02752070	-0.00756410
H	15.84493035	19.99995402	16.43642368	0.00988327	-0.00461221	-0.01672666
H	12.78627962	15.40045245	10.74024575	0.01309595	-0.00083595	0.01960564
O	12.73794346	14.40169480	10.71757106	0.01433939	0.01746093	0.01850747
H	11.87103904	14.20574619	10.30724839	0.01009837	0.00767750	0.01367694
H	14.20358405	13.71061198	10.29015887	0.00631678	0.00717745	0.01579912
O	15.14856336	13.39338150	10.10533495	-0.00297612	0.01584784	0.01936899
H	15.58539855	14.21971602	9.80742092	-0.00286648	0.01462959	0.01717729
H	12.80479338	15.61219603	17.83073441	0.02087633	0.01325158	-0.02211803
O	13.49813446	15.04208623	18.29986467	0.00058205	0.00954798	-0.02102998

H	13.57584385	14.22914646	17.73495765	0.01259792	0.02278048	-0.01346574
H	17.80096105	21.38186106	14.81339861	-0.01975784	-0.01565183	-0.00336135
O	16.94799652	21.92143868	14.66882239	-0.00859774	-0.01627248	0.00134506
H	16.45485402	21.64339798	15.50914685	-0.00372500	-0.02390196	-0.00955986
H	18.13326779	19.29849096	12.43458621	-0.02487266	-0.01534217	0.01768650
O	18.92424929	18.67341745	12.55182263	-0.02391328	-0.00365712	0.00272868
H	19.64116844	19.10093449	12.03502555	-0.01601162	-0.00314470	0.00983230
H	13.78276895	16.80954297	11.55268918	0.00426681	0.00755595	-0.00770087
O	13.33177547	17.18863007	10.75051829	0.01170341	-0.00103217	0.01695194
H	12.58524579	17.67736770	11.19719670	0.01753675	-0.00693048	0.00647513
H	13.38490372	21.30188965	15.20172906	0.00928657	-0.03712409	-0.00546726
O	12.96036456	20.90323710	16.07482138	0.00790536	-0.01895800	-0.00452360
H	12.44994303	21.64333624	16.46963195	0.00800431	-0.01436949	-0.00551380
H	18.86267797	19.53397788	14.33958009	-0.03018784	-0.01512453	0.01520990
O	18.90886093	20.16619469	15.10017269	-0.01802004	-0.02342746	-0.00333487
H	18.48444095	19.66606231	15.85391438	-0.02724395	-0.02490066	-0.01986846
H	9.73934310	16.69898811	14.12022274	0.02422740	0.00879913	0.00258823
O	9.68431182	17.70703881	13.89487955	0.01974970	-0.00533095	0.01186164
H	9.92594534	18.14787300	14.75893804	0.02216259	-0.01231906	-0.00245643
H	16.38041682	10.05960941	13.61293821	-0.00216653	0.01182789	-0.00109804
O	16.10790705	10.85074892	14.12134537	-0.01245510	0.02164495	0.00324033
H	15.93292697	11.54619844	13.41752506	-0.00433977	0.01037452	0.00868917
H	16.85543691	13.45625191	12.72977412	-0.01919808	0.01580834	0.00947877
O	15.95241504	13.05539865	12.55637138	0.00190420	0.01935882	0.02001983
H	15.76328684	13.08041135	11.54234940	0.00162622	0.00997380	0.01692509
C	13.01435368	15.65829176	14.06509081	-0.00090618	-0.00484953	-0.01967364
O	12.26697805	16.00242528	13.10075398	-0.00240938	-0.00559971	0.00952023
O	12.56281623	14.86047707	15.00225882	0.01242772	0.00650520	-0.00641234

*HCOO;*OH

114

Lattice="29.843843899999996 0.0 0.0 0.0 32.0645142 0.0 0.0 0.0 30.226147" Properties=species:S:1:pos:R:3:forces:R:3:initial_charges:R:1

Cu	17.17135546	16.76895891	15.15982010	-0.07709681	-0.01673320	-0.03232175	0.35680000
Cu	14.85824564	17.64014358	15.71082033	0.01235869	0.00074295	0.01069906	0.17770000
Cu	16.01314048	19.04572875	14.09052803	-0.05646330	-0.00418679	0.02297262	0.13270000
Cu	15.37239677	16.71174753	13.61610989	0.00795204	0.02247405	0.01103256	0.23890000
Cu	15.36879586	15.30300228	15.67240019	0.00744447	0.04170750	-0.01893858	0.30780000
Cu	13.79592428	18.62404129	13.55062237	0.06078967	0.01539697	0.04614896	0.11420000
O	18.49897244	16.33231266	13.63866871	-0.01484242	0.01452556	0.01337728	-1.16670000
O	13.76518532	18.58933245	17.15062205	0.00362868	-0.01920720	-0.02248076	-1.22470000
O	11.93975635	18.44938184	12.70780302	0.02719801	-0.00160754	0.01479827	-1.19160000
O	11.16598283	16.20408434	17.06917248	0.01280546	0.01582444	-0.02057046	-1.22290000
O	16.10823856	13.64511838	16.50888276	-0.01549065	0.02085075	-0.01655788	-1.23120000
O	15.48071986	15.65309270	11.94622285	-0.01164120	-0.00138485	0.02140020	-1.22060000

H	19.39648411	16.47367060	14.01223563	-0.00184495	0.00515328	-0.00673578	0.55330000
H	14.48724122	18.73527273	17.84855894	-0.00282490	0.00450197	-0.00566410	0.62420000
H	13.53313706	19.49010747	16.74532377	0.00417181	-0.00156451	-0.00778555	0.62750000
H	11.63473953	19.29316466	12.30794005	-0.00173070	-0.00077690	0.00149596	0.57780000
H	11.04429535	18.02930872	13.21556662	0.02261678	-0.00610680	0.00644307	0.63060000
H	11.07919927	17.12883852	16.71497495	0.02341662	-0.00791114	-0.01689642	0.57820000
H	11.95667553	15.86239263	16.49550309	0.02102566	0.01727632	-0.00219421	0.61080000
H	15.28885771	13.03906462	16.52436484	0.00921358	-0.00576494	-0.00363957	0.61570000
H	16.88181810	13.13598893	15.95786708	-0.00639129	0.00757101	-0.01247553	0.62500000
H	15.49786027	14.68900114	12.24017827	-0.00481876	-0.00244850	0.00586454	0.61660000
H	16.46624322	15.89027458	11.57612424	-0.00142277	-0.00810986	0.00241034	0.62540000
H	17.38713576	11.71105325	14.87821606	-0.00508666	0.02066616	0.00084689	0.60410000
O	17.94826254	12.43425023	15.30274469	-0.00805363	0.01553712	-0.00775475	-1.21540000
H	18.16106535	13.02606594	14.50811742	-0.01963150	0.01429445	0.00912806	0.61220000
H	17.26281898	14.38667362	17.65623788	-0.01536086	0.01681768	-0.01663773	0.61310000
O	17.75482952	15.01822465	18.25851943	-0.02017946	0.00716811	-0.00968953	-1.20550000
H	16.97283836	15.45052463	18.69272493	-0.00691286	0.00839024	-0.02978830	0.59390000
H	13.60158240	11.66504189	16.57381469	0.00621658	0.01466482	-0.00902943	0.56190000
O	13.72136692	12.62038811	16.39581901	-0.00036273	0.00902156	-0.00689827	-1.19380000
H	13.57537919	12.71996917	15.35404501	0.01249589	0.02402754	0.00048300	0.64000000
H	14.54086414	20.04611711	12.25813712	-0.00558742	-0.00221396	-0.00727909	0.48250000
O	14.51138971	20.77795386	11.53866367	0.00068242	-0.01317977	0.00509391	-1.17470000
H	14.64690481	20.22033944	10.72931093	-0.00846510	-0.00413513	0.02358046	0.59460000
H	16.21230240	20.95411278	11.71417669	0.00004803	-0.01905105	0.02778326	0.60660000
O	17.18647068	21.07605841	11.94492656	-0.01344999	-0.00875739	0.01237992	-1.18390000
H	17.15586125	21.75243196	12.65547285	-0.01410426	-0.02018252	0.00418719	0.57670000
H	18.88232852	13.72989297	12.47947127	-0.01459502	0.01148182	0.00312704	0.55320000
O	18.17768329	13.96106218	13.12000204	-0.02270952	0.00343863	0.00084290	-1.19360000
H	18.36312139	15.08298101	13.39837844	-0.02659863	0.01028642	0.00634564	0.62170000
H	18.39879244	19.87088905	18.07098343	-0.01534669	-0.00675778	-0.01225196	0.58250000
O	17.89650844	19.26639160	17.48420159	-0.01663460	-0.00755346	-0.00940389	-1.19440000
H	17.14684150	18.89881578	18.05965197	-0.01218807	-0.01474538	-0.03031039	0.62880000
H	11.13608479	17.72182624	18.76096479	0.01212650	0.00216203	-0.00182059	0.58350000
O	11.47852656	18.64315815	18.69542393	0.01776729	-0.00692031	-0.01279308	-1.17910000
H	12.35239772	18.48881765	18.23683420	0.01147461	-0.00984248	-0.01266290	0.60030000
H	14.34113648	12.80611876	13.42843514	0.00612518	0.02794366	0.01199801	0.60190000
O	13.44109621	12.77652368	13.85131441	0.01934856	0.01984396	-0.00572030	-1.21700000
H	13.09098800	13.70097145	13.64036553	0.02330289	0.01312262	-0.00170404	0.61280000
H	14.00043376	21.70700616	13.19747318	0.01161386	-0.02505953	0.01442726	0.60070000
O	14.06425978	22.10665438	14.09736037	0.01384482	-0.02231460	-0.00512640	-1.20730000
H	15.06941586	22.11218174	14.30974714	-0.00012282	-0.03185920	-0.00494071	0.61960000
H	10.12213059	15.43801156	15.80401698	0.01135955	0.00234459	-0.00543323	0.60580000
O	9.68988263	15.22460871	14.92134140	0.02335994	0.00670714	0.00189938	-1.16730000
H	10.32284527	14.59478600	14.51117840	0.01590049	0.00514493	0.00233785	0.57990000
H	10.82124203	19.00795617	17.12505224	0.01173803	-0.00277677	-0.01744930	0.59770000

O	10.72437503	18.96679882	16.11870031	0.02798207	-0.00977366	-0.01088357	-1.20550000
H	11.51474692	19.47123422	15.79995992	0.01714401	-0.02621087	-0.01261424	0.59030000
H	15.58689552	18.95681338	19.72012113	-0.00256789	-0.00414984	-0.01498212	0.58780000
O	15.74355850	18.47097589	18.88264301	0.00081944	-0.00396443	-0.01939681	-1.20280000
H	15.54739950	17.46340454	19.08578702	0.00182457	0.00028997	-0.03108890	0.64020000
H	18.39282838	16.08748549	17.24248424	-0.00859739	0.00238334	0.00173905	0.62210000
O	18.73489325	16.79636212	16.57096873	-0.02306031	0.00548610	-0.01712140	-1.21800000
H	18.61375697	17.64827565	17.06337091	-0.00706254	-0.00301024	0.00275371	0.59630000
H	17.34279947	17.29301386	10.80647168	-0.00646157	-0.00306875	0.03018609	0.61110000
O	17.72540091	16.43047526	11.12570246	-0.01042304	-0.00094683	0.01342130	-1.22250000
H	18.14329740	16.62489335	12.03633715	-0.02979303	0.01283162	0.02069916	0.62580000
H	15.83279398	18.52187635	11.56160955	-0.00157544	-0.01466515	0.00659103	0.52370000
O	15.88217937	18.48295697	10.56377197	0.00201903	-0.00809555	0.02829458	-1.17830000
H	15.04541341	17.95584059	10.38773454	0.00508792	-0.00372281	0.03147322	0.60470000
H	15.22033417	15.68971498	20.20745307	-0.00242994	0.00075012	-0.01333295	0.57830000
O	15.30409079	15.98950552	19.27696910	-0.00172020	0.00448518	-0.02543468	-1.20020000
H	14.54099122	15.45055224	18.77966875	0.00940671	0.01082795	-0.03297701	0.64230000
H	14.69382437	21.09391476	16.82713894	0.01490397	-0.02805729	-0.01140369	0.58960000
O	15.56193573	21.27455989	17.28085685	-0.00436305	-0.03065237	-0.01245619	-1.17770000
H	16.10068083	20.47438389	17.07471312	-0.01885851	-0.02023617	-0.02748670	0.57180000
H	12.84960149	15.39212552	10.24548345	0.00922074	-0.00112878	0.01407308	0.59490000
O	12.70859816	14.40701077	10.33301230	0.01303972	0.00932228	0.02558586	-1.16050000
H	12.50970151	14.34408063	11.29588390	0.01565088	0.00530996	0.00737104	0.58590000
H	14.21319694	13.74504791	10.09862924	0.00828617	0.00713994	0.01328456	0.61890000
O	15.14999554	13.35564891	9.96718015	0.00072330	0.01766447	0.01864125	-1.17330000
H	15.67283069	14.13723557	9.68906660	-0.00316702	0.01451525	0.01675892	0.56300000
H	12.60541434	15.19061676	18.07652753	0.01873547	0.00811339	-0.01792029	0.59760000
O	13.36269351	14.61332467	18.33997081	0.00182121	0.01040827	-0.02344699	-1.20530000
H	13.45509772	13.95833087	17.58165902	0.00963595	0.02000676	-0.01657218	0.63550000
H	17.09599557	21.41220302	14.79162878	-0.00804714	-0.00662518	0.00314220	0.60550000
O	16.56626661	22.25207627	14.90371428	-0.00458354	-0.01967444	0.00076450	-1.22090000
H	16.26752863	22.14382594	15.85640644	-0.00067570	-0.01836465	-0.01458774	0.60140000
H	18.37088776	19.92423987	12.15441797	-0.02046700	-0.02043363	0.01240374	0.62620000
O	19.05105347	19.27843263	12.54673689	-0.01948840	-0.01983944	0.00090019	-1.17050000
H	19.91672020	19.69789378	12.35687725	-0.01499391	-0.00486361	0.00419022	0.57310000
H	14.10402219	16.46075685	11.27395175	-0.00274576	0.00559426	0.01704024	0.61060000
O	13.51142055	17.02482770	10.69070191	0.00808212	0.00055679	0.02068478	-1.21680000
H	12.87598574	17.43499965	11.33841230	0.01716622	-0.01244762	0.00974045	0.60550000
H	13.45409945	21.32357037	15.10317030	0.01392693	-0.03425315	-0.00699222	0.63510000
O	13.04017639	20.82703410	15.97598587	0.00993820	-0.01516799	-0.00101335	-1.21940000
H	12.49415133	21.50379829	16.43236785	0.00772954	-0.01187114	-0.00516970	0.58140000
H	18.49638298	19.51902251	13.96572179	-0.00176786	-0.01535103	0.00968955	0.63590000
O	17.81686978	19.81175392	14.70435887	-0.02182710	-0.00562491	0.00347356	-1.22160000
H	18.05404365	19.41842112	15.58506353	-0.00364745	-0.01102979	0.00029405	0.61860000
H	9.83303412	16.67622787	14.17141387	0.01876002	0.00793430	0.00311219	0.62390000

O	9.89443034	17.65753838	13.88416014	0.01918907	-0.00801680	0.01259690	-1.21020000
H	10.00464334	18.13871053	14.75280693	0.01641480	-0.01128967	-0.00097096	0.61210000
H	16.48001647	10.05646350	13.62733647	-0.00237388	0.01317745	-0.00071871	0.56220000
O	16.13402480	10.83625292	14.10823444	-0.01207038	0.02474941	0.00497660	-1.16210000
H	15.99307712	11.52017555	13.38276087	-0.00282606	0.01242803	0.00905799	0.60510000
H	16.81668040	13.37884537	12.64893568	-0.01858913	0.02240359	0.01125854	0.62590000
O	15.86471518	13.00422415	12.47819398	-0.00194665	0.01935952	0.01810777	-1.25680000
H	15.68075948	13.00511914	11.46556000	0.00066709	0.01084850	0.01868488	0.61300000
C	12.63087863	15.79305737	14.39445668	-0.01919528	-0.00814793	-0.00656864	1.46100000
O	12.62097505	15.25818073	13.24934660	0.00455557	0.01686830	0.00120017	-1.14850000
O	12.94358189	15.21519445	15.50387141	-0.00034260	-0.01045417	0.00132095	-1.14610000
H	12.31071021	16.86657968	14.47879793	0.01286850	-0.00025362	0.02147760	0.04610000

*CO$_2$;*OH

115

Lattice="29.843843899999996 0.0 0.0 0.0 32.0645142 0.0 0.0 0.0 30.226147" Properties=species:S:1:pos:R:3:forces:R:3

Cu	16.77614836	16.81566741	14.94454118	-0.05391775	0.04405285	-0.02011239
Cu	14.70069458	17.75488145	15.79489818	-0.01618106	-0.04081458	0.00001842
Cu	15.63932803	18.57265711	13.63586691	0.01365094	-0.04734551	0.06391488
Cu	14.56262288	16.46037531	13.78086090	0.00077742	-0.01083598	0.00620378
Cu	15.18915030	15.32548730	15.87863048	0.01198459	0.05858875	-0.03927095
Cu	13.24044502	18.51007111	13.92763886	0.04087024	-0.01405519	0.08074911
O	18.79401346	16.61900061	14.67490695	-0.02677691	0.01290337	-0.00578810
O	13.70427588	18.65177851	17.50935182	0.00420522	-0.01581649	-0.02383773
O	11.59610714	18.60095676	12.70935204	0.02586874	-0.00626374	0.01860622
O	11.51456320	16.24426100	16.74499362	0.01349993	0.01968426	-0.01261874
O	15.76390589	13.43286232	16.44067304	-0.00564279	0.02156885	-0.02030630
O	15.94871381	15.62705858	11.09662052	-0.00734261	0.00011438	0.02522279
H	19.14884951	17.38267086	14.15703518	-0.00098566	0.00216969	-0.00106275
H	14.52404627	18.83624499	18.08241608	-0.00753284	-0.00138235	-0.00206713
H	13.45978210	19.51799000	17.08171956	-0.00454437	-0.00286811	-0.00173963
H	11.32417513	19.50259071	12.42973744	0.00412114	0.00109542	0.00090191
H	10.67623312	18.13874747	13.22719962	0.01061083	-0.01212272	0.00564185
H	11.50621560	17.18657010	16.40848717	0.03498858	-0.00229873	-0.02160274
H	12.15610868	15.76901454	16.10562005	0.02642908	0.02710712	-0.00824994
H	14.84321661	12.83578455	16.43782349	0.00375136	-0.00423329	-0.01074152
H	17.33268194	12.88056818	15.59430746	-0.01185067	0.02295319	-0.01151975
H	15.58405829	15.79379984	12.02142377	0.00711168	0.00891206	0.01790804
H	16.92034268	15.89071060	11.19186715	-0.02144946	0.00462651	0.02933489
H	17.25899054	11.26558097	14.50990836	-0.01089047	0.00867542	0.00051888
O	18.11999438	12.46039499	15.14740330	-0.01410228	0.02300820	-0.00051902
H	18.28410241	13.07441356	14.38079187	-0.01689097	0.01045349	0.00655064
H	16.93551926	15.56450811	17.09622595	-0.00105251	0.00869509	0.01513412
O	17.43593790	15.22034084	17.91379594	-0.00565422	0.01657966	-0.00423311

H	16.84591401	15.57465276	18.63509950	-0.00823046	0.00880165	-0.03079778
H	13.45617573	11.95915375	15.36853852	0.01280287	0.01882791	0.00086883
O	13.52037703	12.38856516	16.30112791	-0.00013926	0.01362113	-0.00403571
H	13.14018435	13.27745236	16.01641958	0.00955682	0.01920803	-0.00874100
H	14.20718695	19.60932655	12.28271471	-0.00001486	-0.00509539	-0.00860898
O	14.29452664	20.44392394	11.69371838	-0.00393387	-0.01228929	-0.00308222
H	14.13348001	20.08761328	10.78889378	0.00259654	-0.01978167	0.02440441
H	16.04903217	20.47858861	12.12258186	0.00654101	0.01084426	0.00286752
O	16.75416475	20.19129093	12.77302082	-0.01373944	-0.01865632	0.01275991
H	16.77254671	20.93379109	13.54892157	-0.00377249	-0.00561612	0.00358326
H	18.44423257	14.61474540	12.23857175	-0.01676351	0.00532481	0.01567364
O	18.12152805	14.39507908	13.14063764	-0.01843907	0.01451337	-0.00215710
H	18.40374500	15.18474977	13.68568420	-0.01624535	-0.00274197	-0.00619599
H	18.62420195	19.69052352	18.05498457	-0.01365598	-0.00504883	-0.00762352
O	18.18798343	19.02290592	17.48278631	-0.02459484	-0.01195403	-0.01843927
H	17.31087351	18.83882758	17.95090246	-0.01231104	-0.01615728	-0.03092306
H	10.92829885	17.69573013	18.78298351	0.01346728	-0.00531866	-0.01157210
O	11.25434810	18.62018942	18.71369886	0.01703899	-0.00876803	-0.01308105
H	12.20783117	18.49181965	18.43923322	0.01127176	-0.01147533	-0.01423623
H	14.03539699	12.29767466	13.39668123	0.00268996	0.02514749	0.00592345
O	13.17779666	11.90913149	13.73232522	0.01428404	0.02502768	-0.00591404
H	12.56717848	12.68101399	13.61459435	0.01253574	0.00792199	-0.00068777
H	13.87559096	21.53830441	13.07041851	0.00840979	-0.02548805	0.01377757
O	13.89881495	22.05725782	13.92148154	0.01640754	-0.02545322	0.00166242
H	14.86172178	22.21573754	14.06328792	-0.00843582	-0.02366431	-0.00286448
H	10.29172846	15.44478974	15.56079612	0.01390382	0.00286940	-0.00684069
O	9.87115784	15.24473601	14.68032083	0.02609163	0.00530439	0.00282504
H	10.63241706	14.85438880	14.16724550	0.01218311	0.00919690	0.00594604
H	10.83723688	18.91366764	17.10329617	0.01570397	-0.00247561	-0.01958243
O	10.85968149	18.87203410	16.08838085	0.02013674	-0.00416926	-0.00472001
H	11.55294268	19.53607714	15.84137247	0.01022669	-0.01923636	-0.00651461
H	15.94073069	19.22480116	19.69949495	-0.00312707	-0.00591906	-0.01363183
O	15.92709842	18.65205087	18.90324400	-0.00068069	-0.00749327	-0.02043493
H	15.72006932	17.68847927	19.23601510	-0.00063479	-0.00063770	-0.02912432
H	18.88478785	16.01792598	17.47932798	-0.01515847	0.00856885	-0.01503490
O	19.55783342	16.65467242	17.07213008	-0.01636083	0.00839839	-0.00186492
H	19.14241711	17.53256736	17.31317024	-0.01689096	-0.00745012	-0.01554951
H	17.84296050	17.28819554	10.41281630	0.00138096	-0.00262123	0.01917994
O	18.41207241	16.64918902	10.93562373	-0.01011141	0.00975020	0.02328057
H	18.66312278	17.23572214	11.69602973	-0.02399845	-0.00822044	0.01620873
H	16.15372422	18.37502492	11.16297902	-0.00691386	-0.01422076	0.00473508
O	16.30063939	18.31310319	10.18124270	-0.01281480	0.00004715	0.03139796
H	15.74743755	17.51245713	10.00016250	-0.01330146	0.00913947	0.02070366
H	15.23437881	15.97595606	20.44240725	-0.00205579	0.00116356	-0.01429958
O	15.36503358	16.23260611	19.50487106	-0.00225448	0.00342324	-0.02243017

H	14.49505357	15.87904636	19.01570239	0.00657234	0.01020141	-0.03087009
H	14.70782680	21.02947456	16.57825924	0.00915814	-0.02782124	-0.01309436
O	15.67368809	20.82027758	16.71807421	-0.00060062	-0.02389101	-0.00715627
H	15.72425572	19.90170635	16.33123912	0.01064804	-0.00246832	-0.02320111
H	12.94477533	15.64460384	10.65650505	0.01393521	-0.00246294	0.01891921
O	12.69346796	14.65750683	10.74643702	0.01231515	0.00660800	0.01695874
H	12.50666364	14.62006988	11.77650866	0.01838446	0.00898648	0.00947377
H	13.66615676	14.02971485	10.56868403	0.00539000	0.01072135	0.02143048
O	14.78073729	13.43006717	10.34664953	0.00128109	0.00737216	0.01868227
H	15.37347149	14.25048134	10.44625608	-0.00546081	0.00621451	0.01528044
H	12.63286408	15.76096103	17.90169552	0.02038879	0.01190605	-0.02203972
O	13.27537298	15.23493101	18.49383076	0.00392743	0.00935917	-0.02173410
H	13.55158589	14.49271081	17.90967530	0.00677247	0.01035370	-0.01455620
H	17.85007932	21.42608941	14.86077631	-0.01798494	-0.01342035	-0.00398733
O	16.94779379	21.86303443	14.65071397	-0.00590824	-0.01724585	0.00185392
H	16.41783258	21.52768213	15.45041689	-0.00279258	-0.02401517	-0.00840337
H	18.13158288	19.34036419	12.60045116	-0.01583463	-0.01647847	0.01028592
O	18.98717358	18.80585302	12.75087838	-0.02126803	-0.00460280	0.00428405
H	19.66916916	19.26906863	12.21826876	-0.01606120	-0.00397905	0.00570284
H	13.92333414	17.18488569	11.67009555	0.00861405	0.00852262	-0.00393239
O	13.28257795	17.26506128	10.89995459	0.01843329	0.00161963	0.01037285
H	12.50302060	17.66130319	11.38782626	0.02107505	-0.00745583	0.01886127
H	13.26637073	21.34700051	15.18179674	0.00749877	-0.03761076	-0.00352802
O	12.92718387	21.00948710	16.10221031	0.00808164	-0.01608492	-0.00088032
H	12.43276928	21.76883483	16.48254807	0.00733018	-0.01743221	-0.00603122
H	19.06034643	19.69536349	14.37307833	-0.02463212	-0.01301936	0.01334350
O	19.01152315	20.29425846	15.16412633	-0.01710095	-0.02404359	-0.00203093
H	18.59466479	19.73601347	15.87665914	-0.02296067	-0.02215315	-0.01935931
H	9.74133806	16.70474731	14.15073189	0.02289793	0.00788305	0.00279226
O	9.66022611	17.71912186	13.92174391	0.01913874	-0.00414568	0.00946362
H	9.88977119	18.16433360	14.78706305	0.01963867	-0.00873540	-0.00217671
H	17.02187149	10.06901669	13.49049926	-0.00128594	0.01032167	0.00252258
O	16.53202987	10.70972487	14.04575277	-0.01212606	0.01420552	0.00638431
H	16.04111979	11.91350738	13.17423486	-0.00508283	0.02234409	0.00633744
H	16.26292384	13.49617407	12.96715128	-0.02017399	0.02159505	0.02133531
O	15.63416759	12.76207601	12.74445469	-0.00037619	0.03365412	0.01993067
H	15.04959417	12.97444768	11.21761199	-0.00657927	0.01363127	0.00967995
C	13.16798882	15.14791544	14.02793749	0.01409146	0.00326026	-0.01219624
O	12.28011003	14.60931889	13.26881701	0.01446880	0.01848759	0.00947404
O	13.11343982	14.81793243	15.32462432	-0.02340652	-0.01196556	-0.01296411
H	19.21657942	16.62986178	15.66945659	0.00077808	0.00324804	-0.00149640
H	16.18348910	13.46852248	17.33192287	-0.00688959	-0.01176323	0.00242325

*HCOO

115

Lattice="29.843843899999996 0.0 0.0 0.0 32.0645142 0.0 0.0 0.0 30.226147"
Properties=species:S:1:pos:R:3:forces:R:3
Cu 17.11620560 17.55300755 14.99990927 -0.07546150 -0.02567169 -0.00260014
Cu 14.75012470 17.84869078 15.42492736 0.00501069 0.01207088 -0.01520057
Cu 15.39623222 17.27885635 13.30053019 0.01083091 0.01517032 0.00604989
Cu 13.63175011 15.95191533 14.18974354 0.10105294 0.01193178 -0.02017089
Cu 15.76545437 15.61785826 15.34939408 -0.00649708 0.00596029 -0.01037817
Cu 13.16074633 18.28523055 13.61851165 0.03700319 -0.00329038 0.09843575
O 18.96597401 16.63737467 14.69977505 -0.02437825 0.01189737 -0.01027654
O 13.79736294 18.57122489 17.10125240 0.00980147 -0.00964392 -0.01679246
O 11.42387374 18.61591355 12.50427426 0.02279083 -0.00825918 0.01886648
O 11.72008762 16.24007567 17.49944865 0.01684663 0.01275510 -0.02415391
O 16.34800013 13.87693265 16.11524431 -0.01290740 0.01997327 -0.02083912
O 15.92756507 16.04899609 11.75774624 -0.00627041 0.00317353 0.01710040
H 19.55922534 17.23263945 14.18420818 -0.00029422 -0.00559772 0.00042076
H 19.32380201 16.59358083 15.77199937 -0.00351475 -0.00801614 0.00050018
H 14.58363059 18.68194544 17.73447088 -0.00758308 -0.00481549 -0.00044630
H 13.52112430 19.48615434 16.76228135 -0.00231341 -0.00372366 -0.00012828
H 11.18067012 19.55898900 12.36950736 0.00558499 -0.00219330 0.00418409
H 10.57254912 18.20248459 13.03253420 0.00778395 -0.00951613 0.00138248
H 12.20565563 16.86250491 16.90323981 0.02457174 -0.00222949 -0.03787193
H 11.29013879 15.59801426 16.83959359 0.01373292 0.01430860 -0.01054619
H 15.52466083 13.28216642 16.26939591 0.00426542 -0.00511936 -0.00802243
H 17.09059012 13.28017595 15.65752107 -0.01142593 0.00724744 -0.00927408
H 15.84468071 15.17875495 12.23076342 -0.00871657 0.00474497 0.01771281
H 16.93462742 16.18826941 11.58158570 -0.00466001 -0.00632593 0.01145893
H 17.54545271 11.72417290 14.68766033 -0.00478343 0.01922352 0.00121087
O 18.15635132 12.42378929 15.09302376 -0.00798240 0.01602318 -0.00600240
H 18.44374218 12.97118511 14.31684983 -0.01516050 0.00582523 0.00549687
H 17.30576398 14.50551036 17.40022900 -0.00918752 0.01588857 -0.01039221
O 17.80677943 14.99823739 18.11647358 -0.01805714 0.00973324 -0.01323956
H 17.05254728 15.43845419 18.60204374 -0.00399865 0.00810708 -0.02790150
H 14.04512079 11.76393977 16.74364964 0.00458586 0.01050694 -0.01071078
O 14.07965503 12.69227232 16.43324602 0.00144053 0.01624756 -0.00982957
H 13.78384882 12.65765857 15.41209968 0.00826302 0.02716850 -0.00437727
H 14.21498237 19.56231127 12.24642422 0.01046894 -0.01157131 0.00598397
O 14.36285857 20.45553333 11.77160883 0.00127735 -0.01978945 0.00486872
H 14.10754085 20.26768377 10.84010967 0.00102831 -0.02112749 0.01874207
H 16.14621643 20.36953186 12.07271044 0.00220660 -0.01853710 0.02345245
O 16.93893970 19.82234066 12.34521940 -0.00751531 -0.00561245 0.01509157
H 16.56162957 19.27266036 13.10791606 -0.01101564 0.01529607 0.00354999
H 18.72935775 14.74289008 12.29166804 -0.01525279 0.00754357 0.01418185
O 18.32060693 14.44980861 13.13713658 -0.02366549 0.01928260 0.00281975
H 18.47842307 15.23912487 13.75304823 -0.03921036 0.01607082 0.00903324
H 18.66143663 19.68518447 18.13236245 -0.01382262 -0.00658906 -0.00918835

O	18.25179898	19.02140582	17.53551563	-0.01683147	-0.00537896	-0.00637674
H	17.37209970	18.77777997	17.98387324	-0.00824001	-0.01444681	-0.02903639
H	11.09833662	17.81311897	18.54232603	0.00759886	0.00397820	-0.00591354
O	11.26780933	18.79100343	18.56132270	0.01858620	-0.00358263	-0.01404623
H	12.23729329	18.78851472	18.36720141	0.00628793	-0.00442940	-0.00807429
H	14.34722822	12.94795780	13.52221076	0.00292766	0.03320074	0.01119235
O	13.51671827	12.60610605	13.95590098	0.01778255	0.01184915	-0.00708699
H	12.84548171	13.33809527	13.78456145	0.01670188	0.00776884	-0.00037034
H	14.03623937	21.54128129	13.24409915	0.00515033	-0.02009153	0.01326919
O	14.10165420	22.02846390	14.10588410	0.00766289	-0.02494386	-0.00215759
H	15.12135569	22.02225791	14.32836484	-0.00496467	-0.02345263	-0.00196856
H	9.23666513	14.90375276	14.99623383	0.00323463	0.00581458	0.00014471
O	8.62616515	15.26679220	14.30216962	0.01175885	0.00489342	0.00201489
H	8.87223139	14.72652830	13.52029078	0.01369227	0.00355102	0.00216523
H	10.88898163	18.88489417	16.90015603	0.01472326	-0.00606095	-0.02012922
O	10.89393315	18.84686072	15.88923998	0.02326308	-0.01470046	-0.00185949
H	11.55109092	19.54734076	15.64841528	0.01521416	-0.02699938	-0.01476551
H	15.77331186	19.03266660	19.47383802	-0.00286810	-0.00434754	-0.01374169
O	15.91113856	18.45616099	18.69263432	-0.00088884	-0.00489931	-0.01996700
H	15.73284875	17.46537825	19.00212643	0.00066098	0.00104342	-0.02922942
H	18.86612932	15.95146636	17.50111962	-0.01693701	0.01233272	-0.01917785
O	19.55490013	16.61869866	17.09391756	-0.01319150	0.00653647	-0.00089447
H	19.15992997	17.50209582	17.34435873	-0.01904345	-0.00794965	-0.01874916
H	17.73779758	17.24246681	10.34886382	-0.00283391	-0.00411802	0.02250189
O	18.30912026	16.64405648	10.92870718	-0.01070945	0.00720522	0.02047525
H	18.75291004	17.30849347	11.52941418	-0.02345856	-0.00677482	0.01854567
H	16.50177194	18.85301289	10.82849233	-0.01206607	-0.01734478	0.01937988
O	16.38618772	18.38429551	9.95540498	-0.01261597	-0.00216879	0.03107520
H	15.54274835	17.88991427	10.08565132	0.00092269	-0.00140075	0.02803690
H	15.46900933	15.77242905	20.21918530	-0.00278221	0.00094030	-0.01115917
O	15.52998121	16.02211317	19.27361806	-0.00218608	0.00574119	-0.02240472
H	14.74357092	15.42888019	18.78353149	0.00374525	0.00971962	-0.02750960
H	14.68605877	21.07296594	16.84454999	0.01075044	-0.02253948	-0.00535297
O	15.61462181	21.00655617	17.20330670	-0.00307526	-0.02830663	-0.01063602
H	15.93134792	20.17647954	16.76716047	-0.00266292	-0.02479370	-0.02472508
H	12.86991481	15.62569390	10.49459204	0.01167908	-0.00008257	0.02399125
O	12.70454464	14.63776488	10.56043777	0.01118317	0.01117500	0.02733197
H	12.41253726	14.55191517	11.50034719	0.01815288	0.01398022	0.01683091
H	14.16782855	13.95441701	10.31609712	0.00836266	0.00800299	0.01530138
O	15.12332952	13.61547689	10.15174769	0.00201703	0.01653629	0.01743629
H	15.58912845	14.45526360	9.94371476	-0.00215464	0.01251582	0.01139052
H	12.93550019	15.26642172	18.00383735	0.01700972	0.00788121	-0.02253212
O	13.68866127	14.66060992	18.32034609	0.00864721	0.00700641	-0.02590549
H	13.80798818	13.97649002	17.59711964	0.00340441	0.02366459	-0.01981864
H	17.24452888	21.40028345	14.69965155	-0.01432989	-0.01932469	-0.00275178

O	16.55788844	22.10573053	14.88588786	-0.00380903	-0.02800630	-0.00398618
H	16.33775673	21.90820067	15.84088781	-0.00005241	-0.01428053	-0.00855355
H	18.50949087	19.26013568	12.36088467	-0.01766318	-0.01445150	0.01093141
O	19.32717806	18.69706254	12.55181532	-0.01947505	-0.00740297	-0.00210901
H	20.06430117	19.18995304	12.13181404	-0.01557351	-0.00457140	0.00762755
H	13.79554678	17.24161554	11.54322310	-0.00306345	-0.01512498	-0.01164959
O	13.30909549	17.35965707	10.67240047	0.00968038	-0.00211337	0.01241160
H	12.47595091	17.78247641	11.01148863	0.01759956	-0.00742090	0.01282918
H	13.47128679	21.34961113	15.11310605	0.00702706	-0.02999365	-0.00546491
O	13.01908819	20.85782548	16.00363361	0.00679883	-0.01709585	-0.00111147
H	12.47264590	21.54197138	16.44739218	0.00648090	-0.01316434	-0.00419538
H	18.86524263	19.59635847	14.39669509	-0.01638205	-0.00571859	0.01284452
O	18.58789669	20.26136945	15.07028446	-0.01102618	-0.01622583	-0.00316209
H	18.39660285	19.73071357	15.89220498	-0.01053423	-0.00570184	-0.00961031
H	9.17538948	16.91001387	14.02365895	0.01649024	0.00874002	0.00318506
O	9.47018869	17.86358219	13.88761110	0.01187501	-0.01120607	0.00783591
H	9.85999856	18.18123771	14.76236515	0.01649444	-0.01151096	-0.00186404
H	16.38241455	10.08186060	13.59760358	-0.00303925	0.01293857	0.00043000
O	16.20821270	10.90994681	14.09114097	-0.01344153	0.02422270	0.00560461
H	16.00596917	11.58877332	13.38104236	-0.00404362	0.01063145	0.00962724
H	16.77591028	13.59932168	12.76158055	-0.02027133	0.01909219	0.01541693
O	15.87565343	13.20055220	12.58890483	0.00217653	0.01963152	0.02353855
H	15.65196569	13.28770054	11.57362855	-0.00050922	0.01121190	0.01959891
C	11.46476056	15.25423393	14.56723252	-0.02241173	-0.00432299	0.00814707
O	12.06972425	14.78860292	13.50302593	0.00333718	0.01538135	-0.00505822
O	11.05159684	14.56011596	15.54205056	0.02024068	-0.00454865	0.00579772
H	11.28073381	16.36084654	14.56734508	-0.00105692	0.03528982	0.00399465

*HCOOH;*OH

115

Lattice="30.2268376 0.0 0.0 0.0 32.1956128 0.0 0.0 0.0 30.5183865"

Properties=species:S:1:pos:R:3:forces:R:3:initial_charges:R:1

Cu	17.34506747	16.67185443	15.32884980	-0.07046145	-0.00732847	-0.02576930	0.32110000
Cu	15.16784669	17.56196954	16.15554025	0.00991305	-0.00906711	0.01283184	0.17470000
Cu	16.29145799	18.83813390	14.43890076	-0.04026313	0.00080159	0.02893446	0.09680000
Cu	15.18030949	16.39569936	14.11797077	0.01681147	-0.00205831	-0.01912938	0.31690000
Cu	15.69964768	15.21397074	16.20681765	-0.00946089	0.04736619	-0.02394016	0.30480000
Cu	14.07618788	18.58730065	13.87631419	0.04004354	0.00635607	0.04796651	0.13230000
O	18.71547118	16.26910987	13.92109848	-0.02453853	0.01361866	0.01473656	-1.17010000
O	14.10845818	18.54478902	17.60321592	0.00475415	-0.01697417	-0.02247863	-1.22790000
O	12.33533342	18.44627899	12.86614930	0.02912551	-0.00816333	0.01853802	-1.18840000
O	11.39043739	16.25027325	17.23763519	0.01626060	0.01042231	-0.02449394	-1.23140000
O	16.39949230	13.48320092	16.88652775	-0.01967543	0.02091704	-0.01694619	-1.22910000
O	15.68353424	15.56730333	12.31506713	-0.01021045	-0.00186244	0.02208789	-1.23150000
H	19.57407020	16.42435553	14.37311683	0.00147274	0.00515786	-0.00722392	0.56560000

H	14.83804518	18.69882790	18.28767508	-0.00226497	0.00650224	-0.00551252	0.62630000
H	13.89300948	19.42885564	17.15597052	0.00359772	-0.00058562	-0.00755901	0.62590000
H	12.08217580	19.28548358	12.42395814	-0.00110392	-0.00015928	-0.00153292	0.57460000
H	11.40304560	18.09741828	13.40867533	0.01551174	-0.00941312	0.00759364	0.63150000
H	11.39318613	17.17142206	16.84762657	0.02764730	-0.00543703	-0.01462359	0.59250000
H	12.17895723	15.83601600	16.75915800	0.02318270	0.01928737	-0.00486246	0.60460000
H	15.63042782	12.83709718	16.89202096	0.00808953	-0.00752234	-0.00589880	0.61590000
H	17.21165414	13.03193726	16.30404104	-0.00703250	0.00722363	-0.01343702	0.62620000
H	15.75692518	14.58732216	12.53580312	0.00111002	-0.00082475	0.00462373	0.61610000
H	16.64400568	15.88501459	11.96905353	-0.00286811	-0.00202928	0.01481400	0.62590000
H	17.75939373	11.67895848	15.22042493	-0.00535154	0.02021949	0.00071906	0.61080000
O	18.28424311	12.42744453	15.65325707	-0.00762437	0.01631635	-0.00724576	-1.21760000
H	18.48571264	13.01613179	14.85011314	-0.02050215	0.01469211	0.00977087	0.61520000
H	17.63646915	14.23540330	17.97579967	-0.01119496	0.01415447	-0.01123637	0.61040000
O	18.12120215	14.90787483	18.52826442	-0.01794462	0.00630929	-0.00725981	-1.21030000
H	17.34563432	15.33329963	18.97868169	-0.00915454	0.00825799	-0.02990560	0.59940000
H	13.57268524	11.62007546	16.99706160	0.00628013	0.01512940	-0.00804240	0.57180000
O	13.94356091	12.47362991	16.68989478	0.00401577	0.00433604	-0.00404895	-1.20530000
H	13.92379455	12.41848578	15.61697445	0.01392758	0.02086369	-0.00099048	0.62240000
H	14.95191524	20.12918941	12.72878439	-0.00386203	0.00682454	-0.00677609	0.48730000
O	14.93297684	20.76834845	11.93321790	0.00025901	-0.00915824	0.00694089	-1.18680000
H	15.19365632	20.10277405	11.22665847	-0.00469094	-0.00880656	0.02554614	0.60110000
H	16.58311407	21.15237732	12.10825252	-0.00162258	-0.01670484	0.02299286	0.61870000
O	17.56188476	21.35458285	12.26482465	-0.01624567	-0.01382142	0.01766525	-1.19790000
H	17.53763378	21.98538183	13.01825604	-0.01128660	-0.01782428	-0.00008134	0.58190000
H	19.20029298	13.64246350	12.80659996	-0.01426476	0.01067917	0.00333795	0.56440000
O	18.48989875	13.87567266	13.43996623	-0.02291787	0.00416819	0.00106822	-1.20040000
H	18.63020479	15.00036888	13.67935158	-0.02306552	0.00440451	0.00391840	0.62120000
H	18.71176338	19.78383913	18.42905051	-0.01327221	-0.00740385	-0.01110568	0.58050000
O	18.09461648	19.30331094	17.83703881	-0.02006551	-0.00825354	-0.01183955	-1.19420000
H	17.42273590	18.86245028	18.45426309	-0.01222772	-0.01430107	-0.02974597	0.62830000
H	11.43044554	17.57029606	18.91834794	0.01305260	0.00279994	-0.00115972	0.58680000
O	11.72125204	18.50415053	19.06294803	0.01779858	-0.00687280	-0.01393691	-1.18080000
H	12.63533804	18.46216974	18.66901781	0.00905473	-0.01003801	-0.01302279	0.59430000
H	14.68439230	12.64902900	13.75279656	0.00539947	0.02965333	0.01209849	0.60650000
O	13.79056911	12.50122777	14.17052524	0.02208389	0.01637438	-0.00932186	-1.21130000
H	13.30008306	13.36230910	13.90927041	0.02119139	0.01537479	0.00114909	0.61090000
H	14.24712066	21.78540458	13.52661992	0.01015881	-0.02191059	0.01308009	0.60410000
O	14.31568871	22.12405865	14.45052263	0.01428012	-0.02152443	-0.00531455	-1.21610000
H	15.32382201	22.11417019	14.64555284	-0.00018657	-0.02859763	-0.00472259	0.62600000
H	10.48280645	15.45269005	15.74896474	0.01264306	0.00096257	-0.00849494	0.59010000
O	10.15592764	15.29443502	14.82003760	0.02735360	0.00484432	0.00611222	-1.17650000
H	10.96047487	14.94184334	14.33935103	0.00895149	0.00545185	0.00543984	0.59360000
H	11.15254577	18.96715557	17.45527197	0.01165509	-0.00220983	-0.01665416	0.60870000
O	11.12739559	18.92400785	16.44574099	0.02682517	-0.01035087	-0.01153597	-1.22050000

H	11.89966895	19.48899135	16.17569084	0.01771960	-0.02508515	-0.01332035	0.59360000
H	15.95770025	18.84946124	20.17100190	-0.00231037	-0.00415906	-0.01404361	0.58390000
O	16.08984750	18.38202349	19.31851301	0.00064305	-0.00489079	-0.01780964	-1.20220000
H	15.91258171	17.37356645	19.50419985	0.00116207	-0.00051626	-0.03033350	0.64610000
H	18.72146242	16.00722718	17.47775121	-0.01355260	0.00511085	-0.00570529	0.62350000
O	19.05065173	16.73098676	16.82912852	-0.02411273	0.00776682	-0.01161905	-1.21730000
H	18.85663823	17.56691622	17.31963287	-0.01373816	-0.00877654	-0.00422740	0.57480000
H	17.49402381	17.34223801	11.16727090	-0.00617815	-0.00201359	0.03002089	0.60280000
O	17.88649359	16.46993811	11.44677928	-0.01272814	-0.00205112	0.01522806	-1.21180000
H	18.34095687	16.64419709	12.33728296	-0.02826076	0.01188580	0.02197539	0.62190000
H	16.01781359	18.45814523	12.02400421	0.00642611	-0.02274747	-0.00171917	0.51040000
O	16.07488073	18.51486103	11.02465247	0.00381748	-0.00709939	0.02315797	-1.17220000
H	15.26785303	17.94644844	10.78437358	0.00228256	-0.00443325	0.03132393	0.61250000
H	15.65210412	15.54054249	20.59358769	-0.00221235	0.00053834	-0.01378225	0.58340000
O	15.70866609	15.87329005	19.67236015	-0.00182393	0.00434010	-0.02375153	-1.20210000
H	14.95439425	15.35119925	19.17707138	0.00976180	0.01034956	-0.03353580	0.64130000
H	15.15458428	20.99765937	17.13083114	0.01681228	-0.02872320	-0.01118291	0.57740000
O	15.94083512	21.31749199	17.64233245	-0.00720758	-0.02950891	-0.01249436	-1.17730000
H	16.59894777	20.57951249	17.55896398	-0.01932391	-0.01946800	-0.02343270	0.58320000
H	13.16658009	15.19633372	10.52890399	0.00768339	-0.00223738	0.01567409	0.59360000
O	13.10111316	14.25414792	10.83451531	0.01332609	0.00926468	0.02655303	-1.18770000
H	12.94424424	14.39141740	11.80951751	0.01718757	0.01082328	0.01368156	0.60570000
H	14.56272856	13.61213096	10.48045771	0.00751063	0.00679202	0.01399399	0.62410000
O	15.49347224	13.22623835	10.27788079	0.00023379	0.01726713	0.01745090	-1.17680000
H	16.00149053	14.01695282	9.99642099	-0.00368407	0.01373563	0.01632325	0.56660000
H	13.03650021	15.10459119	18.36673820	0.02102095	0.00815197	-0.01648501	0.59810000
O	13.73678188	14.50071143	18.70872728	-0.00013525	0.00702677	-0.02173656	-1.19000000
H	13.86128763	13.83886832	17.96858167	0.01107588	0.01942441	-0.01068230	0.61590000
H	17.32686615	21.28015534	15.09316243	-0.01096315	-0.01045488	0.00149278	0.61210000
O	16.86882920	22.16340039	15.19830080	-0.00647080	-0.02349800	0.00033761	-1.21920000
H	16.60786976	22.11778850	16.16632115	-0.00097978	-0.01790056	-0.01432726	0.59060000
H	18.60358098	20.13402770	12.49811184	-0.01857276	-0.02003870	0.01086701	0.62890000
O	19.21842232	19.37622495	12.82158189	-0.01905504	-0.01842336	0.00025678	-1.17900000
H	20.12730728	19.69917969	12.64989501	-0.01476894	-0.00441478	0.00431441	0.57240000
H	14.45063683	16.33789665	11.46741713	0.00199749	0.00773544	0.02099622	0.61700000
O	13.89218268	16.93125295	10.86990437	0.00766390	0.00079842	0.02445388	-1.21460000
H	13.26681457	17.36954232	11.51004070	0.01650384	-0.01093490	0.02172026	0.59780000
H	13.74107591	21.24823370	15.43542802	0.01348642	-0.03466833	-0.00636622	0.63490000
O	13.34726614	20.72820730	16.29594339	0.00799363	-0.01535064	-0.00093087	-1.21480000
H	12.87410389	21.42626151	16.80095150	0.00883075	-0.01061432	-0.00526451	0.57680000
H	18.73917953	19.48285981	14.24490106	-0.00071154	-0.01220857	0.00803952	0.63760000
O	18.07496132	19.70845477	15.02737050	-0.02150859	-0.00468234	0.00415968	-1.22670000
H	18.35748771	19.30340494	15.88435134	-0.00369446	-0.01165173	-0.00094906	0.62010000
H	10.20497202	16.77391561	14.31074086	0.01881535	0.00557248	0.00492225	0.62970000
O	10.29165575	17.78373333	14.07427359	0.02029435	-0.00652197	0.01302603	-1.22190000

H	10.40691533	18.22417084	14.96184780	0.01720781	-0.01061920	-0.00335899	0.61480000
H	16.72784468	10.00287198	14.01028925	-0.00312864	0.01413232	-0.00039816	0.56810000
O	16.50873856	10.84892003	14.45269775	-0.01331217	0.02365048	0.00428684	-1.16980000
H	16.36239394	11.49521264	13.69389329	-0.00356269	0.01315931	0.00849430	0.60600000
H	17.10690166	13.31072517	12.94866832	-0.01901470	0.02069476	0.01179648	0.62210000
O	16.15970603	12.93375889	12.77071004	-0.00375612	0.02037701	0.01913662	-1.26360000
H	15.99381411	12.92013166	11.74680674	-0.00135999	0.01124447	0.01828204	0.62370000
C	13.25153617	15.67911575	14.48740255	-0.00683303	-0.00532278	-0.01789590	0.79510000
O	12.70492044	14.83968504	13.62316892	0.01033518	0.01958885	-0.01317838	-1.12210000
O	13.33353495	15.09039624	15.87915299	0.00392588	-0.01415223	0.00550348	-1.05770000
H	12.67004897	16.62106711	14.65719303	-0.01230310	0.00742361	0.02077061	0.04000000
H	13.11157449	14.12738894	15.80488435	0.01245465	0.02604677	0.00417420	0.58600000

*HCOOH

116

Lattice="31.438136 0.0 0.0 0.0 32.0238699 0.0 0.0 0.0 30.275470499999997"
Properties=species:S:1:pos:R:3:forces:R:3

Cu	18.44654910	17.26503286	15.02434105	-0.06005955	0.00342865	-0.00058144
Cu	16.13541011	17.80836457	15.55977026	0.01023708	0.01314456	-0.01597222
Cu	16.60543080	17.09012695	13.43326248	-0.00698322	0.01013301	0.00163999
Cu	14.85780899	15.92068353	14.65323868	0.04404023	-0.02099196	-0.01277591
Cu	17.03361022	15.47691442	15.62057103	-0.00686524	0.00762651	-0.00343429
Cu	14.52852199	18.26849790	13.73586595	0.03697530	-0.00765281	0.08734163
O	20.31674417	16.62927000	14.73004777	-0.02786570	0.01017669	-0.00914508
O	15.21779426	18.59407889	17.19447883	0.01177361	-0.00894146	-0.01688180
O	12.81664377	18.55524318	12.55428757	0.02218603	-0.00886928	0.01797265
O	13.28156348	16.18509175	17.46602489	0.01340750	0.01536891	-0.01417473
O	17.88857813	13.76396031	16.29342334	-0.01487661	0.02083775	-0.02028453
O	17.26036689	15.98100124	11.91604487	-0.01056001	0.00420629	0.01519715
H	20.84287389	17.26101320	14.18306367	0.00407080	-0.00435041	-0.00214483
H	20.71053019	16.60379310	15.88617874	-0.00107899	-0.00590218	0.00473641
H	15.99335587	18.67218973	17.85017107	-0.00771626	-0.00496014	-0.00086600
H	14.95731524	19.51757414	16.85935446	-0.00172897	-0.00474361	-0.00063239
H	12.61586027	19.50376049	12.39242255	0.00366151	-0.00095045	0.00308822
H	11.91883843	18.18398114	13.09367923	0.00914253	-0.00671719	0.00237795
H	13.81043670	16.83517131	16.93979531	0.01322503	0.00153166	-0.02066900
H	12.83855112	15.61940119	16.70634252	0.01735024	0.01678385	-0.01448248
H	17.10309671	13.13722099	16.47005260	0.00689418	-0.00716493	-0.00623711
H	18.61845772	13.21699811	15.74986968	-0.01473634	0.01446263	-0.01019567
H	17.20763638	15.06327197	12.30545044	-0.00460668	0.00015243	0.01283138
H	18.25563980	16.15294111	11.70538457	-0.00215321	-0.00346695	0.01084091
H	19.05262129	11.73526115	14.66891783	-0.00491809	0.01877536	0.00132608
O	19.67193387	12.42617828	15.07853510	-0.00810468	0.01627026	-0.00542309
H	19.92235226	13.00236825	14.30952661	-0.01504199	0.00578547	0.00553674
H	18.84991214	14.44160430	17.49274295	-0.01019862	0.01713714	-0.01161708

O	19.33160111	14.99525444	18.18446529	-0.01905219	0.00928869	-0.01128046
H	18.55043890	15.42564233	18.64422238	-0.00652765	0.00799889	-0.02848783
H	15.37129062	11.72162121	16.88744930	0.00400142	0.01147960	-0.01018371
O	15.56138918	12.63346603	16.58471417	-0.00086603	0.01258960	-0.00890662
H	15.30816167	12.65158748	15.55213961	0.00704710	0.02815865	-0.00438586
H	15.54875794	19.53034579	12.38806223	0.01113315	-0.01473745	0.00895680
O	15.74355000	20.37333312	11.82965548	0.00087180	-0.01877251	0.00388757
H	15.48967539	20.09428430	10.91941208	-0.00027655	-0.02055971	0.01827654
H	17.46626914	20.17418344	12.12332114	0.00121885	-0.01920290	0.02400188
O	18.23898606	19.57950953	12.36968487	-0.00658650	-0.00789046	0.01589678
H	17.81402257	18.99719964	13.07777891	-0.00798004	0.00861146	0.00724585
H	20.14279620	14.75688039	12.29704362	-0.01385667	0.00566504	0.01537815
O	19.76489594	14.43064065	13.14555636	-0.02420697	0.01824239	0.00368716
H	19.89674784	15.21909800	13.76975163	-0.03274483	0.00842068	0.00539691
H	19.98356850	19.66287042	18.10371795	-0.01368336	-0.00613855	-0.00816366
O	19.56267278	18.97586149	17.54230042	-0.01607285	-0.00715224	-0.00756792
H	18.70419492	18.71856379	18.03807802	-0.00858530	-0.01586072	-0.02801631
H	12.57737402	17.62909745	18.42391061	0.00931098	0.00196798	-0.00958373
O	12.64558616	18.61666004	18.54508840	0.01879381	-0.00298954	-0.01339084
H	13.61503776	18.72198914	18.39069031	0.00674071	-0.00517190	-0.00951908
H	15.89083128	13.01141200	13.65522217	0.00153632	0.03403990	0.01184994
O	15.09554002	12.62071258	14.11101139	0.01705312	0.01604158	-0.00755913
H	14.34447008	13.24007721	13.84602933	0.02229780	0.01787353	0.00226319
H	15.40774355	21.48154389	13.28608274	0.00418390	-0.01967388	0.01300343
O	15.48903151	21.99128817	14.13251184	0.00632104	-0.02446853	-0.00156793
H	16.50816407	21.97995825	14.34404747	-0.00570923	-0.02294772	-0.00101139
H	10.85078427	15.13013170	15.06487366	0.00908837	0.00452877	0.00311877
O	10.02218185	15.46693685	14.57090684	0.01555163	0.00259439	-0.00109082
H	9.96830741	14.87799459	13.78948945	0.01409554	0.00172247	0.00101891
H	12.32265834	18.81372472	16.91237803	0.01772743	-0.00729826	-0.02053277
O	12.32492328	18.85427765	15.89822449	0.02223188	-0.01583689	-0.00092643
H	12.97551662	19.57098308	15.70060352	0.01285046	-0.02639192	-0.01212771
H	17.20197658	18.93463881	19.57182124	-0.00274950	-0.00411418	-0.01405156
O	17.30052597	18.39660569	18.75765329	-0.00097051	-0.00460162	-0.01799028
H	17.13627560	17.36132933	19.03211955	-0.00081404	-0.00039441	-0.03120707
H	20.26014499	15.92502261	17.53579572	-0.01923808	0.01100235	-0.01960648
O	20.93439527	16.62680829	17.09499544	-0.01221211	0.00663956	-0.00200694
H	20.51779911	17.50328791	17.35092472	-0.01925854	-0.00922896	-0.01892575
H	19.00781879	17.15707315	10.38668883	-0.00346024	-0.00355752	0.02282844
O	19.59692621	16.57930055	10.97856062	-0.01006058	0.00690096	0.02002365
H	20.07826060	17.25697385	11.53052570	-0.02150393	-0.00733693	0.01739164
H	17.83265823	18.70389953	10.81759819	-0.01031688	-0.01562685	0.01694088
O	17.73932086	18.22409032	9.94631792	-0.01356910	-0.00160713	0.02962720
H	16.87749129	17.75602314	10.05067634	0.00328937	-0.00247674	0.02624832
H	16.89645233	15.76279555	20.22161529	-0.00266823	0.00131754	-0.01292099

O	16.98228440	15.96933954	19.26709219	-0.00080659	0.00481569	-0.02401769
H	16.13675865	15.34872884	18.76648644	0.00260044	0.00792140	-0.03354869
H	16.18855980	21.07190790	16.96167326	0.01080523	-0.02241307	-0.00495998
O	17.11969110	21.02486781	17.31927663	-0.00421024	-0.02925233	-0.01091884
H	17.43528723	20.17200203	16.93881350	-0.00887066	-0.02287874	-0.02506950
H	14.30969266	15.63099638	10.70035079	0.01171815	-0.00017757	0.02694272
O	14.16835815	14.63948031	10.73621662	0.01060472	0.01213973	0.02972632
H	13.79206944	14.49091320	11.64441343	0.01868779	0.01671654	0.01981691
H	15.58281509	13.95858034	10.41363855	0.00837966	0.00769501	0.01520288
O	16.51631653	13.58030741	10.18437975	0.00267584	0.01578646	0.01659463
H	16.99311730	14.38724283	9.89399031	-0.00054489	0.01524602	0.01522486
H	14.39455417	15.24451940	17.98986209	0.01938532	0.00981826	-0.02617614
O	15.14083762	14.63264092	18.36301186	0.00855100	0.00734938	-0.02113870
H	15.29657264	13.92505833	17.66317575	0.00607006	0.02819415	-0.02197817
H	18.63826655	21.34586208	14.73160787	-0.01479317	-0.01666556	-0.00234195
O	17.94791472	22.05033019	14.91030494	-0.00469451	-0.02734083	-0.00272304
H	17.75238168	21.87955489	15.87537934	-0.00100409	-0.01331736	-0.00776350
H	19.84354221	19.16700231	12.40606396	-0.01604659	-0.01394659	0.01082299
O	20.68834876	18.63678025	12.58272836	-0.01943383	-0.00879558	-0.00107128
H	21.40716134	19.16035751	12.16875526	-0.01504429	-0.00518162	0.00723161
H	15.21524995	17.34233805	11.61173122	0.00056948	-0.01195310	-0.01410551
O	14.71023235	17.40174449	10.74388217	0.00722820	-0.00171036	0.01152685
H	13.86452278	17.80025053	11.09079871	0.01710603	-0.00678277	0.01429957
H	14.91151085	21.33852777	15.19033131	0.00634780	-0.02991287	-0.00496877
O	14.51294344	20.88037674	16.12184330	0.00763397	-0.01667562	-0.00127532
H	13.95978110	21.56761529	16.55117237	0.00606148	-0.01361884	-0.00507224
H	20.19911949	19.53902996	14.40404212	-0.02379419	-0.01396653	0.01615035
O	19.95392278	20.19813582	15.09473650	-0.01313568	-0.02065829	-0.00321134
H	19.71758299	19.65227916	15.89325023	-0.01801702	-0.02011218	-0.01380498
H	10.52895214	16.97472988	14.10419827	0.01726134	0.00725103	0.00400935
O	10.84707216	17.92724549	13.91789690	0.01245257	-0.00875274	0.00768523
H	11.27300820	18.24015318	14.77901648	0.01984540	-0.01561824	-0.00265268
H	17.78894735	10.14145070	13.59585047	-0.00334016	0.01381688	0.00078002
O	17.70682316	10.98225815	14.09146858	-0.01399419	0.02347127	0.00625385
H	17.47625579	11.66643347	13.39316031	-0.00423503	0.01143949	0.00982954
H	18.24479715	13.60248359	12.74570400	-0.01940530	0.01861867	0.01489077
O	17.32848728	13.22897676	12.58910787	0.00239782	0.01978033	0.02244579
H	17.08873708	13.29255562	11.56839232	-0.00052945	0.01102520	0.01884749
C	13.01978447	15.25990938	14.40148526	0.02110061	0.01740348	0.00040080
O	13.11096516	14.20862716	13.33454332	0.00604192	0.00772299	-0.00402907
O	12.40494349	14.80056446	15.48551890	0.01775445	0.00462047	0.01367345
H	12.53371783	16.13356178	13.90596988	0.00418667	0.02857327	-0.01150252
H	12.34863272	13.60964093	13.52660728	0.00858928	0.00816880	-0.00342826

*HC(OH)$_2$;*OH

116
Lattice="30.004320800000002 0.0 0.0 0.0 32.09673 0.0 0.0 0.0 30.5891119" Properties=species:S:1:pos:R:3:forces:R:3
Cu 17.24094085 16.75013034 15.19551989 -0.07600043 -0.00103919 -0.02915669
Cu 15.10993678 17.49403324 16.25794534 0.00738241 -0.02896005 0.01669843
Cu 16.08340465 18.84433004 14.46582304 -0.03163546 -0.00177786 0.03331571
Cu 14.98816489 16.40694809 14.21034678 0.00775616 0.01829617 -0.02866176
Cu 15.88988510 15.22169194 16.34485520 -0.01169526 0.06409545 -0.02722880
Cu 13.84987246 18.54875722 13.92609181 0.02583901 -0.00180025 0.05429645
O 18.72282476 16.21943978 13.91054337 -0.02622735 0.01286180 0.01448971
O 14.00464300 18.47763023 17.67714736 0.00601553 -0.01605400 -0.02288736
O 12.07688001 18.41471051 12.90282483 0.02971429 -0.01041312 0.01795631
O 11.64814508 16.03872705 17.31698883 0.01838002 0.01911846 -0.01953020
O 16.28301235 13.40203097 16.93834727 -0.01907366 0.02066970 -0.01748726
O 15.57374623 15.61246685 12.34724375 -0.01289177 -0.00172679 0.01945565
H 19.53578637 16.31508645 14.45392625 0.00268233 0.00057420 -0.01006845
H 14.72151208 18.64299473 18.37188388 -0.00362843 0.00681878 -0.00560724
H 13.78311962 19.35622612 17.22615030 0.00338457 -0.00046068 -0.00893022
H 11.81235640 19.24218068 12.44468463 0.00168377 -0.00290884 0.00074142
H 11.15619032 18.09328443 13.49826461 0.01619903 -0.01010854 0.00621635
H 11.67054798 16.97970719 16.99752158 0.02907382 -0.00633250 -0.01949758
H 12.25967019 15.56834827 16.64039143 0.02389352 0.01610352 -0.00462634
H 15.46591124 12.80743836 16.85958752 0.00530647 -0.00539566 -0.00845814
H 17.10054506 12.95503613 16.34079044 -0.00473682 -0.00207758 -0.01351072
H 15.64799834 14.63692327 12.59934148 0.00069497 -0.00104929 0.00423772
H 16.52323429 15.90611714 11.99932404 -0.00798719 -0.00010731 0.02310629
H 17.64711369 11.64383807 15.25253645 -0.00509400 0.02059646 0.00161674
O 18.16502913 12.39798081 15.68129633 -0.00772870 0.01645225 -0.00583956
H 18.33908448 12.99727401 14.86746407 -0.01966400 0.01465571 0.01310119
H 17.54879229 14.12822583 18.05883414 0.00146500 0.00551659 0.00272533
O 17.99235649 14.87844764 18.53726953 -0.01230773 0.00665660 -0.00242230
H 17.20009141 15.27719531 18.98760808 -0.00925209 0.00890870 -0.03045071
H 13.78226912 11.34238170 16.95932946 0.00556548 0.01404336 -0.00863708
O 13.87590589 12.28775773 16.72177762 0.00518772 0.01824186 -0.01132913
H 13.73491980 12.31106267 15.69476350 0.01208433 0.01785455 0.00138484
H 14.77237184 20.08920347 12.77769904 -0.00442625 0.00763110 -0.00794845
O 14.79350966 20.75753031 12.00181910 0.00031451 -0.00862891 0.00586306
H 14.98786579 20.10331242 11.26809812 -0.00426008 -0.00746558 0.02534150
H 16.44594987 21.09192469 12.14561569 -0.00134267 -0.01661454 0.02320621
O 17.42964859 21.28420708 12.28813455 -0.01581399 -0.01349857 0.01786086
H 17.41440110 21.91472673 13.04268070 -0.01122929 -0.01763352 -0.00049120
H 19.01498010 13.53730463 12.85007775 -0.01286842 0.00968382 0.00181439
O 18.33940565 13.84028728 13.49220012 -0.02428499 -0.02563387 -0.00419824
H 18.57245626 15.05383652 13.72039625 -0.01171203 0.03647038 0.00957952
H 18.57291985 19.71182436 18.46045278 -0.01348787 -0.00766589 -0.01131006
O 17.97465825 19.21263780 17.86442023 -0.02024697 -0.00953029 -0.01164296

H	17.29710686	18.77640728	18.47831620	-0.01293084	-0.01512181	-0.03034196
H	11.30980828	17.52982244	19.16412371	0.01108011	-0.00056046	-0.00432506
O	11.62904405	18.45879278	19.11984224	0.01758132	-0.00675361	-0.01340194
H	12.53258392	18.34217373	18.71179805	0.01089726	-0.00895996	-0.01136079
H	14.46966635	12.66854477	13.70186905	0.00398376	0.02911938	0.00965259
O	13.59249938	12.40257113	14.10089889	0.01692847	0.02654063	-0.00628757
H	13.05723252	13.22767869	14.00640767	0.01916844	0.00433532	-0.00490185
H	14.09303451	21.72878452	13.57539953	0.01023615	-0.02276765	0.01299353
O	14.19273930	22.06762066	14.49654665	0.01390822	-0.02119030	-0.00507394
H	15.20665020	22.04657170	14.66176894	-0.00015780	-0.02912507	-0.00441875
H	10.45507761	15.40358022	15.91206847	0.01171615	0.00198518	-0.00864431
O	10.01486928	15.31482962	15.02321185	0.02997905	0.00700005	0.00228333
H	10.73551799	14.96119818	14.43830064	0.00641276	0.00351470	0.00522955
H	11.05425405	18.84723890	17.49783113	0.01135885	-0.00113770	-0.01829128
O	11.07731632	18.82434688	16.49103365	0.02651862	-0.01058673	-0.01115012
H	11.80935440	19.45532890	16.25380733	0.01835335	-0.02521343	-0.01404677
H	15.88082988	18.75364034	20.25456040	-0.00223593	-0.00463801	-0.01420530
O	15.97749252	18.30363962	19.38877334	0.00080707	-0.00449326	-0.01636995
H	15.79317119	17.29361957	19.55883657	0.00090402	-0.00058996	-0.03079153
H	18.59396111	15.97512923	17.39197516	-0.02045909	0.00566672	-0.01243523
O	18.92661199	16.70160679	16.75931069	-0.02189174	0.00846479	-0.00565119
H	18.73917412	17.52455958	17.27632437	-0.01752595	-0.01138928	-0.00615144
H	17.41269405	17.33648610	11.17446893	-0.00362447	-0.00253778	0.02941553
O	17.79267675	16.46121367	11.44454613	-0.01355403	-0.00083689	0.01612492
H	18.29170114	16.64111584	12.30068985	-0.02626272	0.01155310	0.02337390
H	15.71222709	18.35030219	12.03808479	0.00753163	-0.02428543	-0.00130022
O	15.79602789	18.44569689	11.04201615	0.00074731	-0.01076056	0.02388346
H	15.04975087	17.85018940	10.74533817	0.00625066	-0.00240917	0.02982919
H	15.50684288	15.44485418	20.62085725	-0.00191181	0.00082069	-0.01447588
O	15.57783463	15.78655744	19.70364338	-0.00233840	0.00519049	-0.02088762
H	14.81494776	15.27891460	19.19169156	0.01055206	0.01139105	-0.03336691
H	15.02669782	20.89657130	17.10088864	0.01636455	-0.03027381	-0.01267956
O	15.82568125	21.20920349	17.59788980	-0.00664306	-0.02988687	-0.01205080
H	16.47222379	20.46172178	17.51707148	-0.01984299	-0.02045406	-0.02406761
H	12.97529952	15.00549612	10.61332837	0.00901158	-0.00028837	0.01179603
O	12.87850159	14.01283111	10.63076266	0.01217790	0.01183009	0.02712760
H	12.71279130	13.84243411	11.58678765	0.01733772	0.00261979	0.00761982
H	14.45341200	13.47427547	10.39260275	0.00716332	0.00821826	0.01306550
O	15.42048489	13.16669194	10.30633369	-0.00096390	0.01675918	0.01852217
H	15.88810401	13.98645838	10.03787603	-0.00454016	0.01345779	0.01613729
H	12.90179334	15.10203253	18.34604075	0.01928122	0.01131042	-0.01915762
O	13.60381866	14.48394798	18.69663271	0.00308471	0.00800655	-0.02573479
H	13.67609989	13.76660120	18.00928861	0.01023690	0.02092517	-0.01474215
H	17.21711568	21.22496115	15.08424868	-0.00978169	-0.00809982	0.00201300
O	16.74969437	22.10617748	15.17939950	-0.00652885	-0.02148609	0.00099651

H	16.48693800	22.05316698	16.14767964	-0.00155132	-0.01779115	-0.01468728
H	18.45695181	20.04986439	12.52436389	-0.01882159	-0.02053867	0.01145806
O	19.07223728	19.28960301	12.84165471	-0.01944460	-0.01869734	0.00172294
H	19.97894285	19.61908514	12.66957650	-0.01498394	-0.00508728	0.00452367
H	14.28407634	16.27243028	11.62669707	0.00220333	0.00791712	0.01150679
O	13.56529676	16.70126092	11.04538064	0.01050906	0.00347215	0.01561051
H	13.07625462	17.33022200	11.64586594	0.01514082	-0.01293817	0.01142088
H	13.62628358	21.19371503	15.47801928	0.01330400	-0.03496248	-0.00704437
O	13.23393616	20.66996291	16.34306185	0.00838920	-0.01582742	-0.00002345
H	12.78435842	21.37455960	16.86066238	0.00842720	-0.01092756	-0.00586707
H	18.59649837	19.41965370	14.28251060	-0.00369626	-0.01628446	0.00941607
O	17.94291673	19.67295831	15.05898496	-0.02076959	-0.00402545	0.00512005
H	18.21385563	19.27155373	15.92088129	-0.00535267	-0.00774120	-0.00181743
H	10.01731286	16.81747838	14.48498576	0.01821904	0.00599360	0.00218560
O	10.08202294	17.81878786	14.23033725	0.02106718	-0.00928411	0.00903777
H	10.30829473	18.24261252	15.11257314	0.01981382	-0.01245933	-0.00258088
H	16.62934169	10.03546148	13.96911977	-0.00336843	0.01467173	-0.00016222
O	16.37231277	10.82816625	14.48343110	-0.01393973	0.02410216	0.00441079
H	16.19637176	11.52776323	13.78112488	-0.00371753	0.01260618	0.01016352
H	16.97991800	13.35020804	13.06345200	-0.02421650	0.02004096	0.01010106
O	15.99781392	13.01152090	12.87606387	-0.00641326	0.01954119	0.01939957
H	15.87410858	12.93603968	11.85910981	-0.00049872	0.01101734	0.01750574
C	13.07890739	15.80772660	14.44867964	0.00239687	-0.00115753	-0.00973456
O	12.37878663	15.08123790	13.35525129	0.01210392	0.01712237	-0.00174437
O	13.00828610	14.80898651	15.51835143	0.00385225	-0.00352021	0.00784922
H	12.34700942	16.58030281	14.77559602	0.00406735	0.01261730	0.01733055
H	12.39886824	15.69898025	12.58481082	0.01035515	0.00862431	0.03186284
H	13.96532725	14.67143394	15.77254367	-0.00648225	0.00028606	0.00619699

*CHO;*OH

116

Lattice="30.2268376 0.0 0.0 0.0 32.1956128 0.0 0.0 0.0 30.5183865" Properties=species:S:1:pos:R:3:forces:R:3:initial_charges:R:1

Cu	17.38852738	16.72869588	15.22288239	-0.07389691	-0.00391014	-0.03249266	0.33270000
Cu	15.31422985	17.55294345	16.22742225	0.09262748	0.02275050	0.05658407	0.20650000
Cu	16.26655863	18.88381476	14.42771871	-0.04152250	0.00136867	0.03193006	0.07520000
Cu	15.16051814	16.39440013	14.10663688	-0.00354777	-0.01508592	-0.03535183	0.32580000
Cu	15.84368973	15.20852558	16.21311427	0.00362740	0.03511303	-0.02122305	0.29760000
Cu	14.06561997	18.56454505	13.78426177	0.02889761	0.00674458	0.03274248	0.15160000
O	18.80882998	16.23166465	13.88745583	-0.02636383	0.01371658	0.01476395	-1.17710000
O	14.13036896	18.52937330	17.63955453	0.00477685	-0.01762955	-0.02243921	-1.21680000
O	12.32574476	18.31816695	12.83120691	0.02927288	-0.01070128	0.01901497	-1.18170000
O	11.51105631	16.01915221	17.41550760	0.01527068	0.01733034	-0.01594829	-1.21970000
O	16.49792362	13.47761111	16.87675555	-0.01987567	0.02187595	-0.01956745	-1.22080000
O	15.72831388	15.63913259	12.30964570	-0.01448881	-0.00256987	0.02094295	-1.23550000

H	19.65052495	16.36677426	14.37481079	0.00219936	0.00309980	-0.00647769	0.57020000
H	14.84005510	18.70545704	18.33877204	-0.00490520	0.00627572	-0.00582004	0.61840000
H	13.90622448	19.40500611	17.18635935	0.00321406	-0.00225447	-0.00903508	0.61860000
H	12.03994817	19.12511017	12.34787835	-0.00323127	0.00011303	-0.00239704	0.56660000
H	11.38612444	18.01288583	13.47481668	0.01607301	-0.01306450	0.00993891	0.62600000
H	11.53289387	16.94519517	17.06490718	0.02167522	-0.00939690	-0.01494360	0.57430000
H	12.12825021	15.54088171	16.73312621	0.01674834	0.01171898	-0.00350172	0.60480000
H	15.71595824	12.83763066	16.93920494	0.00633631	-0.00495902	-0.00534395	0.61530000
H	17.29440059	12.98115900	16.29712077	-0.00749537	0.00608721	-0.01329277	0.62320000
H	15.79838130	14.65829787	12.53256745	0.00191015	0.00038131	0.00275367	0.61460000
H	16.69695343	15.94651553	11.95775701	-0.00425525	-0.00058024	0.01823632	0.62720000
H	17.73658142	11.61925377	15.19291344	-0.00495855	0.02051485	0.00180365	0.61010000
O	18.31407933	12.32583804	15.63372637	-0.00766666	0.01606490	-0.00678590	-1.21400000
H	18.51245426	12.93445055	14.84360116	-0.01990620	0.01420625	0.00962297	0.61180000
H	17.64716518	14.23736370	17.98163242	-0.00520164	0.01039373	-0.00478369	0.61000000
O	18.12326850	14.93333529	18.51671017	-0.01477024	0.00688502	-0.00398402	-1.20540000
H	17.34311100	15.34386787	18.97484815	-0.01018613	0.00857021	-0.03056558	0.59060000
H	13.97690649	11.36473786	17.13442113	0.00502996	0.01421816	-0.00842993	0.57760000
O	14.13887514	12.30122396	16.90072826	0.00370425	0.01357284	-0.00990212	-1.18680000
H	13.96507530	12.37127169	15.89263003	0.01090807	0.02071442	-0.00197958	0.62250000
H	14.86836001	20.12912405	12.68930777	-0.00477743	0.00479966	-0.00595632	0.48570000
O	14.90485771	20.77642886	11.90002991	0.00073527	-0.00952809	0.00627527	-1.18340000
H	15.17946147	20.11378304	11.19881770	-0.00475788	-0.00861909	0.02544664	0.60400000
H	16.55928102	21.13998913	12.10665622	-0.00139393	-0.01733471	0.02347076	0.61330000
O	17.53872095	21.33791239	12.26413804	-0.01593581	-0.01368082	0.01737239	-1.18700000
H	17.51646444	21.98054088	13.00760148	-0.01161570	-0.01818755	0.00012313	0.57580000
H	19.16235400	13.58643120	12.79424667	-0.01422968	0.01098161	0.00335680	0.56090000
O	18.48389349	13.84745625	13.45138965	-0.02341878	0.00304459	0.00131412	-1.20020000
H	18.67516696	14.97779943	13.67460763	-0.02181858	0.00485307	0.00514199	0.62260000
H	18.70890717	19.77800255	18.43285277	-0.01371087	-0.00730745	-0.01122287	0.58170000
O	18.08879669	19.29147409	17.84914598	-0.01994372	-0.00884791	-0.01164244	-1.19470000
H	17.41799692	18.86239977	18.47808525	-0.01254585	-0.01437850	-0.02939580	0.62760000
H	11.42246331	17.56526016	19.11591390	0.01225521	0.00198066	-0.00224990	0.58510000
O	11.73158438	18.49913193	19.06772525	0.01837516	-0.00747172	-0.01396430	-1.17340000
H	12.63882979	18.38776985	18.66708816	0.01052537	-0.01070023	-0.01326477	0.59630000
H	14.61886327	13.05863550	13.91973740	0.01050774	0.02998918	0.01258330	0.61180000
O	13.74372792	12.95844543	14.39204610	0.02201195	0.01536787	-0.00650004	-1.23110000
H	13.59541882	13.84722969	14.90526147	0.02704186	0.01783829	0.00183114	0.61570000
H	14.23557707	21.79960654	13.52542525	0.01078569	-0.02094825	0.01313670	0.60330000
O	14.31445306	22.12340565	14.45304369	0.01472436	-0.02164745	-0.00547781	-1.21550000
H	15.32268770	22.11273154	14.63983353	0.00010340	-0.02838962	-0.00497464	0.62590000
H	10.50286447	15.29278156	15.72182154	0.01354290	0.00097843	-0.01262764	0.59330000
O	10.12737756	15.28518277	14.80723415	0.03117920	0.00482835	0.00567599	-1.17950000
H	10.84841520	14.90379843	14.22968320	0.01196381	0.01073908	0.01266304	0.60670000
H	11.16544168	18.88725649	17.46113033	0.01235456	-0.00163261	-0.01745277	0.60670000

O	11.14643824	18.91038321	16.45331860	0.02735820	-0.00836558	-0.00941668	-1.22900000
H	11.90224788	19.51048818	16.21615356	0.01691868	-0.02395729	-0.01148186	0.60140000
H	15.96862727	18.83078802	20.23453291	-0.00212507	-0.00449572	-0.01428800	0.58200000
O	16.10555257	18.38284748	19.37360290	0.00070678	-0.00448846	-0.01659082	-1.20180000
H	15.92136764	17.36666931	19.53605731	0.00115682	-0.00038822	-0.03026223	0.64300000
H	18.67632530	16.03752616	17.42600951	-0.01749486	0.00532542	-0.00721538	0.62410000
O	18.99144125	16.76903028	16.77764350	-0.02398026	0.00763762	-0.01130921	-1.21490000
H	18.81659284	17.59730889	17.29036930	-0.01654214	-0.00978130	-0.00462422	0.57860000
H	17.54898194	17.36616482	11.16205725	-0.00607348	-0.00217250	0.03005957	0.60800000
O	17.92338387	16.48531450	11.43737539	-0.01226033	-0.00101055	0.01537433	-1.21780000
H	18.40689418	16.64527062	12.31391813	-0.02811161	0.01196732	0.02261963	0.62870000
H	16.00888850	18.45901774	12.01701305	0.01157766	-0.02343018	-0.00529535	0.51580000
O	16.08938122	18.51958439	11.01970137	0.00401750	-0.00761455	0.02230289	-1.17760000
H	15.28959488	17.95539417	10.75699534	0.00236295	-0.00416718	0.03128651	0.61330000
H	15.63117835	15.53893644	20.58674699	-0.00213035	0.00091664	-0.01423751	0.58420000
O	15.70966540	15.87908461	19.66999816	-0.00177444	0.00447073	-0.02388232	-1.21080000
H	14.92625689	15.39947899	19.15849310	0.01068316	0.01137038	-0.03356559	0.64870000
H	15.14252689	20.99139196	17.13036159	0.01729937	-0.02856460	-0.01095893	0.58650000
O	15.93436389	21.30907884	17.63534407	-0.00674800	-0.02951890	-0.01236906	-1.18770000
H	16.58587697	20.56603862	17.55179114	-0.01892736	-0.01987282	-0.02348523	0.58390000
H	13.30537798	15.24747864	10.16333421	0.00579001	-0.00196286	0.01315961	0.58910000
O	13.12199716	14.27205948	10.24377604	0.00927378	0.01142934	0.02288547	-1.19350000
H	12.66553501	14.25861412	11.13328006	0.01442237	0.00681050	0.00476233	0.60800000
H	14.56295629	13.55601331	10.32669697	0.00596053	0.00781352	0.01487800	0.62690000
O	15.50544958	13.13360262	10.35367520	0.00007314	0.01713989	0.01700825	-1.18110000
H	16.07247806	13.85060142	9.99668785	-0.00384732	0.01338274	0.01456432	0.56500000
H	12.97957580	15.20338158	18.32018684	0.02043769	0.01129328	-0.02004191	0.61270000
O	13.72424296	14.63286104	18.66026790	0.00052012	0.00908484	-0.02588047	-1.21150000
H	13.81531969	13.90028339	17.99077527	0.01073302	0.02226420	-0.02126831	0.62050000
H	17.34408629	21.29400537	15.09344303	-0.01000841	-0.00952572	0.00202395	0.60480000
O	16.87282366	22.16958940	15.20002950	-0.00617571	-0.02244002	0.00062809	-1.21360000
H	16.60302740	22.11172715	16.16529919	-0.00095561	-0.01802081	-0.01431078	0.59150000
H	18.59166372	20.11136259	12.49809126	-0.01887858	-0.02042345	0.01117262	0.62740000
O	19.21306242	19.36044557	12.82042798	-0.01911419	-0.01847287	0.00049995	-1.18210000
H	20.11686099	19.69073108	12.63596019	-0.01501884	-0.00481593	0.00429903	0.57550000
H	14.49635958	16.33744468	11.42869792	-0.00055492	0.00498818	0.02172642	0.61460000
O	13.91003089	16.90491260	10.83110737	0.00543637	-0.00205825	0.02328942	-1.22290000
H	13.27273420	17.32708163	11.47397086	0.01499594	-0.01516129	0.01549191	0.60060000
H	13.73486886	21.23623685	15.44343636	0.01347990	-0.03390006	-0.00578459	0.63530000
O	13.34530999	20.72143291	16.30278955	0.00913418	-0.01947028	-0.00277579	-1.21270000
H	12.88084069	21.42489400	16.80832336	0.00860521	-0.01031809	-0.00505190	0.57610000
H	18.74077000	19.48148061	14.26179552	-0.00153581	-0.01444820	0.00891359	0.63620000
O	18.08273948	19.72123154	15.03954085	-0.02114096	-0.00398994	0.00464053	-1.22630000
H	18.35343967	19.31372592	15.89869799	-0.00461005	-0.01032103	-0.00148163	0.61720000
H	10.24655145	16.76403152	14.39755346	0.02314050	0.00463169	0.00454325	0.62650000

O	10.35069352	17.77598226	14.17371496	0.02325394	-0.00883089	0.01114061	-1.23050000
H	10.54410172	18.20089732	15.06551884	0.02015693	-0.01271394	-0.00173360	0.63160000
H	16.75039521	10.07290624	13.90758770	-0.00214637	0.01367121	-0.00025515	0.56950000
O	16.46949525	10.84441401	14.44179845	-0.01247261	0.02380544	0.00570530	-1.17520000
H	16.26123888	11.55398272	13.75476167	-0.00235841	0.01278535	0.01013988	0.61160000
H	17.06110579	13.34462860	13.02315769	-0.01834184	0.02216511	0.01357943	0.62470000
O	16.08760227	13.02308354	12.87095737	-0.00304589	0.01960874	0.02015157	-1.26270000
H	15.91777847	12.95394557	11.84692259	-0.00112782	0.01276697	0.01855349	0.62000000
C	13.59711790	16.14834801	15.18779554	-0.07444565	-0.04235032	-0.03152252	0.32580000
O	12.13852630	14.60882624	12.87211670	0.02604749	0.02590115	0.01236845	-1.17970000
O	13.09767325	15.04203007	15.68748265	-0.00729898	-0.00060842	-0.00374930	-1.12000000
H	13.08192650	17.04547666	15.65631055	-0.03387275	0.00911352	0.01511634	0.00640000
H	12.72750511	15.37037189	13.12519416	0.02101784	0.01985193	0.00776945	0.56190000
H	12.63236710	13.84806123	13.30884877	0.01660073	0.01917353	0.00445337	0.60440000

*CHO

117

Lattice="30.25322906 0.0 0.0 0.0 31.502680539999997 0.0 0.0 0.0 30.983304099999998" Properties=species:S:1:pos:R:3:forces:R:3

C	13.67210337	16.33221035	18.01593623	0.00410238	-0.01305473	-0.02299189
O	13.87430436	17.43260893	18.64099763	0.00509647	-0.00747966	-0.01231581
Cu	14.29463993	15.87717801	16.27844914	0.00184358	-0.00881640	-0.05070998
O	15.57664890	18.53697498	17.04915103	0.01691804	-0.01153929	-0.02140625
Cu	14.91191387	13.85967737	15.04044126	0.02807553	0.00008989	-0.01185539
O	13.69862860	12.44572418	15.90374323	0.01410267	0.01714289	-0.00774788
Cu	15.49322659	14.35364979	12.78057443	-0.02071564	0.00398722	0.05394323
O	15.71013046	15.09174077	10.89120829	0.00888440	-0.00366396	0.02969484
Cu	16.24876145	15.66347356	14.74037081	-0.01013514	-0.00361437	0.00498596
O	17.32579467	17.20224547	14.10764337	-0.01113833	-0.00903739	0.00512950
Cu	13.90662336	15.85769918	13.82136088	0.00733557	0.00290617	-0.00001432
O	12.71324587	16.96001959	12.58041747	0.02520231	-0.00744822	0.01928204
Cu	16.34106940	14.72906641	16.96740061	-0.07953355	-0.02469151	-0.04763907
O	17.48698851	14.97606603	18.67845984	-0.01749986	-0.00281801	-0.02425044
O	13.77438921	13.92718667	9.93499715	0.00297932	-0.00310046	0.01416408
O	15.75918895	14.86204202	20.44419358	-0.01040914	0.00333879	-0.01441016
O	13.32560645	18.58866386	10.73145240	0.01004186	-0.01023116	0.01097644
O	11.38638257	17.84372900	14.67953856	0.01173380	-0.00701713	0.00137097
O	13.46111965	19.12130682	15.43298303	0.01768627	-0.01687607	-0.01196771
O	18.72376590	16.96732854	11.91594301	-0.01669821	-0.01481206	0.01040626
O	11.73633913	14.95434840	11.08647892	0.01151478	0.00090409	0.02089172
O	14.54143370	12.91812716	19.05384495	-0.00437675	0.01110972	-0.02681298
O	12.56683259	11.71051040	13.67514924	0.01439661	0.01390631	0.00427100
O	10.91665111	13.59885887	13.31738131	0.02495388	0.00701843	0.00639407
O	10.51207392	15.32251085	15.05275831	0.02445213	0.00457438	-0.00530456
O	15.70288420	11.07539495	16.94281536	-0.00536830	0.01588027	-0.00823422

O	18.09676045	10.94047769	16.27310002	-0.00984060	0.01894659	-0.00985178
O	17.90989391	10.28855872	13.74160223	-0.01224978	0.02439820	0.00141288
O	11.73591320	14.14830987	17.09202125	0.02135086	-0.00147026	-0.01044741
O	13.34722108	20.58007062	12.51017543	0.00464568	-0.02190997	0.01426666
O	14.27093990	11.73096866	11.54773479	-0.00073718	0.00942667	0.01697516
O	12.16723032	11.70785027	18.33864132	0.01419116	0.01069659	-0.01778738
O	15.15753407	10.30215115	14.27019384	-0.00602294	0.02996492	0.00057842
O	17.10935232	11.70344522	11.51168020	-0.01083324	0.02214123	0.01122353
O	15.51266425	19.18614274	13.48479155	-0.00934839	-0.02947598	0.00093807
O	17.72617192	20.24828467	14.24927053	-0.00764676	-0.02169196	-0.00136959
O	18.13308173	19.38419026	16.67855229	-0.01071626	-0.02446936	-0.00839375
O	13.98751392	16.70891816	21.11307892	0.00023056	-0.00607617	-0.01033348
O	15.92615358	17.77559349	11.13875546	0.00223987	-0.01319649	0.02556721
O	20.29248068	16.00215959	13.89329972	-0.02734506	-0.00935709	0.01000888
O	18.90677333	16.56423448	16.66402572	-0.01862951	-0.00235777	-0.00847031
O	18.49344382	14.34221148	10.74698112	-0.00704945	0.00938262	0.01603911
O	18.69296580	13.79352277	13.28992238	-0.01780941	0.00160977	0.00720093
O	19.06145764	13.52365801	15.92040493	-0.01292996	0.01432593	-0.00429071
H	16.55688220	14.76390344	10.49550310	0.00200781	0.00379100	-0.00142009
H	14.80585973	14.57586261	10.35849991	-0.00545688	0.00605368	0.00095752
H	13.30415500	12.00028585	15.07360092	-0.00248820	0.00409334	0.00756971
H	14.42061686	11.84970421	16.30032739	-0.00437975	0.00694379	0.00225155
H	18.02150413	14.18539926	18.91421030	0.00007611	-0.00319888	-0.00359694
H	16.72401603	15.03339014	19.49594272	-0.00722281	0.00719174	0.00040031
H	12.52853088	13.75970484	16.62500942	0.02524448	0.01269238	-0.01699774
H	12.13919851	14.94343588	17.52344799	0.01059928	-0.01182695	-0.00657905
H	12.08510417	17.43486241	13.21801425	0.00158252	0.00826288	-0.00735320
H	13.00051456	17.65605846	11.83112140	0.01463227	-0.01030314	0.02137538
H	16.60210673	17.88465567	13.97092855	-0.00786099	-0.00141222	0.00380331
H	17.73325133	17.05005107	13.17599900	-0.00973741	-0.00206872	-0.00099289
H	13.30215809	19.44898784	11.26410475	0.00620457	-0.01649069	0.00687616
H	14.29892395	18.41648593	10.62220465	-0.00354775	-0.00582516	0.01507034
H	12.05837544	15.76428234	11.59089603	0.01894454	-0.00884143	0.01730183
H	11.44112303	14.37323778	11.84606246	0.02661431	0.00676564	0.01107316
H	10.68466876	18.52800027	14.67910807	0.01367193	-0.00546929	-0.00143204
H	12.23336785	18.31423984	15.09321466	0.01766560	-0.02442452	-0.00301556
H	18.13067855	13.94179389	15.95991794	-0.01319395	0.00460785	-0.01771748
H	19.62998029	14.28598488	16.18752833	-0.02459665	0.00440563	-0.00359686
H	18.99574510	13.39027632	14.15716873	-0.03024215	0.00462418	-0.00762596
H	17.71877022	13.99841424	13.49166916	0.01144349	-0.01007590	0.00896701
H	16.87473563	17.90121587	10.91160229	-0.01197770	-0.00961543	0.01425216
H	15.80996308	16.76317008	11.09946855	-0.00723412	-0.01402404	0.03734635
H	14.14000044	10.83565073	11.16545391	0.00246556	0.01114197	0.01400740
H	13.64867108	11.75209866	12.34808104	0.01688799	0.02865622	0.00951525
H	11.69498313	12.56890851	18.19660373	0.01003303	0.00197663	-0.00528470

H	12.54037304	11.58564322	17.43440559	0.00533195	0.00517284	-0.00422534
H	14.06046172	19.10985549	14.64066885	0.00583734	-0.03281444	-0.00166369
H	14.12318539	18.86565901	16.14448030	0.00574850	-0.02765777	-0.01639220
H	18.56051017	11.81620698	16.33779534	-0.01797280	0.00823309	-0.00713195
H	18.09189125	10.74406695	15.25472360	-0.01023962	0.01772425	0.00192753
H	13.85743995	17.13602892	20.19811217	0.00617095	-0.00184742	-0.00453146
H	14.43592080	17.41075999	21.62896543	0.00052109	-0.00282574	-0.00992327
H	13.65110715	12.46672361	18.91872795	0.01643450	0.01829243	-0.01608438
H	15.12899450	12.46494013	18.40157054	-0.00018510	0.03396830	-0.01821462
H	12.08994529	10.85464394	13.71371234	0.01026540	0.01109311	0.00322750
H	11.83927265	12.46472163	13.56041778	0.02546866	0.01758667	0.00550361
H	12.93283049	14.36694245	10.39758346	0.01785641	0.00095194	0.02218713
H	13.88106265	13.07105459	10.44230670	0.00847600	0.01779367	0.01984528
H	19.46827933	16.75405337	12.57356503	-0.02273032	-0.00598478	0.00114258
H	18.63877433	16.11299816	11.41187294	-0.01977269	-0.00110216	0.01748907
H	19.70432890	15.20419698	13.76590685	-0.02273632	0.00659287	0.00659607
H	19.96567274	16.38294501	14.74331434	-0.02123044	-0.00840770	-0.00720976
H	10.00121181	13.29130167	13.14481025	0.00964741	0.00505722	0.00510035
H	10.79022893	14.40295535	14.06953933	0.02897953	0.00726215	0.00311593
H	15.35343006	10.44815480	15.23707342	-0.00529537	0.02193122	-0.01268225
H	15.23270198	11.22570903	13.91925693	0.00466022	0.03227788	0.00538916
H	18.54437981	18.47816394	16.68115532	-0.01455411	-0.00530002	-0.01161898
H	17.20079990	19.18830922	16.98506926	-0.00431870	-0.01635074	-0.01266223
H	17.90398009	19.88914061	15.20819501	-0.00959298	-0.01483319	-0.00561212
H	18.41672193	19.79785443	13.71832920	-0.00648343	-0.01930946	0.00238373
H	10.95969082	14.92601433	15.86674887	0.02669580	0.00487429	-0.01321327
H	10.90721397	16.24379380	14.94568894	0.03077336	-0.01426184	-0.00066476
H	17.81900130	10.95133862	12.99416539	-0.00740248	0.01543819	0.01228931
H	16.95746802	10.00712549	13.83602838	0.00298911	0.01285618	-0.00113260
H	18.77482615	14.01284885	11.66272155	-0.01684085	0.00589694	0.01186423
H	19.19242014	14.03652019	10.13107693	-0.01084088	0.00263858	0.01429716
H	18.07598609	16.36531400	16.14043076	0.01442470	0.01267341	0.00279195
H	18.63380044	16.20131899	17.55002709	-0.01399230	0.00186825	-0.02025130
H	16.77509160	11.07237270	16.66884357	-0.00974070	0.02724374	-0.00943195
H	15.58083292	10.29478049	17.52547353	-0.00189263	0.01308516	-0.00660604
H	17.42214877	12.60519315	11.26923104	-0.02393844	0.00630470	0.02092532
H	16.13096002	11.82655567	11.64556525	0.00200616	0.02510999	0.01585890
H	15.11180442	15.61476006	20.69754496	0.00216747	-0.00851830	-0.02119221
H	15.18944487	14.14877775	20.02028677	-0.00128718	0.01380344	-0.01948951
H	13.52787318	21.51033546	12.26401864	0.00511339	-0.01141716	0.00443451
H	14.20662641	20.25721748	12.91697919	-0.00298532	-0.01310057	0.00325650
H	15.66610281	18.72261829	12.60537421	-0.00754303	-0.02240552	0.02009724
H	16.38940023	19.70427325	13.73798270	-0.01269999	-0.02107664	0.00399864
H	13.31541372	15.51585098	18.72909721	0.01045962	-0.02419847	-0.00071941
H	14.96220083	18.22726439	17.80558914	0.00934331	-0.01905702	-0.01494477

H 15.79457125 17.66004775 16.62616369 0.01982539 -0.01043735 -0.01239446

*CH_2O;*OH

117

Lattice="29.989484 0.0 0.0 0.0 32.096683 0.0 0.0 0.0 30.590058999999997" Properties=species:S:1:pos:R:3:forces:R:3:initial_charges:R:1

C 13.35087221 16.16764722 14.81647002 -0.00556502 0.00350029 -0.00714438 0.25480000
O 13.39218522 15.06042795 15.67280749 -0.02312754 -0.00770096 0.00346911 -1.08220000
Cu 15.18556869 17.57310924 16.27437664 0.03594252 -0.01018528 0.02413303 0.20530000
O 14.00095236 18.45702701 17.69111939 0.00528776 -0.01852768 -0.02389556 -1.21990000
Cu 17.32209488 16.63917797 15.45351876 -0.08038638 -0.01204696 -0.03307856 0.31990000
O 18.93255561 16.69424116 16.90796908 -0.02415865 0.00828581 -0.01424022 -1.21860000
Cu 16.21240621 18.89304615 14.49618765 -0.05964842 -0.01394965 0.02316229 0.09620000
O 18.02600674 19.68277318 15.08496339 -0.02111780 -0.00446500 0.00517919 -1.22280000
Cu 15.25747179 16.58006122 14.16281048 0.01127662 0.01107727 -0.01728863 0.29660000
O 15.56053534 15.60510939 12.37794830 -0.01455424 -0.00175335 0.02363793 -1.23660000
Cu 15.55329445 15.17877747 16.16488676 0.01907017 0.04444693 -0.01522718 0.37320000
O 16.35719822 13.48039363 16.88426293 -0.01919694 0.02142588 -0.01866889 -1.22410000
O 18.65844364 16.15159454 13.99439544 -0.02350647 0.01270210 0.01403818 -1.17820000
Cu 14.00952932 18.63575556 13.83662499 0.02113554 -0.00442468 0.04015664 0.15670000
O 12.26644632 18.31205173 12.86385168 0.03037921 -0.01131649 0.01881994 -1.18800000
O 18.32750427 13.78912085 13.47012859 -0.02308885 -0.00720703 -0.00142122 -1.19280000
O 10.26829196 17.71988953 14.14753581 0.02361687 -0.00985551 0.00931867 -1.23040000
O 18.15398196 12.27595415 15.63116535 -0.00783861 0.01500027 -0.00656596 -1.21680000
O 17.79355372 16.42532320 11.49102303 -0.01257334 -0.00069622 0.01635041 -1.21180000
O 19.01281000 19.28241185 12.82582027 -0.01742460 -0.01885995 -0.00079506 -1.18390000
O 13.57339069 12.90938095 14.38605082 0.01905518 0.01708761 -0.00644976 -1.23250000
O 11.56134126 15.81728849 17.28072836 0.01379865 0.02147890 -0.01549728 -1.20990000
O 10.03411008 15.23161217 14.80595231 0.03299149 0.00498403 0.00350147 -1.19530000
O 15.95203720 13.00758689 12.89809355 -0.00423568 0.01898571 0.02005968 -1.26390000
O 15.39239694 13.03279638 10.35442672 -0.00082586 0.01583596 0.01686557 -1.18040000
O 14.00693451 12.26837926 16.93485359 0.00255744 0.01258278 -0.01141679 -1.18720000
O 15.98078469 18.32919111 19.39077885 0.00191969 -0.00370482 -0.01572655 -1.20950000
O 15.60741967 15.83394621 19.71532540 -0.00124177 0.00407506 -0.02513189 -1.20630000
O 13.59284763 14.61411676 18.74231886 0.00283979 0.00800123 -0.02275433 -1.21250000
O 13.00120198 14.20276516 10.23673480 0.00792447 0.01049613 0.02486741 -1.18890000
O 16.21789213 10.81363698 14.47796230 -0.01198161 0.02478987 0.00613168 -1.17560000
O 17.49495653 21.34498662 12.29969559 -0.01552876 -0.01306567 0.01698797 -1.20040000
O 18.02008515 19.24418669 17.90707234 -0.02002718 -0.00891667 -0.01079787 -1.19620000
O 13.73517034 16.81895573 10.89928760 0.00372581 -0.00378579 0.02769338 -1.22860000
O 11.08529692 18.82372875 16.43884183 0.02818950 -0.00691986 -0.01057705 -1.21960000
O 18.02828283 14.85679354 18.55192754 -0.01959292 0.00677529 -0.00743002 -1.20140000
O 13.27347727 20.68052324 16.41729897 0.00967678 -0.01800371 -0.00226785 -1.21300000
O 14.16318941 22.06030815 14.50524553 0.01422734 -0.02009044 -0.00526484 -1.20660000
O 16.71533882 22.10471409 15.21421964 -0.00623893 -0.02081758 0.00107396 -1.22580000

O	11.58300738	18.35241263	19.04583186	0.01843332	-0.00791062	-0.01332011	-1.16940000
O	14.88618837	20.80266677	11.91772080	0.00212080	-0.01003234	0.00627058	-1.17850000
O	15.93698825	18.47566296	11.03575737	0.00314858	-0.00821054	0.02325746	-1.17680000
O	15.84874973	21.27239105	17.69287770	-0.00630643	-0.02965263	-0.01230760	-1.18510000
O	11.70310617	14.01690926	12.81531995	0.02108287	0.02109767	0.00986577	-1.16320000
H	19.53508753	16.25281003	14.42645403	0.00031487	0.00302728	-0.00720963	0.56610000
H	14.71529985	18.60974039	18.39722504	-0.00418598	0.00525737	-0.00513545	0.62000000
H	13.77965275	19.34792704	17.26367482	0.00242032	-0.00213150	-0.00890535	0.62620000
H	11.98143739	19.10903566	12.36461242	-0.00230211	-0.00132428	-0.00136626	0.57070000
H	11.30280098	17.97962640	13.48447255	0.01963233	-0.01078216	0.00965754	0.62440000
H	11.53547495	16.77140200	17.02196218	0.02459687	-0.00823226	-0.01511034	0.56800000
H	12.30699130	15.45506185	16.64311434	0.01884909	0.01246559	-0.00089526	0.61200000
H	15.59546958	12.82241928	16.96897057	0.00813972	-0.00429484	-0.00481917	0.61160000
H	17.13761601	12.98364271	16.30456846	-0.01019622	0.01247097	-0.01312322	0.62380000
H	15.63346378	14.62201124	12.61739788	-0.00159630	-0.00074131	0.00579532	0.61660000
H	16.51817469	15.88555209	11.99800537	-0.00062800	-0.00416770	0.01051294	0.62720000
H	17.54925241	11.58181478	15.21663069	-0.00467107	0.02050871	0.00175391	0.60070000
H	18.35203405	12.87236200	14.82918203	-0.01884932	0.01332055	0.01065850	0.61870000
H	17.51444004	14.22373322	17.96952565	-0.01371044	0.01584960	-0.01375976	0.61270000
H	17.26553819	15.28659790	19.02060797	-0.00992063	0.00917245	-0.02959529	0.58930000
H	13.88234941	11.32244180	17.15674542	0.00540663	0.01419885	-0.00926171	0.57930000
H	13.81718261	12.33902994	15.93386814	0.01045689	0.02058767	-0.00167614	0.62260000
H	14.89423117	20.16906676	12.72092631	-0.00231650	0.00513548	-0.00553539	0.48030000
H	15.10521171	20.11562989	11.21860638	-0.00447922	-0.00837912	0.02607352	0.59780000
H	16.51117966	21.17001448	12.11697918	-0.00270296	-0.01669721	0.02291662	0.61900000
H	17.46908381	21.93682186	13.08386850	-0.01074114	-0.01800990	-0.00053091	0.58610000
H	19.00343278	13.54576380	12.80396195	-0.01360695	0.01059947	0.00265465	0.55430000
H	18.52107399	14.95681334	13.74444006	-0.02132401	0.02098033	0.00875400	0.62130000
H	18.61586298	19.72213967	18.52148056	-0.01382465	-0.00754361	-0.01140006	0.58240000
H	17.32695180	18.80906555	18.50414899	-0.01308688	-0.01481415	-0.03034363	0.62630000
H	11.26709422	17.42678277	19.14131439	0.01217902	-0.00219944	-0.00626368	0.57960000
H	12.49980108	18.23470288	18.66739593	0.01158735	-0.01030848	-0.01313118	0.60120000
H	14.43983647	12.94460894	13.88975888	0.00897271	0.02900544	0.01166345	0.60730000
H	13.52787157	13.83604982	14.88549499	0.01441298	0.00054460	-0.00832775	0.61920000
H	14.04383603	21.71086630	13.59156139	0.00966423	-0.02369841	0.01383633	0.59990000
H	15.17691346	22.03337602	14.66031902	0.00044709	-0.02982515	-0.00505722	0.62070000
H	10.49676528	15.20088991	15.68505221	0.01325046	0.00041154	-0.01324653	0.59850000
H	10.58789042	14.66807638	14.19294164	0.01319470	0.01047045	0.01234121	0.60920000
H	11.06040868	18.76895305	17.44482385	0.01308441	-0.00057389	-0.01768334	0.59790000
H	11.82217426	19.46251406	16.25113823	0.01763846	-0.02362580	-0.01293312	0.59950000
H	15.85708568	18.79505197	20.24568160	-0.00194131	-0.00429788	-0.01475389	0.58440000
H	15.81379310	17.31027564	19.57772949	0.00159932	0.00011616	-0.03051434	0.64550000
H	18.60820575	15.95939994	17.55974210	-0.01125277	0.00376284	-0.00005331	0.62560000
H	18.75039048	17.52925950	17.40691973	-0.01073060	-0.00568198	0.00102248	0.57810000
H	17.43027602	17.31192368	11.22244904	-0.00603080	-0.00180728	0.03032808	0.60290000

H	18.25465148	16.57766398	12.37779995	-0.02759857	0.01282548	0.02196089	0.62660000
H	15.85738164	18.43157812	12.03529049	0.01282844	-0.02115528	-0.00888806	0.52090000
H	15.14511557	17.90604239	10.77638338	0.00357427	-0.00282515	0.03123069	0.60310000
H	15.54107919	15.49450478	20.63315685	-0.00217475	0.00102863	-0.01403304	0.58520000
H	14.80085676	15.36717346	19.21710111	0.01050771	0.01152621	-0.03331985	0.64240000
H	15.03880468	20.94359110	17.22439697	0.01694616	-0.02888043	-0.01165145	0.58230000
H	16.49523333	20.52553021	17.61167959	-0.01942248	-0.02007776	-0.02374287	0.58500000
H	13.13268028	15.18815538	10.33153049	0.00597876	-0.00180250	0.01517541	0.59200000
H	12.45207599	13.97190931	11.03307690	0.01414680	0.00769564	0.00413014	0.59580000
H	14.46121443	13.46634731	10.31438826	0.00600565	0.00728917	0.01421562	0.62540000
H	15.97207631	13.73748050	9.99488318	-0.00407819	0.01297380	0.01433413	0.56720000
H	12.85293959	15.16132927	18.34350779	0.01959301	0.01136884	-0.02012118	0.61480000
H	13.73000586	13.88177241	18.08323393	0.01072078	0.02105371	-0.02005064	0.61690000
H	17.21436072	21.24359308	15.12259330	-0.00713792	-0.00198487	0.00407234	0.60750000
H	16.46477831	22.06049219	16.18650579	-0.00123847	-0.01858239	-0.01490382	0.60170000
H	18.42604808	20.07893006	12.52633859	-0.02025309	-0.02048738	0.01192170	0.62930000
H	19.92709112	19.55257309	12.60140382	-0.01598339	-0.00511811	0.00489779	0.57290000
H	14.35494780	16.28409459	11.49728781	0.00118740	0.00529810	0.02625696	0.61370000
H	13.14350227	17.29508831	11.55090787	0.01595670	-0.01159406	0.02009483	0.60250000
H	13.63208943	21.19305832	15.53791628	0.01439057	-0.03457204	-0.00687073	0.63510000
H	12.80289728	21.37672788	16.92654069	0.00823082	-0.01050799	-0.00585493	0.57710000
H	18.64051267	19.42264346	14.27123910	0.00042956	-0.01347563	0.00821081	0.63440000
H	18.30992301	19.25502252	15.92945729	-0.00364865	-0.01037952	-0.00123341	0.61700000
H	10.18569787	16.70316660	14.36742929	0.02119896	0.00580974	0.00360094	0.63400000
H	10.45367941	18.14449772	15.04262578	0.02034836	-0.01244686	-0.00231461	0.62760000
H	16.52995827	10.04929522	13.94980412	-0.00220758	0.01409844	-0.00032326	0.56850000
H	16.07015334	11.53264908	13.78632327	-0.00254245	0.01278445	0.01070860	0.60930000
H	16.94359222	13.31063763	13.05447352	-0.02029913	0.02082337	0.01179611	0.62530000
H	15.79511566	12.90559751	11.87999143	-0.00088537	0.01171584	0.01770687	0.61960000
H	12.79403191	17.01339219	15.28098734	-0.01974572	-0.00655728	0.00902885	0.02220000
H	12.10035353	14.91827804	12.82320966	0.02300086	0.01891471	0.01083983	0.54440000
H	12.39233591	13.48532318	13.34739409	0.01242696	0.01499201	0.00270985	0.61090000
H	12.87887963	15.95937934	13.82794988	-0.00823999	0.01097076	-0.00449384	-0.00850000

*$CH_2(OH)_2$;*OH

117

Lattice="32.74738258 0.0 0.0 0.0 31.958319290000002 0.0 0.0 0.0 30.279041149999998" Properties=species:S:1:pos:R:3:forces:R:3

Cu	19.73617035	17.49091560	14.98932522	-0.09183557	-0.04137923	-0.00753834
Cu	17.47720010	17.45232788	15.93234654	0.00606871	0.01000106	-0.01652469
Cu	17.78770072	16.83909443	13.69719628	0.00176114	-0.02009975	0.02881587
Cu	16.37827752	15.38955763	14.96486135	0.03052176	0.00964781	0.01190953
Cu	18.77207624	15.36469849	15.52446189	-0.00456323	0.01219603	-0.01792266
Cu	15.49581162	17.54649380	14.36167157	0.03579051	-0.02812076	-0.00352501
O	21.56108099	16.39022805	14.74736008	-0.01865675	0.01306388	-0.01327867

O	16.77429963	18.34085509	17.54299681	0.00249343	-0.00992989	-0.02691894
O	14.15398106	18.34171270	12.54272818	0.01885510	-0.01147755	0.01833955
O	14.44763535	15.63809904	17.12575108	0.00676855	0.00559642	-0.01802455
O	19.49952243	13.62309240	16.18737703	-0.01697292	0.02214503	-0.01977485
O	18.53039764	15.89379932	11.99242645	-0.00504343	0.00157227	0.01826459
H	22.17346021	16.96614365	14.23266680	-0.00403357	-0.00975151	0.00039259
H	21.91692728	16.44672523	15.97600080	-0.01110665	-0.00818289	0.00497882
H	16.53234973	19.24459526	17.22606486	-0.01013156	-0.00544752	-0.00147037
H	14.33378641	19.30693260	12.57561694	0.00964368	-0.00468479	0.01393814
H	13.21688796	18.22939439	12.99143351	0.01532174	-0.00891547	0.00253332
H	15.05056006	16.12803945	16.48043100	0.00526208	0.00129887	0.00940425
H	13.83751389	15.15169382	16.50355355	0.02681829	0.01776247	-0.01582984
H	18.71559053	13.02822238	16.42692890	-0.00167240	-0.00282669	-0.00704557
H	20.20767212	13.03078488	15.65198038	-0.01424440	0.01154421	-0.00767701
H	18.52701697	14.98199235	12.40497647	0.00393045	0.00191054	0.01361155
H	19.52525766	16.09889263	11.86615157	-0.00731785	-0.00080966	0.01778838
H	20.56017578	11.54470736	14.52767457	-0.00446129	0.01859682	0.00247221
O	21.19591573	12.21208529	14.94665756	-0.00881698	0.01455330	-0.00495003
H	21.42889401	12.82795661	14.20094198	-0.01430203	0.00531437	0.00586386
H	20.38078592	14.23018944	17.56867572	-0.01149414	0.01673411	-0.01041286
O	20.79205046	14.75200875	18.31813604	-0.01890768	0.00882738	-0.01316552
H	19.96777658	15.10858680	18.76531237	-0.00590037	0.00835422	-0.02725087
H	17.22999794	11.44137538	17.02347170	0.00273620	0.01202380	-0.01026033
O	17.20701773	12.38404299	16.75538574	0.00516749	0.01689737	-0.00965479
H	16.81634629	12.38669609	15.79813401	0.00948395	0.02700995	-0.00496709
H	16.85394592	18.99019743	13.01408286	0.00881963	0.01767985	0.00418209
O	16.83679776	19.77668803	12.36170114	0.00044685	-0.01772315	0.00804280
H	16.60530303	19.32999834	11.51145353	0.01015137	-0.00640848	0.02146331
H	18.77286778	19.81531620	12.34089087	0.00819465	-0.01864999	0.02445158
O	19.55964785	19.20342745	12.33353584	-0.00913449	-0.00557843	0.01061315
H	19.26402716	18.53037491	13.04166506	-0.00066260	0.00675044	-0.00594803
H	21.35271771	14.77788108	12.30240570	-0.01377915	0.00111023	0.01812334
O	21.13428488	14.34838457	13.16544825	-0.02221953	0.01321645	0.00089352
H	21.26547649	15.13158764	13.84189891	-0.03896139	0.01673878	0.01388717
H	21.26451613	19.40749560	18.16494200	-0.01190895	-0.00466021	-0.01010019
O	20.80323291	18.75757819	17.59111859	-0.00096794	-0.00216444	-0.00100138
H	19.66250295	18.41380605	18.33421133	-0.02762798	-0.02343093	-0.01615088
H	13.96794861	17.21659469	18.02754222	0.01410149	0.00345228	-0.01454169
O	14.16560807	18.18541489	18.08926920	0.01199855	-0.00501977	-0.01197577
H	15.18052005	18.16868148	18.07346362	0.01676231	-0.01065240	-0.02517023
H	17.08687869	12.88459863	13.82474420	0.00319228	0.03113330	0.00765513
O	16.35606090	12.41974663	14.31525070	0.01365475	0.01556389	-0.01237847
H	15.54622073	12.98677245	14.18012360	0.00793423	0.00686194	0.00107418
H	16.67420690	21.23270079	13.38741318	0.00421294	-0.01618423	0.01254670
O	16.76159992	21.88234458	14.13292926	0.00430627	-0.02390674	0.00101701

H	17.77822864	21.88155257	14.33963498	-0.00542910	-0.01872190	0.00093365
H	11.06703027	15.54799976	15.16682001	0.00267843	0.00757990	0.00493663
O	10.41122486	16.19830850	14.80365256	0.01898748	-0.00530664	-0.00091507
H	9.98596133	15.71225454	14.06550549	0.01012770	-0.00151926	0.00084846
H	14.10155007	18.46477536	16.54847732	0.01740948	-0.00830874	-0.01300534
O	14.16420093	18.74590343	15.54117791	0.02201924	-0.01753031	-0.01261843
H	14.69158124	19.60581618	15.65043305	0.00554273	-0.00738118	0.00088406
H	17.85579699	18.37405514	18.32123432	0.00670890	-0.00597689	0.00135615
O	18.77927527	18.20689380	18.95106514	0.00164273	-0.00671864	-0.01579985
H	18.71387423	17.20486041	19.10338339	0.00145454	0.00460216	-0.03514866
H	21.60443553	15.83820008	17.62211389	-0.02141732	0.01258740	-0.02068674
O	22.15190784	16.58122767	17.12224972	-0.01451751	0.00334612	-0.00492996
H	21.65496401	17.46190764	17.37551012	-0.02162550	-0.01659997	-0.01945605
H	20.44602514	16.94929108	10.51248098	-0.00202113	-0.00319012	0.02260972
O	20.98720371	16.41209707	11.18542280	-0.01029561	0.00900209	0.02185626
H	21.46620502	17.11484735	11.70726978	-0.02114306	-0.00614778	0.01565207
H	19.23093670	18.47543450	10.78030682	-0.00898349	-0.01373579	0.01688367
O	19.19144336	17.95669462	9.92224686	-0.01323690	-0.00354314	0.02686574
H	18.36962448	17.42761408	10.03532625	-0.00868192	-0.00351476	0.02713693
H	18.33481322	15.43305547	20.31435592	-0.00306934	0.00058490	-0.01192942
O	18.42076774	15.61578955	19.35449380	-0.00095142	0.00517172	-0.02597931
H	17.57692027	15.13981578	18.93692913	0.00731530	0.01289245	-0.03148380
H	17.45428712	20.83870464	16.86677182	0.00981248	-0.02615794	-0.00757706
O	18.40899967	20.81767105	17.15524299	-0.00063033	-0.02642367	-0.00837871
H	18.69561950	19.91339953	16.88466506	-0.00563713	-0.02285163	-0.02448548
H	15.64271624	15.50724093	11.28117129	0.01284232	0.00602667	0.03084193
O	15.74206811	14.61954107	11.77195621	0.01513197	0.01025376	0.01796905
H	16.17350759	14.97235933	12.61734815	0.00175079	0.00181548	-0.00541678
H	16.95800837	13.77125333	10.79747332	0.00668835	0.00709740	0.01527868
O	17.77344236	13.37026537	10.36609751	0.00009042	0.01721537	0.01972846
H	18.22002564	14.16941762	10.01023239	-0.00048132	0.01596036	0.01710760
H	15.67120989	14.82940388	18.02234391	0.01875332	0.01158098	-0.02401729
O	16.38934404	14.33996666	18.52194968	0.00609110	0.00890932	-0.02482354
H	16.66892914	13.62272025	17.88444160	0.00744427	0.02358150	-0.02189648
H	19.80263415	21.09538078	14.61848220	-0.01152549	-0.02133089	-0.00106524
O	19.26457103	21.88879614	14.87210307	-0.00126068	-0.02889764	-0.00300397
H	19.02872410	21.62491931	15.82180017	-0.00159395	-0.01642884	-0.00637887
H	21.27081218	19.04834983	12.56410208	-0.01530705	-0.01302863	0.01155436
O	22.13795057	18.56630967	12.73164561	-0.01768658	-0.00753217	-0.00407758
H	22.80338919	19.05901475	12.20655087	-0.01545192	-0.00652785	0.00668354
H	16.51298908	17.11571615	11.80549050	-0.00499697	-0.00061301	0.00193890
O	15.96401670	17.17294601	10.96480049	0.01713127	-0.00722559	0.01180338
H	15.13293666	17.58364796	11.36070187	0.01428467	-0.01162755	0.02756069
H	16.17736228	21.28026148	15.24761537	0.00730521	-0.02461393	-0.00348061
O	15.78154651	20.78805944	16.14478528	0.00910236	-0.02017227	-0.00534723

H	15.27427622	21.47865461	16.62315347	0.00738908	-0.01359673	-0.00441005
H	21.61573191	19.23550286	14.74580092	-0.00311213	0.00828497	0.01346586
O	20.73357853	19.37710727	15.16093548	-0.01729925	-0.02252313	-0.00866042
H	20.85074532	19.22533863	16.22147376	-0.00654194	0.01065104	-0.01358394
H	11.43177362	17.49123627	14.22287298	0.02212907	0.00184919	0.00174356
O	12.01636766	18.25307867	13.93411226	0.01059902	-0.01606591	0.00857980
H	12.59064963	18.47812545	14.72098796	0.01691791	-0.01680771	-0.00103792
H	19.32102159	10.08347068	13.30035779	-0.00291359	0.01342945	0.00121023
O	19.17397042	10.79612777	13.95550152	-0.01263002	0.02304447	0.00795803
H	18.92516780	11.59839094	13.40082968	-0.00336506	0.01170721	0.01182727
H	19.66552931	13.60412993	12.93161328	-0.02277909	0.02073562	0.01653046
O	18.73511138	13.20331249	12.79698086	-0.00212072	0.01949087	0.02034299
H	18.52929372	13.16043473	11.79486601	0.00046750	0.01367367	0.02188341
C	13.13249229	14.80217568	13.90179433	0.02023960	0.01903597	-0.00224705
O	13.96216323	13.74279905	13.50508195	0.02037310	0.00053184	-0.00260558
O	12.68592540	14.56255088	15.25606393	0.01499531	0.00820781	0.00780264
H	13.67522897	15.77675680	13.93988499	0.01063105	0.01446768	0.00307674
H	14.51199687	14.07131037	12.70772108	0.01531804	0.01196896	0.01395515
H	12.23836395	14.88325473	13.24153413	0.00174792	0.01753052	-0.00704247
H	12.51320003	13.59376956	15.31987315	0.00590731	0.00625081	0.00387878

*CH_2OH;*OH

118

Lattice="29.892981 0.0 0.0 0.0 32.0554189 0.0 0.0 0.0 30.638273599999998" Properties=species:S:1:pos:R:3:forces:R:3

C	13.10977922	16.16321055	14.68742509	-0.00727215	0.00432393	-0.01065952
O	12.90498323	14.93377261	15.55185567	-0.00364085	-0.00842802	0.00391706
Cu	15.21117454	17.48402419	16.43202482	0.01747831	-0.02830279	0.02566689
O	13.99785741	18.45684706	17.76081157	0.00439933	-0.01769406	-0.02306742
Cu	17.30388100	16.69972300	15.37757995	-0.08044822	0.00215889	-0.03390887
O	18.95299424	16.69433519	16.90191856	-0.02374559	0.00854215	-0.00804129
Cu	16.15839309	18.77629291	14.55657608	-0.04443725	-0.01125061	0.03132652
O	17.99781418	19.69514186	15.07294373	-0.02085447	-0.00255151	0.00558542
Cu	15.06162294	16.44972717	14.34932787	0.00073658	-0.01350321	-0.02269480
O	15.55857260	15.46934145	12.51129968	-0.01412969	-0.00383613	0.02126282
Cu	15.81717021	15.15821933	16.34501272	-0.02968377	0.04449958	-0.03896522
O	16.37169889	13.38761666	16.96737233	-0.01920277	0.01999145	-0.01865477
O	18.75435252	16.04140332	14.05538018	-0.02588225	0.01288583	0.01392309
Cu	13.95087716	18.58556340	13.88861125	0.01160428	0.00058084	0.04075940
O	12.15849130	18.41785991	12.88485666	0.02984863	-0.00816934	0.01829454
O	18.20978201	13.66480475	13.48390518	-0.00658763	0.00296478	0.00191268
O	10.10348741	17.69862908	14.10969735	0.02159332	-0.01236532	0.01380989
O	18.06195414	12.19847362	15.61574950	-0.00958036	0.01432780	-0.00545249
O	17.73651113	16.42650957	11.50403917	-0.01305958	-0.00036485	0.01863996
O	18.94622436	19.26413012	12.77079319	-0.01950176	-0.01806352	0.00039197

O	13.76496148	12.81132475	14.33192900	0.01929852	0.01841225	-0.00221066
O	11.30042128	16.01335171	17.32933400	0.01674917	0.01538351	-0.02413868
O	9.86152251	15.09797981	14.69745475	0.02483603	0.00884978	0.00299789
O	15.95375991	12.80488763	12.88860761	-0.00453405	0.01605339	0.01407828
O	15.36796468	13.00134049	10.31866057	-0.00111019	0.01533796	0.01858689
O	13.94601611	12.23557443	16.93247929	0.00311919	0.01644467	-0.01418365
O	15.95662284	18.39110051	19.53649101	0.00134817	-0.00374528	-0.01496462
O	15.55283815	15.81968819	19.74116993	-0.00174320	0.00549114	-0.02303021
O	13.55024585	14.45639377	18.74571219	0.00134345	0.00933267	-0.02563370
O	12.94248314	14.10494643	10.21642348	0.00615053	0.00831195	0.02508956
O	16.16271771	10.62117602	14.50355180	-0.01101373	0.02568318	0.00586786
O	17.59299432	21.45126923	12.29351236	-0.01468402	-0.01269868	0.01650677
O	17.97587214	19.21819050	17.94075109	-0.02115184	-0.00969916	-0.00970759
O	13.63754515	16.61946850	11.10080430	0.00260751	-0.00470926	0.02621950
O	10.92094715	18.79643743	16.51786411	0.02855494	-0.00901478	-0.01127976
O	18.00653328	14.85999046	18.61470746	-0.01366175	0.00665646	-0.00276293
O	13.22775173	20.61621034	16.44027892	0.00874046	-0.01684665	-0.00167418
O	14.10515466	22.01635252	14.54682929	0.01312095	-0.01876958	-0.00373609
O	16.66085540	22.10292805	15.23514009	-0.00613573	-0.02390420	-0.00245356
O	11.53490912	18.24944402	19.12072971	0.01917397	-0.00637096	-0.01345727
O	14.98127008	20.82029243	12.10357260	0.00396368	-0.00816755	0.00671752
O	15.76705743	18.42124438	11.08846518	0.00398528	-0.00808931	0.02117815
O	15.84369417	21.23268690	17.70025461	-0.00718187	-0.02916887	-0.01200177
O	11.69723383	13.80742582	12.90257855	0.02057139	0.01977204	0.00924287
H	19.55411544	16.01605020	14.62563719	0.00131565	-0.00440793	-0.00901468
H	14.67008979	18.67358978	18.48120303	-0.00464250	0.00790546	-0.00447387
H	13.76335734	19.32335312	17.28325314	0.00177152	-0.00205154	-0.01085252
H	11.89107461	19.21645638	12.38114134	0.00308726	-0.00377680	0.00171587
H	11.26228419	18.07682217	13.41187227	0.01742949	-0.00787626	0.00695137
H	11.23235650	16.93445067	16.95514750	0.02037576	-0.00818954	-0.01425460
H	12.04223626	15.63150916	16.75942269	0.02376691	0.01451492	-0.00274252
H	15.56439776	12.79043837	17.02408088	0.00673187	-0.00605129	-0.00559962
H	17.12487953	12.86327257	16.32917609	-0.00727305	0.00308880	-0.01421173
H	15.66023159	14.48710969	12.70745304	0.00223257	0.00751061	0.00790251
H	16.47578689	15.78633117	12.12891085	-0.00560258	-0.00143983	0.02101969
H	17.47451312	11.47347585	15.23286364	-0.00538786	0.02115842	0.00139172
H	18.22470923	12.79595610	14.78598706	-0.01882459	0.01201884	0.01487943
H	17.55836681	14.14882059	18.07660452	-0.00106190	0.00842810	-0.00077089
H	17.20286966	15.28281328	19.01887375	-0.01027481	0.01002038	-0.03271302
H	13.88190350	11.29092922	17.18050269	0.00546221	0.01347383	-0.00921740
H	13.86997870	12.25645224	15.91706051	0.01037608	0.01882791	-0.00270424
H	15.03666016	20.16437482	12.89117744	0.00069210	0.01159151	-0.00684165
H	15.08577625	20.14442784	11.37139868	-0.00417692	-0.00877770	0.02598556
H	16.60715367	21.25177945	12.16399389	-0.00218485	-0.01517345	0.02074752
H	17.59169885	22.02355800	13.09321891	-0.00931379	-0.01568272	0.00081842

H	18.88543185	13.36160535	12.84197590	-0.01255158	0.00847651	0.00112058
H	18.52787101	14.99777390	13.81268063	-0.01614974	0.01937679	0.00847361
H	18.59014676	19.70298266	18.53341760	-0.01354915	-0.00785466	-0.01103391
H	17.29629311	18.80907489	18.56663338	-0.01344114	-0.01546166	-0.03015089
H	11.28434242	17.30600035	18.97201575	0.01353339	0.00246192	-0.00146017
H	12.45585480	18.25303596	18.73868597	0.00952586	-0.01071164	-0.01330074
H	14.62371694	12.78795645	13.77989188	0.00445300	0.02610327	0.01578114
H	13.77614789	13.72288714	14.76051815	0.02938510	0.02302814	0.00542709
H	14.03495315	21.64064053	13.63615392	0.00874231	-0.02482131	0.01234534
H	15.11708013	22.03330796	14.72280129	0.00044096	-0.02817906	-0.00452842
H	10.21589231	15.10261933	15.62106527	0.00808956	0.00072048	-0.01164449
H	10.45068113	14.50733156	14.14674607	0.00889271	0.00919503	0.00915244
H	10.95936984	18.77256527	17.52728648	0.01215723	-0.00098640	-0.01701749
H	11.71443991	19.34304403	16.27596999	0.01606711	-0.02463997	-0.01295966
H	15.85732889	18.80347216	20.42045916	-0.00124400	-0.00473174	-0.01481149
H	15.78238530	17.38174796	19.66830188	0.00156178	-0.00046061	-0.02981341
H	18.61272100	15.96788399	17.53970652	-0.01648621	0.00484066	-0.00611712
H	18.73690880	17.52748593	17.39242750	-0.01389791	-0.00838169	-0.00124398
H	17.30130687	17.28280577	11.24150918	-0.00398926	-0.00129940	0.02953517
H	18.24800831	16.64528321	12.32893150	-0.02526274	0.01250564	0.02207378
H	15.71239609	18.36022753	12.09142015	0.01387769	-0.02383516	-0.00908963
H	15.00364998	17.81704688	10.84169704	0.00395721	0.00053710	0.03007704
H	15.50781717	15.41958177	20.63632865	-0.00195291	0.00086264	-0.01462761
H	14.79801543	15.33078320	19.22674243	0.01209378	0.01224391	-0.03247893
H	15.04586819	20.88994481	17.22211838	0.01746457	-0.02859857	-0.01159929
H	16.50279082	20.49443739	17.62595353	-0.01923517	-0.02078133	-0.02310440
H	13.07001216	15.07340641	10.44482935	0.00622123	-0.00112198	0.01479183
H	12.38918589	13.76857097	10.96355929	0.01295254	0.00699895	0.00242968
H	14.43201940	13.41313896	10.28023584	0.00602949	0.00721073	0.01472867
H	15.94096814	13.73133291	10.00152900	-0.00427544	0.01249938	0.01495714
H	12.81964962	15.03250728	18.41263931	0.02156104	0.00857111	-0.01726930
H	13.66691296	13.76661550	18.03559455	0.01031006	0.02161957	-0.02056322
H	17.13728562	21.23303485	15.11289106	-0.01050769	-0.00898719	0.00129189
H	16.43660088	22.04059675	16.21299651	-0.00125561	-0.01823065	-0.01462055
H	18.43733352	20.12218318	12.49857157	-0.01937213	-0.01956620	0.01114620
H	19.87979874	19.44568661	12.53689200	-0.01540367	-0.00479826	0.00492441
H	14.30510934	16.14552177	11.70449390	-0.00076187	0.00452862	0.02102710
H	13.07498238	17.15577267	11.72317344	0.01320534	-0.01313850	0.01495690
H	13.58623865	21.14411742	15.56509476	0.01419082	-0.03358722	-0.00726273
H	12.75145981	21.30083942	16.95973785	0.00838589	-0.01084197	-0.00577284
H	18.58866286	19.42992446	14.25321381	-0.00083422	-0.01192080	0.00797579
H	18.31953287	19.27262197	15.90403267	-0.00346820	-0.00739173	-0.00283262
H	10.02701321	16.69738523	14.27067018	0.02130943	0.00526064	0.00490276
H	10.24599972	18.08040130	15.02133707	0.01734351	-0.01237986	-0.00270527
H	16.49227809	9.83571989	14.01924553	-0.00209077	0.01386640	-0.00053377

H	16.05708086	11.31790549	13.78197667	-0.00272422	0.01208738	0.00944863
H	16.97058353	13.17842444	13.07927006	-0.03831373	0.01155719	0.00309058
H	15.78115056	12.77225556	11.87630609	-0.00018592	0.00951286	0.01633418
H	12.12351293	14.68454270	12.76009649	0.02156990	0.01554965	0.00837163
H	12.44774202	13.29193757	13.35407473	0.01032762	0.01449084	-0.00226542
H	12.50666127	16.00404272	13.76704217	0.00365674	0.01479350	-0.00592813
H	12.07649472	14.49927789	15.23224113	0.02801123	0.00738786	0.01471213
H	12.56369544	16.97365071	15.22045132	-0.00284334	0.00101845	0.01834792

*CH_2O

118

Lattice="30.27574 0.0 0.0 0.0 31.377029 0.0 0.0 0.0 30.874285" Properties=species:S:1:pos:R:3:forces:R:3

C	14.05772324	16.06558248	18.19507480	0.00328308	-0.00938390	-0.01905765
O	12.79358750	16.45964501	18.42167776	0.00316122	0.01001869	-0.01516945
Cu	14.55834591	15.95873319	16.27057035	-0.02294217	-0.01433291	-0.06969918
O	15.56982241	18.71741874	17.21058461	0.00852627	-0.01749306	-0.01380519
Cu	14.96075646	13.85053464	15.13572187	0.02305221	0.00034989	-0.00232887
O	13.74329667	12.45467165	15.99249204	0.01546077	0.01743109	-0.00867165
Cu	15.49826364	14.39335574	12.84679697	-0.01730886	-0.00032904	0.05292905
O	15.73393544	15.02088767	10.96802765	0.00905246	-0.00349492	0.02985629
Cu	16.47002330	15.53454072	14.76450969	-0.00317753	-0.00192537	0.01147079
O	17.46448507	17.12830048	14.07889319	-0.01131844	-0.00885128	0.00898108
Cu	13.95407877	15.82789070	13.94076989	0.00348643	-0.00137076	0.00295093
O	12.70978198	16.91555536	12.76412976	0.02502681	-0.00768477	0.01847429
Cu	16.56201278	14.50605818	16.92765976	-0.06984402	-0.04095967	-0.04428750
O	17.45831264	14.91665020	18.78727232	-0.02084725	-0.00341329	-0.02431566
O	13.76191526	13.87424241	10.09853770	0.00306449	-0.00295156	0.01299302
O	15.73238192	14.84651487	20.59607744	-0.01353822	0.00539436	-0.01785213
O	13.33212500	18.50918877	10.88420550	0.01042103	-0.01101273	0.01082450
O	11.37366145	17.83684203	14.82586341	0.01294194	-0.00886217	0.00208237
O	13.35246779	19.28097069	15.60553191	0.01858260	-0.01826798	-0.01211394
O	18.89415357	16.90020978	11.90552013	-0.01877444	-0.01303642	0.00881814
O	11.75988981	14.88643785	11.26251211	0.01164174	0.00126548	0.02087017
O	14.60816065	12.83819841	19.19338899	0.00100936	0.00773152	-0.02559240
O	12.62322183	11.67518801	13.72082685	0.01490912	0.01369912	0.00362537
O	10.96474667	13.54279794	13.47921374	0.02402469	0.00662687	0.00418351
O	10.59889687	15.22892633	15.22591336	0.02506606	0.00566964	-0.00660677
O	15.71961304	11.03271803	17.05236340	-0.00516651	0.01581976	-0.00849330
O	18.11857801	10.87032733	16.41911590	-0.00932573	0.01892469	-0.00986176
O	17.92367615	10.24096095	13.88581597	-0.01178228	0.02489008	0.00164416
O	11.76859386	14.41544848	17.37692473	0.02124173	-0.01080900	-0.01278466
O	13.35026871	20.49242412	12.66551414	0.00432203	-0.02185053	0.01520740
O	14.31305991	11.68519692	11.67576113	0.00079104	0.00981160	0.01597251
O	12.16574122	11.90587175	18.43984944	0.01479451	0.01182083	-0.01767747
O	15.17561771	10.23772231	14.41237714	-0.00548913	0.03036880	-0.00042557

O	17.11808319	11.67036310	11.67103672	-0.01010851	0.02321109	0.01198930
O	15.51481775	19.10453221	13.65707802	-0.00945789	-0.02943518	0.00125617
O	17.74630359	20.15448446	14.40882410	-0.00849639	-0.02263032	-0.00025656
O	18.30219614	19.35822602	16.83870684	-0.01351556	-0.02089981	-0.00723265
O	13.73447035	16.68069711	21.01449496	0.00412486	-0.00579713	-0.01405039
O	15.94458584	17.69890426	11.29348736	0.00287541	-0.01337585	0.02603607
O	20.29970542	15.92992485	14.03398399	-0.02879697	-0.00985715	0.00995052
O	18.80045056	16.65475579	16.83074394	-0.01422423	-0.00417100	-0.00811742
O	18.51681462	14.27527597	10.90352795	-0.00701993	0.00964719	0.01732895
O	18.73220356	13.68979852	13.45229356	-0.01570724	-0.00119146	0.00351461
O	19.28558335	13.34591489	16.04564839	-0.01223159	0.01468685	-0.00420655
H	16.58440671	14.68482945	10.59031102	0.00350087	0.00375097	-0.00278526
H	14.75275217	14.47879431	10.44412468	-0.00732137	0.00420697	0.00010931
H	13.34453768	12.00881827	15.17046827	-0.00292367	0.00504031	0.00821382
H	14.43852008	11.83381663	16.38859524	-0.00296842	0.00605348	0.00290268
H	18.02582730	14.17359436	19.09086115	-0.00240069	-0.00107502	-0.00290885
H	16.70487664	14.99283156	19.57688850	-0.00724435	0.00492330	-0.01223946
H	12.59151039	14.11742063	16.91516464	0.02311181	0.00760992	-0.01806288
H	12.10690406	15.31300748	17.88549515	0.01742530	-0.00694325	-0.01662439
H	12.08920949	17.40187927	13.40506699	0.00250929	0.00834554	-0.00727853
H	12.99774102	17.60117669	12.00863262	0.01380292	-0.00860355	0.01877412
H	16.70531322	17.76694647	13.95825030	-0.00900308	-0.00394577	0.00248885
H	17.87343949	16.99542513	13.14285147	-0.01222953	-0.00324968	0.00076977
H	13.30474176	19.38307353	11.38792416	0.00633851	-0.01523118	0.00702306
H	14.30599075	18.33358825	10.78313771	-0.00348425	-0.00694288	0.01546502
H	12.06839105	15.70097320	11.76913615	0.01920462	-0.00902906	0.01652012
H	11.45185640	14.30413344	12.02375157	0.02677503	0.00725668	0.01053894
H	10.61902061	18.46120046	14.81139600	0.01313756	-0.00534125	-0.00094488
H	12.16115175	18.37057292	15.25165865	0.01609651	-0.02362451	-0.00388098
H	18.40078599	13.86824044	16.12618519	-0.01573303	0.00974169	-0.01512970
H	19.96670428	14.01403987	16.29041330	-0.02484053	0.00968161	-0.00273238
H	19.10365581	13.28044366	14.29214970	-0.03115522	0.00515806	-0.00635037
H	17.79263599	13.92400174	13.75030973	0.00911036	-0.01334519	0.01194397
H	16.86977592	17.85191508	10.99771228	-0.00866121	-0.01211182	0.01624230
H	15.84133298	16.68433085	11.22322178	-0.00570750	-0.00959254	0.03405940
H	14.20159549	10.79992970	11.26577330	0.00207244	0.01056520	0.01364884
H	13.65652629	11.68629519	12.46074378	0.01612910	0.02782833	0.00934826
H	11.80036458	12.81835988	18.23783072	0.01283147	0.00570523	-0.00814012
H	12.47742377	11.65854848	17.54004818	0.00532766	0.00787448	-0.00476611
H	14.00925982	19.15985464	14.87024028	0.00511538	-0.03051821	-0.00245752
H	13.91180038	19.05280107	16.39571460	0.00052208	-0.02334954	-0.01402260
H	18.61805099	11.72682142	16.48025078	-0.01855707	0.00937203	-0.00772664
H	18.10999780	10.67640958	15.40158462	-0.01004441	0.01882581	0.00223806
H	13.17680643	16.67235062	20.17744361	0.00127077	-0.00530344	-0.00429732
H	14.03017726	17.61263706	21.08184180	0.00203583	-0.00498505	-0.01204613

H	13.68207095	12.47240940	18.99650293	0.01603766	0.01780230	-0.01550918
H	15.17943574	12.41002711	18.51051280	0.00174634	0.03455257	-0.01790106
H	12.16389535	10.81238043	13.79531246	0.00977321	0.01058287	0.00317610
H	11.87239117	12.43367107	13.64071678	0.02513963	0.01656002	0.00381408
H	12.91887324	14.32299112	10.59589674	0.01853357	0.00138826	0.02314384
H	13.89056226	13.01439662	10.60200857	0.00880307	0.01889315	0.02037624
H	19.59890898	16.66085899	12.59365576	-0.02199307	-0.00610370	0.00034142
H	18.75699826	16.03234147	11.43502548	-0.02042936	-0.00040755	0.01819177
H	19.70515295	15.13700825	13.90468068	-0.02332025	0.00651484	0.00725556
H	19.88306661	16.38624579	14.80256789	-0.02456233	-0.00983305	-0.00474051
H	10.04647636	13.22119296	13.35442944	0.00987758	0.00498511	0.00481212
H	10.86248499	14.35955487	14.29662229	0.02924902	0.00974034	0.00408938
H	15.36941055	10.40984397	15.37744717	-0.00456435	0.02233372	-0.01237283
H	15.22698450	11.14942163	14.03790149	0.00553884	0.03376420	0.00616049
H	18.56208762	18.38820274	16.83836539	-0.01476544	-0.00654278	-0.01271294
H	17.39292782	19.31984434	17.23217736	0.00079621	-0.01238569	-0.00909033
H	17.96494835	19.83398471	15.37088174	-0.00911786	-0.01475749	-0.00552377
H	18.42428388	19.68169576	13.88024921	-0.00612638	-0.01979025	0.00265859
H	11.03388447	14.90940963	16.11716898	0.02771089	0.00357958	-0.01196431
H	10.96430555	16.15202825	15.08400261	0.03059893	-0.01311048	-0.00055367
H	17.83333644	10.90885047	13.14421542	-0.00725398	0.01556215	0.01236013
H	16.97078212	9.96048404	13.98072944	0.00308846	0.01316207	-0.00089082
H	18.78119069	13.95589771	11.82915091	-0.01841324	0.00723934	0.01297222
H	19.20849872	13.92813099	10.30144244	-0.01048760	0.00255988	0.01392751
H	18.04109414	16.28306470	16.28371221	0.00719829	0.01657050	0.00038761
H	18.57532968	16.25137546	17.71091678	-0.01386488	-0.00027220	-0.01998225
H	16.79546772	11.02658698	16.79858067	-0.00924274	0.02798936	-0.00971192
H	15.58856937	10.26374619	17.64875426	-0.00095905	0.01295303	-0.00691921
H	17.42829665	12.57178403	11.42529960	-0.02413656	0.00668608	0.02170805
H	16.13369043	11.77755899	11.77319592	0.00115469	0.02842698	0.01759851
H	15.04551376	15.57255815	20.74739833	0.00467725	-0.01115584	-0.01372774
H	15.20479781	14.08721157	20.19415455	-0.00034030	0.01052025	-0.01533617
H	13.43215274	21.46316458	12.57427390	0.00477014	-0.01273130	0.00455739
H	14.21657989	20.19530001	13.07303890	-0.00308269	-0.01304340	0.00350687
H	15.65249163	18.65867017	12.76734687	-0.00747775	-0.02368911	0.02028844
H	16.39449150	19.61444547	13.90317448	-0.01320718	-0.02094338	0.00398736
H	14.27902138	14.98469747	18.38225396	0.00947566	-0.01682364	0.00342382
H	14.84680686	16.68018004	18.70485904	0.02608150	-0.00485478	0.00250744
H	15.28882352	18.07275868	17.90564259	0.00782537	-0.01736255	-0.00820019
H	15.77123384	18.07629165	16.47639175	0.01943855	-0.01128741	-0.02235289

*CH_3O;*OH

118

Lattice="29.606955 0.0 0.0 0.0 31.579587 0.0 0.0 0.0 31.52559" Properties=species:S:1:pos:R:3:forces:R:3

O	13.52759546	14.41838312	16.80694903	0.00527984	-0.01569775	-0.01847140

C	12.83546883	15.49471922	17.41868560	-0.01012100	0.00045196	0.00149734
Cu	15.25414325	15.02783932	15.79058839	-0.01506324	-0.01006894	0.00033650
O	15.66298109	13.02945165	15.39760704	-0.01030436	0.01440622	0.01733760
Cu	13.67197083	15.97723279	14.13073185	0.01310187	0.04655478	0.06102044
Cu	15.82952426	16.66138170	13.77875101	-0.03748665	0.02969785	0.03109526
O	17.41579632	17.60511929	12.91486854	-0.02184455	0.00103149	0.00879935
Cu	15.09040262	17.35514094	15.95107069	0.02199730	0.01127748	0.00961369
Cu	17.34295865	16.23914174	15.75264678	-0.07658074	-0.03891433	-0.00262446
O	18.99420235	17.62919465	16.16031357	-0.02419186	-0.00690200	-0.00764499
Cu	16.19363070	16.29493843	17.80201000	-0.02385213	0.00318570	-0.05918504
O	17.10580168	15.97798671	19.53109591	-0.02137798	-0.00173843	-0.02220366
O	11.97190010	14.95368807	13.80910013	0.03250068	0.01111070	0.01281006
O	13.97744905	19.09008967	16.00338032	0.00993683	-0.02565629	-0.00169597
O	18.68231586	14.84039507	15.21798595	-0.02432306	0.01950894	-0.00032147
O	18.83853081	14.17979993	19.41408994	-0.01182759	0.00139454	-0.01642769
O	18.69289731	13.11780128	16.97043690	-0.02367770	0.00383081	-0.00005966
O	16.52208869	11.94205000	17.82343745	-0.00354615	0.02419839	-0.00785502
O	18.36099798	15.58273261	11.79209206	-0.01672960	-0.00942833	0.01921711
O	9.99521542	15.58424387	15.14595412	0.01983850	0.00438919	0.01071724
O	17.62199025	12.92108593	13.77527791	-0.01233559	0.01352712	0.00920905
O	14.38477051	13.32948316	18.96706850	0.01777525	0.00823664	-0.02017123
O	12.24317929	18.44577779	18.18187267	0.01929154	-0.02065802	-0.02240397
O	16.61916475	16.19142069	9.93734868	-0.01052380	0.00686476	0.02075964
O	15.63122915	9.95120012	16.39921443	-0.00197267	0.02267179	-0.00403893
O	13.46137952	12.51550319	13.96560973	0.00592040	0.01941230	0.01730738
O	15.99895900	20.61083204	16.75593650	-0.00085066	-0.01938948	-0.00662908
O	16.00439827	19.41083669	19.02298897	-0.00575079	-0.01492488	-0.01588054
O	14.23938456	17.83096376	19.99861648	-0.00020824	-0.01439319	-0.01874318
O	12.83530243	19.20019300	13.61593573	0.01209894	-0.00774345	0.01117283
O	13.21180131	18.25570753	11.32483678	0.01495283	-0.01148099	0.01343478
O	15.78291173	18.99791490	11.11688247	-0.00192232	-0.01748221	0.02177270
O	13.11853186	10.41991376	15.77116366	0.00724375	0.02474760	0.00260374
O	14.81885659	15.21550371	20.60248324	-0.00268012	-0.00207465	-0.01750825
O	18.58711574	18.20557833	18.75694272	-0.01628144	0.00028985	-0.00515792
O	17.18944411	12.33605867	20.41215447	-0.01486206	0.01751248	-0.01518708
O	12.02185070	12.44013002	17.31508752	0.02023832	0.01549412	-0.00942603
O	9.88601637	14.05323960	17.28508787	0.02275868	0.00800454	-0.00281797
O	17.73782246	19.82814425	14.84216726	-0.01991141	-0.01429107	0.00841895
O	14.22309706	15.68345062	11.08320150	0.00442743	0.00338651	0.00794210
O	10.82568574	18.17965242	15.54641153	0.02985878	-0.00837105	-0.00353266
O	11.36153057	20.64267210	16.75018067	0.01836282	-0.01247937	-0.00795576
O	15.27535051	13.62469220	12.32334386	0.00657678	0.01312442	0.01572075
O	15.38916916	20.64181193	13.30125901	-0.00438781	-0.02639263	0.01958925
H	19.54807299	15.30470305	15.21860644	0.00086160	-0.00349850	-0.00483002
H	14.68770220	19.80024696	16.14055805	-0.00831245	-0.00122973	-0.00678211

H	13.59538198	19.16574994	15.06300046	0.00130434	-0.00808574	-0.00067960
H	11.69208895	15.03530443	12.87097312	-0.00064956	-0.00209605	-0.00413278
H	11.07768730	15.22885820	14.42178517	0.01416710	-0.00201362	0.01241235
H	11.63691943	17.83251139	17.70400657	0.01720884	-0.00639873	-0.00433893
H	12.92805188	18.59201472	17.46854881	0.02144091	-0.01873803	-0.01371142
H	16.36441943	15.63443187	20.13003919	0.00855101	-0.00729113	0.00234875
H	17.83930507	15.18096829	19.45280735	-0.00546376	-0.01069353	-0.00561286
H	15.90525921	12.65291987	16.29333627	-0.00032413	0.00162369	-0.00044621
H	16.51438043	12.93354118	14.77322956	-0.00205698	0.00938914	0.01031856
H	18.34086972	13.44317203	19.89406999	-0.00865684	0.01190997	-0.01615397
H	18.87380911	13.82327126	18.46485090	-0.02159402	0.01534303	-0.00354760
H	18.29433568	17.35794957	19.19158382	-0.00813214	0.00072437	-0.01136034
H	17.71073292	18.67776697	18.76044290	-0.01382928	-0.02327047	-0.02228625
H	14.74676161	14.84965334	21.50823533	0.00388410	-0.00046510	-0.01590075
H	14.64944765	14.39539128	19.94810320	0.00870018	0.00987022	-0.02202064
H	14.46328455	16.08875944	11.98856610	0.00290117	0.00283595	-0.01656162
H	14.44693006	14.72146320	11.32369280	0.00056459	0.01745788	0.02533071
H	15.69922726	15.98139218	10.31460999	0.00196814	0.00892608	0.02241130
H	16.59212251	17.17035964	9.85056755	-0.00589046	-0.00629799	0.01466628
H	19.41704632	12.50361094	16.72780095	-0.01477491	0.00847424	-0.00510211
H	18.70937784	13.93312176	16.16370655	-0.02564737	0.00702932	0.00387649
H	18.31946211	20.59293522	14.64344203	-0.01190727	-0.01269551	0.00305587
H	17.17665648	20.13332351	15.62692141	-0.01178617	-0.03241529	-0.00011477
H	11.50134014	20.06455087	17.54824770	0.00767808	0.00033031	-0.00690645
H	12.26488224	20.61335976	16.36441696	0.00607701	-0.00514712	-0.00275114
H	15.20043705	12.82550389	18.69196835	0.00098361	0.02467125	-0.01912741
H	14.14087780	13.81077525	18.07359791	0.02735273	0.01011300	-0.01450305
H	13.19758024	17.27576092	11.18049043	0.01321559	-0.00224583	0.02312361
H	14.19812392	18.48988746	11.17913488	0.00438492	-0.01667394	0.01979270
H	10.09348855	14.61305692	18.06253036	0.01184581	0.00169326	-0.00641444
H	10.63915919	13.36507733	17.30979693	0.00900465	0.00831793	-0.00796593
H	10.79006833	19.11308582	15.91135658	0.01114492	-0.01625011	-0.00861213
H	11.49632569	18.26526423	14.82061161	0.02045879	-0.02148183	0.01182957
H	15.85558904	21.56685441	16.91636449	-0.00194861	-0.01425371	-0.00314452
H	15.99148682	20.15244084	17.69512102	-0.00163306	-0.02693337	-0.01540918
H	18.86685780	17.81247439	17.15990277	-0.01597035	-0.00123073	-0.00297318
H	18.67268743	18.47481462	15.75327201	-0.01334529	-0.00580603	0.00792146
H	17.09106798	13.20702074	12.98988928	-0.00351309	0.02494751	0.01837061
H	18.11016599	13.75093413	14.09658406	-0.02820777	0.02509837	0.00711353
H	15.35319491	14.37370448	12.98762457	0.01402568	-0.02094478	0.01103108
H	14.56888190	13.06367903	12.79076387	0.00770201	0.02470916	0.01992312
H	16.11409847	19.99004473	19.80803567	-0.00357311	-0.01279328	-0.00838277
H	15.30226841	18.68573663	19.35072277	0.00735798	-0.02161732	-0.02957511
H	14.59396292	20.07390547	13.47435674	0.01872644	-0.02345862	0.01472383
H	16.12321490	20.18894606	13.79019873	-0.01766177	-0.03043630	0.00805757

H	13.13650909	10.99531247	14.95797393	0.00647667	0.01101776	0.00818231
H	12.67029126	11.04500629	16.41466587	0.01179391	0.01072102	-0.00609271
H	14.63870135	10.15156544	16.18403860	0.00499313	0.01664237	0.00118047
H	16.06532381	10.06744574	15.52728712	-0.00508405	0.01831294	-0.00177184
H	13.41731533	17.87869085	19.43703673	0.01728132	-0.01191192	-0.01334522
H	14.40257707	16.85364477	20.17893444	0.00576915	-0.00125156	-0.03026500
H	16.33226710	18.42045593	11.71655793	-0.00989930	-0.00804265	0.01312259
H	15.67665695	19.79527429	11.71853733	0.00044571	-0.02158340	0.00645760
H	17.71664151	15.76141151	10.99990694	-0.01434766	-0.00123644	0.02473095
H	19.25761290	15.54703342	11.40155150	-0.01391572	0.00081492	0.00924564
H	14.18736886	12.71272446	14.64006238	0.00056139	0.02347126	0.01066860
H	12.84204475	13.29171305	14.05207384	0.01719314	0.01225686	0.02312255
H	12.99200642	18.68502912	12.67534800	0.01865861	-0.02238348	0.02256559
H	12.25372845	19.95583374	13.38032797	0.00861834	-0.00989598	0.00624371
H	18.01311757	16.81544032	12.54017440	0.00132409	-0.00091365	0.01159218
H	17.89092730	18.11330622	13.61373976	-0.00010327	-0.00764288	0.00654201
H	9.96818349	15.01044488	15.99870878	0.01879926	0.00694026	-0.00616815
H	10.16050888	16.52800558	15.42622220	0.01649413	-0.00818394	0.00422236
H	17.56500557	11.55422809	20.86613350	-0.00411948	0.00608199	-0.01081096
H	16.94224215	11.99735001	19.49392075	-0.00325510	0.01285717	-0.00545980
H	17.39839825	12.30814841	17.42958367	-0.01914585	0.01899820	-0.00765298
H	16.24549270	11.08049605	17.31739341	-0.00122694	0.02150173	0.00149126
H	12.64687537	13.21722015	16.96256126	0.01837219	0.00776446	-0.02022046
H	12.41135858	12.31272863	18.21291394	0.00421240	0.00645705	-0.00821509
H	12.22097748	15.14496850	18.28352985	0.01360422	0.01208667	-0.00285013
H	13.55804914	16.24662613	17.80912986	-0.00377241	-0.00456241	-0.00374341
H	12.16484340	16.01579040	16.69849934	0.02409325	0.00645826	-0.02416598

*CH_3O

119

Lattice="30.253230000000002 0.0 0.0 0.0 31.502679999999998 0.0 0.0 0.0 30.983305" Properties=species:S:1:pos:R:3:forces:R:3

C	13.66511563	16.47815702	18.73956458	0.00955784	-0.01542070	-0.00863584
Cu	14.33499905	16.01169482	16.01800784	0.01524023	-0.01968981	-0.04842885
Cu	16.23994001	14.81104625	16.89516702	-0.07874084	-0.03253047	-0.03431650
O	17.39904708	14.98521378	18.64473947	-0.02064161	-0.00037482	-0.02478268
O	13.10076788	16.56123278	17.44748750	0.00681224	0.00052175	-0.03726581
Cu	14.93635677	13.87186888	14.91779710	0.03377151	0.00337489	-0.01230212
Cu	14.01040056	15.83536622	13.58204228	0.01176455	0.00037567	0.01094952
O	12.72954482	16.95264281	12.52327468	0.02545909	-0.00780355	0.01996446
Cu	16.29552609	15.67099989	14.61474816	-0.01871897	0.00062481	-0.00100078
O	17.45612066	17.20728402	14.05908369	-0.01294389	-0.00894373	0.00572977
O	13.72898711	12.51712462	15.86962913	0.00981898	0.01413126	-0.00299200
Cu	15.60152711	14.31436878	12.65784443	-0.02304001	0.01659562	0.06443075
O	15.73020210	15.12145193	10.75055901	0.01011207	-0.00466754	0.02646055

O	13.76467197	13.91683631	9.92284443	0.00255791	-0.00309196	0.01388910
O	15.91207912	14.69916514	20.64515243	-0.01616461	0.00834903	-0.01211118
O	11.90936286	14.38751452	17.06475946	0.01654634	-0.01123451	-0.00776585
O	11.72890606	14.93496202	11.07997120	0.01106021	0.00069946	0.01955182
O	13.33498814	18.60718647	10.71890512	0.01010392	-0.01023632	0.01068391
O	10.52636807	15.30243292	15.07422439	0.02499940	0.00580153	-0.00663354
O	10.93351707	13.62792669	13.32171353	0.02537100	0.00698003	0.00490412
O	12.54282973	11.71701790	13.68505675	0.01448389	0.01331378	0.00310127
O	18.83557333	16.96568104	11.85977793	-0.02021398	-0.01373419	0.00754797
O	11.37024566	17.84592407	14.55195253	0.01471988	-0.01088966	0.00095279
O	13.43360394	19.05860075	15.48233687	0.01893293	-0.01232165	-0.01039100
O	14.19957468	11.71608580	11.54532458	-0.00083462	0.01032099	0.01649378
O	15.70532311	11.07935607	16.93263558	-0.00494690	0.01802884	-0.00883785
O	18.10187953	10.91804348	16.27826844	-0.01011422	0.01840690	-0.00928103
O	17.90001149	10.29504163	13.74366022	-0.01211871	0.02463668	0.00114578
O	13.35076013	20.58105176	12.51645477	0.00414840	-0.02224736	0.01361871
O	14.72416525	12.93679261	19.05660750	-0.00436942	0.01371199	-0.02490851
O	15.92687709	17.77542430	11.14230139	0.00244126	-0.01309899	0.02586260
O	12.18003662	12.16105308	18.53911766	0.01398818	0.00976868	-0.01636365
O	20.32048083	15.99154473	13.88134600	-0.02786678	-0.00841598	0.01017742
O	14.04793300	16.45413943	21.84693854	0.00001137	-0.00304303	-0.01919747
O	15.50744654	19.18188554	13.51068722	-0.00909873	-0.03034607	-0.00002579
O	17.73151607	20.27644353	14.25727648	-0.00696192	-0.02152897	-0.00123888
O	18.30263737	19.38215292	16.66741184	-0.01153120	-0.02464758	-0.00782869
O	18.91223197	16.53784742	16.65222143	-0.02124054	0.00093819	-0.00717253
O	17.10170587	11.71037282	11.50663421	-0.01032920	0.01900993	0.00984635
O	18.90115920	13.53585147	15.96941479	-0.01207931	0.01356525	-0.00553716
O	18.72051235	13.79926224	13.29352402	-0.01419633	0.00049240	0.00744417
O	18.50726176	14.32878139	10.73794442	-0.00509822	0.00926672	0.01625866
O	15.77154363	18.35996699	17.09744388	0.01135940	-0.01708315	-0.01341789
O	15.15664968	10.30100493	14.26850675	-0.00561020	0.02980591	-0.00000476
H	16.56441677	14.79927937	10.32983756	-0.00009900	0.00384041	-0.00120095
H	14.80889468	14.58418784	10.27738007	-0.00668238	0.00704973	0.00237749
H	13.29709838	12.04112120	15.07855534	-0.00285205	0.00432573	0.00911680
H	14.41053412	11.90265614	16.29280167	-0.00723504	0.00688783	0.00564741
H	17.94961806	14.17915292	18.76687765	-0.00070927	-0.00470409	-0.00166793
H	16.77515370	14.99164317	19.52097264	-0.00406004	0.00407210	-0.00881484
H	12.63607571	13.91705743	16.57711083	0.02265904	0.00905249	-0.01116003
H	12.38197410	15.33698198	17.29204804	-0.00074240	-0.01703574	-0.00768615
H	12.10446159	17.41487356	13.18020902	0.00307993	0.00690655	-0.00184870
H	13.00333954	17.66334418	11.77746940	0.01194101	-0.00611694	0.01678604
H	16.73065571	17.88638319	13.95417496	-0.00559542	-0.00398632	0.00489723
H	17.84819558	17.07559834	13.11477274	-0.01031423	-0.00214602	0.00018386
H	13.31216411	19.45762306	11.26923491	0.00608717	-0.01738397	0.00676285
H	14.30918589	18.42484105	10.62438758	-0.00361471	-0.00704543	0.01617505

H	12.04996152	15.75769444	11.56174585	0.01513372	-0.00881092	0.01304724
H	11.44680448	14.36829100	11.85967129	0.02680038	0.00678593	0.01121620
H	10.66652157	18.52718609	14.52838595	0.01389840	-0.00577123	-0.00046229
H	12.19568548	18.31510097	14.99628258	0.01689624	-0.02403171	-0.00436720
H	17.95336847	13.92163470	15.92628814	-0.01246375	-0.00037017	-0.01476841
H	19.42054658	14.34158422	16.21504203	-0.02379527	-0.00192304	-0.00772142
H	18.96198765	13.37981066	14.17016408	-0.03058924	0.00497151	-0.00899718
H	17.74073193	14.03462441	13.45321064	0.00969023	-0.01040687	0.00767285
H	16.85864011	17.92556046	10.86758463	-0.00894808	-0.01224157	0.01693772
H	15.81835448	16.76240371	11.05262776	-0.00707272	-0.01482198	0.03883521
H	14.07707785	10.81849475	11.16590034	0.00319728	0.01169355	0.01388737
H	13.58137795	11.73356601	12.35221689	0.01717552	0.02857882	0.00845352
H	11.91046553	13.04344789	18.12722580	0.01365476	0.00783220	-0.00928871
H	12.34857906	11.63324568	17.72915104	0.01067596	0.00839818	-0.01089217
H	14.16983044	19.00532371	14.81185267	0.00734152	-0.03655453	-0.00124858
H	13.81958638	18.58440616	16.26057838	-0.00293266	-0.00117899	-0.01519559
H	18.53314233	11.81120318	16.34071207	-0.01735582	0.00811523	-0.00738779
H	18.09384961	10.72837306	15.25802061	-0.01038283	0.01720587	0.00193256
H	13.22475391	16.26251100	21.34570819	0.00154644	-0.00391987	-0.00104520
H	14.22862513	17.38588743	21.59591555	0.00237573	-0.00367377	-0.00730610
H	13.76548654	12.63458516	18.91061008	0.01639107	0.01912029	-0.01725361
H	15.23829333	12.43291768	18.37923280	0.00078243	0.03429698	-0.01657329
H	12.04977505	10.87161002	13.74255013	0.01026101	0.01074099	0.00237155
H	11.82221352	12.49125279	13.56314238	0.02506179	0.01727333	0.00452443
H	12.92596056	14.35950027	10.39097600	0.01866172	0.00161584	0.02298140
H	13.87661311	13.07024784	10.44598350	0.00984519	0.01865740	0.02123800
H	19.55999072	16.72050436	12.53024975	-0.02231676	-0.00637597	0.00075709
H	18.69950098	16.10738107	11.37359673	-0.02126059	-0.00066248	0.01778125
H	19.72719119	15.19478853	13.75847252	-0.02318545	0.00678213	0.00623341
H	19.96262730	16.40564693	14.70088647	-0.02262795	-0.00807783	-0.00696273
H	10.02054219	13.31513192	13.14554527	0.00979952	0.00494152	0.00487808
H	10.79216207	14.44021588	14.12035837	0.02945595	0.00989369	0.00367400
H	15.33771155	10.46578890	15.23558403	-0.00446835	0.02171534	-0.01329289
H	15.18164518	11.22322418	13.90742246	0.00567731	0.03012139	0.00359365
H	18.73174263	18.48592342	16.62838235	-0.01366124	-0.00456204	-0.01103029
H	17.37898276	19.12453179	16.95067761	-0.00169388	-0.01395136	-0.01438423
H	17.97122102	19.91669805	15.19695262	-0.00921843	-0.01450103	-0.00548288
H	18.42415906	19.87095001	13.69366214	-0.00569248	-0.01875586	0.00254204
H	11.04142185	14.94281894	15.89589055	0.02696021	0.00425819	-0.01229961
H	10.88934495	16.22470631	14.91343052	0.03125547	-0.01267811	-0.00039187
H	17.82912545	10.96414524	13.00235588	-0.00788923	0.01685681	0.01184221
H	16.93667499	10.04508783	13.83973469	0.00257684	0.01380067	-0.00088772
H	18.78630406	13.99435909	11.65166280	-0.01785764	0.00631300	0.01240389
H	19.21722317	14.03358725	10.12921118	-0.01139906	0.00263955	0.01455180
H	18.07315622	16.37830565	16.12676055	0.01353268	0.01247995	0.00482609

H	18.61588428	16.21085253	17.54224618	-0.01825080	0.00335628	-0.02065632
H	16.78003261	11.05673065	16.65996744	-0.00913423	0.02672522	-0.00951101
H	15.57714609	10.30073674	17.51653382	-0.00070547	0.01187703	-0.00689086
H	17.39676586	12.60665503	11.22082250	-0.02147071	0.00486026	0.01869622
H	16.11991976	11.81502257	11.62931526	0.00385056	0.01792807	0.01322136
H	15.28223014	15.36872542	21.03607729	0.00411210	-0.00762729	-0.01703994
H	15.33665109	14.02411927	20.15729471	-0.00246149	0.01272080	-0.01472349
H	13.58145688	21.49583850	12.25323482	0.00514135	-0.01116528	0.00415343
H	14.18754550	20.23157329	12.94956613	-0.00385959	-0.01357003	0.00223010
H	15.65874787	18.70224153	12.63989561	-0.00801823	-0.02447036	0.01928309
H	16.37752808	19.70287820	13.75017835	-0.01241673	-0.02186389	0.00262587
H	13.12213869	15.72497919	19.36236463	0.01632397	-0.01734046	-0.00840144
H	13.61845674	17.46166169	19.26881470	0.00995784	-0.00494554	-0.01004841
H	15.53982430	18.05809815	18.00402655	0.00529040	-0.02280146	-0.02390625
H	15.86235915	17.47136657	16.61875141	0.01012782	0.00712396	-0.00825679
H	14.72865917	16.13631950	18.73979495	0.00941428	-0.01371291	0.00214137

*CH_3OH;*OH

119

Lattice="29.326839 0.0 0.0 0.0 31.579587 0.0 0.0 0.0 31.525589" Properties=species:S:1:pos:R:3:forces:R:3

C	12.65323001	15.43873666	17.53419172	-0.00782593	0.00405232	0.00376558
O	13.05559260	14.39770217	16.61458961	0.01655808	-0.01400132	-0.00838993
Cu	15.05832014	15.16201688	15.46246269	-0.02269680	-0.00727847	0.03960179
Cu	15.84564168	16.35339673	17.56764843	-0.01786100	0.00600284	-0.04765810
O	16.64350632	15.90595339	19.34168987	-0.02759886	-0.00320941	-0.02244023
O	15.44011768	13.11819930	15.36005457	-0.00951755	0.01185885	0.02213559
Cu	13.49348780	16.37362887	13.98976224	0.01618218	0.01486311	0.04605466
O	10.93703851	14.37869726	15.13416854	0.01234871	0.00481402	0.01476410
Cu	15.83018459	16.57417956	13.54105622	-0.04458819	-0.00032443	0.00697352
Cu	17.21358456	16.26969592	15.59910361	-0.08582539	-0.03603086	-0.00824754
O	18.60599840	14.76854852	15.15992809	-0.02547828	0.01679465	0.00027622
Cu	15.02449571	17.50011298	15.63560645	0.03019885	-0.02461755	0.01507631
O	14.54409417	19.37597225	15.11982161	0.01061165	-0.01825977	0.00381812
O	17.40624421	17.78656717	12.73480318	-0.01867633	0.00346979	0.01327493
O	18.80151985	17.64038290	16.13208848	-0.02388045	-0.00861287	-0.00919586
O	18.41028766	14.16734234	19.39940104	-0.01338052	0.00015932	-0.01383271
O	18.45357722	13.12151996	17.00551777	-0.02023372	-0.00524662	0.01074645
O	16.45483719	11.80216572	17.76391598	-0.00696288	0.01737042	-0.00698565
O	18.20997788	15.59754946	11.72077689	-0.01117101	-0.01037207	0.01523272
O	9.54187814	14.95520876	17.34664875	0.02852913	0.00347898	0.00508529
O	17.24463262	12.97510579	13.57938582	-0.01123364	0.01516084	0.00991653
O	14.50111667	13.22347531	18.62407856	0.00815280	0.01674884	-0.01600040
O	12.95042775	18.59094954	17.60025189	0.01373170	-0.01482090	-0.01179990
O	16.43617948	16.39457671	9.93742843	-0.01374124	0.00601193	0.02184952
O	14.28638257	14.98996526	20.56959924	-0.00511883	-0.00303391	-0.02651377

O	15.67212240	9.91713473	16.16860544	-0.00327934	0.02372539	-0.00176014
O	13.24265005	12.48004500	13.97293211	0.00804623	0.01848430	0.02091707
O	15.86640337	20.70600150	16.95637405	0.00080414	-0.02283741	-0.00139565
O	15.83049125	19.40337904	19.24189042	-0.00779822	-0.01682057	-0.01577023
O	13.87686198	17.82721233	20.00186654	-0.00357190	-0.01661879	-0.01999882
O	12.71070708	19.66814298	13.33902231	0.01079327	-0.01022568	0.00539454
O	13.10545268	18.21420705	11.30863810	0.01942957	-0.00816955	0.00352745
O	15.65177926	19.01478406	11.03973709	-0.00324626	-0.01219385	0.02038683
O	13.10225122	10.28481969	15.76232926	0.00660897	0.02624220	0.00302214
O	18.24749231	18.10590693	18.70253889	-0.01533362	0.00023419	-0.00490459
O	16.86016627	12.15144199	20.40856905	-0.01414863	0.01798357	-0.01659887
O	12.27914745	11.95316949	17.82325315	0.01419983	0.02441686	-0.01568134
O	10.82990037	13.54624977	19.55949256	0.01622881	0.00436361	-0.01029399
O	17.54035357	19.84760002	14.86927368	-0.02137982	-0.01283536	0.00777256
O	13.88187225	15.58719036	10.51751491	0.00855718	0.00601116	0.01693998
O	10.30421816	17.72332226	17.47648689	0.01625898	-0.01313398	-0.00139175
O	11.07225046	19.59257750	19.26736278	0.01111603	-0.00721551	-0.00329819
O	14.87142367	13.85957975	12.22501184	0.00588738	0.00361172	0.01020939
O	15.23329443	21.00079022	12.78303117	-0.00261697	-0.02434067	0.01901117
H	19.47072674	15.22782587	15.23426754	-0.00091876	-0.00505143	-0.00256700
H	14.64842317	20.04287739	15.85023077	-0.00627045	-0.00131477	-0.00934015
H	13.67037454	19.49224212	14.61680619	0.00787924	-0.01439908	0.00169303
H	11.09578733	15.24230067	14.66865546	0.01346302	0.00060500	0.01306709
H	10.34010278	14.60794078	15.94447579	0.01888833	0.00104021	-0.00093446
H	12.15206615	18.12331334	17.22818930	0.03142272	-0.01272514	-0.01692460
H	13.72841371	18.18566170	17.08300798	0.00471164	-0.01568503	0.00199938
H	15.91496284	15.59757912	19.95126969	0.00810722	-0.00532980	0.00016150
H	17.40838881	15.10040684	19.33927070	-0.01157303	-0.01022148	-0.01171345
H	15.76748722	12.71103604	16.20893145	-0.00283257	0.01271617	-0.00648367
H	16.23433437	13.01429143	14.66074291	0.00351057	0.00348831	0.01134972
H	17.93573391	13.41377968	19.87145722	-0.00920425	0.01198501	-0.01666361
H	18.50345093	13.79562122	18.43860814	-0.02162967	0.01708281	-0.00178698
H	17.90675889	17.25813448	19.09485328	-0.00604498	0.00169049	-0.01081397
H	17.41805421	18.65133829	18.78996630	-0.01292863	-0.02198311	-0.02429597
H	14.52231513	14.54327889	21.40985271	0.00215283	-0.00071161	-0.01825948
H	14.40182700	14.25956313	19.86106279	0.00311861	0.00720993	-0.01909732
H	14.14796777	14.81188044	11.12674965	0.00679366	0.01774651	0.02159828
H	15.50495069	16.04948136	10.11365435	0.00478292	0.01051216	0.02543493
H	16.30878807	17.37021255	10.03230737	-0.00728272	-0.01040292	0.01570751
H	19.22426581	12.52129069	16.91871891	-0.01168735	0.00431929	-0.00310754
H	18.56570441	14.03600888	15.99890676	-0.01921894	0.02218934	-0.01802196
H	18.14947788	20.60402265	14.73587008	-0.01585625	-0.01344809	0.00149539
H	16.96607115	20.12755592	15.64410639	-0.01095079	-0.03194673	-0.00431447
H	11.44052608	19.12130360	20.04849293	0.01047677	-0.00799144	-0.00695187
H	11.87886196	19.66084357	18.69232830	0.00755146	-0.00244425	-0.00065995

H	15.32621541	12.67108990	18.32903207	-0.00252926	0.02934754	-0.00969360
H	14.30559353	13.75693320	17.80870036	0.02256972	0.01282657	-0.01282198
H	13.16514254	17.22263543	11.33041984	0.01997523	-0.00000286	0.01849969
H	14.08295895	18.47424136	11.16665015	0.00531875	-0.01811152	0.02146714
H	11.54149136	13.94698244	20.10505751	0.01359855	0.00308608	-0.01379018
H	11.31584971	12.84369235	19.03172136	0.00730781	0.00937200	-0.00510712
H	10.40416893	18.38104893	18.24486845	0.00532469	-0.01460289	-0.00619080
H	9.77536114	18.21424683	16.81226224	0.01267171	-0.00561009	0.00243464
H	15.84511961	21.65375244	17.20394252	-0.00012704	-0.01550979	-0.00292088
H	15.79715373	20.20097964	17.84109878	-0.00326356	-0.02701379	-0.01755060
H	18.60671509	17.79752470	17.12521574	-0.01539907	0.00129376	-0.00235167
H	18.44847655	18.47682721	15.72109150	-0.01276019	-0.00278061	0.00772381
H	16.67776536	13.37251853	12.86635105	-0.00133017	0.02627625	0.02012659
H	17.83515033	13.72551050	13.88454748	-0.02607328	0.02686566	0.00816712
H	14.90108530	14.64998432	12.86874855	0.01156899	-0.02195408	-0.01498924
H	14.20622644	13.26936019	12.70054923	0.01225573	0.02457525	0.01680163
H	16.00162552	19.95540239	20.03425051	-0.00456950	-0.01080077	-0.00775977
H	15.11578007	18.71866021	19.55626599	0.00427719	-0.01685825	-0.02424166
H	14.29445274	20.70745638	12.94525495	0.01085578	-0.01159657	0.00394179
H	15.70170816	20.51843663	13.50785550	-0.00292429	-0.02292056	0.00269888
H	13.01878412	10.94439168	15.02122226	0.00703235	0.00939167	0.01099564
H	12.75292919	10.79030147	16.55229151	0.01107179	0.01284293	-0.00643484
H	14.66156398	10.06005989	16.00533994	0.00401598	0.01738734	0.00227219
H	16.07449949	10.12922160	15.29951320	-0.00608088	0.01894098	-0.00014172
H	13.43270420	17.88327957	19.09961445	0.01490954	-0.01503269	-0.01122022
H	14.02926988	16.85928470	20.17265801	0.00197663	0.00158892	-0.02678729
H	16.25538750	18.54918089	11.68242129	-0.01127718	-0.00522318	0.01420736
H	15.53275741	19.89603164	11.52718007	0.00029401	-0.02089006	0.00996769
H	17.53242287	15.83506791	10.98051780	-0.01433506	-0.00120501	0.02594467
H	19.05508070	15.45268742	11.24676971	-0.01460089	0.00140283	0.00958738
H	13.97871099	12.73730301	14.61636034	0.00129179	0.01935216	0.01382673
H	12.49662610	13.09003275	14.19207290	0.02419711	0.01427405	0.01034147
H	12.81132947	18.96839548	12.55071150	0.01975214	-0.01674604	0.01595801
H	11.82721155	20.07404142	13.23447781	0.01359599	-0.01032619	0.00132704
H	17.99831630	16.98800503	12.45080899	0.00445332	0.00349515	0.01229712
H	17.75448034	18.23921217	13.54011585	-0.00390047	-0.00619711	0.00860189
H	9.94773699	14.48558635	18.13067582	0.00867000	0.00654417	-0.01039661
H	9.74424755	15.91167067	17.52650673	0.01328769	-0.00789338	0.00226002
H	17.37789228	11.45675480	20.86581781	-0.00372251	0.00580747	-0.01090075
H	16.73470458	11.79036322	19.47557651	-0.00505980	0.01097828	-0.00565432
H	17.32861739	12.29214890	17.37879051	-0.02645011	0.00571982	-0.00543276
H	16.19573028	10.99179828	17.16933351	-0.00258771	0.01758601	0.00290435
H	12.23728678	12.72379067	17.19690159	0.00886953	0.00958670	-0.00778348
H	13.15638294	12.20088655	18.25934737	0.00806862	0.01066498	-0.01220939
H	12.29338126	14.37375178	15.91385708	0.03377489	0.00847000	-0.00101623

H	12.08071284	15.01486321	18.37747456	0.02089212	0.01865674	-0.01023777
H	13.57823173	15.93531016	17.89109217	-0.00322557	0.00179992	-0.01416355
H	12.04289467	16.18605864	17.00414537	0.02907545	0.00524601	-0.01078051
H	13.41979456	15.17479488	9.75753892	0.00572474	0.00530655	0.01405887

CH_3OH;*OH

119

Lattice="31.857210000000002 0.0 0.0 0.0 31.26031 0.0 0.0 0.0 30.666656" Properties=species:S:1:pos:R:3:forces:R:3

C	16.32332495	14.66206744	12.09409066	0.00508932	0.00584417	0.02213690
Cu	16.35265598	14.66803593	15.70488114	0.02290222	0.00654366	0.00759505
Cu	18.50323476	14.91019019	14.47013250	-0.05698331	0.06187284	-0.02272117
O	19.10250335	13.62390633	12.92259910	-0.01664400	0.01754879	0.02534663
O	15.29813252	14.86302317	11.09150974	-0.00771028	0.00395413	0.01512716
Cu	16.91787859	16.78713119	15.03759435	-0.02906493	-0.01654255	-0.03527090
Cu	14.61580465	16.37570204	15.56049025	0.06753069	-0.00240080	0.00146792
O	12.70493461	16.80895391	16.01222943	0.02645337	-0.01299563	-0.01171019
O	17.70028334	18.46787953	14.12908045	-0.01027866	-0.02288930	0.00565247
Cu	16.08655258	16.31692895	17.47454735	0.01291337	-0.04604998	-0.07577243
O	15.54169843	14.82149544	19.12955566	0.00781764	0.00860520	-0.02449349
Cu	18.37621007	15.71875148	16.69081106	0.00144646	0.02953951	-0.04303563
O	19.09165189	16.46552291	18.55775247	-0.00129063	0.00047720	-0.02283416
O	15.50726410	17.76657545	18.92464473	0.01266807	-0.00901132	-0.02209436
O	15.75492414	12.78074215	15.97287353	-0.00034036	0.01772667	-0.01557911
O	11.51419027	15.24780845	17.56155252	0.01885403	-0.00530957	-0.00621042
O	13.36699406	13.45805559	18.33844132	0.01523878	0.00487481	-0.02006165
O	13.17427182	12.60548003	15.97910722	0.00675164	0.00746558	-0.00721863
O	18.90522261	14.22845663	19.73727129	-0.01500313	-0.00442681	-0.02352011
O	19.17461953	14.60575435	10.61474692	-0.01756045	0.00620824	0.01005851
O	16.70803423	12.23733174	18.32525302	-0.00438113	0.01988194	-0.01350232
O	13.52377894	14.02818705	13.91243219	0.01413421	0.00142856	0.00638212
O	15.72395560	17.61369620	11.41914987	0.01308635	-0.02559064	0.02096481
O	21.33862975	14.20210995	18.78755366	-0.01243645	0.01156523	-0.01657837
O	13.90123426	10.02051313	15.75187143	0.00982145	0.02121692	-0.00089505
O	17.47017606	11.60644766	14.26334469	-0.01311434	0.02702786	-0.00210930
O	16.93399010	20.23325909	15.92877943	-0.00046356	-0.02196820	0.00245349
O	14.53724399	19.85362216	15.14062024	0.00950700	-0.02104566	0.00251152
O	13.89140303	18.41671103	13.17554298	0.01040678	-0.01566175	0.00534326
O	20.28904481	17.80028027	13.91284032	-0.01739861	-0.00622405	0.00785681
O	21.88241777	16.26965961	15.10272739	-0.02152769	-0.00690774	0.00879052
O	21.52041751	17.21664112	17.54648361	-0.02184683	-0.01325349	-0.00674375
O	15.30468508	9.99283502	13.48279148	0.00298788	0.02613160	0.00503669
O	12.14553250	16.23110518	13.49559134	0.01062824	-0.00966143	0.00143829
O	13.54621891	18.87156815	17.57364159	0.01094638	-0.00557062	-0.01555083
O	10.75176824	13.61152296	15.59817298	0.02434370	0.01191610	-0.00728062
O	14.50100533	12.40459500	12.07879191	0.01564628	0.00831805	0.02069315

O	16.95829854	14.22077828	9.07566785	-0.00258900	0.00556572	0.02081107
O	17.80274148	19.20180319	18.22427844	-0.00324168	-0.00941940	-0.01826523
O	20.94386339	13.84215714	16.13599538	-0.00705765	0.00540108	0.00189328
O	18.71406095	17.06118593	11.57872471	-0.00395574	-0.00580770	0.02288710
O	17.74797429	19.61469562	11.50062922	-0.00500195	-0.01296244	0.01927403
O	18.87487200	12.22597879	16.55463803	-0.01182382	0.02000366	-0.00093338
O	20.11709807	19.25349676	16.37703107	-0.02491224	-0.01888831	-0.00801108
H	15.22311263	15.51810326	19.74602235	-0.00238067	-0.01183387	-0.00068051
H	17.56420101	19.18559845	14.83291112	0.00646226	-0.00604741	0.00137514
H	18.68695982	18.25668535	14.11300411	0.00083893	-0.00559640	0.00588227
H	20.03164207	13.34777470	13.09171742	-0.00051678	-0.00116423	0.00226675
H	19.10963534	14.02961574	11.90442620	-0.00910565	0.00578509	0.01286614
H	16.56378027	17.30982339	11.84750584	-0.00551037	-0.01547191	0.02921117
H	15.32952206	16.75765037	11.09948129	0.00705464	0.00315094	0.01174496
H	12.22656320	16.68318400	15.13590249	-0.00873200	-0.00208280	0.00953377
H	12.22651888	16.11742894	16.69663452	0.01075975	-0.01188212	-0.00603388
H	14.70811135	12.73317181	15.97715709	0.00485686	0.00141464	-0.00092287
H	16.05880818	12.54595714	16.94075252	-0.00974350	0.00170434	-0.00045917
H	11.09235476	14.63823164	16.86576165	0.02147357	0.00535501	-0.00374911
H	12.19105209	14.63359569	17.98493650	0.01550212	0.00892876	-0.01780272
H	13.07521572	18.12280744	17.10618302	0.01816360	-0.00846840	-0.00810964
H	13.96017593	19.30600153	16.77916344	0.01603301	-0.02842093	-0.00378360
H	11.26050105	16.06583072	13.10964837	0.01492801	-0.00478452	0.01070659
H	12.57696067	15.30603887	13.61064795	0.02106635	0.00101807	0.01398243
H	20.14930649	14.48148459	16.05276351	0.01095002	0.00019627	0.01108278
H	20.42303866	12.98218216	16.19324573	-0.01702792	0.02354673	-0.00715830
H	21.25306786	14.03383499	17.79194639	-0.01760636	0.01583645	-0.00534104
H	21.72215296	15.10618280	18.82128020	-0.01300622	0.00121621	-0.01271013
H	13.09740846	12.76230467	18.97302305	0.00807197	0.00306034	-0.01548040
H	14.70494881	14.33371394	18.82365324	0.00996472	0.01080208	-0.02993804
H	18.12280371	19.94986370	18.77329394	-0.00281230	-0.01284516	-0.01243214
H	17.38826451	19.63766726	17.40789914	-0.00615195	-0.03257112	-0.01068458
H	16.89891436	19.32864183	11.08782161	0.00268826	0.00065921	0.00995588
H	17.59477835	19.38278292	12.45584000	-0.00725079	-0.01125391	0.00956080
H	13.30272147	13.40408670	14.74730528	0.01973474	0.02556599	0.00165025
H	14.28568940	14.57980779	14.30274388	-0.00716810	-0.00645392	-0.01084161
H	21.66096802	15.30580465	15.13595337	-0.02635344	0.00652708	0.00382356
H	21.76560392	16.56806648	16.08218257	-0.02483645	-0.00972620	-0.00179310
H	16.23233150	14.52553220	9.70160136	0.00392275	0.00537717	0.00545812
H	16.74989592	13.27015462	8.95698564	-0.00260805	0.00369973	0.01355743
H	18.52380929	18.02395899	11.34337723	0.00174440	-0.01790023	0.01465560
H	19.28196829	17.13094189	12.39244298	-0.02459069	-0.01901911	0.01711991
H	17.21783577	21.14538775	15.70968880	-0.00045972	-0.01472619	-0.00086527
H	15.94702879	20.13401513	15.59058827	0.00682615	-0.02972469	0.00488099
H	14.72568106	18.22231039	18.41985041	0.00218199	0.00223447	-0.00839179

H	16.26127298	18.40703033	18.81922909	-0.00252200	0.00225693	-0.00592518
H	17.62926057	12.12956062	17.95921794	-0.00623123	0.02665627	-0.00787168
H	16.73659626	13.12147095	18.77236199	0.01070485	0.01942432	-0.02634324
H	18.50808781	13.15334815	16.40859957	-0.00920984	-0.01697177	0.01081307
H	18.47995023	11.76292690	15.75213502	-0.00850250	0.02886466	0.00109044
H	14.03115278	20.65420124	14.88507312	0.00466678	-0.01424708	-0.00045032
H	14.31540754	19.16036289	14.35612421	0.01679261	-0.02818318	0.01660942
H	20.06719946	18.69763863	15.55390493	-0.02711297	-0.01737827	0.01272047
H	19.31736830	18.99341860	16.90017395	-0.01131379	-0.03044405	-0.01996616
H	16.17046647	10.45041750	13.66973600	-0.00501803	0.01325179	0.00463385
H	14.81951820	10.66264998	12.93457011	0.00424774	0.00742514	0.01356125
H	14.44248706	9.97683230	14.88950827	0.00289964	0.01604363	0.00513918
H	14.59418720	9.98058596	16.44420756	0.00884585	0.01705236	-0.00465404
H	14.63724423	18.09014649	12.57331119	0.01090214	-0.01458489	0.02648730
H	13.28819307	17.62882323	13.28316730	0.02330721	-0.01085047	0.01366103
H	20.66496941	16.87376367	17.93334690	-0.01674155	-0.00782515	-0.01666257
H	21.20104111	18.10701930	17.20442371	-0.01281962	-0.01786740	-0.00186516
H	19.88529783	14.12428518	19.42843306	-0.01798473	0.00442132	-0.02167368
H	18.93637829	14.17524607	20.71421000	-0.00310876	0.00248614	-0.01568451
H	16.78105571	12.11818463	14.79944236	-0.00391044	0.02190535	-0.00549770
H	17.93890507	12.29636813	13.71787643	-0.02001530	0.01582405	0.01062047
H	20.92768100	17.06412817	14.39506920	-0.03217108	-0.01277454	0.00905135
H	20.91144126	18.41122098	13.46119597	-0.01073691	-0.00795516	0.00588552
H	18.87358598	15.65370042	19.17836471	-0.00965903	0.00319941	-0.00332997
H	18.42776268	17.18069109	18.71917580	-0.00329495	-0.00099704	0.00401580
H	18.37597560	14.39437665	10.02719837	0.00003307	0.00807439	0.01752890
H	19.06391138	15.58643189	10.80918466	-0.01205602	-0.00523548	0.01123729
H	10.05314325	12.98372560	15.87674237	0.01414854	0.00238186	0.00075336
H	11.61993710	13.08897876	15.73116762	0.00956430	0.01388641	-0.00243608
H	13.29020508	13.01443120	17.35646902	0.01926679	0.01952964	0.00050765
H	13.28671878	11.60250633	15.86101233	0.00816032	0.01287597	-0.00479872
H	17.07201270	15.46413418	11.99728176	0.00612447	-0.00256942	0.03686240
H	15.45109655	12.42555290	12.32884363	0.00657800	0.00656652	0.01921343
H	14.08586703	12.96973984	12.82669397	0.01325746	0.00198572	0.01739951
H	15.92188937	14.71774632	13.12598845	0.01353981	0.01455760	0.02272053
H	14.68228918	14.08209441	11.22581125	-0.00242245	0.01387482	0.00459374
H	16.85010860	13.70047792	11.93378904	0.00988107	0.00270399	0.02658781

CH_3OH

120

Lattice="30.299939000000002 0.0 0.0 0.0 31.45075 0.0 0.0 0.0 31.924093999999997"
Properties=species:S:1:pos:R:3:forces:R:3

C	13.47002670	16.53540714	19.07768018	0.01751950	-0.01295881	-0.00297752
O	12.92652800	16.70731946	17.74960099	0.00685706	-0.00108920	-0.04058655
Cu	14.31096153	15.99744444	15.97834591	-0.00373880	-0.00375660	-0.03209496

Cu	16.32808370	15.57913515	14.68429639	-0.01639329	-0.00031395	-0.00240501
Cu	15.60923626	14.28330498	12.76347436	-0.02185863	0.01871695	0.06165664
O	15.75036559	15.07088980	10.83346006	0.00988766	-0.00413760	0.02761888
Cu	16.20335303	14.77865793	16.96895078	-0.07899891	-0.02656090	-0.01684952
Cu	14.95207308	13.82780866	15.01442365	0.04425673	0.00697284	-0.01299632
O	13.71881182	12.45881276	15.94883882	0.01001452	0.01105494	-0.00099416
Cu	13.90102300	15.75739404	13.65546510	0.00408868	0.00038693	0.00521108
O	12.58271135	16.92353231	12.54586908	0.02337666	-0.00654053	0.02073640
O	17.53333219	14.99117025	18.72623422	-0.01992977	-0.00010526	-0.02328864
O	17.37845614	17.20117025	14.18358594	-0.01166120	-0.00915383	0.00513604
O	13.75536408	13.90029873	9.96504231	0.00182050	-0.00294229	0.01522230
O	11.93012461	14.18635217	17.06308431	0.01723527	0.00121316	-0.00645054
O	15.97343859	14.64949809	20.72343231	-0.01609361	0.00813986	-0.01089741
O	13.34100069	18.58448021	10.76046396	0.01064188	-0.01008448	0.01132185
O	11.62129186	14.88838108	11.09493231	0.01097864	-0.00098113	0.02029413
O	10.38648025	15.28710489	15.13520252	0.02616611	0.00824345	-0.00280218
O	10.77252601	13.51973081	13.36524394	0.02398494	0.00696476	0.00552072
O	12.54040668	11.69160600	13.73979790	0.01399622	0.01494484	0.00324567
O	11.39507819	17.88123759	14.77945889	0.01627214	-0.01138853	0.00407447
O	13.55568050	19.11166011	15.57117376	0.01529695	-0.01287493	-0.00955743
O	18.74534031	16.94748186	11.96518890	-0.01608198	-0.01545107	0.01076011
O	14.75522197	12.90755934	19.14066910	-0.00540584	0.01126790	-0.02433988
O	15.71243812	11.03996561	17.01555871	-0.00530263	0.01758955	-0.00830708
O	18.10478673	10.90445766	16.35490073	-0.01060197	0.01773263	-0.00977831
O	17.90519353	10.26765359	13.80740010	-0.01213270	0.02518847	0.00110204
O	13.36608574	20.55366929	12.57999066	0.00368068	-0.02215931	0.01380546
O	12.18568024	12.09921933	18.78550967	0.01249460	0.00958632	-0.01529135
O	15.94562092	17.75215299	11.21574736	0.00149711	-0.01376932	0.02583549
O	20.31210686	15.99148730	13.93808326	-0.02771155	-0.00882236	0.01044825
O	14.18546112	11.70563290	11.58657216	-0.00085563	0.01014975	0.01685605
O	18.90103093	13.49437428	16.09633079	-0.01120847	0.01412200	-0.00521209
O	18.75247269	13.76058693	13.36246478	-0.01385476	-0.00037348	0.00869983
O	18.51807820	14.31844839	10.80841602	-0.00494546	0.00988635	0.01569489
O	15.51874911	19.16951288	13.56133019	-0.00858476	-0.02900115	-0.00021023
O	17.75768752	20.24549424	14.30142205	-0.00757118	-0.02211655	-0.00134009
O	18.31205119	19.32699904	16.71524005	-0.01280155	-0.02458399	-0.00781751
O	15.76989797	18.28775413	17.18610437	0.00786006	-0.01645927	-0.01213861
O	17.02006327	11.66463990	11.56471940	-0.00889564	0.01875375	0.00985035
O	15.16784477	10.30853730	14.33378847	-0.00591797	0.02915867	0.00009037
O	14.18567299	16.32038613	22.14200150	-0.00179399	-0.00173858	-0.01785259
O	18.93494681	16.51412568	16.72210819	-0.02228832	0.00127079	-0.00738914
H	16.58434218	14.72865712	10.42632466	-0.00022466	0.00380637	-0.00099865
H	14.86365879	14.58028342	10.36100845	-0.00483329	0.00716542	0.00255464
H	13.31195689	11.99081927	15.14554472	-0.00176150	0.00240301	0.00728103
H	14.40930953	11.85372182	16.37464869	-0.00798740	0.00602506	0.00630481

H	18.05200591	14.15651368	18.76570419	-0.00248518	-0.00512356	-0.00436795
H	16.93753964	14.96672467	19.58483834	-0.00405864	0.00408158	-0.01272760
H	12.66693294	13.70213305	16.57713359	0.02364705	0.01973606	-0.00921318
H	12.36375277	15.05030463	17.31314034	0.00642471	-0.02173285	-0.00763092
H	12.05363904	17.40655655	13.25171726	0.00323323	0.00706371	-0.00333355
H	12.90472313	17.62609260	11.82989581	0.01331889	-0.00770675	0.01884699
H	16.63110202	17.85281327	14.06811264	-0.00785196	-0.00242336	0.00567358
H	17.77033828	17.06376269	13.24579563	-0.00807197	0.00064798	-0.00012648
H	13.32338037	19.42747174	11.31758160	0.00607475	-0.01677920	0.00646745
H	14.31224764	18.38803641	10.68437506	-0.00325685	-0.00617863	0.01537980
H	11.91473110	15.71112548	11.59413203	0.01557003	-0.00820909	0.01528049
H	11.34949508	14.30192861	11.85336630	0.02553402	0.00655237	0.01020124
H	10.77138115	18.62947673	14.66434114	0.01463295	-0.00795571	0.00020309
H	12.27042756	18.31582783	15.12880967	0.01585822	-0.02587034	-0.00360703
H	17.94830837	13.86249510	15.96330130	-0.00791267	-0.00514840	-0.01146519
H	19.39346728	14.32151362	16.32320332	-0.02334289	-0.00443087	-0.00994186
H	19.01596305	13.28937207	14.20106927	-0.02912054	0.00438370	-0.00950936
H	17.79098120	14.03945221	13.59625906	0.01051201	-0.01025585	0.00772432
H	16.90914114	17.85401597	11.04053697	-0.01383441	-0.00969590	0.01368762
H	15.81444057	16.74498999	11.14870080	-0.00675058	-0.01481782	0.03809656
H	14.05580953	10.80479012	11.21960619	0.00283411	0.01132004	0.01383317
H	13.58930315	11.73627441	12.40380477	0.01805646	0.02861555	0.00938745
H	11.94542985	12.91855318	18.25622586	0.01202193	0.00569314	-0.00721918
H	12.30261673	11.43757661	18.07122398	0.01186170	0.00890287	-0.01325395
H	14.20869417	19.04256216	14.81729038	0.00556807	-0.03495794	-0.00161711
H	14.08895880	18.71103377	16.30796414	0.00118262	-0.01343169	-0.01196269
H	18.51926241	11.80890556	16.41917386	-0.01794779	0.00895292	-0.00771383
H	18.09331792	10.71907147	15.33689197	-0.01056008	0.01748999	0.00202392
H	13.25981064	16.11556033	21.89207221	0.00325981	-0.00232930	-0.00502712
H	14.27889314	17.26842107	21.91072325	0.00161197	-0.00365084	-0.00755622
H	13.80716523	12.56823853	19.05415565	0.01566897	0.01817808	-0.01741373
H	15.25813527	12.41235152	18.44586903	0.00037799	0.03195025	-0.01696010
H	12.09191408	10.82066334	13.77533313	0.00982676	0.01095906	0.00235869
H	11.79171388	12.40599806	13.62413736	0.02485956	0.01758336	0.00461453
H	12.91591492	14.32766922	10.40420702	0.01826349	0.00131919	0.02209985
H	13.85835133	13.05601416	10.49108671	0.00924889	0.01781023	0.02074977
H	19.48140688	16.72245377	12.63206804	-0.02348144	-0.00640849	0.00158521
H	18.63591753	16.08452955	11.47904525	-0.02079381	-0.00209129	0.01854828
H	19.75520416	15.16563289	13.82315149	-0.02273072	0.00705570	0.00651163
H	19.97939375	16.35906961	14.79078049	-0.02184846	-0.00848605	-0.00741014
H	9.86207293	13.20644731	13.17924911	0.00877970	0.00507558	0.00428583
H	10.64169295	14.30749392	14.08141593	0.02689614	0.00614023	0.00127884
H	15.35395113	10.43957896	15.30544958	-0.00421150	0.02189557	-0.01351540
H	15.25703065	11.24178198	14.00586382	0.00507340	0.02716640	0.00136190
H	18.74265674	18.42944845	16.67019244	-0.01422751	-0.00511513	-0.01091965

H	17.38196544	19.05482597	16.95702529	-0.00202181	-0.01391968	-0.01644973
H	17.99511332	19.87451761	15.23713839	-0.00938767	-0.01431590	-0.00541714
H	18.43340515	19.81898441	13.73198811	-0.00616185	-0.01927528	0.00245121
H	10.94594992	14.87866090	15.86857455	0.02559918	0.00737503	-0.01150030
H	10.81701128	16.17428887	14.97087708	0.03098394	-0.00929539	0.00008683
H	17.80168122	10.93350146	13.06601014	-0.00779373	0.01661870	0.01277549
H	16.94698071	10.00512147	13.91516231	0.00245597	0.01356538	-0.00088402
H	18.78542585	13.97399072	11.72317353	-0.01791090	0.00647581	0.01214016
H	19.23529310	14.02982527	10.20591157	-0.01167576	0.00262299	0.01468242
H	18.10501638	16.32075923	16.19066650	0.01142030	0.01196141	0.00551945
H	18.64414248	16.17277458	17.61027723	-0.02174222	0.00262500	-0.02073508
H	16.79402855	11.03588895	16.73277577	-0.00842139	0.02698268	-0.00984456
H	15.60094762	10.23893036	17.57252521	-0.00066607	0.01239406	-0.00650134
H	17.35260008	12.57214954	11.37476598	-0.01915175	0.00274557	0.01721649
H	16.04551666	11.80022894	11.71343141	0.00458940	0.01597585	0.01246874
H	15.35108531	15.26339851	21.19444893	0.00188263	-0.00583505	-0.01753070
H	15.40352889	13.97893411	20.22671515	-0.00354498	0.01254595	-0.01343154
H	13.59804997	21.46492516	12.30556094	0.00516859	-0.01119433	0.00436261
H	14.21142002	20.20335294	12.99641465	-0.00355126	-0.01327434	0.00254881
H	15.66237368	18.66616349	12.69934400	-0.00777662	-0.02365077	0.01957749
H	16.39944279	19.67732696	13.79758657	-0.01169839	-0.02080639	0.00260679
H	12.81951518	15.87853526	19.68981472	0.01073196	-0.01748989	-0.00689223
H	13.61767285	17.51115457	19.58009753	0.01428506	-0.00338615	-0.00385197
H	15.76268729	17.98971965	18.12297435	-0.00392208	-0.02890702	-0.01867804
H	15.76650184	17.37922420	16.70426074	0.00940002	0.00723131	-0.01026824
H	14.44399395	16.02712851	18.93143173	0.02023710	-0.01945795	-0.02160766
H	12.14168313	17.30674465	17.77062336	0.01247144	-0.00746675	-0.02729758

Gr-$Cu_6[H_2O]_{25}$

*

112

Lattice="9.84 0.0 0.0 0.0 8.52169 0.0 0.0 0.0 11.75" Properties=species:S:1:pos:R:3:forces:R:3

C	1.07621597	0.82912545	0.78899691	-0.00114170	-0.00312456	-0.00473542
C	1.07772837	2.25624335	0.73561859	0.00154629	-0.00597292	-0.00034965
C	2.30592527	2.96708714	0.89016600	0.00412510	0.00086697	0.00666334
C	2.31015520	4.38945427	0.96527878	0.01259963	0.00588594	0.01295958
C	1.08541050	5.10089326	0.81087620	-0.00569325	0.00653338	0.00891805
C	1.08133815	6.51752056	0.90167281	0.00364407	0.00115505	0.00773173
C	2.31945454	7.21688003	1.03810130	0.00458487	-0.00398631	0.00234713
C	2.30605411	8.64315360	0.94095472	0.00184933	-0.00352748	0.00156016
C	3.53452679	0.83602741	1.03839072	0.00028348	-0.00549297	0.01001520
C	3.53430109	2.25971544	1.04348790	0.00559914	0.00249990	-0.00685942
C	4.72549513	2.95807201	1.41558534	0.01510175	-0.00509482	-0.04368358
C	4.57717963	4.29068976	1.91703537	0.00993025	0.00081118	0.00893960

C	3.48384335	5.07284273	1.42181888	0.00112343	0.02024820	-0.00399099
C	3.52262566	6.51257037	1.35938354	0.00561556	-0.00438229	0.00679697
C	4.76558977	7.24483885	1.45909080	-0.00807770	-0.00845617	-0.00017199
C	4.76068251	8.65158278	1.21183229	0.00193296	0.00014431	0.00415330
C	5.99204347	0.85422981	1.17913596	0.00189704	-0.00305836	0.00529560
C	5.99412089	2.26934459	1.30749772	0.00902103	0.00607417	-0.00044202
C	7.25227313	2.96039754	1.23929393	-0.01382953	0.00359947	-0.00012832
C	7.41552955	4.30068074	1.70320281	-0.00652871	0.00139686	0.01478775
C	6.01298108	6.65017353	1.80289080	-0.02149012	-0.02586423	-0.05590319
C	7.22767962	7.25063661	1.35590439	0.00613177	-0.00755465	-0.01703352
C	7.22034860	8.65497902	1.08358604	0.00203215	0.00420759	-0.00282062
C	8.43710136	0.83335810	0.85129352	-0.00025085	-0.00695258	0.00304180
C	8.43367580	2.25882832	0.82973737	-0.01060808	0.00280566	0.00027423
C	9.68011058	2.96608890	0.71291886	-0.00919063	0.00331620	-0.01298707
C	9.68772101	4.39017516	0.77517823	-0.01102171	0.00515006	-0.01568093
C	8.49490110	5.08031880	1.18041446	-0.00861220	0.00987334	-0.02650422
C	8.46169713	6.52210034	1.18700579	-0.00088383	0.00317678	-0.01107105
C	9.67516858	7.22581943	0.93924307	-0.00733633	-0.00607634	0.01065122
C	9.67531336	8.64969230	0.82705418	-0.00676378	-0.00173475	-0.00072187
Cu	6.65622680	4.58173291	5.25964738	-0.01972817	0.00810543	-0.00633433
Cu	4.77975866	5.87323755	4.77695617	0.03020041	0.03092233	0.00673570
Cu	6.87998547	6.91649632	4.14201165	0.01102058	0.02183228	0.09825069
Cu	4.63326512	3.53172418	3.92389394	0.01677322	-0.00454801	0.03530952
Cu	6.04499695	5.08097007	2.84763119	-0.00064604	-0.00604929	0.06120613
Cu	8.30121640	4.52973750	3.45814256	-0.01893768	-0.01680846	0.06345817
H	3.88794388	3.46273363	8.09810565	0.00512016	0.00777973	-0.01704438
O	3.49113576	4.10613343	7.41011496	0.01802919	0.00837958	-0.00161164
H	4.27524580	4.62232060	7.06280758	0.01947491	0.00328126	-0.00456486
H	0.79177457	8.30738744	3.02710899	-0.00873718	-0.00082221	0.01874471
O	0.98083754	7.95566883	3.93568708	-0.00603136	0.00412066	-0.02162230
H	1.81107290	7.37901859	3.86576961	0.00855677	-0.00217652	-0.01091799
H	2.44366188	2.25093737	5.19673399	0.01329998	0.01382715	-0.01168759
O	2.92023729	3.12095602	4.98051659	0.00440796	-0.00079381	-0.01830854
H	3.12171401	3.44918249	5.91722528	-0.00049190	0.00486337	0.00143870
H	7.46873294	3.13187754	9.46499875	-0.00474916	-0.00518839	0.00332258
O	7.08035582	2.25318457	9.78504658	0.00100550	-0.01359296	0.01054623
H	7.15711158	2.32788305	10.77430159	-0.00705903	0.00093162	-0.00642959
H	4.05007259	-1.16417737	5.90602609	0.01308404	0.00332286	-0.00167261
O	3.84470839	-0.57141383	6.70144254	-0.00565243	0.00673397	-0.00285675
H	4.81532024	-0.53255494	7.09856576	0.00300539	-0.00049089	-0.01367191
H	0.81591105	6.10731063	7.04096819	0.00686491	0.00264935	0.00278625
O	1.37947444	5.41096860	6.58749957	0.00297379	0.00811401	0.00193334
H	2.03108330	4.98449930	7.22503579	-0.00022853	0.01083322	-0.01013205
H	5.17493862	2.05993551	7.54694166	0.00466099	0.00211038	-0.01757336
O	5.61609190	1.92610491	6.62573175	-0.00728725	-0.01192960	0.00716611

H	5.70657827	2.85364593	6.21992253	0.00031523	-0.00567323	0.01235242
H	8.36573874	8.99800648	7.11938059	-0.01217579	0.00531390	0.00096071
O	8.12891788	9.83129036	6.63099187	-0.00170565	-0.00823072	0.00183821
H	7.12511702	9.97105807	6.79838487	-0.00538221	-0.00381491	-0.00656460
H	4.75364091	8.86869651	3.84507318	-0.00059902	-0.01209108	0.00398276
O	5.20163192	9.69434273	4.16593964	-0.00899274	0.00468956	-0.01503372
H	5.11466517	9.70588248	5.17516078	0.00427145	-0.01656976	-0.00086983
H	5.65203441	2.36271304	9.39470460	-0.00168989	-0.00622553	0.00188051
O	4.65933428	2.38809160	9.02116545	0.00173017	-0.01057612	0.00522516
H	4.28568102	1.49248065	9.36263114	0.00432074	-0.00304065	0.00294052
H	7.94757216	7.14342386	10.05810859	0.00357294	0.00502913	0.01036701
O	8.85943683	6.75651148	9.79392560	0.00345486	0.00318628	0.00998879
H	9.26285142	6.54228004	10.67731365	-0.00353199	0.00059983	-0.00242986
H	0.08916772	-0.53126800	9.24571931	0.00054166	0.00222100	0.00150186
O	0.93978590	-0.00097744	9.11194450	0.00131740	0.00206644	0.00988203
H	0.86073472	0.43482225	8.22663768	-0.00076317	-0.00527844	0.00216780
H	8.76746724	6.79775733	6.22842927	-0.02289783	-0.00392803	-0.00514893
O	9.35289221	7.32838853	6.84152368	-0.00419919	-0.00984521	-0.00416063
H	9.89344873	7.87037375	6.20826350	-0.01187012	-0.01240231	-0.00818573
H	6.31851204	-1.20079392	8.43057867	-0.00184879	0.00030681	-0.01652024
O	6.26703940	-0.89006593	7.45750191	0.00618614	0.00190136	-0.00072634
H	6.34303579	-1.69446292	6.86102620	0.00481231	-0.00355519	-0.00012564
H	8.45527116	2.99906745	6.46460160	0.00451274	0.00276925	-0.01117450
O	8.27634934	3.99001445	6.41614060	-0.00865126	0.00546193	-0.00087583
H	8.01467803	4.25626803	7.36228808	0.00843660	0.00089259	-0.00175028
H	7.91778364	0.63722045	5.19804568	-0.00200391	0.00724306	-0.00607434
O	7.72067847	0.31392205	4.23639107	0.00295270	-0.01365523	-0.02052621
H	6.82135740	0.76657501	4.05471123	-0.00025925	0.03578978	-0.01016479
H	2.21026059	5.82888856	5.39008319	-0.00199631	-0.00115471	-0.00522323
O	2.72729424	6.07851389	4.50797566	0.00077618	0.00438054	-0.01644890
H	2.58925885	5.28621149	3.91915390	-0.02789661	-0.00108309	-0.01694837
H	6.60665903	4.93450570	8.99972849	-0.00304897	-0.00090146	-0.01441593
O	7.56045182	4.68386587	8.83225885	-0.01926194	0.00914311	0.00094545
H	8.13954570	5.44664504	9.21571121	-0.00700252	0.00361676	-0.00161183
H	5.60530498	7.91446336	10.22038510	0.00591417	0.00285044	0.00914442
O	6.35430584	7.22645521	10.09126723	-0.00162788	0.00734561	0.02346473
H	5.89024344	6.36977882	10.34818156	-0.00113416	0.00409763	0.02108507
H	3.60284860	7.84259939	9.59694805	0.00139247	0.00168257	0.00724553
O	4.17451427	8.61023134	10.01482437	0.00123388	-0.00108306	0.01373627
H	3.78644979	8.77597179	10.91697220	0.00131118	-0.00514933	-0.00611690
H	2.54651799	8.96754280	6.29877990	0.00569268	-0.00880499	0.00023334
O	1.70292867	9.47522052	6.06484642	0.00446152	-0.00587694	-0.00428172
H	1.40168105	9.04731963	5.20419126	-0.00146276	-0.00336900	0.00022073
O	9.52578098	2.60330622	3.78913710	-0.00170428	-0.00240092	-0.01264329
O	9.61185255	5.61254357	4.47827004	0.01736817	-0.00749156	-0.02985319

H 8.88762022 1.83453720 3.73046858 -0.00461388 0.00537903 -0.01015836
H 10.00804835 2.56712688 2.92300570 0.01216439 -0.01030939 0.01091336
H 10.12171494 5.22107213 5.24463563 0.00034127 -0.00076889 -0.00662670
H 10.01410973 6.49226618 4.19580763 -0.01483818 -0.00045646 -0.00891537
O 2.81391235 6.76507202 8.87257374 0.00428391 -0.01136257 -0.00296702
H 3.16046040 7.05103067 7.96141211 0.00257105 -0.00328698 -0.00683370
H 1.97626824 7.38267456 8.97770278 0.00527123 -0.00602294 0.00110378
O 4.86537461 5.08671286 9.82186883 -0.00046211 -0.00091095 0.01121188
H 4.02178672 5.55338904 9.53215739 0.00458024 -0.00300849 0.00383716
H 4.58368092 4.16522982 10.06258748 0.00331465 0.01048786 0.01962438

*CO_2

115

Lattice="9.84 0.0 0.0 0.0 8.52169 0.0 0.0 0.0 11.75"
Properties=species:S:1:pos:R:3:momenta:R:3:forces:R:3:initial_charges:R:1

C 1.08229451 0.88045056 0.78943125 -0.63930134 -0.16052394 0.33046290 0.19414904
0.24282169 -0.05210249 -0.02200000

C 1.08600537 2.30595416 0.70659378 -0.19105792 -0.38048175 -0.41895856 0.03590796 -
0.31978919 0.01536221 -0.03430000

C 2.31702065 3.01527019 0.87166092 0.11595940 -0.51466081 -1.06830368 0.13051135
0.19470190 -0.00611485 -0.03390000

C 2.32391865 4.44529059 0.95726811 0.32783697 0.02121348 -0.50589512 -0.25586620
0.10636532 -0.03793914 -0.00810000

C 1.10080840 5.15217257 0.81195217 0.59602582 0.50143164 -0.58509512 -0.11051880 -
0.16511351 -0.10315250 -0.03390000

C 1.09314703 6.57464484 0.92130221 -0.20525025 0.54733435 -0.49562380 0.25155620 -
0.21256164 -0.05663073 -0.01570000

C 2.33074445 7.27186339 1.07846318 0.03434510 0.05680816 0.29229469 -0.07213176
0.00615233 0.07689521 -0.01840000

C 2.30903388 8.69490104 0.98326557 -0.66446264 -0.28307961 -0.16310956 0.35494039 -
0.00207708 -0.01416035 -0.05590000

C 3.54251062 0.88841841 1.08721719 1.27230180 0.95680518 -0.28136159 -0.56222714 -
0.19144878 -0.24955367 -0.08790000

C 3.53056817 2.30651921 1.05415168 -0.40355465 0.35516241 -0.00644324 -0.03806324 -
0.29634423 -0.21079716 0.02090000

C 4.72918881 2.99764849 1.40646446 -0.37178584 -0.70551825 0.30327444 0.20607498
0.36067759 0.06352498 -0.07850000

C 4.59930077 4.35247184 1.84989101 0.67362865 0.27773342 -0.11867492 -0.25629247 -
0.47112540 -0.40543329 -0.16700000

C 3.49163347 5.12991461 1.41825769 -0.23606263 0.36802061 -0.29603078 0.39122909 -
0.09977701 0.07777827 0.01930000

C 3.54379334 6.57254697 1.41472825 0.39393795 -0.67577886 0.45048746 -0.01786375 -
0.04187684 0.03168684 -0.02450000

C 4.76868555 7.31663833 1.58060158 0.20568490 0.24662989 0.83899419 0.29038346 -
0.02310993 0.06209486 -0.03090000

C 4.76026356 8.70436912 1.27963760 -0.91550268 -0.46142089 0.20026256 0.52131179
0.24480760 -0.10123946 -0.01790000
C 5.99848377 0.90443319 1.18399923 -0.53856550 0.85220013 -0.69239757 -0.01293082 -
0.58754378 0.01758445 -0.01360000
C 6.00715805 2.31498452 1.27356302 -0.01282290 0.04705230 0.26031008 -0.14286735
0.22728620 -0.10422628 -0.00800000
C 7.26499373 2.99391813 1.14498722 0.30934569 -1.17709664 -0.89498063 -0.41353432
0.62120152 -0.27893012 -0.05300000
C 7.41964802 4.34667919 1.59175228 0.29789071 -0.19205233 -0.42457998 -0.35551885 -
0.47123288 -0.27016532 -0.17740000
C 6.05527750 6.76927047 2.01654365 1.52652006 0.84990726 1.44197171 -0.07149589
0.56298497 0.14168064 -0.17460000
C 7.24584215 7.31431354 1.38934324 -0.30547354 0.49460576 0.41994779 -0.09046337
0.21563786 0.09683297 -0.03240000
C 7.23337287 8.70303588 1.06709944 0.66996163 -0.30286719 -0.21603556 -0.38924833
0.05531388 -0.23654603 -0.00620000
C 8.44712281 0.87152439 0.79799924 0.08454163 -0.93760360 -0.29443557 0.14055416
0.22190810 -0.07536797 -0.00500000
C 8.44517726 2.30080849 0.75610087 -0.39631604 -0.08889650 0.21816956 0.42576848 -
0.45882756 -0.16067148 -0.03500000
C 9.68797456 3.01157579 0.65048927 -0.32298134 -0.29705642 -0.03808223 -0.25153217
0.34251802 0.02102392 -0.01850000
C 9.70235656 4.44500961 0.73431482 -0.32001594 0.32022737 0.92279611 0.24033892 -
0.07148152 -0.58051824 -0.06040000
C 8.49761383 5.13618363 1.10843384 -0.92773859 0.17120916 -0.12446200 -0.13605083
0.19630046 0.18319184 -0.08860000
C 8.46651395 6.58153680 1.16834039 0.42276645 0.25266407 -0.51556007 -0.26968656 -
0.30147421 0.12459868 -0.00350000
C 9.69204375 7.27796996 0.93573182 0.73916955 -0.46476913 -0.15986008 -0.00238836
0.17281319 -0.09226126 -0.07430000
C 9.68894702 8.69579842 0.81207997 0.44223134 -0.87092211 0.64212888 -0.27861403 -
0.14635622 0.06386290 0.01750000
Cu 6.71437217 4.12316082 4.95361390 -0.53839682 -1.20513263 -1.01960576 -0.69023711
0.15325322 -0.47384121 0.28070000
Cu 4.68058170 5.45563720 4.78157479 -0.54756265 -0.19739332 -0.43672350 0.68141093
0.36929266 0.25018962 0.33560000
Cu 6.63600190 6.99543238 3.96212804 -1.17414548 -0.21337580 2.07849666 -0.26948616
-0.33245875 -0.29339858 0.53750000
Cu 4.73047456 3.22686915 3.69297219 2.04641124 2.49106798 1.85886711 0.52577825
0.32216241 -0.10113044 0.45430000
Cu 6.07589525 5.02834605 2.77442434 -2.67776385 -0.69864027 0.79705099 0.12939282
-0.30329265 0.20087247 0.26310000
Cu 8.41121849 4.56700721 3.29508538 1.14163176 -0.01624805 -0.59657082 -0.48122391
-0.02708449 -0.21701131 0.56990000

C 5.85533206 5.87371393 6.24285844 -0.03162323 -0.13095524 -0.14319609 -1.45219788 -0.04966701 -2.44656806 1.06760000

O 5.68940297 5.45054712 7.45531397 -0.10732169 -0.73972592 -0.45574435 0.16925626 -0.70819120 2.94696427 -1.14660000

O 6.87044633 6.73864367 5.97542406 0.37408554 0.61952348 -0.78435571 1.34192137 1.04758850 1.11020002 -1.09480000

H 3.64705015 3.35630930 8.06813954 -0.11405126 0.18185706 0.28551597 -0.00494496 0.13834133 -0.19231263 0.62690000

O 3.44181654 4.06317222 7.37050672 -0.11325822 -0.10512089 -0.52089073 -0.26310260 -0.23223073 0.56953114 -1.25160000

H 4.28437042 4.66348762 7.43559299 0.10343780 0.09508587 -0.09002650 0.48320337 0.29604325 -0.18550377 0.62510000

H 0.81004774 8.28969278 3.18904255 -0.34535853 0.22343113 0.01329258 0.06969523 -0.02627547 -0.23935819 0.61480000

O 1.12899097 7.92215503 4.06191960 0.21181268 -0.21453331 0.47760017 -0.30530793 0.30127948 0.16835376 -1.22980000

H 1.88421590 7.27086015 3.88395999 0.01770240 -0.01351196 0.03907274 0.28185152 -0.08813514 -0.19315971 0.63180000

H 2.49733037 2.13451098 5.25806043 0.04230277 0.25008948 0.19184150 -0.02270260 -0.00758768 -0.20138334 0.62750000

O 3.00347227 2.94090984 4.88746537 0.30735181 0.74092827 -0.30855978 -0.52649692 -0.03484887 0.71568681 -1.24380000

H 3.23694518 3.37814743 5.77864478 -0.02223772 0.14719323 0.08791032 0.09838507 -0.08544975 -0.54966974 0.60970000

H 7.44098532 3.20462835 9.32083426 -0.09004126 -0.05085604 -0.12467486 -0.00591076 -0.02149040 0.06180043 0.62900000

O 7.05834471 2.35370851 9.73302718 0.25934583 -0.57117865 0.17538779 -0.08257182 0.07935886 -0.23444625 -1.20790000

H 7.11090616 2.53405467 10.71441922 0.12445482 0.17975663 0.15168416 0.12737599 -0.03200784 0.45218058 0.57710000

H 4.18439250 -1.34015785 6.10338764 0.18756757 -0.32268268 -0.09225598 0.42597060 -0.65631422 -0.48456595 0.52650000

O 3.87176219 -0.57380899 6.70816539 0.35599333 1.10664476 0.13218837 -0.41300018 0.64240354 0.73098641 -1.22230000

H 4.75537220 -0.34989493 7.25041900 -0.10099998 0.12203997 -0.07839105 -0.20229986 -0.08095827 -0.18557379 0.63090000

H 0.91777778 6.01475372 6.98693724 0.04369326 -0.02917256 -0.15742052 -0.25133762 0.44155671 0.27622661 0.62490000

O 1.36015187 5.28844416 6.48217400 -1.00544560 1.10623114 -0.40735331 0.33111764 -0.81292456 0.44998666 -1.24740000

H 2.06667887 4.82805125 7.08555404 -0.02113058 -0.07347826 0.22051982 -0.19361690 0.24952707 -0.29410830 0.64380000

H 5.16017617 2.04560127 7.54111503 -0.18619504 0.07476868 -0.08827616 0.19492596 -0.05313842 -0.39151590 0.63370000

O 5.65516406 2.08987027 6.63893365 0.34748521 0.01276104 0.20337456 -0.22484139
0.15734268 0.82070269 -1.20160000

H 5.48900996 3.04373714 6.30323412 -0.01854369 0.43234960 -0.27626906 0.27843975 -
0.14180375 -0.49640806 0.52170000

H 8.69037967 8.96046565 7.04304678 -0.11364742 -0.33117798 0.10782530 -0.14114918
0.29433184 -0.20578381 0.61470000

O 8.32192843 9.84039349 6.67374050 -1.76786514 0.20844649 0.37816814 0.60585181 -
0.44150026 0.36067273 -1.23330000

H 7.38895783 9.93729003 7.00752319 0.03685100 0.19653798 -0.03245756 -0.50429741
0.04789023 0.01534195 0.64030000

H 4.59075467 8.95938972 4.16494338 -0.10122674 -0.09674300 -0.05615187 0.17876442
-0.12962779 -0.09365476 0.58440000

O 5.37466601 9.56203955 4.29457774 1.31084685 0.19834040 -0.21001739 -0.21336511 -
0.07940957 0.45105884 -1.21800000

H 5.35533120 9.81094462 5.30188148 -0.17506062 -0.01822874 0.03847416 -0.04229900
-0.10499132 -0.59343642 0.63350000

H 5.60947631 2.36364799 9.36006661 -0.05445454 0.02323794 0.00554038 0.25669852
0.03419283 0.05170461 0.63140000

O 4.60302095 2.34069170 9.03894071 0.31559571 -0.20019555 -0.77773124 -0.09356344
0.17788328 0.14499452 -1.27780000

H 4.29501278 1.45174437 9.43651092 0.23884625 0.13037388 0.20717893 -0.10983772 -
0.30801827 0.15150733 0.63430000

H 7.93654698 6.88344663 10.10929561 -0.13437498 0.07909160 0.06752533 0.27708007
-0.04194947 0.06476941 0.64250000

O 8.89731635 6.66955110 9.78555463 0.20670548 0.59443680 0.08204107 -0.38251671
0.12566774 -0.42669712 -1.21830000

H 9.35675628 6.36604936 10.60729679 -0.07726037 -0.13006478 0.02446069 0.17719388
-0.02776486 0.66326048 0.59410000

H 0.09372084 -0.50407311 9.30103659 -0.14150782 0.12110422 0.00746253 -0.13827416
-0.11538649 0.10160520 0.63270000

O 0.94353801 0.02933699 9.21846054 -0.73113469 -0.49051912 1.29372397 0.12408916
0.26249147 -0.08293046 -1.22190000

H 0.89671572 0.44843770 8.31295307 0.04998643 -0.00411414 -0.07149700 0.00316480 -
0.05297273 -0.09927017 0.60920000

H 8.45689360 6.90494073 6.66606988 -0.17936292 0.00365990 0.05904446 -0.49044633 -
0.17227223 -0.25859449 0.62680000

O 9.34651156 7.35025776 6.90461567 0.27944877 -0.66707588 0.38782442 0.01234229
0.07394127 0.60816105 -1.21090000

H 9.69134701 7.55123169 5.99967119 0.18142135 -0.17902426 0.05534424 0.17384009
0.09528321 -0.46464402 0.59990000

H 6.15895974 -0.79326812 8.71306570 0.04767326 -0.41236193 -0.14030115 -0.04777808
0.15175616 -0.22237226 0.61710000

O 6.08369380 -0.17966598 7.89845297 0.48317755 -1.15622554 -1.01450133 0.22240822
0.04835830 0.68997887 -1.22400000

H 6.55463599 -0.72465741 7.18236270 -0.00907553 -0.11617192 -0.23705206 0.02981598 -0.34622530 -0.51184142 0.60180000

H 8.61644546 2.93837049 6.27868902 0.00334545 0.20838731 0.09741426 0.06432453 -0.10722157 -0.09113348 0.63530000

O 8.34481672 3.88537307 6.12602363 -0.26153063 -0.32326567 -0.26728049 0.68302866 0.27176649 1.08794190 -1.21890000

H 8.23649410 4.23141841 7.10206378 0.10874090 0.04143554 0.13867803 0.05041769 -0.15987808 -0.53131847 0.63330000

H 8.17188081 0.91416682 5.11789236 0.13661174 0.09078525 0.27819822 -0.11577308 -0.15506880 -0.91995359 0.60650000

O 8.15140374 0.73747386 4.08920567 0.08653699 -1.71260835 -0.48345688 0.90321611 0.04530844 1.08288975 -1.24060000

H 7.23197062 1.00220598 3.85936967 0.28242225 -0.06434527 0.10195206 -0.79856176 0.17271997 -0.30154864 0.61740000

H 2.15548489 5.62861582 5.26671175 0.00243876 -0.01969241 -0.38591023 0.23686111 0.13686921 -0.27498749 0.63890000

O 2.68641766 5.80842981 4.37794753 -0.51898554 0.54251986 0.75836952 -0.47604028 0.17501636 0.03589232 -1.21620000

H 2.46810716 5.00842225 3.83437245 0.04425665 0.08425304 0.05799246 -0.05191798 -0.25248458 -0.46985835 0.62090000

H 6.98091959 5.03384842 8.26082738 -0.27523733 -0.07045818 -0.08992407 -0.44583058 0.18582776 -0.42557010 0.62190000

O 7.87254174 4.59845326 8.55664444 0.28115318 0.52271109 0.01132447 0.37775055 0.08702962 0.48046383 -1.24420000

H 8.41247541 5.34920392 9.01590815 0.18148360 0.16239090 0.10299580 -0.21842754 -0.12702170 -0.05816077 0.62620000

H 5.68637005 7.62099219 10.53673220 -0.05221602 -0.07608848 0.03334070 0.03699179 0.06164341 0.26668174 0.62310000

O 6.33564528 6.94213100 10.17891769 0.91897196 0.61631228 -0.46933816 -0.28078432 -0.30243105 -0.23559686 -1.25140000

H 5.75461187 6.08129012 10.17293456 0.12609592 -0.19610541 0.09304677 0.15668773 0.30815233 0.07178115 0.63770000

H 3.62898438 7.79434867 9.63478003 -0.03945314 0.03656194 0.15320560 0.08016112 0.17938204 0.09980974 0.61870000

O 4.15484070 8.53311851 10.13187060 -0.05031348 0.16796749 0.28137316 0.16109146 -0.10693909 -0.69539710 -1.22310000

H 3.60159605 8.73074571 10.92540389 0.11644342 0.14672901 0.06207929 -0.24579689 0.17697390 0.85870671 0.58760000

H 2.61651106 8.94066562 6.41627389 0.26652064 -0.13393356 -0.06689837 -0.11208674 0.06602757 -0.08600613 0.62280000

O 1.77757479 9.50845325 6.22448792 -0.47338534 -0.48005046 -0.35078966 0.09364112 -0.03049802 0.43449569 -1.23190000

H 1.38606784 9.01091661 5.44431177 -0.05660471 -0.18708286 -0.04141425 -0.04155554 -0.08314565 -0.17317960 0.60640000

O 9.74244926 2.87031622 3.70603703 0.03570389 0.27875331 0.31487541 0.44314316 -
0.36140335 -0.03240218 -1.19280000
O 9.41368258 5.78488246 4.50586606 0.12157853 -0.35005296 0.85378780 0.20783335
0.61015820 -0.17267002 -1.23630000
H 9.12170010 2.05801700 3.76407553 0.03637957 -0.01791859 0.00710271 -0.01208523
0.09234758 -0.10554459 0.62100000
H 10.28115431 2.68459547 2.88272025 0.24109336 -0.11193484 -0.06861885 -0.18700996
0.08100713 0.08125399 0.57160000
H 9.85779867 5.27612913 5.22761925 -0.32409005 0.21973426 0.07355732 0.23052314 -
0.14938704 0.17235296 0.65510000
H 10.00873481 6.55097979 4.20891603 0.06327449 0.14667495 -0.04359380 -0.11787656
-0.23027435 0.12066715 0.61900000
O 2.77483339 6.75029993 8.83552292 -0.82163209 -0.05936621 -1.00813619 -0.34345270
-0.34858985 0.63255248 -1.26290000
H 3.14550829 7.07337515 7.96319807 -0.25517668 0.00692432 0.19647926 0.38136881
0.26184703 -0.81616247 0.61840000
H 1.97038180 7.38602344 8.97442962 -0.06022899 -0.06301638 -0.17021604 -0.06529705
0.00110697 0.09210410 0.62220000
O 4.51216940 5.04588872 10.21751472 -0.11056688 0.81042573 -0.59466319 0.68393252
-0.55487054 0.25987367 -1.24680000
H 3.80524453 5.48853378 9.70144050 0.38921222 0.01425458 0.19060841 -0.61563108
0.34372434 -0.40550861 0.63770000
H 4.51526322 4.09352123 9.91595245 0.06111922 0.00777248 -0.09474395 -0.00539731
0.15389519 0.11935293 0.61400000

*HCOO;*OH (reduced CO_2 adsorption pathway)

115
Lattice="9.84 0.0 0.0 0.0 8.52169 0.0 0.0 0.0 11.75" Properties=species:S:1:pos:R:3:forces:R:3:initial_charges:R:1
C 1.18531970 0.79293649 0.82168232 -0.00209859 -0.00267429 -0.00100099 -0.01690000
C 1.18807698 2.21973992 0.73974046 0.00176280 -0.00531051 -0.00210934 -0.01780000
C 2.41721349 2.93375458 0.89959746 0.01318135 0.00198148 0.00595916 -0.00340000
C 2.42604639 4.35754051 0.98623816 0.01475794 0.00423401 0.01220425 -0.00860000
C 1.19992831 5.06361677 0.84904782 -0.00448526 0.00618812 0.00355955 -0.01490000
C 1.19782091 6.47731307 0.95160214 0.00165650 0.00131713 0.00509317 -0.02760000
C 2.43266661 7.18245529 1.12078882 0.00313353 -0.00568809 0.00455804 -0.01950000
C 2.40973916 8.60642169 1.02939769 0.00222290 -0.00453776 0.00500573 -0.04780000
C 3.63422386 0.79683217 1.10910765 0.00547709 0.00573211 0.01491845 -0.03910000
C 3.62955820 2.21233580 1.05582515 0.00390919 0.00533846 -0.01156758 -0.02880000
C 4.84388906 2.91029500 1.34383114 0.00830157 0.00676376 -0.01373195 -0.05840000
C 4.75068844 4.26598782 1.71541723 0.01655490 0.00913019 -0.00177614 -0.15260000
C 3.61197981 5.04715408 1.40053583 0.00518048 0.00991733 -0.01798719 0.01380000
C 3.64748214 6.49030330 1.43757778 0.00753820 -0.00228471 -0.00449004 -0.01260000
C 4.87736873 7.24361333 1.59460309 -0.01378448 -0.00224624 -0.00582774 -0.02800000
C 4.86614228 8.61919088 1.27088251 0.01021370 -0.01031021 0.01654948 -0.01610000
C 6.10566839 0.80785124 1.14500127 -0.00568313 0.00606995 0.00269235 -0.01870000

C	6.11506504	2.21067643	1.21269786	0.00805264	0.00725988	-0.00532037	-0.04030000
C	7.36105756	2.90232434	1.10011286	-0.01736698	0.00942801	-0.00424751	-0.03760000
C	7.49574238	4.24368811	1.55197992	-0.00948942	0.00586419	0.00989668	-0.16690000
C	6.16113483	6.69704208	2.00891144	0.00311429	-0.00609515	0.00913220	-0.16590000
C	7.34308036	7.22860438	1.37034126	0.00765590	-0.00941288	-0.02808848	-0.02170000
C	7.33386001	8.60912430	1.02632286	-0.00605112	-0.00785172	-0.00299343	0.00110000
C	8.55251890	0.78305940	0.77956605	-0.00047634	-0.00252345	-0.00268828	-0.01570000
C	8.55463844	2.20522630	0.74684742	-0.00659888	0.00265985	0.00007766	0.00030000
C	9.79436267	2.92654432	0.67534443	-0.00732150	0.00357006	-0.02062284	-0.00720000
C	9.80470074	4.35253338	0.76227638	-0.00953606	0.00486788	-0.01978943	-0.02520000
C	8.59226343	5.04769861	1.10587703	-0.01177356	0.00211614	-0.00466329	-0.07830000
C	8.56593328	6.48615644	1.16718791	-0.00835561	0.00245019	-0.01281324	-0.03220000
C	9.79132171	7.18611987	0.94623810	-0.00483780	-0.00802596	0.01228254	-0.05090000
C	9.79205543	8.60561629	0.81944216	-0.00403861	-0.00143819	0.00185923	-0.01910000
Cu	6.63387134	4.16148330	4.94595417	-0.02664921	-0.09168730	-0.04846511	0.22850000
Cu	4.59372934	5.47999201	4.42176325	-0.01228374	0.00441689	0.02597380	0.30260000
Cu	6.96665077	6.80535807	3.98807612	-0.00000090	0.04295022	0.01102344	0.57740000
Cu	4.79295490	3.16092320	3.64880033	0.01698657	0.01917750	0.02854427	0.47250000
Cu	6.17487529	4.94622536	2.73579723	0.00539089	-0.01174422	0.06253375	0.26920000
Cu	8.49879134	4.48675373	3.27356843	-0.01491713	-0.01910311	0.03965546	0.60030000
C	5.96809714	5.96968571	6.67565579	0.01710171	0.00710050	0.07171374	1.45810000
O	6.01663255	5.20950759	7.67426755	0.00682808	-0.00147937	0.02720173	-1.11440000
O	6.88445249	6.77624606	6.20816772	0.01402139	0.02204649	0.05294548	-1.13700000
H	3.67890044	3.35758449	8.03274434	0.00240495	0.00425379	-0.01598876	0.63260000
O	3.54931701	4.12474373	7.38905816	0.00931631	0.00612394	-0.00332965	-1.25760000
H	4.42533129	4.61497983	7.56522630	0.00351854	0.00342481	-0.01847338	0.61620000
H	0.96022140	8.25399371	3.23666939	-0.00665799	-0.00077240	0.00849235	0.60430000
O	1.10727493	7.95591794	4.16617979	0.00118968	0.01012646	-0.02276135	-1.24630000
H	1.78149190	7.17803424	4.13165816	0.00841020	-0.00125401	-0.00823009	0.63170000
H	2.42730206	2.23640925	5.29149322	0.01868593	0.01559538	-0.01399151	0.61640000
O	2.96052757	3.00921595	4.86254304	0.00627968	-0.00772482	-0.01269805	-1.24160000
H	3.24827066	3.47763544	5.70713324	-0.00178323	-0.00083571	0.00549988	0.60540000
H	7.69565830	3.22963862	9.37255872	-0.01077380	-0.00175513	0.00073044	0.63680000
O	7.06648543	2.46758028	9.73171475	-0.00288959	-0.01093208	0.01241803	-1.22140000
H	7.12194306	2.55160675	10.71363240	-0.00591974	0.00275893	-0.00395720	0.57180000
H	4.08825513	-0.86311342	6.02904708	0.00588736	-0.00259171	-0.00841586	0.56110000
O	3.82935032	-0.37591886	6.84863916	-0.00112404	-0.00208528	-0.01185128	-1.21390000
H	4.76167883	-0.22271425	7.38506429	0.00211816	0.00049585	-0.00482273	0.62760000
H	0.76118198	6.01542992	6.88586608	0.00465317	0.00168061	-0.00181425	0.64000000
O	1.32574803	5.24808192	6.53342392	-0.00667919	0.00308837	-0.00489433	-1.23490000
H	2.01792912	4.96371189	7.21244647	0.00013576	0.00721731	-0.01184295	0.64720000
H	5.20508204	2.07684908	7.57860669	0.00189232	0.01366878	-0.01857254	0.63590000
O	5.64028631	2.07798054	6.62655099	-0.00408612	-0.00146636	0.00351420	-1.20900000
H	5.45543295	2.99997457	6.27971447	0.00202867	-0.00003115	0.00471862	0.53250000
H	8.64468106	9.25288841	7.09881641	-0.01318663	0.01171194	-0.00010164	0.62100000

O	8.28648797	9.97284469	6.51649614	-0.00609854	-0.00970300	0.00438939	-1.24360000
H	7.34580213	10.17429006	6.82986076	-0.00427078	0.00664399	-0.00904512	0.64420000
H	4.45068681	9.04402290	4.08229345	0.00397944	-0.02187560	-0.00352926	0.59820000
O	5.22391573	9.62522013	4.27844028	-0.00700424	-0.00207333	-0.01160886	-1.21870000
H	5.21803866	9.82723030	5.29507109	0.01104838	-0.01953284	0.01238972	0.63530000
H	5.70048663	2.43833073	9.35108126	-0.00364228	-0.00439010	-0.00162919	0.63650000
O	4.67030516	2.36348881	9.02082429	-0.00242625	-0.01035693	0.00712061	-1.27310000
H	4.35118746	1.47592223	9.40705637	0.00492137	-0.00183882	0.00242704	0.63120000
H	7.75590295	6.91182193	10.02009935	0.00465761	0.00177856	0.00528545	0.63520000
O	8.73881202	6.77792409	9.77318182	-0.00291774	0.00304074	0.00594453	-1.21250000
H	9.16440698	6.65163243	10.65822487	-0.00113114	-0.00030258	-0.00032456	0.57990000
H	0.00564675	-0.47311730	9.24908565	-0.00099870	0.00222124	0.00275308	0.63460000
O	0.87646842	0.03455160	9.19923993	0.00118207	0.00320266	0.01169074	-1.22120000
H	0.86740691	0.46976414	8.30706322	-0.00002334	-0.00608578	0.00614173	0.60720000
H	8.57040193	6.93328637	6.76518228	-0.00825519	-0.00763956	-0.00933363	0.63100000
O	9.48173071	7.35512841	6.88553795	0.00133240	-0.01085389	-0.00593629	-1.21360000
H	9.68968713	7.55941551	5.93596689	-0.01340792	-0.00331659	0.00490223	0.59810000
H	6.08430742	-0.73678780	8.85359924	-0.00340874	0.00640864	-0.00916829	0.62180000
O	6.02899015	-0.08259546	8.06220171	0.00781607	0.00181330	0.00847554	-1.22110000
H	6.59179899	-0.52279312	7.36316076	-0.00436213	0.00299409	-0.00300199	0.60740000
H	8.85305424	3.33797087	6.05328985	0.01346420	0.00990213	-0.01393350	0.63660000
O	8.31846532	4.17733616	6.11079406	-0.01261809	-0.00204094	0.00228251	-1.20590000
H	8.05504664	0.80375665	5.08168860	-0.01142079	-0.00592718	-0.01653261	0.61710000
O	7.95285064	0.56046273	4.08195903	-0.00140486	-0.00932232	-0.02005023	-1.23820000
H	7.02560321	0.88261244	3.88826090	-0.00378437	0.03627861	-0.00921985	0.61890000
H	2.00202319	5.59371909	5.51942918	0.00494581	0.00040008	-0.00474152	0.62970000
O	2.59032915	5.85959658	4.52671788	0.01476832	0.00000882	-0.01355886	-1.19430000
H	2.29793891	5.10310594	3.95283833	-0.02446871	-0.01078086	-0.01902621	0.60160000
H	8.44272594	4.30522206	7.87695063	-0.01375029	0.01039976	-0.01033619	0.62140000
O	8.71602394	4.20346063	8.83514709	-0.00146594	0.01193755	-0.00332865	-1.24100000
H	8.65578364	5.14100730	9.19880624	-0.00545014	0.00179952	-0.00695534	0.61920000
H	5.52381588	7.59998797	10.60045595	0.00508074	-0.00088882	0.00691372	0.62220000
O	6.15891054	6.93647060	10.19714141	-0.00196048	-0.00104744	0.01974869	-1.25220000
H	5.57794710	6.07896303	10.14535618	0.00335220	0.00116862	0.00780071	0.64100000
H	3.51659777	7.81509067	9.63140638	0.00250438	0.00181048	0.00558080	0.62190000
O	4.02240273	8.56636942	10.12954443	-0.00062144	0.00041456	0.01104159	-1.22010000
H	3.41340561	8.79865297	10.87148064	-0.00054034	-0.00634592	-0.01118104	0.57800000
H	2.54235714	9.15635914	6.52760392	0.00587079	-0.00295875	0.00164551	0.62310000
O	1.71199629	9.67647091	6.26451512	-0.00120011	-0.00478967	-0.00571566	-1.23460000
H	1.34742361	9.11429527	5.51860755	-0.00329706	-0.00095839	-0.00056447	0.61470000
O	9.59434272	2.69487487	3.75115184	0.00366160	0.00400487	-0.01458295	-1.19430000
O	8.96529618	5.98034277	4.47871024	0.01299929	0.00685811	-0.02954609	-1.20080000
H	8.97246488	1.88195900	3.73486622	-0.00715357	-0.00424428	-0.00633871	0.62260000
H	10.26882979	2.50806045	3.05215034	0.00899294	-0.00994918	0.00915552	0.58590000
H	8.83712984	4.92962727	5.59215923	0.01148320	0.01651182	0.01848868	0.64180000

H	9.66773885	6.60947233	4.16143641	-0.00079337	0.00523816	-0.00213621	0.59230000
O	2.69229610	6.74774466	8.82959303	0.00850388	-0.01098206	-0.00663384	-1.25390000
H	3.08644819	7.10195221	7.96784815	0.00394811	-0.00660443	-0.00278240	0.61250000
H	1.87346821	7.37769319	8.96536615	0.00480934	-0.00703995	0.00053818	0.62210000
O	4.39065905	5.03328400	10.22932121	0.00069213	-0.00346333	0.01345626	-1.24090000
H	3.65972295	5.45918880	9.69232372	0.00284843	-0.00099433	0.00678719	0.63000000
H	4.47797537	4.10491794	9.87534734	0.00340083	0.00693258	0.01473671	0.61850000
H	4.94059106	6.05459778	6.14719701	-0.01910066	-0.00432671	-0.02777978	0.00520000

*HCOO

116

Lattice="9.84 0.0 0.0 0.0 8.52169 0.0 0.0 0.0 11.75" Properties=species:S:1:pos:R:3:forces:R:3

C	1.11420724	0.84240165	0.80373084	-0.00065317	-0.00378139	-0.00400286
C	1.11756041	2.27070710	0.72016352	0.00150561	-0.00548111	-0.00037804
C	2.34764205	2.98112995	0.88484198	0.01435830	0.00256708	0.00624440
C	2.35541537	4.40884148	0.98043968	0.01429910	0.00516009	0.01420406
C	1.12800357	5.11187251	0.84318188	-0.00422246	0.00681531	0.00695484
C	1.12578589	6.53227534	0.94949059	0.00327007	-0.00020627	0.00732401
C	2.36261662	7.23133659	1.10614698	0.00369704	-0.00696504	0.00274937
C	2.33952394	8.65626215	1.00316408	0.00225770	-0.00335160	0.00289464
C	3.56542399	0.84617654	1.08839927	0.00411314	0.00494501	0.01495653
C	3.56181647	2.26630884	1.04664638	0.00286515	0.00544439	-0.01055909
C	4.76765877	2.96178298	1.37832830	0.00783694	0.00565521	-0.01742980
C	4.65686325	4.30916550	1.80984164	0.01333283	0.00632636	0.00398617
C	3.53490583	5.09178767	1.42713027	0.00740633	0.01245683	-0.00877812
C	3.57636368	6.53974576	1.44003317	0.01181094	-0.00647626	0.00902468
C	4.80545683	7.28755147	1.59434634	-0.01627166	-0.00830838	-0.00020481
C	4.79531370	8.66964450	1.26460478	0.01060169	-0.01112873	0.01485929
C	6.02981241	0.86243230	1.14919407	-0.00438075	0.00456029	0.00259725
C	6.03915095	2.27217889	1.23214108	0.00792814	0.00740744	-0.00574090
C	7.29052855	2.95786581	1.12966177	-0.01734304	0.00792077	-0.00483623
C	7.44590280	4.29432093	1.60970160	-0.00762050	0.00611689	0.01173693
C	6.08755434	6.74928871	2.02581805	0.00230106	-0.00723505	0.01106239
C	7.27232252	7.28212558	1.38628231	0.01040334	-0.01142511	-0.02732710
C	7.25794672	8.66493948	1.03470241	-0.00495390	-0.00660202	-0.00530299
C	8.47647499	0.83612114	0.78350080	0.00072407	-0.00391076	-0.00201500
C	8.47807689	2.26248463	0.74955244	-0.00786571	0.00195426	-0.00057841
C	9.72055512	2.97570450	0.66303844	-0.00727155	0.00305614	-0.01860212
C	9.73196517	4.40628062	0.75399546	-0.00955824	0.00619545	-0.01748637
C	8.52622385	5.09807440	1.12828656	-0.01162777	0.00452547	-0.00881691
C	8.49377262	6.54180583	1.18170657	-0.00840106	0.00255362	-0.00894875
C	9.71906336	7.23915309	0.95014280	-0.00526834	-0.00798677	0.01211728
C	9.71698768	8.65988768	0.81723960	-0.00531314	-0.00151047	0.00005780
Cu	6.75186145	4.29551923	5.05738356	-0.01113320	-0.02667143	-0.01378330
Cu	4.71635653	5.47315210	4.68629146	0.02245358	0.01529468	0.02228502

Cu	6.75514037	6.94594697	3.98515812	-0.01922882	0.04683519	0.06521855
Cu	4.77857807	3.23690829	3.69914433	0.00419621	0.01791141	0.02733052
Cu	6.12320828	5.00071237	2.78254423	-0.00083243	-0.00893950	0.06008063
Cu	8.43861360	4.47952816	3.32384117	-0.02030375	-0.01052363	0.05968464
C	5.93267905	5.84798902	6.35780023	-0.00519487	-0.00796546	-0.00062884
O	5.84676876	5.22309363	7.51538622	-0.00562623	0.00557031	0.02905154
O	6.96581280	6.71179995	6.06110889	0.02036734	0.01279685	0.00591082
H	3.64250168	3.35274546	8.06763621	0.00478674	0.00470428	-0.01581539
O	3.46000254	4.09184678	7.40671177	0.01047399	0.00144068	-0.00038404
H	4.34239760	4.61682929	7.49244515	0.00929103	0.00486039	-0.01770464
H	0.88599978	8.29266328	3.13074148	-0.00747766	0.00035520	0.01606302
O	1.12725601	7.94634857	4.02667462	0.00128913	0.00801268	-0.02197771
H	1.89609977	7.29661298	3.89828479	0.00913869	-0.00065706	-0.00847024
H	2.39849111	2.28373679	5.20850967	0.01866019	0.01641743	-0.01438403
O	2.97053508	3.06983399	4.87580092	0.00511603	-0.00869849	-0.01322309
H	3.24660675	3.44543896	5.77249612	-0.00133845	-0.00245392	0.00358272
H	7.54887195	3.15684161	9.35227759	-0.00793939	-0.00277602	-0.00097943
O	7.08840510	2.33022709	9.74275704	-0.00030347	-0.01096571	0.00972230
H	7.12727111	2.49352106	10.71967400	-0.00617379	0.00300763	-0.00516529
H	3.91402516	-0.78447423	6.00776827	0.00897187	-0.00511021	-0.00874830
O	3.73436696	-0.33385959	6.86809852	-0.00478409	0.00271834	-0.01063812
H	4.73149396	-0.24503617	7.33905899	0.00556740	-0.00047623	-0.00482508
H	0.90371198	6.08092834	6.98514852	0.00679650	0.00318340	0.00280686
O	1.40199557	5.36061988	6.49607328	0.00039210	0.00520998	0.00310753
H	2.07294597	4.89792700	7.11123994	0.00220431	0.01188943	-0.01075575
H	5.17397119	2.08619407	7.55260573	0.00536852	0.01192258	-0.01870188
O	5.64188618	2.06981113	6.62793824	-0.00289205	0.00024542	0.00638992
H	5.59350745	3.01295644	6.28913813	-0.00224561	0.00195480	0.00468581
H	8.64920393	8.97694189	7.05002953	-0.01056896	0.00433525	-0.00082691
O	8.25924759	9.79718094	6.62469131	-0.00960753	-0.00494938	0.00203926
H	7.31457083	9.91349893	6.95261277	0.00037720	0.00796045	-0.00999299
H	4.58623921	9.06258585	4.01642643	0.00596489	-0.02291392	-0.00010166
O	5.30999511	9.68292031	4.27146072	-0.00865635	0.00439306	-0.01038652
H	5.26527181	9.85367361	5.29373074	0.01109678	-0.02139838	0.00998873
H	5.67763726	2.36073785	9.35617720	-0.00325227	-0.00384501	-0.00213903
O	4.66117238	2.34788988	9.02873900	0.00028283	-0.00739552	0.00527309
H	4.33180091	1.45479175	9.40719040	0.00431552	-0.00160288	0.00242341
H	7.84507105	6.95074373	10.03011086	0.00391188	0.00375009	0.00564732
O	8.82740034	6.77199605	9.78615244	-0.00153206	0.00482658	0.00677331
H	9.23084741	6.56304306	10.66655412	-0.00192116	0.00039876	-0.00223830
H	0.05344227	-0.45845294	9.32234407	-0.00142065	0.00230994	0.00101836
O	0.91267757	0.06510688	9.22409322	0.00180033	0.00285863	0.01113520
H	0.85094892	0.48003283	8.32680173	0.00073364	-0.00553703	0.00128417
H	8.55006798	6.89518224	6.63724392	-0.01306092	-0.01575206	-0.01542380
O	9.42530274	7.35842370	6.88502454	0.00067602	-0.00941010	-0.00146074

H	9.75124682	7.63681615	5.99280549	-0.01373715	-0.00240883	0.00120621
H	6.08945537	-0.82037740	8.70219361	-0.00216446	0.00200405	-0.01233585
O	6.01676930	-0.20972185	7.88147949	0.00304229	-0.00707148	0.00956876
H	6.51689353	-0.72459557	7.16156903	-0.00469574	-0.00524935	-0.00768957
H	8.63575639	2.87757328	6.23669568	0.00796497	0.00358677	-0.00986126
O	8.45887074	3.86221521	6.11999194	-0.01580896	-0.00181084	-0.00004977
H	8.41313508	4.18192076	7.09822817	0.00238090	0.00545955	-0.00980155
H	8.07357689	0.77818738	5.08658314	-0.01233830	-0.00379562	-0.01592501
O	8.01836996	0.56417751	4.08293506	0.00081362	-0.00936336	-0.01803833
H	7.11610101	0.93627312	3.85801060	-0.00027684	0.04162897	-0.00799696
H	2.19530334	5.69956699	5.34369731	-0.00208217	0.00132119	-0.00358477
O	2.72921694	5.88526414	4.42684302	0.01246940	0.00342300	-0.01359653
H	2.47887013	5.09241277	3.87708374	-0.01906561	-0.00864683	-0.01957431
H	7.14779434	4.86498645	8.25524449	-0.00885034	0.00161770	-0.00917320
O	8.05321588	4.50565780	8.60163788	-0.00734179	0.00790162	0.00209195
H	8.48120023	5.30458868	9.06353037	-0.00660725	0.00345869	-0.00610203
H	5.62068216	7.63063033	10.49859785	0.00499671	0.00096361	0.00959270
O	6.25141994	6.94443662	10.11938500	-0.00167179	0.00007532	0.01684740
H	5.67022521	6.08622016	10.13228547	0.00397514	0.00062598	0.00668463
H	3.57745492	7.81146209	9.66133817	0.00200374	0.00160257	0.00569673
O	4.13109311	8.55045934	10.13059079	0.00087875	-0.00003885	0.01103971
H	3.59466460	8.77991513	10.92918548	-0.00057176	-0.00581376	-0.01099944
H	2.46694331	9.18481525	6.47535825	0.00645066	-0.00470668	0.00162918
O	1.64692178	9.68057361	6.13926810	-0.00756889	-0.00627300	-0.00465376
H	1.35494593	9.11734786	5.36717272	-0.00352734	-0.00057656	-0.00036487
O	9.70078798	2.71670747	3.70503010	0.00269392	0.00398620	-0.01216893
O	9.50967340	5.73816832	4.44936733	0.01075208	-0.00136817	-0.02143806
H	9.07588404	1.91660555	3.72339036	-0.00308282	-0.00492413	-0.00572649
H	10.22814547	2.58231798	2.87673584	0.01112684	-0.01002378	0.01266080
H	9.96975639	5.28632632	5.21422238	-0.00133060	0.00920055	-0.00222583
H	10.02209201	6.55884831	4.17895337	-0.01934332	-0.00604988	-0.00912516
O	2.72737901	6.75964046	8.87995185	0.00789208	-0.01272562	-0.00743848
H	3.09277451	7.10998797	8.00268303	0.00577329	-0.00866060	-0.00430437
H	1.91785443	7.39593803	9.02289017	0.00547771	-0.00693645	0.00102252
O	4.49339178	5.03384765	10.22620962	0.00414837	-0.00429558	0.01154338
H	3.74836713	5.46190347	9.71240441	0.00365473	-0.00175901	0.00505586
H	4.54676733	4.09730321	9.88974447	0.00559784	0.00752196	0.01238392
H	4.90344211	6.38505371	6.15931619	0.00127294	-0.01829250	-0.02735906

*COOH

116

Lattice="9.84 0.0 0.0 0.0 8.52169 0.0 0.0 0.0 11.75" Properties=species:S:1:pos:R:3:forces:R:3

C	1.08219741	0.85774827	0.78297108	-0.00101698	-0.00347748	-0.00283019
C	1.08489755	2.28391490	0.70056909	0.00151746	-0.00522726	-0.00059407
C	2.31524799	2.99215489	0.85667382	0.01296708	0.00299972	0.00458291

C	2.32039402	4.42086649	0.93999904	0.01364764	0.00567086	0.01213709
C	1.09395935	5.12554099	0.80304236	-0.00438185	0.00613419	0.00742796
C	1.09130763	6.54722010	0.91375762	0.00407244	-0.00000459	0.00861895
C	2.32861023	7.24587866	1.06247352	0.00311953	-0.00727158	0.00243857
C	2.30897864	8.67077887	0.96896844	0.00216668	-0.00375161	0.00240080
C	3.53524930	0.86012288	1.06479257	0.00348106	0.00325599	0.01422912
C	3.53246301	2.28093802	1.02563341	0.00351853	0.00470818	-0.01110252
C	4.73154032	2.97625149	1.37100758	0.00915424	0.00338988	-0.03130149
C	4.59870629	4.32124856	1.82297365	0.01381326	0.00670302	0.00417629
C	3.49493551	5.10288550	1.39222983	0.00867141	0.01475018	-0.00766710
C	3.54105871	6.55037234	1.39076856	0.01330875	-0.00659110	0.00890442
C	4.76729029	7.29438175	1.55478546	-0.01604136	-0.00983122	-0.00129427
C	4.76295911	8.68350479	1.25309310	0.01003073	-0.01028429	0.01483353
C	5.99869524	0.87875019	1.15802525	-0.00378284	0.00387100	0.00278751
C	6.00497717	2.29069888	1.24413275	0.00812531	0.00663587	-0.00535325
C	7.26131686	2.97668727	1.13845362	-0.01697886	0.00748674	-0.00409336
C	7.41530586	4.31742512	1.60488637	-0.00807740	0.00565761	0.01136096
C	6.04349599	6.74813974	1.99467080	0.00136184	-0.00748087	0.01094054
C	7.24322723	7.29286796	1.38055371	0.01212313	-0.01013526	-0.03658948
C	7.22781405	8.68108010	1.04536828	-0.00517172	-0.00494825	-0.00841754
C	8.44476046	0.85272643	0.78856744	0.00058936	-0.00410967	-0.00153832
C	8.44515032	2.27874761	0.74966018	-0.00805546	0.00343388	0.00153247
C	9.68737790	2.98935310	0.64925830	-0.00832971	0.00400399	-0.01691009
C	9.69787844	4.41992583	0.72706651	-0.01000753	0.00512982	-0.01614389
C	8.49638838	5.10953796	1.11662523	-0.01103091	0.00483357	-0.01050833
C	8.46278424	6.55579753	1.17414500	-0.00815325	0.00197181	-0.00823786
C	9.68580071	7.25487158	0.93601211	-0.00494113	-0.00826129	0.01252564
C	9.68471045	8.67569180	0.80796754	-0.00580563	-0.00205672	-0.00018148
Cu	6.67183127	4.30513298	5.03836214	-0.00548977	-0.03857825	-0.01037109
Cu	4.61515936	5.56997400	4.73927927	0.02239293	0.00763298	0.02072132
Cu	6.66897351	7.01005795	3.92197293	-0.01666803	0.04673172	0.06526864
Cu	4.71315919	3.28421520	3.73996729	0.00744172	0.00154994	0.02969189
Cu	6.06536449	5.01071286	2.76879100	0.00230924	-0.01022120	0.04879293
Cu	8.37791418	4.53874075	3.32928041	-0.02033437	-0.01497442	0.05492482
C	5.84499717	5.96099858	6.11381489	0.00076506	-0.00625271	-0.00023083
O	5.66493876	5.47251745	7.50345710	-0.00530889	0.00190406	0.02317365
O	6.89267951	6.79825827	6.00503278	0.01242025	0.01248082	0.01140281
H	3.63847466	3.32572290	8.08810454	0.00518443	0.00635627	-0.01523191
O	3.37659784	4.03568007	7.41513372	0.01517788	0.00455693	0.00054370
H	4.17733752	4.65034459	7.45420443	0.01077658	0.00251015	-0.01822754
H	0.83472627	8.31591400	3.14022284	-0.00656969	0.00002891	0.01343139
O	1.10899076	7.94881356	4.01791323	0.00035153	0.00768338	-0.02277921
H	1.86696831	7.29874862	3.85361839	0.01067913	-0.00116240	-0.00790940
H	2.47323999	2.13307972	5.24315409	0.01712981	0.01605318	-0.01385693
O	2.98118096	2.95310951	4.90329811	0.00493048	-0.00469191	-0.01583822

H	3.20237536	3.37583921	5.79327392	0.00014244	0.00252611	0.00154393
H	7.48845918	3.17227762	9.35809254	-0.00767837	-0.00275941	0.00012745
O	7.06540162	2.33647634	9.75275003	-0.00005573	-0.01072949	0.01005314
H	7.12916102	2.49581352	10.73080047	-0.00628785	0.00291036	-0.00507276
H	4.25444134	-1.34045458	6.12810715	0.00693330	0.00301399	-0.00425826
O	3.82477660	-0.59460605	6.69948629	-0.00138652	0.00464590	-0.00208711
H	4.64124282	-0.28090978	7.24634995	0.00184392	0.00195129	-0.01008432
H	0.88541234	6.03944838	6.96670462	0.00809804	0.00334102	0.00285919
O	1.34867409	5.30735198	6.46712199	-0.00064243	0.00685592	0.00258641
H	2.00923529	4.82846749	7.07806248	0.00199723	0.01259866	-0.01005433
H	5.17684956	2.10347941	7.55382057	0.00473475	0.01007944	-0.01980407
O	5.64815949	2.06424661	6.64394007	-0.00358011	-0.00000670	0.00696497
H	5.56805677	2.98277800	6.23542088	0.00044835	0.00431491	0.01420364
H	8.65063369	8.99185907	7.03698248	-0.00924026	0.00481139	-0.00077525
O	8.27356530	9.83243698	6.63916910	-0.00906883	-0.00651749	0.00212538
H	7.32130364	9.93545570	6.95093827	0.00001830	0.00735846	-0.01092661
H	4.59778137	8.96664111	4.05940313	0.00341196	-0.00826252	-0.00212829
O	5.32990499	9.59595520	4.26613746	-0.00856944	0.00329322	-0.00905875
H	5.28956158	9.76953237	5.27441280	0.01078228	-0.02065275	0.00567123
H	5.63941992	2.37502809	9.38336984	-0.00279202	-0.00387079	-0.00057464
O	4.62049403	2.36422158	9.07337896	0.00072279	-0.00683923	0.00575158
H	4.30701792	1.45976627	9.44255488	0.00420200	-0.00212628	0.00384391
H	7.91920843	6.92205274	10.14665114	0.00225269	0.00312396	0.00678911
O	8.86317459	6.72748721	9.80517843	-0.00216670	0.00573931	0.00695590
H	9.34315535	6.45420430	10.62848494	-0.00263524	0.00181844	-0.00551507
H	0.07823882	-0.47543474	9.28599510	-0.00186080	0.00266602	0.00086514
O	0.93602478	0.04292336	9.19350185	0.00133372	0.00213568	0.01122542
H	0.89957630	0.44462818	8.28597110	-0.00075378	-0.00670683	0.00460659
H	8.50960881	6.89765210	6.61552474	-0.01283736	-0.01530298	-0.01353008
O	9.37572752	7.35604022	6.88382393	-0.00117429	-0.00909615	-0.00036174
H	9.72120673	7.60342894	5.98907374	-0.01350239	0.00043511	0.00280836
H	6.27990364	-0.66264847	8.80809896	0.00017427	0.00587671	-0.00865585
O	6.07266897	-0.15956809	7.95732502	0.00209765	-0.00037039	0.00986274
H	6.56107483	-0.65022995	7.22019561	-0.00448312	-0.00281050	-0.00769633
H	8.57728406	2.92669113	6.25668816	0.00706140	0.00558194	-0.00946587
O	8.36139095	3.90308349	6.15223406	-0.01586182	-0.00091790	0.00019449
H	8.31444933	4.21887158	7.12491418	0.00103695	0.00580413	-0.01115698
H	8.11086737	0.87241987	5.08265443	-0.01403482	-0.00613199	-0.01693655
O	8.08191557	0.69677055	4.07258623	0.00074555	-0.00874978	-0.01852792
H	7.16287552	1.01620347	3.83935599	-0.00033659	0.03977051	-0.00758464
H	2.12456162	5.63223452	5.24513005	-0.00743324	-0.00098442	-0.00460622
O	2.63836747	5.81651294	4.33206360	0.00868353	0.00352704	-0.01418089
H	2.41155759	5.02791754	3.76972460	-0.02091687	-0.01238503	-0.01753108
H	7.04673926	4.95315240	8.28621807	-0.01037688	0.00084020	-0.01107659
O	7.93025044	4.58207145	8.60808198	-0.01330019	0.00889965	0.00097312

H	8.40152080	5.36834826	9.06381883	-0.00838416	0.00454010	-0.00558455
H	5.60908582	7.63954019	10.51546481	0.00613275	-0.00019232	0.00794253
O	6.28415751	6.94753068	10.21553991	0.00088115	0.00123492	0.02350471
H	5.72135208	6.08614881	10.26893220	0.00441820	0.00024220	0.01433672
H	3.60314914	7.81184794	9.63293533	0.00277938	0.00065301	0.00524723
O	4.16195362	8.53845153	10.11817927	0.00248905	-0.00262294	0.00902919
H	3.64086124	8.74120997	10.93633572	0.00042980	-0.00537245	-0.01034096
H	2.56784269	8.94287261	6.39211864	0.00447413	-0.00654875	-0.00107780
O	1.74397502	9.50964743	6.21791057	-0.00186733	-0.00805680	-0.00543091
H	1.35363338	9.04563118	5.42253396	-0.00304196	-0.00146355	-0.00005371
O	9.72972670	2.83200604	3.71893305	0.00540782	0.00567014	-0.01159412
O	9.42472269	5.79105693	4.48446622	0.00940877	-0.00435172	-0.02434422
H	9.10795109	2.02395805	3.74522309	-0.00198118	-0.00651612	-0.00555190
H	10.25019592	2.69532883	2.88732553	0.01065520	-0.00805638	0.01173687
H	9.91121056	5.29485534	5.20539882	0.00025875	0.00896582	-0.00135274
H	9.98616733	6.56499933	4.17388699	-0.01730916	-0.00357067	-0.00873662
O	2.75049094	6.75961093	8.85371035	0.00734649	-0.01236806	-0.00518982
H	3.09991964	7.07231079	7.95163651	0.00462972	-0.00809876	-0.00483196
H	1.93883644	7.39825438	8.99820407	0.00602241	-0.00729786	0.00037839
O	4.51608560	5.02642319	10.20053468	0.00503911	-0.00228215	0.01039151
H	3.73199270	5.44962735	9.74552188	0.00231924	-0.00160966	0.00596068
H	4.49877371	4.06168896	9.94238371	0.00612564	0.00852440	0.01151861
H	5.52002550	6.28663599	8.06740880	-0.00706143	-0.01893368	-0.01395819

*CO;*OH

116

Lattice="9.84 0.0 0.0 0.0 8.52169 0.0 0.0 0.0 11.75" Properties=species:S:1:pos:R:3:forces:R:3

C	1.05595989	0.82360871	0.77074589	-0.00077029	-0.00340367	-0.00307685
C	1.05584964	2.25105174	0.68704124	0.00210081	-0.00526132	-0.00089598
C	2.28742256	2.95981276	0.84489376	0.00914561	0.00322845	0.00562443
C	2.28895601	4.38618738	0.94354952	0.00811170	0.00635598	0.01700657
C	1.06308234	5.09323185	0.81051698	-0.00087393	0.00570953	0.00672041
C	1.06503221	6.51384131	0.91946603	0.00623884	-0.00010568	0.00838091
C	2.30500281	7.21232068	1.06357323	0.00476740	-0.00811409	0.00070051
C	2.28490761	8.63652005	0.95204087	0.00267711	-0.00534601	0.00200413
C	3.51256932	0.82323734	1.03685556	0.00140809	-0.00032572	0.01138064
C	3.50645260	2.24627854	1.00277491	0.00366707	0.00411735	-0.01162877
C	4.70636840	2.94107208	1.34639350	0.01097955	-0.00034540	-0.04207706
C	4.58502330	4.28109446	1.81031641	0.01310460	0.00671956	0.00928892
C	3.46965200	5.06827996	1.40513855	0.00461334	0.01758957	0.00156712
C	3.51629262	6.52212802	1.41129826	0.01192152	-0.00221020	0.01212009
C	4.74830884	7.26560996	1.57970188	-0.01768019	-0.01066599	-0.00031813
C	4.74085623	8.64749272	1.23727091	0.00992611	-0.01413223	0.01175553
C	5.97395803	0.84291541	1.13875771	-0.00300789	0.00031718	0.00537276
C	5.97929167	2.25444330	1.21951055	0.00901786	0.00783546	-0.00337352

C	7.23091190	2.94405192	1.12370752	-0.01556045	0.00954361	-0.00284881
C	7.37657836	4.28506452	1.59481092	-0.00882056	0.00467817	0.00829014
C	6.02241705	6.72277204	2.02555029	-0.00002181	-0.00762958	0.00895560
C	7.21497282	7.26335014	1.40271316	0.01072580	-0.01041177	-0.03239886
C	7.20162659	8.64673782	1.04463733	-0.00491576	-0.00655820	-0.00315103
C	8.41770516	0.81946719	0.78483890	0.00059462	-0.00311449	-0.00166570
C	8.41772133	2.24521517	0.74366247	-0.00559246	0.00296525	0.00257153
C	9.65816076	2.95735945	0.64390814	-0.00786781	0.00418244	-0.01786695
C	9.66615431	4.38833526	0.72720059	-0.00939687	0.00379239	-0.01619146
C	8.46236999	5.07920335	1.11380555	-0.01225110	0.00431302	-0.00988784
C	8.43704073	6.52390540	1.18649819	-0.00838466	0.00214442	-0.01034078
C	9.65855704	7.22207537	0.94346892	-0.00535327	-0.00885847	0.01277391
C	9.65804530	8.64293472	0.80848591	-0.00537963	-0.00324866	0.00079536
Cu	6.70859014	4.13283241	4.99456084	0.00431143	-0.06649362	-0.05242809
Cu	4.76537097	5.51266583	4.80582118	0.03875463	0.00420611	0.01037772
Cu	6.75287906	6.88524516	3.97277928	-0.01660937	0.06272378	0.05858402
Cu	4.66252933	3.27099692	3.76109008	0.00047143	0.00895165	0.03348199
Cu	6.04555824	4.96850392	2.79670473	0.00070908	-0.00432199	0.05643526
Cu	8.38518408	4.47299455	3.30694280	-0.03451693	-0.01416437	0.06154712
C	5.11578189	5.34999243	6.60348460	-0.00815954	0.00082229	0.01888135
O	5.59481921	5.15348199	7.67599434	-0.01069483	0.00516683	0.01465831
O	7.53492579	7.01077703	5.78461034	0.00383099	0.00672551	-0.00993909
H	3.37320704	3.20029274	8.20223261	0.00292660	0.00799417	-0.00884856
O	2.83491789	3.89616388	7.68509204	0.01339572	0.00374039	-0.00393991
H	2.99158784	4.74819273	8.18011934	0.01102286	-0.00332020	-0.01197884
H	0.87022315	8.42954500	3.01636348	-0.00684359	-0.00003626	0.01790637
O	1.07072136	8.10644249	3.92949445	-0.00647196	0.00361289	-0.01723480
H	1.81249630	7.42481106	3.84271339	0.00805170	-0.00098426	-0.00879449
H	2.47566238	2.23946386	5.28487696	0.01415521	0.01407968	-0.01062397
O	2.94429332	3.09333046	4.96797389	0.00109692	0.00221983	-0.01594118
H	3.10104721	3.53332429	5.85548543	0.00649326	0.00206633	-0.00004112
H	7.43408524	3.03801345	9.41567015	-0.00497398	-0.00142563	-0.00027781
O	6.83946235	2.31780054	9.79311655	0.00015672	-0.00930908	0.01121889
H	6.79482289	2.56188511	10.75375152	-0.00888708	0.00539083	-0.00283674
H	3.98930893	-1.26834281	6.15401998	0.00109149	0.01557207	0.00409428
O	3.83405410	-0.56684188	6.84137035	-0.00981373	0.00760851	-0.00950113
H	4.86930580	-0.45545610	7.24217475	0.00536664	-0.00297640	-0.00942972
H	0.77350731	6.20566910	6.75179173	0.00230711	0.00177242	0.00117694
O	1.27062923	5.42808114	6.28670492	0.00142789	0.00366321	0.00342571
H	1.59132915	4.71805759	6.92354652	-0.00270656	0.00847805	-0.00879945
H	5.08702239	1.92564041	7.46845756	0.00534700	0.00738426	-0.01904238
O	5.63158765	1.91170345	6.55756103	-0.00307324	-0.00389500	0.00524335
H	5.57548729	2.84823270	6.19726737	-0.00267860	-0.00181455	0.01333889
H	8.61850924	9.14145945	7.17577538	-0.01248763	0.00538243	0.00122672
O	8.24091005	9.87833603	6.62714106	-0.00471212	-0.01034717	-0.00084803

H	7.24984224	9.93862886	6.84434953	-0.00134828	0.00860824	-0.01001091
H	4.62615987	8.93492196	3.92264667	0.00427220	-0.01018681	0.00268718
O	5.24382230	9.66424170	4.17229085	-0.00916315	0.00149796	-0.01283625
H	5.24589496	9.75209031	5.20668355	0.01081295	-0.02113769	0.00660515
H	5.37771030	2.36968530	9.24388196	-0.00441414	0.00072617	-0.00085891
O	4.47036247	2.14850934	8.78444667	-0.00134838	-0.00176832	0.00391514
H	4.24673039	1.24647169	9.21691562	0.00242837	-0.00372149	0.00011134
H	8.05078956	6.89222290	10.03970520	0.00392258	0.00255472	0.00677439
O	9.01254630	6.62190066	9.79060793	0.00141360	0.00621447	0.00790146
H	9.40825298	6.39899392	10.67165881	-0.00338218	0.00340983	-0.00721914
H	0.25885284	-0.55883793	9.34977814	0.00255088	0.00261912	0.00521552
O	1.12329414	-0.03908289	9.34162389	0.00630649	0.00389294	0.01411146
H	1.09098849	0.45221058	8.48459301	0.00867914	0.00148351	0.00220032
H	8.90502799	7.35936967	6.33642902	-0.00522055	-0.00933829	-0.01501421
O	9.74942657	7.52646422	6.93998583	0.00196510	-0.01524606	-0.00710783
H	10.32391796	8.20447872	6.47857584	-0.00658848	-0.01317551	-0.01277013
H	6.28388499	-0.95551068	8.52242971	-0.00248314	0.00057696	-0.01328058
O	6.17212443	-0.42080313	7.67290018	0.00102004	-0.00847301	0.00996818
H	6.71354475	-0.91457217	6.92642259	-0.00959205	-0.01017182	-0.01414179
H	8.55576400	2.93560922	6.28619408	0.01340508	0.00216935	-0.00652514
O	8.32695649	3.92074257	6.20298212	-0.00221125	-0.00008559	-0.00702720
H	8.25834226	4.19244339	7.19208343	0.00008298	0.00342438	-0.01229292
H	8.10823891	0.81329665	4.99500211	-0.01062972	-0.00247481	-0.01859502
O	8.01169181	0.73860072	3.98696902	-0.00025649	-0.00681240	-0.01563505
H	7.06027639	1.02201714	3.84639243	-0.00143860	0.03182628	-0.00746333
H	2.22704031	5.79617637	5.19336045	-0.00435086	0.00202713	-0.00416893
O	2.79724662	6.07126823	4.34619032	0.00926363	0.00355206	-0.01464496
H	2.50728663	5.40522247	3.66814300	-0.01290590	-0.01723527	-0.01033153
H	7.12502261	4.84194660	8.60866722	-0.00828883	0.00577402	-0.00869574
O	8.04595701	4.48384150	8.70611430	-0.00862142	0.00918806	0.00207049
H	8.56594858	5.27226932	9.12855553	-0.00577508	0.00339632	-0.00410742
H	5.86647267	7.71672559	10.41527357	0.00308804	0.00250045	0.01118081
O	6.45638219	6.96789748	10.10494877	-0.00340095	0.00283643	0.01923382
H	5.84270791	6.14827584	10.26611913	0.00218556	0.00101455	0.00996099
H	3.67820189	7.69269951	9.66965258	0.00179436	0.00033364	0.00604145
O	4.20321682	8.45908418	10.09056377	-0.00043703	-0.00419710	0.01151648
H	3.66232003	8.72831601	10.87675877	0.00186993	-0.00725623	-0.00941109
H	2.60950489	8.94650673	6.32715920	0.01049533	-0.00353025	0.00185981
O	1.78114559	9.47316686	6.05124125	0.00081357	-0.00333712	0.00336426
H	1.51338514	9.06176042	5.15964693	-0.00147702	-0.00223148	-0.00012367
O	9.67957165	2.74572804	3.73390446	0.00585637	0.00429927	-0.01294506
O	9.84584900	5.58330740	4.07015469	-0.00515440	-0.00209224	-0.02092507
H	9.03603953	1.94661294	3.70307888	-0.00290639	-0.00799117	-0.00485411
H	10.26773777	2.62965609	2.94856671	0.01092240	-0.00795276	0.01054116
H	10.12692884	5.28710365	4.99987782	-0.00219872	0.00543332	0.00014730

H	9.80305364	6.57831866	4.10759744	-0.02022727	0.00340452	-0.00135361
O	2.84560805	6.55568868	8.83140225	0.00144052	-0.00276058	0.00018764
H	3.22966209	7.00845455	7.98542997	0.00227711	-0.00167380	-0.00582953
H	2.04384454	7.18153682	9.02227770	0.00562097	-0.00798659	-0.00004624
O	4.71922399	5.04338919	10.34690549	0.00148674	-0.00799443	0.01373475
H	3.95058521	5.54457566	9.94404657	0.00489602	-0.00122433	0.00989580
H	4.45840693	4.91449168	11.29737497	0.01271438	0.00263191	-0.00322582
H	7.59562174	6.04473299	5.99350801	0.01082512	0.01242596	0.01153792

*CO_2;*OH (oxidated)

116

Lattice="9.84 0.0 0.0 0.0 8.52169 0.0 0.0 0.0 11.75" Properties=species:S:1:pos:R:3:forces:R:3

C	1.17670276	0.81960951	0.78785998	-0.00043230	-0.00307365	-0.00278003
C	1.17902110	2.24742788	0.70789018	0.00294557	-0.00342338	0.00043804
C	2.41030297	2.95676781	0.83806356	0.00866609	0.00060458	0.00046512
C	2.42299739	4.38487723	0.90917917	0.01541771	0.00429586	0.00125355
C	1.19271228	5.09033892	0.79553484	-0.00722085	0.00572622	-0.00164468
C	1.18948417	6.50955349	0.90824845	0.00365670	0.00086742	0.00709142
C	2.42500300	7.20554183	1.06930074	0.00334039	-0.00703950	0.00436628
C	2.40236929	8.63006527	0.97950319	0.00304775	-0.00366351	0.00239161
C	3.62954558	0.81802300	1.06762550	0.00329880	0.00301667	0.01521750
C	3.62462744	2.23780537	1.00865619	0.00518192	0.00531132	-0.01157839
C	4.82266431	2.93082276	1.35497434	0.01420127	0.00083168	-0.03364681
C	4.68489759	4.26579314	1.83189166	0.01300346	0.00323439	0.00921962
C	3.58962898	5.06354449	1.38307408	0.00803504	0.01628983	-0.00545316
C	3.63658385	6.50594732	1.39590977	0.01200111	-0.00683941	0.00904831
C	4.86628394	7.24823497	1.54907771	-0.01565419	-0.00675470	-0.00220472
C	4.85875337	8.63947630	1.24942879	0.00973891	-0.01053536	0.01722344
C	6.09219014	0.83543889	1.14462072	-0.00409496	0.00493462	0.00367615
C	6.09447550	2.24814168	1.24097583	0.00902555	0.00685349	-0.00154586
C	7.34770485	2.93778856	1.16915809	-0.01784870	0.00613976	-0.00000105
C	7.50508081	4.28413070	1.63243668	-0.01007893	0.00373318	0.01313582
C	6.15491704	6.70102608	1.95059918	0.00132104	-0.00831979	0.00984043
C	7.34116578	7.25357070	1.34068538	0.01042157	-0.01185538	-0.04356110
C	7.32303726	8.64512898	1.02475385	-0.00555884	-0.00349693	-0.01058732
C	8.54081619	0.81875596	0.78968801	-0.00070685	-0.00219758	-0.00242414
C	8.53940228	2.24479941	0.78430179	-0.00885863	0.00275236	0.00081214
C	9.78016674	2.95359986	0.68949085	-0.00583047	0.00521213	-0.01316249
C	9.79674459	4.38194129	0.77244403	-0.00910454	0.00469318	-0.01244223
C	8.59592049	5.07739930	1.14959447	-0.00878065	0.00476826	-0.01414754
C	8.56274916	6.51796098	1.15321475	-0.00710945	0.00092657	-0.01096321
C	9.78603962	7.21788862	0.92104795	-0.00225816	-0.00628884	0.01512936
C	9.78050914	8.63871758	0.80108723	-0.00417637	-0.00285293	0.00217433
Cu	6.65267398	4.22316237	5.03544756	-0.00847624	-0.04318112	-0.02578828
Cu	4.72741327	5.59931631	4.71028765	-0.01591277	-0.00940999	0.00644056

Cu	6.87020053	6.98603249	3.86961717	-0.00623532	0.04587862	0.06879630
Cu	4.53934549	3.35619368	3.69666538	-0.00157228	0.00658065	0.04289065
Cu	6.14929042	4.98635914	2.76896905	0.00712335	-0.01008441	0.04969906
Cu	8.46953406	4.48039381	3.38188931	-0.01421556	-0.02032948	0.06204916
C	6.01913632	5.76715860	6.20510845	-0.00469876	0.00055369	0.00692530
O	5.63879567	5.55099409	7.41840506	-0.01070691	0.00389555	0.02550214
O	6.91088384	6.74058438	5.94908157	0.00998857	0.01176196	0.00315173
H	3.70512782	3.36194335	8.08570437	0.00269443	0.00570294	-0.01200165
O	3.44608812	4.08862069	7.43190574	0.00532539	0.00597433	0.00595688
H	4.25737674	4.71491889	7.46567804	0.01021323	0.00990738	-0.01665064
H	1.17331834	8.31110557	3.22603077	-0.00558760	-0.00058901	0.00709957
O	1.15873443	7.92232148	4.13271871	-0.00459426	0.00839563	-0.02537818
H	2.10440933	7.42021144	4.29588475	0.01330089	-0.01041924	-0.01010174
H	2.51368538	2.17668320	5.40063960	0.01561282	0.01047073	-0.01266757
O	3.06139039	2.99145943	5.04924571	0.01032447	-0.00517286	-0.01292848
H	3.23827194	3.46946794	5.92860027	0.00310747	0.00712302	-0.00233874
H	7.47654647	3.25999649	9.32610221	-0.00841118	-0.00313714	-0.00026571
O	7.08492756	2.42328274	9.75504155	-0.00060960	-0.00999652	0.01089472
H	7.12410926	2.61242074	10.72591387	-0.00668639	0.00422173	-0.00411552
H	3.68304796	-0.98552911	6.12124880	0.00718464	0.00884606	0.00702298
O	3.75274878	-0.39499601	6.97066024	0.00837511	0.00409106	-0.00191838
H	4.72655781	-0.28286982	7.28127532	0.00138642	-0.00570630	-0.01266214
H	0.86408312	5.95943637	7.05000323	0.00670774	0.00286991	0.00286211
O	1.33737124	5.29945852	6.47276288	0.00397488	0.00338229	-0.00586020
H	1.99560253	4.79895611	7.05313892	0.00131742	0.01064329	-0.01105364
H	5.17443513	2.02747593	7.52873544	0.00639890	0.01127292	-0.01947670
O	5.59800827	2.15005060	6.60007701	-0.00480193	0.00379063	0.00528422
H	5.26500969	3.05068323	6.34404297	0.00707189	-0.00657923	0.00098477
H	8.87243173	8.93651594	6.89210871	-0.00773555	-0.00083771	-0.00566891
O	8.26327459	9.70153408	6.63317239	-0.00797843	-0.00252540	-0.00006165
H	7.43842905	9.53547872	7.18881903	0.00039233	-0.00125606	-0.00194832
H	4.54174748	9.00168055	4.15367826	0.00408154	-0.00910633	-0.00359130
O	5.21883371	9.71029319	4.27830176	-0.01119048	-0.00171858	-0.01130940
H	5.23495989	9.93116688	5.29973726	0.01134443	-0.01433960	0.00595838
H	5.64796434	2.37774943	9.35626526	-0.00343112	-0.00502182	-0.00132617
O	4.64135057	2.33575102	9.03924511	-0.00090922	-0.00793210	0.00446160
H	4.33481500	1.42698682	9.40851289	0.00320193	-0.00167030	0.00245889
H	7.86819451	6.95031619	10.05856628	0.00441311	0.00369969	0.00735590
O	8.83599649	6.74454861	9.77062435	0.00080222	0.00548189	0.00751127
H	9.27428748	6.48712497	10.61942916	-0.00173498	0.00001927	-0.00160566
H	0.05878757	-0.45202473	9.27722605	-0.00210292	0.00307713	-0.00021246
O	0.91640722	0.07256915	9.22638897	0.00217470	0.00180776	0.01071042
H	0.91180470	0.48223402	8.32330018	0.00002180	-0.00719315	0.00470310
H	8.59528237	6.93063993	6.61729367	-0.01103786	-0.01385002	-0.01430583
O	9.46710545	7.37488474	6.86494267	-0.00017401	-0.00819645	-0.00188539

H	9.86678416	7.49450963	5.96381420	-0.01326018	0.00435440	0.00777793
H	6.26166344	-0.72312866	8.68664835	-0.00166316	0.00293656	-0.01016445
O	6.22838137	-0.14546674	7.85338883	-0.00242771	-0.00717325	0.00669721
H	6.56776278	-0.77918207	7.13465452	-0.00407999	0.00146146	-0.01057739
H	8.40315345	2.72861791	6.26641968	0.00608902	0.00366025	-0.00758493
O	8.29270655	3.72229510	6.07125586	-0.01077601	0.00458207	-0.00556757
H	8.24810261	4.13802886	7.00336793	0.00824343	0.00380416	-0.00455892
H	7.93509343	0.67847719	5.10588994	-0.00737229	0.00330004	-0.01011094
O	7.77140569	0.50716598	4.10426586	0.00232860	-0.01288172	-0.01590378
H	6.82866844	0.87800432	4.00431185	-0.00050263	0.04207656	-0.00498585
H	2.52912390	6.12174587	5.47848090	-0.01114333	0.00469778	-0.00112796
O	3.15963730	6.69196343	4.92395817	0.01124903	-0.01540414	-0.01105425
H	1.59072267	4.79906893	3.35528446	-0.09128982	0.15930832	-0.14673885
H	6.99758507	5.04372559	8.26559065	-0.01142185	0.00218589	-0.00807017
O	7.89002266	4.63078325	8.50800357	-0.01109213	0.00945326	-0.00245623
H	8.38921922	5.38312945	8.99025857	-0.00738266	0.00477528	-0.00819396
H	5.59386600	7.66567305	10.43195782	0.00437882	0.00016118	0.00688056
O	6.29160438	6.97664592	10.17549972	-0.00304976	0.00039002	0.01696145
H	5.73530404	6.11265505	10.15517339	0.00148048	0.00090668	0.00812109
H	3.59845192	7.79221643	9.67531312	0.00229810	0.00159745	0.00454853
O	4.18238848	8.54287907	10.11643588	0.00211805	-0.00049997	0.00922441
H	3.68237974	8.78011951	10.93556733	-0.00105361	-0.00581172	-0.01180139
H	2.54967558	9.11587018	6.56375837	0.00712664	-0.00304380	-0.00044964
O	1.73883423	9.64957286	6.23812079	-0.00187439	-0.00676522	-0.00621198
H	1.40003119	9.10568318	5.46218230	-0.00321114	0.00201922	0.00129874
O	9.53449844	2.58323895	3.73937561	0.00502522	0.00250410	-0.01238508
O	9.47520123	5.79089172	4.50931566	0.00197132	-0.00819455	-0.02287920
H	8.88605728	1.80545591	3.70298810	-0.00596085	-0.00149451	-0.00808691
H	10.13023358	2.44850865	2.96316189	0.01040917	-0.01070760	0.01112700
H	10.07289163	5.31378741	5.17260649	-0.00464603	0.00461920	0.00618266
H	10.04947358	6.54183586	4.13392551	-0.01807490	0.00142210	-0.00749196
O	2.75653310	6.76220209	8.95547930	0.00266237	-0.01268416	-0.00328976
H	3.14762225	7.14663346	8.08412917	0.00673551	-0.00662020	-0.00535864
H	1.93750406	7.38053160	9.07728446	0.00595213	-0.00731169	0.00104281
O	4.54621998	5.02062901	10.21509306	0.00488173	-0.00435713	0.01204345
H	3.78427839	5.46831050	9.74148658	0.00270495	-0.00244226	0.00586082
H	4.53555312	4.07497060	9.90287905	0.00504892	0.00873148	0.01090869
H	1.90728992	4.27626613	3.84581180	0.09625364	-0.14860142	0.13743697

*HCOOH

117

Lattice="9.84 0.0 0.0 0.0 8.52169 0.0 0.0 0.0 11.75" Properties=species:S:1:pos:R:3:forces:R:3

C	1.21280811	0.77019385	0.76978027	-0.00067270	-0.00629204	-0.00171357
C	1.21541312	2.19802115	0.69169605	0.00166734	-0.00467159	-0.00141720
C	2.44206021	2.90757681	0.84198391	0.00676137	0.00197817	0.00260109

C	2.44460089	4.33479682	0.94510085	0.01277140	0.00565108	0.01222151
C	1.21947073	5.04015371	0.81498862	-0.00429648	0.00519272	0.00563769
C	1.21956253	6.45950079	0.92321472	0.00276536	0.00174082	0.00613436
C	2.45590108	7.15814752	1.07254709	0.00214825	-0.00420904	0.00612382
C	2.43772950	8.58227613	0.95926364	-0.00136289	-0.00504110	0.00834089
C	3.66676248	0.77118480	1.02441180	0.00256613	0.00008367	0.01166428
C	3.66385463	2.19503436	0.98225593	0.00550316	0.00501365	-0.01379642
C	4.86118546	2.88930644	1.32356763	0.01555697	-0.00142060	-0.04677671
C	4.72262279	4.21810804	1.83747267	0.01354572	0.00270760	0.00919773
C	3.61614858	5.01395889	1.40356689	0.00468217	0.01653202	-0.00653861
C	3.66798956	6.45757256	1.39810210	0.01194559	-0.00571864	0.00639746
C	4.90302515	7.20438071	1.52922886	-0.01491375	-0.00667360	-0.00388170
C	4.89439333	8.59012746	1.20402207	0.01073464	-0.01229137	0.01223434
C	6.12611541	0.78986598	1.10586053	-0.00188879	0.00040364	0.00335699
C	6.12771754	2.20254072	1.20483289	0.00897056	0.00681293	-0.00166569
C	7.38498837	2.89235183	1.12267472	-0.01799029	0.00487611	-0.00314075
C	7.52811440	4.23445916	1.59107372	-0.00988205	0.00412224	0.01417162
C	6.17880685	6.65930387	1.95529999	0.00190155	-0.00803902	0.00740706
C	7.36829325	7.20772750	1.34211029	0.01051347	-0.01103795	-0.02716296
C	7.35552064	8.59352283	1.00160370	-0.00458131	-0.00463874	-0.00487623
C	8.57421800	0.76863165	0.76436747	0.00008054	-0.00316647	-0.00156727
C	8.57409376	2.19506567	0.73836588	-0.00939838	0.00030543	-0.00362196
C	9.81606685	2.90435677	0.65260902	-0.00579776	0.00282824	-0.01706486
C	9.82449881	4.33322776	0.74234890	-0.00966406	0.00615895	-0.01639231
C	8.62172883	5.02166434	1.11656969	-0.01007449	0.01037525	-0.02356496
C	8.58995565	6.46675481	1.15867623	-0.00702342	0.00265646	-0.00962059
C	9.81501617	7.16806924	0.92687477	-0.00277555	-0.00637100	0.01159722
C	9.81455328	8.59067322	0.79621399	-0.00416863	-0.00007641	0.00107694
Cu	6.81068946	4.41482681	5.09153308	0.00934102	-0.01061227	-0.00044819
Cu	4.79376379	5.57914092	4.65586443	0.02095389	-0.00332322	0.03130706
Cu	6.88262842	6.88936821	3.94394374	-0.00564493	0.02965302	0.04580168
Cu	4.63695978	3.35117737	3.70483887	-0.00179546	0.02247195	0.04394869
Cu	6.19774248	4.94279392	2.75949596	0.00450792	-0.00686897	0.06024846
Cu	8.46230959	4.33034284	3.35348241	-0.04498412	-0.03493989	0.07045735
C	6.06424033	5.82255015	6.35229792	-0.00787485	-0.00587935	-0.00342015
O	6.01616816	5.23151417	7.70970601	-0.00852015	-0.00264661	0.02135784
O	7.08630446	6.78446561	6.17653036	0.02294183	0.00017668	0.01581447
H	3.67446090	3.34085710	8.06931455	0.00435323	0.00641855	-0.01517487
O	3.47769925	4.10268188	7.43635652	0.01659969	0.00828457	-0.00194908
H	4.34776353	4.60168678	7.52056356	0.00543288	0.00313945	-0.01877751
H	0.54813778	8.23169817	3.24347644	-0.00739046	-0.00379737	0.00427515
O	0.80026620	7.91923561	4.14905403	-0.00864439	0.00428283	-0.01838713
H	1.69626650	7.45031899	4.06899736	0.00583408	-0.00073221	-0.01013911
H	2.40721047	2.34399373	5.22725758	0.01372164	0.01621116	-0.01217137
O	2.96248421	3.14860459	4.90531716	0.00182182	0.00295299	-0.01702592

H	3.22831584	3.53200039	5.79977118	-0.00058959	0.00576104	0.00291177
H	7.68704834	3.06793510	9.36372981	-0.00745958	-0.00205033	-0.00035420
O	7.12488203	2.31110028	9.74731911	-0.00145368	-0.00930552	0.00978583
H	7.16862761	2.47332831	10.72585019	-0.00604411	0.00391563	-0.00467750
H	3.84245233	-0.83487096	5.94203513	0.01111623	0.01042381	0.00536348
O	3.66829765	-0.37017846	6.79698961	-0.00420426	0.00039012	-0.00849216
H	4.64968102	-0.23060032	7.23178243	0.00352979	0.00033901	-0.00759624
H	0.84061484	6.13052605	6.88413469	0.00365410	0.00395281	0.00100727
O	1.45660323	5.43291798	6.47873629	0.00110511	0.00412736	0.00621017
H	2.06011887	5.00987961	7.16459600	-0.00042269	0.00871260	-0.01224135
H	5.20990959	2.15840007	7.48485995	0.00516989	0.01171123	-0.01909041
O	5.65343724	2.13131782	6.56114666	-0.00271841	-0.00061449	0.00726355
H	5.66130170	3.08043830	6.22264974	-0.00078143	0.00359404	0.00303531
H	8.68057015	8.95675000	6.95430521	-0.00950813	0.00450179	-0.00304465
O	8.25053844	9.73841923	6.49844337	-0.00808285	-0.00345672	0.00249072
H	7.33122996	9.86258899	6.88713167	0.00291157	0.00807439	-0.00948829
H	4.51671878	9.05984956	4.00451638	0.00412979	-0.01272271	-0.00279166
O	5.22010495	9.71382010	4.23023374	-0.00567409	0.00134704	-0.01045583
H	5.19539304	9.90204042	5.24580745	0.01020368	-0.02027333	0.00915954
H	5.69916145	2.39072739	9.33843066	-0.00364345	-0.00174485	-0.00156164
O	4.68464673	2.37177641	9.02865297	0.00178714	-0.00632178	0.00559140
H	4.37035066	1.45767209	9.36574107	0.00384084	-0.00175489	0.00305334
H	7.80739433	6.88282442	9.99594021	-0.00017062	0.00365346	0.00411083
O	8.80353644	6.77143421	9.80127246	-0.00577254	0.00416807	0.00534925
H	9.17429400	6.65695494	10.71694070	-0.00320341	-0.00117313	-0.00131963
H	0.00636576	-0.46695108	9.30334558	-0.00194576	0.00152389	-0.00013033
O	0.86748006	0.05528084	9.21428006	0.00118624	0.00322177	0.01057583
H	0.81158494	0.47996894	8.32099304	0.00102685	-0.00512435	-0.00169381
H	8.57756972	6.90454999	6.59904819	-0.00495376	-0.01561877	-0.01466754
O	9.50507128	7.33417772	6.79587965	0.00628772	-0.00866823	-0.00241175
H	9.79514805	7.62976977	5.89090367	-0.01274417	-0.00781939	-0.00235857
H	6.10761571	-0.62202065	8.62135888	0.00259742	0.00492019	-0.00984062
O	5.99979907	-0.14090157	7.75836799	0.00093851	-0.00777709	0.01411628
H	6.55106520	-0.73062647	7.06285261	-0.01081033	0.01135537	0.00017278
H	8.71922477	2.85027792	6.13184669	0.00599255	0.00371636	-0.01518325
O	8.62639616	3.85010638	6.06687755	-0.01346847	0.00270666	-0.00112538
H	8.66133842	4.13566009	7.04699257	-0.00158977	0.00553184	-0.01563680
H	7.94553072	0.62132630	5.05224389	-0.00559894	0.00764651	-0.00865210
O	7.77571429	0.37974355	4.06466583	0.00031490	-0.01237923	-0.01625159
H	6.86706532	0.80139498	3.91842707	0.00155412	0.04035601	-0.00695144
H	2.28804320	5.90890652	5.41861210	0.00212082	0.00130820	-0.00001096
O	2.83595897	6.22062897	4.53126511	0.01398178	-0.00111202	-0.01664882
H	2.48482858	5.55951861	3.87005592	-0.00153618	-0.01318291	-0.01669194
H	7.42295790	4.74000901	8.28782197	-0.01076739	0.00177912	-0.01192810
O	8.33230527	4.42020793	8.61729592	-0.00540310	0.00861539	0.00150511

H	8.68527750	5.23892216	9.10746925	-0.00487127	0.00270617	-0.00475316
H	5.50730127	7.53141027	10.32543051	0.00350155	-0.00061897	0.00728161
O	6.16283769	6.78608958	10.09237272	0.00164783	0.00177044	0.01875861
H	5.58236525	5.94737991	10.28228962	0.00677132	0.00310646	0.01050346
H	3.54913815	7.81237898	9.59660506	0.00308683	-0.00071850	0.00507604
O	4.17684394	8.53163423	10.01996456	0.00049669	-0.00103559	0.01297404
H	3.75528065	8.75294898	10.89149008	-0.00072413	-0.00599439	-0.01085219
H	2.41254469	9.17796184	6.38698006	0.00406961	-0.00624815	0.00128941
O	1.63043797	9.75520302	6.10580185	-0.00780464	-0.00708187	-0.00315054
H	1.20918429	9.25620407	5.35067385	-0.00358203	0.00221179	0.00037489
O	9.63148204	2.37375224	3.70747420	0.00006498	-0.00097801	-0.01134941
O	10.15274518	5.18947252	4.06391146	-0.01586287	-0.00279528	-0.02011413
H	8.93701476	1.64784273	3.62013502	-0.00616088	0.00178506	-0.00699189
H	10.13957100	2.33981378	2.85786571	0.01046484	-0.00977306	0.01374776
H	10.22976467	4.85482005	5.00854974	0.00911503	0.00575531	-0.00130580
H	10.03564849	6.17821051	4.15089732	-0.01351121	0.00515263	-0.00050689
O	2.68299311	6.77681954	8.87543797	0.01100321	-0.01318483	-0.00566073
H	3.01676601	7.09655364	7.97510833	0.00704275	-0.00753081	-0.00288097
H	1.86499160	7.41361966	9.01647449	0.00526338	-0.00718126	0.00165300
O	4.38093583	4.93583990	10.25025218	0.00144470	-0.00155580	0.01082526
H	3.63536612	5.38228600	9.75541236	0.00309425	-0.00054746	0.00601605
H	4.41151132	3.99023933	9.92812729	0.00510037	0.00599168	0.01226885
H	5.08422759	6.40709268	6.36204155	0.00133707	-0.01413916	-0.02284483
H	5.86676307	5.96352079	8.38779451	-0.00416701	-0.00793307	-0.01184293

*CO

117

Lattice="9.84 0.0 0.0 0.0 8.52169 0.0 0.0 0.0 11.75" Properties=species:S:1:pos:R:3:forces:R:3

C	1.07937749	0.80621517	0.77313884	-0.00113553	-0.00335822	-0.00526229
C	1.08623455	2.23171942	0.70011904	0.00249941	-0.00577915	-0.00247834
C	2.31182527	2.94032709	0.87389078	0.00478895	0.00240448	0.00429563
C	2.31241533	4.36780587	0.94322608	0.01298746	0.00607924	0.01221060
C	1.09333056	5.07577912	0.77710109	-0.00430274	0.00638614	0.00453811
C	1.08722093	6.49692446	0.87739618	0.00557280	0.00048717	0.00609229
C	2.31883129	7.19582534	1.03301742	0.00475558	-0.00745660	0.00011533
C	2.30684215	8.62203376	0.96143989	0.00272510	-0.00362158	0.00016473
C	3.53272835	0.81498753	1.07950394	-0.00025115	-0.00399967	0.01468559
C	3.53500223	2.23792183	1.05224282	0.00485354	0.00392860	-0.00600299
C	4.73738403	2.93603544	1.38429974	0.01463631	-0.00454204	-0.04168316
C	4.58244914	4.28006889	1.86050885	0.01512487	0.00712385	0.00952689
C	3.48633788	5.05354350	1.38642454	0.00486291	0.02103577	-0.00537550
C	3.52382455	6.49480358	1.33776335	0.01547108	-0.00591355	0.00911097
C	4.75824447	7.22592636	1.45107064	-0.00445426	-0.01117111	-0.00319283
C	4.76127162	8.63266343	1.23061768	0.00993269	-0.01108890	0.01736665
C	5.99608977	0.82942619	1.14152205	0.00482552	-0.00837426	-0.00199109

C	6.00039334	2.24631323	1.24987615	0.00753367	0.00675751	-0.00097369
C	7.26138773	2.93137781	1.13705614	-0.01477315	0.00591107	-0.00222999
C	7.42324164	4.27467398	1.58057517	-0.01096173	0.00238699	0.01152109
C	6.01299878	6.62239990	1.75717542	-0.00041905	-0.01250995	0.00440190
C	7.23706222	7.22857010	1.31249154	0.00229444	-0.00848215	-0.00534579
C	7.22343502	8.63629486	1.01795968	-0.00607497	-0.00102222	-0.01461595
C	8.44229013	0.80486667	0.77711451	-0.00688596	-0.01307093	0.00216393
C	8.44418828	2.23106953	0.73877585	-0.00989434	0.00210186	0.00092029
C	9.68941109	2.93770706	0.63043081	-0.00858754	0.00349580	-0.01701666
C	9.70198648	4.36517417	0.69366917	-0.01118116	0.00603217	-0.01972213
C	8.50163665	5.05150221	1.08001585	-0.01361291	0.01147055	-0.02117479
C	8.47023271	6.49683847	1.12311571	-0.00747135	0.00132370	-0.01651119
C	9.68329496	7.20160277	0.90125802	-0.01641801	-0.00751237	0.01407466
C	9.68190920	8.62459155	0.78656000	-0.00709653	-0.00038937	0.00135790
Cu	6.67935216	4.46019479	5.07562469	-0.01024866	-0.04567733	-0.00173321
Cu	4.70377321	5.91585741	4.82370261	0.00496065	0.01536055	0.03742384
Cu	7.01790662	7.08949392	3.57314117	0.02058606	0.04143081	0.03779926
Cu	4.61033242	3.57303725	3.90601815	0.03716418	-0.00249824	0.03387507
Cu	6.07099039	5.01829176	2.75841442	0.00820307	-0.00299187	0.03371082
Cu	8.36594704	4.51871988	3.33318098	-0.02087113	-0.01281087	0.06098549
C	6.36854684	6.56056967	5.32355530	0.00068893	0.02614318	0.00942668
O	5.56721032	5.05689080	7.97950889	-0.00086773	0.00880572	0.00065779
O	7.14722786	6.75419397	6.35162922	0.01527671	0.00404383	0.00273059
H	3.50764377	3.20425067	8.07975366	0.00325514	0.00366412	-0.01296033
O	3.22643364	3.91984558	7.42773516	0.01593370	0.00512423	-0.00092814
H	4.07156538	4.48193461	7.42138896	0.01357386	0.00982790	-0.02486348
H	0.94096787	8.34068655	3.02079283	-0.00973127	-0.00049572	0.01835894
O	1.10026272	7.97224781	3.92650894	-0.00448642	0.00647360	-0.02457582
H	1.88899708	7.33337197	3.85349542	0.01010888	-0.00292021	-0.00939277
H	2.49264234	2.21531208	5.14266960	0.00955832	0.01212583	-0.01042415
O	2.89460476	3.12303285	4.90182828	0.00579435	-0.00182880	-0.01795488
H	3.06147502	3.45155209	5.84887710	0.00167100	0.00547732	0.00146755
H	7.57309338	3.24422488	9.36615741	-0.00853742	-0.00155274	0.00078064
O	7.07135682	2.45074632	9.76099869	-0.00287508	-0.00746525	0.00974844
H	7.15552133	2.58806410	10.74072880	-0.00668446	0.00514357	-0.00386237
H	4.24024300	-1.17123588	6.15058631	0.00329450	-0.00092184	-0.00209824
O	3.80269689	-0.60587255	6.89328110	-0.00048504	0.00006694	-0.00150608
H	4.66602990	-0.31648038	7.41113427	0.00180577	-0.00052972	-0.00758835
H	0.83897182	6.14544072	6.89152473	0.00493401	0.00358042	0.00167421
O	1.37202201	5.40891278	6.43985726	0.00180519	0.00501348	0.00396061
H	1.91737189	4.86617524	7.09531110	0.00049212	0.01353242	-0.00760469
H	5.19170489	2.11346365	7.56513812	0.00472612	0.00926793	-0.01762695
O	5.64934096	2.08362393	6.65455433	-0.00491284	-0.00427912	0.00854033
H	5.63811129	3.03138090	6.31352122	-0.00128637	0.00152403	0.01480150
H	8.64670855	9.00159838	7.00231539	-0.00944171	0.00322611	-0.00046624

O	8.21419075	9.73676741	6.48186916	-0.00712656	-0.00796504	0.00183409
H	7.28355250	9.88286156	6.84847057	0.00023883	0.00583031	-0.01009156
H	4.78431407	8.86561125	4.06955360	0.00001471	-0.01329438	-0.00239720
O	5.23195831	9.73441244	4.23654968	-0.00838085	0.00049291	-0.01111230
H	5.23283039	9.85937257	5.24917701	0.00738535	-0.01606890	0.00124386
H	5.63415988	2.42428130	9.40911421	-0.00368586	-0.00383761	0.00018495
O	4.60924466	2.35660335	9.13025611	0.00132796	-0.00727613	0.00725824
H	4.34790466	1.42433666	9.45724954	0.00434995	-0.00173620	0.00432997
H	7.87757036	7.05127680	10.08524273	0.00170549	0.00318505	0.00633475
O	8.82783391	6.84039556	9.78749060	-0.00362520	0.00445264	0.00610317
H	9.26931462	6.64470434	10.65591259	-0.00315839	-0.00116842	-0.00304117
H	0.08539770	-0.40969658	9.26483861	-0.00090100	0.00168860	0.00218293
O	0.96210546	0.08327934	9.19932238	0.00309365	0.00224459	0.01182188
H	0.96961840	0.45193331	8.27945805	0.00338638	-0.00353520	0.00079346
H	8.69884741	6.99950733	6.52243972	-0.00744504	-0.01181249	-0.01357710
O	9.58072321	7.40054759	6.87498242	0.00315208	-0.01057368	-0.00624880
H	9.98374165	7.82167491	6.07574229	-0.01408926	-0.01034377	-0.00147015
H	6.17086327	-0.70051178	8.86600365	0.00063081	0.00463003	-0.00651176
O	6.07152600	-0.16753890	8.02460218	0.00063503	-0.00455126	0.01142192
H	6.56458987	-0.74469201	7.34114233	-0.00650103	-0.00290575	-0.00077980
H	8.52754609	2.88671977	6.22093873	0.00342765	0.00483737	-0.01255472
O	8.34997605	3.87217538	6.13692563	-0.01382597	0.00303701	-0.00086986
H	8.37878406	4.21237119	7.10057948	0.00124535	0.00473894	-0.01006470
H	7.99164751	0.66997899	4.99012262	-0.00349742	0.00391523	-0.00821324
O	7.80483463	0.54610837	3.98335208	0.00334734	-0.01384098	-0.01781122
H	6.85846572	0.93641642	3.93881059	-0.00115753	0.04196249	-0.00406675
H	2.20180704	5.76296558	5.29008615	-0.00662750	-0.00190909	-0.00439344
O	2.73332653	5.98923903	4.38522879	0.00569556	0.01103792	-0.01299422
H	2.54869791	5.19842442	3.80830108	-0.03582971	0.00309878	-0.01958345
H	7.13313422	4.87621110	8.37148537	-0.00833959	0.00394339	-0.01335213
O	8.08615194	4.60853030	8.61157058	-0.00822456	0.01227512	0.00132642
H	8.48893562	5.43036581	9.06332098	-0.00851970	0.00450906	-0.00585293
H	5.47044571	7.64171628	10.52180202	0.00550875	-0.00288042	0.00223385
O	6.24399533	6.98924646	10.35410259	-0.00612546	-0.00884877	0.01536185
H	5.85333906	6.11274901	10.61687778	0.00481813	0.00067995	0.01779591
H	3.56217455	7.76428200	9.69913317	0.00401515	-0.00126618	0.00617207
O	4.14966137	8.51099326	10.13663324	-0.00108908	-0.00174443	0.01035197
H	3.65669947	8.76025411	10.96011035	0.00036816	-0.00490236	-0.01096418
H	2.61797051	8.95603696	6.33955473	0.00475853	-0.00756551	-0.00011137
O	1.81329360	9.50798363	6.05317123	0.00303863	-0.00506244	-0.00041185
H	1.49384937	9.03431397	5.22687170	-0.00166756	-0.00238273	0.00085835
O	9.56805824	2.67900962	3.70474868	0.00175892	0.00099828	-0.01201698
O	9.54022696	5.73059523	4.40788389	0.01117554	0.00072295	-0.01728028
H	8.92382207	1.90296191	3.64943393	-0.00516984	-0.00569075	-0.00784100
H	10.12040511	2.57849139	2.88869804	0.01107683	-0.01005228	0.01136898

H	10.02639407	5.30267428	5.17234218	-0.00212115	0.00738828	-0.00195206
H	10.01917570	6.56597784	4.12896788	-0.02238971	-0.00425543	-0.00936480
O	2.71868588	6.71502813	9.00041906	0.00600438	-0.01325736	-0.00067531
H	3.10352725	6.97995862	8.09244225	0.00504699	-0.00781999	-0.00530630
H	1.91579244	7.37402268	9.07887243	0.00586020	-0.00773268	0.00268319
O	4.37712356	4.88493389	10.22326048	0.00074353	0.00042921	0.02373086
H	3.54043135	5.35898910	9.92716707	0.00384776	0.00074563	0.00999553
H	4.22391104	3.89845758	10.09830803	0.00474340	0.00679470	0.01529316
H	5.64104852	5.98412078	7.64188359	-0.01210674	-0.01931843	-0.00678184
H	5.13378863	5.09581880	8.92806466	0.00645530	0.00546715	-0.01431504

*CHO;*OH

117

Lattice="9.84 0.0 0.0 0.0 8.52169 0.0 0.0 0.0 11.75" Properties=species:S:1:pos:R:3:forces:R:3

C	1.06768540	0.80176796	0.77201380	-0.00050074	-0.00368193	-0.00355937
C	1.06781468	2.23058187	0.70318708	0.00256240	-0.00540703	-0.00133351
C	2.29860417	2.93855926	0.86620521	0.00865960	0.00296236	0.00411292
C	2.30092243	4.36464356	0.96399121	0.01013148	0.00688819	0.01457142
C	1.07689448	5.07170806	0.81953102	-0.00143270	0.00565727	0.00547220
C	1.07683347	6.49332137	0.91723791	0.00626733	0.00077664	0.00867177
C	2.31561020	7.19145835	1.05715886	0.00439986	-0.00671416	0.00152071
C	2.29743949	8.61524557	0.94857210	0.00248316	-0.00598913	0.00223933
C	3.52445948	0.80381597	1.03533255	0.00178226	-0.00094697	0.01015456
C	3.51999965	2.22718154	1.01983659	0.00438247	0.00385744	-0.01261062
C	4.71743243	2.91898118	1.37871673	0.01350198	-0.00165570	-0.04173969
C	4.58153475	4.25392406	1.88005730	0.01275525	0.00201518	0.01625544
C	3.47509735	5.04738545	1.43493418	0.00374325	0.01705872	0.00052692
C	3.52774392	6.49922342	1.41063986	0.01205479	-0.00148054	0.01046365
C	4.75848615	7.24422425	1.56583680	-0.01715819	-0.00980223	-0.00129366
C	4.75188480	8.62649077	1.22674145	0.01044967	-0.01462358	0.01080124
C	5.98546623	0.82400491	1.13753345	-0.00325749	0.00045803	0.00502836
C	5.99075216	2.23710425	1.24492302	0.00957212	0.00787284	-0.00100904
C	7.24795715	2.92181866	1.16626719	-0.01638921	0.00813654	-0.00012301
C	7.39996475	4.26356247	1.63762512	-0.00981684	0.00261308	0.01121445
C	6.03623987	6.70221962	2.01224664	0.00002407	-0.00776124	0.00836902
C	7.22748441	7.24125821	1.38049812	0.00981061	-0.01057159	-0.03733628
C	7.21319308	8.62654069	1.03222903	-0.00513940	-0.00581118	-0.00565013
C	8.43046706	0.79973094	0.78134801	0.00024506	-0.00275083	-0.00234498
C	8.42993111	2.22542876	0.76255764	-0.00598760	0.00388051	0.00339337
C	9.67046867	2.93584292	0.66650735	-0.00784939	0.00410942	-0.01669702
C	9.68009220	4.36663341	0.75052836	-0.00940252	0.00448264	-0.01642388
C	8.47939201	5.05645145	1.13309228	-0.01200383	0.00599539	-0.01717398
C	8.44764376	6.50029392	1.17523510	-0.00778936	0.00228684	-0.01259925
C	9.67071782	7.19980088	0.92827944	-0.00381744	-0.00740662	0.01350103
C	9.66984206	8.62076337	0.79753229	-0.00526863	-0.00364859	0.00042008

Cu	6.64860157	4.25793747	5.07177733	0.05019145	-0.09002681	-0.10377419
Cu	4.73612590	5.61422525	4.80673449	0.03409073	0.02336778	-0.01890092
Cu	6.81976512	6.89545938	3.94258015	-0.01415325	0.05590733	0.06833309
Cu	4.57871283	3.32947033	3.77357318	-0.00556390	0.01096013	0.03408417
Cu	6.05503071	4.97129226	2.79901613	0.00096008	-0.00640887	0.05653839
Cu	8.37850186	4.46976890	3.36744362	-0.04291712	-0.01919673	0.06532519
C	5.17212579	5.08466018	6.62132432	-0.05143424	0.04910258	0.09339568
O	6.03023717	5.48202557	7.47586336	-0.01492081	-0.00891017	0.02389286
O	7.62630694	7.05877092	5.71271375	0.00084992	0.00342540	-0.01043685
H	3.13324027	3.21883329	8.38789780	0.00328776	0.00247171	-0.00573358
O	2.43308394	3.87986947	8.06184536	0.00263059	0.00054092	0.00555840
H	2.65739882	4.73935573	8.52415913	0.00524115	-0.00000826	-0.00284638
H	0.88812755	8.51770932	3.02216489	-0.00688623	-0.00195186	0.01538166
O	1.01256057	8.14835058	3.93127551	-0.00811950	0.00382689	-0.01718527
H	1.78546408	7.49972213	3.87583081	0.00791296	-0.00100434	-0.00900928
H	2.50764173	2.23233971	5.39692624	0.01399015	0.01400592	-0.00818085
O	2.94727630	3.11097736	5.06607948	0.00329894	0.00473274	-0.01118279
H	3.05082329	3.58922995	5.92977801	0.00949719	0.00299215	-0.00924592
H	7.38346934	3.05259977	9.43515776	-0.00538077	-0.00105799	-0.00037181
O	6.77898228	2.32262333	9.79584249	-0.00026544	-0.01035907	0.01136077
H	6.70270120	2.56674356	10.75260173	-0.00954824	0.00482231	-0.00333269
H	4.06272056	-1.34791577	6.17017483	0.00766958	0.01197757	0.00296284
O	3.86162070	-0.62392358	6.83353298	-0.00804595	0.00454570	-0.00741625
H	4.88410412	-0.45226266	7.22730830	0.00587140	-0.00564731	-0.01054450
H	0.79538255	6.25018458	6.72797108	0.00197178	0.00145018	0.00128060
O	1.31529227	5.46432792	6.28168639	0.00503624	0.00186739	0.00473081
H	1.60422684	4.76207807	6.95182975	-0.00117737	0.00859782	-0.01008364
H	5.06113282	1.95364306	7.42596853	0.00248982	0.00768079	-0.01761208
O	5.61814241	1.92541887	6.51869497	-0.00334047	-0.00099982	0.00518590
H	5.59388696	2.87132649	6.18999410	0.00173816	-0.00619786	0.00779244
H	8.64981727	9.15050555	7.13553747	-0.01098523	0.00507124	0.00046946
O	8.25045871	9.88343754	6.59649900	-0.00518009	-0.00966844	-0.00193499
H	7.27080935	9.93143472	6.84938804	-0.00050994	0.01132064	-0.00947795
H	4.57754221	8.96117305	3.91214914	0.00409848	-0.00994341	0.00248120
O	5.18407456	9.70353306	4.14779907	-0.00759468	0.00127278	-0.01210717
H	5.20889854	9.79101099	5.18644754	0.01040343	-0.02050832	0.00615060
H	5.33239072	2.41768855	9.20475377	-0.00452890	0.00151022	-0.00233379
O	4.42995809	2.18904354	8.72827189	-0.00236398	0.00221123	-0.00194952
H	4.23593658	1.27674749	9.15157708	0.00264703	-0.00341453	-0.00164097
H	8.05904248	6.90276207	9.97719462	0.00338084	0.00219246	0.00532970
O	9.02937941	6.61386952	9.78072612	0.00088264	0.00399407	0.00635450
H	9.37956278	6.41784335	10.68602477	-0.00296655	0.00292580	-0.00641816
H	0.26936307	-0.64187446	9.39357681	0.00310822	0.00150881	0.00467837
O	1.13778798	-0.11971797	9.41570749	0.00690560	0.00560640	0.01386832
H	1.10177725	0.44018592	8.60266358	0.00833762	0.00106347	0.00136536

H	8.93784888	7.35647839	6.31311999	-0.00656482	-0.01089171	-0.01529241
O	9.77908249	7.53636863	6.94087039	0.00308894	-0.01294390	-0.00373656
H	10.35401781	8.22451566	6.50059079	-0.00649135	-0.01310514	-0.01124862
H	6.32352323	-0.92862712	8.45031624	-0.00129521	0.00862439	-0.01026686
O	6.19787899	-0.32989642	7.64595749	0.00094452	-0.00487943	0.00895046
H	6.72370466	-0.77058844	6.87695257	-0.00990870	-0.01141143	-0.01879204
H	8.54106472	2.92865877	6.24636127	0.01041683	0.00354757	-0.00958621
O	8.31828964	3.91555999	6.16895122	-0.00497982	0.00791075	-0.00835564
H	8.27550082	4.18108124	7.16325212	0.00365279	0.00484430	-0.00999101
H	8.03541150	0.80474907	4.95245242	-0.00882453	0.00173876	-0.01841498
O	7.90937984	0.75451163	3.94680373	-0.00022661	-0.00562491	-0.01532844
H	6.95709707	1.05434835	3.83411263	-0.00112819	0.03145932	-0.00665938
H	2.23988932	5.88314946	5.22780057	-0.00691959	0.00157960	-0.00280094
O	2.81011708	6.19500249	4.37392767	0.00998641	0.00342138	-0.01586390
H	2.50467021	5.55869245	3.67206668	-0.01141825	-0.01601020	-0.01045695
H	7.15001837	4.82966642	8.50488626	-0.00921643	0.00502042	-0.00954278
O	8.04634825	4.41724376	8.72515392	-0.00462029	0.00627275	0.00435251
H	8.57201595	5.19345597	9.13428921	-0.00404383	0.00304499	-0.00395483
H	5.90307292	7.76160459	10.35241352	0.00301646	0.00297094	0.01085186
O	6.48600427	7.01379385	10.03240820	-0.00426669	0.00329181	0.01785611
H	5.89905120	6.17993198	10.20428994	0.00175649	0.00099219	0.00827625
H	3.71008922	7.70002555	9.64600157	0.00133251	0.00063310	0.00620467
O	4.20937364	8.49397863	10.05048224	-0.00076081	-0.00312185	0.01271097
H	3.65623630	8.75825675	10.82823900	0.00161070	-0.00666200	-0.00930616
H	2.65896351	8.91513174	6.30273450	0.01052193	-0.00422980	0.00078846
O	1.84908578	9.47961165	6.04317081	0.00293222	-0.00556852	0.00560138
H	1.53248284	9.08627801	5.15802688	-0.00036599	-0.00278135	-0.00066700
O	9.65332638	2.71722831	3.74487546	0.00450930	0.00398381	-0.01201429
O	9.91684085	5.55879037	4.03610952	-0.00838397	-0.00220366	-0.02077416
H	8.98594686	1.93999474	3.69218495	-0.00294063	-0.01056107	-0.00511075
H	10.22304112	2.61418084	2.94436293	0.01213586	-0.00929897	0.01050678
H	10.17714610	5.28286037	4.98058241	-0.00151930	0.00561196	0.00131266
H	9.79435823	6.54679531	4.08939122	-0.01817917	0.00778107	-0.00050754
O	2.88882794	6.54771644	8.85558787	0.00469328	-0.00541664	-0.00217341
H	3.25797449	6.97187644	7.98622300	0.00408198	-0.00383948	-0.00763009
H	2.08545238	7.17678724	9.05590794	0.00513486	-0.00778301	0.00024738
O	4.80612333	5.04341083	10.33756029	0.00111247	-0.00862367	0.00865121
H	4.04988981	5.54043927	9.90739923	0.00647278	-0.00188949	0.00743108
H	4.53236295	4.96079615	11.28774513	0.01091159	0.00148973	-0.00136051
H	7.39698438	6.22144728	6.19912002	0.01665273	0.01376472	0.01427873
H	4.40781346	4.35515789	7.04813404	-0.00053163	-0.01758569	0.00019217

*COH;*OH

117

Lattice="9.84 0.0 0.0 0.0 8.52169 0.0 0.0 0.0 11.75" Properties=species:S:1:pos:R:3:forces:R:3

C	1.06301537	0.78031624	0.72756977	-0.00096191	-0.00371788	-0.00431716
C	1.06294893	2.20953348	0.64732680	0.00190056	-0.00528026	-0.00239892
C	2.29325946	2.91986856	0.78445923	0.00551689	0.00278033	0.00141201
C	2.29875467	4.34402911	0.88064776	0.01217490	0.00758685	0.00987732
C	1.07021421	5.05417732	0.75211891	-0.00054286	0.00595556	0.00502513
C	1.07248823	6.46800278	0.86454325	0.00598979	0.00168089	0.00818330
C	2.31350889	7.16893879	1.00271610	0.00432384	-0.00524442	0.00234087
C	2.29292530	8.59114703	0.89230823	0.00235822	-0.00686368	0.00181744
C	3.52154170	0.78076511	0.96607115	-0.00095144	-0.00441751	0.00981502
C	3.51828978	2.20433296	0.93482307	0.00509204	0.00529493	-0.01292146
C	4.70804229	2.89939052	1.30389025	0.01737392	-0.00494668	-0.05039307
C	4.56047712	4.21815062	1.84667289	0.01206172	0.00182924	0.01844479
C	3.46859851	5.02320463	1.36624199	0.00274897	0.02082491	-0.00058270
C	3.52174160	6.46938587	1.35302247	0.01275234	-0.00145489	0.00705472
C	4.75404596	7.22105093	1.52403301	-0.01694657	-0.00863057	-0.00424057
C	4.74689210	8.60057991	1.18466089	0.00931576	-0.01563990	0.01223167
C	5.98017691	0.80318461	1.11029843	-0.00062613	-0.00157059	0.00378540
C	5.97935135	2.21404915	1.20361377	0.00968548	0.00768337	-0.00169590
C	7.23671733	2.91075187	1.13312116	-0.01541471	0.00611488	-0.00333917
C	7.38587678	4.24661187	1.61914172	-0.00968676	0.00282211	0.01186796
C	6.02360670	6.67363372	1.97469273	0.00023047	-0.00843405	0.00561289
C	7.22507247	7.22645096	1.38372021	0.01035572	-0.00954532	-0.03961030
C	7.21179569	8.60674055	1.02806633	-0.00522556	-0.00539805	-0.00802777
C	8.42650809	0.78223415	0.76708075	0.00033295	-0.00285792	-0.00157638
C	8.42280381	2.20819363	0.73743149	-0.00719935	0.00440070	0.00264385
C	9.66403421	2.92070473	0.62687969	-0.00889052	0.00315004	-0.01467565
C	9.66860259	4.34741217	0.71126108	-0.00998142	0.00316498	-0.01433323
C	8.47360972	5.03551161	1.12538270	-0.01228890	0.01086489	-0.03166964
C	8.44865114	6.47886046	1.17920537	-0.00660747	0.00216178	-0.01103194
C	9.66526606	7.17878980	0.90825078	-0.00213630	-0.00447029	0.01450137
C	9.66575979	8.59938520	0.77424745	-0.00635695	-0.00364662	-0.00058832
Cu	6.61241361	4.26793193	5.09691511	-0.00824208	-0.05361731	-0.02443756
Cu	4.68675855	5.74297569	4.75516145	0.03882302	0.02918760	0.01479549
Cu	6.80686955	6.88967842	3.93008313	-0.01534135	0.06168892	0.06713509
Cu	4.51070910	3.37894470	3.74550074	-0.00032597	0.00291331	0.02902623
Cu	6.03583498	4.94992134	2.78529550	0.00382126	-0.00670090	0.05477586
Cu	8.31815023	4.42166694	3.40230289	-0.04651560	-0.02017979	0.08024192
C	5.34952025	5.09061357	6.38232718	-0.00299258	0.00333232	0.02656020
O	5.57375895	5.39775102	7.62434367	-0.02101698	0.00217654	0.01241496
O	7.72533412	7.00687533	5.69585505	0.00021359	0.00159577	-0.00809828
H	2.95709117	3.30862680	8.28249608	0.00712788	0.00562245	-0.00860525
O	2.31409768	4.04922221	8.04802203	0.01042557	0.00284426	0.00084275
H	2.62905424	4.85395855	8.55504964	0.00860506	-0.00143711	-0.00496809
H	0.85061205	8.58280159	3.03627045	-0.00638954	-0.00361190	0.01213383
O	0.97668370	8.17971529	3.93113281	-0.01044014	0.00404303	-0.01560103

H	1.74373890	7.52062885	3.83694731	0.00801652	-0.00182844	-0.00883519
H	2.60079196	2.28505276	5.48932715	0.01078036	0.01316891	-0.00571084
O	2.96645441	3.16743205	5.09358183	0.00209689	0.00410481	-0.01080897
H	3.18736166	3.71791100	5.89052803	0.00237833	0.00181115	0.00712507
H	7.33681962	1.75601544	9.45563149	-0.00805723	-0.01215653	0.00010519
O	6.82439040	2.52950119	9.79600160	-0.00658864	-0.00971723	0.01258149
H	6.93746188	2.51055249	10.79109565	-0.01126128	0.00218052	-0.00159918
H	4.05373084	-1.58666988	6.29633376	0.00498820	0.00390931	0.00018426
O	3.85989379	-0.77513024	6.88356280	-0.00894913	0.00495654	-0.00291467
H	4.87035160	-0.55576041	7.18704665	0.00390939	-0.00445177	-0.01173206
H	0.85221708	6.35679616	6.72184276	0.00424146	0.00149008	0.00127627
O	1.35220806	5.60174345	6.24755657	0.00129583	0.00578875	0.00381677
H	1.66648147	4.88617686	6.91506747	0.00043010	0.01117490	-0.01244608
H	5.00621903	1.93682653	7.34388589	0.00434151	0.00637275	-0.01590481
O	5.61090816	1.92943364	6.50545917	-0.00096334	-0.00261869	0.00741392
H	5.63296921	2.91187030	6.23528902	-0.00274275	0.00107714	-0.00043805
H	8.67480812	9.12982863	7.02633067	-0.00828732	0.00030749	-0.00230570
O	8.24794177	9.93464647	6.61259960	-0.00716650	-0.00580846	-0.00320456
H	7.25772433	9.90016745	6.81179277	-0.00076600	0.01006889	-0.00924478
H	4.52135713	8.93085868	3.95555965	0.00394584	-0.00956439	-0.00033007
O	5.10757819	9.70669178	4.13059180	-0.00756029	0.00012373	-0.01338063
H	5.17166440	9.82888474	5.16133963	0.00987612	-0.02119908	0.00410749
H	4.93214483	2.65552501	9.17251322	0.00149787	0.00541260	0.00379989
O	4.20215926	2.22148603	8.64810716	0.00732211	-0.00009735	-0.00014414
H	4.11877482	1.31050395	9.13147488	0.00286250	-0.00445663	-0.00049654
H	8.01632880	7.01230007	10.01200092	0.00297548	0.00221683	0.00700581
O	8.93224380	6.59639183	9.77036317	0.00233129	0.00329797	0.00692299
H	9.28744850	6.32952184	10.65773405	-0.00432255	0.00294418	-0.00758772
H	0.30255212	-0.72710889	9.38601999	0.00373657	0.00206778	0.00622975
O	1.16588982	-0.20551215	9.43760643	0.00613152	0.00594521	0.01489911
H	1.16945541	0.30661055	8.59316840	0.01199583	0.00335791	0.00671669
H	8.99345364	7.45156296	6.30205805	-0.00414566	-0.00913233	-0.01518320
O	9.78303582	7.71140889	6.96900768	-0.00056817	-0.01747858	-0.01103642
H	10.40276086	8.33777181	6.49316898	-0.00750426	-0.01171514	-0.01189175
H	6.37008234	-0.92100357	8.41668843	-0.00113276	0.00109863	-0.01376603
O	6.26581163	-0.39582928	7.56367764	-0.00033795	-0.00652549	0.00932126
H	6.79801780	-0.91566655	6.83699667	-0.01211903	-0.01522601	-0.01568976
H	8.66248496	3.01786663	6.24987552	0.00611167	0.00637637	-0.01345834
O	8.51741604	4.01899984	6.21606899	-0.00137685	0.00106402	-0.00302469
H	8.30997724	4.22510383	7.18427846	0.00668560	0.00222920	-0.01431376
H	7.96445509	0.83339698	4.91279059	-0.00916652	0.00103838	-0.01841740
O	7.80310334	0.74253791	3.91986927	0.00066999	-0.00597308	-0.01325615
H	6.84475987	1.04227291	3.82583430	-0.00021032	0.03220428	-0.00689790
H	2.22090493	5.97787815	5.14545245	-0.00970433	0.00194215	-0.00492283
O	2.75109545	6.23776641	4.25278148	0.01273585	0.00581265	-0.01423864

H	2.40232375	5.57561630	3.59889161	-0.00533782	-0.01596651	-0.01097295
H	7.45024337	3.84935132	9.23011527	-0.00693375	0.01153450	0.00059814
O	7.69114457	4.64887737	8.63405745	-0.00631050	0.00716415	0.00047083
H	8.24826202	5.36709034	9.13736048	-0.00704839	0.00265018	-0.00156499
H	5.76895122	7.86978607	10.26252274	0.00415288	0.00034987	0.00845286
O	6.47837283	7.17895486	10.05092256	-0.00380970	0.00388019	0.01870101
H	5.98889250	6.29324451	10.23945577	-0.00036226	0.00214846	0.00982233
H	3.75882172	7.72279865	9.68937178	0.00173224	-0.00141614	0.00593954
O	4.25812123	8.57062807	10.00209891	0.00243781	-0.00297545	0.01170698
H	3.82480867	8.81969076	10.86330622	0.00185877	-0.00774417	-0.00989662
H	2.74477594	8.83019164	6.29491937	0.01033267	-0.00644839	-0.00085517
O	1.97768596	9.45646453	6.04269600	0.00641449	-0.00682516	0.00532715
H	1.63292826	9.10082395	5.15256190	0.00153221	-0.00362002	-0.00127390
O	9.59812292	2.68369434	3.73550045	0.00368216	0.00366322	-0.01170908
O	9.91452490	5.54588117	3.96749854	-0.01298589	-0.00233576	-0.02016276
H	8.92099155	1.92278419	3.64547311	-0.00272374	-0.01455646	-0.00540399
H	10.17462696	2.60947220	2.93617906	0.01352013	-0.00943431	0.00878811
H	10.18627951	5.31577322	4.91909951	0.00070608	0.00707068	-0.00092899
H	9.79299805	6.53690353	3.99375854	-0.01873615	0.00631658	-0.00126279
O	2.99468341	6.53575940	9.02686842	0.00582735	-0.00420376	0.00581998
H	3.33354334	6.90758204	8.11513843	0.00586365	-0.00007859	-0.00656699
H	2.15619022	7.13415086	9.19050024	0.00458559	-0.00488567	0.00293830
O	5.01023959	5.02689501	10.36999202	0.00185090	-0.00708007	0.01266312
H	4.20854278	5.46703708	9.95952957	0.00697919	-0.00207266	0.00795434
H	4.76502348	4.92382497	11.32782519	0.01140225	0.00315414	-0.00015459
H	7.84355578	6.04107417	5.88675435	0.01600097	0.01126177	0.00854529
H	6.51787117	5.03184258	8.09781282	-0.00920595	0.00280143	-0.01472797

*HCOO;*OH (oxidated)

115

Lattice="9.84 0.0 0.0 0.0 8.52169 0.0 0.0 0.0 11.75" Properties=species:S:1:pos:R:3:forces:R:3

C	1.27683500	0.72777831	0.85176447	-0.00030684	-0.00108277	-0.00049656
C	1.28690850	2.15338514	0.77390480	0.00205362	-0.00382792	0.00148609
C	2.52166120	2.86815797	0.87109095	0.00156730	-0.00453894	-0.01093394
C	2.52622068	4.29261410	0.92840693	0.00472531	0.00700136	-0.00606265
C	1.29137698	4.99851046	0.84993112	0.00281078	0.00314947	0.00520316
C	1.28842629	6.41186595	0.95651173	0.00341618	0.00240863	0.00328097
C	2.52205675	7.12206130	1.11205866	0.00249466	-0.00245755	0.00728349
C	2.50293384	8.54507722	1.04708033	0.00288263	-0.00227476	0.00734043
C	3.73199748	0.73781007	1.13781363	0.00482289	0.00482670	0.02280812
C	3.74120290	2.15557278	1.05997556	0.00907129	0.00215288	-0.00984939
C	4.94988418	2.85695196	1.38179421	0.02443166	-0.00716010	-0.03559146
C	4.84370065	4.22551023	1.74125185	0.01225986	0.00149218	-0.00221904
C	3.71058726	4.98656610	1.34671701	0.00583484	0.01998511	-0.02871317
C	3.73655741	6.42517644	1.40466732	0.00865136	-0.00202065	-0.00205575

C	4.96205941	7.16487063	1.57782762	-0.01249096	0.00044924	-0.01492643
C	4.95952515	8.55398701	1.31061927	0.00849412	-0.00783902	0.01207915
C	6.20316885	0.74955742	1.25473726	-0.00460918	0.00663299	0.00415959
C	6.22022821	2.15296150	1.37770594	0.00702289	0.00556754	0.00297891
C	7.48755004	2.84427989	1.35651673	-0.02204689	-0.00446381	0.00486365
C	7.66709869	4.16923725	1.88614179	-0.00717788	0.00249601	0.01630316
C	6.23084898	6.59411210	2.03102098	0.00435573	-0.00894592	0.00899282
C	7.43810075	7.14283965	1.43204193	0.00830314	-0.00742102	-0.02929316
C	7.42804069	8.53582136	1.13097289	-0.00386815	-0.00159622	-0.01009307
C	8.64229681	0.71948944	0.88688757	-0.00038975	-0.00374985	-0.00170968
C	8.65117762	2.14756046	0.90820236	-0.00986506	0.00379715	0.00308284
C	9.89228895	2.85957349	0.79470960	-0.00386968	0.00099012	-0.00196401
C	9.89392458	4.28312460	0.87736201	-0.00865987	0.00261717	-0.00081740
C	8.71033825	4.96519793	1.30325923	-0.00746853	0.01376067	-0.01637051
C	8.66460217	6.40321983	1.26835511	-0.00866910	-0.00329515	0.00336502
C	9.88016246	7.11536503	0.98759666	0.00219232	0.00398063	0.01024865
C	9.88055227	8.53692854	0.87030001	-0.00432185	-0.00087037	-0.00104056
Cu	6.17712356	3.52933842	4.87837118	0.03860138	-0.06203497	-0.00945802
Cu	4.64204145	5.65004492	4.52243433	-0.06163393	0.01182108	0.00426024
Cu	6.73775740	6.96672697	3.98352331	-0.00367500	0.04007267	0.04784249
Cu	4.18726466	3.52147886	3.55134603	0.03345622	0.00895603	0.11504325
Cu	6.21857680	4.89354452	2.90741088	0.00771020	-0.00429683	0.07090206
Cu	8.30617963	4.12919626	3.85784916	-0.01534378	-0.01608116	0.06433367
C	6.24106105	5.91493558	6.55215646	0.00590668	0.02645048	0.04454474
O	5.34804754	6.00474282	7.44430213	-0.01717781	0.01385163	0.02050355
O	6.91449621	6.93914367	6.05976603	0.00761931	0.00934739	0.01173036
H	4.02712767	3.41994824	8.40798287	0.00560903	0.00478702	-0.01674073
O	3.76466322	3.88116666	7.51101702	0.00257460	0.00387093	-0.01113946
H	4.36053879	4.71312103	7.50853850	0.00698620	0.00533845	-0.01746639
H	1.17001507	8.23396439	3.31932436	-0.00070343	0.00251218	0.00644820
O	1.11769384	7.73460859	4.16780418	-0.00841994	0.01034001	-0.02490015
H	2.10927097	7.37754346	4.43744631	0.01935791	-0.00805416	-0.01234997
H	2.16019813	2.50417530	4.94383873	-0.00301386	0.00597759	-0.00327748
O	2.34412704	3.06541458	4.08366646	0.01512503	0.00372236	-0.01930202
H	1.71357369	3.85168219	4.11119003	-0.01656821	0.00849453	-0.00503709
H	7.61720324	3.17284422	9.39133711	-0.00470871	-0.00745648	0.00016242
O	7.24817725	2.30494990	9.76155126	-0.00300617	-0.01776535	0.00654768
H	7.50915804	2.32785624	10.71724312	-0.00481456	-0.00316560	-0.00400625
H	3.56746623	-0.81569387	6.23617796	0.01636735	-0.01131665	-0.00209499
O	3.57436159	-0.14343205	7.04878315	0.01334600	0.00383279	0.00062566
H	4.52755774	-0.01717344	7.34244214	-0.00042640	-0.00674979	-0.00822863
H	1.33028080	5.81904862	7.34433643	-0.00095057	-0.00123533	-0.00730944
O	1.63764718	5.19866034	6.63606885	0.00089413	0.00562327	-0.00427526
H	2.32155628	4.58708594	7.07655070	0.00803282	0.00287815	-0.01848489
H	5.77895166	1.72640315	7.04260471	0.00266149	0.01640912	-0.00850391

O	5.39819473	2.34722987	6.34340793	0.01581672	0.00053919	0.00538443
H	4.65082764	2.94210009	6.79469567	-0.01033533	-0.00264735	0.00111464
H	9.05119480	8.85469765	6.64729783	-0.00835972	0.00142985	-0.00926956
O	8.45759321	9.69263553	6.61297194	-0.00495914	-0.00033794	-0.00211413
H	7.74453204	9.51066245	7.30121557	-0.00175280	0.00166408	-0.00404976
H	4.30452068	8.58714448	4.31865973	0.01510479	0.00046097	-0.01238103
O	4.78855473	9.44904365	4.24021352	-0.01912444	-0.01688543	-0.01275288
H	4.80030234	9.84897155	5.17351871	-0.01098272	-0.02586220	-0.00862264
H	5.67809249	2.55115200	9.66348444	-0.00403972	-0.00878670	0.00083140
O	4.65925330	2.73209887	9.62499733	0.00614607	-0.00973297	0.01318609
H	4.25376828	1.80989484	9.75566326	0.00737262	-0.00372570	0.00262087
H	7.74617401	7.16045894	10.01689205	0.00057421	0.00602502	0.00803340
O	8.69437386	6.81133569	9.79305673	0.00130825	0.00653599	0.00919599
H	9.07728200	6.60801206	10.68590779	-0.00172493	0.00002217	0.00265329
H	-0.12998151	-0.37383216	9.29465482	-0.00301327	0.00526999	-0.00137146
O	0.69753682	0.19857219	9.23816866	-0.00119780	0.00446315	0.01070215
H	0.65907494	0.63375888	8.35061527	0.00199398	-0.00422165	-0.00587848
H	8.76744056	6.95720177	6.32433425	-0.00655104	-0.01085983	-0.01313089
O	9.65763670	7.37737945	6.50176383	0.00130714	-0.00573700	0.00013141
H	10.05320439	7.50252997	5.58290291	-0.01351487	-0.00337547	-0.00030897
H	6.23046432	-0.29134174	8.62681847	-0.00241262	0.00094010	-0.00601673
O	6.21989436	0.26632893	7.76203315	-0.00648698	0.00033067	0.00373228
H	6.44765574	-0.42567423	7.04060608	-0.00016372	0.00081326	-0.01378801
H	8.67770419	2.76862146	6.26347828	-0.01044901	0.00779382	-0.01514867
O	8.52001538	3.74947333	6.05882720	0.00313286	0.00108230	-0.00587129
H	8.31436476	4.16164023	6.95236410	-0.00733610	-0.00238412	-0.03254019
H	7.73141900	0.69612981	5.14376170	-0.00998078	0.01799115	-0.01047997
O	7.44829765	0.46425940	4.18537394	-0.00072351	-0.00827793	-0.01439831
H	6.49249530	0.81357109	4.10927966	0.00170933	0.04112630	-0.00420979
H	2.64746849	6.16059732	5.70538194	-0.00627144	0.00601690	0.00237713
O	3.20368717	6.79381371	5.12577965	0.01078039	-0.01798621	-0.00920359
H	6.84421064	4.93901404	8.56512816	-0.00761666	-0.00415757	0.00278300
O	7.79455566	4.65055897	8.60206853	-0.01757448	0.00485352	-0.00590064
H	8.25308698	5.44775866	9.07031306	-0.00732355	0.00292552	-0.00902466
H	5.47929568	8.03952759	10.30564932	0.00552913	0.00148220	0.00582322
O	6.21739745	7.39985849	10.00807118	-0.00356461	0.00465863	0.01464157
H	5.68988567	6.51312617	10.02699911	-0.00254256	0.00365402	0.00530594
H	3.42594938	8.00059947	9.69964758	0.00305881	0.00324015	0.00497776
O	4.00361696	8.75315218	10.11740669	0.00083793	-0.00137972	0.00820405
H	3.63493036	8.85067027	11.03222161	-0.00209521	-0.00854382	-0.01500979
H	2.53621945	9.50805636	6.46861681	0.00370670	-0.00074361	-0.00332027
O	1.75850728	10.03084429	6.06226689	-0.00382631	-0.00508588	-0.00429299
H	1.35225769	9.37037493	5.43700567	0.00133779	0.00943628	0.00098665
O	9.48538743	2.23324536	3.89763883	0.00139581	-0.00278649	-0.01400609
O	10.25754396	5.04788084	4.23905754	-0.00668089	0.00343065	-0.01977628

H	8.79839797	1.53912016	3.66373988	-0.00143203	-0.01259254	-0.00616847
H	10.13350551	2.26309400	3.15166417	0.00094133	-0.00947414	0.00624433
H	10.42761141	5.02651232	5.23617953	0.00580944	0.00359991	-0.00868702
H	10.45484866	6.00482124	3.98529728	-0.00918954	-0.01559008	-0.01295037
O	2.56930212	6.91175178	8.97202756	0.00887585	-0.01065581	0.00513640
H	2.98413988	7.34122273	8.13346238	0.00866997	-0.00383908	-0.00371580
H	1.75651566	7.53874536	9.10346463	0.00683172	-0.00595748	0.00336425
O	4.59500838	5.39204138	10.22005946	0.00076744	0.00215415	0.01281477
H	3.76247678	5.72429794	9.77789692	0.00308525	-0.00383560	0.00494740
H	4.56075383	4.38971250	10.18621730	0.00367233	0.00838884	0.01085420
H	6.59978041	4.90436848	6.20093900	0.00600713	-0.01075963	-0.02336680

*COOH;*OH (oxidated)

115

Lattice="9.84 0.0 0.0 0.0 8.52169 0.0 0.0 0.0 11.75" Properties=species:S:1:pos:R:3:forces:R:3

C	1.20618449	0.60604091	0.80263958	-0.00071627	-0.00254509	-0.00522785
C	1.21353103	2.03298069	0.75132136	0.00429089	-0.00409561	-0.00041440
C	2.43632934	2.74463159	0.92083500	0.00115696	-0.00062675	0.00294240
C	2.44422385	4.16872920	0.97026279	0.00779068	0.00384633	0.00433496
C	1.22285914	4.87876091	0.79724197	-0.00332722	0.00291360	0.00405749
C	1.21643302	6.29233443	0.88073071	0.00210455	0.00287378	0.00265768
C	2.44630498	6.99545894	1.06234649	0.00141781	-0.00171349	0.00713912
C	2.42894980	8.42050235	0.99808800	0.00018389	-0.00268076	0.00412567
C	3.65342679	0.61654897	1.10649009	-0.00088340	-0.00291688	0.01828562
C	3.65789458	2.03616691	1.08910422	0.00435814	0.00293113	-0.00372236
C	4.86386492	2.73230166	1.39692020	0.01979793	-0.00774344	-0.02684216
C	4.73009126	4.07629093	1.86521511	0.01312933	0.00409727	0.01694360
C	3.61702156	4.85721250	1.40280127	0.00053499	0.02086283	-0.00495083
C	3.65151701	6.29227355	1.36806522	0.01111457	-0.00267696	0.01150310
C	4.89228013	7.02936238	1.43627937	-0.00949898	-0.00723332	-0.00285429
C	4.88552828	8.43127573	1.21032183	0.00884553	-0.01189458	0.01220109
C	6.11877420	0.63545307	1.11663799	0.00266367	-0.00238424	0.00201495
C	6.12579589	2.04110856	1.28446076	0.00440968	0.00953927	0.00828684
C	7.38869049	2.73583641	1.20408721	-0.01361476	0.00063485	0.00359824
C	7.54023462	4.08473086	1.63270467	-0.01038364	-0.00391777	0.01051018
C	6.15857957	6.43775402	1.73837437	-0.00565464	-0.01193638	-0.00451244
C	7.35002285	7.03145932	1.20662790	0.00113771	-0.00540791	-0.06316808
C	7.34139498	8.43439606	0.94839827	-0.00585221	0.00149375	-0.01825937
C	8.56792038	0.61048708	0.75532371	-0.00135565	-0.00575929	-0.00007038
C	8.56835759	2.03602684	0.78119526	-0.01146729	0.00468838	-0.00121483
C	9.81737243	2.74095363	0.69684286	-0.00712240	0.00306146	-0.01012073
C	9.82716398	4.16401567	0.75216882	-0.00880586	0.00191768	-0.01472544
C	8.62384313	4.85584518	1.10385874	-0.00733574	0.00508229	-0.03931112
C	8.58752903	6.29574949	1.06501650	-0.00596174	0.00126847	-0.02101225
C	9.80999842	7.00148057	0.85917194	-0.00426383	-0.00225471	0.01192044

C	9.80795008	8.42517663	0.76680035	-0.00376647	0.00013000	0.00188972
Cu	6.77896966	4.38032081	5.14328408	-0.00469205	-0.04883843	-0.01361182
Cu	5.02947640	5.90746544	4.82472413	-0.03342308	-0.00311072	-0.01860981
Cu	7.23189076	6.75780510	3.64893362	0.03930029	0.02237889	0.06536480
Cu	4.38896218	3.73010646	3.83453749	0.01347273	0.03464000	0.05291289
Cu	6.19884530	4.84293482	2.77885077	0.01160325	-0.00280921	0.05866958
Cu	8.52291782	4.45038965	3.44356638	-0.02144151	-0.01474584	0.08778667
C	6.04480316	5.76432227	6.53200584	-0.00978916	0.00914036	0.01784952
O	5.70944351	5.24838017	7.64762388	-0.00930639	0.00858683	0.01705499
O	7.04302975	6.76326583	6.68975061	0.00460038	0.01324482	0.01557334
H	3.71488605	3.25297930	8.29580324	0.00426884	-0.00016051	-0.01594976
O	3.59538523	3.78598704	7.41940193	-0.00091327	0.00009698	-0.00941480
H	4.35023378	4.47399784	7.53725537	0.00731090	0.00644516	-0.01778478
H	1.06055143	8.25801780	3.27069727	-0.00808534	-0.00046809	0.00625849
O	1.13935511	7.73398671	4.10376355	-0.01032355	0.00759893	-0.02339705
H	2.17004733	7.50877784	4.26894607	0.01518503	-0.00504756	-0.01020117
H	2.29150410	2.51101174	5.12389208	0.00316195	0.00655158	-0.00749406
O	2.58588161	3.07806917	4.30902254	0.00896111	0.00519885	-0.01913066
H	1.87960874	3.77160608	4.16223399	-0.02158916	0.00377292	-0.00791284
H	7.52435905	3.16448984	9.42897241	-0.00531933	-0.00047095	0.00228666
O	7.02013688	2.33122956	9.78135581	-0.00441136	-0.00554442	0.01636227
H	7.43143158	2.11657588	10.65893836	-0.00408204	-0.00286452	-0.00490040
H	3.72944910	-0.70646369	6.14893735	0.00399563	0.00362031	0.00764018
O	3.70111976	-0.15535030	7.04628766	0.01610470	0.00465264	0.00580832
H	4.62019942	0.11055603	7.31551488	-0.00057845	-0.00906139	-0.00872429
H	1.38358649	5.92580163	7.10655043	0.00433954	-0.00410977	-0.00377588
O	1.75787149	5.31809264	6.41768792	0.00309217	0.00533089	-0.00268669
H	2.37514361	4.65393127	6.90287635	0.00949293	0.00462354	-0.01667617
H	5.79514159	1.86952355	6.83960560	0.00048185	0.02226764	-0.01389603
O	5.43163906	2.48273873	6.13938984	0.01406935	-0.00756144	0.01232748
H	4.61925658	2.94252672	6.59337111	0.01159135	0.00989668	-0.01778299
H	9.09035860	8.88526803	6.45911072	-0.00950429	0.00246498	-0.00803033
O	8.43673338	9.65171548	6.29719578	-0.00256813	0.00162179	-0.00190840
H	7.69462649	9.44734458	6.97244629	0.00081159	-0.00123934	-0.00881583
H	4.41702124	8.68723887	4.29658193	0.00891785	0.00217153	-0.01278079
O	5.01919876	9.45594514	4.12121484	-0.00963133	-0.01379644	-0.01440894
H	5.05353898	10.00887028	4.98970219	0.00960467	-0.01607020	-0.00171889
H	5.27053883	2.60489096	9.71796624	-0.00255905	-0.00198908	0.00532701
O	4.26778556	2.60395975	9.62253024	0.01108842	-0.01092534	0.01232069
H	4.04617999	1.61665954	9.76389208	0.00576255	-0.00169234	0.00340051
H	7.70184291	6.93992116	9.84702330	0.00271777	0.00383383	0.00537005
O	8.72399914	6.74585698	9.70308557	0.00067452	0.00641806	0.00910376
H	9.06836233	6.61806794	10.62580484	-0.00281526	-0.00036805	0.00097261
H	-0.19028901	-0.42271263	9.23945484	-0.00610352	0.00437562	-0.00136833
O	0.58768787	0.22645010	9.20750477	-0.00420954	0.00566013	0.00999611

H	0.56635239	0.63124949	8.30491624	-0.00056269	-0.00407761	-0.00338400
H	8.99836183	6.81418119	6.57361144	-0.00617813	-0.01444578	-0.01280230
O	9.79959837	7.40134181	6.51705105	-0.00181019	-0.00269940	-0.00203425
H	10.08973997	7.40191839	5.55242265	-0.01607252	-0.00676318	-0.00375283
H	6.71075574	1.21173291	8.74865654	-0.00311139	-0.01183668	-0.00213950
O	6.44654874	0.62711852	7.93799433	-0.00512810	-0.00822726	-0.00142319
H	6.49103582	-0.32641057	8.25194956	-0.00231378	0.00286775	-0.00283730
H	8.66604380	2.85571230	6.15952414	0.00862614	0.00065871	-0.01074588
O	8.50168961	3.85127610	6.17620819	-0.00376315	0.00479583	-0.00654317
H	8.42241490	4.08638097	7.16808367	0.00465191	0.00012358	-0.00793757
H	7.83983925	0.54278052	4.99953030	-0.00687082	0.00959750	-0.00723626
O	7.48058746	0.18608692	4.08957858	0.00333600	-0.00601996	-0.02317557
H	6.47804936	0.50907912	4.05549618	-0.00574560	0.02688253	-0.00075587
H	2.84228902	6.34142333	5.47610667	-0.01096897	0.00013170	-0.00408475
O	3.39116052	6.99605549	4.92179411	0.00553477	-0.01618543	-0.01211778
H	7.08544493	4.81828866	8.40823277	-0.00983514	0.00031464	-0.00775117
O	7.99651272	4.45042485	8.68050928	-0.00698880	0.00366929	0.00308315
H	8.44066248	5.27055250	9.11887228	-0.00513922	0.00420873	-0.00340606
H	5.53883398	7.74660574	10.02546154	0.00269714	0.00074797	0.00637503
O	6.22540383	7.03675759	9.77754184	-0.00443852	0.00232492	0.01494162
H	3.44446027	7.90434248	9.71472383	0.00188540	0.00338563	0.00820807
O	4.15540315	8.57407031	10.07141122	0.00442283	0.00128449	0.01094469
H	4.00271362	8.60187326	11.05214075	0.00351985	-0.00740780	-0.01239062
H	2.53993887	9.46711554	6.55396491	0.00147152	-0.00258531	-0.00172302
O	1.74718435	9.98447203	6.17916922	-0.00269951	-0.00651668	-0.00281552
H	1.37207891	9.35759784	5.50415961	0.00244775	0.00740030	0.00077260
O	9.46611964	2.31258534	3.73283704	0.00033158	-0.00425271	-0.00939296
O	10.39124451	5.04012903	4.09325721	-0.00745173	0.00236912	-0.02105090
H	8.74177844	1.64372317	3.57056997	-0.00466919	0.00860856	-0.00657585
H	10.01847508	2.26262268	2.91143250	0.00707462	-0.00631417	0.01391090
H	10.57299131	5.02780584	5.09880527	-0.00333913	-0.00049493	-0.00728140
H	10.62070187	5.99535187	3.85149609	0.00610039	-0.00326152	-0.00962065
O	2.47573225	6.97150090	8.94222647	0.00550000	-0.01012138	0.00304326
H	2.94604024	7.35247620	8.12288648	0.00550112	-0.00526118	-0.00325175
H	1.68432604	7.63584275	9.05852029	0.00539013	-0.00515294	0.00376252
O	4.29008313	5.28388804	10.21300071	0.00165633	0.00292232	0.01710906
H	3.49994799	5.69932466	9.76534778	0.00204044	-0.00618634	0.00653326
H	4.13711747	4.29898942	10.18333997	0.00358563	0.01004024	0.01344473
H	5.64725970	6.19867639	9.87349325	0.00258476	0.00540265	0.00821473
H	7.13238180	7.13129457	5.75279059	0.00653851	-0.00319941	0.01847668

*CH$_2$O;*OH

118

Lattice="9.84 0.0 0.0 0.0 8.52169 0.0 0.0 0.0 11.75" Properties=species:S:1:pos:R:3:forces:R:3

C	1.24173625	0.72402119	0.78044980	-0.00102026	-0.00569676	-0.00276626

C	1.24228541	2.15256700	0.72214791	0.00162824	-0.00505300	-0.00143396
C	2.46416185	2.86254060	0.91051580	0.00442170	0.00216352	0.00457311
C	2.46783131	4.28617376	1.02724525	0.00941525	0.00509074	0.02024932
C	1.25219344	4.99375468	0.85081922	-0.00283034	0.00569662	0.00431141
C	1.25250361	6.41477168	0.92728339	0.00215440	0.00230951	0.00316715
C	2.48582798	7.10985769	1.09515894	0.00275666	-0.00373902	0.00621805
C	2.46407146	8.53403556	0.98186936	-0.00003574	-0.00620393	0.00890577
C	3.69039004	0.72513117	1.04791004	0.00138186	-0.00356335	0.01243136
C	3.68393839	2.14679594	1.03032292	0.00519614	0.00471408	-0.01055443
C	4.88862942	2.83970810	1.34442636	0.01695428	-0.00378363	-0.05359380
C	4.76394166	4.16428218	1.85104400	0.01448976	0.00437159	0.00965174
C	3.64313348	4.96710021	1.47438257	0.00041264	0.01857913	0.00482795
C	3.69757812	6.41265421	1.43188130	0.01207090	-0.00495717	0.00654652
C	4.93312913	7.15771344	1.50841240	-0.01522713	-0.00782571	-0.00610375
C	4.92182048	8.54385260	1.18582319	0.00918390	-0.01291686	0.01075175
C	6.15134142	0.74354029	1.05952697	-0.00209271	-0.00055529	-0.00177037
C	6.15482234	2.15566340	1.18332255	0.00794363	0.00812465	-0.00206139
C	7.40714772	2.84266582	1.10374772	-0.01490673	0.00754125	-0.00316562
C	7.55888378	4.19314559	1.55621515	-0.01160227	0.00448480	0.01270180
C	6.22811400	6.61662819	1.88250278	0.00238689	-0.00984610	0.01025883
C	7.39893061	7.16475490	1.24858730	0.01120682	-0.01465125	-0.04244669
C	7.38063123	8.55324182	0.92612981	-0.00490689	-0.00341470	-0.01300755
C	8.60434587	0.72622095	0.72122293	0.00093377	-0.00175600	-0.00146991
C	8.60333337	2.15113952	0.72840368	-0.00848826	-0.00059346	-0.00243993
C	9.84650596	2.85963422	0.66250687	-0.00650183	0.00208038	-0.02019989
C	9.86114892	4.28694442	0.74755873	-0.00826864	0.00535192	-0.02111837
C	8.64801080	4.98670005	1.06869098	-0.01163559	0.00298271	-0.00990369
C	8.62012931	6.42542932	1.06804733	-0.00905289	0.00179521	-0.01326519
C	9.85137498	7.12396328	0.87582518	-0.00250003	-0.00719431	0.01022837
C	9.84436287	8.54560514	0.76043829	-0.00368708	-0.00044670	-0.00065315
Cu	6.88105268	4.38799749	4.97844092	0.00501129	-0.02169060	-0.01628083
Cu	4.84302141	5.68921344	4.70785400	0.01393731	-0.00230721	0.03826769
Cu	6.88135820	6.94398939	3.78374085	-0.00717825	0.03072579	0.10193961
Cu	4.70708800	3.38852324	3.76680262	0.01188700	0.02758580	0.04246238
Cu	6.25416956	4.91099662	2.72825086	0.01163926	-0.00884885	0.05669253
Cu	8.58685413	4.32487017	3.25975666	-0.03941034	-0.02059580	0.06722820
C	6.30876582	6.02664046	6.09565438	-0.00491085	-0.00610472	-0.00514726
O	5.82068511	4.66311953	8.46546416	0.00543135	0.00439602	-0.01263764
O	7.08043397	7.17108212	5.76604137	0.00651152	0.01907617	-0.01042421
H	3.28036012	3.43347913	7.93781612	-0.00260644	0.00687349	-0.01252228
O	3.45527471	4.22768811	7.37376792	0.01639421	0.00682195	-0.00019377
H	4.93278267	4.54924524	7.99194003	0.00731010	0.01241007	-0.01391344
H	0.69445650	8.19181764	3.25132456	-0.00760470	-0.00231770	0.00364939
O	0.92513549	7.92322240	4.17404678	-0.00993909	0.00621261	-0.01864132
H	1.85660802	7.45030032	4.15581279	0.00959588	-0.00144681	-0.01080051

H	2.49262664	2.44832157	5.20766368	0.00918948	0.01431472	-0.01084547
O	3.00106452	3.28186681	4.87536547	-0.00026974	0.00137620	-0.02125979
H	3.24724001	3.67677481	5.77493755	-0.00197326	0.00610488	0.00639299
H	7.86792191	2.81275658	9.32956774	-0.00460354	-0.00437021	-0.00081370
O	7.26504917	2.11931059	9.73877412	-0.00165223	-0.01115082	0.00714488
H	7.27710081	2.38628971	10.69364548	-0.00570117	0.00488804	-0.00611427
H	3.64537338	-1.02882619	6.00956681	0.00034864	0.01749103	0.00847876
O	3.57862900	-0.47101161	6.82907418	-0.00115551	-0.00287346	-0.00667460
H	4.55347751	-0.32504833	7.19893084	0.00275789	0.00053871	-0.00939655
H	0.81336399	6.16155071	6.78358293	0.00174840	0.00465373	-0.00142904
O	1.48553305	5.46770194	6.48087615	-0.00349090	0.00616599	0.00028699
H	2.56102946	4.75468762	7.24226722	0.01872228	0.00433501	-0.00819200
H	5.19413426	2.15336417	7.56487857	0.00420085	0.00796469	-0.01643665
O	5.64180928	2.13305509	6.61026548	0.00053957	0.00197155	0.00577354
H	5.60557472	3.07474270	6.27625163	-0.00415185	0.00224403	0.01257192
H	8.64764644	9.04388677	6.85242773	-0.01023413	0.00546801	-0.00273219
O	8.24595191	9.85982121	6.43057888	-0.00845819	-0.00586993	0.00169758
H	7.31328967	10.00524776	6.78528103	0.00033141	0.00891864	-0.00843551
H	4.38868679	9.10259679	4.11451104	0.00729928	-0.01100238	-0.00437443
O	5.15181084	9.70837649	4.27051192	-0.00683340	-0.00174915	-0.00817729
H	5.19131849	9.90304682	5.28471728	0.01064171	-0.02257058	0.00850665
H	5.71947525	2.30634950	9.31864008	-0.00552588	-0.00421886	0.00038431
O	4.73307220	2.31345701	9.00467546	0.00397307	-0.01498567	0.01167984
H	4.40422064	1.40222451	9.31734722	0.00538418	0.00193888	0.00049170
H	7.62271523	6.85893030	10.00587406	-0.00028065	0.00392730	0.00874402
O	8.60992098	6.77251549	9.77156069	-0.00712392	0.00363463	0.00625104
H	9.00934449	6.61417793	10.66740720	-0.00294256	-0.00082149	0.00172398
H	-0.16987194	-0.44515732	9.27844458	-0.00436625	0.00347116	-0.00007980
O	0.69830594	0.06454276	9.21113010	-0.00118389	0.00434182	0.01029230
H	0.68333069	0.45571264	8.30187552	0.00194403	-0.00289330	-0.00425635
H	8.51502555	7.14331565	6.31248952	-0.00462180	-0.01559085	-0.01862003
O	9.47174762	7.41064136	6.65255823	0.00490673	-0.01023062	-0.00059178
H	9.88480273	7.72153060	5.79906751	-0.01171445	-0.00525857	-0.00418522
H	6.09107191	-0.83048320	8.51603540	0.00089828	0.00718866	-0.01120521
O	5.96512304	-0.17614319	7.78131581	0.00328160	-0.00800084	0.01704175
H	6.50390950	-0.54105846	6.98372670	-0.00573654	-0.00437931	-0.00959107
H	8.72466483	2.89682873	6.11885873	0.00911601	0.00082660	-0.01190257
O	8.60689905	3.89757590	6.03591368	-0.01438853	0.00309735	-0.00310196
H	8.60028604	4.19366945	7.02422530	0.00297924	0.00264019	-0.01310772
H	7.93523548	0.75951282	4.88457741	-0.00549097	0.00996402	-0.01560524
O	7.79673667	0.60923519	3.88369531	-0.00081646	-0.00697168	-0.01638329
H	6.85553456	0.94676135	3.77339496	-0.00080186	0.04287545	-0.00552025
H	2.08652245	5.92609273	5.78240175	0.01440915	-0.00117559	0.00008795
O	3.01141081	6.42570916	4.51261130	0.00979379	-0.00173808	-0.01616376
H	2.62046334	5.70259626	3.95167522	-0.00268173	-0.01302023	-0.01733355

H	7.36255441	4.44287857	8.51244628	-0.01056474	0.00013115	-0.00913203
O	8.37919602	4.36775775	8.58348745	-0.00620697	0.00809021	0.00029542
H	8.62896390	5.24445715	9.03023311	-0.00516653	0.00076215	-0.00473457
H	5.31373088	7.54689946	10.25932891	0.00241852	-0.00031651	0.00708273
O	5.95972731	6.74637764	10.18815775	0.00159559	0.00104307	0.01904938
H	5.35187333	5.97941900	10.51341575	0.00612753	0.00752889	0.01752618
H	3.42535015	7.80501955	9.62602749	0.00455058	-0.00148127	0.00671134
O	4.09759045	8.52453921	10.00347218	0.00199667	-0.00212767	0.01309165
H	3.72887331	8.78004773	10.88659238	-0.00126136	-0.00649888	-0.00991174
H	2.45966804	9.22736348	6.37963459	0.00382781	-0.00445738	0.00098626
O	1.73529906	9.88028776	6.10063200	-0.00626027	-0.00680197	-0.00195855
H	1.25632744	9.40660489	5.36520938	-0.00342812	0.00348251	-0.00109946
O	9.71298807	2.42109818	3.68918740	0.00210903	0.00119327	-0.01087395
O	10.17288668	5.22871985	4.06145744	-0.00867503	-0.00140040	-0.02060767
H	8.98094297	1.71918083	3.57999094	-0.00518538	-0.00873590	-0.00457010
H	10.29586889	2.30981368	2.89851961	0.00837061	-0.00774806	0.01313463
H	10.33387123	4.92222896	5.00959253	0.01013976	0.00468236	-0.00242563
H	10.13928307	6.22827981	4.13028496	-0.01398890	0.00184211	-0.00337468
O	2.54923604	6.78540640	9.01209329	0.00906358	-0.01537642	-0.00270651
H	2.87283715	7.01912218	8.08382769	0.00686645	-0.00692039	-0.00014725
H	1.72184438	7.41488683	9.09607266	0.00593907	-0.00822982	0.00314905
O	4.05927685	4.87983589	10.50212470	0.00016303	-0.00045901	0.00685973
H	3.41847814	5.35802797	9.90338562	0.00268669	-0.00077445	0.00749179
H	4.35758793	4.08264381	9.98180769	-0.00695498	0.00140347	0.02403161
H	5.33333954	6.30366960	6.59555473	0.00052920	-0.02129485	-0.00673744
H	5.76897397	5.57257033	8.87329813	-0.00375650	-0.00234556	-0.01236943
H	6.80829420	5.38498925	6.84970893	-0.00705022	0.00946063	-0.01232938

*CHO

118

Lattice="9.84 0.0 0.0 0.0 8.52169 0.0 0.0 0.0 11.75" Properties=species:S:1:pos:R:3:forces:R:3

C	1.07141765	0.74913002	0.78472993	-0.00131863	-0.00277640	-0.00506889
C	1.07432583	2.17394549	0.70770824	0.00207030	-0.00609399	-0.00282168
C	2.29934477	2.88475350	0.89378970	0.00506926	0.00214536	0.00425165
C	2.30391947	4.30742836	0.95451437	0.01264737	0.00561797	0.01374768
C	1.08677985	5.01920225	0.75610161	-0.00419459	0.00671341	0.00210824
C	1.08231995	6.43320515	0.86551378	0.00530576	0.00229124	0.00537954
C	2.31136998	7.13473803	1.05797456	0.00357691	-0.00657815	0.00350355
C	2.29605238	8.56229906	0.99278042	0.00267813	-0.00437074	0.00161809
C	3.52067877	0.75569058	1.12590005	-0.00016703	-0.00389438	0.01715692
C	3.51966821	2.17623915	1.09104179	0.00471408	0.00410587	-0.00574884
C	4.72079704	2.87709164	1.42330444	0.01519156	-0.00744801	-0.04931333
C	4.56970487	4.21540836	1.89601215	0.01354353	0.00495591	0.01246670
C	3.47008572	4.99153412	1.42375519	0.00210876	0.02096637	-0.00241090
C	3.51255943	6.42974666	1.37632466	0.01194876	-0.00506064	0.01041270

C	4.75638823	7.16391190	1.47050040	-0.00758935	-0.00878374	-0.00052760
C	4.75224268	8.57179328	1.26932652	0.01051090	-0.01047596	0.01998805
C	5.98817102	0.77293212	1.17716433	0.00298534	-0.00679987	0.00215384
C	5.99091340	2.18696144	1.29349950	0.00880267	0.00526201	-0.00076357
C	7.24645873	2.87912593	1.16391016	-0.01330471	0.00608821	-0.00099446
C	7.40590690	4.23763005	1.56013393	-0.01002716	-0.00175800	0.00756485
C	6.02022644	6.55777204	1.72966719	-0.01083375	-0.01578641	-0.01045798
C	7.22954300	7.17178418	1.28172897	0.00285869	-0.00449995	-0.04212392
C	7.21627460	8.58060373	1.02614733	-0.00705490	0.00104121	-0.01544757
C	8.43446236	0.75332987	0.78710616	-0.00405060	-0.00850652	0.00155036
C	8.43290875	2.17586686	0.76485328	-0.00836803	0.00185160	0.00255907
C	9.67750976	2.88294103	0.63500436	-0.00912375	0.00306381	-0.01746694
C	9.69093445	4.30699986	0.67385983	-0.01091795	0.00415629	-0.02077289
C	8.48900595	5.00223044	1.04272210	-0.01072096	0.00454504	-0.01810809
C	8.46430089	6.44087643	1.08433249	-0.00700634	0.00242146	-0.01971226
C	9.67792898	7.14680688	0.87533433	-0.01050381	-0.00705943	0.01351691
C	9.67407030	8.56914068	0.77820253	-0.00571700	-0.00043418	0.00069530
Cu	6.73138202	4.43415136	5.00839513	-0.00289348	-0.01706663	-0.02604418
Cu	4.80185233	5.87763707	4.82556645	-0.00653296	-0.00812763	0.02451782
Cu	7.15092488	6.88900340	3.66971721	0.04022517	0.02464208	0.09084076
Cu	4.61158873	3.51179173	3.88990299	0.02531443	0.01665013	0.04273065
Cu	6.06787393	4.98353399	2.75399701	0.00868774	-0.00258473	0.04819891
Cu	8.39764219	4.50547758	3.27882670	-0.04295558	-0.01221342	0.05990437
C	6.30685153	6.83937480	5.49670602	-0.00077870	0.00806871	-0.00430097
O	5.62632832	4.93475915	7.87020596	-0.00674421	0.00687352	0.01302753
O	7.06658156	6.56397629	6.63295134	0.01913201	0.00096943	0.00596971
H	3.44197966	3.22995228	8.03729075	0.00266201	0.00269776	-0.01304891
O	3.23167116	3.99512406	7.42539661	0.01609115	0.00567661	-0.00011624
H	4.13960529	4.46808490	7.43134189	0.01342769	0.01215038	-0.02358146
H	0.95901317	8.35015839	3.08389314	-0.00935224	-0.00251364	0.01396950
O	1.02999247	7.94394441	3.98553542	-0.00477168	0.00798644	-0.02473453
H	1.86949381	7.36483291	3.97627639	0.00953771	-0.00266542	-0.01043267
H	2.56143454	2.20152740	5.22568947	0.00780090	0.01176168	-0.00897910
O	2.93490271	3.11724130	4.93351575	0.00486373	0.00401170	-0.02153879
H	3.07974941	3.50141455	5.86647927	0.00215974	0.00725533	0.00039106
H	7.62723936	3.03579145	9.40236016	-0.00763689	-0.00250258	-0.00047051
O	7.08449359	2.27257015	9.79514498	-0.00252949	-0.01089193	0.00872151
H	7.13552147	2.45271938	10.76944871	-0.00663089	0.00321374	-0.00489662
H	4.25785660	-1.23152434	6.29340428	0.00117097	-0.00226643	0.00425818
O	3.82167594	-0.62975650	6.99266222	0.00305980	-0.00191042	-0.00276934
H	4.70481005	-0.32852473	7.50594907	0.00388535	-0.00322491	-0.00589242
H	0.76483177	6.18232999	6.88457334	0.00349583	0.00401731	0.00196663
O	1.33652818	5.45673436	6.46145179	-0.00101792	0.00582158	0.00522756
H	1.89354811	4.95781894	7.13835808	0.00046751	0.01286696	-0.00789479
H	5.17803355	2.11797161	7.57357266	0.00466280	0.00950682	-0.01856781

O	5.62087748	2.09152428	6.65119996	-0.00397064	-0.00533225	0.00902950
H	5.69168250	3.04863818	6.36150667	-0.00485099	-0.00168873	0.00571965
H	8.58906333	9.01717023	6.93589941	-0.01035307	0.00421938	-0.00072772
O	8.15826045	9.76549686	6.43047603	-0.00841659	-0.00793655	0.00206456
H	7.22543067	9.91027655	6.79880957	0.00006143	0.00392793	-0.00953027
H	4.51308223	9.06410546	4.05714913	0.00244191	-0.00531796	-0.00376901
O	5.21548269	9.73321233	4.23996556	-0.00558277	-0.00004362	-0.01526585
H	5.19678660	9.90666344	5.24895826	0.00732499	-0.01685658	0.00568480
H	5.63351875	2.36495722	9.41218789	-0.00345612	-0.00372671	-0.00028633
O	4.61340908	2.35880896	9.13211416	0.00293269	-0.00600503	0.00638545
H	4.30627685	1.44595501	9.47409516	0.00456178	-0.00166762	0.00338438
H	7.79213233	6.98522796	10.03922537	0.00151364	0.00301351	0.00457084
O	8.74706739	6.79739867	9.74110692	-0.00465308	0.00398884	0.00473787
H	9.19677321	6.62956363	10.61003604	-0.00288679	-0.00094907	-0.00201261
H	-0.01525779	-0.33339301	9.24643890	-0.00342319	0.00228133	0.00104579
O	0.85109618	0.17405961	9.18526405	0.00083083	0.00071315	0.01189688
H	0.87913560	0.48526053	8.24376888	0.00182018	-0.00445464	-0.00120486
H	8.50323009	6.98390480	6.70036186	0.00732214	-0.01119305	-0.01391662
O	9.46546816	7.40149233	6.82501688	0.00390171	-0.00807605	-0.00570163
H	9.71894307	7.69782745	5.91513274	-0.01667861	-0.00829754	-0.00284265
H	6.09673047	-0.57234099	8.95282770	0.00171701	0.00173413	-0.01031994
O	6.06316633	-0.10407993	8.07159619	0.00179730	-0.00811242	0.01111541
H	6.56698512	-0.78783067	7.49336844	-0.00906382	0.00396544	0.00796529
H	8.51833156	2.91325049	6.15792272	0.00756512	0.00117236	-0.00959197
O	8.34300805	3.90338503	6.11162139	-0.01332997	0.00713155	-0.00252699
H	8.34604672	4.19363966	7.09724547	0.00052514	0.00557045	-0.00922714
H	7.99748613	0.72732338	4.90956632	-0.00492051	0.00356315	-0.01381595
O	7.88251968	0.54940481	3.89960360	0.00028938	-0.00975906	-0.01841218
H	6.94856201	0.90823208	3.76653135	-0.00271043	0.04186699	-0.00663728
H	2.23620546	5.84294444	5.37646530	0.00240481	0.00069935	-0.00245425
O	2.80444128	6.05311296	4.48906987	0.01349215	0.01156522	-0.01161665
H	2.48552314	5.33479602	3.87725312	-0.00814299	-0.01135403	-0.01733702
H	7.11623327	4.62700306	8.44379649	-0.01153352	0.00381577	-0.01140869
O	8.09711092	4.43397775	8.65126741	-0.00895924	0.01082395	0.00294209
H	8.44749306	5.29632808	9.06790222	-0.00814600	0.00390998	-0.00421763
H	5.42454732	7.62143624	10.55743253	0.00696728	-0.00182210	0.00171517
O	6.17189662	6.95357458	10.37323329	-0.00360788	-0.00615421	0.01244010
H	5.69062776	6.08044363	10.48357786	0.00525818	0.00122689	0.01385223
H	3.46674654	7.79397323	9.74094509	0.00334275	-0.00041470	0.00649631
O	4.07688914	8.53169546	10.15726872	-0.00188016	-0.00085372	0.01169917
H	3.61966621	8.78387861	10.99981362	0.00033056	-0.00594062	-0.01221842
H	2.69013587	8.91808152	6.38758068	0.00478253	-0.00698837	-0.00047936
O	1.92620149	9.52240885	6.08295549	0.00036156	-0.01081088	-0.00086003
H	1.50231422	9.02753188	5.32377229	-0.00035582	-0.00128472	0.00080172
O	9.57435660	2.65834906	3.70594423	0.00217558	-0.00139925	-0.01287110

O	10.01782518	5.37375664	4.05776079	-0.01057477	-0.00386889	-0.01926834
H	8.95935901	1.86332794	3.59585212	-0.00541642	-0.00165283	-0.00683809
H	10.21699050	2.58019352	2.95897890	0.00966962	-0.00774085	0.01061455
H	10.12390815	5.10193047	5.01879568	0.00135449	0.00548516	-0.00032850
H	10.07209896	6.37542354	4.03706084	-0.01464925	0.00489758	-0.00517841
O	2.60866359	6.75140206	9.02945328	0.00444846	-0.01351724	0.00117826
H	3.03901824	7.03105630	8.14096970	0.00661453	-0.00686075	-0.00455597
H	1.81987963	7.41642930	9.09739926	0.00561721	-0.00745695	0.00350052
O	4.36835223	4.91905017	10.19693103	0.00137124	0.00096061	0.02147225
H	3.53209359	5.37253369	9.87650337	0.00084873	-0.00045340	0.00933297
H	4.27605261	3.93775637	9.98909996	0.00511366	0.00606365	0.01466555
H	6.14623493	5.71736421	7.39670084	-0.02075622	-0.01442226	0.00614285
H	5.22895593	5.20840739	8.76026277	0.00750833	0.00892401	-0.02032546
H	6.27017274	7.94901769	5.30124379	0.00413794	0.00779043	0.01190232

*COH

118

Lattice="9.84 0.0 0.0 0.0 8.52169 0.0 0.0 0.0 11.75" Properties=species:S:1:pos:R:3:forces:R:3

C	1.06682697	0.73253062	0.79446252	-0.00146253	-0.00296019	-0.00492527
C	1.06863849	2.15682964	0.70474711	0.00178915	-0.00585611	-0.00327337
C	2.29339703	2.86762072	0.87617625	0.00415604	0.00217061	0.00280073
C	2.29966729	4.29092685	0.94133746	0.01266946	0.00593260	0.01248712
C	1.08187010	5.00266719	0.75283545	-0.00424156	0.00666729	0.00068558
C	1.07899387	6.41720807	0.87006516	0.00502824	0.00202063	0.00492482
C	2.30883616	7.11686103	1.06819306	0.00257721	-0.00610653	0.00532815
C	2.29012011	8.54344197	1.00112967	0.00249936	-0.00476991	0.00236642
C	3.51589274	0.73584235	1.11420473	-0.00062482	-0.00436009	0.01763771
C	3.51375564	2.15571199	1.06463081	0.00607279	0.00571604	-0.00427781
C	4.71078548	2.85581712	1.39240201	0.01488298	-0.00416916	-0.03606669
C	4.55966123	4.18263427	1.89050520	0.01426688	0.00901854	0.01574365
C	3.46494956	4.96872449	1.42373492	0.00292239	0.02181158	-0.00329932
C	3.51260380	6.41143477	1.39423063	0.01398912	-0.00524279	0.01086349
C	4.75206703	7.14925471	1.51198368	-0.01134827	-0.01017921	-0.00051556
C	4.74566064	8.55132246	1.27227792	0.01003457	-0.01169067	0.01556865
C	5.98190945	0.75391263	1.16607017	0.00184087	-0.00388408	-0.00123483
C	5.98081820	2.16588976	1.26082514	0.00752712	0.00596106	-0.00056661
C	7.23534687	2.86152380	1.12722450	-0.01304846	0.00618756	-0.00370509
C	7.38665498	4.22029553	1.52346759	-0.01117056	0.00266011	0.01154414
C	6.03152053	6.56345004	1.81617660	-0.00448933	-0.01143666	0.00793594
C	7.23022604	7.16019131	1.30745091	0.00873999	-0.00570212	-0.05262917
C	7.21373444	8.56219908	1.02637114	-0.00681999	-0.00039254	-0.01574500
C	8.42961573	0.73566228	0.78328356	-0.00273450	-0.00639585	0.00081432
C	8.42638364	2.15808228	0.74538487	-0.00845023	0.00096441	0.00031076
C	9.67201570	2.86715788	0.62576376	-0.00850532	0.00269189	-0.01857021
C	9.68534249	4.29161360	0.67001889	-0.01060434	0.00440527	-0.02149311

C	8.47984225	4.98730105	1.03490225	-0.01145383	0.00544237	-0.01243730
C	8.45681221	6.42624009	1.08857632	-0.00755828	0.00166836	-0.01699148
C	9.67440922	7.13058947	0.88413356	-0.00812039	-0.00740144	0.01313461
C	9.67096705	8.55273752	0.79005715	-0.00493377	0.00048067	0.00154455
Cu	6.79572556	4.49522317	5.04799543	0.01634792	-0.02387144	-0.02027511
Cu	4.75355454	5.81917881	5.00447408	0.00457428	0.00932966	0.00788324
Cu	6.89797507	6.94462270	3.71347871	0.00484650	0.02207694	0.10426526
Cu	4.59098346	3.54406304	3.94252783	0.02932455	0.01022072	0.02072077
Cu	6.06817917	4.91101363	2.74796372	0.01122053	-0.01289462	0.03722712
Cu	8.38671800	4.47557977	3.24875981	-0.03767890	-0.02560265	0.05326015
C	6.42380986	6.49395356	5.49589578	0.00492816	0.01630305	-0.00060721
O	5.63004186	5.02527153	7.90255953	-0.00307115	0.00003020	0.01771604
O	7.27467906	6.67255829	6.64551943	0.01719713	0.00819715	0.01178025
H	3.39754137	3.13897708	8.12825914	0.00102476	0.00290331	-0.01483720
O	3.11709271	3.85936143	7.48513681	0.01865950	0.00569013	-0.00095001
H	3.98639538	4.34309101	7.41758349	0.01345506	0.01049130	-0.02186132
H	0.82997092	8.36556297	3.15833580	-0.00869243	-0.00338521	0.00915765
O	0.96189727	7.95458854	4.05149164	-0.00693079	0.00560989	-0.02194061
H	1.80064273	7.38075719	3.99700645	0.00875015	-0.00192857	-0.01025336
H	2.52915188	2.19211566	5.18027463	0.00506661	0.01071115	-0.00799196
O	2.83900048	3.13082432	4.88357541	0.00384855	0.00470478	-0.02004166
H	2.93299785	3.50155895	5.81571632	0.00042209	0.00624540	0.00145636
H	7.64481862	3.03598854	9.42042426	-0.00731774	-0.00270240	-0.00031721
O	7.07561601	2.28759592	9.79035701	-0.00205679	-0.01092193	0.00827369
H	7.15464160	2.41942789	10.77282007	-0.00595220	0.00260913	-0.00512613
H	4.28773189	-1.20802422	6.31930219	0.00242546	0.00282378	-0.00106309
O	3.82318615	-0.61534553	7.01592138	0.00140616	-0.00238549	-0.00043923
H	4.67487310	-0.28156103	7.53357071	0.00408471	-0.00449587	-0.00504553
H	0.87113642	6.27062614	6.83089557	0.00560585	0.00226130	0.00165258
O	1.35341080	5.45714030	6.46203731	-0.00011046	0.00505224	0.00641824
H	1.84897503	4.95208314	7.17338181	0.00076674	0.01295983	-0.00777151
H	5.19197191	2.15404055	7.58968834	0.00289929	0.01278264	-0.01906423
O	5.63940046	2.11398429	6.67579436	-0.00633062	-0.00028181	0.01009959
H	5.64011173	3.05672760	6.32292622	-0.00290149	0.00158999	0.01657548
H	8.63028456	9.17872724	6.99025774	-0.01146679	0.00754373	0.00161672
O	8.17246954	9.86366922	6.43460976	-0.00577170	-0.00880336	0.00167941
H	7.24518792	10.05623801	6.80116240	-0.00075318	0.00550364	-0.00825756
H	4.70722821	8.99466067	3.98196014	0.00133253	-0.00874803	0.00003257
O	5.22619260	9.80078860	4.22948424	-0.00539757	0.00067964	-0.01134970
H	5.22513405	9.84898229	5.24235755	0.00688804	-0.01822306	0.00321811
H	5.62065835	2.37676807	9.44441106	-0.00392080	-0.00440217	0.00003494
O	4.59309392	2.37756436	9.18554708	0.00313313	-0.00638931	0.00744841
H	4.28529228	1.45783926	9.51206815	0.00501444	-0.00270015	0.00468760
H	7.75620944	6.99306585	10.08005820	0.00203346	0.00372259	0.00613210
O	8.71032533	6.80923859	9.76763651	-0.00344535	0.00484274	0.00497458

H	9.17352430	6.68680493	10.64076074	-0.00320529	-0.00155934	-0.00288759
H	-0.03457371	-0.30307267	9.30052952	-0.00344124	0.00227750	0.00101236
O	0.84476371	0.17504390	9.21780410	0.00117965	0.00158995	0.01249705
H	0.86930737	0.46384274	8.26937579	0.00648003	-0.00002192	0.00118907
H	8.79906264	7.09860379	6.62985696	-0.00598053	-0.01168554	-0.01380319
O	9.69878681	7.56756551	6.71549347	0.00236749	-0.01364519	-0.01120520
H	9.90971750	7.84367371	5.78443707	-0.01331836	-0.00661331	-0.00364585
H	6.09858347	-0.66402538	8.92609054	-0.00131404	0.00275292	-0.00779535
O	6.04378301	-0.02599617	8.14866270	0.00400051	-0.01066813	0.00852578
H	6.59176740	-0.49121120	7.45421183	-0.00630887	-0.00574578	-0.00169957
H	8.67065560	2.94892077	6.13181701	0.00646623	0.00474436	-0.01270644
O	8.53688884	3.94773807	6.11170251	-0.01423503	0.00663008	0.00255576
H	8.53061299	4.19606781	7.09698113	-0.00138044	0.00411789	-0.01340172
H	7.96600038	0.71231094	4.89775585	-0.00650359	0.00117502	-0.01368386
O	7.82391442	0.55946039	3.90041601	0.00066931	-0.01152422	-0.01511359
H	6.88488372	0.93205317	3.81207252	0.00156946	0.04298381	-0.00611876
H	2.23492817	5.81688379	5.38253879	-0.00443490	0.00114085	-0.00271306
O	2.81967109	6.08266207	4.51689779	0.01119508	0.01196024	-0.00940782
H	2.55908407	5.37630452	3.86417044	-0.01751312	-0.00512834	-0.01792140
H	7.20937367	4.64671038	8.47265871	-0.01119319	0.00184847	-0.01049300
O	8.17414374	4.45613433	8.68192348	-0.00957823	0.00636423	0.00309310
H	8.49404904	5.30863913	9.15507999	-0.00668607	0.00279251	-0.00248909
H	5.39874940	7.65389383	10.56458044	0.00655302	-0.00157645	0.00160311
O	6.14217319	6.98291100	10.36213920	-0.00386235	-0.00719545	0.01227072
H	5.67540068	6.10916127	10.50225436	0.00465516	0.00067882	0.01452144
H	3.43322028	7.79667721	9.75807998	0.00310898	-0.00021163	0.00638559
O	4.03621504	8.53792234	10.16186194	-0.00100054	-0.00154144	0.00927465
H	3.59311480	8.77310920	11.01978294	0.00018482	-0.00669286	-0.01399038
H	2.71366593	8.93705518	6.37177077	0.00352180	-0.00611597	-0.00039926
O	1.95308529	9.53877461	6.06530938	-0.00014981	-0.00961835	0.00316791
H	1.53139607	9.04501746	5.30453151	-0.00032600	-0.00122372	0.00222170
O	9.54854606	2.58474974	3.70611170	0.00337667	-0.00023990	-0.01216399
O	10.01113379	5.34939350	4.03934424	-0.00928730	-0.00045660	-0.01923294
H	8.90246773	1.81417622	3.57026952	-0.00533749	-0.00024974	-0.00661435
H	10.17308755	2.53889266	2.94102803	0.01045320	-0.00896157	0.01129601
H	10.05984149	5.04176405	4.99305578	0.00518407	0.00810966	-0.00150733
H	9.99712415	6.34825718	4.06384870	-0.01276680	0.00336373	-0.00297217
O	2.56599963	6.73608898	9.03809511	0.00455530	-0.01346765	-0.00054919
H	3.00175830	7.03003717	8.16137564	0.00526282	-0.00725586	-0.00559165
H	1.78466927	7.40951776	9.12041006	0.00575904	-0.00739406	0.00349886
O	4.35621695	4.94088171	10.12509969	0.00043350	0.00120408	0.02112830
H	3.47654699	5.37515097	9.90881881	0.00171014	-0.00026330	0.00925445
H	4.25224193	3.94110330	10.01817417	0.00525134	0.00646352	0.01396507
H	5.27919220	5.54718234	7.08437210	-0.00932661	-0.01140829	0.01713432
H	5.06829042	5.17860767	8.76397408	0.00515592	0.00911602	-0.01869557

H	6.90670037	6.09193556	7.38084306	-0.00883137	0.00075048	-0.01487101

*CHOH;*OH

118

Lattice="9.84 0.0 0.0 0.0 8.52169 0.0 0.0 0.0 11.75" Properties=species:S:1:pos:R:3:forces:R:3

C	1.06392801	0.79968542	0.75866996	-0.00031914	-0.00394931	-0.00311190
C	1.06488297	2.22923746	0.68795885	0.00264096	-0.00522533	-0.00090242
C	2.29564539	2.94061848	0.83166994	0.00580024	0.00285691	0.00368827
C	2.29840176	4.36397153	0.93578636	0.01154751	0.00741991	0.01134242
C	1.06955855	5.07312860	0.80972083	0.00032700	0.00538443	0.00766092
C	1.07077873	6.48888359	0.91175231	0.00584744	0.00156359	0.00908462
C	2.31285665	7.18977363	1.03912832	0.00475931	-0.00540761	0.00203500
C	2.29359562	8.61140468	0.92158442	0.00234805	-0.00673065	0.00256843
C	3.52259339	0.80165350	0.98721885	0.00039547	-0.00389864	0.00872092
C	3.51999343	2.22465234	0.96982784	0.00475082	0.00469073	-0.01274581
C	4.71385116	2.91902939	1.33499110	0.01621923	-0.00347518	-0.05025348
C	4.57980654	4.24453924	1.85084486	0.01249700	0.00229492	0.01667566
C	3.47478044	5.04626488	1.40523838	0.00300989	0.01953749	-0.00036148
C	3.52342970	6.49417839	1.38626792	0.01267842	-0.00056087	0.00740002
C	4.75515575	7.24616584	1.54526570	-0.01738245	-0.00984042	-0.00362270
C	4.74837169	8.62119013	1.19201006	0.01004679	-0.01615282	0.00854234
C	5.98267677	0.82165375	1.11422512	-0.00252098	-0.00101424	0.00435308
C	5.98559633	2.23118819	1.22205066	0.00984993	0.00790259	-0.00065518
C	7.24257046	2.92331126	1.16768374	-0.01507097	0.00672542	0.00013457
C	7.38922082	4.25541490	1.66487206	-0.00957694	0.00309336	0.01342933
C	6.02524813	6.70359760	2.00543667	0.00054350	-0.00809405	0.00825238
C	7.22426820	7.24450747	1.39334827	0.01136503	-0.01042440	-0.04433810
C	7.21194508	8.62320223	1.03010701	-0.00524467	-0.00558060	-0.00800307
C	8.42707284	0.80078558	0.78183901	0.00051990	-0.00292281	-0.00227274
C	8.42530319	2.22574486	0.76557531	-0.00735797	0.00417665	0.00193758
C	9.66635458	2.93838505	0.67302524	-0.00786258	0.00383936	-0.01388385
C	9.66990740	4.36494425	0.76558693	-0.00866230	0.00369127	-0.01258686
C	8.47363726	5.05410900	1.16993858	-0.01158959	0.01059147	-0.02868000
C	8.44670072	6.49727869	1.20581432	-0.00654534	0.00250632	-0.01114133
C	9.66486027	7.19863115	0.94130690	-0.00258120	-0.00487035	0.01448554
C	9.66564551	8.61790754	0.79911892	-0.00539342	-0.00486096	0.00001562
Cu	6.54121997	4.27672353	5.13055561	0.00210899	-0.04789558	-0.04518817
Cu	4.69189237	5.70997552	4.78014559	0.04131761	0.03574512	0.00829603
Cu	6.81857074	6.93771325	3.91809915	-0.02215153	0.05400490	0.08414092
Cu	4.54262174	3.35634831	3.77130967	-0.00112562	0.00190016	0.04755568
Cu	6.03341284	4.97755585	2.81657010	0.00164350	-0.00594279	0.06012756
Cu	8.31057304	4.42923358	3.43789763	-0.04479811	-0.02046656	0.08351285
C	5.03853800	4.95930108	6.50561074	-0.00606244	0.01839566	0.02525895
O	5.66178757	5.39030047	7.67105889	-0.00967248	-0.00166175	0.02003071
O	7.72568112	7.01236937	5.69693628	0.00347809	0.00140620	-0.01158387

H	3.08022575	3.25838317	8.33153207	0.00563500	0.00481822	-0.00648691
O	2.33856795	3.91716284	8.12421378	0.01170740	0.00300201	-0.00007484
H	2.59721554	4.75133844	8.61394054	0.00770122	-0.00071701	-0.00362941
H	0.85964960	8.57309290	3.01640534	-0.00652611	-0.00237045	0.01416753
O	0.97088996	8.17215320	3.91509480	-0.00947826	0.00338681	-0.01677710
H	1.74799256	7.52503386	3.84348434	0.00805016	-0.00163161	-0.00785652
H	2.47650190	2.26449240	5.42583913	0.01435005	0.01429486	-0.00850858
O	2.89604947	3.12173449	5.03704628	0.00336847	0.00447448	-0.01233317
H	2.99548344	3.69940910	5.83467187	0.00536482	0.00084370	0.00064968
H	7.27719156	3.07888784	9.41399841	-0.00603482	-0.00075971	0.00057477
O	6.68899361	2.33397558	9.76762665	-0.00083911	-0.01061558	0.01164535
H	6.56813479	2.58351292	10.71908879	-0.00990691	0.00364091	-0.00263334
H	4.01798224	-1.62989903	6.30296937	0.00484890	0.00456456	-0.00067146
O	3.77228010	-0.76016238	6.79248911	-0.00401941	0.00493023	-0.00275416
H	4.70360828	-0.48894945	7.11778271	0.00024555	-0.00371636	-0.01272658
H	0.86619121	6.27034707	6.72923336	0.00367229	0.00129748	0.00127700
O	1.35091859	5.48394065	6.26849432	0.00182887	0.00484731	0.00347944
H	1.66868947	4.79591247	6.95578175	0.00068491	0.01008868	-0.01136688
H	5.06841225	1.86072407	7.35919749	0.00283804	0.00457751	-0.01529580
O	5.64084457	1.73723314	6.46975555	-0.00287635	-0.00472771	0.00351963
H	5.69852134	2.68168077	6.11395884	0.00221830	-0.00526582	0.00367241
H	8.67443592	9.19418355	7.03755556	-0.01069297	0.00549670	-0.00214882
O	8.23165195	9.88199387	6.47374817	-0.00311693	-0.01088059	-0.00112262
H	7.23909170	9.90240491	6.71984552	-0.00511636	0.00581483	-0.00790584
H	4.46846653	8.90725802	3.80363230	0.00390402	-0.00773980	0.00621076
O	5.02822544	9.67422511	4.07837479	-0.00821596	0.00273468	-0.01231739
H	5.09739618	9.68011240	5.10638134	0.00767766	-0.02084982	0.00339720
H	5.27253035	2.44601699	9.11848313	-0.00384140	0.00302563	-0.00231480
O	4.39153933	2.19888288	8.61062397	-0.00264487	0.00259472	-0.00418852
H	4.18877442	1.30791059	9.07368858	0.00276285	-0.00442658	-0.00278714
H	8.10286644	6.92615679	10.04694925	0.00262549	0.00124073	0.00847100
O	9.02663755	6.54842501	9.78954740	0.00103744	0.00235237	0.00639993
H	9.38009810	6.23532968	10.66083432	-0.00397894	0.00388611	-0.01002382
H	0.28671530	-0.75030595	9.46829848	0.00347456	0.00139185	0.00482803
O	1.14112371	-0.20011480	9.48736695	0.00687622	0.00664654	0.01505682
H	1.08082847	0.35787884	8.67592750	0.00998443	0.00213501	0.00615664
H	9.05892955	7.41204776	6.32766900	-0.00476177	-0.00862865	-0.01487115
O	9.84797469	7.58870186	6.99045187	-0.00087180	-0.01819821	-0.00742498
H	10.44329572	8.27859433	6.56555792	-0.00479425	-0.01284095	-0.01194542
H	6.59992957	-0.78623562	8.50333761	-0.00248372	0.00287356	-0.01375960
O	6.26348556	-0.66944286	7.55919671	-0.00136072	0.00746829	0.00571851
H	6.96810629	-0.96450273	6.80057353	-0.01253056	-0.01482812	-0.01652033
H	8.54601440	2.97784581	6.15143504	0.01159732	0.00471102	-0.01390826
O	8.31928835	3.96616348	6.14817649	-0.00404290	0.00206474	-0.00846647
H	8.26234155	4.16783546	7.16443963	0.00306615	0.00395073	-0.01095752

H	7.93788778	0.82591903	4.89489333	-0.00672009	0.00396748	-0.01631339
O	7.76671393	0.73138286	3.89753659	-0.00117164	-0.00641617	-0.01550514
H	6.82555764	1.06415312	3.78594559	-0.00008390	0.03622109	-0.00638010
H	2.23880978	5.92073276	5.14427172	-0.01049862	0.00192606	-0.00529868
O	2.76284176	6.23060912	4.27437941	0.01178079	0.00532849	-0.01388475
H	2.44455649	5.58587054	3.58724839	-0.00883108	-0.01595996	-0.00927330
H	7.01763311	4.81523151	8.40336591	-0.00795941	0.00351940	-0.01063644
O	7.92818044	4.43019187	8.63770319	-0.00878314	0.01042658	0.00062998
H	8.44880902	5.19601707	9.07037692	-0.00707503	0.00484128	-0.00594341
H	5.88820675	7.88643879	10.34723326	0.00356715	0.00070831	0.00907955
O	6.56378235	7.17260694	10.13251680	-0.00477367	0.00367211	0.01925989
H	6.01715085	6.30606748	10.27686296	0.00014384	0.00086609	0.00867695
H	3.75949655	7.70742242	9.68424630	0.00249934	-0.00152356	0.00531799
O	4.25609153	8.54517897	10.00364614	0.00117715	-0.00466091	0.01125840
H	3.76701613	8.82210697	10.82270361	0.00209663	-0.00657494	-0.00871495
H	2.61092427	8.86979661	6.25237667	0.01078219	-0.00610132	-0.00235164
O	1.82102450	9.46809852	6.03179526	0.00563905	-0.00514108	0.00453433
H	1.48816740	9.10719406	5.13389587	-0.00024664	-0.00312145	-0.00138833
O	9.57423811	2.66846155	3.73827247	0.00319959	0.00384033	-0.01129705
O	9.90321606	5.54387670	4.02214699	-0.01023787	-0.00269628	-0.02038749
H	8.89454489	1.91327090	3.62799992	-0.00306460	-0.01462502	-0.00553355
H	10.13546543	2.61866431	2.92563822	0.01473756	-0.01146817	0.01046620
H	10.20206065	5.30014053	4.96213990	-0.00012747	0.00540088	-0.00047937
H	9.80002042	6.53621049	4.03615509	-0.01811322	0.00672730	-0.00198889
O	2.92814399	6.52258286	9.00589603	0.00685259	-0.00512234	0.00113241
H	3.24179935	6.87107192	8.09872481	0.00441477	-0.00204524	-0.00749986
H	2.11671186	7.16096023	9.18576865	0.00509516	-0.00685614	0.00165425
O	5.01671352	5.06733198	10.39908216	0.00029451	-0.00669087	0.00967623
H	4.23977808	5.46435531	9.91202639	0.00838666	-0.00384573	0.00585726
H	4.69853314	5.00278402	11.33784859	0.01015303	0.00245052	-0.00001617
H	7.82116564	6.04799449	5.90551401	0.01289655	0.00958311	0.01040076
H	4.50926137	4.01728568	6.78933591	-0.01516339	-0.01116349	0.00555483
H	5.88090415	6.40634685	7.57866980	0.00197021	-0.00917525	-0.01397228

*HCOOH;*OH (oxidated)

116

Lattice="9.84 0.0 0.0 0.0 8.52169 0.0 0.0 0.0 11.75" Properties=species:S:1:pos:R:3:forces:R:3

C	1.27263951	0.73005863	0.86349459	-0.00020805	-0.00152460	-0.00101657
C	1.28333421	2.15575233	0.80181823	0.00293890	-0.00393462	0.00151854
C	2.51648276	2.86952702	0.90125081	-0.00424214	-0.00654283	-0.00974574
C	2.51841586	4.29212606	0.95052987	0.00369013	0.00504736	-0.00737992
C	1.28510949	5.00087600	0.86793558	0.00085359	0.00279956	0.00509429
C	1.28205236	6.41503818	0.95662499	0.00255881	0.00297831	0.00285924
C	2.51517920	7.12383636	1.09356909	0.00099917	-0.00098427	0.00786286
C	2.50077881	8.54903642	1.03377053	0.00205772	-0.00113578	0.00819936

C	3.73086129	0.74320031	1.11411739	0.00062514	-0.00061590	0.02131262
C	3.74273935	2.16329146	1.06862256	0.00874349	0.00057415	-0.00647348
C	4.94867526	2.86242477	1.39920711	0.03179410	-0.01549647	-0.03934682
C	4.83229930	4.22631325	1.79148871	0.00991187	0.00515452	0.00664399
C	3.70523716	4.98669109	1.35814632	0.00242591	0.03312584	-0.03566502
C	3.72542562	6.42524179	1.36571097	0.01354659	0.00130110	0.00671755
C	4.95792030	7.15535363	1.46923445	-0.01267200	-0.00778947	-0.00941772
C	4.95993202	8.55563027	1.23537394	0.00240872	-0.00422343	0.00276282
C	6.19780241	0.75507155	1.19609971	-0.00109367	0.00730683	-0.00752731
C	6.21147652	2.16046794	1.37964392	0.00219340	0.01011333	0.01022287
C	7.47942886	2.85047649	1.38763414	-0.01635998	-0.00550016	0.00603081
C	7.66749568	4.17099352	1.93066469	-0.00597077	0.00408135	0.01787087
C	6.21108001	6.55562805	1.85647609	0.00185294	-0.01256730	0.00894509
C	7.42623938	7.13958316	1.34908347	0.01640899	-0.01151323	-0.05613074
C	7.41874321	8.54357507	1.06448277	-0.00102833	0.00073031	-0.01796388
C	8.63862925	0.72820606	0.87370962	-0.00146155	-0.00619096	-0.00047183
C	8.64415547	2.15723561	0.92639117	-0.01068068	0.00358480	0.00120970
C	9.88884240	2.86283392	0.82702166	-0.00450926	0.00148218	-0.00133299
C	9.89056036	4.28404044	0.91053881	-0.00888876	0.00246570	0.00022868
C	8.70644415	4.96784855	1.33139006	-0.00729651	0.01432542	-0.01221844
C	8.66358559	6.40365747	1.24726256	-0.00600242	-0.00627674	0.00097624
C	9.87632206	7.11724517	0.98024910	-0.00095124	0.00363442	0.00978925
C	9.87625833	8.54186793	0.86614172	-0.00471752	-0.00032045	-0.00193603
Cu	6.13896194	3.61544275	4.90979995	0.03283292	-0.03119989	-0.01501615
Cu	4.94626588	5.82626236	4.88707712	-0.01501716	0.01657924	-0.00675893
Cu	6.73187821	7.18641497	3.69719081	-0.01949845	0.02456604	0.09424969
Cu	4.10425366	3.85774649	3.63403992	0.03612984	0.01182303	0.13896881
Cu	6.22298393	4.96785258	2.93575169	0.01466733	0.00359452	0.07545658
Cu	8.30676066	4.10730125	3.90470731	0.00211708	-0.03823907	0.04423306
C	6.20649074	5.51409493	6.43673892	-0.01459634	0.00118955	0.00705787
O	5.31659385	5.96858397	7.28764338	-0.02149256	0.01602947	0.02200848
O	7.35930500	6.40456167	6.26582427	0.01720877	0.01333114	0.00985188
H	4.07098935	3.39433129	8.46577652	0.00557111	0.00583867	-0.01800486
O	3.79469518	3.83847777	7.55990033	0.00158769	0.00261046	-0.00850026
H	4.34965275	4.70564959	7.52965351	0.00645930	0.00619831	-0.01670222
H	1.07532012	8.18609161	3.31127030	-0.00061482	0.00320863	0.00731602
O	1.08372850	7.66006721	4.14533441	-0.00890770	0.00749765	-0.02409900
H	2.09843751	7.35130006	4.37408721	0.01612890	-0.00587852	-0.01092373
H	2.19236734	2.57468559	4.93691711	-0.00382036	0.00324779	-0.00214391
O	2.34653228	3.14843838	4.08700212	0.01168819	0.00328762	-0.01965267
H	1.64473190	3.87798442	4.10636574	-0.02473430	0.00261782	-0.00515134
H	7.58053746	3.19474310	9.39986548	-0.00525224	-0.00677437	-0.00022265
O	7.23952556	2.29718681	9.73488196	-0.00302216	-0.01815476	0.00471443
H	7.56094364	2.25036128	10.67047091	-0.00460226	-0.00446539	-0.00335015
H	3.52526247	-0.80423763	6.16436488	0.00518142	0.00146298	0.00764152

O	3.56615773	-0.13234185	6.97869528	0.01170974	0.00448560	-0.00032158
H	4.52944690	-0.05954723	7.25867628	-0.00111562	-0.00865841	-0.00996963
H	1.68741863	5.85436492	7.45341818	-0.00008137	-0.00083126	-0.01309569
O	1.71102072	5.22134859	6.67680671	0.00073302	0.00582351	-0.00609020
H	2.35188179	4.50774459	7.00634618	0.00977236	0.00248968	-0.02028859
H	5.74395462	1.68940540	6.94742537	0.00171961	0.01554082	-0.00654945
O	5.32823456	2.32237977	6.29238252	0.01399093	-0.00283705	0.00448067
H	4.62015696	2.93650676	6.81674153	-0.01041710	-0.00308529	0.00085068
H	9.07518445	8.88329217	6.63617944	-0.00864738	0.00038120	-0.00992798
O	8.40932439	9.67390970	6.57527038	-0.00526823	0.00129744	-0.00068127
H	7.71992437	9.46283908	7.27842963	0.00028098	0.00275600	-0.00345793
H	4.22070570	8.57870154	4.30509232	0.01433288	0.00198685	-0.01291833
O	4.65189379	9.46458634	4.19519709	-0.02210292	-0.01718225	-0.01471375
H	4.64676991	9.88490523	5.11864814	-0.00614723	-0.00881634	-0.01567499
H	5.68052758	2.55540406	9.71919588	-0.00397355	-0.00875127	0.00133588
O	4.66096785	2.74432508	9.69004101	0.00630804	-0.00917195	0.01428566
H	4.24799344	1.82411456	9.79408686	0.00748339	-0.00368479	0.00276209
H	7.74866006	7.16062506	10.02035863	0.00093821	0.00615953	0.00854150
O	8.70089465	6.81777518	9.81652106	0.00172930	0.00672311	0.00895666
H	9.06701531	6.61758058	10.71847451	-0.00225729	0.00008024	0.00217650
H	-0.11852004	-0.35586782	9.37670498	-0.00207844	0.00539951	0.00037190
O	0.69934831	0.22798864	9.29461675	-0.00142829	0.00401889	0.01144880
H	0.61979062	0.64717025	8.40261264	0.00291957	-0.00078606	-0.00139977
H	9.16668034	6.90625778	6.71590110	-0.00012468	-0.00670098	-0.00397044
O	9.90590722	7.56861313	6.63760050	0.00558369	-0.01297545	-0.00498026
H	10.18349057	7.55225532	5.66496502	-0.00920113	-0.00388190	0.00205706
H	6.21182892	-0.39012370	8.57124261	-0.00235006	-0.00045832	-0.00682944
O	6.22826263	0.12899658	7.66776752	-0.00549793	-0.00329464	-0.00373088
H	6.46507183	-0.60212843	7.02818581	-0.00089951	0.00293311	-0.01542771
H	8.77288276	2.75246295	6.29198808	-0.01113152	0.01015181	-0.01467270
O	8.71987245	3.74189386	6.08413977	0.00214921	-0.00027448	-0.00255427
H	8.47374275	4.16834199	6.95724391	-0.00516753	-0.00190505	-0.03164798
H	7.63211644	0.70121387	5.24466396	-0.00856701	0.01499763	-0.00633080
O	7.25688992	0.48737157	4.30210795	-0.00103369	-0.01205790	-0.01432055
H	6.28008944	0.82520657	4.25509821	0.00071973	0.03483368	0.00060198
H	2.68987919	6.14701789	5.62208201	-0.00996893	0.00124108	-0.00379289
O	3.22459459	6.79317813	5.03689935	0.00751451	-0.01401040	-0.00779462
H	6.87149781	5.01227125	8.56076561	-0.00639355	-0.00027365	-0.00104141
O	7.79242276	4.65035945	8.65591137	-0.01440716	0.00723497	-0.00077813
H	8.27258461	5.43125256	9.12342120	-0.00707419	0.00236853	-0.00865380
H	5.47703036	8.04444296	10.27210869	0.00611890	0.00147082	0.00604461
O	6.20565486	7.40147559	9.96139558	-0.00243180	0.00414301	0.01511187
H	5.66613447	6.51803502	9.98811259	-0.00227658	0.00361208	0.00469022
H	3.41432935	8.01969211	9.64946271	0.00249284	0.00285499	0.00472334
O	3.98758720	8.75042504	10.08779082	-0.00049823	-0.00226455	0.00739159

H	3.61308770	8.83093689	11.00470508	-0.00272044	-0.00846090	-0.01425762
H	2.51357181	9.54612034	6.44046900	0.00258184	-0.00177300	-0.00206927
O	1.75457369	10.11206579	6.06835879	-0.00327852	-0.00616140	-0.00332496
H	1.28636225	9.50258549	5.44023850	0.00416247	0.00786588	-0.00081690
O	9.49073900	2.10833874	3.90060892	0.00067284	-0.00173223	-0.01293894
O	10.26525699	5.04054634	4.27268689	-0.00762293	0.00373202	-0.01877461
H	8.74319683	1.49031710	3.65840791	-0.00225206	-0.01076909	-0.00744514
H	10.05243155	2.18251521	3.08814593	0.00456395	-0.00509647	0.01232308
H	10.44138120	5.02184032	5.26775188	0.00890745	0.00755551	-0.00804055
H	10.47702266	5.99630054	4.00536863	-0.01096217	-0.01562933	-0.01089332
O	2.52180486	6.93269099	8.84123854	0.00833097	-0.00972746	0.00273437
H	2.95425873	7.42461133	8.04021873	0.00859548	-0.00345840	-0.00496881
H	1.72801297	7.57128326	9.03805228	0.00568540	-0.00386336	0.00344683
O	4.55998346	5.43649501	10.19766566	-0.00028409	0.00275870	0.01072505
H	3.73581387	5.74902426	9.72773480	0.00349961	-0.00351669	0.00466947
H	4.53657651	4.43391528	10.20084970	0.00323762	0.00841454	0.01054399
H	6.60221041	4.47703340	6.62735417	-0.00697868	-0.01217912	-0.01422698
H	7.69646065	6.12713782	5.35305911	-0.00703556	-0.00150118	0.00650759

*CH_2O_2;*OH (oxidated)

116

Lattice="9.84 0.0 0.0 0.0 8.52169 0.0 0.0 0.0 11.75" Properties=species:S:1:pos:R:3:forces:R:3

C	1.31548428	0.71684023	0.89682088	0.00061942	-0.00111073	-0.00059861
C	1.32477720	2.14375139	0.83285303	0.00290013	-0.00368834	0.00186936
C	2.55875124	2.85669549	0.94769930	0.00182253	-0.00289879	-0.00701412
C	2.56189991	4.28120103	1.00751843	0.00564536	0.00505863	-0.00187486
C	1.32897810	4.98825465	0.92439621	0.00409620	0.00281329	0.00699253
C	1.32561326	6.40329571	1.01268322	0.00208152	0.00232474	0.00346588
C	2.55855248	7.11321545	1.15616639	0.00144153	-0.00111371	0.00867073
C	2.54298606	8.53493479	1.08579576	0.00151495	-0.00132989	0.00981861
C	3.77324410	0.72733615	1.18376795	0.00369453	0.00387574	0.02135117
C	3.78090531	2.14555069	1.12412899	0.00773950	0.00331797	-0.00792477
C	4.99095325	2.84470448	1.45018071	0.02324442	-0.00651659	-0.03955576
C	4.86816668	4.20941474	1.84714286	0.01428677	-0.00135315	0.00803810
C	3.74272200	4.97668381	1.42070806	0.00255890	0.00992915	-0.02782999
C	3.77142618	6.41380163	1.45980311	0.00690373	-0.00116872	-0.01242324
C	4.99980894	7.15265636	1.63529763	-0.00887694	0.00459660	-0.02363090
C	4.99998083	8.54388129	1.36198646	0.00793498	-0.00681812	0.01130382
C	6.24233075	0.73744903	1.30952446	-0.00434850	0.00609049	0.00501421
C	6.25713713	2.14673252	1.42507501	0.00794527	0.00636350	0.00061059
C	7.52380982	2.83542540	1.42037788	-0.02168127	-0.00535661	0.00490462
C	7.70798637	4.15898662	1.96030898	-0.00808488	0.00293036	0.01928950
C	6.26668719	6.58442277	2.09962170	0.00580051	-0.01016322	0.01503395
C	7.48330643	7.13417795	1.52160400	0.01067192	-0.00640464	-0.02398469
C	7.46819101	8.52755720	1.20400966	-0.00244084	-0.00183714	-0.00646979

C	8.67841690	0.70986646	0.94262263	-0.00118932	-0.00272415	-0.00277684
C	8.68554697	2.13848248	0.96431044	-0.00964431	0.00317510	0.00312097
C	9.92808949	2.85017139	0.85735498	-0.00265422	0.00020597	-0.00103850
C	9.93317555	4.27363379	0.95143764	-0.00688734	0.00234340	-0.00015248
C	8.75296041	4.95803604	1.38247325	-0.00692213	0.01496297	-0.01568429
C	8.70601897	6.39434530	1.34175474	-0.01003859	-0.00220181	0.00769960
C	9.91762059	7.10567148	1.04125898	0.00200522	0.00352391	0.00911832
C	9.91757491	8.52616804	0.91609361	-0.00486121	-0.00063328	-0.00403308
Cu	6.37434931	3.42480911	5.01440345	0.04452013	-0.02701719	-0.00137552
Cu	4.41248090	5.85593111	4.10410732	-0.04601133	0.03340124	0.04582532
Cu	6.71344756	6.99468140	4.06271206	-0.00367745	0.02999608	0.08326816
Cu	4.31245146	3.46186383	3.67828029	0.03400489	0.02887366	0.10478114
Cu	6.25975785	4.87922561	2.97991286	0.00424982	-0.00266039	0.08203609
Cu	8.38877534	4.10091820	3.91133966	-0.02276017	-0.01596258	0.06081137
C	6.01159842	5.87516827	6.49160762	-0.00247315	0.00800629	0.01195684
O	5.85841660	5.74327729	7.84792111	-0.04804126	0.06232603	-0.00861993
O	6.88583460	6.92295665	6.04757154	0.00766225	0.00791545	-0.01310128
H	4.12553328	3.47783588	8.53162183	0.00657926	0.00476277	-0.01536298
O	3.97787394	4.03201809	7.65398202	-0.00038794	0.00836402	-0.01125959
H	4.72729803	4.79551187	7.79758023	-0.01582833	-0.01194051	-0.01107013
H	0.94643618	8.20197839	3.29800618	0.00038620	0.00274462	0.01173680
O	0.91559465	7.69279568	4.14010948	-0.01163795	0.01022820	-0.02652884
H	1.93934702	7.37482597	4.41601822	0.01820104	-0.00599513	-0.01231325
H	2.19778940	2.48741702	4.95777625	-0.00531788	0.00604442	-0.00559267
O	2.42034513	3.05419192	4.10306821	0.01034873	0.00161519	-0.02198054
H	1.78691637	3.83666631	4.10207276	-0.01634149	0.00928310	-0.00873166
H	7.59928770	3.20221812	9.24729254	-0.00878082	-0.00636114	-0.00475678
O	7.24749154	2.33577634	9.68592141	-0.00466860	-0.01735051	0.00311081
H	7.60231121	2.36207119	10.60712123	-0.00389282	-0.00425537	-0.00042818
H	3.53639506	-0.79992175	6.18804091	0.01489862	-0.00904227	-0.00661829
O	3.59797475	-0.17938614	7.01059335	0.01161745	-0.00046858	-0.00319295
H	4.57967671	-0.05033627	7.26514278	0.00218683	-0.00667108	-0.00944570
H	1.39974636	5.78584520	7.34799056	-0.00534099	0.00141928	-0.01002962
O	1.75914980	5.17499025	6.65374955	0.00238575	0.00533550	-0.00500139
H	2.51832435	4.66961567	7.11488227	0.00994666	0.00209964	-0.01961601
H	5.68104365	1.74326032	6.99912961	-0.00000914	0.00636349	0.00156390
O	5.25507888	2.37482890	6.33517076	0.01699057	-0.00068269	0.00844192
H	4.63966945	3.11688761	6.89750619	-0.00946615	-0.00221190	-0.00487677
H	8.98556898	8.77161274	6.66268908	-0.00888616	0.00101679	-0.01091735
O	8.45875637	9.66931594	6.63277919	-0.00393667	-0.00013148	0.00034129
H	7.70766421	9.51478652	7.28573303	-0.00103848	0.00468574	-0.00597878
H	4.19062766	8.69150718	4.25555875	0.01470170	0.00430056	-0.01271789
O	4.76258463	9.50101816	4.25201995	-0.01817484	-0.01625835	-0.01584849
H	4.73634747	9.88029512	5.20168188	-0.00624411	-0.03627745	0.00004203
H	5.68432249	2.58122244	9.72258901	-0.00344607	-0.00734938	0.00079460

O	4.66117039	2.75437631	9.75670449	0.00526919	-0.00860048	0.01548347
H	4.26316549	1.82124766	9.80704227	0.00760456	-0.00339238	0.00224041
H	7.76855074	7.12763642	9.96228305	-0.00091195	0.00543223	0.00491559
O	8.72882575	6.80224712	9.77413051	-0.00168625	0.00384993	0.00723283
H	9.07949569	6.60045443	10.67681677	-0.00215453	-0.00007501	0.00152343
H	-0.14862422	-0.41104455	9.33149667	-0.00292748	0.00480796	-0.00117946
O	0.65243782	0.20798086	9.24006982	-0.00125039	0.00613210	0.01000490
H	0.53011371	0.67463562	8.37758010	-0.00022355	-0.00357449	-0.00533469
H	8.56015319	6.95625258	6.37194126	-0.00877123	-0.01355300	-0.01420921
O	9.50380788	7.32242378	6.51365451	0.00751656	-0.01251559	0.00176368
H	9.86016133	7.44598599	5.58268626	-0.01515880	-0.00313029	-0.00230223
H	6.21084014	-0.29603007	8.53841058	-0.00136271	0.00048835	-0.00817045
O	6.14382561	0.20668517	7.65279225	-0.00590713	-0.00090799	0.00529601
H	6.46993329	-0.52140100	6.94508163	-0.00395056	-0.00276697	-0.01558196
H	8.72558385	2.74487131	6.23326368	0.00376681	-0.00006090	-0.00005784
O	8.49122928	3.70665025	6.01603320	-0.00224512	0.00301192	-0.01497126
H	8.30860310	4.14007676	6.95677191	-0.00505748	-0.00029421	-0.02961575
H	7.71540263	0.74125665	5.20632926	-0.01073707	0.01940841	-0.01230263
O	7.42370191	0.54778576	4.23506388	-0.00049552	-0.01441949	-0.01765507
H	6.45139159	0.84119131	4.17653330	0.00179324	0.02841777	-0.00775050
H	2.57782581	6.16020714	5.64173503	-0.00702152	0.00390185	0.00519460
O	3.03153632	6.83457430	5.00975819	0.00671394	-0.01160261	-0.01536577
H	6.97000681	5.06233760	8.22198772	0.04500098	-0.03082856	0.01524979
O	7.92694547	4.51545905	8.40000092	-0.00822045	0.00655288	-0.00283580
H	8.45530351	5.23372726	8.86247825	-0.00566321	0.00403433	-0.01023913
H	5.50425525	8.07642333	10.32123539	0.00599004	0.00103137	0.00708358
O	6.24386094	7.42864722	10.07641524	-0.00283375	0.00573616	0.01425584
H	5.71037776	6.55487470	10.05278621	-0.00097721	0.00433716	0.01128804
H	3.40969292	8.02965960	9.68478332	0.00279091	0.00318891	0.00521178
O	3.99170838	8.77579006	10.11937808	0.00074219	-0.00184202	0.01044322
H	3.60538585	8.86273725	11.02385570	-0.00270278	-0.00833854	-0.01256243
H	2.53039838	9.49462297	6.46175013	0.00354153	-0.00198321	-0.00388505
O	1.77232814	10.04838863	6.06204870	-0.00532571	-0.00637152	-0.00476833
H	1.27937613	9.40786957	5.48281960	0.00120487	0.00743421	-0.00033850
O	9.53375528	2.15506843	3.91233755	0.00385940	-0.00211822	-0.01511432
O	10.32266970	5.01362076	4.23870167	-0.00683780	0.00078130	-0.01970316
H	8.79378433	1.49751724	3.72273779	-0.00404891	-0.01393676	-0.00546825
H	10.13241510	2.16103677	3.12741803	0.00262659	-0.00581164	0.01235676
H	10.49516745	4.97367785	5.23336546	0.00721972	0.00320132	-0.00674606
H	10.44340202	5.99855431	4.01194555	-0.00284848	-0.01165294	-0.01244631
O	2.54516812	6.96524934	8.96667705	0.01064548	-0.01060289	0.00396547
H	2.98017342	7.35643108	8.12300066	0.01030752	-0.00462926	-0.00226884
H	1.73127379	7.60585978	9.06106267	0.00633171	-0.00604521	0.00175918
O	4.49943396	5.45593822	10.40175865	0.00006472	0.00231944	0.01085832
H	3.71541786	5.76996590	9.87025501	0.00268680	-0.00416060	0.00634718

H	4.53258733	4.46345290	10.28121316	0.00296931	0.00830821	0.01174862
H	6.42870288	4.92422908	6.01878696	-0.01103227	-0.00526166	-0.04478696
H	4.99734106	6.02360215	6.01975014	0.00410981	-0.01823835	-0.00921844

*CH$_2$OH;*OH

119

Lattice="9.84 0.0 0.0 0.0 8.52169 0.0 0.0 0.0 11.75" Properties=species:S:1:pos:R:3:forces:R:3

C	1.23650078	0.66967838	0.78133776	-0.00298994	-0.00432199	-0.00258425
C	1.24218134	2.09497521	0.71424220	0.00133603	-0.00548189	-0.00263141
C	2.45926585	2.80836076	0.92369972	0.00357045	0.00227752	0.00300616
C	2.46270882	4.23016038	0.99552215	0.00939400	0.00476960	0.01733289
C	1.25371483	4.93960412	0.76682101	-0.00334215	0.00652580	0.00087421
C	1.24734446	6.35503835	0.85948043	0.00203367	0.00333171	0.00198483
C	2.47354122	7.05467181	1.06805851	0.00174771	-0.00449930	0.00745462
C	2.45491762	8.48064846	1.00146104	0.00027450	-0.00680218	0.00868548
C	3.68021405	0.67578501	1.11382808	0.00054176	-0.00604061	0.01804968
C	3.67976355	2.09469571	1.09348893	0.00550905	0.00456518	-0.00591746
C	4.88603737	2.79047109	1.40323907	0.01741204	-0.00847544	-0.04570368
C	4.74854180	4.13053767	1.87573993	0.01416940	0.00512038	0.00989364
C	3.63276573	4.91473498	1.44739218	-0.00105399	0.02044569	0.00284584
C	3.67980160	6.35274595	1.38952368	0.01129360	-0.00411360	0.00507059
C	4.92074408	7.09267768	1.44994889	-0.00766258	-0.00700108	-0.00760929
C	4.91287121	8.49235728	1.21796362	0.01258764	-0.01473870	0.01612064
C	6.14692648	0.69408292	1.10784948	0.00142320	-0.00774833	-0.00359285
C	6.15499104	2.10322761	1.25589420	0.00736981	0.00723113	0.00055172
C	7.41620678	2.79594398	1.12359848	-0.01172788	0.00579197	-0.00022420
C	7.56914915	4.15379446	1.51386863	-0.01090354	-0.00148104	0.00885679
C	6.19932900	6.50932999	1.73874984	-0.00163541	-0.01045011	0.00260773
C	7.38863328	7.09874767	1.18397964	-0.00023057	-0.00635424	-0.04962247
C	7.37324615	8.49920701	0.91703296	-0.00683643	-0.00202032	-0.02109677
C	8.59862557	0.67412016	0.71207104	-0.00246314	-0.00739328	0.00140420
C	8.60050526	2.09614365	0.71533450	-0.00881677	0.00246341	0.00074675
C	9.84717983	2.80271792	0.61741596	-0.00878238	0.00235446	-0.01812129
C	9.86109923	4.22610089	0.66064884	-0.00984938	0.00413596	-0.02286956
C	8.65135763	4.92131038	0.99338837	-0.01085556	0.00232534	-0.01710546
C	8.61856406	6.36200744	0.99773385	-0.00777904	0.00210170	-0.02135445
C	9.84419791	7.06819086	0.82048675	-0.00700434	-0.00587763	0.01263130
C	9.83881028	8.49028207	0.73525799	-0.00380003	-0.00111990	0.00045764
Cu	6.91432263	4.57734895	4.98463766	0.00723378	0.00207469	-0.00898840
Cu	4.92278713	5.85849616	4.66942348	-0.00782674	-0.02279775	0.02769977
Cu	7.27962343	6.82496379	3.58022138	0.05243757	0.00327428	0.08647348
Cu	4.68808018	3.55649573	3.84667091	0.00852264	0.02280296	0.04036213
Cu	6.25782196	4.89167718	2.70045195	0.01456606	0.00392624	0.05946144
Cu	8.58779034	4.47169907	3.24674854	-0.04882982	-0.02042347	0.06637515
C	6.21719425	6.20850071	6.15931496	-0.00776176	-0.00668268	-0.00378945

O	5.94726938	4.62443987	8.44277096	0.00096137	-0.00166831	-0.00355257
O	6.86436826	7.50889572	5.90265570	0.00329184	0.01403551	-0.00006441
H	3.33787815	3.49681668	8.05569694	-0.00300626	0.00626447	-0.01216400
O	3.49838854	4.25023652	7.43153366	0.01674196	0.01253320	-0.00062534
H	5.05377328	4.53491486	7.98613854	0.00834380	0.01301469	-0.01543790
H	0.83682405	8.28967414	3.23227845	-0.01012984	-0.00537131	0.00292308
O	0.99119220	7.92841586	4.13978280	-0.01093199	0.00667002	-0.01973810
H	1.94275197	7.45047615	4.16156769	0.00977510	-0.00176719	-0.01183844
H	2.60384123	2.33564184	5.33784109	0.01162017	0.01137178	-0.01092509
O	3.19725930	3.13706804	5.03634699	0.00466877	0.00248393	-0.01923201
H	3.38541703	3.58145484	5.93050257	0.00191650	0.01452720	0.00541549
H	7.86980290	2.75562313	9.36032528	-0.00450618	-0.00514879	0.00050597
O	7.22963941	2.08573171	9.74912287	-0.00186984	-0.01182983	0.00640394
H	7.28421072	2.30212444	10.71554402	-0.00531811	0.00410895	-0.00636930
H	3.63269917	-1.11963436	6.08281752	-0.00445300	0.01479386	0.01401836
O	3.47864490	-0.55541294	6.88617115	0.00133699	-0.00337920	-0.00833413
H	4.41447839	-0.36989885	7.33397651	0.00186765	-0.00049061	-0.00855165
H	0.87340090	6.16091047	6.75749245	0.00172524	0.00312554	-0.00171011
O	1.52239195	5.46060751	6.45083991	-0.00443819	0.00743101	0.00046316
H	2.60857180	4.76383222	7.27084228	0.01928795	0.00442364	-0.00720185
H	5.22160687	2.17468449	7.56896231	0.00253703	0.00666238	-0.01675877
O	5.67966560	2.12074846	6.62831077	-0.00068830	0.00066226	0.00435377
H	5.68745382	3.04806477	6.24893321	-0.00257383	-0.00310725	0.01879029
H	8.65543806	9.20670708	6.71175038	-0.01381730	0.00705988	-0.00290406
O	8.22247435	9.95313382	6.21121932	-0.00503160	-0.00415020	0.00142865
H	7.29507586	10.13604952	6.59176339	-0.00381554	0.00283472	-0.00420543
H	4.36512417	8.84066859	4.23896789	0.00529744	-0.00903050	-0.00865633
O	5.25862428	9.12214095	4.54679213	-0.01735025	-0.00046232	-0.00682098
H	5.14003196	9.75808685	5.34326205	0.00340903	-0.00484625	-0.00731950
H	5.67135498	2.32492210	9.35633993	-0.00550729	-0.00538326	0.00115374
O	4.68542296	2.34066114	9.03829729	0.00184029	-0.01325889	0.01117709
H	4.34300750	1.43640111	9.35763870	0.00338050	0.00330406	0.00265881
H	7.50744062	6.81493794	10.00162540	0.00124402	0.00433953	0.01029560
O	8.49252627	6.74907624	9.75802426	-0.00843513	0.00283934	0.00506017
H	8.89873867	6.62537872	10.65922961	-0.00348085	-0.00064123	0.00111748
H	-0.29604648	-0.45094853	9.28303945	-0.00466808	0.00361656	0.00138532
O	0.57426407	0.05512468	9.22293708	-0.00110536	0.00475395	0.01134256
H	0.58311276	0.42692383	8.30706168	0.00294361	-0.00089214	-0.00249968
H	8.58571922	7.27643573	6.30430154	-0.00379286	-0.01422515	-0.01985510
O	9.52062658	7.53544611	6.55454447	-0.00083702	-0.01483471	-0.00672788
H	9.91464376	7.76226712	5.65965777	-0.01300632	-0.00751059	-0.00817867
H	5.84933938	-0.84007604	8.74860629	0.00009346	0.00699212	-0.00753160
O	5.73795180	-0.13585664	8.04825351	0.01378007	-0.00393663	0.00412716
H	6.38799192	-0.38060255	7.31947573	0.00105333	-0.00003258	0.00058316
H	8.81263363	3.05661963	5.97241672	0.01113738	0.00238638	-0.01333196

O	8.69843488	4.05471985	6.01602933	-0.01351350	0.00501573	-0.00207942
H	8.71500388	4.22442454	7.03413880	-0.00159205	0.00247096	-0.01805181
H	8.00457306	0.75632867	4.77124884	-0.00153083	0.00752968	-0.01888982
O	7.93360043	0.42429977	3.79216261	0.00072537	-0.00824254	-0.01997914
H	7.07401312	0.83496034	3.49691296	0.00060682	0.03864104	0.00239584
H	2.12464820	5.92009576	5.75699995	0.01581577	-0.00122746	-0.00076920
O	3.03899379	6.48765436	4.48863884	0.01344617	0.00037705	-0.01373801
H	2.69937626	5.76266708	3.89709445	-0.00007189	-0.01201499	-0.01551775
H	7.46392523	4.35240810	8.47070483	-0.00982209	-0.00153171	-0.00809087
O	8.47714152	4.27024662	8.59118811	-0.00520829	0.00779302	0.00294960
H	8.67708646	5.14502168	9.05851398	-0.00331677	-0.00003627	-0.00206061
H	5.21728528	7.51299417	10.38488557	0.00340843	-0.00260789	0.00442482
O	5.83286340	6.71055401	10.21601005	0.00220480	0.00182633	0.01855146
H	5.18108114	5.92232899	10.43899960	0.00377880	0.00739989	0.01439537
H	3.31786448	7.83648516	9.70079064	0.00354376	-0.00211780	0.00779826
O	3.97356034	8.55131095	10.08709316	-0.00335643	-0.00064714	0.01655607
H	3.56871997	8.81315083	10.95257471	-0.00180962	-0.00692098	-0.01342808
H	2.44119055	9.15968550	6.41529386	0.00322536	-0.00600794	0.00062216
O	1.75767224	9.83825614	6.08295440	-0.00748046	-0.00893533	-0.00094048
H	1.32158728	9.36351255	5.31972801	-0.00329320	0.00236459	-0.00007645
O	9.73824119	2.49277551	3.64848861	0.00050012	-0.00343122	-0.01091825
O	10.33266760	5.21521472	3.93011646	-0.00721270	-0.00162554	-0.02080963
H	9.09250227	1.74163807	3.47609600	-0.00475115	0.00464182	-0.00547602
H	10.33254350	2.47965378	2.85704109	0.00723718	-0.00831228	0.01362259
H	10.39581159	4.94090960	4.89990706	0.01252387	0.00520920	-0.00373719
H	10.30346855	6.21906583	3.97047536	-0.01032180	0.00606819	-0.00501838
O	2.43744803	6.76975165	9.04867372	0.00827273	-0.01398870	0.00055731
H	2.77586953	7.01607636	8.12766557	0.00742284	-0.00410146	0.00031035
H	1.61048384	7.39122208	9.13420068	0.00640159	-0.00859788	0.00443161
O	3.99709909	4.87554996	10.41196221	-0.00006741	-0.00036471	0.00563286
H	3.32279319	5.33537416	9.83313591	0.00478600	0.00089615	0.00642966
H	4.24950853	4.02433206	9.95370638	-0.00517168	0.00297129	0.02273055
H	5.37883484	6.35409892	6.88007243	-0.00119732	-0.02001353	0.00088124
H	5.88402682	5.50244935	8.90617694	-0.00502609	-0.00701634	-0.00910906
H	6.96186134	5.65156404	6.76947683	-0.00581992	0.01151813	-0.01419173
H	6.21194069	8.06693077	5.33574833	-0.00409823	0.01305611	0.02018751

*CH_2O

119

Lattice="9.84 0.0 0.0 0.0 8.52169 0.0 0.0 0.0 11.75" Properties=species:S:1:pos:R:3:forces:R:3

C	1.07262329	0.70011100	0.78192683	-0.00114801	-0.00254834	-0.00425002
C	1.07825278	2.12194273	0.68952382	0.00165026	-0.00652497	-0.00346995
C	2.30330358	2.83410920	0.87512400	0.00601651	0.00171242	0.00165194
C	2.30411210	4.25661030	0.92178185	0.01311735	0.00520049	0.01150577
C	1.09132768	4.96834938	0.71264815	-0.00307119	0.00630916	-0.00100159

C	1.08459097	6.38412427	0.83365674	0.00467310	0.00260897	0.00416886
C	2.30801062	7.08648072	1.04747563	0.00315107	-0.00506929	0.00598524
C	2.29332817	8.51338282	0.99949957	0.00294880	-0.00474629	0.00410092
C	3.52040536	0.70481868	1.14012251	0.00039609	-0.00418801	0.01930509
C	3.52032948	2.12459159	1.09508722	0.00664877	0.00482742	-0.00301746
C	4.72315629	2.82797524	1.42038389	0.01443127	-0.00776857	-0.04003130
C	4.56306502	4.17333434	1.86110073	0.01478964	0.00682323	0.01048840
C	3.46792335	4.94228579	1.39283986	0.00278607	0.02012069	-0.00285278
C	3.51106311	6.38166464	1.35386833	0.01517723	-0.00455272	0.00996888
C	4.75123948	7.10838130	1.45566991	-0.00503337	-0.00848067	-0.00431150
C	4.75257652	8.51925273	1.27660363	0.00946130	-0.00949203	0.01794398
C	5.99069344	0.72128420	1.18762661	0.00350418	-0.00670701	-0.00221946
C	5.99703586	2.13643093	1.29953139	0.00696969	0.00555286	-0.00027702
C	7.25733041	2.82434981	1.15048567	-0.01300645	0.00569413	0.00055422
C	7.43070449	4.18627479	1.52937351	-0.01090092	-0.00067012	0.00836580
C	6.02015207	6.48908822	1.71393961	-0.00112707	-0.00900503	0.00012392
C	7.23724737	7.10986370	1.25850069	0.00242443	-0.00474675	-0.01300329
C	7.21798457	8.52627233	1.02402116	-0.00710529	-0.00001384	-0.01298057
C	8.43510834	0.69862553	0.77585544	-0.00686594	-0.01135818	0.00307170
C	8.43785835	2.12096905	0.74156629	-0.00906035	0.00319121	0.00401224
C	9.68280644	2.82924117	0.59976976	-0.01013634	0.00233718	-0.01716589
C	9.69835437	4.25308674	0.62065033	-0.01181752	0.00355963	-0.02294610
C	8.49597495	4.94970816	0.98355348	-0.01470072	0.00785629	-0.01417567
C	8.46606332	6.38725278	1.02490257	-0.01028567	0.00168878	-0.02000075
C	9.68177423	7.09634828	0.83959907	-0.01393358	-0.00627270	0.01327279
C	9.67622707	8.51821080	0.76530994	-0.00539354	-0.00026470	0.00172078
Cu	6.81502858	4.15721510	4.95387038	-0.00749868	-0.04127133	-0.01899139
Cu	5.08765440	5.85379498	4.77443153	0.00169808	-0.00982953	0.00827503
Cu	6.92133175	6.91285065	3.53142625	0.01135308	0.04343027	0.06212165
Cu	4.60051337	3.53508345	3.93521811	0.03157078	0.01865891	0.02641574
Cu	6.08520503	4.88200489	2.71679520	0.01372434	0.00913146	0.05832832
Cu	8.43725092	4.54275149	3.22705899	-0.03761954	-0.00812912	0.06002126
C	6.51040628	7.12230823	5.55559750	0.00181009	0.01274770	-0.01797941
O	5.61800367	4.90609350	7.81397510	-0.00707245	0.00762902	0.01451139
O	7.09438054	6.54794189	6.72379467	0.02870824	0.00813728	-0.01015015
H	3.41046116	3.25596904	8.02833318	0.00199192	0.00327333	-0.01252266
O	3.21767816	4.02867841	7.42308811	0.01589297	0.00650959	0.00029804
H	4.14608082	4.47009858	7.41833449	0.01357532	0.01144213	-0.02215562
H	0.82350758	8.36550953	3.18287509	-0.00945380	-0.00275003	0.00637184
O	0.96515289	7.95686621	4.07554400	-0.00693004	0.00903608	-0.02213518
H	1.84081284	7.43181943	4.03371924	0.00799539	-0.00219853	-0.01089479
H	2.57575273	2.22027200	5.23394248	0.00521346	0.00911841	-0.00734542
O	2.91841909	3.14563876	4.94374552	0.00638976	0.00905516	-0.02255011
H	3.04431788	3.54018433	5.87624982	0.00056352	0.00519580	0.00127281
H	7.62107522	3.01353574	9.38749240	-0.00696669	-0.00256156	-0.00059703

O	7.07558279	2.25419692	9.78318975	-0.00211511	-0.01118004	0.00926981
H	7.14285091	2.42398521	10.75799723	-0.00641172	0.00294134	-0.00431913
H	4.11072569	-1.32209780	6.34099214	-0.00076070	0.00511919	0.01612834
O	3.80620304	-0.71076220	7.06264731	-0.00225933	-0.00599403	-0.00434622
H	4.74055393	-0.43406997	7.55599671	0.00294453	-0.00254118	-0.00170506
H	0.77277142	6.25644084	6.84386378	0.00331286	0.00479537	0.00262352
O	1.38552886	5.55221980	6.43978451	-0.00102521	0.00597940	0.00639906
H	1.89299485	5.02277269	7.12984014	-0.00153300	0.01159878	-0.00718664
H	5.17257834	2.09222181	7.57525194	0.00552541	0.00615629	-0.01938810
O	5.62291864	2.05902651	6.65539957	-0.00216112	-0.00744825	0.00863642
H	5.65825823	3.01655825	6.35383949	-0.00163972	-0.00245775	0.00868106
H	8.64426621	9.01713203	6.86589732	-0.01038263	0.00354338	-0.00103245
O	8.22395261	9.83739687	6.46451060	-0.00989633	-0.01257694	0.00073150
H	7.28632518	9.93860675	6.82606840	-0.00339524	-0.00028573	-0.00643877
H	4.44700454	9.06675820	4.04701983	0.00673040	-0.00518831	-0.00303950
O	5.16039594	9.72259414	4.23003441	-0.00406436	-0.00637108	-0.01329845
H	5.16139003	9.89191006	5.23806665	0.00974826	-0.01841954	0.00414555
H	5.62073079	2.34970926	9.40673962	-0.00366345	-0.00383263	-0.00007184
O	4.60415314	2.35671670	9.11640191	0.00288463	-0.00628319	0.00627052
H	4.27366176	1.45330201	9.46034693	0.00460045	-0.00238999	0.00327567
H	7.71480131	6.97409494	10.03629557	0.00080505	0.00290905	0.00437820
O	8.66326914	6.79848622	9.71826261	-0.00507745	0.00343869	0.00513977
H	9.13200565	6.63629643	10.57796656	-0.00295295	-0.00082835	-0.00162088
H	-0.08742106	-0.31924294	9.23199285	-0.00444382	0.00254380	-0.00006585
O	0.79290458	0.16290841	9.19556075	0.00006684	0.00060388	0.01183588
H	0.85474155	0.48374894	8.25924556	0.00150226	-0.00523525	-0.00229641
H	8.48833890	6.93158750	6.73957591	0.02889324	-0.00805032	-0.00995986
O	9.44593775	7.41611558	6.74873839	0.00349147	-0.00438165	-0.00647682
H	9.64446226	7.62032039	5.80050588	-0.01571723	-0.00830114	-0.00184637
H	6.00861142	-0.60574033	9.03601912	0.00215330	0.00264283	-0.01052641
O	6.02542589	-0.16431693	8.13823196	0.00510557	0.00047376	0.01641098
H	6.58675991	-0.86812663	7.62196509	-0.01009175	0.01356803	0.01381551
H	8.66582796	2.93264608	6.19123056	0.01375150	0.00084307	-0.00483504
O	8.47703136	3.91796408	6.09051600	-0.01504205	0.00778446	-0.00151465
H	8.39600040	4.22517975	7.06687456	0.00093749	0.00854265	-0.00880984
H	8.00335188	0.97452820	4.86720454	-0.01115192	0.00119691	-0.01891045
O	7.88419030	0.81576837	3.86029217	-0.00278309	0.00014084	-0.01950474
H	6.92568056	1.08950167	3.73305321	-0.00275682	0.03169174	-0.00309298
H	2.33303473	5.95776610	5.40387699	0.01769616	-0.00169648	-0.00918830
O	2.94287705	6.22866911	4.55944895	0.00777747	0.00873300	-0.01610095
H	2.70390687	5.51374020	3.90849155	-0.01867802	-0.00594224	-0.01319378
H	7.11140084	4.58413955	8.39915329	-0.01181129	0.00252743	-0.01080116
O	8.09032941	4.41453290	8.62771954	-0.00935263	0.01028027	0.00295978
H	8.40758907	5.28329341	9.05385991	-0.00790020	0.00384899	-0.00347648
H	5.33148497	7.61597893	10.61401529	0.00801198	-0.00259297	-0.00128542

O	6.07268750	6.94821451	10.41334554	-0.00247661	-0.00627710	0.01245687
H	5.57813193	6.07298927	10.47255523	0.00569138	0.00150812	0.01360851
H	3.39113798	7.79391333	9.76210170	0.00339037	-0.00071873	0.00648052
O	3.99334342	8.55000213	10.15967218	-0.00315014	-0.00025300	0.01180516
H	3.52037085	8.81687661	10.98813680	-0.00088562	-0.00626305	-0.01370810
H	2.69822131	8.90147445	6.42193067	0.00518082	-0.00614085	0.00042891
O	1.96171501	9.52774663	6.11039393	0.00190003	-0.01117858	0.00040380
H	1.48840023	9.03069533	5.38405135	-0.00051551	-0.00089879	0.00165030
O	9.67610166	2.71076993	3.68370898	0.00588839	-0.00285967	-0.01370248
O	10.00958729	5.40628195	4.05546874	-0.00436895	0.00091502	-0.01759178
H	8.98750404	1.97207254	3.55185407	-0.00477371	-0.00827761	-0.00442040
H	10.30087366	2.61874538	2.92533290	0.00589728	-0.00539423	0.01191360
H	10.14672678	5.10016582	5.00268640	0.00444156	0.00457178	-0.00157922
H	10.08585123	6.40635184	4.06174435	-0.01055106	0.00209423	-0.00469997
O	2.53920686	6.73078650	9.09176579	0.00430828	-0.01367674	0.00321679
H	2.97211941	6.98688473	8.20149284	0.00603993	-0.00412937	-0.00237931
H	1.74881227	7.39605892	9.13970216	0.00556858	-0.00744294	0.00427098
O	4.35357853	4.89820959	10.18887889	0.00125347	0.00147011	0.02059716
H	3.51086182	5.34565183	9.87906671	-0.00002291	-0.00059143	0.00941564
H	4.27831514	3.91978147	9.95841648	0.00481503	0.00580381	0.01487698
H	6.19072857	5.68836205	7.33185096	-0.04089642	-0.04510851	0.01890923
H	5.25114914	5.20026438	8.70543269	0.00769256	0.00949782	-0.01988181
H	5.88180930	8.00825363	5.81142966	0.01073630	0.02551187	-0.00382407
H	7.38771431	7.66898187	5.05495585	-0.01354032	-0.02376655	-0.01144785

*CHOH

119

Lattice="9.84 0.0 0.0 0.0 8.52169 0.0 0.0 0.0 11.75" Properties=species:S:1:pos:R:3:forces:R:3

C	1.05794478	0.73248919	0.81306897	-0.00120188	-0.00271558	-0.00376017
C	1.06434233	2.15368630	0.72390732	0.00196446	-0.00592537	-0.00265623
C	2.29124208	2.86573684	0.89899196	0.00532374	0.00184847	0.00364091
C	2.29246411	4.28846425	0.94323804	0.01304191	0.00514514	0.01188807
C	1.07602146	5.00011135	0.74942024	-0.00373825	0.00605818	0.00093026
C	1.07016328	6.41597878	0.86721021	0.00511750	0.00258988	0.00449588
C	2.29563288	7.11930216	1.06292753	0.00298283	-0.00579113	0.00560856
C	2.28258136	8.54678524	1.01463185	0.00292322	-0.00428346	0.00367633
C	3.51012176	0.73728390	1.14806842	0.00077275	-0.00338019	0.01832666
C	3.51153350	2.15787149	1.11066989	0.00533794	0.00319280	-0.00579589
C	4.71480388	2.86312489	1.43881721	0.01669945	-0.00948072	-0.05017181
C	4.55835316	4.21410298	1.87703388	0.01378741	0.00637086	0.01175441
C	3.45909642	4.97624416	1.40422870	0.00390200	0.01991323	-0.00477546
C	3.49734289	6.41399276	1.36415607	0.01549444	-0.00503901	0.00943833
C	4.73901795	7.13680327	1.47608455	-0.00593200	-0.00982563	-0.00151950
C	4.74020325	8.54903115	1.29233065	0.00864389	-0.00867836	0.01626435
C	5.97722692	0.75025297	1.20948573	0.00256189	-0.00408606	-0.00358671

C	5.98334075	2.16633605	1.32318175	0.00818572	0.00565997	-0.00032524
C	7.23941767	2.85991701	1.18391450	-0.01398030	0.00481518	0.00059729
C	7.40723424	4.21762075	1.57958632	-0.01032391	0.00249364	0.00896919
C	6.00012674	6.51680169	1.74058926	-0.00326956	-0.01165782	0.00110734
C	7.21985048	7.13759840	1.31498916	0.00398902	-0.00621580	-0.01902801
C	7.20257768	8.55364600	1.05735615	-0.00631746	-0.00001767	-0.01673855
C	8.41940795	0.72981584	0.81211059	-0.00577210	-0.01165491	0.00375511
C	8.42061878	2.15454193	0.77613702	-0.00943970	0.00255738	0.00297233
C	9.66791347	2.86250609	0.64277325	-0.00958995	0.00244160	-0.01609763
C	9.68190490	4.28534665	0.67220428	-0.01129517	0.00398677	-0.02107052
C	8.48115708	4.97859416	1.04947767	-0.01429043	0.01016253	-0.01847441
C	8.45479516	6.41640836	1.09570710	-0.00865142	0.00141326	-0.01746189
C	9.66595936	7.12656757	0.89272075	-0.01348839	-0.00582710	0.01481332
C	9.66127690	8.55007510	0.80687489	-0.00593393	-0.00042796	0.00220329
Cu	6.70968723	3.98805420	4.95132859	-0.01995364	-0.07512794	-0.01681325
Cu	5.00627212	5.74777276	4.89495178	-0.00378055	-0.00816917	-0.00639060
Cu	6.90937845	7.04531465	3.62008602	0.02234219	0.02674536	0.05369771
Cu	4.52516424	3.51541785	3.89398255	0.03385649	0.01792997	0.04402321
Cu	6.06370325	4.94641020	2.79060911	0.00949232	0.00374922	0.05438460
Cu	8.37873360	4.51349330	3.30870131	-0.03712160	-0.02264780	0.06692817
C	6.31927118	6.98088735	5.50695344	-0.00083294	0.01731473	-0.00685083
O	5.52850622	5.01298658	7.93880238	0.00487458	0.00756007	-0.00028940
O	7.41375768	6.57494893	6.45589756	0.01983173	0.00210160	0.01652185
H	3.36740517	3.15795475	8.06332882	0.00141977	0.00312288	-0.01409024
O	3.16131259	3.91037543	7.43818400	0.01655179	0.00438597	-0.00111909
H	4.05791552	4.38760516	7.45460677	0.01418191	0.00921339	-0.02234194
H	0.86624469	8.36246115	3.19032429	-0.00875913	-0.00308999	0.00848744
O	0.99469987	7.95538086	4.08405127	-0.00817630	0.00810599	-0.02163273
H	1.84347986	7.37224479	4.04391130	0.01026302	-0.00344731	-0.01031591
H	2.56048239	2.20901894	5.19803160	0.00100203	0.00683564	-0.00511656
O	2.81129584	3.15858475	4.88148248	0.00482160	0.00807088	-0.02274369
H	2.96321765	3.53983146	5.80784195	0.00244684	0.00519267	0.00084200
H	7.54975791	3.11301859	9.41929348	-0.00762725	-0.00283505	0.00071458
O	7.07355265	2.29175865	9.78153795	-0.00117759	-0.01118571	0.00891940
H	7.18938115	2.37969895	10.76305587	-0.00616939	0.00186183	-0.00495128
H	4.36644454	-1.30284047	6.32989010	-0.00207417	0.00212005	0.00092697
O	3.85281466	-0.74515749	7.03182126	-0.00051119	-0.00062654	-0.00188586
H	4.68731336	-0.40134657	7.54768704	0.00070520	-0.00010965	-0.00275670
H	0.86742048	6.27584328	6.82950109	0.00354474	0.00252661	0.00303534
O	1.35692928	5.47991549	6.43808237	-0.00206684	0.00560919	0.00493454
H	1.86373187	4.96346818	7.13668411	0.00045043	0.01246096	-0.00681755
H	5.15745136	2.07859027	7.62633702	0.00626834	0.00478567	-0.01961700
O	5.58255090	2.01312865	6.69806068	-0.00523231	-0.00628845	0.00576634
H	5.53822471	2.95152833	6.32466793	-0.00126276	0.00625995	0.01092876
H	8.68308355	9.12725132	6.89333029	-0.01228555	0.00288159	-0.00159780

O	8.17487410	9.85430854	6.42993194	-0.00701013	-0.01346265	0.00191400
H	7.25176660	9.95174781	6.83330488	-0.00217460	0.00454442	-0.00853718
H	4.50627210	8.94755918	4.03227336	0.00654583	-0.00672009	-0.00242303
O	5.09117409	9.71680383	4.23565016	-0.01009881	-0.00561248	-0.01311156
H	5.10067248	9.81687538	5.24573862	0.00786385	-0.01073815	0.00026203
H	5.62110898	2.35929718	9.47098408	-0.00343438	-0.00383144	0.00060397
O	4.59557431	2.35671248	9.19086998	0.00234142	-0.00609821	0.00758212
H	4.27892674	1.44336113	9.51814750	0.00451549	-0.00212392	0.00426782
H	7.75606615	7.00730846	10.09285552	0.00097331	0.00370483	0.00729365
O	8.69848034	6.81468916	9.76933323	-0.00335767	0.00424441	0.00620001
H	9.16750513	6.64986472	10.63093732	-0.00310815	-0.00101441	-0.00183807
H	-0.04700635	-0.31040587	9.29305753	-0.00345789	0.00238959	0.00065526
O	0.83407864	0.16430194	9.20791080	0.00167313	0.00129862	0.01216258
H	0.85600380	0.43843790	8.25475888	0.00745246	0.00043910	0.00141051
H	8.77758666	7.17351750	6.53231529	-0.00559876	-0.00861043	-0.01227028
O	9.70039404	7.62603104	6.69755736	0.00132585	-0.01206389	-0.01236158
H	9.98930342	7.88705453	5.78272209	-0.01354016	-0.00503995	-0.00395368
H	6.11923961	-0.72672707	9.03325391	-0.00037116	0.00286823	-0.00904649
O	6.15029224	-0.20812639	8.17143790	0.00166278	-0.00595365	0.00582620
H	6.67591427	-0.83595350	7.60435586	0.00010637	-0.00283251	0.00219394
H	8.59044842	2.91788571	6.20176356	0.01484804	0.00159185	-0.00567082
O	8.39107671	3.91244999	6.20730550	-0.00660021	0.00305962	-0.00486494
H	8.34680463	4.12787488	7.21753036	-0.00811067	0.00308349	-0.01985308
H	7.95497545	0.76087032	4.88974697	-0.00473891	0.00718818	-0.01445327
O	7.81784202	0.60587800	3.88775223	-0.00447644	-0.01092461	-0.01222078
H	6.89153380	0.98746504	3.77614056	-0.00048328	0.04737067	-0.00145233
H	2.32507660	5.90073378	5.33462763	0.01103113	0.00142997	-0.00664344
O	2.87709550	6.19047473	4.48443651	0.01084093	0.01218094	-0.01015088
H	2.61779890	5.49351575	3.82306817	-0.01165381	-0.00431690	-0.01059804
H	7.04600825	4.78712384	8.41811927	-0.00944282	0.00186681	-0.01016469
O	7.99469222	4.50350344	8.67650940	-0.01152816	0.01157438	0.00237531
H	8.40008746	5.33854799	9.10222822	-0.00827651	0.00488761	-0.00429063
H	5.32741080	7.61172431	10.60727494	0.00703136	-0.00263025	-0.00058619
O	6.09367505	6.95853660	10.43077625	-0.00527586	-0.00799779	0.01494925
H	5.65144070	6.07397505	10.56992781	0.00488279	0.00030136	0.01565543
H	3.40878186	7.77097213	9.74910864	0.00383235	-0.00090147	0.00602641
O	4.00093759	8.51648470	10.16760989	0.00049528	-0.00215016	0.00887557
H	3.51892149	8.76682783	10.99839502	-0.00090782	-0.00553443	-0.01249666
H	2.79127915	8.89056654	6.39108995	0.00439339	-0.00520914	-0.00111955
O	2.05777458	9.52002901	6.07414884	0.00321821	-0.01099473	0.00390424
H	1.61669059	9.03342681	5.31993618	0.00149617	-0.00104398	0.00147259
O	9.59075900	2.61855260	3.73265790	0.00559981	-0.00352902	-0.01257928
O	9.99700861	5.37209452	4.06790197	-0.00961627	0.00019800	-0.01686550
H	8.94362604	1.85807609	3.57521361	-0.00623199	-0.00139087	-0.00576078
H	10.21201689	2.58605979	2.96489633	0.00776801	-0.00672351	0.01127094

H	10.20469904	5.08736285	5.00995440	0.00183143	0.00168031	0.00022091
H	10.03549841	6.37098328	4.06845752	-0.01087141	0.00447530	-0.00381992
O	2.54173310	6.70430633	9.04971849	0.00457381	-0.01311605	0.00076435
H	2.98977754	6.95833600	8.16579487	0.00487778	-0.00480062	-0.00488188
H	1.76782282	7.38964610	9.11128946	0.00610442	-0.00727630	0.00336548
O	4.29109881	4.89736919	10.18142565	0.00024491	0.00207124	0.02227607
H	3.42926594	5.34141709	9.91632835	0.00271148	0.00036882	0.00967900
H	4.19295813	3.90349162	10.03009570	0.00489514	0.00714447	0.01396966
H	5.46481939	5.82682390	7.35751681	-0.00487148	-0.01434607	0.02491083
H	5.04840971	5.16180806	8.84605930	0.00655106	0.00888344	-0.01684409
H	6.19312785	8.06729549	5.71218240	-0.00597156	0.01763647	0.01353990
H	7.60524702	5.62053282	6.23916165	-0.00300461	0.02155679	-0.01852955

*CH2OH;*OH (oxidated)

119

Lattice="9.84 0.0 0.0 0.0 8.52169 0.0 0.0 0.0 11.75" Properties=species:S:1:pos:R:3:forces:R:3

C	1.05907206	0.78089955	0.74313692	-0.00010012	-0.00413953	-0.00373249
C	1.05976036	2.21054657	0.67486101	0.00275659	-0.00536963	-0.00135067
C	2.28977944	2.92043836	0.80738046	0.00378428	0.00217377	0.00178029
C	2.29294536	4.34418476	0.90802626	0.00987136	0.00740323	0.01105219
C	1.06457836	5.05479545	0.78713827	0.00184516	0.00530951	0.00540030
C	1.06656891	6.47004327	0.89326862	0.00679276	0.00197192	0.00867353
C	2.30822408	7.17075089	1.02154033	0.00451344	-0.00475840	0.00262462
C	2.28973564	8.59230463	0.90326834	0.00263398	-0.00682207	0.00247655
C	3.51936741	0.78238232	0.96785111	-0.00028942	-0.00576056	0.00829744
C	3.51729348	2.20544733	0.94290475	0.00584063	0.00419127	-0.01389446
C	4.70572158	2.89986001	1.31593838	0.01951264	-0.00463143	-0.04972632
C	4.55260656	4.21392103	1.87165905	0.01220350	0.00056432	0.01848257
C	3.46297796	5.02371702	1.38986902	0.00247076	0.02096088	-0.00132330
C	3.51719560	6.47052770	1.37136286	0.01157663	-0.00006802	0.00902267
C	4.75023857	7.22261213	1.52822853	-0.01593273	-0.00872236	-0.00558910
C	4.74381585	8.60073566	1.17743438	0.01018943	-0.01634477	0.00791600
C	5.97654148	0.80418025	1.10731654	-0.00078980	-0.00358797	0.00519329
C	5.97701064	2.21568848	1.21999419	0.01009954	0.00758591	0.00138147
C	7.23790299	2.90726009	1.17481306	-0.01556609	0.00344680	0.00063859
C	7.39122483	4.24132359	1.66603034	-0.01101038	0.00277513	0.01501235
C	6.02228410	6.67712555	1.97976491	-0.00000481	-0.00894548	0.00546628
C	7.22026091	7.22566136	1.37727059	0.00821914	-0.00987708	-0.03161780
C	7.20692389	8.60566342	1.02122615	-0.00519518	-0.00640450	-0.00609355
C	8.42198093	0.78466091	0.77420538	-0.00012549	-0.00355497	-0.00242418
C	8.41894716	2.20893322	0.76607057	-0.00845490	0.00338563	0.00012696
C	9.66045261	2.92003876	0.66957212	-0.00689497	0.00245105	-0.01386815
C	9.66584826	4.34643727	0.75916390	-0.00862186	0.00422192	-0.01369534
C	8.47339023	5.03567399	1.16638087	-0.01125828	0.01603150	-0.04211302
C	8.44276218	6.47826484	1.19381773	-0.00658135	0.00338854	-0.00978422

C	9.66029849	7.18075705	0.92526504	-0.00120842	-0.00189262	0.01475249
C	9.66043856	8.60019130	0.78531062	-0.00528599	-0.00494938	-0.00024432
Cu	6.49020135	4.29002837	5.13503771	-0.02200361	-0.05823627	-0.03750523
Cu	4.71048393	5.72123598	4.71028200	0.04494398	0.00473773	0.02499983
Cu	6.81080320	6.89590583	3.96008990	-0.03290722	0.04226688	0.07123160
Cu	4.44350413	3.41579461	3.77621570	-0.00619198	0.01253583	0.04991957
Cu	6.02851113	4.96335762	2.79188050	0.00141821	-0.00311351	0.06568486
Cu	8.27628522	4.37214249	3.47120637	-0.04738338	-0.03604336	0.09175548
C	5.16694997	4.83121628	6.65549832	0.00331530	0.01151343	0.00896400
O	6.02946842	5.49089833	7.61080655	-0.00436611	-0.00261220	0.01975772
O	7.68182145	6.82546465	5.80939005	0.00672077	0.01099758	-0.00078842
H	3.08291084	3.24041315	8.34577153	0.00408188	0.00367246	-0.00506912
O	2.35400458	3.90231440	8.09593738	0.01140598	0.00325718	0.00154349
H	2.61000373	4.74962410	8.56524012	0.00645030	-0.00135436	-0.00340002
H	0.86315959	8.58030641	3.02583029	-0.00594840	-0.00313869	0.01284017
O	0.94594906	8.15865598	3.91849475	-0.01056766	0.00275499	-0.01548500
H	1.73811396	7.52949194	3.86264039	0.00795624	-0.00138107	-0.00868883
H	2.44502479	2.25793048	5.42166177	0.01370858	0.01305076	-0.00692488
O	2.88674288	3.13633702	5.10483408	0.00615816	0.00722914	-0.00800959
H	3.01223171	3.61039176	5.96724623	0.00632039	0.00315118	-0.01167839
H	7.30964100	2.99045047	9.45289491	-0.00685792	-0.00152072	0.00024835
O	6.65995539	2.30326812	9.81662163	-0.00210328	-0.00990898	0.01150387
H	6.51602496	2.60991115	10.74731958	-0.00976574	0.00452949	-0.00257676
H	4.06922930	-1.12791950	5.98675307	0.01313588	0.01310281	0.00260417
O	3.89120275	-0.58826923	6.80494952	-0.00685891	0.00390914	-0.00606070
H	4.89987787	-0.44175157	7.19604424	0.00376172	-0.00975092	-0.01135156
H	0.83194645	6.28775988	6.75161691	0.00364459	0.00071814	0.00127929
O	1.33037548	5.50394234	6.28523481	0.00323760	0.00299536	0.00437051
H	1.63543954	4.79670882	6.95458325	-0.00016887	0.00849743	-0.01089722
H	5.09634954	1.86149529	7.29754189	-0.00005260	0.00931056	-0.01647007
O	5.64913284	1.79065465	6.39919262	-0.00141258	-0.00471426	0.00008032
H	5.64702545	2.73492169	6.05089931	0.00444721	-0.00258721	0.00550788
H	8.64744676	9.24839581	7.06989475	-0.01244070	0.00797675	-0.00114812
O	8.24334084	9.88615995	6.42664225	-0.00293415	-0.00878654	-0.00167753
H	7.25554694	9.97143337	6.67534586	-0.00242514	0.01228434	-0.01038214
H	4.50802881	8.89885434	3.74960150	0.00206835	-0.00725932	0.00780644
O	5.01407303	9.69552149	4.04247606	-0.00744326	0.00110680	-0.01187210
H	5.08485436	9.70207330	5.07134851	0.00724629	-0.01676921	0.00174803
H	5.28100137	2.40419600	9.11652130	-0.00358315	0.00066984	-0.00229839
O	4.40122982	2.18293260	8.59061302	-0.00415824	-0.00358471	-0.00060187
H	4.18357103	1.28831859	9.04066498	0.00253926	-0.00312033	-0.00264520
H	8.05972351	6.87611585	9.95564291	0.00241352	0.00223271	0.00497102
O	9.01760594	6.54737460	9.77313225	-0.00089727	0.00234004	0.00394905
H	9.32736937	6.30664786	10.68276619	-0.00420641	0.00425045	-0.00875180
H	0.29145696	-0.76231088	9.46483877	0.00410629	0.00109440	0.00630481

O	1.15550823	-0.22197493	9.47645997	0.00762019	0.00685973	0.01569397
H	1.07361282	0.36418908	8.68742793	0.01017068	0.00242990	0.00642958
H	8.99566352	7.38325883	6.41816367	-0.00484925	-0.00947683	-0.01399643
O	9.82657495	7.57890208	7.02042700	-0.00203785	-0.01904238	-0.01052302
H	10.38849238	8.27301976	6.56131399	-0.00531977	-0.01348418	-0.01242559
H	6.32857544	-0.85809461	8.53174806	-0.00378979	0.01011351	-0.00855983
O	6.23603787	-0.30226626	7.67894897	0.00137550	-0.00102306	0.00684397
H	6.80096388	-0.76945977	6.98989736	-0.01150123	-0.01219534	-0.01640733
H	8.61061187	3.00671450	6.07078370	0.00767596	0.00776578	-0.01462679
O	8.35896127	3.98358745	6.12563258	-0.00593513	0.00455987	-0.00727906
H	8.33679469	4.12249309	7.15317424	-0.00230599	0.00336781	-0.01654530
H	7.91543726	0.70810625	4.89586959	-0.00379116	0.00600521	-0.01138140
O	7.69978848	0.55264773	3.91354984	-0.00036486	-0.00897906	-0.01382043
H	6.78325645	0.96090200	3.80579828	0.00125200	0.03986080	-0.00651557
H	2.21065737	5.94818384	5.22562508	-0.00650826	0.00153348	-0.00233459
O	2.76342774	6.29027022	4.36900433	0.01013544	0.00315848	-0.01517039
H	2.50222139	5.63641225	3.66525886	-0.01033311	-0.01439243	-0.00919049
H	7.16265727	4.71202079	8.40940560	-0.00906363	0.00457278	-0.00974110
O	8.05433541	4.27664568	8.67671412	-0.00603880	0.00694067	0.00191486
H	8.56341221	5.04439144	9.10767137	-0.00420534	0.00316127	-0.00458578
H	5.91053085	7.81445599	10.38371065	0.00324124	0.00177538	0.00924421
O	6.48911050	7.08229174	10.02298678	-0.00382810	0.00223557	0.01774712
H	5.91430468	6.23444307	10.17836130	0.00150834	-0.00009322	0.00566998
H	3.73749054	7.70970680	9.63156979	0.00208747	0.00018425	0.00603345
O	4.21424118	8.53563448	9.98983697	-0.00055895	-0.00315608	0.01137062
H	3.67457825	8.81288549	10.77423319	0.00245405	-0.00719357	-0.01043899
H	2.62757316	8.94408503	6.30045246	0.01165592	-0.00481154	0.00032236
O	1.80078367	9.47460333	6.03820512	0.00488180	-0.00618308	0.00508916
H	1.49264688	9.08023158	5.14672150	0.00049112	-0.00347219	-0.00080946
O	9.53822929	2.54873275	3.73038868	0.00180551	0.00251044	-0.01123900
O	9.91889173	5.48340671	3.99870588	-0.00753318	-0.00242226	-0.01981204
H	8.87341196	1.79219402	3.60523109	-0.00378856	-0.00970398	-0.00636154
H	10.05910514	2.56129829	2.88847733	0.01546613	-0.01245361	0.01084277
H	10.21920791	5.28052649	4.94868038	0.00275800	0.00416745	-0.00323523
H	9.82467208	6.47844076	3.97555731	-0.01804390	0.00618232	-0.00253650
O	2.92579200	6.53187491	8.86040749	0.00812435	-0.00507654	-0.00503901
H	3.27908860	6.92091832	7.98365740	0.00296428	-0.00142397	-0.00461204
H	2.11645375	7.16618999	9.07312244	0.00527620	-0.00707406	0.00015341
O	4.87337537	5.05288558	10.33548454	0.00113450	-0.00841438	0.00622562
H	4.10884508	5.51414765	9.88339354	0.00713168	-0.00275358	0.00616679
H	4.58280756	4.98403664	11.28241230	0.01071410	0.00189228	-0.00107846
H	8.07158480	5.91028655	5.74988299	0.01736674	0.01525724	0.01792082
H	4.80169148	3.90397709	7.14405943	-0.00723295	0.01654452	-0.01086385
H	4.19841028	5.42610184	6.61706033	0.00460341	-0.01067505	-0.00677116
H	6.63998107	6.05082604	7.02750358	-0.00425875	0.00608522	-0.00739655

*CH_2OOH;*OH (oxidated)

117

Lattice="9.84 0.0 0.0 0.0 8.52169 0.0 0.0 0.0 11.75" Properties=species:S:1:pos:R:3:forces:R:3

C	1.28253918	0.68419365	0.85034166	-0.00080516	-0.00199394	0.00087810
C	1.29263134	2.10946212	0.78672390	0.00201939	-0.00387364	0.00141985
C	2.52589635	2.82257316	0.86272162	-0.00245287	-0.00579660	-0.01299990
C	2.52671699	4.24635292	0.90970418	0.00533998	0.00618437	-0.00955330
C	1.29295390	4.95477709	0.84913172	0.00149352	0.00310564	0.00527777
C	1.29160961	6.36787277	0.94178479	0.00302643	0.00315819	0.00288311
C	2.52427492	7.07892175	1.06860567	0.00068466	-0.00026660	0.00870197
C	2.51159284	8.50279214	1.00935637	0.00115090	-0.00306134	0.01041419
C	3.74061991	0.69702421	1.08903930	0.00312260	0.00118576	0.02034138
C	3.75051700	2.11495611	1.03180206	0.01072363	0.00126786	-0.00931656
C	4.95895796	2.81417139	1.35623730	0.03473972	-0.01560550	-0.05196833
C	4.84590985	4.18137388	1.72390823	0.01193882	0.00067855	-0.00279966
C	3.71259553	4.94094096	1.30657485	0.00069091	0.02510161	-0.04511676
C	3.73517749	6.37860725	1.33548923	0.00808378	0.00323128	-0.00881308
C	4.96529254	7.11360823	1.45357255	-0.01127110	-0.00033733	-0.02779898
C	4.96924163	8.51118676	1.22252362	0.00307368	-0.00408146	0.00369540
C	6.21034602	0.70898497	1.20150281	-0.00392661	0.01016741	-0.00541974
C	6.22611141	2.11320364	1.37114828	0.00476195	0.00712247	0.00525401
C	7.49518272	2.80252655	1.39575830	-0.01853621	-0.00533272	0.00565412
C	7.67444812	4.12832328	1.93222131	-0.00657357	0.00420496	0.01597489
C	6.21829293	6.50968638	1.85800551	0.00520531	-0.01213428	0.00704677
C	7.43873961	7.09238991	1.35689582	0.01853569	-0.01164213	-0.05783193
C	7.43291479	8.49617222	1.08630859	-0.00099900	0.00038777	-0.01584608
C	8.65160894	0.68045217	0.89163084	-0.00098275	-0.00434787	-0.00072292
C	8.65974563	2.10811589	0.94514110	-0.01055913	0.00475668	0.00274285
C	9.89991599	2.81598172	0.83325593	-0.00469784	0.00172339	-0.00062749
C	9.90031296	4.23835471	0.91336721	-0.01008261	0.00200255	0.00362454
C	8.72026089	4.92047080	1.33764168	-0.00598458	0.01233478	-0.00861385
C	8.67437560	6.35569964	1.25593269	-0.00652775	-0.00608629	0.00273936
C	9.88637982	7.06977661	0.98004482	0.00122424	0.00299245	0.00962855
C	9.88766149	8.49400296	0.87015275	-0.00432724	-0.00021129	-0.00298688
Cu	6.08287289	3.47346417	4.81797674	0.04382623	-0.01214861	-0.00315990
Cu	4.55680019	5.86337189	4.26756900	-0.01977137	0.02097873	0.05264999
Cu	6.67915164	7.10774280	3.72979979	-0.02164798	0.02788011	0.08006094
Cu	4.09362588	3.64022893	3.51703875	0.03308271	0.01506911	0.14874150
Cu	6.21237543	4.89795517	2.88792786	0.01172096	0.00455647	0.08746864
Cu	8.26104601	4.09999739	3.95787802	-0.01924762	-0.01471911	0.03572636
C	6.24158177	5.76258519	6.59830749	-0.00546590	0.00574327	0.01773899
O	5.83418806	5.60889018	7.86638981	-0.01687496	0.02134575	-0.00156788
O	7.50059545	6.57315460	6.49549867	0.01625554	0.00252446	0.00410355
H	4.13466446	3.38576852	8.52852772	0.00455816	0.00335775	-0.01490433

O	3.95339392	3.88958691	7.62285565	0.00114728	0.00699873	-0.01175692
H	4.66372661	4.67051963	7.72328757	-0.00171822	0.00194559	-0.01500156
H	0.93211896	8.14773411	3.36828620	0.00091670	0.00472556	0.00208987
O	0.93767462	7.56107372	4.15962363	-0.01335523	0.01403462	-0.02301798
H	1.99237660	7.28138523	4.44574385	0.02024448	-0.00638327	-0.01002892
H	2.17467814	2.55984155	4.93606051	-0.00535820	0.00404004	-0.00117265
O	2.30041830	3.10474432	4.05740757	0.01154388	0.00302316	-0.02107517
H	1.64113170	3.87386414	4.09701654	-0.02115205	0.00724854	-0.00537684
H	7.62231598	3.13869429	9.35319948	-0.00691066	-0.00610689	-0.00096731
O	7.23814559	2.25088602	9.72045817	-0.00372820	-0.01972764	0.00486715
H	7.60504046	2.18055798	10.63467220	-0.00363841	-0.00587812	-0.00297060
H	3.49487601	-0.80874881	6.15096874	0.01157914	-0.00651566	-0.00035683
O	3.59750868	-0.15454394	6.96533576	0.01245914	0.00118728	-0.00215311
H	4.58186937	-0.09183673	7.20994156	-0.00005062	-0.00762005	-0.00964784
H	1.76636952	5.84146446	7.51439869	-0.00169374	0.00119807	-0.01175518
O	1.77480719	5.21524342	6.72462994	-0.00095156	0.00351199	-0.00593281
H	2.47434223	4.54483250	7.02516821	0.00854410	0.00210704	-0.02136850
H	5.75211955	1.71015635	6.90827582	0.00269181	0.01086932	-0.00117771
O	5.28878644	2.32882103	6.27406658	0.01594226	-0.00134889	0.00637138
H	4.64365775	3.01927821	6.87440862	-0.00883148	-0.00243622	0.00099341
H	9.06151608	8.93891426	6.62573124	-0.00928441	0.00041202	-0.00749228
O	8.35325185	9.69410134	6.50748552	-0.00466066	0.00239494	0.00040126
H	7.70726175	9.54567366	7.26132902	-0.00011627	0.00347193	-0.00324726
H	4.14388204	8.59384141	4.26975273	0.01215700	0.01371923	-0.01542970
O	4.61411401	9.46402235	4.19870525	-0.02041552	-0.01790731	-0.01566452
H	4.61118855	9.86397996	5.13655532	-0.01032554	-0.02052071	-0.01267272
H	5.69029565	2.56354301	9.77101558	-0.00417145	-0.00687992	0.00247656
O	4.66941280	2.76026766	9.78137964	0.00568837	-0.00784481	0.01797171
H	4.25712180	1.83799963	9.82385870	0.00756420	-0.00337057	0.00313737
H	7.75053333	7.11242876	9.97261284	-0.00032681	0.00522146	0.00542493
O	8.71592924	6.80452138	9.79535257	-0.00153504	0.00420052	0.00700890
H	9.05577012	6.60225892	10.70423014	-0.00262564	0.00057166	0.00086716
H	-0.13740068	-0.37635527	9.46984271	-0.00239205	0.00524763	0.00177072
O	0.67847858	0.22487077	9.42180698	-0.00089124	0.00526008	0.01400253
H	0.59278642	0.70140053	8.56107266	0.00087519	-0.00043930	0.00093382
H	9.31203450	7.02678275	6.90313507	0.00290573	-0.00677438	-0.00198024
O	9.99127601	7.72510355	6.69540760	0.00709290	-0.01967598	-0.00225890
H	10.19585316	7.62259862	5.70528653	-0.00801806	-0.00406608	0.00320365
H	6.18684862	-0.41619288	8.57272743	-0.00105893	0.00110015	-0.00657639
O	6.18113275	0.07708099	7.65523541	-0.00814383	-0.00255131	-0.00311682
H	6.65260279	-0.58893614	7.06577295	-0.00297331	-0.00324240	-0.00779836
H	8.74682201	2.78621402	6.22822682	-0.00933407	0.00371142	-0.00223891
O	8.70679858	3.78298877	6.07211699	-0.00199140	-0.00052471	-0.00187376
H	8.43469273	4.14619709	6.98824591	-0.00498642	-0.00326620	-0.03011886
H	7.60116471	0.66615328	5.25130718	-0.00920348	0.01424524	-0.00943835

O	7.21178744	0.40483014	4.31505320	-0.00168195	-0.01617269	-0.01457504
H	6.25097996	0.77288166	4.25680820	0.00050197	0.02578790	-0.00229057
H	2.60961064	6.11852374	5.70372003	-0.00958354	0.00090739	0.00491560
O	3.06976646	6.79970374	5.06842792	0.01169791	-0.01529366	-0.01098941
H	7.00960190	4.89163363	8.38206411	-0.00472633	-0.00182065	0.00170750
O	7.95580257	4.44445117	8.58277240	-0.00690814	0.00661342	-0.00274263
H	8.42897713	5.20631520	9.03929369	-0.00558870	0.00296954	-0.00668261
H	5.49591540	8.05328210	10.31497763	0.00732456	0.00068302	0.00609478
O	6.20183776	7.40580312	9.99196407	-0.00094104	0.00453329	0.01790342
H	5.64644091	6.53689308	9.97787404	-0.00043562	0.00517730	0.01282121
H	3.41411942	8.02676457	9.58518503	0.00278531	0.00305852	0.00494106
O	3.95803146	8.76099781	10.04271380	-0.00090693	-0.00264306	0.00780599
H	3.53025234	8.82393940	10.93400163	-0.00366897	-0.00857280	-0.01370044
H	2.54699516	9.54624185	6.45125354	0.00280274	-0.00194427	-0.00187630
O	1.80599059	10.15008376	6.09609226	-0.00323089	-0.00637536	-0.00239446
H	1.24116755	9.55205217	5.54761610	0.00607154	0.00618801	-0.00889466
O	9.42608865	2.09744354	3.92834095	0.00100719	-0.00379198	-0.01504728
O	10.22607955	5.00697584	4.26412727	-0.00622918	0.00284701	-0.01728667
H	8.69398636	1.46779977	3.67178125	-0.00283016	-0.00851735	-0.00706887
H	10.01431948	2.17409437	3.13613362	0.00302573	-0.00490621	0.01108965
H	10.34326301	4.94477108	5.26121645	0.01621774	0.01251186	-0.00420725
H	10.40808871	5.99054917	4.04142923	-0.00877700	-0.01471132	-0.00970614
O	2.47740015	6.94160258	8.80620889	0.00715527	-0.00899800	0.00225827
H	2.92086043	7.44546732	8.01499493	0.00746333	-0.00342566	-0.00325759
H	1.69596887	7.58118732	9.03970087	0.00509548	-0.00353539	0.00357316
O	4.42889620	5.49353083	10.33181376	-0.00062514	0.00318364	0.00940370
H	3.67184324	5.77373881	9.74603799	0.00350443	-0.00370481	0.00584884
H	4.49856360	4.50160730	10.23596307	0.00245827	0.00833111	0.01218634
H	6.51366401	4.79785698	6.06399511	-0.01631223	-0.00517553	-0.03409143
H	7.82575027	6.34584754	5.57378713	0.00587407	-0.01573138	-0.00092689
H	5.48876262	6.31514618	5.94785051	-0.01657401	-0.01426039	-0.00538402

*CH_3OH;*OH
120
Lattice="9.84 0.0 0.0 0.0 8.52169 0.0 0.0 0.0 11.75" Properties=species:S:1:pos:R:3:forces:R:3

C	1.25864413	0.62676560	0.81128440	-0.00598758	-0.00306621	-0.00047946
C	1.26763821	2.05143531	0.72976088	0.00241633	-0.00598652	-0.00307840
C	2.48554218	2.76614183	0.94255740	0.00353219	0.00143403	0.00253660
C	2.49054959	4.18783745	1.00857879	0.00982413	0.00421710	0.01728439
C	1.28329048	4.89723357	0.77054037	-0.00075540	0.00666603	-0.00215842
C	1.27385094	6.31086694	0.87220219	0.00260824	0.00408206	0.00264040
C	2.49495864	7.00999877	1.10906669	0.00122729	-0.00552513	0.01055715
C	2.47589044	8.43912484	1.05188146	0.00149583	-0.00689610	0.01034469
C	3.70176535	0.63457322	1.15070808	0.00139910	-0.00603283	0.02147794
C	3.70303613	2.05122512	1.11120055	0.00554906	0.00413227	-0.00485547

C	4.91988441	2.74956396	1.38037385	0.01696587	-0.00751794	-0.04490599
C	4.79863039	4.08931980	1.82900057	0.01394298	0.00360251	0.00529024
C	3.66570910	4.87017387	1.45188662	-0.00569974	0.01898899	-0.00205278
C	3.70270076	6.31054068	1.41162954	0.00471304	-0.00269705	-0.00242729
C	4.94902092	7.05105640	1.43094004	-0.01272733	-0.00350693	-0.01577396
C	4.93758594	8.45153564	1.20473746	0.01108678	-0.01231785	0.01574810
C	6.16960623	0.64906165	1.05841199	-0.00222793	-0.00365651	-0.00542610
C	6.18533760	2.05775578	1.21431809	0.00530150	0.00848172	-0.00091005
C	7.44063067	2.75129102	1.07310954	-0.01089210	0.00633094	0.00017565
C	7.59856281	4.10578271	1.47217824	-0.01155089	-0.00081582	0.00748131
C	6.22744311	6.46215448	1.65418922	-0.00737296	-0.01462995	-0.01354188
C	7.40375884	7.05157946	1.11435331	-0.00149170	-0.00734118	-0.06096691
C	7.39301569	8.45365472	0.85347146	-0.00605103	-0.00250304	-0.02530887
C	8.62478086	0.62819264	0.67448370	-0.00010953	-0.00169786	0.00009305
C	8.63072561	2.05035480	0.68345320	-0.00820563	0.00410556	0.00264379
C	9.87749010	2.75839853	0.60236726	-0.00953895	0.00198228	-0.01778495
C	9.89369615	4.18273006	0.63792575	-0.00954843	0.00301314	-0.02491283
C	8.67915264	4.87793291	0.95518142	-0.01087044	-0.00222712	-0.01069978
C	8.64647873	6.31817100	0.96027492	-0.00685753	0.00286911	-0.02338846
C	9.87108759	7.02292014	0.81145748	-0.00172815	-0.00405917	0.01065151
C	9.86462360	8.44592261	0.72852700	-0.00206763	-0.00100509	0.00019048
Cu	6.94207427	4.38367118	4.89548220	0.00107180	-0.00098427	-0.00941935
Cu	5.03248939	5.87911519	4.41421227	-0.02345565	-0.01577816	0.04104582
Cu	7.28596963	6.77652918	3.63563357	0.04912015	-0.00608676	0.10165207
Cu	4.74756946	3.53053236	3.84298468	0.01183295	0.02628029	0.03514245
Cu	6.31766172	4.86167451	2.66329805	0.01438120	-0.00003309	0.06725992
Cu	8.66760734	4.53373189	3.20214778	-0.04897244	-0.02064977	0.06722983
C	6.56788906	6.53013581	6.84169362	0.00635205	0.00473467	0.00171875
O	5.93370032	4.59117841	8.42469459	-0.00110295	-0.00431046	-0.00250744
O	6.90531352	7.69024903	6.06638614	-0.00262892	0.00952911	-0.00225454
H	3.41667257	3.47507836	8.03236856	-0.00171483	0.00478908	-0.01071466
O	3.51269077	4.23449529	7.39452025	0.01689073	0.01370612	-0.00263685
H	5.03787805	4.53470651	7.96690791	0.00891933	0.01135093	-0.01453699
H	0.98460485	8.35435857	3.19488498	-0.00993371	-0.00459551	0.00780031
O	1.06971450	7.96136498	4.09920210	-0.00821593	0.00634107	-0.02101375
H	2.03423071	7.55022527	4.19525857	0.01079910	-0.00144694	-0.01300280
H	2.61112825	2.33191605	5.30810115	0.01201174	0.00942858	-0.01200568
O	3.18347126	3.13272050	4.96933125	0.00507187	0.00427526	-0.02048506
H	3.37086789	3.59404227	5.85331574	-0.00143250	0.01277615	0.00416978
H	7.80827322	2.74691982	9.35809963	-0.00570434	-0.00605135	0.00041006
O	7.15043019	2.10024267	9.75503872	-0.00328487	-0.01151369	0.00595501
H	7.23986493	2.30362464	10.72153517	-0.00531998	0.00409614	-0.00691016
H	3.58423704	-1.11479599	6.15160627	0.00547144	-0.00074126	0.00408209
O	3.40685489	-0.55267330	6.95704298	0.00633319	-0.00586900	-0.00796703
H	4.31695080	-0.28819315	7.41245842	0.00202545	-0.00011409	-0.00741670

H	0.82513766	6.15075071	6.70848065	-0.00091440	0.00276997	-0.00094862
O	1.50590487	5.46561469	6.43434999	-0.00533756	0.00694031	-0.00056889
H	2.61189348	4.71964653	7.28644400	0.01661879	0.00437158	-0.00446802
H	5.22107364	2.23036486	7.54552910	0.00467387	0.01034071	-0.02224029
O	5.67134437	2.14392012	6.63263045	-0.00398816	0.00544346	0.00902187
H	5.65403976	3.05443291	6.20305154	0.00084018	-0.00166447	0.02682401
H	8.70940458	9.29384076	6.54833910	-0.01071092	0.00885893	-0.00286598
O	8.26757264	10.04644174	6.06201831	-0.00362562	-0.00832331	0.00251089
H	7.35028898	10.19931337	6.46434021	-0.00392717	0.00524833	-0.00744332
H	4.34225294	8.62112409	4.31968592	0.00884089	0.00663702	-0.01315174
O	5.20523291	9.04594407	4.55095315	-0.01693116	0.00353657	-0.00483078
H	5.04537585	9.69018840	5.31779664	0.00353005	-0.00585721	-0.00732831
H	5.54977648	2.37880419	9.42534264	-0.00531829	-0.00631449	0.00385253
O	4.54864323	2.42549046	9.17929722	0.00132617	-0.01037816	0.01398783
H	4.20275553	1.51585722	9.47038861	0.00110740	0.00498735	0.00721473
H	7.42375318	6.81330771	10.04727070	0.00020647	0.00467315	0.01320664
O	8.40006228	6.74772836	9.76836146	-0.00618076	0.00329849	0.01134025
H	8.84346374	6.61869444	10.65090679	-0.00368275	-0.00066475	-0.00177055
H	-0.41353358	-0.43505485	9.27813151	-0.00683465	0.00334562	0.00056393
O	0.46324740	0.05790515	9.21770613	-0.00215740	0.00455551	0.01078482
H	0.49300319	0.41192926	8.29494869	0.00179679	-0.00146425	-0.00292222
H	8.60684221	7.38011554	6.14769482	0.00016823	-0.01559346	-0.02243552
O	9.54539422	7.57814063	6.44025968	-0.00012513	-0.01466282	-0.00467353
H	9.98797971	7.78849874	5.56278601	-0.01293106	-0.00699527	-0.00697747
H	5.72983915	-0.67340091	8.82652778	-0.00291542	0.01087312	0.00339582
O	5.55783437	0.08594318	8.19682501	0.01583471	-0.00927316	0.00409204
H	6.28558381	0.03929767	7.52300898	0.00069017	0.00185140	0.00952376
H	8.86142465	3.11518123	5.93297600	0.01803145	0.00205937	-0.00858912
O	8.68994942	4.10536880	5.99000892	-0.01050521	0.00797849	-0.00184021
H	8.68117343	4.26112641	7.01056723	-0.00255630	0.00496416	-0.01662480
H	7.97845422	0.75520467	4.66791646	-0.00297806	0.01104523	-0.01898995
O	7.92953751	0.35807943	3.71727783	-0.00215603	-0.00940801	-0.01667983
H	7.09387986	0.76115934	3.35504274	0.00034294	0.03598725	0.00635409
H	2.15477454	5.96700249	5.82949408	0.01123220	-0.00086001	-0.00191483
O	3.18099705	6.61852828	4.60995264	0.00727617	-0.00811441	-0.01034559
H	2.84277378	5.89756459	4.00973924	0.00256674	-0.01135518	-0.01338580
H	7.45222487	4.32888057	8.43347786	-0.00903775	-0.00355552	-0.00999048
O	8.46402148	4.24218544	8.57180773	-0.00523933	0.00960658	0.00354232
H	8.65711275	5.10514204	9.05501373	-0.00190385	-0.00118382	-0.00031664
H	5.08914648	7.52833830	10.40523159	0.00518191	-0.00386162	0.00172864
O	5.72561208	6.75106906	10.19910697	0.00333336	0.00217206	0.01598946
H	5.08325308	5.94929880	10.40784734	0.00490283	0.00766943	0.01075063
H	3.17387662	7.81806311	9.73186419	0.00403650	-0.00039755	0.00839326
O	3.80081208	8.54741955	10.13016713	-0.00477113	-0.00031435	0.01609318
H	3.39941215	8.74422212	11.01272111	-0.00025262	-0.00665664	-0.01710060

H	2.40420627	9.16794518	6.44486937	0.00202507	-0.00530668	0.00003020
O	1.75093536	9.87205031	6.10104345	-0.00657588	-0.00865231	-0.00164223
H	1.30425920	9.41872571	5.33429195	-0.00365391	0.00285779	0.00026986
O	9.76240846	2.50060997	3.65307647	-0.00038670	-0.00615722	-0.01123172
O	10.43882159	5.23653999	3.88241021	-0.00469557	-0.00139993	-0.02091319
H	9.13479116	1.75372401	3.42487600	-0.00486825	0.01008203	-0.00463895
H	10.39651440	2.51210387	2.89248770	0.00599781	-0.00826839	0.01323562
H	10.51169745	5.00178710	4.86258594	0.01214395	0.00371047	-0.00346822
H	10.41668502	6.24070568	3.88672394	-0.00553959	0.00590464	-0.00560261
O	2.31576147	6.74396945	9.05946432	0.00620044	-0.01309933	0.00223499
H	2.68121207	7.02027154	8.15539502	0.00649471	-0.00339785	0.00164128
H	1.48980937	7.36056055	9.14182993	0.00650125	-0.00852626	0.00472481
O	3.94172608	4.89355493	10.39373358	-0.00195460	0.00136928	0.00788696
H	3.23083593	5.32655951	9.84057414	0.00557276	0.00009210	0.00670790
H	4.13668191	3.99217250	9.99267097	-0.00247042	0.00309120	0.01959816
H	5.49221155	6.49345809	7.09419122	-0.01051526	-0.02043850	-0.01698142
H	5.84689497	5.40855514	8.97993762	-0.00505032	-0.00967451	-0.00165550
H	7.19261876	6.56768253	7.75451806	0.00245105	-0.00632053	-0.01736236
H	6.14935514	7.99252493	5.43759666	-0.00063049	0.01927166	0.01691795
H	6.82507765	5.59277112	6.29584358	0.00650253	-0.00500977	-0.00455789

*CH_3OH;*OH (oxidated)

120

Lattice="9.84 0.0 0.0 0.0 8.52169 0.0 0.0 0.0 11.75" Properties=species:S:1:pos:R:3:forces:R:3

C	1.25498670	0.68170222	0.78954930	-0.00270187	-0.00750785	0.00106229
C	1.26052036	2.10460269	0.69920951	0.00168620	-0.00580629	-0.00179892
C	2.48038611	2.81700764	0.90766176	0.00490966	0.00351139	0.00310623
C	2.48565912	4.23791593	0.98298959	0.00976103	0.00454327	0.01930380
C	1.27374198	4.94780674	0.77579730	-0.00089451	0.00808431	0.00084916
C	1.26725849	6.36488574	0.87824631	0.00160733	0.00288078	0.00257676
C	2.49152486	7.06640942	1.07900576	0.00047434	-0.00385396	0.00163941
C	2.47207875	8.49357144	1.01764700	-0.00125648	-0.00691640	0.01059906
C	3.70045492	0.68416823	1.12066210	0.00035143	-0.00635870	0.01949063
C	3.69931903	2.10240192	1.08404343	0.00499086	0.00593077	-0.00662597
C	4.90926486	2.80076821	1.37519200	0.01521725	-0.00650467	-0.04978129
C	4.78353570	4.14353092	1.82819863	0.01381770	0.00692773	0.00944917
C	3.66510889	4.91873904	1.42309409	0.00040073	0.01869933	0.00441502
C	3.70354295	6.36525242	1.40436117	0.01659205	-0.00671275	-0.00677147
C	4.95223243	7.10662245	1.47927554	-0.00928690	-0.00741910	-0.00470314
C	4.93576237	8.50416715	1.22771078	0.01117643	-0.01416077	0.01678845
C	6.16581566	0.70315784	1.10345156	-0.00047031	-0.00430212	-0.00087631
C	6.17513719	2.11142570	1.22377813	0.00816844	0.00663841	-0.00112381
C	7.42831158	2.80753630	1.05947139	-0.01158339	0.00799965	-0.00242600
C	7.56207734	4.16547227	1.43701469	-0.01186946	-0.00072214	0.00598357
C	6.22357970	6.52964020	1.79052330	-0.00599736	-0.00965326	0.00247933

C	7.40508787	7.11206896	1.21709848	0.00183781	-0.00422243	-0.05980864
C	7.39242828	8.50616974	0.92860920	-0.00564115	-0.00170118	-0.01476563
C	8.61815356	0.68161554	0.71036914	-0.00066166	-0.00151638	-0.00084585
C	8.61928704	2.10328641	0.67651015	-0.00925779	0.00145396	-0.00059749
C	9.86559288	2.81356756	0.58454286	-0.00870175	0.00257537	-0.02014611
C	9.88147162	4.23782905	0.64685239	-0.00883927	0.00432907	-0.02414213
C	8.66453389	4.93175122	0.97805906	-0.00937265	-0.00236178	-0.00581091
C	8.63731373	6.37649355	1.02701404	-0.00632040	0.00309085	-0.02063824
C	9.86206313	7.07822815	0.85147917	-0.00153308	-0.00842904	0.01083559
C	9.85858089	8.50062712	0.75593766	-0.00174172	0.00000848	-0.00029851
Cu	7.06620319	4.31892950	4.93508141	0.01629352	-0.01390245	-0.01930540
Cu	5.07590839	5.64649686	4.80090758	-0.01216697	-0.02405144	0.00664580
Cu	7.27995317	6.72905408	3.70791379	0.04458048	0.01733455	0.09994314
Cu	4.77252378	3.47422152	3.82899065	0.00957110	0.03080774	0.02501750
Cu	6.28548258	4.86876516	2.70738678	0.00795030	0.00131928	0.06369796
Cu	8.64785531	4.48846539	3.16693505	-0.04450225	-0.01482592	0.05561808
C	6.11305396	6.30744595	6.29278178	-0.00521143	-0.00604030	-0.00322034
O	5.97134510	4.62163772	8.54642483	0.00479817	-0.00133960	-0.00678705
O	6.80986620	7.56827468	5.91243045	0.00339215	0.01154997	-0.00678038
H	3.52075265	3.48750482	8.06251856	0.00053490	0.00651763	-0.01121149
O	3.52093761	4.24389845	7.40650317	0.01410635	0.01135860	-0.00036138
H	5.13191232	4.60333419	7.99544159	0.00641066	0.00832096	-0.01343497
H	0.35697866	8.17570883	3.27117767	-0.00943322	-0.00821807	0.00153770
O	0.77702632	7.95442762	4.14280576	-0.00543738	0.00181007	-0.01935547
H	1.68147306	7.53981022	3.95406926	-0.00079652	0.00030554	-0.00630315
H	2.55592587	2.43203028	5.24318168	0.01064496	0.01418142	-0.01074637
O	3.11070603	3.24361821	4.93047563	-0.00074924	0.00257681	-0.02082720
H	3.32735864	3.65506318	5.83495966	-0.00379459	0.01030005	0.00488006
H	7.76971507	2.74793274	9.36596976	-0.00548950	-0.00546698	0.00001374
O	7.11038277	2.10916090	9.76897953	-0.00196941	-0.01036102	0.00585319
H	7.15699943	2.36618270	10.72755779	-0.00649825	0.00474051	-0.00631256
H	3.94898428	-1.13496989	6.05060964	-0.00726945	0.01730550	0.00962302
O	3.52937759	-0.58361061	6.77396982	0.00455553	0.00372974	-0.00752855
H	4.37624887	-0.29376415	7.33662518	0.00160818	0.00252171	-0.00514321
H	0.68112326	6.20060306	6.91815545	0.00065101	-0.00023038	-0.00334585
O	1.47577953	5.63325347	6.67372824	-0.00057318	0.00901914	-0.00935373
H	2.61734540	4.70933369	7.41559978	0.01570373	0.00315436	-0.00643201
H	5.17343190	2.22677717	7.54772319	0.00403116	0.00961441	-0.01899261
O	5.65718401	2.18041223	6.62671022	-0.00072914	-0.00037306	0.00880787
H	5.64181716	3.09561847	6.20224655	-0.00131311	0.00108788	0.02432896
H	8.58121195	9.24896965	6.81810434	-0.01397041	0.00818458	-0.00242542
O	8.22470034	10.03025858	6.30957640	-0.00543818	-0.01030402	0.00256085
H	7.28300992	10.23901982	6.63938702	-0.00462358	0.00667701	-0.00656219
H	4.31140685	9.06454726	4.20659988	0.00643101	-0.00167534	-0.00795919
O	5.22723847	9.23413826	4.52606422	-0.01462271	-0.01066302	-0.00864825

H	5.15524611	9.83484623	5.35891067	0.00745835	-0.01064158	0.00073584
H	5.57133233	2.37848085	9.35364123	-0.00558467	-0.00786282	0.00188675
O	4.58467242	2.38748712	9.03015873	-0.00058085	-0.01086807	0.00934593
H	4.25081140	1.48267526	9.36593542	0.00286580	0.00336645	0.00442837
H	7.53229801	6.84239528	9.99685931	0.00073976	0.00470422	0.00902680
O	8.52135109	6.74460600	9.77568851	-0.00775203	0.00241283	0.00469439
H	8.90103781	6.62260204	10.69012152	-0.00336119	-0.00039153	0.00034702
H	-0.26227975	-0.49045874	9.30814338	-0.00349753	0.00252781	0.00126025
O	0.60502424	0.02571435	9.22593361	-0.00083608	0.00409834	0.01038615
H	0.57455630	0.42889980	8.32455542	0.00315300	-0.00158054	-0.00293868
H	8.44649946	7.33999639	6.33448226	-0.00206753	-0.01365748	-0.01831843
O	9.38675308	7.53560580	6.63707962	-0.00328542	-0.01466446	-0.00678461
H	9.81735629	7.78154823	5.76769547	-0.01288764	-0.00848072	-0.00716151
H	5.83396372	-0.68329517	8.75728119	0.00226717	0.00640999	-0.00760051
O	5.69265289	0.01918437	8.06212029	0.01729450	-0.00450681	0.00767796
H	6.33173532	-0.22372213	7.32537463	0.00009851	0.00101553	0.00233456
H	8.96447284	3.03917795	6.06296634	0.01509209	-0.00291458	-0.00747376
O	8.90209391	4.04529317	6.06242328	-0.01141656	0.00641332	-0.00056458
H	8.83189066	4.23237240	7.08587084	-0.00511966	0.00524347	-0.02013186
H	7.99536885	0.86820895	4.82209146	-0.00561753	0.00499464	-0.02004095
O	7.94396793	0.53809654	3.84989631	-0.00358950	-0.00482802	-0.01881704
H	7.07140425	0.90703983	3.54089243	0.00252198	0.02717328	0.00104198
H	2.11244167	6.30129721	6.31949905	-0.00446023	-0.00597307	-0.00629671
O	3.00110764	6.37542679	4.14315994	0.00739824	0.00182424	-0.01059700
H	2.33476332	5.60729763	4.13754965	0.01691027	-0.01372216	-0.01152832
H	7.48143819	4.37347050	8.44250276	-0.00815778	-0.00204698	-0.00981718
O	8.49341051	4.26581909	8.58410634	-0.00575496	0.00771750	0.00328442
H	8.69801306	5.13100632	9.06621325	-0.00352380	-0.00052533	-0.00222940
H	5.27231932	7.59351080	10.40345796	0.00416995	-0.00216400	0.00387379
O	5.86649833	6.78393934	10.21414355	0.00189158	0.00311800	0.01804162
H	5.19622829	6.01220555	10.44724695	0.00354304	0.00810767	0.01447159
H	3.34305708	7.87136922	9.71459634	0.00370087	-0.00073177	0.00780712
O	3.97854082	8.59938004	10.10907876	-0.00430253	-0.00082291	0.01752772
H	3.57181267	8.83483116	10.98077971	-0.00126885	-0.00774890	-0.01467065
H	2.43150897	9.14776111	6.29220782	0.00252635	-0.00516350	0.00043369
O	1.74515252	9.85424178	6.05086254	-0.00639996	-0.00889323	-0.00033865
H	1.23736297	9.45296904	5.29327636	-0.00377939	0.00398961	-0.00204634
O	9.76779890	2.51783073	3.59199068	0.00184870	-0.00236987	-0.01081868
O	10.39154255	5.17317777	4.08759864	-0.00024931	0.00155602	-0.02228340
H	9.06992659	1.79627434	3.49284080	-0.00625555	0.00501216	-0.00422001
H	10.33701710	2.41345735	2.78844412	0.00369461	-0.00521740	0.01679197
H	10.17129165	4.74996929	4.98558939	0.00168980	0.00485371	0.00034579
H	10.17319784	6.13710956	4.23214476	-0.00987885	0.00398235	0.00149021
O	2.48797307	6.80410965	9.07351234	0.01033003	-0.01134100	0.00145447
H	2.82166707	6.98091544	8.14442666	0.01209922	0.00125805	0.00760914

H	1.64384404	7.42021813	9.12726679	0.00648719	-0.00834953	0.00514038
O	3.99901217	5.00836929	10.44339939	0.00124056	-0.00178742	0.00358070
H	3.32231156	5.47115176	9.86653555	0.00432730	-0.00112787	0.00617984
H	4.23031099	4.15064784	9.99318936	-0.00674896	0.00509680	0.02409514
H	5.51671619	6.50931001	7.20640659	-0.00102317	-0.01522061	0.00850630
H	5.94948856	5.52679168	8.96027801	-0.00502058	-0.00710783	-0.00917584
H	6.94638021	5.65547012	6.64850599	0.01332191	0.00742286	0.00453437
H	3.30487832	6.39811159	3.17194595	-0.00767562	-0.00409135	0.00690466
H	6.17226178	8.10344510	5.32148517	-0.00811129	0.00901205	0.01370601

*CH_2OH

120

Lattice="9.84 0.0 0.0 0.0 8.52169 0.0 0.0 0.0 11.75" Properties=species:S:1:pos:R:3:forces:R:3

C	1.06449942	0.67814969	0.80275366	-0.00108634	-0.00298506	-0.00430643
C	1.07000188	2.10176019	0.70497578	0.00183292	-0.00631565	-0.00354621
C	2.29513259	2.81347338	0.88288365	0.00533385	0.00178687	0.00202879
C	2.29721475	4.23684817	0.94619791	0.01284005	0.00565761	0.01146475
C	1.07984145	4.94685193	0.75470528	-0.00251302	0.00646232	-0.00016013
C	1.07510022	6.36259518	0.87618529	0.00515240	0.00199331	0.00471400
C	2.30046145	7.06298817	1.08201755	0.00366027	-0.00494117	0.00664903
C	2.28439222	8.48995221	1.01751083	0.00297225	-0.00548607	0.00477848
C	3.51303344	0.68089520	1.12839837	-0.00048146	-0.00426758	0.01985330
C	3.51415600	2.09998913	1.07668845	0.00553171	0.00567687	-0.00335379
C	4.71765882	2.80229919	1.38683489	0.01510121	-0.00377849	-0.03631562
C	4.57584195	4.13882289	1.84533449	0.01390567	0.00829585	0.00608333
C	3.46847786	4.91730588	1.41316792	0.00294112	0.02048207	-0.00444149
C	3.50612921	6.36006536	1.39700912	0.01615789	-0.00405525	0.00733788
C	4.74652971	7.09445874	1.50670479	-0.00729632	-0.00983899	-0.00184561
C	4.74414001	8.49698320	1.27180244	0.00783474	-0.01034604	0.01437773
C	5.98110715	0.69719492	1.16979819	0.00271628	-0.00562704	-0.00499462
C	5.98635307	2.10893450	1.26470882	0.00521144	0.00751991	-0.00184161
C	7.24468870	2.79975307	1.13144023	-0.01302660	0.00681184	-0.00084334
C	7.40309521	4.15135473	1.53944557	-0.01143238	0.00385246	0.01065506
C	6.01606365	6.49347305	1.80545432	-0.00236523	-0.00716964	0.00168988
C	7.22635114	7.09943057	1.32082024	0.00522189	-0.00559700	-0.02068350
C	7.21198548	8.50397967	1.03482477	-0.00651241	-0.00103432	-0.01513630
C	8.42875140	0.67544652	0.78713591	-0.00438818	-0.01143481	0.00438743
C	8.42969840	2.09847954	0.74035958	-0.00827670	0.00419535	0.00364917
C	9.67412993	2.81061925	0.61693851	-0.00998585	0.00199835	-0.01588508
C	9.68492091	4.23536547	0.65926118	-0.01103586	0.00413943	-0.02238618
C	8.47917466	4.92736135	1.02964602	-0.01529742	0.00918718	-0.01369203
C	8.45393276	6.36723938	1.09374800	-0.00879904	0.00125395	-0.01755760
C	9.67118626	7.07298839	0.89021339	-0.01229493	-0.00594562	0.01345754
C	9.66896792	8.49546369	0.79623823	-0.00489070	0.00009798	0.00153567
Cu	6.77410483	3.95101283	4.96938467	-0.02239767	-0.06821637	-0.01525380

Cu	5.12080200	5.67459296	4.79554090	0.01176356	0.00241696	0.04083061
Cu	6.83507008	6.97720251	3.61715842	0.00843609	0.03834107	0.05329214
Cu	4.55671546	3.49010541	3.94453332	0.03405257	0.02175276	0.02126398
Cu	6.09174044	4.82931497	2.74936441	0.01236911	0.00603372	0.06209576
Cu	8.40511539	4.51020859	3.27592469	-0.03177650	-0.01860037	0.06045840
C	6.53989353	7.17853593	5.53669524	-0.00426958	0.00904820	-0.01644119
O	5.44687637	5.06271136	7.97244473	0.00557021	0.00977639	-0.00768557
O	7.50936722	6.54978275	6.46574693	0.02344697	-0.00105111	0.01751496
H	3.34362082	3.16011105	8.03907963	0.00050124	0.00413092	-0.01355327
O	3.12360716	3.91811727	7.42774865	0.01590955	0.00436088	-0.00057411
H	4.00694501	4.41536852	7.42370226	0.01579424	0.00689204	-0.02379659
H	0.77363076	8.42287710	3.20634443	-0.00826756	-0.00216967	0.00703798
O	0.94929935	8.01391263	4.09266849	-0.00765076	0.00758319	-0.01954388
H	1.81735329	7.47433419	4.02503574	0.00785513	-0.00208662	-0.01113334
H	2.46669096	2.33429496	5.13487989	-0.00318623	0.00597006	-0.00484893
O	2.75194645	3.27309480	4.84466857	0.00364462	0.00784260	-0.02263083
H	2.92354804	3.60943164	5.78170616	0.00023356	0.00537010	-0.00002424
H	7.51955495	3.08633480	9.43865022	-0.00682274	-0.00235261	0.00167027
O	7.06880324	2.23411258	9.76288190	-0.00043335	-0.01226336	0.00978686
H	7.21650373	2.26282825	10.74491185	-0.00575610	0.00097756	-0.00552099
H	3.77154920	-1.29081508	6.30984142	0.00179586	0.01106578	0.01601342
O	3.62669001	-0.74380279	7.12346215	-0.00094061	-0.00477738	-0.00426791
H	4.58167422	-0.55988989	7.50051750	-0.00201567	0.00238853	-0.00589676
H	0.91809725	6.36785077	6.85511525	0.00150884	0.00138090	0.00178502
O	1.41305451	5.59050976	6.44102313	-0.00294210	0.00638863	0.00521880
H	1.85602357	5.00557762	7.13002152	-0.00253633	0.01063889	-0.00640939
H	5.16267848	2.04006193	7.63001667	0.00651856	0.00250833	-0.01892524
O	5.59647224	1.91445786	6.71337770	-0.00508084	-0.00927466	0.00667878
H	5.56657040	2.83034217	6.27205870	0.00010704	0.00511807	0.01724201
H	8.75363053	9.16762200	6.86241717	-0.01147432	0.00215500	-0.00243993
O	8.22633194	9.91281320	6.44659033	-0.00134650	-0.01162363	0.00300274
H	7.30278432	9.99273328	6.85202484	-0.00412482	0.00079771	-0.00275370
H	4.46445649	8.92672190	3.93990790	0.00792264	-0.00644039	0.00028927
O	5.01849024	9.69306006	4.22665148	-0.00822892	-0.00940156	-0.00935028
H	5.00841243	9.71755460	5.23603656	0.00539611	-0.01373561	-0.00201532
H	5.61664474	2.32920637	9.48219320	-0.00328150	-0.00410796	0.00187478
O	4.59098925	2.35304448	9.19137834	0.00170926	-0.00483284	0.00843383
H	4.25817698	1.44243196	9.50272914	0.00386974	-0.00272546	0.00482553
H	7.73584866	7.00348835	10.15442742	0.00053788	0.00425435	0.00776350
O	8.65437625	6.81330568	9.77624912	-0.00397978	0.00365262	0.00600410
H	9.16502826	6.63227840	10.61184722	-0.00177898	-0.00133340	-0.00234178
H	-0.08953198	-0.25183555	9.35169712	-0.00317218	0.00364314	0.00145596
O	0.81202768	0.17278794	9.24003927	0.00207787	0.00195720	0.01227389
H	0.83699077	0.38064460	8.27085635	0.00862126	0.00038528	0.00257230
H	8.85079896	7.20433008	6.60740860	-0.00325808	-0.00837575	-0.00936840

O	9.73956459	7.72544181	6.72366861	-0.00044945	-0.01295475	-0.01399195
H	9.98731320	7.93821753	5.78212156	-0.01290248	-0.00471560	-0.00371192
H	6.01579082	-0.91514508	9.07924019	0.00058807	0.00583972	-0.00631203
O	5.99523363	-0.42596217	8.18497752	0.00732713	0.01178676	0.00494499
H	6.72936307	-0.86457083	7.68295395	0.00218341	-0.00015745	0.00665745
H	8.70796451	2.97847209	6.22385377	0.01742557	0.00209255	-0.00617493
O	8.51003619	3.97131280	6.23934068	-0.00565653	0.00075171	-0.00334465
H	8.36298157	4.15517443	7.24368940	-0.00664948	0.00312643	-0.01750442
H	7.96431152	0.94930040	4.84874249	-0.00962712	0.00288663	-0.02074979
O	7.85749915	0.79370988	3.84969268	-0.00410946	-0.00723342	-0.01439304
H	6.92086451	1.11141300	3.69596140	-0.00085477	0.03946400	-0.00051202
H	2.36157915	5.99272016	5.33149837	0.01777061	-0.00239209	-0.01079705
O	2.95230403	6.37601493	4.54403374	0.00839096	0.00512699	-0.01761255
H	2.85242670	5.68073585	3.83422869	-0.01876183	-0.00488405	-0.00946607
H	6.96345223	4.75174797	8.42309307	-0.00945200	0.00295887	-0.01411269
O	7.91035582	4.48651198	8.70138823	-0.01258534	0.01122538	0.00378732
H	8.30814881	5.32814474	9.11696931	-0.00867192	0.00488846	-0.00366203
H	5.28945645	7.60600760	10.65891100	0.00735859	-0.00409011	-0.00336529
O	6.04094758	6.93726251	10.48070279	-0.00485463	-0.00829460	0.01747418
H	5.58696567	6.06070757	10.63796815	0.00541562	0.00012350	0.01809358
H	3.34318564	7.76139024	9.83868591	0.00347973	-0.00155647	0.00599193
O	3.96885395	8.51163521	10.16276074	-0.00152020	-0.00303899	0.00723763
H	3.55872773	8.80227762	11.02254455	-0.00064722	-0.00749058	-0.01453013
H	2.66339024	9.03904545	6.46616124	0.00547345	-0.00258459	-0.00103508
O	1.96549235	9.65474769	6.07669702	0.00180632	-0.00909201	0.00172589
H	1.56209480	9.13065380	5.32718777	0.00118382	0.00032037	0.00081192
O	9.67463342	2.68877752	3.71513523	0.00665861	-0.00293395	-0.01278511
O	10.01258389	5.40437542	4.05664809	-0.00307328	0.00239674	-0.01525586
H	8.98834050	1.95828554	3.54663208	-0.00327674	-0.00944487	-0.00368834
H	10.29890181	2.65343800	2.94930594	0.00764360	-0.00767021	0.01130122
H	10.21210480	5.10453736	4.99239249	0.00288255	0.00311808	-0.00102595
H	10.02786468	6.40216730	4.07753798	-0.01104913	0.00221665	-0.00289343
O	2.40900247	6.62095205	9.22284628	0.00495857	-0.01176845	0.00480725
H	2.82436219	6.85089735	8.32970486	0.00616525	-0.00189864	-0.00039123
H	1.66895033	7.34711552	9.27531674	0.00540541	-0.00685020	0.00576238
O	4.26303282	4.86017309	10.20373623	0.00043645	0.00287100	0.02406905
H	3.37808795	5.29461623	10.00283286	0.00353768	0.00072313	0.01088283
H	4.16448472	3.86073073	10.08432004	0.00467404	0.00755881	0.01435091
H	5.56249990	6.01562949	7.72949122	-0.00960280	-0.02392851	-0.01189732
H	4.97761431	5.07367143	8.91497769	0.00586242	0.00810060	-0.01399231
H	6.59844523	8.26644830	5.74705793	-0.01736620	0.00371344	0.00969793
H	7.73606809	5.65044489	6.07833584	-0.00442720	0.01830620	-0.01925246
H	5.52364506	6.95703162	6.02523983	-0.00274212	-0.01066725	-0.01542075

*CHO;*OH (oxidated)

117

Lattice="9.84 0.0 0.0 0.0 8.52169 0.0 0.0 0.0 11.75" Properties=species:S:1:pos:R:3:forces:R:3

C	1.26392361	0.70583066	0.87251184	-0.00067442	-0.00031579	-0.00267324
C	1.27351890	2.13046929	0.80683965	0.00246711	-0.00437132	0.00138267
C	2.50799264	2.84296245	0.89877442	-0.00301072	-0.00673813	-0.01024839
C	2.50533586	4.26711088	0.93546567	0.00455948	0.00437577	-0.00970221
C	1.27170800	4.97555840	0.87316326	0.00086600	0.00398980	0.00517289
C	1.27027213	6.39109819	0.96546975	0.00263770	0.00243304	0.00344911
C	2.50499920	7.10013031	1.08651072	0.00126268	-0.00194555	0.00833151
C	2.49421055	8.52402545	1.03006398	0.00271980	-0.00159107	0.00552312
C	3.72545479	0.71686545	1.13201599	0.00045831	-0.00191585	0.02099264
C	3.73515807	2.13722834	1.08550276	0.00855757	0.00003986	-0.00583757
C	4.93754944	2.83710080	1.42466529	0.02612855	-0.01260272	-0.02793819
C	4.80340337	4.20828779	1.80810193	0.00955630	0.00600320	0.00917862
C	3.68981490	4.96503832	1.34362779	0.00404310	0.03333698	-0.04535256
C	3.71470567	6.40030907	1.36109266	0.01265929	0.00447900	0.00266101
C	4.94402280	7.12501666	1.51783405	-0.01368205	-0.00632548	-0.00642801
C	4.95103621	8.52858841	1.28816423	0.00512012	-0.00696472	0.00726229
C	6.18743755	0.72780149	1.26763191	-0.00157515	0.00345799	-0.00092237
C	6.19774348	2.13936496	1.42083064	0.00091155	0.01042746	0.01042921
C	7.47165871	2.82729871	1.43252931	-0.01584990	-0.00665626	0.00034123
C	7.65469489	4.14603640	1.97113158	-0.00529744	0.00261461	0.01920596
C	6.18761804	6.52775380	1.94471161	0.00128754	-0.01204505	0.00681716
C	7.42143278	7.11280166	1.46049776	0.01667962	-0.00739670	-0.05461053
C	7.41156562	8.51529213	1.16169511	-0.00015877	0.00101224	-0.01261041
C	8.62695437	0.70090835	0.93536125	-0.00086078	-0.00359283	-0.00189885
C	8.63274442	2.13157062	0.96293932	-0.00953936	0.00328321	0.00297420
C	9.87609357	2.83749539	0.84665146	-0.00420868	0.00165707	-0.00100585
C	9.87868445	4.25848115	0.94215541	-0.00981846	0.00342954	0.00370207
C	8.70318659	4.93781954	1.39794876	-0.00307490	0.01327651	-0.00681821
C	8.65707945	6.37844402	1.33190114	-0.00424407	-0.00530633	0.00205294
C	9.86533564	7.09322124	1.03004885	0.00050628	0.00126063	0.00894853
C	9.86556913	8.51675730	0.90852833	-0.00504499	0.00066212	-0.00239034
Cu	5.98722270	3.50743755	4.94754859	0.03227047	-0.05103063	-0.02600145
Cu	4.87620597	5.86538631	4.94514627	-0.00009071	0.02399663	0.00797637
Cu	6.75282055	7.04210103	3.86344656	-0.02659279	0.02291924	0.10013693
Cu	3.99487417	3.97349921	3.62567613	0.03761954	0.00502102	0.13211876
Cu	6.17761628	4.91489625	2.95747594	0.01407399	0.00073271	0.06262290
Cu	8.23200217	4.06806530	3.97134112	0.02393862	-0.02950838	0.02726051
C	6.03671327	5.21739417	6.26628203	-0.02901165	0.00613528	0.03419207
O	5.64704866	5.24877343	7.51012019	-0.00682165	0.02366928	0.01822119
O	8.34801476	6.63589083	6.71465874	0.01294339	0.00635232	0.00219693
H	3.92732179	3.26376424	8.56471125	0.00115266	0.00204598	-0.01678464
O	3.62405097	3.67047435	7.65696626	-0.00264851	0.00391884	-0.00827782
H	4.25275758	4.47307206	7.62053989	0.00607142	0.00624764	-0.01882620

H	0.98385164	8.22708614	3.20639834	-0.00013170	0.00363105	0.01484260
O	0.99580527	7.60592803	3.97241098	-0.00855814	0.01231722	-0.02033422
H	2.01108461	7.32220970	4.17767047	0.01344888	-0.00656639	-0.01026515
H	2.15850169	2.73462704	4.86998627	-0.00967337	-0.00100873	0.00072439
O	2.21428096	3.27374799	3.98030904	0.01247163	0.00483226	-0.01890722
H	1.51719017	4.00818161	4.04845565	-0.02599445	0.00352780	-0.00483414
H	7.64574997	3.18030638	9.27941158	-0.00471512	-0.00442656	-0.00467329
O	7.27344110	2.28552725	9.57862262	-0.00320156	-0.01629973	0.00022970
H	7.66255934	2.14610112	10.47875774	-0.00234571	-0.00639485	0.00206074
H	3.41881566	-0.77400377	6.02672043	-0.00162945	0.00836652	0.00803055
O	3.53719913	-0.15856661	6.86533826	0.00463358	0.00633279	-0.00039032
H	4.52490129	-0.18776676	7.09165628	-0.00188817	-0.00957155	-0.01187321
H	1.79941342	5.87691617	7.44780916	-0.00270713	-0.00001438	-0.01443475
O	1.69366549	5.27677455	6.64586779	-0.00446364	0.00775985	-0.00764667
H	2.19098029	4.45078530	6.94310000	0.00647326	0.00149710	-0.02086918
H	5.68755082	1.61842137	6.97631233	0.00090209	0.01488491	-0.00317642
O	5.22529361	2.24702631	6.35852672	0.01583725	-0.00219215	0.00115537
H	4.48651354	2.80738543	6.91427180	-0.00671789	-0.00119665	0.00028433
H	9.12509880	9.08765128	6.53140254	-0.00990034	-0.00379053	-0.00695333
O	8.26137013	9.67388580	6.52747087	-0.00692917	-0.00060856	0.00199581
H	7.66488499	9.33285381	7.25934259	0.00339646	0.00228189	-0.00281992
H	4.14923595	8.58947285	4.16857115	0.01482252	0.00094453	-0.01247742
O	4.61411551	9.46473205	4.14141772	-0.01971667	-0.01608939	-0.01678894
H	4.56347958	9.81894402	5.08527170	-0.00840830	-0.01311734	-0.01940642
H	5.68229388	2.57742687	9.72263325	-0.00523216	-0.00550387	0.00111051
O	4.67150516	2.74716396	9.80771826	0.00644841	-0.00713292	0.01693939
H	4.28244334	1.81999615	9.92535389	0.00690498	-0.00191266	0.00387426
H	7.65611706	7.13760391	10.13512739	0.00269706	0.00520292	0.00851532
O	8.62479077	6.80763028	9.98613647	0.00247014	0.00567312	0.00915457
H	8.95683252	6.62094659	10.90517760	-0.00345299	-0.00062597	-0.00019999
H	-0.15663490	-0.41533787	9.54335675	-0.00275435	0.00416380	-0.00128973
O	0.65930003	0.16773373	9.41903378	-0.00253726	0.00500014	0.01128467
H	0.56768364	0.55047457	8.51232841	0.00034829	0.00140258	0.00549183
H	9.70074374	7.40986575	6.68592717	0.00679676	-0.00209219	-0.00601479
O	10.35135614	8.17348140	6.49229254	0.01044719	-0.02373336	-0.00986877
H	10.52562990	8.05154390	5.49023256	-0.00332967	-0.00520358	0.00227087
H	6.14575718	-0.63591800	8.51385816	-0.00026734	-0.00262720	-0.00908929
O	6.16193737	-0.17889669	7.57379547	-0.00323633	-0.01145809	-0.00741386
H	6.59352025	-0.87127993	6.99825774	-0.01144526	-0.00554315	-0.01719351
H	8.66002385	2.68684882	6.32324542	-0.01143303	0.01318540	-0.01081100
O	8.91966120	3.65906894	6.19641526	0.00747711	-0.00485019	0.00228678
H	8.73299757	4.03925937	7.10170683	-0.00269292	-0.00531381	-0.02620522
H	7.54638288	0.67655583	5.23933772	-0.00725510	0.01790496	-0.00534046
O	7.20404733	0.41074985	4.29552640	0.00062304	-0.01252469	-0.01487406
H	6.23031815	0.74608628	4.20314549	-0.00013806	0.03532927	-0.00104778

H	2.64187842	6.15738770	5.46340439	-0.00840458	-0.00298002	-0.00939230
O	3.15858067	6.79360193	4.85942737	0.00488582	-0.00995408	-0.00779604
H	6.92162570	4.91959129	8.33324420	-0.01483320	0.00142890	-0.00080625
O	7.91163008	4.71225042	8.59569078	-0.00893361	0.00157293	0.00019619
H	8.20910681	5.43618692	9.26334202	-0.00706442	0.00089517	-0.00472809
H	5.43698551	8.02170408	10.25912005	0.00560185	0.00217973	0.00828019
O	6.13206074	7.34295063	9.95400941	-0.00041170	0.00373549	0.01466879
H	5.56008189	6.47165428	10.02991087	0.00090559	0.00159464	0.00429915
H	3.41524549	8.04686983	9.58876855	0.00193636	0.00413878	0.00502797
O	3.96045859	8.74496804	10.10069917	-0.00248773	-0.00107500	0.00727683
H	3.53530923	8.75691788	10.99887565	-0.00372354	-0.00758137	-0.01336143
H	2.58991635	9.75149428	6.44041777	0.00173935	0.00010056	-0.00285594
O	1.96038384	10.49585297	6.16609634	0.00009411	-0.00330383	-0.00221982
H	1.11612694	10.02682177	5.97067877	0.00302883	0.01150667	-0.01164714
O	9.41813285	2.09936628	3.95191940	0.00196701	-0.00341127	-0.01264975
O	10.12781964	5.10639809	4.29928710	-0.00908633	0.00385436	-0.01440188
H	8.69939326	1.46413178	3.67106640	-0.00042417	-0.01138935	-0.00592657
H	10.03369758	2.18326611	3.18069129	0.00213616	-0.00741838	0.01056124
H	10.37772133	5.10694224	5.27813340	0.00728279	0.00790107	-0.01004760
H	10.31971794	6.05686386	3.98059792	-0.01466715	-0.01187195	-0.00619176
O	2.51042254	6.95434467	8.76426579	0.00733195	-0.01026630	-0.00039570
H	2.89727651	7.47868055	7.95075592	0.00782226	-0.00398146	-0.00513588
H	1.70836803	7.54978313	9.04065385	0.00441408	-0.00333287	0.00401698
O	4.42554994	5.46166953	10.24580568	-0.00139460	0.00237023	0.01085634
H	3.64353351	5.75900131	9.70189371	0.00309185	-0.00373833	0.00450413
H	4.46556310	4.46133136	10.20965425	0.00200665	0.00759491	0.01083411
H	7.08574236	4.82947849	6.13531823	-0.01074490	0.00922037	-0.00349530
H	8.29486288	6.17296527	5.83522140	-0.00378978	-0.00879954	-0.00571565
H	8.28972780	5.88384546	7.39434021	-0.00000846	0.00809916	-0.01363141

*HC(OH)$_2$;*OH

117

Lattice="9.84 0.0 0.0 0.0 8.52169 0.0 0.0 0.0 11.75" Properties=species:S:1:pos:R:3:forces:R:3

C	1.27845480	0.73514337	0.84286840	-0.00024547	-0.00108683	-0.00185343
C	1.28914923	2.16011636	0.77435505	0.00392250	-0.00395458	0.00023593
C	2.52161550	2.87398429	0.87624263	-0.00292171	-0.00636626	-0.01426539
C	2.52183397	4.29965217	0.91634063	0.00386692	0.00338813	-0.00886928
C	1.28881726	5.00724608	0.85039197	0.00023167	0.00174217	0.00486999
C	1.28632682	6.42272140	0.95116399	0.00247420	0.00265445	0.00323601
C	2.51927688	7.13106263	1.07279562	0.00042016	-0.00055714	0.00854921
C	2.50792464	8.55610263	1.00930106	0.00121684	-0.00008323	0.00861034
C	3.74055811	0.74850356	1.08568400	0.00023694	0.00016720	0.02079954
C	3.75351712	2.16913593	1.04078629	0.00848008	0.00078511	-0.00619777
C	4.95745028	2.87027456	1.36794400	0.02778956	-0.01416923	-0.03189797
C	4.82694505	4.23502341	1.77072517	0.00884181	0.00493011	0.00754545

C	3.70837367	4.99409848	1.31879565	0.00195716	0.03111541	-0.04058454
C	3.72916408	6.43084507	1.33202326	0.01366954	0.00317147	0.00518118
C	4.96417324	7.15390351	1.43592833	-0.01156135	-0.00809782	-0.01088338
C	4.96915887	8.55969406	1.20760711	0.00184209	-0.00313119	0.00126498
C	6.20362297	0.76025588	1.17556915	0.00057054	0.00730747	-0.00768501
C	6.21545033	2.17106824	1.35504058	0.00183450	0.01061230	0.01177580
C	7.48637547	2.85973868	1.37080046	-0.02049369	-0.00449422	0.00220500
C	7.68104595	4.17117018	1.93228731	-0.00570072	0.00431187	0.01811543
C	6.20963740	6.54717429	1.82290603	0.00197569	-0.01243250	0.00727795
C	7.43314931	7.14236278	1.35545086	0.01678360	-0.01061267	-0.05057511
C	7.42454428	8.54743109	1.05786367	0.00002827	0.00133746	-0.01699703
C	8.64216289	0.73128625	0.85443518	-0.00441021	-0.00984569	-0.00413588
C	8.64496728	2.16346853	0.89119246	-0.00906723	0.00411719	0.00250481
C	9.89290161	2.86887277	0.79571934	-0.00406669	0.00138216	0.00033237
C	9.89514570	4.29055519	0.89764942	-0.00977214	0.00291408	0.00253454
C	8.71858045	4.96925966	1.34393485	-0.00652437	0.01348917	-0.00622260
C	8.67242004	6.40886966	1.26568818	-0.00506525	-0.00676191	0.00277289
C	9.88278249	7.12448133	0.99437256	-0.00250939	0.00293623	0.01041286
C	9.88090395	8.54767703	0.86095927	-0.00452437	0.00104078	-0.00252594
Cu	6.07627500	3.54258118	4.86408374	0.03089600	-0.00206222	-0.01473564
Cu	5.05287032	5.81680499	4.90102856	-0.03010027	0.01769214	-0.00660021
Cu	6.69603309	7.21743127	3.67039623	-0.00754955	0.00565185	0.09705854
Cu	4.08924404	3.94958129	3.60593597	0.04608504	0.01199540	0.15046333
Cu	6.21926669	4.96846436	2.91054395	0.01347737	0.00271139	0.07474274
Cu	8.24823949	4.08541092	3.94598500	0.00570307	-0.01603639	0.04712768
C	6.26139094	5.48217149	6.44430155	-0.01647432	0.00263053	0.00857714
O	5.51220784	5.77602698	7.51192249	-0.01252044	0.01769564	0.01515037
O	7.42474665	6.43546875	6.33238972	0.01575888	0.00775205	0.00865176
H	4.08025790	3.39085387	8.49650677	0.00542678	0.00413255	-0.01658310
O	3.84177317	3.86201128	7.59094755	0.00031058	0.00161198	-0.00698155
H	4.51514764	4.69288835	7.58999666	0.00233892	0.00245761	-0.01730864
H	1.00467486	8.20607510	3.27849782	-0.00019816	0.00383120	0.00876972
O	1.03447619	7.73610580	4.14742123	-0.00792478	0.00680610	-0.02352946
H	2.00563485	7.41868391	4.33647060	0.01606449	-0.00455927	-0.01233549
H	1.98652402	2.31223837	5.58965633	0.00625356	0.00604976	-0.00470983
O	2.27325160	3.29418911	4.13156775	0.01289296	0.00209609	-0.01741125
H	1.55092581	4.05195916	4.06241059	-0.02355918	0.00261334	-0.00808789
H	7.58589659	3.20747382	9.37058559	-0.00608002	-0.00601449	-0.00045241
O	7.25034626	2.29462978	9.69079803	-0.00348686	-0.01873388	0.00429396
H	7.57118941	2.22774649	10.62590891	-0.00404719	-0.00504688	-0.00253468
H	3.42131793	-1.06345245	5.82350920	-0.00884060	0.00335767	0.00296315
O	3.58460073	-0.15432861	6.93246554	0.00861234	0.00294235	0.00002366
H	4.55083568	0.04248926	7.21481095	0.00119042	-0.00575078	-0.00776423
H	1.70999650	5.86261531	7.47066321	-0.00017076	-0.00028710	-0.01280338
O	1.80364588	5.23884809	6.68379218	-0.00297588	0.00286321	-0.00476375

H	2.49116735	4.56764869	7.04692318	0.01019835	0.00259941	-0.02181177
H	5.71189893	1.70373771	6.91394479	0.00305662	0.01206654	-0.00293297
O	5.23359923	2.29264608	6.26550395	0.01449723	-0.00057013	0.00350154
H	4.57872556	2.95967774	6.83377680	-0.00897966	-0.00192612	0.00029185
H	8.99634629	8.87812113	6.69903566	-0.00706281	0.00039982	-0.00892084
O	8.33313547	9.67048585	6.62201036	-0.00701661	0.00219725	-0.00007188
H	7.62994869	9.46422409	7.31148417	0.00059069	0.00426411	-0.00299266
H	4.35375555	8.54632528	4.14901674	0.00016127	0.00383174	-0.00834346
O	4.60341960	9.50312842	4.08580419	-0.02376963	-0.01547634	-0.01575944
H	4.59571454	9.85925343	5.03372405	-0.00879750	-0.01075223	-0.01725193
H	5.68681652	2.55764209	9.72291685	-0.00401468	-0.00827245	0.00127540
O	4.66645012	2.74706364	9.72749985	0.00563838	-0.00896780	0.01510690
H	4.25394694	1.82729858	9.81985494	0.00737184	-0.00346313	0.00286019
H	7.74301249	7.13809345	10.04016955	-0.00009914	0.00607604	0.00717491
O	8.70960376	6.80877493	9.90575182	0.00025891	0.00546229	0.00948239
H	9.00968823	6.61593493	10.83379663	-0.00315867	-0.00023507	0.00094093
H	-0.13321133	-0.39033099	9.50252466	-0.00153755	0.00516972	0.00207914
O	0.67508982	0.21229779	9.39499257	-0.00094882	0.00516876	0.01421953
H	0.53331318	0.67866568	8.53531029	0.00107103	-0.00181625	-0.00213870
H	9.07107706	6.92547064	6.75476827	-0.00299668	-0.00828196	-0.00413000
O	9.84984715	7.56073211	6.69555776	0.01112950	-0.01146557	-0.00459450
H	10.10298428	7.56166084	5.72670250	-0.00885435	-0.00405754	0.00204233
H	6.12361577	-0.46757594	8.53656997	-0.00137580	-0.00061418	-0.00604689
O	6.10098086	0.04126907	7.61608463	-0.00518703	-0.00333184	-0.00458382
H	6.43663231	-0.68146006	7.00008102	-0.00394920	0.00220347	-0.01796176
H	8.80662816	2.74533883	6.30083918	-0.01015775	0.00751376	-0.01297224
O	8.88225778	3.72302783	6.05489335	0.00399744	-0.00290677	-0.00194148
H	8.57935827	4.18886651	6.89242852	-0.00458217	-0.00031337	-0.02982038
H	7.56821399	0.72590686	5.28246426	-0.01059289	0.01402639	-0.00873229
O	7.25954478	0.52627308	4.31027367	0.00032668	-0.01311411	-0.01638253
H	6.32835319	0.93852286	4.18596696	0.00325727	0.02562971	-0.00425147
H	2.63162085	6.06734611	5.70866423	-0.00530518	-0.00017047	-0.00238611
O	3.17909028	6.73336965	5.09101157	0.00830867	-0.00400207	-0.00899111
H	6.94332051	5.02813365	8.46089804	-0.01061771	0.00284053	-0.00398232
O	7.85251768	4.63039579	8.63656637	-0.01233727	0.00696922	0.00024633
H	8.31425764	5.37959693	9.15319799	-0.00685505	0.00227112	-0.00864750
H	5.50802711	8.03900911	10.27940629	0.00685559	0.00135301	0.00612511
O	6.19943524	7.39440885	9.91326868	-0.00133501	0.00427134	0.01485007
H	5.64786319	6.51494273	9.95078537	-0.00097852	0.00286128	0.00505754
H	3.40503486	8.02594874	9.62082567	0.00287733	0.00292329	0.00507912
O	3.96823748	8.75257948	10.06146990	-0.00126056	-0.00277900	0.00561416
H	3.57960488	8.81906260	10.97387862	-0.00320198	-0.00829823	-0.01452796
H	3.01250923	9.19322334	6.70471875	0.00986904	-0.00321527	0.00314576
O	1.76187094	10.03575306	6.15758072	-0.00196798	-0.00215325	-0.00817010
H	1.34009209	9.44397867	5.47773812	-0.00037096	0.01132533	-0.00321311

O	9.52554887	2.06092904	3.75036203	-0.00145181	-0.00395324	-0.00905672
O	10.30280732	5.07854332	4.25763721	-0.00850705	0.00723660	-0.01853853
H	8.71620462	1.46932541	3.82428953	-0.00185038	-0.01143736	-0.00929251
H	9.58800273	2.17886711	2.76508993	0.00812853	-0.01171785	0.01547483
H	10.40285936	5.04281261	5.25911945	0.00804301	0.00795286	-0.01001746
H	10.48031050	6.04287918	4.00258054	-0.01286060	-0.01672621	-0.00974406
O	2.46046758	6.93328198	8.81760223	0.00990997	-0.00764009	0.00117640
H	2.89845123	7.41341531	8.03135738	0.00801302	-0.00724759	-0.00006838
H	1.68271113	7.58761761	9.04411207	0.00478114	-0.00379019	0.00374211
O	4.53444612	5.45321021	10.22886980	-0.00115765	0.00282904	0.01092625
H	3.71568108	5.73899660	9.73944884	0.00425930	-0.00379201	0.00530747
H	4.54590368	4.45210189	10.21208970	0.00334188	0.00818846	0.01062503
H	6.73466228	4.45907714	6.50120990	-0.00080735	-0.01674310	-0.01444808
H	7.61073938	6.36682557	5.34124929	0.01350579	-0.00856658	-0.00219915
H	1.98819005	2.62612514	3.45660149	-0.01586434	-0.01204303	-0.00817266

*$CH_2(OH)_2$;*OH (oxidated)

118

Lattice="9.84 0.0 0.0 0.0 8.52169 0.0 0.0 0.0 11.75" Properties=species:S:1:pos:R:3:forces:R:3

C	1.27788439	0.65684376	0.79896066	-0.00099502	-0.00316461	0.00176442
C	1.28394442	2.08013571	0.71984743	0.00176423	-0.00292457	-0.00180336
C	2.51768457	2.79251296	0.76932428	-0.00459890	-0.00652118	-0.01645827
C	2.51821873	4.21712041	0.82265370	0.00326856	0.00399385	-0.01285207
C	1.28260838	4.92755138	0.80424909	-0.00270890	0.00317789	0.00106326
C	1.28259542	6.34119202	0.91817118	0.00253598	0.00236668	0.00390745
C	2.51691809	7.05122128	1.00703653	0.00086401	0.00271927	0.00581957
C	2.50765496	8.47427462	0.92173676	-0.00029969	-0.00326075	0.00816586
C	3.73974186	0.66370173	0.96222497	0.00478135	-0.00429284	0.00893907
C	3.74578601	2.08596670	0.92228162	0.00966696	0.00082897	-0.01121193
C	4.95051791	2.78492653	1.24908900	0.02805695	-0.01368746	-0.03871555
C	4.83277617	4.14461322	1.66169110	0.00765700	0.00158071	-0.00178148
C	3.71297080	4.91248308	1.21507524	0.00382965	0.03280152	-0.04482443
C	3.72660458	6.35011028	1.25501767	0.01040069	0.00542604	0.00011165
C	4.96589619	7.07863186	1.37326405	-0.01052218	-0.00095552	-0.01761311
C	4.96445522	8.47848003	1.11568192	0.00560388	-0.00936058	0.01148589
C	6.19983063	0.67911091	1.11816660	-0.00096879	0.00025027	0.00702739
C	6.20299944	2.08664804	1.25810893	-0.00456393	0.00377767	0.00990878
C	7.46717014	2.78741047	1.27688934	-0.00828915	0.00416296	0.01864975
C	7.59461840	4.12422818	1.71100892	-0.01333062	-0.00305313	0.00251139
C	6.19778282	6.47794364	1.76334362	-0.00171539	-0.01039699	-0.00696971
C	7.42706123	7.07238187	1.34609006	0.00980764	-0.00362146	-0.05371891
C	7.43069078	8.47406509	1.06305230	0.00174653	0.00006717	-0.00750776
C	8.65489987	0.65533203	0.88829535	-0.00165039	-0.00531021	-0.00116887
C	8.65631691	2.08062778	0.89923409	-0.00512373	-0.00257154	0.00274174
C	9.89001345	2.79055086	0.78168571	-0.00412934	0.00304923	-0.00447631

C	9.88895469	4.21285744	0.86269122	-0.00658825	0.00286348	0.00425931
C	8.69570918	4.89321481	1.26068505	-0.00391227	0.00238578	-0.00619069
C	8.67282355	6.33679543	1.25278597	-0.00074402	0.00035148	-0.00253562
C	9.88323910	7.04744073	1.00153489	-0.00009818	0.00234106	0.01618696
C	9.88636243	8.47071385	0.87359414	-0.00286758	0.00014445	0.00250212
Cu	5.96643720	3.61853918	4.87772051	0.02543545	-0.05448436	-0.01449099
Cu	4.81921380	5.84200573	4.46345104	-0.06898182	0.04280434	0.02321059
Cu	6.98486809	6.88646874	3.79834041	0.01454729	0.01824858	0.08950423
Cu	4.00949491	3.78434750	3.46771798	0.03271352	0.01060997	0.14156772
Cu	6.21527102	4.85531185	2.81777896	0.00273895	0.00417779	0.07467881
Cu	8.03731320	4.72023997	4.40504019	0.02791531	-0.05529182	0.00344154
C	5.96398700	5.69907952	7.27058971	-0.00004702	0.00947043	0.01392766
O	6.23809550	5.03686852	8.46954046	-0.00254817	-0.00862284	0.00424302
O	7.04169374	6.57588935	6.88518651	0.01195445	0.01072420	0.01573116
H	4.09383864	3.39248246	8.59355049	0.00140822	0.00253976	-0.00869550
O	3.58926224	3.65121697	7.66624497	0.00412927	0.00864148	-0.00579583
H	3.85945969	4.58243061	7.49752368	0.00461485	0.00233508	-0.01625217
H	0.68451789	8.44322485	3.92605975	0.00012167	-0.01028345	-0.00873009
O	0.93062958	7.52534969	4.28527771	-0.00078767	0.00632681	-0.01927770
H	1.94696103	7.40175976	4.43081790	0.00916493	-0.00115221	-0.01156335
H	2.10419085	2.65188580	4.81391430	-0.00354411	-0.00017689	0.00254613
O	2.21292340	3.12508736	3.89373306	0.01468599	0.00436479	-0.01785990
H	1.52971424	3.87818304	3.91263488	-0.02152049	0.00596092	-0.00398850
H	7.98411835	2.79416324	9.39910096	-0.00329877	-0.00945097	0.00214969
O	7.32117022	2.12257900	9.76178631	-0.00326656	-0.01681146	0.00655806
H	7.49232638	2.20214549	10.73617671	-0.00158398	-0.00350575	-0.00724475
H	3.32401555	-0.72603917	6.16548161	0.01161220	-0.00739550	0.00091486
O	3.22772885	-0.05001561	6.99147115	0.00772475	0.00448877	-0.00007213
H	4.16885894	0.14275250	7.29560817	-0.00368708	-0.00344982	-0.00415208
H	1.68292747	5.74066391	7.42409682	-0.00465564	0.00046223	-0.00975032
O	1.53972659	5.16280516	6.59387889	-0.00206479	0.00296559	-0.00587296
H	1.86648083	4.26164378	6.87588838	-0.00359327	0.00573442	-0.01462969
H	5.58087143	1.70567752	6.82217767	0.00126901	0.01038289	-0.00058766
O	5.12245672	2.34397152	6.19310055	0.01161176	-0.00033168	0.00462637
H	4.42421276	2.89943516	6.79823730	-0.00892869	-0.00484885	0.00043590
H	8.85462493	8.87087104	6.59238890	-0.00928267	-0.00098628	-0.00644258
O	8.16786528	9.65368001	6.46901917	-0.00397527	0.00037511	0.00132732
H	7.48260642	9.51945183	7.18624819	0.00254867	0.00297907	-0.00371078
H	4.15475645	8.45948988	4.24535837	0.00959888	0.01647793	-0.01442907
O	4.61196669	9.32737098	4.09270419	-0.01649134	-0.01838341	-0.01489769
H	4.56635174	9.82408006	4.97440294	-0.00895368	-0.00761200	-0.01264433
H	5.68686752	2.69946677	9.68409903	-0.00807692	-0.00163520	0.00094768
O	4.70213135	2.92935137	9.78401055	0.01133854	-0.00109983	0.02104465
H	4.22839046	2.02445489	9.85640806	0.01261586	0.00063009	0.00439042
H	7.85456280	7.08047277	9.90517896	-0.00091929	-0.00098536	-0.00186109

O	8.82667219	6.78493739	9.93462216	-0.00624668	0.00285801	0.00995912
H	8.96437598	6.61677126	10.90718414	-0.00569156	-0.00007769	-0.00368205
H	-0.03719831	-0.41610406	9.66551659	0.00031659	0.00643044	0.00642352
O	0.72341326	0.20503561	9.38492536	-0.00718783	0.00544250	0.01150369
H	0.51823855	0.38577905	8.43230650	-0.00028068	0.00497117	0.00302141
H	9.26197628	6.94657796	6.90286140	0.00584668	-0.01132089	-0.00815037
O	9.82541654	7.73295505	6.69139006	0.00524659	-0.02025190	-0.00641203
H	10.19677642	7.57776673	5.72823751	-0.00821759	-0.00698094	0.00129965
H	5.98455362	0.02612929	8.64701003	-0.00430196	0.00061318	-0.00161575
O	5.87675551	0.26410454	7.65410814	-0.01082785	-0.00397615	-0.00020230
H	6.33033549	-0.53354948	7.20095574	-0.00020468	-0.00099675	-0.01399207
H	8.54247435	2.71788530	6.25029667	-0.00997314	0.00996032	-0.01227591
O	8.71138873	3.70648109	6.13279099	0.00064658	0.00095647	-0.00349754
H	8.82668227	4.05057348	7.07956828	-0.00932374	0.00316025	-0.02480738
H	7.48299191	0.62845702	5.13147528	-0.00756131	0.01329723	-0.00306526
O	7.19812880	0.34749049	4.17748502	-0.00090415	-0.00790042	-0.01356069
H	6.19822142	0.61246179	4.08447651	-0.00512091	0.03331559	-0.00123357
H	2.72516797	6.10781430	5.57938624	-0.00940989	-0.00472582	0.00178016
O	3.26503996	6.81848289	5.10544434	0.00848706	-0.01942475	-0.00461667
H	7.73716696	4.58097748	8.67088695	-0.00434751	0.00385866	-0.00357967
O	8.69760320	4.25171959	8.74891335	0.00450684	0.00965508	0.00338594
H	9.09389053	5.03018609	9.23531564	0.00148226	0.00047620	-0.00145629
H	6.33055235	8.37562162	10.78489827	-0.00392452	0.00387165	-0.00475292
O	6.28860433	7.75398363	10.01204151	-0.00575821	0.00775034	0.01785713
H	5.51978347	7.11734927	10.21353403	-0.00144447	0.00797014	0.01097922
H	2.50187069	9.18794839	9.60890659	0.01179947	0.00124770	0.00910716
O	3.48860916	9.12417084	9.76972881	0.00829898	-0.01559221	0.00587954
H	3.51535278	8.84667758	10.72242531	0.00187955	-0.01160633	-0.01336152
H	2.25231352	9.71264045	6.41910825	0.00070130	-0.00090365	-0.00444477
O	1.65256770	10.47309349	6.10786343	-0.00269775	-0.00826489	0.00026819
H	0.86085931	10.07012445	5.66697401	0.00195709	0.00271405	0.00417264
O	9.73002028	1.37108911	3.90485745	-0.00859455	0.01097743	-0.01885805
O	10.15135394	5.00476233	4.21471515	-0.00734045	-0.00084024	-0.01833966
H	8.77207167	1.18740911	3.66834170	-0.01009587	0.00917487	-0.00695658
H	10.09051717	1.99373402	3.22593250	-0.01193636	0.00651144	0.00458947
H	10.42638011	5.00526044	5.19332171	0.02381566	0.00931756	-0.00486079
H	10.35535987	5.96538592	3.94963297	0.00351637	0.00077395	-0.01305497
O	2.34631121	6.67788568	8.61429648	0.00303976	-0.00099607	0.00366398
H	2.76624264	7.40705361	7.98332179	0.00428881	-0.00153161	-0.00464792
H	1.66631893	7.28257570	9.06504851	0.00288587	-0.00700277	0.00544792
O	4.37622488	5.93441469	9.99360244	-0.00422876	0.00514188	0.01790197
H	3.49441650	6.24309287	9.50885622	0.00254750	0.00186274	0.00556143
H	4.13001316	5.21350487	10.63044500	0.00245326	-0.00053214	-0.00212855
H	5.83929788	4.96221926	6.42166903	0.01079323	-0.01229693	-0.00578288
H	7.17710910	6.38180979	5.86997058	-0.00710941	0.00574169	0.01292695

H	5.02123766	6.30112917	7.36935805	-0.02067009	0.00241435	-0.02088707
H	5.51149147	5.32782229	9.14494304	0.01047106	-0.00260295	-0.00101903

*CH$_2$O;*OH

118

Lattice="9.84 0.0 0.0 0.0 8.52169 0.0 0.0 0.0 11.75" Properties=species:S:1:pos:R:3:forces:R:3

C	1.28242745	0.72766780	0.79022104	-0.00103740	-0.00507620	-0.00374895
C	1.29268416	2.15389242	0.74785814	0.00202530	-0.00356968	-0.00149784
C	2.52245244	2.86640336	0.86787170	-0.00007665	-0.00443350	-0.00735934
C	2.52064873	4.28866482	0.92113777	0.00162816	0.00173137	-0.00237912
C	1.29162009	5.00052513	0.81910092	0.00067612	0.00184415	0.00405101
C	1.28878045	6.41636672	0.90102851	0.00234692	0.00335043	0.00520413
C	2.51912370	7.12546409	1.02952976	0.00178205	-0.00092388	0.00998314
C	2.51013411	8.54764874	0.96007080	-0.00084397	-0.00219017	0.00999138
C	3.74234908	0.74184723	1.04208008	0.00161174	-0.00127960	0.01569669
C	3.75358743	2.16318577	1.02670438	0.00867178	0.00292248	-0.00714237
C	4.95380085	2.85768319	1.38040073	0.02485572	-0.01009488	-0.02505598
C	4.80735087	4.20745972	1.83993247	0.01201833	0.00125059	0.01907857
C	3.70205860	4.98229564	1.34627986	0.00047298	0.02644369	-0.01839301
C	3.73257221	6.42382671	1.31592753	0.01374627	0.00187253	0.01118508
C	4.96627194	7.15483482	1.41516618	-0.00917857	-0.00584253	-0.00790778
C	4.97004240	8.55503771	1.16598719	0.00311751	-0.00681625	-0.00175164
C	6.20522441	0.75755237	1.13730621	0.00032290	0.00513446	-0.01148554
C	6.21736867	2.16474721	1.33612307	0.00110238	0.01194394	0.00672293
C	7.49215901	2.84928564	1.35359915	-0.01764342	-0.00709506	-0.00337479
C	7.68128117	4.17215090	1.89631362	-0.00922304	0.00282123	0.02542567
C	6.21896949	6.55993328	1.82063729	0.00313582	-0.01234726	0.00733339
C	7.43691150	7.14290660	1.30148804	0.01301588	-0.01167576	-0.04732717
C	7.42663053	8.54479501	1.00461197	-0.00031390	0.00047509	-0.02074505
C	8.64649555	0.72912763	0.80638898	-0.00304738	-0.00846512	-0.00073515
C	8.65154034	2.15717739	0.87398778	-0.01058723	0.00209456	-0.00265383
C	9.89777723	2.86176426	0.77863295	-0.00497836	0.00052727	-0.00505564
C	9.89805254	4.28447262	0.86142244	-0.00998853	0.00171733	-0.00166363
C	8.71860657	4.96877008	1.28780195	-0.00844114	0.01801219	-0.01121038
C	8.67094090	6.40366809	1.19617073	-0.00722821	-0.00756927	0.00297471
C	9.88320251	7.11934758	0.91787001	-0.00353526	0.00570011	0.01229621
C	9.88592057	8.54176522	0.79552292	-0.00195070	0.00205434	0.00276016
Cu	6.15719918	3.89909925	5.14510493	0.00846450	-0.05231223	-0.00133995
Cu	5.01336145	5.97884744	4.82358496	-0.00169846	0.01988752	0.01426360
Cu	6.77971346	7.24605107	3.61534450	-0.01348315	0.03083060	0.08352469
Cu	4.16323244	3.92505566	3.69655206	0.03733655	0.00972738	0.10206622
Cu	6.22677062	4.97851069	2.87991038	0.01301193	0.00533518	0.05924520
Cu	8.23357180	4.02199952	3.83864318	0.01513044	-0.06914826	0.03174058
C	6.46622611	5.69741657	6.26868236	-0.00751968	-0.00007462	0.01230573
O	4.07495013	5.87550486	8.03729890	0.00649372	0.01409497	-0.00569722

O	7.81537590	6.20289838	6.06506329	0.02480899	0.02653892	0.01344071
H	3.81658553	3.09784109	8.45280143	0.00110846	-0.00168137	-0.01336534
O	3.40620649	3.46151941	7.59052052	0.00242700	-0.00557528	-0.00946123
H	3.73368183	4.44557770	7.65388294	0.01254665	0.00094170	-0.02259197
H	0.72535181	8.45669909	3.76466168	-0.00036417	-0.00909861	-0.00826821
O	0.92287157	7.55221405	4.15526410	-0.00804108	0.00619079	-0.01808735
H	1.94611176	7.38352624	4.27656992	0.01056787	-0.00147321	-0.01209345
H	2.10432601	2.78706630	4.98255572	-0.00211955	0.00245835	-0.00166872
O	2.37426844	3.28757691	4.11128430	0.00958931	0.00184214	-0.01836491
H	1.65929990	3.99316131	4.00353447	-0.02479397	0.00153545	-0.00741875
H	7.85116541	3.17656792	9.38886556	-0.00196479	-0.00871570	0.00280500
O	7.41022121	2.27515885	9.56525179	-0.00155252	-0.01948379	0.00320935
H	7.77589504	1.99539617	10.44201702	-0.00387781	-0.00774634	-0.00152140
H	3.26915677	-0.69267406	5.97016422	0.00803578	-0.00328605	0.00553458
O	3.18160297	0.06497239	6.71161625	0.01556396	0.00344748	-0.00148091
H	4.08774446	0.17272722	7.11063216	-0.00307350	-0.00532039	-0.00507048
H	1.45729967	5.84245456	7.42068206	0.00139615	-0.00758898	-0.01062059
O	1.45747607	5.18387339	6.67295723	-0.00264001	0.00664668	-0.00518259
H	1.90661874	4.36239862	7.04032652	0.00223584	0.00296806	-0.01688959
H	5.65113283	1.76251779	6.91891905	0.00294760	0.01603992	-0.00607626
O	5.23171373	2.39111647	6.26276642	0.01466475	-0.00215718	0.00667207
H	4.40782439	2.84697586	6.78730231	-0.00715278	-0.00074276	-0.00531577
H	9.07086993	8.77646078	6.67273492	-0.00530164	-0.00572028	-0.00706321
O	8.31280450	9.55031458	6.60090170	-0.00216232	-0.00274026	0.00278243
H	7.65421668	9.42186298	7.34071003	0.00495100	0.00161557	0.00056689
H	4.28090076	8.65551574	4.21009001	0.01307749	0.00318606	-0.01251670
O	4.73080612	9.53260069	4.11028799	-0.01846631	-0.00953331	-0.01395481
H	4.67909921	9.98185109	5.02314744	0.00263381	0.00004538	-0.01442593
H	5.86472287	2.49169548	9.61366400	-0.00752377	-0.00662900	-0.00094127
O	4.84638334	2.68568928	9.66121451	0.00334892	-0.00832816	0.01180604
H	4.42324711	1.78090456	9.85664789	0.00545485	-0.00220205	0.00449157
H	7.73835363	7.31211999	9.99412669	-0.00278816	0.00864911	0.00795858
O	8.70792317	7.05754160	9.79878387	0.00035065	0.00915950	0.00618222
H	9.11480815	6.96240006	10.70230388	-0.00231286	0.00083349	-0.00254039
H	-0.41701712	0.10565859	9.40099520	-0.00333760	0.00570349	0.00041841
O	0.30122903	0.81066654	9.36854733	-0.00560194	0.00607629	0.01215961
H	0.15993534	1.27770254	8.50903915	-0.00243181	0.00052389	-0.00350178
H	9.27000710	6.98083230	6.69746298	-0.00297203	-0.00041414	-0.00298780
O	9.96233721	7.70494911	6.66597420	0.00762848	-0.00229191	-0.00173618
H	10.29312026	7.65653668	5.68809257	-0.00622911	-0.00129964	0.00414073
H	6.07860130	-0.39997613	8.49124683	-0.00246894	0.00383574	-0.00548739
O	5.96544618	0.18218293	7.62867657	-0.00642714	-0.00147468	-0.00215520
H	6.26219241	-0.40595630	6.89108294	-0.00481535	-0.01437158	-0.01697347
H	8.52367340	2.49756163	6.24024669	-0.01093237	0.02197797	-0.01396359
O	8.48766894	3.46187526	5.88277853	0.00212302	0.00059230	-0.00804820

H	8.83451612	4.05246815	6.60183520	-0.00443125	-0.00993238	-0.02388134
H	7.58320451	0.68713674	5.23353900	-0.00595484	0.01238233	-0.00672893
O	7.26989933	0.54925810	4.25853092	0.00070342	-0.00815404	-0.01252046
H	6.28400973	0.88719268	4.17282799	0.00163850	0.03953432	0.00125485
H	2.74738743	6.12615199	5.48288231	-0.00568655	-0.00691939	-0.00390600
O	3.22313012	6.80584497	4.92050484	0.00291280	-0.01657765	-0.00385137
H	7.57667722	4.92221717	8.23892459	-0.01662616	0.00521167	-0.00693934
O	8.36129112	4.61640042	8.75967006	-0.00916092	0.00789817	-0.00279128
H	8.62126437	5.47763405	9.23972760	-0.00242990	0.00025481	-0.00467715
H	5.24198385	8.02288650	10.07399558	0.00655030	-0.00189996	0.00668564
O	6.13403216	7.48015052	9.87654212	0.00262283	0.00002782	0.01293596
H	5.83795592	6.53335235	10.08348503	0.00476930	0.00365187	0.00828016
H	3.14612320	8.13026938	9.50031635	0.00514268	-0.00238325	0.00474923
O	3.94140384	8.72425614	9.98282842	0.00086963	-0.00024239	0.00860686
H	3.62770153	8.79316415	10.92193474	-0.00061629	-0.00634841	-0.01294529
H	2.09071031	9.77528411	6.31952954	-0.00130894	-0.00028669	-0.00483142
O	1.40943061	10.49417715	6.09177733	-0.00772627	-0.00524700	0.00316008
H	0.79423336	10.11134337	5.40974456	-0.00231182	0.00606561	0.00015417
O	9.75010810	1.51623337	3.75301698	0.00097481	0.00092238	-0.01655893
O	10.15892675	5.01036510	4.23879189	-0.00645566	-0.00212477	-0.01731068
H	8.78529490	1.25187500	3.63373017	-0.01112470	0.00485676	-0.00723165
H	10.02998043	1.98001371	2.92281242	-0.00503268	0.01169221	0.02083773
H	10.42036609	5.04886928	5.21741474	-0.00280076	0.00119809	-0.01099509
H	10.31327602	5.96731778	3.93184866	-0.01468205	-0.00821046	-0.00860463
O	2.10247822	7.48658359	8.85007338	-0.00243326	-0.00763350	0.00810104
H	2.44418108	7.78338547	7.93290045	-0.00047605	0.00024722	-0.00252849
H	1.37127797	8.18338131	9.02753664	0.00460767	-0.00677429	0.00072002
O	4.87232775	5.18515137	10.39591554	0.00608594	0.00264118	0.01945402
H	4.43626005	5.33754167	11.27891607	0.00768853	0.00157707	-0.00120430
H	4.86986490	4.16465237	10.27274874	0.00697211	0.00287868	0.01347509
H	6.44546813	4.92692483	7.09492906	-0.00962762	-0.00059650	0.00942505
H	4.13381453	5.71216462	9.04480830	-0.00409582	0.01051968	-0.01245741
H	5.02962319	5.98981095	7.81917350	-0.00285212	0.00170892	-0.01717704
H	5.86006916	6.56134900	6.64568698	0.00236625	-0.00276525	-0.01181215

*CH_3OH

121

Lattice="9.84 0.0 0.0 0.0 8.52169 0.0 0.0 0.0 11.75" Properties=species:S:1:pos:R:3:forces:R:3

C	1.06449942	0.67814969	0.80275366	-0.00108634	-0.00298506	-0.00430643
C	1.07000188	2.10176019	0.70497578	0.00183292	-0.00631565	-0.00354621
C	2.29513259	2.81347338	0.88288365	0.00533385	0.00178687	0.00202879
C	2.29721475	4.23684817	0.94619791	0.01284005	0.00565761	0.01146475
C	1.07984145	4.94685193	0.75470528	-0.00251302	0.00646232	-0.00016013
C	1.07510022	6.36259518	0.87618529	0.00515240	0.00199331	0.00471400
C	2.30046145	7.06298817	1.08201755	0.00366027	-0.00494117	0.00664903

C	2.28439222	8.48995221	1.01751083	0.00297225	-0.00548607	0.00477848
C	3.51303344	0.68089520	1.12839837	-0.00048146	-0.00426758	0.01985330
C	3.51415600	2.09998913	1.07668845	0.00553171	0.00567687	-0.00335379
C	4.71765882	2.80229919	1.38683489	0.01510121	-0.00377849	-0.03631562
C	4.57584195	4.13882289	1.84533449	0.01390567	0.00829585	0.00608333
C	3.46847786	4.91730588	1.41316792	0.00294112	0.02048207	-0.00444149
C	3.50612921	6.36006536	1.39700912	0.01615789	-0.00405525	0.00733788
C	4.74652971	7.09445874	1.50670479	-0.00729632	-0.00983899	-0.00184561
C	4.74414001	8.49698320	1.27180244	0.00783474	-0.01034604	0.01437773
C	5.98110715	0.69719492	1.16979819	0.00271628	-0.00562704	-0.00499462
C	5.98635307	2.10893450	1.26470882	0.00521144	0.00751991	-0.00184161
C	7.24468870	2.79975307	1.13144023	-0.01302660	0.00681184	-0.00084334
C	7.40309521	4.15135473	1.53944557	-0.01143238	0.00385246	0.01065506
C	6.01606365	6.49347305	1.80545432	-0.00236523	-0.00716964	0.00168988
C	7.22635114	7.09943057	1.32082024	0.00522189	-0.00559700	-0.02068350
C	7.21198548	8.50397967	1.03482477	-0.00651241	-0.00103432	-0.01513630
C	8.42875140	0.67544652	0.78713591	-0.00438818	-0.01143481	0.00438743
C	8.42969840	2.09847954	0.74035958	-0.00827670	0.00419535	0.00364917
C	9.67412993	2.81061925	0.61693851	-0.00998585	0.00199835	-0.01588508
C	9.68492091	4.23536547	0.65926118	-0.01103586	0.00413943	-0.02238618
C	8.47917466	4.92736135	1.02964602	-0.01529742	0.00918718	-0.01369203
C	8.45393276	6.36723938	1.09374800	-0.00879904	0.00125395	-0.01755760
C	9.67118626	7.07298839	0.89021339	-0.01229493	-0.00594562	0.01345754
C	9.66896792	8.49546369	0.79623823	-0.00489070	0.00009798	0.00153567
Cu	6.77410483	3.95101283	4.96938467	-0.02239767	-0.06821637	-0.01525380
Cu	5.12080200	5.67459296	4.79554090	0.01176356	0.00241696	0.04083061
Cu	6.83507008	6.97720251	3.61715842	0.00843609	0.03834107	0.05329214
Cu	4.55671546	3.49010541	3.94453332	0.03405257	0.02175276	0.02126398
Cu	6.09174044	4.82931497	2.74936441	0.01236911	0.00603372	0.06209576
Cu	8.40511539	4.51020859	3.27592469	-0.03177650	-0.01860037	0.06045840
C	6.53989353	7.17853593	5.53669524	-0.00426958	0.00904820	-0.01644119
O	5.44687637	5.06271136	7.97244473	0.00557021	0.00977639	-0.00768557
O	7.50936722	6.54978275	6.46574693	0.02344697	-0.00105111	0.01751496
H	3.34362082	3.16011105	8.03907963	0.00050124	0.00413092	-0.01355327
O	3.12360716	3.91811727	7.42774865	0.01590955	0.00436088	-0.00057411
H	4.00694501	4.41536852	7.42370226	0.01579424	0.00689204	-0.02379659
H	0.77363076	8.42287710	3.20634443	-0.00826756	-0.00216967	0.00703798
O	0.94929935	8.01391263	4.09266849	-0.00765076	0.00758319	-0.01954388
H	1.81735329	7.47433419	4.02503574	0.00785513	-0.00208662	-0.01113334
H	2.46669096	2.33429496	5.13487989	-0.00318623	0.00597006	-0.00484893
O	2.75194645	3.27309480	4.84466857	0.00364462	0.00784260	-0.02263083
H	2.92354804	3.60943164	5.78170616	0.00023356	0.00537010	-0.00002424
H	7.51955495	3.08633480	9.43865022	-0.00682274	-0.00235261	0.00167027
O	7.06880324	2.23411258	9.76288190	-0.00043335	-0.01226336	0.00978686
H	7.21650373	2.26282825	10.74491185	-0.00575610	0.00097756	-0.00552099

H	3.77154920	-1.29081508	6.30984142	0.00179586	0.01106578	0.01601342
O	3.62669001	-0.74380279	7.12346215	-0.00094061	-0.00477738	-0.00426791
H	4.58167422	-0.55988989	7.50051750	-0.00201567	0.00238853	-0.00589676
H	0.91809725	6.36785077	6.85511525	0.00150884	0.00138090	0.00178502
O	1.41305451	5.59050976	6.44102313	-0.00294210	0.00638863	0.00521880
H	1.85602357	5.00557762	7.13002152	-0.00253633	0.01063889	-0.00640939
H	5.16267848	2.04006193	7.63001667	0.00651856	0.00250833	-0.01892524
O	5.59647224	1.91445786	6.71337770	-0.00508084	-0.00927466	0.00667878
H	5.56657040	2.83034217	6.27205870	0.00010704	0.00511807	0.01724201
H	8.75363053	9.16762200	6.86241717	-0.01147432	0.00215500	-0.00243993
O	8.22633194	9.91281320	6.44659033	-0.00134650	-0.01162363	0.00300274
H	7.30278432	9.99273328	6.85202484	-0.00412482	0.00079771	-0.00275370
H	4.46445649	8.92672190	3.93990790	0.00792264	-0.00644039	0.00028927
O	5.01849024	9.69306006	4.22665148	-0.00822892	-0.00940156	-0.00935028
H	5.00841243	9.71755460	5.23603656	0.00539611	-0.01373561	-0.00201532
H	5.61664474	2.32920637	9.48219320	-0.00328150	-0.00410796	0.00187478
O	4.59098925	2.35304448	9.19137834	0.00170926	-0.00483284	0.00843383
H	4.25817698	1.44243196	9.50272914	0.00386974	-0.00272546	0.00482553
H	7.73584866	7.00348835	10.15442742	0.00053788	0.00425435	0.00776350
O	8.65437625	6.81330568	9.77624912	-0.00397978	0.00365262	0.00600410
H	9.16502826	6.63227840	10.61184722	-0.00177898	-0.00133340	-0.00234178
H	-0.08953198	-0.25183555	9.35169712	-0.00317218	0.00364314	0.00145596
O	0.81202768	0.17278794	9.24003927	0.00207787	0.00195720	0.01227389
H	0.83699077	0.38064460	8.27085635	0.00862126	0.00038528	0.00257230
H	8.85079896	7.20433008	6.60740860	-0.00325808	-0.00837575	-0.00936840
O	9.73956459	7.72544181	6.72366861	-0.00044945	-0.01295475	-0.01399195
H	9.98731320	7.93821753	5.78212156	-0.01290248	-0.00471560	-0.00371192
H	6.01579082	-0.91514508	9.07924019	0.00058807	0.00583972	-0.00631203
O	5.99523363	-0.42596217	8.18497752	0.00732713	0.01178676	0.00494499
H	6.72936307	-0.86457083	7.68295395	0.00218341	-0.00015745	0.00665745
H	8.70796451	2.97847209	6.22385377	0.01742557	0.00209255	-0.00617493
O	8.51003619	3.97131280	6.23934068	-0.00565653	0.00075171	-0.00334465
H	8.36298157	4.15517443	7.24368940	-0.00664948	0.00312643	-0.01750442
H	7.96431152	0.94930040	4.84874249	-0.00962712	0.00288663	-0.02074979
O	7.85749915	0.79370988	3.84969268	-0.00410946	-0.00723342	-0.01439304
H	6.92086451	1.11141300	3.69596140	-0.00085477	0.03946400	-0.00051202
H	2.36157915	5.99272016	5.33149837	0.01777061	-0.00239209	-0.01079705
O	2.95230403	6.37601493	4.54403374	0.00839096	0.00512699	-0.01761255
H	2.85242670	5.68073585	3.83422869	-0.01876183	-0.00488405	-0.00946607
H	6.96345223	4.75174797	8.42309307	-0.00945200	0.00295887	-0.01411269
O	7.91035582	4.48651198	8.70138823	-0.01258534	0.01122538	0.00378732
H	8.30814881	5.32814474	9.11696931	-0.00867192	0.00488846	-0.00366203
H	5.28945645	7.60600760	10.65891100	0.00735859	-0.00409011	-0.00336529
O	6.04094758	6.93726251	10.48070279	-0.00485463	-0.00829460	0.01747418
H	5.58696567	6.06070757	10.63796815	0.00541562	0.00012350	0.01809358

H	3.34318564	7.76139024	9.83868591	0.00347973	-0.00155647	0.00599193
O	3.96885395	8.51163521	10.16276074	-0.00152020	-0.00303899	0.00723763
H	3.55872773	8.80227762	11.02254455	-0.00064722	-0.00749058	-0.01453013
H	2.66339024	9.03904545	6.46616124	0.00547345	-0.00258459	-0.00103508
O	1.96549235	9.65474769	6.07669702	0.00180632	-0.00909201	0.00172589
H	1.56209480	9.13065380	5.32718777	0.00118382	0.00032037	0.00081192
O	9.67463342	2.68877752	3.71513523	0.00665861	-0.00293395	-0.01278511
O	10.01258389	5.40437542	4.05664809	-0.00307328	0.00239674	-0.01525586
H	8.98834050	1.95828554	3.54663208	-0.00327674	-0.00944487	-0.00368834
H	10.29890181	2.65343800	2.94930594	0.00764360	-0.00767021	0.01130122
H	10.21210480	5.10453736	4.99239249	0.00288255	0.00311808	-0.00102595
H	10.02786468	6.40216730	4.07753798	-0.01104913	0.00221665	-0.00289343
O	2.40900247	6.62095205	9.22284628	0.00495857	-0.01176845	0.00480725
H	2.82436219	6.85089735	8.32970486	0.00616525	-0.00189864	-0.00039123
H	1.66895033	7.34711552	9.27531674	0.00540541	-0.00685020	0.00576238
O	4.26303282	4.86017309	10.20373623	0.00043645	0.00287100	0.02406905
H	3.37808795	5.29461623	10.00283286	0.00353768	0.00072313	0.01088283
H	4.16448472	3.86073073	10.08432004	0.00467404	0.00755881	0.01435091
H	5.56249990	6.01562949	7.72949122	-0.00960280	-0.02392851	-0.01189732
H	4.97761431	5.07367143	8.91497769	0.00586242	0.00810060	-0.01399231
H	7.73606809	5.65044489	6.07833584	-0.00442720	0.01830620	-0.01925246
H	5.52364506	6.95703162	6.02523983	-0.00274212	-0.01066725	-0.01542075
H	6.53989353	7.62853593	6.15669524	-0.00426958	0.00904820	-0.01644119
H	7.04989353	6.69853593	5.22669524	-0.03260058	0.25896473	-0.00956870

*CH$_2$;*OH

120

Lattice="9.84 0.0 0.0 0.0 8.52169 0.0 0.0 0.0 11.75" Properties=species:S:1:pos:R:3:forces:R:3

C	1.09644869	0.74314041	0.84237733	-0.00201361	-0.00457830	-0.00274332
C	1.10043790	2.16621934	0.76400269	0.00178790	-0.00630250	-0.00317933
C	2.32659212	2.87635569	0.93839698	0.00503508	0.00238717	0.00279416
C	2.33505309	4.29939560	0.97244099	0.01424205	0.00627288	0.00903557
C	1.11941856	5.01454126	0.78966711	-0.00226413	0.00578914	0.00009818
C	1.11069035	6.42832842	0.90684069	0.00182734	0.00355222	0.00321194
C	2.33507331	7.13192818	1.09262467	0.00181292	-0.00431502	0.00805597
C	2.31956387	8.55669728	1.04273225	-0.00041145	-0.00524603	0.00863872
C	3.54551524	0.74783678	1.16419687	0.00130725	-0.00192283	0.01917560
C	3.54505924	2.16834305	1.13262653	0.00655678	0.00310253	-0.00514472
C	4.75382613	2.87473622	1.43342775	0.01865913	-0.01262666	-0.05320951
C	4.61464676	4.22653540	1.87339512	0.01360547	0.00282362	0.00975908
C	3.50464062	4.98838097	1.41035700	0.00396108	0.01950368	-0.00784050
C	3.54114594	6.42344860	1.35857104	0.01598625	-0.00555402	0.00703677
C	4.78228685	7.14654253	1.40745696	-0.00572223	-0.01138958	-0.01985931
C	4.78084002	8.56189209	1.26177498	0.00266292	-0.00424103	0.00684518
C	6.01436762	0.76572645	1.16110494	0.00160292	0.00191471	-0.00929066

C	6.02088423	2.18106390	1.28703624	0.01040213	0.00677708	-0.00600994
C	7.26684024	2.87363869	1.15976786	-0.01781891	0.00540065	-0.00274780
C	7.45088463	4.23248056	1.57482512	-0.01337663	0.00588261	0.01431576
C	6.05017589	6.52819008	1.62442279	0.00206852	-0.01775676	-0.00533422
C	7.26158663	7.15728791	1.23315329	0.00630402	-0.01249843	-0.02065395
C	7.24046698	8.57545118	1.00365830	-0.00358173	-0.00062373	-0.01927788
C	8.46072946	0.74782447	0.79975228	-0.00553762	-0.01348597	0.00614706
C	8.45884076	2.17197817	0.77566985	-0.01008482	-0.00039825	-0.00149253
C	9.70876054	2.87587587	0.67557947	-0.00674987	-0.00023520	-0.02091681
C	9.72965213	4.29962298	0.71174999	-0.00964941	0.00470261	-0.02261088
C	8.52705501	5.00268161	1.04329831	-0.01765058	0.01356317	-0.02342598
C	8.49845079	6.43103720	1.05402465	-0.00804879	0.00207752	-0.01811958
C	9.71248542	7.14119959	0.89264608	-0.01261902	-0.00399922	0.01237640
C	9.70361035	8.56287093	0.81369633	-0.00479065	-0.00304165	-0.00260260
Cu	6.73241483	4.12963563	4.98730169	-0.00686699	-0.02538220	-0.03720752
Cu	5.01383102	5.85241299	4.61551152	-0.02550595	-0.01344235	0.04409436
Cu	6.76776773	7.29194416	3.51907814	0.02167762	0.01515796	0.02766902
Cu	4.56904995	3.58635864	3.81842504	0.03284611	0.02786722	0.06223432
Cu	6.15373745	5.01714346	2.73994913	0.01063547	0.01380860	0.06433911
Cu	8.43391376	4.34550457	3.29730462	-0.03827076	-0.02412302	0.07662119
C	5.24817911	7.81475066	4.88814091	-0.00809554	-0.00709435	-0.00155651
O	5.20441575	5.32479030	7.80045988	0.00590611	0.01118910	-0.00052548
O	7.45193290	6.54386413	6.64243240	0.00427131	0.00735801	0.01022934
H	3.18472715	3.07137403	8.01010338	0.00050932	0.00275856	-0.00964910
O	3.00287998	3.87117208	7.45604048	0.01758551	0.00312038	0.00419103
H	3.87032679	4.39845054	7.52446129	0.01463809	0.00879960	-0.01577770
H	0.55749008	8.32448120	3.34432212	-0.00738288	-0.00508032	-0.00155596
O	0.89445554	7.93321759	4.18768549	-0.00709952	0.00629112	-0.02023126
H	1.74420941	7.38166766	4.02352381	0.00944735	-0.00215561	-0.01179776
H	2.82710186	2.11292186	5.20454720	0.00569294	0.00197018	-0.00365811
O	2.95745520	3.08595318	4.86455729	0.00674505	0.01133090	-0.02216403
H	2.99761371	3.50704575	5.78353357	0.00260532	0.00722261	0.00014369
H	7.61324633	3.01421214	9.48677693	-0.00569200	-0.00380289	0.00209429
O	7.23695254	2.09707881	9.69797408	-0.00112350	-0.01802838	0.00615882
H	7.56410953	1.89695335	10.60861182	-0.00541218	-0.00833205	-0.00846744
H	4.46800297	-1.04290392	5.61880219	0.01686894	0.00057442	-0.01489968
O	3.60045626	-1.04576853	7.36845835	-0.00352417	-0.00592761	-0.00018318
H	4.44735249	-0.54756221	7.71244901	0.00301092	-0.00174646	0.00187769
H	0.91911432	6.30352804	6.89799018	0.00490358	0.00302583	0.00359278
O	1.31563908	5.48428398	6.45880594	0.00209566	0.00796745	0.00498888
H	1.79144288	4.89801830	7.12764986	0.00029587	0.01453985	-0.00443438
H	5.09070217	2.17079061	7.79977071	0.00901735	0.00349990	-0.01991784
O	5.41737086	2.28371834	6.84481339	-0.01112900	-0.00322842	0.00726651
H	5.43188284	3.26588162	6.71232675	-0.00887318	-0.00475447	-0.00152605
H	8.75145878	9.15302737	7.06086976	-0.01471051	0.00175872	-0.00105238

O	8.04404120	9.72679046	6.65068866	-0.01585676	-0.01015311	-0.00261702
H	7.25574191	9.68290579	7.26978110	0.00570893	-0.00097387	-0.00758044
H	4.73492672	8.48110051	4.16175272	-0.02962553	0.02124943	-0.00693514
O	5.95882486	10.53648006	4.30063005	-0.00199324	0.00779844	-0.01392982
H	5.72775460	10.29678988	5.24773064	0.00871667	-0.02942845	0.00411668
H	5.68662775	2.27171275	9.60623932	-0.00423955	-0.00424041	0.00364430
O	4.66820526	2.36091155	9.39378879	0.00758047	-0.00588893	0.01591263
H	4.32906694	1.42422636	9.66588081	0.00276262	-0.00179026	0.00417411
H	7.49726728	7.01330012	10.04800696	0.06769355	-0.00310788	-0.00599018
O	8.53745153	6.77195558	9.75532743	0.00528279	0.00837725	0.00469610
H	8.95436822	6.56867590	10.62931589	-0.00154950	0.00122528	-0.00016152
H	-0.20945924	-0.36010706	9.45870907	-0.00737108	0.00412423	0.00354263
O	0.65258696	0.16407098	9.44879747	-0.00064938	0.00238943	0.01616717
H	0.79311902	0.32124802	8.48254928	0.00768379	0.00069675	0.00491854
H	8.93614880	7.15023756	6.86217332	-0.00653780	-0.00634500	-0.00733855
O	9.80064566	7.68659243	6.87325865	-0.00014510	-0.01252303	-0.01467911
H	9.96786414	7.88157843	5.90862241	-0.00903328	-0.00533496	-0.00362948
H	6.08390269	-0.68053620	8.90035994	0.00271954	0.01209166	-0.02648704
O	5.94395194	-0.16029528	8.01239693	0.00094334	-0.00514985	-0.00584595
H	6.46626027	-0.71243426	7.36308940	-0.00116896	-0.00328616	-0.00156941
H	8.29509485	2.74344408	6.39674356	0.00524803	-0.00179849	-0.00424825
O	8.26786204	3.75751882	6.23981801	-0.01206192	0.00789278	-0.00482923
H	8.19273777	4.13508064	7.17907376	0.00482276	-0.00076328	-0.00464919
H	8.01954292	0.43182592	4.89213363	0.00737313	0.01701315	0.00151701
O	7.92689706	0.36756109	3.90000574	0.00435129	-0.01827733	-0.00959211
H	6.74470322	1.38676844	4.01725696	-0.00652108	0.04804701	-0.00969807
H	2.29347346	5.90518896	5.33229201	0.02243804	0.00184370	-0.00770420
O	2.78204268	6.15316504	4.43974025	0.00719178	0.00798981	-0.01221829
H	2.45136272	5.43805189	3.83561378	0.00446825	-0.00756328	-0.00944057
H	6.88092047	4.74508472	8.62877580	-0.00754605	0.00181923	-0.00617147
O	7.82883944	4.49922026	8.79291469	-0.01919893	0.00411228	0.00405032
H	8.22698924	5.39029957	9.18790233	-0.00625786	0.00363767	-0.00202058
H	4.98952443	7.99357186	10.32949724	-0.04311105	0.02809612	0.00806710
O	6.15074809	7.19595478	10.27999594	-0.02966315	-0.03095616	0.04462058
H	5.75506788	6.29835079	10.42076985	-0.00089067	-0.00508784	0.01587743
H	3.05233101	7.29398430	9.94445256	0.00179793	-0.00197062	0.00692529
O	4.03698399	8.50619407	10.16867600	-0.00475798	-0.00218591	0.00823079
H	3.73249833	8.76563967	11.07276261	0.00536926	-0.00614535	-0.01346153
H	2.82295649	8.73973828	6.50020701	0.00361663	-0.00302140	-0.00396533
O	2.26346500	9.47256515	6.07749798	0.00441224	-0.01505802	0.00328138
H	1.71618249	9.00920384	5.38504763	0.00267413	-0.00313953	0.00120173
O	9.43289973	2.49578340	3.74233755	0.00361525	0.00132805	-0.01390810
O	9.93822006	5.33600260	4.13365670	-0.01467902	0.00099381	-0.01890955
H	8.84271260	1.64186957	3.67160073	-0.01258915	0.00131858	-0.00716750
H	10.16192811	2.37122365	3.08956007	0.01044696	-0.01096930	0.00830597

H	10.16085440	5.09101424	5.08971544	-0.00464653	0.00104470	0.00220758
H	9.94992294	6.33848011	4.12677004	-0.01475561	0.00471862	-0.00428723
O	2.37921447	6.61560786	9.56351132	-0.00160693	-0.00898433	0.01287054
H	3.04993374	7.18565477	8.17978178	0.00697072	-0.00365007	-0.00500815
H	1.57291061	7.24087090	9.55965728	0.00491831	-0.00756390	0.01049218
O	4.36992221	4.86955750	10.16418050	0.00222894	0.00542137	0.02318963
H	3.43925540	5.24416482	10.08309553	0.00525589	0.00111780	0.00889578
H	4.31883265	3.85510223	10.04914520	0.00366881	0.00600865	0.01070198
H	4.66186286	6.14959299	7.52375823	0.00300646	-0.00857843	-0.01293465
H	4.89807285	5.20118807	8.81356928	0.00595364	0.00729081	-0.01237888
H	5.97244244	8.38337224	5.49836890	-0.01312177	0.00367345	0.02442773
H	7.20750303	6.34924303	5.68265534	0.03688506	0.00388057	0.02488057
H	6.81352341	5.93422339	7.12388354	-0.00879599	0.00805781	-0.02148677

*CHOH;*OH

121

Lattice="9.84 0.0 0.0 0.0 8.52169 0.0 0.0 0.0 11.75" Properties=species:S:1:pos:R:3:forces:R:3

C	1.27243997	0.58455100	0.72785536	-0.00005016	-0.00602025	-0.00472718
C	1.27852977	2.01345893	0.66972994	0.00079044	-0.00498408	-0.00175190
C	2.50375573	2.72731716	0.85528211	-0.00206440	-0.00244504	-0.00311688
C	2.50589502	4.14929040	0.95199995	0.00077291	0.00607951	0.00516283
C	1.28608394	4.85678830	0.77578912	-0.00824337	0.00613524	-0.00034984
C	1.28331146	6.27208955	0.86142292	0.00147866	0.00316220	0.00278107
C	2.51336293	6.97752642	1.03408137	0.00173565	-0.00071216	0.00689180
C	2.49510238	8.40016507	0.93105562	-0.00128634	-0.00594749	0.01006747
C	3.72545500	0.59282011	1.01403779	0.00031262	-0.00654123	0.01535003
C	3.72772148	2.01604015	1.01345810	0.00560039	0.00047874	-0.00801798
C	4.92927887	2.71077994	1.36294206	0.02424172	-0.01409806	-0.04333428
C	4.80174119	4.05974932	1.83291260	0.01223551	0.00588038	0.01224534
C	3.67763957	4.83985613	1.40699106	-0.00111019	0.03056477	-0.01062955
C	3.72308778	6.28025829	1.37658965	0.01026522	-0.00267685	0.00188669
C	4.96299323	7.02197669	1.48175908	-0.01220041	-0.00564411	-0.00246063
C	4.95172697	8.40492496	1.16724015	0.00969176	-0.01336918	0.01384005
C	6.18522570	0.60681422	1.07164815	-0.00179331	-0.00541484	-0.00679137
C	6.19418768	2.01454755	1.24033445	0.00380990	0.01073110	0.00221927
C	7.45738857	2.70377642	1.16247196	-0.00930714	0.00497775	-0.00071638
C	7.61542088	4.04115937	1.63411325	-0.00834485	0.00240820	0.01702978
C	6.23971584	6.46717717	1.88766070	-0.00363447	-0.00584685	0.00829012
C	7.42112127	7.01252879	1.26102790	0.00846000	-0.00865152	-0.04185933
C	7.40806342	8.39955821	0.91714141	-0.00251378	-0.00619968	-0.02397280
C	8.63004439	0.58028636	0.70179643	0.00196123	-0.00578793	-0.00214667
C	8.63009961	2.00910560	0.71587609	-0.01063745	0.00343579	-0.00663832
C	9.87971622	2.72075999	0.62586538	-0.00947068	0.00412830	-0.01610995
C	9.88846068	4.14456243	0.70983154	-0.00917838	0.00340693	-0.02031547
C	8.68761268	4.83132727	1.09835679	-0.00885879	0.00203407	-0.01177193

C	8.65332170	6.27413033	1.09011040	-0.00656619	0.00396445	-0.01140507
C	9.87651255	6.97945005	0.84987809	-0.00310805	-0.00615256	0.00800175
C	9.87378150	8.40034264	0.72342259	-0.00492793	-0.00186406	-0.00410852
Cu	7.99992813	5.30014916	5.51737538	-0.00108362	0.01510059	0.00671781
Cu	5.39357899	5.33170475	4.99003426	-0.03724196	0.01005662	0.01070022
Cu	7.05861055	6.78252302	3.83363416	0.00203093	0.01912682	0.09396023
Cu	4.34741304	3.64876431	3.70038106	0.02325342	0.05734365	0.08923378
Cu	6.28395736	4.77171217	2.79462902	-0.00831200	-0.01101819	0.03940625
Cu	8.61955353	4.35281540	3.35609532	-0.02427182	-0.05554763	0.05806947
C	6.53143373	6.58889838	5.92121111	-0.00148097	-0.00349646	-0.00312744
O	6.05087155	4.61990444	8.35078176	-0.00874199	0.00110911	0.00051507
O	6.21967523	7.98510077	6.09196955	-0.00797026	0.01909301	0.00768700
H	3.46346515	3.43197988	7.99770063	-0.00213736	0.00663737	-0.01155642
O	3.57320828	4.07700932	7.24843353	0.00409576	0.00989656	0.00882643
H	5.24560002	4.61654072	7.75776885	0.01165759	0.00443232	-0.00842267
H	0.42419167	8.16267662	3.07203635	-0.01026661	-0.00480355	0.01214773
O	0.72337019	7.70744842	3.89714842	-0.01115740	0.01227700	-0.01696462
H	1.75979814	7.66449835	3.88720298	-0.00501393	0.00698019	-0.00971951
H	2.39141243	2.32309189	5.29568732	0.00417730	0.00872933	-0.01052461
O	3.39046049	2.70510227	5.08617979	0.00098148	-0.00207611	-0.01384350
H	3.56007486	3.23332466	5.95851355	-0.00093374	0.00084029	0.01411915
H	7.82851581	2.83735375	9.41592071	-0.00463348	-0.00218692	0.00096519
O	7.17055384	2.13619450	9.78814350	-0.00353417	-0.00002531	0.01581560
H	7.48175684	1.99410997	10.72181932	-0.00198809	0.00172776	-0.00991007
H	3.30491618	-0.67058178	6.01910220	0.00648528	-0.00477270	-0.00376738
O	2.95784867	-0.16062895	6.81049016	0.01246964	0.00021849	-0.00582197
H	3.77324226	0.26202731	7.17815627	0.01167433	0.00530176	-0.00613447
H	1.01915403	6.30923811	6.47470859	-0.00471145	-0.00229103	-0.00601448
O	1.76284530	5.65559858	6.26198449	0.00400898	0.00739717	-0.00752214
H	2.74064746	4.64363779	7.10774427	0.01301836	0.00054382	-0.00591022
H	5.41810151	2.37495435	7.49872632	0.00755768	-0.00489029	-0.01827737
O	5.91048108	2.27525606	6.62658154	-0.00402552	-0.00070356	-0.00314737
H	5.84250962	3.14930076	6.14461457	0.01562921	-0.00852808	-0.00836622
H	8.65593432	9.19903283	6.47763700	-0.01079725	0.00305427	-0.00719113
O	8.26526408	10.00377804	6.02956169	-0.00301108	-0.00754484	0.00198750
H	7.35169662	10.22722635	6.44068511	0.00411125	0.00488797	-0.00500782
H	3.93039247	8.13763297	4.20001333	0.01707987	-0.00657268	-0.00990465
O	4.82504685	9.14060121	4.29209191	-0.00130287	0.00088622	-0.00987570
H	4.32831171	9.92234277	4.71762251	0.01408245	-0.02905519	0.00282025
H	5.47737625	2.44856747	9.45700515	-0.00295704	-0.00124925	0.00600611
O	4.55386927	2.34375631	9.05054448	0.00966089	-0.00655937	0.00719144
H	4.25408402	1.41922175	9.37069330	0.00868669	0.00085757	0.00185616
H	7.51610646	6.44143284	10.15520190	0.00046568	0.00410766	0.01164517
O	8.50191673	6.36059357	9.92923038	-0.00548730	0.00502550	0.00624841
H	8.90150203	6.29448570	10.84130427	-0.00400508	0.00183447	-0.00404412

H	-0.55553628	-0.67906328	9.39142909	-0.00390896	0.00476219	0.00133411
O	0.20427857	-0.03209510	9.23076322	-0.01198332	0.00814495	0.00928125
H	0.10238078	0.16405698	8.26651772	-0.00368310	0.00306781	0.00289978
H	8.78327385	7.03140324	6.27398320	-0.00241512	0.00481986	-0.01118657
O	9.62862201	7.59736966	6.40562955	0.00055333	-0.00085355	-0.00628447
H	9.89648144	7.75269302	5.43465841	-0.01775178	-0.01272421	-0.00868263
H	6.62822540	-0.69945855	8.98715155	0.00215086	0.00775283	-0.00418043
O	7.08947315	-0.04169633	8.41013409	-0.00690575	-0.01110975	0.00128589
H	7.15692689	0.82331575	8.96909171	-0.00269167	-0.01162481	-0.00181733
H	8.99566627	2.96609080	6.02711353	0.00115977	-0.00381977	-0.01453294
O	9.26742289	3.92878805	6.21354877	-0.00615685	0.00488785	0.00078651
H	9.14906095	3.98844154	7.23721858	0.00902805	-0.01238117	-0.00193904
H	7.78252224	0.76080773	4.70581054	0.00167176	0.00415434	-0.01336147
O	7.67317698	0.38519273	3.74826202	-0.00268169	-0.00996488	-0.01194657
H	6.83904240	0.81722942	3.41655682	0.00532175	0.03011979	0.00340665
H	2.36116544	6.15693474	5.63787538	0.00845415	-0.00802788	-0.00119199
O	3.19591213	7.29707405	4.31252424	-0.00535768	-0.00402815	-0.01751013
H	3.57766083	6.56448391	3.75356839	-0.00118862	-0.00450930	-0.01226010
H	7.55756576	4.26927830	8.33439535	-0.00572974	0.00279542	-0.01651473
O	8.51288807	4.05243091	8.65437541	-0.00661780	-0.00014636	0.00459086
H	8.68815995	4.89218876	9.21203985	-0.00408759	0.00521572	-0.00385023
H	5.19232414	7.37299373	10.18290381	0.00544846	-0.00014241	0.01129528
O	5.82064301	6.56562750	10.21213212	0.00307484	0.00423920	0.01907192
H	5.12700825	5.82297882	10.42220352	0.00570589	0.00544392	0.01437543
H	3.21603326	7.81924995	9.62006641	-0.00034522	0.00081222	0.01221598
O	3.99039329	8.45922322	9.92959461	0.00872249	-0.00431288	0.01016067
H	3.75438575	8.67961442	10.87397903	0.00008220	-0.00737723	-0.01015527
H	1.77215735	9.38578029	6.14353695	-0.00824981	-0.00283599	-0.00865767
O	1.29905428	10.22713984	5.84977986	-0.01026532	-0.00624627	-0.00346494
H	0.75730910	10.07369186	5.01278918	0.00192227	-0.00104463	-0.00363707
O	9.73769912	2.14267793	3.60047364	-0.00029528	-0.00322842	-0.01492379
O	10.52802870	4.98501463	3.80250189	0.01346710	-0.00511466	-0.02424267
H	8.97407013	1.50920659	3.40540604	-0.00546103	0.00765710	-0.00444381
H	10.23303281	2.22335372	2.74384152	0.00495295	-0.00326300	0.01579811
H	10.71381895	4.84309284	4.77848181	0.02003155	-0.00020941	-0.00992432
H	10.47927362	5.99491996	3.73392655	0.00200953	0.00749456	0.00130161
O	2.17890610	6.81600486	9.09549170	0.00441271	-0.01216510	0.00208736
H	2.41311226	6.95682709	8.13588656	-0.00111801	-0.00298846	0.00875629
H	1.34748686	7.44637675	9.20095354	0.00470668	-0.00648023	0.00607892
O	3.84253261	4.87585353	10.32345961	0.00322187	0.00340158	-0.00130418
H	3.12907738	5.35774230	9.81739432	0.00499475	-0.00202631	0.00709265
H	3.98993068	3.99116026	9.88852328	0.00108693	0.00927857	0.01077383
H	4.66054495	6.52176878	7.33991341	0.03504548	-0.05579569	-0.19168618
H	5.93703943	5.45752412	8.89230081	-0.00072848	-0.01169551	-0.00899919
H	6.74144014	6.14974372	6.93968008	0.02096653	0.01158069	0.01518427

H	5.41197874	8.76578689	5.05569141	-0.00217310	-0.00202928	-0.00886238
H	6.53078061	8.27879275	7.03696580	-0.00577453	-0.01104200	-0.01662104
H	4.50015965	6.72810864	8.07852959	-0.03674340	0.05038286	0.17999655

CH_3OH

121

Lattice="9.84 0.0 0.0 0.0 8.52169 0.0 0.0 0.0 11.75" Properties=species:S:1:pos:R:3:forces:R:3

C	1.27332642	0.75324739	0.69242801	-0.00167066	-0.00554135	-0.00343142
C	1.27339027	2.17998593	0.60449704	0.00264788	-0.00504026	-0.00752556
C	2.50171012	2.88965181	0.77959679	0.00669378	0.00160869	0.00717413
C	2.50450790	4.31270911	0.91396002	0.01311594	0.00632236	0.02233848
C	1.27991818	5.02553659	0.78541030	-0.00625490	0.00857032	0.00999144
C	1.27924503	6.44176091	0.89490263	0.00074277	-0.00084125	0.00411352
C	2.50929208	7.14546838	1.04447202	0.00244014	-0.00377920	0.00043002
C	2.49232534	8.56583244	0.90823757	-0.00162446	-0.00468763	0.00953226
C	3.72679893	0.75253021	0.94249239	0.00024288	-0.00211967	0.01040646
C	3.72829470	2.17371443	0.89450769	-0.00003919	0.00700352	-0.01604159
C	4.93033967	2.87238862	1.21627692	0.01017675	0.00311166	-0.05111761
C	4.81319121	4.20060529	1.70951849	0.01140134	0.00684961	0.00337402
C	3.69951227	4.99224133	1.32437837	0.00344789	0.01587118	-0.01728579
C	3.72653288	6.44389047	1.35831708	0.01350849	-0.00533227	-0.01031481
C	4.97410081	7.19269423	1.44783255	-0.01418832	-0.00329948	-0.00206691
C	4.95772812	8.57575750	1.10847534	0.00798668	-0.01378787	0.01278473
C	6.18769632	0.77593861	0.99269945	-0.00169498	-0.00315238	0.00146452
C	6.19575909	2.18501270	1.08739513	0.01182778	0.00777598	-0.01046475
C	7.44490261	2.88242532	0.99392108	-0.01393152	0.00910876	-0.00960619
C	7.58830506	4.21484097	1.46855056	-0.00960556	0.00592396	0.00829650
C	6.24109206	6.64255238	1.84300012	-0.00035634	-0.00667536	0.00707384
C	7.43173059	7.19996627	1.26086920	0.01271856	-0.00713441	-0.01391349
C	7.41730584	8.57785951	0.89124660	-0.00365614	-0.01111870	-0.00132123
C	8.63530895	0.75622463	0.64685676	0.00174753	-0.00690379	-0.00184087
C	8.63141929	2.18445834	0.60911400	-0.00768966	0.00432531	-0.00533919
C	9.87908573	2.89455761	0.53967707	-0.00583529	0.00421927	-0.02549092
C	9.89048937	4.31844605	0.65408981	-0.00547970	0.00680743	-0.02536318
C	8.68466812	5.00936356	1.03374881	-0.01062688	0.00642305	-0.00882224
C	8.65807375	6.45444122	1.08702024	-0.00873617	0.00421873	-0.00746678
C	9.87553762	7.15467237	0.86114114	-0.00488802	-0.00974563	0.00626443
C	9.87627450	8.57275608	0.70242875	-0.00440300	-0.00068820	-0.00618696
Cu	6.88786670	4.22729862	4.86493824	0.02294503	-0.03679426	-0.00418868
Cu	4.84493367	5.49478979	4.54619247	0.02885306	-0.00467230	0.01568550
Cu	6.83709361	6.80969130	3.93537011	-0.01299159	0.03486445	0.05464787
Cu	4.86649281	3.24239621	3.63172151	0.01502548	0.03325291	0.02493197
Cu	6.28208422	4.92113034	2.66933757	0.00538618	-0.00279368	0.05840024
Cu	8.60612750	4.43172981	3.21791533	-0.03157257	-0.02287270	0.03880974
C	6.55299609	6.08700291	6.94069530	0.00806791	-0.00192806	0.01804528

O	5.22149340	4.61972282	9.19596613	0.00742878	0.00690138	-0.00365117
O	7.10924486	7.10293038	6.08153925	0.01441417	0.03090816	-0.01524367
H	3.27337074	3.52605882	7.66753546	-0.00350780	0.00569338	-0.01633001
O	3.49516877	4.27264922	7.04071057	0.00983207	0.00158140	0.00024870
H	4.25832948	4.64568179	9.62824775	0.00582884	0.00818682	0.00604282
H	0.45703358	8.10981047	3.19411658	-0.00953898	-0.00448804	0.00321098
O	0.84956656	7.98398028	4.09590127	-0.00378100	0.00905030	-0.02199745
H	1.68193063	7.40437309	3.99355577	0.00695549	0.00009216	-0.00665927
H	2.40634878	2.49342821	4.99580732	0.01385570	0.01625470	-0.01527224
O	2.94814837	3.27070955	4.63743088	-0.00218110	-0.00948302	-0.02214204
H	3.19990202	3.69292724	5.53110736	-0.01341441	-0.00713606	0.00997987
H	7.85859889	2.49026578	9.36034004	-0.00366491	-0.00750793	0.00039678
O	7.22801259	1.73840746	9.55699728	-0.00607795	-0.00578836	0.00900395
H	7.56164262	1.37733129	10.41624491	-0.00662628	-0.00715968	-0.01115305
H	4.28165380	-0.78613631	5.79978912	0.00805357	0.00521168	-0.00186311
O	3.81354040	-0.45533637	6.61472764	0.00349779	-0.00158543	-0.00213955
H	4.59371651	-0.21095666	7.25276930	-0.00008673	-0.00603351	-0.00624229
H	0.48789071	6.94425192	6.84503167	-0.00850684	-0.00126413	-0.00464178
O	1.83975161	6.29320404	7.22983212	-0.00058359	0.00048131	-0.01504817
H	2.75212989	4.96788082	7.17436732	0.01098389	0.00015658	-0.01097220
H	5.06384448	2.12323175	7.33377349	0.00469120	0.01621597	-0.01999966
O	5.66301451	2.14441839	6.48353749	0.00066997	-0.00016244	0.01205045
H	5.54593225	3.04682706	6.02628058	-0.00243742	0.00470074	0.02474265
H	8.57738779	9.18894672	6.79175870	-0.01122187	0.00889233	-0.00089866
O	8.20868359	9.94828978	6.26848701	-0.00211865	-0.00978312	0.00457005
H	7.25875141	10.17326495	6.56829462	-0.00225520	0.00666705	-0.01222960
H	4.08038604	9.26028566	4.07029794	0.00054296	-0.02185894	-0.00693802
O	5.01802106	9.53081055	4.20453835	-0.00861493	-0.00038307	-0.00858352
H	5.10136037	9.82921092	5.18259458	0.01307371	-0.02076469	0.00922546
H	4.80883942	2.73409520	9.16656125	0.00953895	-0.01579048	0.01061503
O	4.19668364	2.15000622	8.63734686	0.00013789	-0.00761076	0.00798204
H	4.22633439	1.24788464	9.10868844	-0.00628030	0.00794429	0.00535048
H	7.78497646	6.84041060	9.93807664	0.00430347	0.01330064	0.00677300
O	8.75065976	6.76735917	9.59140447	0.00287236	0.00843301	0.00354901
H	9.28299814	6.75829366	10.42988528	0.00135380	-0.00133728	-0.00062911
H	-0.41167789	-0.08450973	8.96940472	-0.00779856	0.00206474	-0.00411046
O	0.32271921	0.59528239	9.03498458	-0.00955502	0.00226844	0.00639330
H	0.58866123	0.78453582	8.09643307	-0.00279224	-0.00567804	0.00137296
H	8.13934944	7.12747814	6.26642857	-0.00043577	-0.01330566	-0.00635762
O	9.54137498	7.53046230	6.49462405	0.00018424	-0.00630265	0.00266862
H	9.86697530	7.79003126	5.58001562	-0.01158044	-0.00593639	-0.00170326
H	6.47394040	0.69374165	8.56624730	0.00072481	-0.01250943	-0.00310790
O	5.99937679	-0.01520758	8.01540755	0.01373416	0.00050454	-0.00018610
H	6.59292737	-0.24724037	7.24694517	-0.00569410	0.00335745	-0.00208634
H	8.95510598	2.97551480	6.20872126	-0.00094053	0.00214555	-0.01242781

O	8.93516787	3.98079869	6.27979845	0.00032110	0.00229707	0.00680969
H	8.71420676	4.13450310	7.26462226	-0.00780835	0.00396084	-0.02442304
H	7.88964179	0.80562206	4.81249415	-0.01061983	0.00193847	-0.01402438
O	7.81164488	0.45915002	3.85350926	-0.00137489	-0.00798484	-0.01666735
H	6.95402722	0.86005111	3.54023853	0.00106350	0.03803169	-0.00184239
H	2.55545620	6.91996522	6.88316560	0.00311356	-0.00570685	-0.00651041
O	2.86484371	6.22855280	4.12646013	0.01005714	0.00119266	-0.00162909
H	2.19838177	5.46558233	4.21718561	-0.00544726	-0.00267053	-0.01269069
H	7.21576851	4.33163295	8.96308117	0.00161050	-0.00848724	0.00114034
O	8.19966669	4.22516042	8.84997063	-0.01462991	0.00600869	0.00600038
H	8.56252147	5.11104665	9.19973926	-0.00096940	-0.00013069	0.00403723
H	5.04098601	7.76465635	10.02905642	-0.00141220	-0.00075221	0.00927160
O	6.22480293	6.69339702	10.35203575	-0.00102823	0.00762979	0.01864973
H	6.20849633	6.48264243	11.32984111	-0.00095518	-0.01325505	0.00365223
H	2.87890100	7.57285373	9.73659773	-0.00010613	-0.00112118	0.00558145
O	4.20345329	8.35789533	9.95791845	0.00267539	-0.00705736	0.01223656
H	4.07225709	8.67135394	10.89691268	0.00905738	-0.00565133	-0.00793172
H	2.51565409	9.25845819	6.29324882	0.00213245	-0.00001047	0.00207665
O	1.73089023	9.85295851	6.08550244	0.00063350	-0.00810492	-0.00414097
H	1.27402938	9.38065914	5.33517182	-0.00305735	0.00216589	-0.00008785
O	9.73571479	2.47758212	3.59154227	-0.00071833	-0.00308791	-0.01123540
O	10.21564553	5.17097408	4.34885848	-0.00090367	0.00548330	-0.01955714
H	9.03660898	1.76159147	3.47921929	-0.00441897	0.00179262	-0.00549259
H	10.29749450	2.39357846	2.77953859	0.00691560	-0.00693594	0.01341540
H	9.87770503	4.73926314	5.22386662	0.00546603	0.01309550	0.01083511
H	9.98257126	6.13263894	4.43527231	-0.01406952	0.00367223	0.00535040
O	1.95084008	7.14402325	9.61388329	0.00042790	-0.00079612	0.02486884
H	1.86820324	6.58553998	8.24697883	0.00141270	0.00212293	-0.00343247
H	1.33220710	7.95936836	9.49285885	0.00690344	-0.00426172	0.01195082
O	2.76201109	4.61405519	9.80335857	-0.00358203	0.00483349	0.01587951
H	2.36148591	5.51980392	9.98389170	0.00128360	0.00081220	0.01140289
H	2.58352220	4.09200927	10.62764413	0.00708119	0.00153613	-0.00777574
H	6.77822326	6.34121465	7.99289702	-0.00006389	-0.01167919	-0.00952010
H	5.83268841	5.85881428	9.88514010	-0.00514760	0.00638244	0.01543450
H	6.95225531	5.07092974	6.69547246	-0.02009700	-0.00038656	-0.01079776
H	3.11527576	6.21844754	3.13994707	-0.00649099	-0.00456268	-0.00071773
H	4.90547470	4.70911377	8.24522249	0.00343177	0.00398464	-0.00947805
H	5.44924036	6.06943796	6.79091593	0.00259966	-0.01079004	0.01182261

*CH;*OH

119

Lattice="9.84 0.0 0.0 0.0 8.52169 0.0 0.0 0.0 11.75" Properties=species:S:1:pos:R:3:forces:R:3

C	1.26926632	0.74572520	0.79268172	-0.00319498	-0.00384523	-0.00183725
C	1.27876467	2.17076192	0.74924779	0.00121245	-0.00292814	-0.00120960
C	2.51085748	2.88382991	0.85914837	-0.00019365	-0.00588051	-0.00837306

C	2.50724040	4.30537856	0.91256013	0.00159856	0.00257677	-0.00375217
C	1.27763146	5.01734244	0.82691534	0.00014790	0.00189044	0.00544337
C	1.27646011	6.43324364	0.91347235	0.00141785	0.00291912	0.00739193
C	2.50936082	7.14369414	1.03293940	0.00009097	-0.00231578	0.01459525
C	2.49967227	8.56682558	0.95479153	-0.00163385	-0.00124657	0.01195846
C	3.73250024	0.76097761	1.03412619	0.00312262	0.00029541	0.01220263
C	3.74236482	2.18184138	1.02006917	0.01000374	0.00004669	-0.00889643
C	4.93901531	2.87633848	1.38696677	0.02556599	-0.01093126	-0.02208151
C	4.80092417	4.23404049	1.82004684	0.01021439	0.00435807	0.01736901
C	3.69384329	5.00103914	1.32752242	0.00156368	0.02938110	-0.02814214
C	3.72448823	6.44393858	1.31040636	0.01337520	0.00314896	0.00999260
C	4.95428899	7.17569773	1.43154297	-0.01391031	-0.00954000	-0.00726038
C	4.95711742	8.57401010	1.17585977	0.00309231	-0.00482222	-0.00311112
C	6.19436562	0.77496165	1.15752914	-0.00181003	0.00916528	-0.01190673
C	6.20503633	2.17900998	1.35672457	0.00054920	0.01272961	0.00794808
C	7.47972208	2.86603767	1.37014200	-0.01913461	-0.00594107	-0.00331664
C	7.66210437	4.19386917	1.90280701	-0.00720081	0.00382000	0.02086931
C	6.20745153	6.58701358	1.86452761	0.00161235	-0.01384877	0.01193764
C	7.42520563	7.15859803	1.32141113	0.02009689	-0.01474412	-0.05766217
C	7.41603436	8.55818775	1.02710166	0.00113591	0.00121659	-0.01957835
C	8.63355987	0.74353077	0.82469855	-0.00244799	-0.00572445	-0.00394072
C	8.63940130	2.17242913	0.89038462	-0.01090646	0.00063929	-0.00387025
C	9.88475683	2.87803808	0.79203411	-0.00438163	0.00056820	-0.00493841
C	9.88369102	4.30098836	0.87470075	-0.01007197	0.00193119	-0.00054356
C	8.70354812	4.98522250	1.29935634	-0.00747860	0.01599522	-0.00847576
C	8.65696368	6.42112811	1.21135070	-0.00913428	-0.00816950	0.00784513
C	9.87098625	7.13601402	0.93497919	0.00012499	0.00578364	0.01351744
C	9.87269869	8.55781321	0.81010534	-0.00256336	0.00169592	0.00143675
Cu	6.09978500	3.58381662	4.90466177	0.02331847	-0.02694393	-0.01720376
Cu	5.12516443	5.85870665	5.01767915	-0.05434799	0.03171307	-0.03451634
Cu	6.66191137	7.27425455	3.68622111	-0.00395870	0.01736212	0.11238181
Cu	4.08539990	3.99777737	3.66934543	0.03945364	0.00508437	0.10971037
Cu	6.20448063	4.99478410	2.90295909	0.00410382	-0.00042749	0.05375306
Cu	8.20738956	3.94458813	3.84423720	0.00900950	-0.05039888	0.02860959
C	6.47610851	5.95323150	6.44801256	-0.00432483	0.00961113	0.01125339
O	4.00649911	5.92959278	8.19174277	0.00864635	0.01858861	-0.00688161
H	3.76475104	3.04444376	8.45961751	0.00074702	-0.00240840	-0.01280479
O	3.36080852	3.50776618	7.65116756	0.00440108	-0.00740421	-0.00908778
H	3.69326723	4.48114347	7.78988938	0.01416415	0.00052771	-0.01870741
H	0.59550991	8.41114971	3.74939839	-0.00473718	-0.00796637	-0.00582228
O	0.92928861	7.52380629	4.13499930	-0.00251590	0.00446558	-0.01583831
H	2.39604373	6.94092484	4.47295226	0.02328225	-0.01271795	-0.00698270
H	2.08182190	2.77031513	4.93302584	-0.00221055	0.00293510	-0.00133867
O	2.32021444	3.30752756	4.07877307	0.01024824	0.00234452	-0.01852391
H	1.57933243	3.99324921	4.00458304	-0.02633945	-0.00090738	-0.00697812

H	7.82078456	3.22259678	9.31270327	-0.00416519	-0.00856794	0.00266422
O	7.47374233	2.30099243	9.57687644	-0.00287125	-0.01713656	0.00517633
H	7.77702277	2.19795755	10.51556360	-0.00340309	-0.00389208	0.00015460
H	3.36560038	-0.73375461	6.04856211	0.00836366	-0.00286998	0.00341891
O	3.37740736	0.06634053	6.67773880	0.00787599	0.00157829	-0.00360407
H	4.32786010	0.12703531	7.04570716	0.00011171	-0.00761520	-0.00803190
H	1.58217185	5.78245389	7.50456605	-0.00130823	-0.00466302	-0.01128333
O	1.51285340	5.17548518	6.71270246	-0.00695471	0.00824453	-0.00643023
H	1.93105561	4.31271118	7.03422816	0.00098656	0.00151805	-0.01764845
H	5.56028438	1.85469817	6.99780012	0.00554278	0.00911293	-0.00331118
O	5.19604296	2.47650795	6.29303923	0.01488453	0.00172541	0.00298199
H	4.37944213	2.94766477	6.77013034	-0.00666136	0.00375421	-0.00540236
H	8.74871089	8.85329063	6.63856110	-0.00427039	-0.00021430	-0.00887372
O	7.97040596	9.49491335	6.50202192	0.00224511	-0.00690766	0.00504012
H	6.78641567	9.03233401	7.24249577	-0.00518106	0.00028485	-0.00517623
H	4.27776536	8.66147078	4.13160214	0.00400172	-0.00481442	-0.00569010
O	4.59398806	9.59511156	4.07291777	-0.01878547	-0.01406393	-0.01398304
H	4.60455835	9.92576314	5.02325086	-0.00693674	-0.00944393	-0.01875463
H	5.89091687	2.34964167	9.55573860	-0.00849998	-0.00764947	-0.00113656
O	4.87103465	2.52269401	9.63129178	0.00103311	-0.01344569	0.01061922
H	4.44870096	1.63141567	9.82616754	0.00710274	-0.00040746	0.00441675
H	7.74418333	7.32934662	9.96591794	-0.00270156	0.00903796	0.00510560
O	8.72658667	7.11505403	9.78232746	0.00022250	0.00706427	0.00353296
H	9.09254753	6.94460412	10.69106118	-0.00177656	0.00058289	-0.00320264
H	-0.44028025	0.20093645	9.55339671	-0.00326573	0.00529633	0.00245641
O	0.26261510	0.91368145	9.40625227	-0.00330229	0.00515087	0.01473111
H	-0.13660208	1.49801866	8.71764480	-0.00570834	-0.00192249	-0.00664417
H	10.40211044	7.47154932	7.34629303	-0.00337280	-0.00280880	-0.00732074
O	9.77344040	7.62670932	6.55711503	0.00270843	-0.00266993	-0.00367833
H	10.41885238	7.58722345	5.10230472	-0.00675213	0.00281303	-0.00456695
H	6.00083809	-0.15152592	8.52106467	0.00044612	0.00398425	-0.00187181
O	5.83519521	0.34188414	7.66749117	-0.00603068	-0.00256929	0.00674752
H	6.36538395	-1.68459851	7.11987824	0.00451783	-0.00071369	0.00269788
H	8.35131187	2.35920326	6.19447386	-0.00974858	0.01607703	-0.01047470
O	8.54139526	3.31653809	5.80379164	0.00475881	0.00046474	-0.00788231
H	8.60025704	3.96318354	6.55483555	-0.00319303	-0.01603922	-0.01619544
H	7.61995493	0.72686876	5.46124519	-0.00824217	0.00248114	-0.00952401
O	7.29602348	0.52562400	4.18546183	-0.00526926	-0.01407267	-0.01940087
H	6.43228567	1.01556576	4.01885471	0.00519938	0.03234811	-0.00692514
H	2.55912119	5.91996660	5.71205564	-0.00089640	-0.00488656	-0.00843818
O	3.11269427	6.51885486	5.07927594	0.00588169	0.00162970	-0.00353110
H	7.51845641	4.99380534	8.12694685	-0.01660351	0.00083411	-0.00428962
O	8.31322949	4.64770129	8.61703703	-0.00816988	0.01186291	-0.00378982
H	8.61815020	5.48405037	9.10142395	-0.00117234	0.00066352	-0.00781922
H	5.27172640	7.85837358	10.18383682	0.00799923	-0.00127448	0.00745974

O	6.15351371	7.38766094	9.98558979	-0.00073469	0.00444035	0.01177774
H	5.92989833	6.42418077	10.17961220	0.00404611	0.00538405	0.00604027
H	3.10912163	8.00342131	9.44030358	0.00687682	-0.00228064	0.00520549
O	3.78435270	8.53683530	9.98232014	0.00045604	-0.00366370	0.00546004
H	3.34589164	8.64645673	10.86835203	-0.00154716	-0.00258466	-0.01255575
H	2.12965631	9.75836682	6.23449293	-0.00017470	0.00128008	-0.00680095
O	1.42995186	10.46204909	6.07528833	-0.00628300	-0.00901378	0.00537495
H	0.77392453	10.08642252	5.42300496	-0.00436923	0.00417172	0.00221700
O	9.68888821	1.35627333	3.77310715	0.00246927	0.00403794	-0.01843588
O	10.12526688	4.99725763	4.22468355	-0.00834129	-0.00010771	-0.01442912
H	8.68055342	1.09411621	3.74025349	-0.01313701	0.00720778	-0.00978957
H	9.91403895	1.84166063	2.94304823	-0.00690929	0.01127846	0.02439321
H	10.27131358	5.97009042	3.94679609	-0.01839141	-0.01114162	-0.00938217
O	1.75715672	7.57349004	8.44270898	0.00306436	-0.00839116	0.00260361
H	2.41223049	7.98322655	7.75608639	0.00248820	-0.00267161	-0.00179726
H	1.21836461	8.39025366	8.80189471	0.00367616	-0.00471100	-0.00165303
O	5.04778730	5.00471988	10.41709317	0.00954772	-0.00195530	0.01968713
H	4.53226325	5.15337947	11.25751179	0.00879520	-0.00053826	0.00130636
H	4.98284816	3.99305637	10.25712411	0.00659386	0.00284330	0.01249371
H	6.34369249	5.00996449	7.05701786	0.00465784	-0.01259658	0.03206447
H	4.40631268	5.66110382	9.09362581	-0.00326218	0.01021059	-0.01349995
H	4.76590549	6.23632420	7.63824182	-0.00645747	0.00538143	0.00791187
H	9.07021974	6.89302876	6.49622389	-0.00358753	-0.00427969	-0.00434314
O	1.36261510	2.12368145	9.80379160	-0.00645747	0.00538143	0.00791187
H	1.36261510	2.88692045	9.20748260	-0.01258747	0.01158143	0.00290617
H	1.36261510	1.36044245	9.20748260	-0.00525747	-0.00538143	0.00791187

*CH_3;*OH

121

Lattice="9.84 0.0 0.0 0.0 8.52169 0.0 0.0 0.0 11.75" Properties=species:S:1:pos:R:3:forces:R:3

C	1.25752162	0.54037501	0.73518019	0.00000017	-0.00479090	-0.00512622
C	1.26173333	1.96585280	0.66346164	0.00216711	-0.00456511	-0.00169386
C	2.48820186	2.68047952	0.81352935	0.00003281	-0.00373328	-0.00252548
C	2.48326830	4.10184427	0.90670994	0.00136424	0.00509286	-0.00203557
C	1.26244226	4.81214853	0.78368282	-0.00149177	0.00563113	-0.00050172
C	1.26606159	6.22621393	0.87896291	0.00130894	0.00270049	-0.00081810
C	2.50038051	6.93239774	1.02691465	0.00145013	0.00093215	0.00478370
C	2.48128093	8.35382252	0.92334241	-0.00032087	-0.00504048	0.00771418
C	3.71046356	0.54417489	0.99182325	0.00119387	-0.00417533	0.01322763
C	3.71313945	1.96634787	0.96135863	0.00932594	0.00448657	-0.00670232
C	4.91109008	2.66371758	1.29404669	0.01977375	-0.00887797	-0.02451674
C	4.78143130	4.01191630	1.72595902	0.01509401	0.00639662	0.01314026
C	3.66123608	4.79340907	1.33725336	-0.00006693	0.03056825	-0.03083030
C	3.71030931	6.23646506	1.34474095	0.00808837	0.00427509	-0.00186984
C	4.94667273	6.97756365	1.48062938	-0.01217079	-0.00502717	-0.00055493

C	4.93757454	8.36064599	1.16270747	0.00941418	-0.01223394	0.01206534
C	6.17232712	0.56106624	1.08746657	-0.00176129	-0.00561260	-0.00379629
C	6.17798679	1.96718566	1.23459697	0.00153584	0.01315191	0.00790856
C	7.44750938	2.65949549	1.17166954	-0.01238229	-0.00412466	0.00054129
C	7.59908032	3.99727778	1.62757896	-0.00991783	0.00090379	0.01267626
C	6.21894123	6.44296807	1.92978637	-0.00416713	-0.00983385	0.01676945
C	7.41316019	6.97863768	1.32093064	0.00540178	-0.00362069	-0.01994073
C	7.40033109	8.35708510	0.96418697	-0.00314589	-0.00759960	-0.01682624
C	8.62042262	0.53643096	0.73801433	-0.00248320	-0.00187081	-0.00820312
C	8.62365408	1.96033919	0.74693262	-0.01087519	0.00467735	-0.00690109
C	9.86291391	2.67634910	0.65065486	-0.00784642	0.00069602	-0.01038708
C	9.86901882	4.10097089	0.74686499	-0.01087690	0.00582883	-0.01661223
C	8.67672103	4.78740978	1.14482966	-0.00910320	0.01473807	-0.04749160
C	8.64434565	6.23279146	1.16348768	-0.00386888	0.00335146	-0.00706911
C	9.86228901	6.93573627	0.90299358	0.00034843	0.00210373	0.00588692
C	9.86024745	8.35595351	0.76181674	-0.00553582	-0.00227533	-0.00447247
Cu	8.07105724	5.47786557	5.78560033	-0.00659748	0.02603131	-0.02129124
Cu	6.23226113	4.18588189	4.97510425	-0.01773018	-0.02259536	0.01476682
Cu	6.91401691	6.59063457	3.93531424	0.00206921	0.06306171	0.06710549
Cu	4.24218139	3.86919854	3.66438825	0.02494398	0.00622033	0.09370468
Cu	6.22054514	4.71238495	2.69968188	-0.00618538	-0.00332995	0.06828466
Cu	8.36616602	4.20706195	3.59562453	-0.02841022	-0.06665315	0.07751471
C	6.88614387	7.05507421	6.02552719	-0.00579396	0.00911000	0.00399915
O	6.05482890	4.64838802	7.97785261	-0.01346830	-0.00973784	0.01631991
H	3.47047982	3.39752775	7.96572142	-0.00110656	0.00619540	-0.01033387
O	3.58163398	4.00562080	7.18316606	0.00645500	0.00445785	0.00109971
H	5.13636300	4.44698803	7.61457678	0.01668746	0.00231756	-0.01084059
H	0.67176970	7.97341592	3.06998784	-0.00884287	0.00079477	0.01567319
O	0.94169907	7.55410569	3.92528682	-0.00582512	0.01029997	-0.01593848
H	1.95011600	7.43082336	3.89202485	-0.00144226	0.00516758	-0.01182890
H	2.14217136	2.46513425	5.18076636	0.00607049	0.01149406	-0.01021674
O	3.12658140	2.86751068	4.89201620	-0.00071968	0.00515384	-0.01515856
H	3.42222108	3.24843025	5.79445865	0.00133488	-0.00292858	0.01212362
H	7.58053902	3.00051484	9.65433914	-0.00478812	0.00253479	0.00473368
O	6.86092539	2.35834387	10.01751355	-0.00722045	0.00391172	0.01795517
H	6.90420669	2.45303598	11.00716966	-0.00713616	0.00925624	-0.00300725
H	3.24440714	-0.51690979	6.16259064	-0.00118976	0.00744194	0.00023554
O	2.81069229	0.08750297	6.82562449	0.01100921	-0.00405629	-0.00494502
H	3.54273070	0.71433313	7.05148594	-0.00212049	-0.00596676	-0.00189455
H	1.16276558	6.39566189	6.49706717	-0.00590380	-0.00378195	-0.00710545
O	1.93597176	5.75203099	6.33958591	0.00275035	0.00431328	-0.00856415
H	2.78963663	4.64138616	7.06284665	0.00685109	-0.00716797	-0.00695709
H	5.21647835	1.99839676	7.58055680	0.01077446	0.00254509	-0.01366915
O	5.59135416	1.77730056	6.64611644	0.00403041	-0.01114286	0.00477755
H	5.54842476	2.65488219	6.11215784	0.00375735	0.00424730	-0.00823776

H	8.65571649	9.56938376	6.71280654	-0.00590692	0.01241773	-0.00764987
O	8.14020911	10.18023537	6.12649081	0.00270868	-0.00425336	-0.00033674
H	7.20651078	10.19431510	6.54591361	0.00075746	0.01911746	-0.00932532
H	4.06012606	7.98739374	4.33209964	0.01128855	-0.01071894	-0.01355681
O	4.73498913	9.29486635	4.30089190	0.00489217	-0.00491543	-0.01109002
H	4.03718362	10.03417637	4.37052597	0.01018330	-0.02495886	-0.00369641
H	5.42154649	2.46369357	9.44654940	-0.00389055	0.00165017	0.00056299
O	4.48962736	2.32274590	9.00580037	0.00682186	-0.00185525	0.00740633
H	4.20472242	1.39698838	9.35822491	0.00665198	0.00233119	0.00523309
H	7.45657084	6.49018098	10.08880326	0.00381286	0.00032539	0.01113431
O	8.46675865	6.36100938	9.97224051	-0.00289650	0.00469342	0.00800007
H	8.78206541	6.26722649	10.91219657	-0.00393651	0.00108138	-0.00613654
H	-0.50094397	-0.72433609	9.45635797	0.00320609	0.00054549	0.00311015
O	0.32298190	-0.18262154	9.23440364	-0.00927809	0.00685591	0.00827913
H	0.23142746	-0.07424416	8.25116507	-0.00302676	-0.00105931	-0.00060535
H	8.97280858	7.20537728	6.43247446	0.00074941	0.00335932	-0.00718445
O	9.89442923	7.62710609	6.48048340	-0.00084254	-0.00036624	-0.00876588
H	10.09564324	7.78436721	5.50160490	-0.02029257	-0.01041900	-0.00570369
H	6.70697968	-0.50238117	8.75774536	0.00185783	0.01029539	-0.00890570
O	7.36520410	0.20239631	8.55852396	-0.01720398	-0.00674989	0.00602752
H	7.21794019	0.90129603	9.26825787	-0.00145567	-0.01241453	0.00041355
H	8.96188040	3.18488139	6.19342353	-0.00421281	-0.00854746	-0.01499121
O	9.33105362	4.09399595	6.45513727	0.00314694	0.00655325	0.00224342
H	9.16971117	4.07213873	7.47998735	0.00709731	-0.01145879	-0.00423140
H	7.74868831	0.81230864	4.76693614	-0.00515210	0.00119646	-0.02067121
O	7.66216495	0.41765995	3.82729567	-0.00436676	-0.00740120	-0.01101868
H	6.82115204	0.81935162	3.47807047	0.00543462	0.01861571	0.00746757
H	2.53369927	6.21051830	5.68002226	-0.00069872	-0.00876579	-0.00408163
O	3.51353334	7.09107001	4.49408723	0.00420950	-0.00834815	-0.01009363
H	4.10611699	6.35435971	4.14951114	0.02198613	0.00732717	-0.00197048
H	7.51915671	4.22809916	8.37351181	-0.00901161	0.00097436	-0.01926143
O	8.41685613	4.04393601	8.83220002	-0.00628666	-0.00576196	0.01043348
H	8.55231343	4.93491550	9.34404903	-0.00456796	0.00568381	-0.00265599
H	5.24664125	7.34537081	10.01745043	0.00274235	-0.00139486	0.01198495
O	5.85889109	6.53019124	9.97633069	-0.00142849	-0.00116373	0.01632248
H	5.19438086	5.77515822	10.22800215	0.00677710	0.00188458	0.01153799
H	3.25462096	7.79166539	9.61019215	-0.00037228	0.00158501	0.01108822
O	4.02825393	8.45690140	9.89842664	0.00849193	-0.00152141	0.01066814
H	3.85764473	8.62807079	10.86475661	0.00242866	-0.00656029	-0.01003631
H	1.60457365	9.49931097	6.04666183	-0.00434205	0.00326990	-0.01020183
O	1.11301326	10.32618558	5.74242247	-0.00739170	-0.00671211	-0.00084948
H	0.57242973	10.18288358	4.89148308	-0.00242693	0.00234424	-0.00113546
O	9.58713452	2.12984256	3.58769558	-0.00017793	0.00098459	-0.01568076
O	10.37130178	4.81496747	3.90120588	0.01261870	-0.00351183	-0.02219772
H	8.84320103	1.44526879	3.42998731	-0.00464578	0.00239649	-0.00217976

H	10.07047833	2.23590388	2.72890857	0.00376422	-0.00211205	0.01477873
H	10.63087877	4.65288532	4.85062053	0.01337571	0.00012041	-0.01284728
H	10.44890926	5.81616867	3.81717187	-0.00385924	-0.00118150	0.00220936
O	2.26325643	6.74368364	9.23300814	0.00498533	-0.01114184	0.00077854
H	2.41892723	6.68096186	8.25523719	-0.00134432	0.00372332	0.01101139
H	1.38965478	7.36368488	9.28712441	0.00461697	-0.00613986	0.00590964
O	3.95351562	4.83518754	10.24850484	0.00506775	-0.00051200	0.00250995
H	3.17166721	5.34235843	9.87898431	0.00463957	-0.00307140	0.00856714
H	3.96308587	3.91802059	9.86286603	0.00291584	0.00704601	0.01085723
H	4.29500962	6.41402517	7.33034420	-0.03180807	-0.11215221	-0.08932370
H	5.90795900	5.44089818	8.57962053	0.00511688	-0.01371668	-0.00956249
H	7.30077573	7.97772200	6.47726322	-0.01172038	-0.01188709	-0.01611409
H	5.15836888	9.42755702	5.20508168	0.00541082	0.00763889	-0.01026707
H	4.50892180	7.01691053	7.76670886	0.04617615	0.11056644	0.07576138
H	6.03054160	7.42855190	5.38483693	0.00327198	-0.01267151	0.00017430
H	6.34986515	6.49942807	6.82205848	-0.00739907	0.00951442	-0.01628628

*CH_3O;*OH

120

Lattice="9.84 0.0 0.0 0.0 8.52169 0.0 0.0 0.0 11.75" Properties=species:S:1:pos:R:3:forces:R:3

C	1.26187087	0.72153677	0.74407192	-0.00758652	-0.00401982	0.00076268
C	1.27450117	2.14732396	0.70244652	0.00371522	-0.00596620	-0.00216902
C	2.50040833	2.86195879	0.89233010	0.01266187	-0.00038429	0.00557890
C	2.50535368	4.28554356	0.95588333	0.01481387	0.00340495	0.01130172
C	1.28272254	4.99142017	0.76868514	0.00591543	0.00713795	0.00341287
C	1.27660573	6.40734222	0.83672989	0.00553516	0.00520868	-0.00121285
C	2.50741535	7.10901892	1.02077020	0.00400630	-0.00927594	0.00111265
C	2.48791931	8.53885422	0.95838660	-0.00061323	-0.00521464	0.00718648
C	3.71327134	0.73622607	1.09423923	0.01045938	0.00742510	0.01471971
C	3.71752985	2.15141013	1.07829529	0.00294595	0.00266134	-0.00946836
C	4.92849722	2.85154113	1.41431450	0.00802824	0.00396067	-0.01079438
C	4.81212431	4.21511601	1.79956436	0.01403192	0.00422998	0.00323440
C	3.68368787	4.97704154	1.38495050	0.00467207	0.01050208	-0.01911663
C	3.71253718	6.41801829	1.35794844	0.00970710	-0.00307487	-0.00458685
C	4.94442339	7.16292704	1.48759807	-0.00681560	-0.00437071	-0.00244674
C	4.94264986	8.55341882	1.22130765	0.00835660	-0.00901240	0.01460081
C	6.18272155	0.75087035	1.12320504	-0.00380321	0.00029223	-0.00148220
C	6.20279025	2.15476298	1.28456159	0.00766126	0.00710495	-0.00201427
C	7.46490451	2.84297774	1.18873385	-0.02546389	-0.00334097	-0.00121839
C	7.64534502	4.17652310	1.67426922	-0.01128766	-0.00433467	0.01165441
C	6.22014742	6.58233682	1.83268143	0.00713390	-0.00985749	0.01138934
C	7.41234954	7.14149079	1.22368310	-0.00417035	-0.00474833	0.00500317
C	7.40043803	8.54629570	0.93338230	-0.00685353	0.00074365	-0.01784573
C	8.62431972	0.71891106	0.71028848	-0.00238612	-0.01527319	0.00328649
C	8.63040627	2.14548196	0.73881011	-0.00993344	0.00034374	-0.00476464

C	9.87896946	2.85422289	0.65224099	-0.00326591	-0.00397456	-0.01409894
C	9.88673721	4.27645115	0.71667794	-0.00818869	0.00454240	-0.01566546
C	8.68505228	4.96305868	1.08345621	-0.01224566	0.01531140	-0.03671352
C	8.64722826	6.39912444	1.03422872	-0.00486166	-0.00882972	-0.02372806
C	9.86864024	7.11294114	0.80994925	-0.00118557	0.00600811	0.00592016
C	9.86494549	8.53473088	0.70737704	-0.00287384	-0.00097672	0.00323296
Cu	6.51227644	4.21009698	5.09213450	-0.00298074	-0.07206329	0.02844949
Cu	4.61789470	5.53680901	4.42846118	-0.02802717	-0.01604988	0.05930776
Cu	7.47529916	6.98810152	3.37818268	-0.00208171	0.06883037	0.00568410
Cu	4.77038851	3.19857156	3.67426946	-0.00326238	-0.01575678	0.02046619
Cu	6.28090603	4.94198054	2.79593736	0.00453770	0.01275097	0.06388036
Cu	8.38339117	4.06239172	3.59667743	-0.01340530	0.01785414	0.10280325
C	7.01938543	6.69940146	6.25007922	0.00699365	0.00333344	0.01213684
O	5.56446769	4.90110716	7.97714768	0.00096866	0.00718577	0.00583105
O	7.49689793	8.02898949	6.48642180	0.00006934	0.00446365	0.00608214
H	3.51507223	3.14137924	7.91908280	0.00412707	0.00167506	-0.01450315
O	3.37611482	3.84018890	7.21267166	0.01040361	0.00251450	-0.00098277
H	4.27689556	4.33191999	7.30313540	0.01091440	0.01250437	-0.02375853
H	1.88134694	8.53016068	2.87448387	-0.00083202	0.00334161	0.01328285
O	1.56122709	8.21717961	3.75813836	0.00058984	0.01051848	-0.02036063
H	2.15581874	7.36279254	4.01128135	0.01132933	-0.00556126	-0.00661897
H	2.31946367	2.29992019	4.85559826	0.01219842	0.01642593	-0.01542367
O	2.89551121	3.09323360	4.67243442	-0.00365654	-0.01067856	-0.01809531
H	3.12392867	3.36672436	5.62442068	0.00068264	0.00434561	-0.00141964
H	7.50499009	3.21555199	9.48159441	-0.00728821	-0.00087654	0.00931397
O	7.09220555	2.31893777	9.72788042	-0.01050542	-0.00865592	0.01460235
H	7.37054140	2.16472731	10.66719289	-0.00432271	-0.00143796	-0.01115480
H	3.78264328	-1.43327779	6.10918772	0.00018681	0.01241558	0.01785300
O	3.57456279	-0.78625872	6.83217446	0.00196166	-0.00652511	-0.00975749
H	4.48328884	-0.55266512	7.35424522	0.00104122	-0.00148602	-0.00591781
H	0.64560292	6.53952094	6.76979348	-0.00306741	0.00977202	-0.00192689
O	1.30679560	5.21421245	6.42882376	0.00198047	0.00593267	-0.00320201
H	1.99084656	4.71000491	6.98185538	0.00471382	0.01163576	-0.00648313
H	5.18295887	1.97214930	7.54995034	0.00594672	0.01223244	-0.01956414
O	5.65304044	1.92988521	6.65323388	-0.00177230	0.00212752	0.00789304
H	5.49215919	2.83739902	6.23174455	0.00516510	-0.00159229	0.00990669
H	8.32594438	10.42090085	8.25460419	-0.00627665	-0.00825479	-0.00848010
O	8.44022710	10.30624564	7.26828375	-0.00757600	-0.00491231	-0.00460623
H	7.47150397	10.28711671	6.99380190	-0.00135569	0.01654206	-0.00934661
H	4.55475995	8.78071354	4.03648199	0.00347506	-0.00675946	-0.00476857
O	5.14676592	9.53507519	4.27668131	-0.01115698	-0.00893856	-0.01139827
H	5.14064421	9.63851518	5.29094480	0.00323373	-0.01502626	0.00459932
H	5.46156744	2.32534945	9.46564486	-0.00276364	-0.00597229	0.00073668
O	4.47146673	2.32842343	9.20073422	0.00857179	-0.00637474	0.00858195
H	4.14517599	1.40246278	9.50323769	0.00531266	0.00058002	0.00562206

H	7.47299441	6.94983100	10.03407151	0.00296424	0.00414270	0.00545223
O	8.45143090	6.80779719	9.77741416	-0.00466409	0.00650321	0.00119681
H	8.85379897	6.60620563	10.66791172	-0.00281060	-0.00011867	0.00066191
H	-0.23416675	-0.46438293	9.33209269	-0.00417886	0.00461260	-0.00025212
O	0.64626440	0.01938853	9.25657508	-0.00042992	0.00386138	0.01228532
H	0.60793291	0.42331478	8.35526311	-0.00071376	0.00279906	0.00349424
O	9.91266097	7.39526116	6.63663466	-0.00241238	-0.01242317	-0.00251834
H	10.53331530	8.19122880	6.45778325	-0.00237360	-0.00712675	-0.00718706
H	5.73506563	-0.91982517	8.85888207	-0.00066379	0.00210859	-0.00837031
O	5.58904634	-0.18060589	8.18905983	0.00978160	-0.00613321	0.00905709
H	6.42171034	-0.16020683	7.61702297	-0.00343260	-0.00080665	0.01126823
H	8.86111701	3.09822049	6.42402212	-0.01118765	0.00908521	-0.02614520
O	8.81963050	4.05747754	6.05940571	-0.00773111	0.00770034	-0.01276871
H	7.73574963	0.28298226	5.08143279	-0.00068757	0.02963084	0.00762436
O	7.78853296	0.56766860	4.09798832	0.00235322	-0.00441662	-0.01654459
H	6.84645906	0.90698040	3.95925817	-0.00396417	0.04033512	-0.00476360
H	1.88503432	5.54687194	5.62354608	0.01101662	0.00173520	-0.00851969
O	2.75009259	6.13661086	4.49590933	0.00519190	-0.00453241	-0.01704922
H	2.44513882	5.47248748	3.82329947	-0.01801314	-0.01035294	-0.01417157
H	8.51558924	4.40060518	7.71976469	-0.00822160	0.00763847	-0.01178311
O	8.11162036	4.47860032	8.64604877	-0.00227144	0.00119649	-0.00036625
H	8.36322445	5.38740579	9.04573236	0.00010631	0.00184812	-0.00640262
H	5.18364445	7.61097230	10.58081633	0.00530462	-0.00257910	0.00025530
O	5.88026525	6.90567042	10.36742052	-0.00212811	-0.00411177	0.01849290
H	5.36901152	6.04876606	10.48748705	0.00350830	0.00208710	0.01561780
H	3.26218637	7.75967620	9.64590841	0.00171245	-0.00021039	0.00703355
O	3.77515872	8.52117324	10.11967253	-0.00445928	-0.00279110	0.01202623
H	3.20098658	8.71809161	10.90171942	0.00264991	-0.00403430	-0.01465761
H	2.48775159	8.94845717	6.36388264	0.00741088	0.00054784	-0.00092180
O	1.64980823	9.44228409	6.07085538	-0.00159656	0.00151036	0.00132326
H	1.55765354	9.09333261	5.11165519	-0.00319943	-0.00260722	-0.00107231
O	9.73202481	2.47025878	3.75230304	0.00336413	-0.00108065	-0.01302847
O	9.38293851	6.56441002	4.09964756	-0.00279969	0.00250489	-0.01113145
H	9.06519000	1.70482001	3.84003624	0.00346883	-0.02852676	-0.00429577
H	10.12547646	2.34488132	2.84963254	0.01780888	-0.01882825	0.00619271
H	9.78565961	4.41092963	6.05602697	-0.01762809	-0.00180943	-0.02115310
H	10.06133637	7.20293549	3.68850680	0.00307658	-0.00753842	-0.00270045
O	2.43214934	6.60416888	8.98191975	0.00418907	-0.01011885	-0.00052074
H	2.81118595	6.86130586	8.07223761	0.00316101	-0.00461958	-0.00250674
H	1.62027880	7.22992830	9.06608267	0.00570220	-0.00781741	0.00367992
O	4.14009169	4.84979053	10.18542053	-0.00131615	0.00337764	0.02140707
H	3.33733173	5.31338158	9.79239938	0.00019831	0.00066783	0.00873448
H	4.03915624	3.85807992	10.03679155	0.00199658	0.00671781	0.01482489
H	5.96142868	6.60884304	6.55930716	0.00417580	-0.01330501	-0.02144014
H	6.53761455	4.69703052	8.17995276	-0.01606726	-0.00484916	-0.01516018

H	5.10118730	4.91545179	8.87959546	0.01130181	0.00449271	-0.01377393
H	7.61907041	5.96467939	6.82187755	-0.00200100	0.00577084	-0.01442159
H	7.07931663	6.41295273	5.16076517	0.00868681	0.00029514	-0.01069759
H	9.49242563	6.82845245	5.07508649	0.00771686	-0.01489707	-0.00824671
H	2.63009259	6.94661086	4.61590933	0.00417580	-0.01330501	-0.02144014

CH_4;*OH

122

Lattice="9.84 0.0 0.0 0.0 8.52169 0.0 0.0 0.0 11.75" Properties=species:S:1:pos:R:3:forces:R:3

C	1.38464602	0.54545964	0.65168673	0.00034929	-0.00580869	-0.00687536
C	1.38743090	1.97199049	0.60418093	0.00326512	-0.00431764	-0.00530008
C	2.61441972	2.68382122	0.78256722	-0.00411398	-0.00728770	0.00163794
C	2.61497761	4.10720344	0.89652207	-0.00037915	0.00637701	0.00465084
C	1.38876603	4.81720779	0.74471239	-0.01028236	0.00731913	0.00269586
C	1.38986285	6.23393948	0.81520065	0.00290737	0.00068090	0.00046758
C	2.62390124	6.93641872	0.96562372	0.00139434	-0.00109651	0.00572840
C	2.60942955	8.36006148	0.83871762	-0.00184106	-0.00547721	0.00740489
C	3.84229337	0.55138471	0.89894470	-0.00053666	-0.00521034	0.00713795
C	3.84553301	1.97439422	0.91168840	0.00510324	0.00172380	-0.01121431
C	5.04648836	2.66804010	1.26137660	0.01815950	-0.00614021	-0.01883520
C	4.93029693	4.00907181	1.74573569	0.01298899	0.00615465	0.01437494
C	3.80509284	4.79934979	1.32384541	-0.00145594	0.03636438	-0.03584385
C	3.83599483	6.24293710	1.29686177	0.00766118	0.00170047	-0.00464390
C	5.08152840	6.98201956	1.38497147	-0.01465162	-0.00750634	-0.00096617
C	5.06864529	8.36462895	1.04707686	0.00691425	-0.01322880	0.01073350
C	6.29585366	0.56528767	0.97075194	0.00004393	-0.00641005	0.00101021
C	6.30254810	1.97386989	1.14165136	0.00397758	0.01307176	0.01489579
C	7.56182458	2.66658965	1.09021783	-0.00659007	0.00538426	0.00059994
C	7.69080093	4.00557410	1.55393218	-0.00919235	0.00177979	0.01376179
C	6.33854665	6.42375104	1.78584595	-0.00890809	-0.00767091	-0.00057021
C	7.52212855	6.98063198	1.19864225	0.00840778	-0.00774740	-0.03779861
C	7.51873607	8.36629050	0.84545550	-0.00109403	-0.00309947	-0.01364292
C	8.74164285	0.54790107	0.63235532	-0.00028998	-0.00217750	-0.01027178
C	8.74189476	1.97364762	0.65371904	-0.00763641	0.00202912	-0.01210723
C	9.98796758	2.68292033	0.57605339	-0.00750462	0.00468207	-0.02114363
C	9.99316269	4.10518408	0.67325536	-0.00646234	0.00283482	-0.02301533
C	8.78483238	4.79621391	1.05281631	-0.00735248	-0.00057579	-0.00167045
C	8.75971961	6.24210835	1.03732527	-0.00568184	0.00234862	-0.01621899
C	9.98189592	6.94073971	0.78891243	-0.00283975	-0.00650455	0.00400173
C	9.98397019	8.36352748	0.65112017	-0.00380724	-0.00045520	-0.00984217
Cu	7.68461744	4.73435826	5.30444998	-0.03520300	0.00551860	-0.00743826
Cu	5.48972913	5.40801335	4.96689757	-0.00516267	0.00798614	0.02437050
Cu	7.24655354	6.66336898	3.78960318	-0.00808316	0.02015405	0.08120486
Cu	4.31721397	3.82392197	3.62436747	0.02570706	0.02557822	0.09504610
Cu	6.37520061	4.74088669	2.77639511	-0.00107760	-0.01092028	0.05100979

Cu	8.75125362	4.39650744	3.22048100	-0.02234962	-0.04031833	0.04315309
C	6.11916888	7.61719217	6.89428878	0.00457849	0.01380350	0.01137181
O	6.01364648	4.62876098	7.98863063	-0.01438894	-0.01195073	0.00924371
H	3.52300835	3.33855084	7.95118731	-0.00321969	0.00237804	-0.01060235
O	3.51033059	3.85622289	7.09024023	-0.00097307	-0.00252113	0.00508626
H	5.11256928	4.47339283	7.58050329	0.01450253	0.00066203	-0.00588065
H	0.53176555	7.79884287	2.97892480	-0.01310568	-0.00375581	0.01088058
O	0.83082932	7.52841370	3.88369260	-0.00699462	0.01206527	-0.01825592
H	1.85677188	7.54293072	3.88523624	-0.00479561	0.00498342	-0.00792317
H	2.23445534	2.35375374	5.03202668	0.00652634	0.01052260	-0.00925510
O	3.24014156	2.73630647	4.81373353	0.00261103	0.00430816	-0.01291299
H	3.44167637	3.12622719	5.75097043	-0.00349524	-0.00203294	0.01437794
H	7.52419003	3.01793645	9.59430290	-0.00288917	0.00010674	0.00500974
O	6.85446448	2.31640218	9.91573511	-0.00600050	0.00200792	0.01360693
H	6.83301649	2.39984393	10.90869947	-0.00516602	0.01047028	-0.00580986
H	3.22684873	-0.53130794	6.18043113	0.00190956	0.00420659	0.00608481
O	2.80594789	0.08217117	6.83796936	0.01968550	-0.01179076	-0.00526553
H	3.53210133	0.71019390	7.07738214	-0.00200931	-0.00880168	-0.01375284
H	0.91320931	6.26918106	6.50599066	-0.00136549	-0.00197835	-0.00687120
O	1.83831286	5.81064793	6.60302277	0.00198932	0.00169759	-0.01455110
H	2.71938145	4.48716541	7.07554770	0.00646611	-0.00531103	-0.00876682
H	5.33687275	2.08867602	7.26940561	0.00992852	0.00813027	-0.01374362
O	5.83547568	2.11903928	6.35761771	-0.00329540	-0.00242624	0.00224086
H	5.71500985	3.03179253	5.95485710	-0.00058427	-0.01794650	-0.00142895
H	8.44561567	9.34569064	7.01624748	0.00078857	0.00557427	-0.00672301
O	8.28973792	9.90019908	6.18730832	0.00438443	-0.00061768	0.00312838
H	7.35455952	10.28532859	6.36164121	0.00368199	0.01437996	-0.00393697
H	4.05037960	8.02627110	4.27761706	0.01511156	-0.01535217	-0.01206135
O	4.88289370	9.26607357	4.28994953	0.00818559	-0.00920000	-0.00810215
H	4.17729779	10.00934807	4.29476054	0.00862763	-0.02795735	-0.00160684
H	5.36200340	2.28685757	9.18008718	-0.00381613	0.00223075	0.00035263
O	4.56182188	1.98512407	8.62189293	0.00550224	0.00303751	0.01025390
H	4.30238116	1.08839471	9.06144107	0.00067901	0.00452440	0.00609114
H	7.32052904	6.30305090	10.13215601	0.00007019	0.00191847	0.01468220
O	8.28176751	6.43287649	9.80787760	0.00465882	0.00673670	0.01016182
H	8.80505080	6.49373814	10.65310668	0.00126985	0.00261912	-0.00243201
H	-0.07498663	0.68398532	9.08836391	0.00144803	0.00084745	0.00550809
O	0.94879633	0.73379307	9.22755583	0.00873181	0.00594755	0.01246784
H	1.28259690	1.01578467	8.34362366	-0.00207846	0.00314333	-0.00004443
H	8.72360125	6.57447546	6.02984259	0.00494333	0.00823756	0.00372380
O	9.49583302	7.17153166	6.27909276	-0.00251883	0.01813780	0.01102809
H	9.79188524	7.49130129	5.36984331	-0.01868413	-0.00438462	-0.00766846
H	8.20578781	-0.53887284	9.15383954	-0.00490884	0.00395035	-0.00156299
O	8.27634893	0.39799846	8.77311111	-0.01775161	-0.00484542	0.00171526
H	7.69101383	1.01177636	9.33453979	-0.00780727	-0.00744440	0.00141592

H	9.18933446	2.90767246	6.16319602	0.00447815	-0.00202200	-0.00114471
O	9.30220301	3.90820544	6.27799602	-0.00001024	0.00324502	0.00062027
H	9.13207963	4.01533011	7.29496221	0.01269655	-0.00628281	0.00035323
H	7.88093651	0.56611467	4.89477939	-0.00000158	0.00399080	-0.01006891
O	7.62956897	0.18791432	3.95446333	-0.00485323	-0.00954791	-0.01835530
H	6.71887561	0.56435495	3.75839825	-0.00483096	0.02892425	-0.00849241
H	2.37695066	6.15820687	5.84225617	0.00396089	-0.00995943	-0.00723246
O	3.36805495	7.21915628	4.36768087	-0.00151501	-0.00057569	-0.01625191
H	3.76254299	6.49395238	3.79684494	-0.00536790	0.00995318	-0.01403063
H	7.49539207	4.22664447	8.23712922	-0.00727075	-0.00113432	-0.01933803
O	8.41314072	4.05660708	8.66851977	-0.00205637	-0.00746964	0.00645781
H	8.55226387	4.94551517	9.15224786	-0.00243510	0.00184677	-0.00340306
H	5.19707954	7.03679297	10.03723077	0.00501144	0.00295469	0.01231921
O	5.70348830	6.16320704	10.16315929	0.00139995	0.00367259	0.01916174
H	4.94845263	5.51764329	10.38290010	0.00797697	0.00199516	0.01294527
H	3.21006030	7.68723073	9.51102144	-0.00086996	0.00369276	0.00958946
O	4.06348345	8.24626413	9.77733034	0.00495486	-0.00023931	0.01294435
H	3.86829006	8.52966237	10.71215841	-0.00371752	-0.00287602	-0.00916054
H	1.63192204	9.44806830	5.97266077	-0.00955727	-0.00286937	-0.00959522
O	1.17517842	10.27748840	5.63120123	0.00048853	-0.01076998	0.00065482
H	0.58435836	10.09233837	4.82070584	-0.00061377	-0.00036158	-0.00102521
O	9.60050369	1.96812668	3.51530724	-0.00261526	-0.00234491	-0.01552138
O	10.59724108	4.80606721	3.94739259	0.01422964	-0.00383392	-0.01911366
H	8.83529317	1.32847478	3.37851630	-0.00415893	0.01042563	-0.00662271
H	10.08937922	1.99192801	2.65280962	-0.00080513	0.00360900	0.01647114
H	10.49127655	4.57424716	4.91803008	0.01966194	0.00022357	0.00038388
H	10.61913531	5.82045681	3.92201697	0.00947791	0.00114818	0.00054808
O	1.93277628	6.88457095	9.26719799	-0.00170704	-0.00909984	0.00744123
H	1.97471206	6.63580061	8.30034844	-0.00408125	0.00718774	0.00299387
H	1.39947318	7.78944938	9.27295780	0.00151764	-0.00878338	0.00608125
O	3.52547619	4.66209856	9.93451075	0.00541959	0.00335936	0.00764835
H	2.82785730	5.38169600	9.88726401	0.00336532	-0.00279195	0.00995096
H	3.19901460	4.06993686	10.65827430	0.00816406	0.00019239	-0.01351618
H	3.83166709	6.21267613	7.58390152	-0.05327554	-0.03787597	-0.07866820
H	5.83639035	5.27273606	8.75216012	-0.00351741	-0.01362830	-0.01308713
H	5.70573445	8.26355780	7.69607892	0.00401086	-0.00029925	-0.00040861
H	5.29586669	9.52397259	5.17971594	-0.00298302	0.00305997	-0.01252120
H	4.30036995	6.50033998	8.12019067	0.05956259	0.03462523	0.07098297
H	5.29919511	7.27592418	6.22054909	0.01230262	0.01127146	-0.00898416
H	6.58968189	6.71846581	7.33449058	0.01374748	-0.00289241	-0.02203179
H	6.86539370	8.16458425	6.30067243	-0.01099955	-0.02776545	-0.01473513

*HCOOH;*OH (reduced CO_2 adsorption pathway)

116

Lattice="9.84 0.0 0.0 0.0 8.52169 0.0 0.0 0.0 11.75" Properties=species:S:1:pos:R:3:forces:R:3:initial_charges:R:1

C	1.19407060	0.77356001	0.81748540	-0.00224144	-0.00270348	-0.00251651	-0.03710000
C	1.19625561	2.20139453	0.74392484	0.00218257	-0.00570553	-0.00152915	-0.02900000
C	2.42424026	2.91391723	0.90272357	0.01060335	0.00157002	0.00562999	-0.00610000
C	2.43149915	4.33824043	0.99469509	0.01501767	0.00429275	0.01276129	-0.00890000
C	1.20537839	5.04478667	0.84715420	-0.00470530	0.00647887	0.00480303	-0.02440000
C	1.20526019	6.45903260	0.94462828	0.00138407	0.00184110	0.00469816	-0.03050000
C	2.44207339	7.16039753	1.11184213	0.00355203	-0.00532068	0.00383920	-0.01270000
C	2.41953760	8.58501680	1.01277266	0.00282945	-0.00402453	0.00266824	-0.06910000
C	3.64507927	0.77717968	1.08917033	0.00410367	0.00301050	0.01421033	-0.05680000
C	3.64161818	2.19495877	1.04871465	0.00397642	0.00494352	-0.01202070	-0.00640000
C	4.84764715	2.89138811	1.36627276	0.01031689	0.00258400	-0.03325223	-0.07550000
C	4.73168995	4.23004496	1.81891464	0.01478350	0.00437367	0.00592944	-0.16170000
C	3.60965177	5.02233322	1.43771794	0.00647553	0.01413862	-0.00882166	0.03080000
C	3.65272353	6.46484813	1.44507153	0.01080788	-0.00540653	0.00620115	-0.02980000
C	4.88537487	7.21926275	1.58848420	-0.01568364	-0.00716639	0.00179234	-0.03170000
C	4.87379218	8.59630049	1.25511268	0.00974871	-0.01089966	0.01520512	-0.03840000
C	6.10937755	0.79114216	1.13931830	-0.00432198	0.00472862	0.00211454	-0.02230000
C	6.11598793	2.19751039	1.22616874	0.00814661	0.00735935	-0.00335737	-0.01210000
C	7.36485750	2.88923024	1.13237734	-0.01608839	0.00835468	-0.00142087	-0.06430000
C	7.50162903	4.22995706	1.59655511	-0.00911740	0.00393411	0.01283718	-0.16730000
C	6.16885371	6.67588443	1.99931303	-0.00065536	-0.00674739	0.00857660	-0.18210000
C	7.35038210	7.21407319	1.36717784	0.00785799	-0.00855719	-0.04175248	-0.03270000
C	7.33907260	8.59550393	1.02192974	-0.00583571	-0.00498076	-0.00712985	-0.01040000
C	8.55812504	0.77042815	0.78498995	0.00014579	-0.00205340	-0.00202212	0.03850000
C	8.55807879	2.19344079	0.76553220	-0.00726535	0.00196001	0.00179061	-0.06810000
C	9.79997475	2.90940734	0.68971827	-0.00772929	0.00303494	-0.01766277	-0.01050000
C	9.80822068	4.33432970	0.77245492	-0.00959604	0.00489433	-0.01751504	-0.02800000
C	8.59745939	5.02937436	1.12693669	-0.01034078	0.00104052	-0.00370110	-0.09990000
C	8.57221551	6.46940504	1.16253177	-0.00732039	0.00197844	-0.01571625	-0.01860000
C	9.79881311	7.16970402	0.93618797	-0.00410173	-0.00743355	0.01266596	-0.04270000
C	9.79809594	8.59006117	0.81736337	-0.00380291	-0.00076928	0.00220188	-0.02730000
Cu	6.73752022	4.36374334	5.07696120	-0.02981734	-0.03828713	0.00359832	0.27990000
Cu	4.72482015	5.52358445	4.69947674	0.01124390	0.01421888	0.03098111	0.38970000
Cu	7.08095101	6.85909872	3.93433823	0.00837690	0.03612135	0.04222051	0.57300000
Cu	4.76614633	3.25956237	3.72579785	0.01188696	0.02457867	0.02905230	0.47170000
Cu	6.17411108	4.95705172	2.78389171	0.00659757	-0.01171776	0.06252617	0.26930000
Cu	8.48907475	4.51061465	3.29524958	-0.02160265	-0.02318014	0.04206568	0.56430000
C	5.99991655	5.82816124	6.40549376	-0.00552775	-0.00846065	0.00273554	0.86530000
O	5.93465364	5.20283374	7.65676902	-0.00558414	0.00235949	0.02459528	-1.10730000
O	6.96625766	6.76720646	6.21155085	0.01823759	0.00933898	0.03795420	-1.10160000
H	3.63861828	3.32612024	8.05413075	0.00309649	0.00502916	-0.01701813	0.62940000
O	3.48661799	4.10451631	7.43371065	0.01303692	0.00290798	-0.00059905	-1.26130000
H	4.35824469	4.59054352	7.59933039	0.00714411	0.00434182	-0.01994231	0.63070000
H	1.03056402	8.30727573	3.20399552	-0.00784454	-0.00095260	0.01078997	0.59910000
O	1.17750693	7.98542974	4.12695274	0.00265971	0.00909015	-0.02167054	-1.23050000

H	1.87406957	7.24278433	4.07115830	0.00803628	-0.00036897	-0.00763142	0.62280000
H	2.42511641	2.28440528	5.28755690	0.01761807	0.01565043	-0.01355996	0.61860000
O	2.96144596	3.07428502	4.89465807	0.00487892	-0.01027596	-0.01246512	-1.24730000
H	3.22146637	3.51920914	5.76230476	-0.00358835	-0.00277195	0.00706801	0.61390000
H	7.60834528	3.12990982	9.36881356	-0.00806916	-0.00354376	0.00231323	0.63480000
O	7.10984076	2.34517102	9.77063814	-0.00065613	-0.00910310	0.00976732	-1.21880000
H	7.14695274	2.52712210	10.74563304	-0.00591416	0.00383228	-0.00400676	0.58700000
H	4.02850142	-0.69023183	5.97411423	0.00799030	-0.00332922	-0.00523375	0.55430000
O	3.78816769	-0.33115769	6.86140436	-0.00730316	0.00249711	-0.01164762	-1.21440000
H	4.77907601	-0.22501085	7.39319883	0.00830840	0.00025363	-0.00182598	0.62600000
H	0.77721495	6.10734732	6.84209960	0.00415858	0.00351515	-0.00194401	0.63120000
O	1.36158980	5.34981058	6.50786741	-0.00181547	0.00474441	-0.00063572	-1.24180000
H	2.01018000	5.05582437	7.21865833	-0.00086148	0.00435206	-0.01509978	0.64590000
H	5.21468091	2.09589881	7.55860107	0.00341873	0.01251474	-0.01826972	0.63430000
O	5.66378565	2.10492001	6.62213722	-0.00341839	-0.00189703	0.00700873	-1.21040000
H	5.53062377	3.03839098	6.27912498	-0.00099040	0.00163832	0.00919979	0.53350000
H	8.63546941	9.15760344	6.97431572	-0.01244576	0.00808871	-0.00158966	0.62120000
O	8.29055874	9.94404852	6.46842810	-0.00724974	-0.00602020	0.00407173	-1.24990000
H	7.35048888	10.13199912	6.78822768	-0.00407333	0.00473091	-0.00750300	0.64430000
H	4.41917250	9.13060814	4.04142635	0.00303209	-0.02206671	-0.00393000	0.59250000
O	5.20022880	9.68440981	4.27759378	-0.00504264	-0.00158998	-0.01067600	-1.21180000
H	5.19041833	9.85990487	5.29635966	0.01030044	-0.01968894	0.01174758	0.63420000
H	5.67954297	2.36954086	9.36859683	-0.00438168	-0.00393053	-0.00187955	0.63230000
O	4.67707288	2.34775209	9.02963723	0.00054454	-0.00779345	0.00549878	-1.27700000
H	4.32787974	1.45565305	9.39752590	0.00481552	-0.00175486	0.00252719	0.63780000
H	7.71220386	6.96566706	10.02920289	0.00486342	0.00269116	0.00543132	0.64660000
O	8.70330045	6.80505705	9.80410663	-0.00181474	0.00382590	0.00581992	-1.22660000
H	9.09639428	6.67716708	10.70747127	-0.00269031	-0.00103999	0.00027842	0.58570000
H	-0.01464193	-0.43673440	9.27757151	-0.00119460	0.00199823	0.00115142	0.62930000
O	0.86547378	0.04985974	9.21200380	0.00096414	0.00225536	0.01133993	-1.21770000
H	0.84506874	0.47619610	8.31556024	-0.00021127	-0.00626305	0.00424160	0.61140000
H	8.53621712	6.95198422	6.69704936	-0.00842262	-0.01218154	-0.01185247	0.63810000
O	9.44551029	7.38921469	6.86261244	0.00271384	-0.00913750	-0.00147579	-1.22470000
H	9.69960826	7.56306394	5.91807526	-0.01413786	-0.00456055	0.00455384	0.60100000
H	6.02657170	-0.79610562	8.77186145	-0.00310761	0.00314782	-0.01203020	0.62310000
O	6.00795104	-0.17493115	7.96137460	0.00680038	-0.00172649	0.00622149	-1.23130000
H	6.51523164	-0.71642320	7.24490544	-0.00653506	0.00913799	0.00266416	0.61160000
H	9.00217732	3.22985502	5.96046136	0.00635533	0.01028815	-0.01239932	0.64420000
O	8.56948277	4.11592450	6.10566595	-0.01236873	-0.00040590	0.00010902	-1.22400000
H	8.00035401	0.78869758	5.04722470	-0.00884589	-0.00198691	-0.01377592	0.62420000
O	7.86108562	0.52226387	4.05644642	-0.00031238	-0.01143501	-0.01945080	-1.24020000
H	6.93235728	0.86265459	3.88455381	-0.00417054	0.03953373	-0.00785065	0.61810000
H	2.11514754	5.65663770	5.44399338	-0.00164695	0.00025338	-0.00445406	0.63440000
O	2.72308312	5.88209779	4.51417325	0.01272618	0.00403968	-0.01344080	-1.20760000
H	2.46589262	5.12201652	3.92648860	-0.02168674	-0.00907287	-0.02061026	0.61630000

H	8.60779778	4.39408374	7.70492265	-0.01163569	0.01064778	-0.01365846	0.62960000
O	8.18104339	4.52239717	8.62409754	-0.00300651	0.00574315	-0.00118397	-1.25020000
H	8.54056847	5.37243466	9.07517820	-0.00361010	0.00221761	-0.00844102	0.63590000
H	5.49438163	7.63171756	10.55386575	0.00539119	-0.00042637	0.00654144	0.62340000
O	6.13260112	6.94536542	10.19216628	-0.00250491	-0.00083907	0.01645393	-1.25480000
H	5.55300480	6.08884833	10.18290090	0.00439436	0.00138008	0.00661778	0.64210000
H	3.47881770	7.83351728	9.59949419	0.00216605	0.00110859	0.00501626	0.62050000
O	4.00375159	8.58047523	10.08781231	-0.00022748	-0.00081301	0.01110581	-1.22450000
H	3.43052763	8.80535627	10.86247905	-0.00137948	-0.00620381	-0.01116275	0.58790000
H	2.53153552	9.19810054	6.54523225	0.00638732	-0.00374696	0.00219875	0.62320000
O	1.70431082	9.71230213	6.24802158	-0.00274755	-0.00458090	-0.00550992	-1.23830000
H	1.36902845	9.14447420	5.49305308	-0.00359572	-0.00074860	-0.00062685	0.61280000
O	9.59944494	2.64600893	3.74309198	0.00279190	0.00060446	-0.01854615	-1.20410000
O	9.12812555	5.93232125	4.48655965	0.01101448	0.00463872	-0.02962239	-1.19980000
H	8.95763062	1.86157267	3.67420908	-0.00654221	-0.00134881	-0.00676309	0.61950000
H	10.24362572	2.50133851	3.00578226	0.00803276	-0.00822422	0.01125178	0.59160000
H	9.08460228	4.85452917	5.54921153	0.01223355	0.01104243	0.01376910	0.64520000
H	9.81639644	6.54225042	4.11211393	-0.00333375	0.00557797	-0.00293925	0.58730000
O	2.66255292	6.75603445	8.82254348	0.01038945	-0.00890762	-0.00501248	-1.25190000
H	3.03855052	7.10996801	7.95038041	0.00577477	-0.00666259	-0.00423598	0.61080000
H	1.84148260	7.38204815	8.96680592	0.00471620	-0.00669095	0.00069465	0.62190000
O	4.37823301	5.02756333	10.25041480	0.00270841	-0.00314267	0.01085480	-1.23360000
H	3.65296288	5.45566378	9.70809697	0.00351489	-0.00005764	0.00665675	0.62800000
H	4.45779425	4.09599875	9.90513473	0.00501690	0.00715088	0.01447635	0.61330000
H	4.93852369	6.28820638	6.33975250	-0.00037163	-0.01899001	-0.02041843	0.00720000
H	6.89062434	4.94018804	8.04156966	-0.01517738	0.00008902	-0.02008785	0.62900000

*CHO;*OH (reduced CO_2 adsorption pathway)

117

Lattice="9.84 0.0 0.0 0.0 8.52169 0.0 0.0 0.0 11.75" Properties=species:S:1:pos:R:3:forces:R:3:initial_charges:R:1

C	1.19488624	0.71325254	0.81600584	-0.00283976	-0.00202119	-0.00281830	-0.02820000
C	1.19763527	2.13669972	0.73343648	0.00307121	-0.00593346	-0.00323740	-0.02630000
C	2.42381515	2.84828113	0.90011990	0.00520059	0.00055781	0.00448763	-0.00870000
C	2.42716788	4.27198941	0.94643097	0.01520002	0.00449284	0.01023526	-0.00840000
C	1.20993357	4.98320177	0.76660742	-0.00463133	0.00581398	0.00057830	-0.01430000
C	1.20720889	6.39903940	0.87818274	0.00215499	0.00318374	0.00172030	-0.02520000
C	2.43631106	7.10057601	1.06750085	0.00394889	-0.00565183	0.00379220	-0.03040000
C	2.41976639	8.52663147	1.01668966	0.00369947	-0.00434758	0.00379859	-0.05020000
C	3.64589546	0.71787785	1.13843794	0.00191545	-0.00297628	0.01729685	-0.05050000
C	3.64313910	2.13554147	1.09011934	0.00526410	0.00304299	-0.00914977	-0.02430000
C	4.84774195	2.83858822	1.40252345	0.01443578	-0.00678360	-0.05318708	-0.03880000
C	4.69827568	4.18568527	1.83705855	0.01552285	0.00369283	0.00658137	-0.17530000
C	3.59627444	4.95904847	1.39367797	0.00448261	0.01830262	-0.01006117	-0.03240000
C	3.64193733	6.39651558	1.37131641	0.01083179	-0.00430081	0.00195453	0.00770000
C	4.87886135	7.13049122	1.50198535	-0.01331034	-0.00483489	-0.00789587	-0.02260000

C	4.87611434	8.53408000	1.28301600	0.00923015	-0.00937187	0.01832514	-0.02180000
C	6.11381375	0.73124255	1.18906815	-0.00077811	-0.00133756	0.00431362	-0.04850000
C	6.11765333	2.14522532	1.27970236	0.00830484	0.00552610	-0.00023316	-0.01350000
C	7.37165043	2.83534958	1.15079006	-0.01406822	0.00645714	-0.00101350	-0.03670000
C	7.53390197	4.18716902	1.57216325	-0.01174662	0.00368998	0.01503870	-0.19270000
C	6.15156188	6.53852409	1.80932849	-0.00320667	-0.01258993	-0.00718978	-0.17240000
C	7.35975961	7.12979988	1.31488202	0.00704778	-0.00580119	-0.05242432	-0.04040000
C	7.34211014	8.53665570	1.04866181	-0.00526807	-0.00054695	-0.00937760	0.00220000
C	8.55790955	0.71102281	0.79931032	-0.00125857	-0.00233246	-0.00050644	-0.01670000
C	8.55654443	2.13411485	0.75960706	-0.00933560	0.00137455	-0.00079815	-0.02410000
C	9.80293356	2.84446228	0.65176511	-0.00806829	0.00276844	-0.01924853	-0.02860000
C	9.81734369	4.26859196	0.69786456	-0.01119948	0.00419853	-0.02066905	-0.02590000
C	8.61480043	4.96348420	1.06257306	-0.01117753	0.00255073	-0.01040582	-0.09180000
C	8.58516908	6.40058386	1.09050566	-0.00845656	0.00193881	-0.01762137	-0.02130000
C	9.80354204	7.10994394	0.88956527	-0.00340241	-0.00684887	0.01227226	-0.05320000
C	9.79937662	8.53042144	0.80353400	-0.00405460	0.00000174	0.00234411	-0.02910000
Cu	6.75847704	4.41287029	5.10188714	-0.02929424	-0.04132672	-0.00229137	0.27600000
Cu	4.81129808	5.77503846	4.59448148	0.00661773	0.00096025	0.04453890	0.43960000
Cu	7.04344320	6.87375261	3.83568967	-0.00238380	0.01771851	0.10705639	0.57810000
Cu	4.70056296	3.42685364	3.85007343	0.02052916	0.02251432	0.05113230	0.44870000
Cu	6.17829800	4.91345694	2.74275604	0.00792722	-0.00852764	0.05324040	0.27550000
Cu	8.48756102	4.47527667	3.28072311	-0.02185963	-0.02621547	0.03936079	0.55180000
C	6.39264401	6.40610808	5.69797127	0.00050653	0.00480981	0.00016527	0.34590000
O	5.85751262	4.89492084	8.04487491	-0.00068057	0.00719101	0.00903660	-1.26300000
O	7.29518462	6.87064016	6.51993024	0.01043064	-0.00021881	0.00822926	-1.06840000
H	3.60730799	3.33937791	7.95432365	0.00409980	0.00345997	-0.01502678	0.62610000
O	3.51454241	4.10405411	7.31112272	0.01508411	0.00544946	-0.00053799	-1.25060000
H	4.46161946	4.48486063	7.39190788	0.00844733	0.01237606	-0.02387998	0.62690000
H	1.04887972	8.32162000	3.18664491	-0.00782609	-0.00339469	0.00936184	0.60420000
O	1.14563816	7.98144513	4.10927589	-0.00307683	0.00790867	-0.02121046	-1.24220000
H	1.90732106	7.27737023	4.10013379	0.01132479	-0.00172999	-0.00902179	0.62490000
H	2.46844931	2.33328784	5.23379369	0.01227047	0.01265464	-0.01096614	0.62550000
O	2.96489025	3.17171630	4.87530966	0.00505157	-0.00443078	-0.01852178	-1.24490000
H	3.20840933	3.58289530	5.77233408	0.00400243	0.00635455	0.00129650	0.62070000
H	7.63160050	3.06607714	9.39850486	-0.00654837	-0.00525904	0.00385900	0.63910000
O	7.07438478	2.30042940	9.76415046	-0.00266877	-0.00905826	0.00976438	-1.20560000
H	7.16286943	2.41439803	10.74519052	-0.00649425	0.00354575	-0.00524210	0.56690000
H	3.89052248	-0.93371797	6.04412391	-0.00167876	0.01296540	0.00782829	0.55390000
O	3.68270991	-0.41299443	6.85791370	-0.00646570	-0.00194364	-0.01224159	-1.21800000
H	4.66374645	-0.31853257	7.38880423	0.00516151	0.00110218	-0.00450573	0.62960000
H	0.87262589	6.22384352	6.71717167	0.00331022	0.00300502	-0.00303812	0.64160000
O	1.45789271	5.40507449	6.42981783	-0.00125238	0.00088664	-0.00299159	-1.24340000
H	2.11281152	5.10034493	7.13326768	-0.00003669	0.00696409	-0.01460023	0.65140000
H	5.21583818	2.07930787	7.59035436	0.00337616	0.00832568	-0.01622639	0.63630000
O	5.67445735	2.05310558	6.67137401	-0.00545354	-0.00385025	0.00856057	-1.20710000

H	5.67507213	3.00679629	6.35564869	-0.00346096	0.00043157	0.00620909	0.53110000
H	8.67192810	9.18080239	7.00527521	-0.01604292	0.00907553	-0.00069202	0.61620000
O	8.25896462	9.88443559	6.44243212	-0.00391442	-0.00735496	0.00212270	-1.23950000
H	7.32365209	10.05541570	6.79424527	-0.00436846	0.00051957	-0.00510665	0.64290000
H	4.43696279	9.11430893	4.05163083	0.00225885	-0.01104179	-0.00281421	0.60200000
O	5.18293039	9.71547196	4.28745859	-0.00560562	-0.00282997	-0.01114931	-1.23970000
H	5.16493288	9.85996638	5.30423781	0.00724676	-0.02214608	0.00826560	0.64550000
H	5.60786776	2.37061855	9.40864076	-0.00436265	-0.00514653	0.00012032	0.63480000
O	4.59712186	2.35059773	9.10396825	0.00121507	-0.00710888	0.00632164	-1.28080000
H	4.26736745	1.44808029	9.45183954	0.00476739	-0.00036308	0.00259072	0.63840000
H	7.66849612	7.00196357	10.00132666	0.00150982	0.00246358	0.00591769	0.64500000
O	8.64908738	6.84776719	9.77634574	-0.00570036	0.00304019	0.00561975	-1.21480000
H	9.03219696	6.69816523	10.68240580	-0.00355024	-0.00124019	0.00096066	0.58100000
H	-0.04460288	-0.42900812	9.30693806	-0.00093991	0.00128991	0.00064585	0.63000000
O	0.84205848	0.04567296	9.22799918	0.00221655	0.00256683	0.01189815	-1.21320000
H	0.82673078	0.42960829	8.31502941	0.00600030	-0.00117548	0.00067629	0.60470000
H	8.84400183	7.03922568	6.53996324	-0.00595047	-0.00746655	-0.00828414	0.63970000
O	9.77601929	7.43187126	6.73306618	0.00608919	-0.00824303	-0.00854208	-1.21810000
H	10.03321807	7.74349589	5.82420506	-0.01069976	-0.00603118	0.00133056	0.60290000
H	5.83061033	-0.83290775	8.87010448	-0.00059530	0.00117876	-0.01155559	0.61200000
O	5.85136031	-0.24394016	8.04991583	0.00546720	-0.00198825	0.00488625	-1.22840000
H	6.50827183	-0.77690419	7.45869160	-0.00284765	0.00175700	0.00057510	0.62530000
H	8.92945648	3.18802408	6.02698622	0.00907441	0.00696866	-0.01392274	0.64040000
O	8.55767358	4.10132219	6.18286840	-0.01276815	0.00060056	0.00278596	-1.22170000
H	7.94945050	0.62459027	5.04882932	-0.00385932	0.00160161	-0.00886828	0.61920000
O	7.73852257	0.38364215	4.06642409	0.00169482	-0.01406615	-0.01852667	-1.22730000
H	6.81191155	0.78103514	3.96776129	-0.00273553	0.03665708	-0.00622489	0.62720000
H	2.12060557	5.71020604	5.51054161	0.00793697	-0.00105377	-0.00253949	0.62940000
O	2.81630169	6.06207582	4.49273580	0.01075249	0.00498683	-0.01559061	-1.19080000
H	2.58450266	5.33275645	3.85994731	-0.02044069	-0.00881052	-0.01551518	0.60230000
H	8.65651214	4.35402150	7.79480316	-0.01072930	0.01142371	-0.01230895	0.63190000
O	8.37678890	4.40953899	8.77029554	-0.00186428	0.00768853	0.00250115	-1.26630000
H	8.59818334	5.33628885	9.12044528	-0.00309411	0.00248389	-0.00689984	0.63170000
H	5.33679894	7.66100851	10.60574277	0.00703133	-0.00184844	0.00011906	0.62930000
O	6.04077446	6.96864319	10.37409309	-0.00219923	-0.00574647	0.01938488	-1.25590000
H	5.53179622	6.10953308	10.47291322	0.00444226	0.00117302	0.01572702	0.63190000
H	3.42950138	7.80417746	9.63889105	0.00130655	-0.00087183	0.00693622	0.62130000
O	3.95134430	8.54741125	10.14022360	-0.00439608	0.00044664	0.01441923	-1.22880000
H	3.37689482	8.75461696	10.91935488	-0.00119647	-0.00486751	-0.01273988	0.59480000
H	2.53210469	9.20292195	6.42567553	0.00557133	-0.00429034	0.00231726	0.62740000
O	1.75535219	9.76749261	6.08906263	-0.00466187	-0.00748543	0.00137561	-1.23900000
H	1.42358599	9.21675671	5.31753280	-0.00308192	-0.00044107	0.00149607	0.60720000
O	9.53530147	2.52659970	3.74678921	-0.00062321	-0.00185940	-0.01541775	-1.19950000
O	9.15708346	5.84011656	4.48298592	0.01472358	0.00984789	-0.02806121	-1.19710000
H	8.88008143	1.76432359	3.65383218	-0.00690739	0.00282350	-0.00789421	0.62120000

H	10.15749123	2.40513270	2.98672839	0.00828090	-0.00969303	0.01146102	0.58670000
H	9.08885329	4.79602772	5.58767615	0.01700990	0.01089823	0.01816051	0.64090000
H	9.84485579	6.45967368	4.12814170	-0.01138911	0.00753380	-0.00236332	0.57990000
O	2.63180914	6.72825274	8.85497858	0.01083386	-0.01120105	-0.00041765	-1.25770000
H	3.00789327	7.09621645	7.98016405	0.00513307	-0.00548628	-0.00279620	0.61370000
H	1.80564316	7.34350521	8.99796320	0.00515459	-0.00713945	0.00180498	0.62250000
O	4.26186911	4.88960391	10.20242514	-0.00175751	0.00252258	0.02042029	-1.24900000
H	3.52581741	5.37050134	9.71618491	-0.00001731	0.00124806	0.00684505	0.63060000
H	4.15581231	3.90991597	10.00922930	0.00311308	0.00683504	0.01515951	0.62890000
H	5.37852989	6.43844791	6.27002664	-0.00426646	-0.00469486	-0.01546901	0.01710000
H	6.83693174	4.71489134	8.25911861	-0.01686350	0.00496303	-0.02039293	0.61830000
H	5.39670940	4.90727007	8.93946384	0.01213156	0.00451486	-0.01499136	0.59940000

*CH_2O;*OH (reduced CO_2 adsorption pathway)

118

Lattice="9.84 0.0 0.0 0.0 8.52169 0.0 0.0 0.0 11.75" Properties=species:S:1:pos:R:3:forces:R:3:initial_charges:R:1

C	1.20512291	0.73952323	0.82499093	-0.00241387	-0.00213834	-0.00241234	-0.03830000
C	1.20554475	2.16553891	0.74589926	0.00328598	-0.00591877	-0.00253012	-0.01480000
C	2.43091329	2.87733224	0.91930091	0.00635180	0.00078637	0.00467251	-0.01250000
C	2.43772416	4.29928441	1.01161146	0.01457933	0.00486704	0.01358710	-0.00760000
C	1.21925505	5.01083357	0.83774847	-0.00178861	0.00645790	0.00058286	-0.02210000
C	1.21958693	6.42525816	0.93772565	0.00243632	0.00214880	0.00278208	-0.03020000
C	2.45228564	7.12378225	1.12929628	0.00406119	-0.00592266	0.00483607	-0.01240000
C	2.43026716	8.55029743	1.03438098	0.00392032	-0.00430868	0.00344209	-0.07340000
C	3.65607741	0.73994772	1.12048009	0.00213660	-0.00083640	0.01498902	-0.05650000
C	3.64934959	2.15745468	1.06719403	0.00482234	0.00359409	-0.01186941	-0.01030000
C	4.85468439	2.85683468	1.36757207	0.01424184	-0.00207110	-0.05267716	-0.05930000
C	4.73004618	4.18726999	1.83579676	0.01483403	0.00424003	0.00636514	-0.17860000
C	3.61271182	4.98262190	1.46244960	0.00424222	0.01763261	-0.00473600	0.02580000
C	3.66022123	6.42541188	1.46145564	0.00925789	-0.00530882	0.00492521	-0.02190000
C	4.90095780	7.17150729	1.57835683	-0.01274752	-0.00478013	-0.00392801	-0.02420000
C	4.88755869	8.55940435	1.27682979	0.00907217	-0.01002287	0.01636848	-0.03130000
C	6.12122160	0.75534490	1.14769897	-0.00207213	-0.00020342	0.00263573	-0.01300000
C	6.12274442	2.16538306	1.22503702	0.00810669	0.00758262	-0.00342182	-0.01340000
C	7.36866999	2.86002409	1.10696698	-0.01482420	0.00947180	-0.00245239	-0.03800000
C	7.51210524	4.21326093	1.52953128	-0.01208422	0.00400579	0.00960509	-0.17120000
C	6.18936591	6.61078902	1.91836807	-0.00500030	-0.01028142	-0.00659807	-0.17900000
C	7.36796981	7.17543135	1.33517335	0.00528554	-0.00850942	-0.02391351	-0.02460000
C	7.35079250	8.56542981	1.01915615	-0.00608905	-0.00557207	-0.00397743	-0.00210000
C	8.56883738	0.73902733	0.78113741	-0.00068185	-0.00225877	-0.00067956	-0.01160000
C	8.56668386	2.16076059	0.74889915	-0.00808957	0.00058730	-0.00040497	-0.00760000
C	9.81079081	2.87521908	0.66870577	-0.00749958	0.00173174	-0.02061822	-0.02200000
C	9.82568454	4.29924084	0.73732890	-0.00876198	0.00424897	-0.02304262	-0.02260000
C	8.61146819	5.00153898	1.06746606	-0.01260760	0.00061848	-0.00240664	-0.09470000
C	8.59080998	6.43747343	1.11032821	-0.00959596	0.00328063	-0.01582336	-0.02970000

C	9.81552691	7.13758680	0.91627948	-0.00495559	-0.00719883	0.01130857	-0.05430000
C	9.81073664	8.55901022	0.81337948	-0.00367737	-0.00059619	0.00148005	-0.02530000
Cu	6.78656836	4.11768631	4.92247477	-0.03767716	-0.04906774	-0.00446958	0.25930000
Cu	4.87365883	5.70254451	4.67278004	0.00342053	-0.00055360	0.02958302	0.36210000
Cu	7.08334261	6.74437039	3.98792291	-0.00651738	0.02706095	0.06963354	0.60760000
Cu	4.72233881	3.33195621	3.78783273	0.02404647	0.02167710	0.04625306	0.45510000
Cu	6.20548632	4.91882770	2.74785206	0.01105558	-0.00910946	0.06188793	0.28290000
Cu	8.56447253	4.46772276	3.21504619	-0.02925206	-0.00705328	0.04845860	0.59350000
C	6.35028731	6.24963904	5.99348556	-0.00103978	0.00034768	-0.00693877	0.19220000
O	5.73932450	4.85958235	8.25742809	0.00040723	0.00189814	0.00934945	-1.27150000
O	7.08866251	7.41766893	6.03222141	0.00444627	0.01357224	0.00975141	-1.06760000
H	3.53747007	3.30404141	7.90506875	0.00418430	0.00410148	-0.01383867	0.61270000
O	3.49290135	4.09530517	7.29275578	0.01414068	0.00497993	0.00030624	-1.24670000
H	4.42400570	4.47922204	7.50767435	0.00483787	0.00952321	-0.02207781	0.63290000
H	1.00371258	8.27826661	3.19518883	-0.00825266	-0.00155719	0.01076090	0.59750000
O	1.15090974	7.96705762	4.12108144	-0.00091456	0.00783919	-0.02272813	-1.22820000
H	1.89001416	7.25723057	4.08515580	0.00864118	-0.00111681	-0.00836897	0.62410000
H	2.47783919	2.30479676	5.23025525	0.01326357	0.01296086	-0.01168445	0.62600000
O	2.97393974	3.13615178	4.86092487	0.00496622	-0.00941098	-0.01774469	-1.24520000
H	3.21454810	3.57072646	5.75048027	0.00169537	0.00750226	0.00284746	0.61960000
H	7.63291538	3.02498280	9.35029415	-0.00590837	-0.00553013	0.00227456	0.63430000
O	7.07048843	2.29228862	9.76126928	-0.00339759	-0.00880314	0.00948982	-1.20480000
H	7.15709921	2.46871717	10.73126239	-0.00684034	0.00479997	-0.00505266	0.56880000
H	3.94267543	-0.92505364	6.03962771	-0.00164315	0.01382958	0.00507680	0.55300000
O	3.69804889	-0.42601751	6.85672391	-0.00453211	-0.00119716	-0.01171625	-1.21630000
H	4.66071369	-0.29550731	7.40256932	0.00464353	0.00172171	-0.00452524	0.63180000
H	0.76008981	6.14716333	6.78183302	0.00206326	0.00357023	-0.00268080	0.63270000
O	1.37921421	5.40169755	6.46199503	-0.00375736	0.00295690	-0.00081152	-1.24410000
H	2.01173409	5.11414292	7.18585234	-0.00097081	0.00405846	-0.01457328	0.65010000
H	5.19434634	2.07985333	7.57386539	0.00460532	0.01056828	-0.01808650	0.63570000
O	5.65401813	2.06874978	6.65456441	-0.00239675	-0.00042455	0.00851651	-1.21030000
H	5.51333970	2.99384229	6.29557879	0.00214891	0.00040669	0.01543239	0.53320000
H	8.68972056	9.16645139	6.95269508	-0.01228039	0.00829427	-0.00206686	0.61060000
O	8.33908271	9.95046578	6.44859854	-0.00644441	-0.00726900	0.00176776	-1.22900000
H	7.39510170	10.11069891	6.75918964	-0.00565259	0.00657569	-0.00785794	0.62980000
H	4.43064587	9.07036736	4.06262870	0.00575693	-0.01231097	-0.00237004	0.59500000
O	5.19061488	9.66399585	4.26926951	-0.00440775	-0.00721728	-0.01071550	-1.22190000
H	5.20465769	9.80250229	5.28492190	0.01036470	-0.02188863	0.00847982	0.63600000
H	5.57426060	2.37391192	9.39014993	-0.00437304	-0.00527712	-0.00032614	0.63170000
O	4.56980671	2.34212982	9.09372800	0.00191784	-0.00767469	0.00705349	-1.27540000
H	4.26514937	1.43163857	9.43559598	0.00506064	0.00076927	0.00193678	0.63540000
H	7.69534286	7.00183374	10.01059128	0.00178474	0.00333796	0.00609462	0.63990000
O	8.66967308	6.81824596	9.77420385	-0.00478478	0.00279559	0.00533417	-1.21540000
H	9.05804914	6.64974005	10.67307890	-0.00259574	-0.00083374	0.00173034	0.58570000
H	-0.03127997	-0.44356498	9.28632878	-0.00063409	0.00229062	0.00139728	0.63480000

O	0.85520833	0.03297783	9.22329665	0.00213523	0.00295370	0.01141859	-1.22340000
H	0.83944536	0.44107050	8.32039038	0.00456042	-0.00262778	-0.00005541	0.61110000
H	8.55774664	7.21521300	6.52702395	-0.00132193	-0.01323709	-0.01139612	0.62930000
O	9.53350786	7.43049253	6.83361611	0.00303953	-0.01397322	-0.00511235	-1.21840000
H	9.90465787	7.72407148	5.96134632	-0.01227037	-0.00686499	0.00232265	0.58970000
H	5.95826101	-0.90120981	8.73102923	-0.00365837	0.00032541	-0.01217843	0.61050000
O	5.86612542	-0.18144953	8.03560993	0.00884505	-0.00578213	0.00810822	-1.22190000
H	6.46834055	-0.47444364	7.24315779	-0.00804903	0.00247589	0.00537650	0.62270000
H	9.01330231	3.15639397	6.01476455	0.01023087	0.00709544	-0.01267955	0.64890000
O	8.56581590	4.04707034	6.09001713	-0.00681679	-0.00308052	-0.00014216	-1.22840000
H	8.02656285	0.75674778	4.92531861	-0.00553614	0.00341333	-0.01966357	0.61700000
O	7.89531298	0.61158596	3.92254097	-0.00004914	-0.00857661	-0.01708154	-1.23350000
H	6.93332709	0.88117630	3.81950480	-0.00328360	0.02820105	-0.00566795	0.62000000
H	2.16591880	5.73993742	5.45789241	0.00871486	0.00005180	-0.00686266	0.62890000
O	2.80833750	5.98936817	4.53801381	0.01194900	0.00424888	-0.01424803	-1.20630000
H	2.58082680	5.21691767	3.95340313	-0.02281030	-0.00632194	-0.01856755	0.60760000
H	8.59087538	4.35649100	7.70213896	-0.00783186	0.00855430	-0.01136750	0.62410000
O	8.30876527	4.42637112	8.67509100	-0.00366389	0.00858993	-0.00347197	-1.25890000
H	8.57513263	5.33600352	9.04142212	-0.00328740	0.00139702	-0.00812977	0.63220000
H	5.36385501	7.69782120	10.50823526	0.00511206	-0.00132066	0.00443561	0.63220000
O	6.08408591	6.99191935	10.35995815	-0.00496732	-0.00705754	0.01893453	-1.24600000
H	5.60291573	6.14830671	10.58045374	0.00504402	0.00224008	0.01665171	0.61790000
H	3.45015109	7.77514690	9.63682633	0.00265599	-0.00058492	0.00652579	0.62130000
O	3.97804064	8.52316671	10.12693492	-0.00108506	-0.00118695	0.01266187	-1.22560000
H	3.41899394	8.72593817	10.91822711	-0.00216403	-0.00504395	-0.01177878	0.59430000
H	2.54432185	9.17113873	6.45018838	0.00535010	-0.00362954	0.00243500	0.61960000
O	1.76078952	9.73474434	6.12566789	-0.00480814	-0.00633789	-0.00028514	-1.23920000
H	1.40646786	9.17667442	5.37134298	-0.00364543	0.00033922	-0.00000127	0.61510000
O	9.60882202	2.63768112	3.71479608	0.00642175	0.00136067	-0.01771866	-1.19890000
O	9.10281031	5.87836912	4.46132231	0.01143494	0.01437050	-0.02045347	-1.19360000
H	8.92910973	1.86919299	3.64477151	-0.00721672	-0.00724946	-0.00390018	0.63020000
H	10.26600189	2.45355687	2.99937315	0.00925084	-0.01145104	0.01149200	0.57740000
H	9.08911097	4.77348945	5.54382596	0.01274901	0.01526300	0.01641613	0.64520000
H	9.78017806	6.53563744	4.15022214	-0.00163474	0.00527853	-0.00148735	0.59170000
O	2.63557760	6.71116431	8.85653903	0.01188034	-0.01020888	-0.00082273	-1.25700000
H	3.01622586	7.06766408	7.98055458	0.00610717	-0.00565004	-0.00233959	0.61380000
H	1.81387276	7.33738571	8.99198834	0.00500028	-0.00680543	0.00145506	0.62350000
O	4.16176399	4.87611998	10.34573108	-0.00101854	0.00214657	0.01878983	-1.22290000
H	3.47310691	5.37821057	9.81088037	0.00221576	0.00126881	0.00768019	0.62540000
H	4.05561133	3.90951556	10.09831939	0.00265832	0.00677252	0.01497117	0.60850000
H	5.35960072	6.33660240	6.53576522	0.00337025	-0.01681968	-0.01454773	0.06340000
H	6.72966864	4.67517860	8.41787509	-0.01714886	0.00189675	-0.01209472	0.62420000
H	5.29041771	4.87543489	9.15925918	0.00984094	0.00205238	-0.00997512	0.60440000
H	6.86662069	5.39941381	6.48780259	0.00077781	0.00704547	-0.00851612	0.08630000

*CH_3O;*OH (direct CO_2 adsorption pathway)

119

Lattice="9.84 0.0 0.0 0.0 8.52169 0.0 0.0 0.0 11.75" Properties=species:S:1:pos:R:3:forces:R:3

C	1.26675428	0.64133363	0.76678795	-0.00511317	-0.00275463	0.00067635
C	1.27449763	2.06413642	0.72173766	0.00414145	-0.00749426	-0.00121182
C	2.50128089	2.77763955	0.91657764	0.01340228	-0.00036568	0.00623064
C	2.50430706	4.20179976	0.97171980	0.01473944	0.00328491	0.01361813
C	1.28646336	4.91050300	0.76068083	-0.00101841	0.00741750	0.00088343
C	1.28019792	6.32681996	0.83678519	0.00266523	0.00282659	-0.00027467
C	2.51141765	7.02711670	1.03582488	0.00457956	-0.00882607	-0.00025442
C	2.49500402	8.45561265	0.97598960	0.00198620	-0.00430082	0.00217226
C	3.71866893	0.64970221	1.14828367	0.00891252	0.00432933	0.01444829
C	3.71883764	2.06764147	1.12625701	0.00324469	0.00319554	-0.00829457
C	4.92007272	2.77237255	1.47388802	0.00975057	-0.00124686	-0.03466130
C	4.78888074	4.13364291	1.85704586	0.01284033	0.00527657	0.00318761
C	3.67376292	4.89283564	1.42391763	0.00537302	0.01524389	-0.00121390
C	3.71013092	6.33235089	1.39275395	0.01370370	-0.00658649	0.01162753
C	4.94109061	7.06790091	1.55333284	-0.00936565	-0.00804770	0.00991633
C	4.94395159	8.46636650	1.33135419	0.00765136	-0.00862473	0.01998908
C	6.18781138	0.66650042	1.26452391	-0.00131588	-0.00235984	0.00397612
C	6.20246815	2.07472741	1.38516673	0.00510346	0.00517793	-0.00250418
C	7.46670047	2.76611107	1.24302163	-0.01514415	0.00669568	0.00263612
C	7.63500306	4.12467584	1.63474865	-0.01129753	-0.00373885	0.00450017
C	6.21890127	6.47737344	1.88464968	-0.00128738	-0.00960310	0.00700304
C	7.42546931	7.05547070	1.31202456	-0.00208889	-0.00366604	-0.00253644
C	7.41300741	8.46870709	1.07995781	-0.00698961	0.00016341	-0.01204998
C	8.62946314	0.64055182	0.81039277	-0.00610365	-0.01144402	0.00502218
C	8.63477041	2.06264707	0.81049344	-0.00644476	0.00784974	0.01021596
C	9.87791517	2.77098351	0.66811078	-0.01095650	0.00194337	-0.01126237
C	9.89169315	4.19343248	0.69665603	-0.01127481	0.00301985	-0.02113294
C	8.68822087	4.88875046	1.05637794	-0.01422454	0.00483702	-0.02366367
C	8.65047218	6.32776114	1.04331832	-0.01004307	-0.00147458	-0.03078709
C	9.87205379	7.03729332	0.82750838	-0.00871245	-0.00400768	0.00881305
C	9.86804727	8.45800285	0.75501720	-0.00283420	-0.00117079	0.00347457
Cu	7.02227554	4.05803413	5.01263185	-0.00380290	-0.01246906	0.02292544
Cu	5.02653919	5.34345375	4.82233786	-0.00307413	0.02339749	0.03294803
Cu	7.41632760	6.84821757	3.51870881	0.01815251	0.07213009	0.02099811
Cu	4.80346433	3.21090251	3.82511685	0.01401240	0.02275226	0.02313171
Cu	6.27824490	4.82156889	2.81594950	0.00598398	0.00589410	0.05605923
Cu	8.71007681	4.65759706	3.35650843	-0.00633833	0.00743015	0.08910498
O	5.29601280	6.95065850	5.82603434	-0.02854983	-0.01888188	-0.00160674
O	5.66080564	5.10051033	7.99628809	0.00181604	0.00338447	0.00397165
C	6.33993690	7.77308560	5.35695273	0.01064997	-0.00152187	-0.01354855
H	3.51998236	3.31877023	7.94606800	0.00311919	0.00314261	-0.01622184
O	3.38236337	4.08868736	7.32272332	0.00834590	-0.00107292	0.00497415

H	4.30647827	4.53984222	7.41517344	0.00471016	0.00940298	-0.01491381
H	1.80253141	8.40566671	2.84861169	-0.00397173	0.00233091	0.01437676
O	1.57109038	8.11969166	3.76782481	-0.00294497	0.00972566	-0.01937285
H	2.12752350	7.26599702	3.95336216	0.01111000	-0.00270003	-0.00621459
H	2.27158244	2.45836945	5.14904505	0.00962661	0.02020806	-0.01137799
O	2.80216463	3.26901534	4.86021471	-0.00665017	-0.01881667	-0.01347945
H	3.10080678	3.60113547	5.77510245	-0.00415654	-0.00643373	-0.00162039
H	7.51524912	3.10762428	9.46482549	-0.00608242	-0.00527955	0.00509370
O	7.02063177	2.28630851	9.81113975	-0.00119542	-0.01035349	0.01148441
H	7.13229541	2.34733656	10.79267723	-0.00687275	0.00119148	-0.00537925
H	4.31905987	-0.97243190	6.38440178	-0.00093155	-0.00979277	-0.00963685
O	3.57252469	-0.44791821	7.07827468	0.00062359	0.00198962	-0.00000546
H	4.26244430	-0.06197551	7.68998557	-0.00183904	0.00222793	-0.01100063
H	0.74759601	6.14018956	6.77220099	0.00192540	0.00418011	-0.00171836
O	1.15209875	5.25994208	6.45347884	0.00333604	0.00371932	-0.00660364
H	1.84382811	4.95076427	7.11662966	0.00175194	0.01048498	-0.00462995
H	5.15856277	2.12471141	7.59588704	0.00921768	0.00455139	-0.02113436
O	5.59264641	2.07183718	6.67543279	-0.00790761	-0.00354364	0.01257927
H	5.55613574	3.01031215	6.32200128	0.00024824	-0.00578965	0.02273883
H	8.74633421	8.91027508	6.80083514	-0.00935567	-0.00003165	-0.00414373
O	8.36078858	9.84948554	6.67667015	-0.00884315	-0.00413619	0.00104006
H	7.45134308	9.84470971	7.09217598	0.00142183	0.00658152	-0.00344458
H	3.62131050	9.42294936	4.26694841	0.00904168	-0.02016974	-0.00714199
O	4.57360793	9.68632837	4.30582930	-0.00537266	-0.00002791	-0.01758988
H	4.75441881	9.83262087	5.29334165	-0.00053097	-0.01467746	0.00767220
H	5.55580847	2.34871230	9.46877288	-0.00415489	-0.00471342	0.00008371
O	4.54219180	2.35004953	9.16873429	0.00072222	-0.00614079	0.00714786
H	4.20297631	1.44442109	9.48688339	0.00408681	0.00028214	0.00616531
H	7.54681229	6.98426731	10.04385610	0.00045233	0.00341361	0.00969255
O	8.51724573	6.80681514	9.79443561	-0.00364563	0.00379105	0.00909446
H	8.90925423	6.61132215	10.68788383	-0.00361376	-0.00061369	0.00276316
H	-0.21726013	-0.45366905	9.34295495	-0.00256098	0.00382474	0.00078051
O	0.67942788	0.01068445	9.31644194	0.00378543	0.00347484	0.01289462
H	0.65126206	0.55008946	8.49080298	0.00369607	-0.00092228	0.00080718
H	9.45433154	7.15073139	5.65500819	-0.01038618	-0.01047900	-0.01950358
O	9.71418610	7.56359506	6.58876282	-0.00450057	-0.00669276	0.00465558
H	10.44671561	8.23385747	6.37048169	-0.00320782	-0.00660082	-0.01133083
H	6.15621684	-0.77121263	9.04066801	-0.00315732	0.00624346	-0.00804981
O	6.21491911	-0.38200998	8.12647658	0.00137542	-0.00558612	0.01564068
H	6.02665484	-1.19650566	7.57578584	0.00431841	-0.00344033	0.00863951
H	8.84467976	2.87833566	6.41917400	0.01573111	-0.00235148	-0.00464596
O	8.69029414	3.87426317	6.26653885	-0.01284481	0.00184369	-0.00933341
H	8.13073712	1.22810060	5.02200284	-0.00742525	0.01056427	-0.02365320
O	8.13692029	1.18148309	4.00065162	-0.00304110	-0.01266280	-0.01771483
H	7.32923072	1.69678398	3.73177658	-0.01389284	0.00437396	-0.00106131

H	2.13154575	5.69745832	5.29408064	0.01261265	0.00231587	-0.01087537
O	2.75729749	5.94292272	4.49795809	0.00724945	0.00978852	-0.01265350
H	2.58119128	5.16060904	3.91192730	-0.01618777	-0.00375482	-0.02023086
H	8.48921500	4.30424393	7.85837151	-0.01002943	0.00685969	-0.01192888
O	8.10333379	4.43280082	8.78356596	-0.00388617	0.00383687	0.00434152
H	8.37348581	5.35470402	9.13298165	-0.00142817	0.00172162	-0.00316851
H	5.20083207	7.61569948	10.59048788	0.00469485	-0.00227599	0.00022460
O	5.96970782	6.94842659	10.57226800	-0.00575654	-0.00511151	0.01824699
H	5.47809474	6.07434864	10.57380075	0.00354971	0.00127362	0.01503525
H	3.31594277	7.72889176	9.69074417	0.00226659	-0.00113248	0.00917601
O	3.86952526	8.50306974	10.11806772	0.00303079	-0.00303541	0.01107069
H	3.33821250	8.73573357	10.92006791	0.00079577	-0.00450119	-0.01257827
H	2.52331018	9.01467120	6.43369237	0.00496440	-0.00078504	-0.00160605
O	1.71899458	9.50178437	5.98807954	-0.00390446	0.00458735	-0.00161481
H	1.65861474	9.05112482	5.07710313	-0.00624479	0.00038616	0.00037566
O	9.95792639	3.00627644	3.73946200	0.01408134	0.00628500	-0.01278444
O	9.23001408	6.43304636	4.30498889	0.00347907	0.00060917	-0.02273174
H	9.28368081	2.22040442	3.74824384	0.00086251	-0.01891096	-0.00208815
H	10.56986430	2.81187210	2.98860345	0.01276938	-0.01157640	0.01321056
H	9.59719667	4.31856485	6.11074849	-0.00854327	-0.00133803	-0.02900747
H	9.89834315	6.97416964	3.79613351	0.01867607	0.00868910	-0.00427750
O	2.48830443	6.63522232	8.97953733	0.00415758	-0.01225285	0.00079975
H	2.90673486	7.01917534	8.11485755	0.00579213	-0.00610610	-0.00536558
H	1.67336382	7.25631199	9.08604915	0.00564167	-0.00768660	0.00407125
O	4.20511312	4.87295005	10.23216184	-0.00078215	0.00200000	0.02078662
H	3.41625650	5.32719801	9.80430541	0.00044241	0.00008399	0.00817800
H	4.14791410	3.89378618	10.00343204	0.00284974	0.00674515	0.01506019
H	6.60573073	4.78164582	8.20301061	-0.01683421	0.00258533	-0.01967049
H	5.22176558	5.09512737	8.90619097	0.00853260	0.00531967	-0.01364856
H	6.20339413	8.03849441	4.25714683	0.00139462	-0.00906243	-0.01219668
H	7.33518380	7.32060123	5.59852096	-0.00134250	-0.01704967	0.00329294
H	6.32980052	8.76503192	5.84856608	0.00480701	-0.00069204	-0.02737248

CH_3OH;*OH (direct CO_2 adsorption pathway)

120

Lattice="9.84 0.0 0.0 0.0 8.52169 0.0 0.0 0.0 11.75" Properties=species:S:1:pos:R:3:forces:R:3

C	1.39731409	0.68037476	0.77042020	-0.00728112	-0.00111329	0.00218834
C	1.41239550	2.10384004	0.67781112	0.00432397	-0.00536333	0.00087910
C	2.64069227	2.82019202	0.85613878	0.01079618	-0.00067130	0.00583573
C	2.64453203	4.24488788	0.91384543	0.00327503	0.00256453	0.00787268
C	1.42006434	4.95109649	0.73590489	-0.00059787	0.00589856	0.00228911
C	1.41126186	6.36507736	0.84606145	0.00246282	0.00208067	0.00056593
C	2.63701512	7.06892672	1.06939711	0.00339088	-0.01035374	0.00192846
C	2.61940228	8.49957217	1.01411293	-0.00056141	-0.00293146	0.00771504
C	3.84656984	0.69324607	1.13864877	0.00732826	0.00490575	0.01390361

C	3.85414545	2.10805994	1.06548025	-0.00774848	-0.00294286	-0.01049358
C	5.06445694	2.81565910	1.36785637	-0.00104152	0.00604342	-0.03929691
C	4.96128128	4.18093328	1.70524701	0.00491437	0.04340139	-0.05263174
C	3.82472903	4.93555722	1.35166102	0.00537478	0.00702444	-0.00099083
C	3.84353550	6.37820842	1.39650952	0.00642786	0.00137677	0.00622949
C	5.07491862	7.12246148	1.55740068	-0.00787809	-0.00690796	0.01272761
C	5.07438954	8.51275994	1.29730575	0.00654971	-0.01032486	0.01830836
C	6.31821057	0.70140250	1.18516094	0.00253422	-0.01085883	0.00005766
C	6.34047017	2.10866450	1.29810482	0.01362135	-0.00196430	-0.01563089
C	7.60216370	2.79746966	1.14986400	-0.00506944	0.00610404	-0.00764811
C	7.76283749	4.13905681	1.58950366	0.00017158	-0.00043739	0.00027211
C	6.35172911	6.53279824	1.87774800	0.00245813	-0.00736192	0.01073150
C	7.54844964	7.10169490	1.28626846	-0.00346352	-0.00492966	-0.00358982
C	7.53912035	8.50439536	1.00388503	-0.00563738	-0.00238600	-0.01342952
C	8.76106576	0.67207066	0.74451694	-0.00541962	-0.01118328	0.00491206
C	8.76789301	2.09698631	0.71857662	-0.00874695	0.00727280	0.00266963
C	10.01388123	2.81032664	0.59924060	-0.00809317	0.00233956	-0.01109465
C	10.02370429	4.23554915	0.65042331	-0.01029701	0.00489880	-0.02229660
C	8.81707909	4.92208855	1.03156336	-0.01249398	0.00241992	-0.02461141
C	8.77987174	6.36399287	1.03441567	-0.00814621	-0.00194926	-0.03280347
C	10.00316676	7.07246154	0.82770344	-0.00662010	-0.00314301	0.00807290
C	10.00089259	8.49404124	0.74299467	-0.00175652	-0.00211858	0.00343721
Cu	7.08559384	4.31911230	5.01958227	0.00743849	-0.00786954	0.01089212
Cu	4.84858937	5.11727168	4.62116050	-0.03643558	-0.04392371	0.03429849
Cu	7.58535018	6.76852160	3.48077872	0.02726240	0.05582634	0.03016414
Cu	5.48125678	2.93166378	3.73397641	0.01786056	-0.00911041	0.11130033
Cu	6.39249461	4.84246760	2.75680589	0.01259007	0.00798188	0.06939628
Cu	8.82709923	4.63728217	3.33522816	0.00974643	0.01612952	0.09761395
O	6.94510158	6.78155858	6.33616267	0.02645520	-0.00380406	0.02710615
O	5.40136544	5.10198869	7.64930042	-0.00809523	0.00952800	0.01119052
C	6.32104014	7.69800354	5.42438409	0.01262056	0.01814682	-0.01194971
H	3.33160581	3.03860118	7.88249471	-0.00069238	0.00318178	-0.01874303
O	3.11182513	3.72025385	7.19030346	0.01493075	-0.00402012	-0.00468333
H	3.98726068	4.23038143	7.16727478	0.00876624	0.00817526	-0.01728938
H	2.02844956	8.51281832	3.02703530	-0.00284434	0.00583872	0.01360871
O	1.70943603	8.14989520	3.89311301	0.00156216	0.00810856	-0.01736809
H	2.27904947	7.32985335	4.03964759	0.01057943	-0.00360024	-0.00803453
H	1.59415478	2.96869016	4.25571821	0.00113810	0.00740255	-0.00477113
O	2.57434662	3.14917563	4.50933169	-0.01602259	-0.00845362	-0.01053377
H	2.62471171	3.21322144	5.51467406	0.01172865	0.00241200	-0.01199557
H	7.43207104	3.13594976	9.43837458	-0.00763032	-0.00377834	0.00309545
O	7.05637641	2.25895646	9.78908461	0.00065222	-0.01248660	0.01023099
H	7.24040719	2.28488381	10.76222154	-0.00654694	-0.00007670	-0.00626821
H	3.99270019	-1.39181207	6.31655569	-0.00161854	-0.00428605	0.00393689
O	3.74428089	-0.81448443	7.07807765	0.00280347	-0.00955111	-0.01075274

H	4.66651607	-0.56575131	7.57195963	-0.00253721	-0.00099308	0.00406358
H	0.65119809	6.18536512	6.80499514	-0.00248743	0.00555357	-0.00315665
O	1.10336973	5.30320378	6.49888037	0.00428315	0.00550798	-0.00609130
H	1.67905722	4.91677144	7.22190332	0.00252521	0.00889012	-0.00607732
H	5.21492322	2.18798876	7.66079104	0.00993366	0.00189570	-0.01737223
O	5.68078355	2.09547466	6.75446965	-0.00202733	0.00415920	0.01327255
H	5.68591970	3.00735389	6.33335744	-0.00407529	-0.00571872	0.02375481
H	8.74704588	8.88643103	6.80621242	-0.01264049	-0.00376404	-0.00569895
O	8.38038071	9.84267841	6.68339301	-0.01173986	-0.00308709	0.00146313
H	7.43712693	9.91090283	7.02073332	-0.00184777	0.00403068	-0.00299546
H	3.58086040	10.38616751	4.42490336	-0.00393398	-0.00597892	-0.00182494
O	4.45720721	9.85917032	4.45427980	0.00715173	-0.01296832	-0.01553768
H	4.74847356	9.97548782	5.41809070	-0.00750362	-0.00796876	0.01192578
H	5.59410369	2.32321211	9.51556026	-0.00421378	-0.00428020	0.00158272
O	4.58309805	2.36917273	9.18810953	0.00159627	-0.00675287	0.00927842
H	4.20934442	1.46530388	9.47119681	0.00526314	-0.00372666	0.00394478
H	7.50105486	6.97369396	10.13788581	0.00143608	0.00394279	0.00960301
O	8.43488986	6.77358391	9.79139376	-0.00198376	0.00420247	0.00626922
H	8.89890944	6.52433935	10.63787609	-0.00263773	0.00005308	0.00221169
H	-0.28508353	-0.43575612	9.32005128	-0.00326495	0.00463833	-0.00078155
O	0.61483635	0.01393762	9.28475467	0.00222418	0.00340204	0.01317847
H	0.60562571	0.53467431	8.44626819	0.00182691	0.00031332	0.00742760
H	9.43671284	7.16121667	5.67838879	-0.01611375	-0.01218090	-0.01589976
O	9.68018226	7.55291714	6.63302179	-0.01050053	-0.01011502	0.00441839
H	10.48131104	8.17769334	6.46795786	-0.00344431	-0.00899838	-0.01097207
H	5.89229901	-0.79254715	9.08783310	-0.00091940	0.00425534	-0.00836001
O	5.92653452	-0.23753453	8.23966603	0.00345446	0.00312223	0.01477745
H	6.57427591	-0.76484286	7.67219691	0.00397935	-0.00551742	0.00746164
H	8.85981893	2.90891379	6.45778684	0.00846697	0.00268704	-0.01038747
O	8.71452659	3.90053988	6.29584757	-0.01347506	0.00150001	-0.01131166
H	8.24108932	1.31796530	5.11084600	-0.01099068	0.00891998	-0.02046478
O	8.22490078	1.34176258	4.07853336	0.00380182	-0.01390083	-0.01700394
H	7.45537669	1.97178699	3.88820057	0.01285142	-0.00802155	-0.01247725
H	2.20880185	5.68882469	5.47402467	0.00315359	0.00045711	-0.00723129
O	2.87737419	5.79731199	4.67564721	0.00797756	0.00392058	-0.01374246
H	2.66980078	4.90103869	4.23300128	-0.01643214	0.00234364	-0.01898558
H	8.23623776	4.33821174	7.76583049	-0.01241910	0.00856407	-0.01723813
O	7.72766667	4.51218842	8.63213277	-0.01075738	0.00494155	-0.00114483
H	8.08947329	5.37633606	9.04629678	-0.00398828	0.00319184	-0.00970895
H	5.17912728	7.59938909	10.74863843	0.00649700	-0.00364031	-0.00379347
O	5.87733667	6.92082222	10.49354473	-0.00496031	-0.00385597	0.01886071
H	5.34593685	6.06302024	10.45194059	0.00347222	0.00198612	0.01456716
H	3.22794875	7.77102523	9.69596464	0.00244516	0.00045513	0.00613557
O	3.76560676	8.54681898	10.09466312	-0.00255121	-0.00291674	0.00659426
H	3.22130837	8.79712980	10.88389278	0.00298464	-0.00514682	-0.01238068

H	2.59100180	8.83563341	6.63386882	0.00550489	0.00387589	-0.00083924
O	1.76620231	9.32541257	6.30804800	0.00643155	0.00922281	-0.00785006
H	1.79918306	9.10413786	5.32096108	-0.00532405	0.00312611	0.00124182
O	10.07182521	2.91957353	3.55100134	-0.00149823	0.00616693	-0.01272085
O	9.34069183	6.46757810	4.32757360	0.00359392	0.00151184	-0.01978656
H	9.31599181	2.18455290	3.77964758	-0.00529615	-0.01807981	-0.00300790
H	10.32356249	2.70659729	2.60951679	0.01228589	-0.01963889	0.01072678
H	9.62136793	4.37996130	6.23067501	-0.01167779	0.00003162	-0.03158990
H	10.02134295	7.00963674	3.83535898	0.01647895	0.00343737	-0.00364601
O	2.34316478	6.58778081	9.00733080	0.00139141	-0.00770587	0.00135584
H	2.83046998	6.88464337	8.16061276	0.00225650	-0.00237509	-0.00302078
H	1.56493330	7.26481671	9.07845988	0.00587589	-0.00703328	0.00327255
O	4.19963370	4.90957447	9.96432612	0.00098848	0.00198866	0.02040353
H	3.30306623	5.31941109	9.76196733	0.00141090	-0.00036044	0.00961740
H	4.15425129	3.90186393	9.85479963	0.00307861	0.00645054	0.01333937
H	6.22040600	6.18738893	6.74099276	0.00810660	0.00178328	0.00196224
H	6.31971869	4.77768845	8.01184798	-0.01154441	0.00080705	-0.01858012
H	4.89515727	5.19565334	8.54200433	0.00886843	0.00722773	-0.01689264
H	5.48444727	7.23776743	4.83841125	-0.01828331	0.01437450	-0.00970993
H	7.09338402	8.09748015	4.70862641	-0.01165411	-0.00940214	-0.01012567
H	5.94333037	8.59814740	5.94907489	0.00210125	0.00002932	-0.03082594

*HC(OH)$_2$;*OH (direct CO_2 adsorption pathway)

117

Lattice="9.84 0.0 0.0 0.0 8.52169 0.0 0.0 0.0 11.75" Properties=species:S:1:pos:R:3:forces:R:3

C	1.21728486	0.70416316	0.76534341	-0.00208018	-0.00312934	-0.00515482
C	1.22294785	2.13144329	0.71627646	0.00201583	-0.00669337	-0.00280488
C	2.44856375	2.84314672	0.85049459	0.00322556	-0.00042311	-0.00032627
C	2.45357455	4.26636009	0.91964194	0.01298832	0.00548612	0.00579853
C	1.23086296	4.97770555	0.77924870	0.00374906	0.00593949	0.00121314
C	1.22800812	6.39245848	0.85988374	0.00327909	0.00561771	0.00093979
C	2.46320376	7.09298674	1.00585320	0.00319945	-0.00213726	0.00171508
C	2.44613448	8.51751678	0.93182483	0.00393007	-0.00576038	0.00132203
C	3.67042004	0.71415845	1.02166558	0.00143142	-0.00420342	0.01359090
C	3.67231694	2.13118995	0.99651017	0.00789722	0.00309841	-0.01131651
C	4.87076575	2.82541365	1.34042709	0.02208119	-0.00769670	-0.05091446
C	4.72473129	4.15754817	1.82035956	0.01376823	0.00060232	0.00912791
C	3.62469738	4.95030117	1.36020855	0.00087552	0.01511859	-0.01858865
C	3.67191963	6.39027450	1.31488865	0.00853228	-0.00309774	-0.01058638
C	4.90093693	7.14205960	1.42111453	-0.00986850	-0.00404621	-0.01900150
C	4.89984538	8.53394640	1.15756103	0.00955984	-0.01160051	0.01525423
C	6.13830079	0.73449711	1.10441441	0.00049873	-0.00399034	0.00169685
C	6.14591916	2.14255959	1.26563454	0.00640345	0.00605641	0.00432790
C	7.41322041	2.83030953	1.23773712	-0.01662496	-0.00414745	0.00521038
C	7.59206781	4.16688218	1.72568753	-0.01235515	-0.00078070	0.01275915

C	6.17049323	6.56888046	1.79553086	0.00295043	-0.01175064	0.00778394
C	7.37673955	7.13758608	1.26577368	0.00600847	-0.00530299	-0.05261482
C	7.36529328	8.53577365	0.97812038	-0.00384323	-0.00292331	-0.01425411
C	8.58472475	0.71055164	0.77598296	-0.00231772	-0.00661871	0.00044395
C	8.58714852	2.13312563	0.81362122	-0.00960874	0.00445998	0.00378934
C	9.82874752	2.84165096	0.71158402	-0.00716969	-0.00265184	-0.00956967
C	9.83778227	4.26504585	0.77654075	-0.01141114	0.00510273	-0.01804459
C	8.64872044	4.95553785	1.17099754	-0.01128481	0.01590037	-0.05525410
C	8.60595272	6.39380454	1.12203760	-0.00696914	0.00275440	-0.01617145
C	9.82308364	7.09982765	0.86792686	-0.00258746	0.00344933	0.01109253
C	9.82059595	8.52155096	0.76426862	-0.00570647	-0.00176389	-0.00276596
Cu	6.39484559	4.52341553	5.03883362	-0.01121401	-0.07577059	-0.00795353
Cu	4.70473679	6.10002132	4.18298960	0.02154813	0.03249331	0.03746569
Cu	7.03695328	6.94990116	3.67089876	-0.04587082	0.03323709	0.05567994
Cu	4.51077094	3.53745149	3.75110301	0.02789862	0.00691983	0.07331386
Cu	6.18746715	4.93197478	2.71425707	0.00623524	0.00046496	0.07164568
Cu	8.39838765	4.54549628	3.63907683	-0.01575254	-0.00788765	0.11722091
C	6.13216973	5.68685387	6.56853086	0.00144579	-0.00761957	0.01255176
O	5.83155867	5.16867295	7.84665618	-0.00587133	0.00588285	0.01211951
O	7.25944930	6.57347840	6.67211936	-0.00535932	0.00349792	0.01034176
H	3.62935454	3.32107944	8.06319164	0.00274722	0.00314165	-0.01335285
O	3.47231043	4.10668772	7.45161338	0.01322470	0.00678457	-0.00415788
H	4.36272845	4.59112626	7.61238306	0.00714449	0.00549171	-0.01845720
H	1.01514339	8.54396857	3.03669783	-0.00528619	-0.00161565	0.01169108
O	0.95116725	8.16453395	3.94787552	-0.00571205	0.00545651	-0.01601407
H	1.68470949	7.46380595	4.00157425	0.00743395	-0.00160315	-0.00923648
H	2.45399682	2.43142227	5.25487164	0.00615596	0.01176160	-0.00747203
O	2.95009620	3.30370778	4.96060975	0.00849530	0.00524317	-0.01577560
H	3.26370120	3.63064714	5.86942144	0.00601990	0.00687337	0.00346215
H	7.61167271	2.97361009	9.43925371	-0.00594438	-0.00592473	0.00341478
O	7.10771908	2.17221142	9.81229756	0.00038552	-0.01443889	0.01011765
H	7.13766795	2.33272720	10.79058154	-0.00641029	0.00216670	-0.00779449
H	3.83197433	-0.95135215	6.02213743	0.00461880	0.00677764	0.01601687
O	3.65702856	-0.39069825	6.81974637	-0.00392518	-0.00281580	-0.00942091
H	4.65251970	-0.29403562	7.30783656	0.00599040	0.00132725	-0.00507082
H	1.06733309	6.20305475	6.92311774	0.00119907	0.00330566	-0.00826774
O	1.47922133	5.39796485	6.46860507	-0.00150860	0.00563462	-0.00137166
H	2.16780770	4.97919831	7.09616180	0.00076579	0.01090969	-0.00939855
H	5.27542182	2.13101867	7.55733186	0.00269561	0.01037414	-0.01590401
O	5.75578070	2.10058606	6.64273027	-0.00296124	0.00183697	0.00328438
H	5.78971103	3.05911177	6.32393178	-0.00358313	0.00217519	-0.00773348
H	8.73211091	9.03844592	6.90678862	-0.00981604	0.00307674	-0.00466424
O	8.29535533	9.81122616	6.44534868	-0.00338860	-0.00897456	0.00214600
H	7.36487188	9.98274394	6.81483463	-0.00259508	0.00139467	-0.00374316
H	4.45441844	8.97923201	4.15005260	0.00155652	-0.00586537	-0.00932593

O	5.05894049	9.75128651	4.28402862	-0.00650370	-0.00370394	-0.01137902
H	5.13142716	9.91447329	5.29446974	0.00414380	-0.01078802	0.00020392
H	5.67687467	2.32123879	9.39024558	-0.00475449	-0.00381009	-0.00042150
O	4.68439909	2.37117570	9.03198246	0.00109096	-0.00602525	0.00619248
H	4.30276820	1.47360543	9.35569161	0.00447845	-0.00241689	0.00339934
H	7.66111003	6.93217266	9.98394379	0.00434106	0.00371101	0.00462203
O	8.65498335	6.79341689	9.73874595	-0.00299013	0.00264344	0.01301275
H	9.07169156	6.61342732	10.62239083	-0.00220117	-0.00066123	-0.00043324
H	-0.05149425	-0.23849625	9.50181196	-0.00085907	0.00656834	0.00898947
O	0.83910939	0.21055928	9.40708276	0.00102987	0.00124795	0.01733034
H	0.80673668	0.60421660	8.49926441	0.00465306	0.00109371	-0.00058558
H	9.14480939	7.06222345	7.67205866	0.00162875	-0.00520011	-0.01088186
O	9.76544916	7.49441002	7.02463026	0.00297241	-0.00667332	-0.00533223
H	9.46543734	7.09477930	6.12822583	-0.00224903	-0.00597173	-0.01339281
H	5.89572541	-0.78959694	8.75938063	0.00047990	0.00172763	-0.01083228
O	5.87382866	-0.19853192	7.92494358	0.00805851	-0.00431782	0.00211010
H	6.51721334	-0.70165320	7.32067716	0.00236890	-0.00020616	-0.00689596
H	9.00948374	2.88072946	6.32053803	-0.00753372	0.01426593	-0.02034812
O	8.94958049	3.88584765	6.34268961	0.00176578	0.00740952	-0.00283932
H	7.87696044	0.64624227	5.02356586	-0.00411134	0.00006468	-0.00685965
O	7.60064947	0.44155496	4.05512801	0.00160898	-0.01366669	-0.01744778
H	6.66592295	0.84378961	4.00605150	-0.00173587	0.03497851	-0.00636889
H	2.18257957	5.84637044	5.38621072	0.00883493	-0.00367848	-0.00716425
O	2.68400947	6.21867205	4.46476296	0.00688938	0.00287978	-0.01483860
H	2.48233777	5.48330525	3.82644109	-0.02095802	-0.01235056	-0.01002767
H	8.54793128	4.22187098	7.77125124	-0.01160740	0.01206052	-0.01449171
O	8.09512869	4.36031010	8.70231401	-0.00573904	0.00751848	0.00064049
H	8.43160332	5.22775393	9.10252318	-0.00355362	0.00085313	-0.00659652
H	5.50289663	7.64023855	10.57534492	0.00735193	-0.00116620	0.00101683
O	6.09032385	6.94244631	10.16590418	-0.00223666	0.00070936	0.01761671
H	5.48356225	6.10040169	10.16132570	0.00327971	0.00322317	0.00779497
H	3.44769558	7.85332876	9.57409031	0.00214433	-0.00057290	0.00614246
O	3.97963632	8.61513251	10.01943573	-0.00458474	-0.00077510	0.01204655
H	3.43073535	8.84525487	10.80952184	-0.00112499	-0.00703582	-0.01280304
H	2.48078920	9.24245679	6.36429350	0.00328976	-0.00470826	0.00242290
O	1.73533602	9.85227345	6.04723413	-0.00686804	-0.00880602	0.00356376
H	1.28644529	9.30961470	5.33036711	-0.00556629	-0.00231050	0.00307836
O	9.54585613	2.63200839	3.75867964	-0.00162171	-0.00294790	-0.01170399
O	8.92088673	6.27560030	4.70001955	0.01539754	0.00202342	-0.01821620
H	8.85046776	1.90661848	3.71635553	-0.00489053	-0.00644839	-0.00854673
H	10.00022967	2.55371651	2.88015242	0.00976086	-0.01299151	0.01331087
H	9.85774571	4.25943332	6.15735397	-0.01586876	0.00127451	-0.02367670
H	9.46671885	6.95308175	4.19753711	0.01532536	0.00073087	-0.00001235
O	2.56339949	6.78436244	8.83010639	0.00611563	-0.01015754	-0.00000832
H	2.92629766	7.18615255	7.96277642	0.00561002	-0.00478818	0.00062186

H	1.78292963	7.41292550	9.04533288	0.00592556	-0.00790214	0.00313772
O	4.32523931	5.02856562	10.25068306	0.00050202	-0.00129714	0.01117203
H	3.60625272	5.43348339	9.68937847	0.00384325	0.00131197	0.00730702
H	4.45093600	4.10473020	9.89443039	0.00519960	0.00654184	0.01498898
H	5.22878717	6.31384796	6.31959585	-0.01642102	0.01059170	0.00329801
H	6.72349904	4.76702387	8.22762334	-0.01382218	-0.00073477	-0.01076806
H	7.86540912	6.44695888	5.84317632	0.00878202	0.00010342	0.01479764

$CH_2(OH)_2$;*OH (direct CO_2 adsorption pathway)

118

Lattice="9.84 0.0 0.0 0.0 8.52169 0.0 0.0 0.0 11.75" Properties=species:S:1:pos:R:3:forces:R:3

C	8.65167185	4.96280904	1.16346038	-0.01030425	0.01113933	-0.05429918
C	7.59353792	4.18481146	1.71718505	-0.01260630	0.00071221	0.00989676
C	7.41954820	2.84181874	1.24915827	-0.01759369	-0.00432229	0.00689145
C	8.59560771	2.14279258	0.83445861	-0.00970627	0.00529383	0.00429459
C	9.83411077	2.85021844	0.71349059	-0.00806575	-0.00178481	-0.00921563
C	9.83938326	4.27252069	0.76441757	-0.01170840	0.00496695	-0.01892853
C	6.15478854	2.15087090	1.25912074	0.00633873	0.00587462	0.00310893
C	6.14661648	0.74331737	1.09197117	-0.00385762	0.00247376	0.00565651
Cu	6.15524101	4.96711329	2.67468364	0.00655296	-0.00397217	0.07860464
Cu	8.36820668	4.69930090	3.64124363	-0.01491925	-0.01517454	0.12202937
O	8.92760069	6.32969815	4.79832700	0.01568450	-0.00237591	-0.01638165
Cu	6.83725583	6.98016850	3.72875930	-0.06327226	0.01453047	0.06614251
Cu	4.55236218	6.07152053	4.13223776	0.02162369	0.02622660	0.03873582
O	2.52941284	6.19226484	4.37147609	0.00766057	0.00355461	-0.01454866
C	8.59370461	0.72030096	0.79502186	-0.00026751	-0.00333474	0.00062594
C	8.61512914	6.40310088	1.11355511	-0.00663657	0.00241789	-0.01536721
C	9.82765763	7.10815795	0.87030748	0.00158991	0.00227846	0.01013651
C	9.82656669	8.53222399	0.77510412	-0.00514235	-0.00116892	-0.00262385
C	4.87806569	2.83673363	1.29860871	0.02393529	-0.00831490	-0.05394830
C	3.67635103	2.13777750	0.96698634	0.00941513	0.00306242	-0.01228282
C	2.45427760	2.85133309	0.81440442	0.00348757	-0.00114820	-0.00345386
C	2.45270655	4.27367743	0.87478388	0.01178073	0.00567899	0.00341143
C	3.63006476	4.96207112	1.28737140	0.00209875	0.01303326	-0.01642652
C	4.73757891	4.18334844	1.71086455	0.01453377	0.00152337	0.00391577
C	3.67079350	6.40250760	1.26648639	0.00874023	-0.00274599	-0.01093050
C	2.46339160	7.10366710	0.99036581	0.00340026	-0.00133841	0.00254891
C	1.23040984	6.40123981	0.84692591	0.00333249	0.00537364	0.00015653
C	1.23025404	4.98661723	0.75676190	0.00332869	0.00567264	-0.00021894
C	1.22714900	2.13859467	0.70673922	0.00195293	-0.00637880	-0.00332458
C	1.22187711	0.71443029	0.76904386	-0.00235186	-0.00304780	-0.00445148
C	2.45007728	8.52888720	0.92640450	0.00420556	-0.00625033	0.00258589
C	3.67450441	0.72279575	1.00916419	0.00395016	-0.00196056	0.01513414
Cu	4.45634870	3.50735492	3.64974107	0.02616736	0.00736277	0.08922461
O	2.89980551	3.30432992	4.89820326	0.00614869	0.00342433	-0.01397397

C	4.90868094	7.14406719	1.34225577	-0.01250452	-0.00457757	-0.02073236
C	6.16562319	6.55775027	1.68840757	0.00203582	-0.01371102	-0.00139527
C	7.37962058	7.13974821	1.22973242	0.00953786	-0.00607265	-0.05371022
C	7.37243372	8.54395889	0.97249416	-0.00190343	-0.00300132	-0.01237458
C	4.90755908	8.54398475	1.12165417	0.00698438	-0.00883450	0.01197529
Cu	6.26831817	4.46938383	4.89421792	-0.00628867	-0.04730782	0.00015356
C	6.41714013	5.62155925	6.80754148	0.00536052	-0.00662676	0.01143343
O	7.34613259	6.68825642	6.75909605	-0.00609752	0.01365336	0.01995594
O	9.52728036	2.73564280	3.77923584	-0.00126264	-0.00291956	-0.01034509
O	5.92641138	5.34697791	8.07887974	-0.00952815	0.00240870	0.00357151
O	1.51130407	5.37238387	6.48593174	-0.00066484	0.00432548	-0.00094571
O	8.12741136	4.30511103	8.85750568	-0.00482011	0.00574717	0.00338487
O	9.04246669	3.87529876	6.50028621	0.00663424	0.00460919	-0.00323457
O	3.59196344	4.18635896	7.42477999	0.01633088	0.00839438	-0.00711104
O	4.68347798	2.41926707	9.02572534	0.00212233	-0.00453753	0.00626140
O	7.09595402	2.10318132	9.83443263	0.00034916	-0.01599979	0.01087561
O	5.80178566	2.07298364	6.62359075	-0.00246392	0.00630755	0.00281237
O	5.79610573	-0.15955592	7.97570738	0.00974042	-0.00646381	0.00148225
O	3.64001715	-0.47929905	6.77055313	-0.00077034	-0.00687594	-0.00915298
O	0.90429774	8.24888056	3.94806786	-0.00600976	0.00569331	-0.01645474
O	9.88729263	7.48812373	7.04399808	0.00427511	-0.00691424	-0.00586718
O	8.65984263	6.78462755	9.75664136	-0.00397738	0.00302556	0.01292336
O	6.07980045	7.00180014	10.20824455	-0.00271203	0.00375507	0.02296724
O	4.29331094	5.05902694	10.30384760	0.00197951	-0.00029230	0.00859117
O	3.92178625	8.69729463	9.99946823	-0.00775207	-0.00087569	0.01294019
O	2.55643832	6.78677263	8.84569923	0.00529199	-0.00948804	0.00355210
O	8.35531252	9.77779855	6.41476462	-0.00312023	-0.00834476	0.00056259
O	1.74586684	9.85452215	6.04078640	-0.00527466	-0.00940389	0.00557832
O	7.51185241	0.41334282	4.04366478	0.00142138	-0.01465486	-0.01751694
O	4.99530464	9.72065649	4.26088722	-0.01031330	0.00075116	-0.01087437
O	0.82694573	0.22236686	9.41887734	0.00082342	0.00136381	0.01833430
H	3.75875119	3.39632630	8.03964286	0.00442903	0.00479288	-0.01486194
H	4.40349553	4.73730848	7.68245116	0.00548447	0.00448662	-0.01892451
H	0.96258385	8.60872061	3.02794218	-0.00499469	-0.00167753	0.01094075
H	1.58450415	7.49368140	3.98269670	0.00731753	-0.00255149	-0.00868387
H	2.42481536	2.43269131	5.24664459	0.00755475	0.01296689	-0.00873168
H	3.26794192	3.66800444	5.76161080	0.00874626	0.00879845	0.00414100
H	7.62229036	2.90927860	9.51077219	-0.00557028	-0.00601310	0.00386414
H	7.10355472	2.22793610	10.81780685	-0.00701468	0.00190317	-0.01056627
H	3.89356440	-1.03147494	5.98311469	0.00421496	0.00372955	0.01377003
H	4.59366871	-0.32129813	7.30205700	0.00670863	-0.00265170	-0.00751254
H	1.11689966	6.18596001	6.94683051	-0.00032625	0.00346435	-0.00961456
H	2.24301679	4.97923934	7.08238422	0.00158185	0.01059150	-0.00968996
H	5.30879386	2.14147198	7.52392001	0.00320389	0.01309265	-0.01639839
H	5.69704844	2.96910153	6.17343744	-0.00564641	-0.00099146	-0.00444371

H	8.81024066	9.02090857	6.88518752	-0.01052384	0.00189300	-0.00513043
H	7.44439270	9.95943704	6.81160107	-0.00368643	0.00059936	-0.00213949
H	4.50552263	8.86477205	4.13590882	0.00254258	-0.00183141	-0.01116273
H	5.09217658	9.85428879	5.26640154	0.00251624	-0.00531275	-0.00467309
H	5.67027797	2.32481420	9.38734787	-0.00402123	-0.00268826	0.00036167
H	4.27037140	1.52644512	9.33921149	0.00463390	-0.00282891	0.00450685
H	7.66816150	6.93936678	9.98742431	0.00359568	0.00411836	0.00444019
H	9.06591902	6.65450053	10.65574860	-0.00332732	-0.00086882	-0.00152767
H	-0.06277207	-0.22551684	9.51719769	-0.00117463	0.00689923	0.00940340
H	0.79283168	0.61298553	8.50971086	0.00492020	0.00263749	0.00126794
H	9.24194889	7.07921887	7.68433965	0.00325684	-0.00822608	-0.00889223
H	9.56365290	7.09636665	6.14877337	-0.00063066	-0.00552909	-0.01287822
H	5.82713789	-0.74091305	8.81536745	0.00204996	0.00195976	-0.00969747
H	6.46686379	-0.62863248	7.38456530	-0.00035730	-0.00230209	-0.00734939
H	9.09739663	2.87919824	6.37987490	-0.00598840	0.01374309	-0.01914913
H	7.84288933	0.62714907	4.98885834	-0.00266215	0.00158679	-0.00601772
H	6.59399644	0.85889541	4.01003451	0.00033082	0.03665729	-0.00770631
H	2.10946942	5.82632000	5.32538886	0.00053747	-0.00371070	-0.00491762
H	2.29163687	5.44416602	3.76246811	-0.02118558	-0.01745741	-0.01281799
H	8.59521869	4.15064690	7.93494700	-0.00625243	0.01027672	-0.00881470
H	8.49595300	5.16569296	9.24750044	-0.00239678	0.00135750	-0.00449195
H	5.55547580	7.68901965	10.70111891	0.00755265	-0.00342445	-0.00319750
H	5.46657720	6.17301643	10.22738083	0.00109493	0.00611758	0.01620332
H	3.43350131	7.91036176	9.55907844	0.00338620	-0.00163732	0.00814618
H	3.34860757	8.90995588	10.77584793	0.00100250	-0.00868135	-0.01250508
H	2.48415382	9.22390465	6.32966901	0.00256864	-0.00464537	0.00182816
H	1.28825842	9.33361191	5.30922789	-0.00437816	-0.00251048	0.00302395
H	8.82567494	2.02447189	3.72691145	-0.00528328	0.00094328	-0.00830897
H	9.97597731	2.65148794	2.89729330	0.00787727	-0.00865905	0.01502612
H	9.94374690	4.27566576	6.31649710	-0.01484524	0.00184828	-0.02452479
H	9.35418487	7.05981352	4.25777805	0.01418756	0.00911059	0.00012152
H	2.90506238	7.15343071	7.95758455	0.00609672	-0.00324047	0.00118732
H	1.77641337	7.41684809	9.05391348	0.00596864	-0.00822752	0.00366700
H	3.57582149	5.47499192	9.74629645	0.00357043	0.00207681	0.00819889
H	4.40196885	4.13242021	9.94975400	0.00505468	0.00696714	0.01565096
H	5.51700771	5.86751347	6.16646129	-0.00286183	0.00366020	-0.00711936
H	6.72585235	4.85540259	8.53942741	-0.01389861	0.00258170	-0.00608644
H	7.93282510	6.56723077	5.87090388	-0.00339037	-0.01454684	0.00682335
H	7.01343548	4.69036096	6.44894839	-0.00848808	-0.00306106	-0.03440217

Structures for investigating low-coordination sites

Cu_6

9

Lattice="20.4792934 0.0 0.0 0.0 21.4450873 0.0 0.0 0.0 21.688688499999998" Properties=species:S:1:pos:R:3

Cu	11.33362670	9.91127170	11.38402770
Cu	9.64791420	10.09955540	9.70461480
Cu	11.38569180	11.70717120	9.80598870
Cu	9.71076380	11.87588750	8.06466210
Cu	11.45938710	13.44508730	8.18298240
Cu	8.00000000	10.26019120	8.00000000
H	12.47929340	8.00000000	12.67536270
O	12.19819900	8.89215780	12.92547880
H	11.61812520	8.75701810	13.68868850

Cu_6 (a)

9

Lattice="19.805719578 0.0 0.0 0.0 21.857855724 0.0 0.0 0.0 18.987002938" Properties=species:S:1:pos:R:3

Cu	9.43294666	12.72829138	10.13351034
Cu	11.49277673	12.40070998	8.99739387
Cu	10.46772633	10.50887662	10.16851273
Cu	9.38820209	11.51138447	8.17462016
Cu	8.00000000	10.81350599	9.97560406
Cu	9.71910186	13.85785572	8.00000000
H	11.80571958	8.31732997	10.59798414
O	10.99478235	8.67478935	10.98700294
H	10.32498089	8.00000000	10.80589925

Cu_6 (b)

9

Lattice="20.619914379 0.0 0.0 0.0 19.690794957999998 0.0 0.0 0.0 20.373699367" Properties=species:S:1:pos:R:3

Cu	9.13581859	9.56389858	9.89394427
Cu	10.55707331	10.24833413	8.09992483
Cu	10.39647461	11.69079496	9.99462003
Cu	12.61991438	9.02521102	8.00000000
Cu	12.47914750	11.31649875	8.84008107
Cu	10.50293842	8.00000000	8.67079776
H	9.06017153	10.93019624	12.37369937
O	8.82760184	11.10200123	11.44765985
H	8.00000000	11.60726957	11.47444727

Cu(111) surface

30

Lattice="7.65796644025031 0.0 0.0 3.828983220125155 6.6319934785854535 0.0 0.0 0.0 21.968468943549098" Properties=species:S:1:pos:R:3:tags:I:1:forces:R:3

Cu	1.23660153	0.74265532	7.96044825	3	-0.00000765	0.00110009	0.11540190
Cu	3.78747625	0.74647292	7.94395401	3	0.00012951	-0.00052465	0.11947958
Cu	6.34262209	0.74451857	7.95424619	3	0.00094803	0.00048742	0.11683886
Cu	2.51462936	2.95536186	7.95445871	3	-0.00064998	0.00143344	0.11757325
Cu	5.06092013	2.95509907	7.95011954	3	0.00124028	0.00131394	0.11889599

Cu	7.61895433	2.95529302	7.95614258	3	0.00143384	-0.00074771	0.11617535
Cu	3.78713882	5.16326738	7.95823747	3	0.00027645	0.00066565	0.11582697
Cu	6.33878600	5.16822576	7.95164754	3	-0.00008002	0.00072842	0.11717589
Cu	8.89728338	5.16573221	7.94867634	3	0.00175194	0.00019568	0.11696107
Cu	-0.02254619	1.49020969	10.02380322	2	-0.00024162	-0.00197014	0.00477550
Cu	2.53078496	1.49387788	10.01215634	2	-0.00450552	0.00102781	0.00064568
Cu	5.07868205	1.49611815	9.99983922	2	0.00362849	-0.00306640	0.00737946
Cu	1.25158545	3.70936690	10.00505118	2	0.00135354	-0.00233366	0.00256417
Cu	3.80361355	3.70369633	10.01723673	2	-0.00109690	0.00516442	0.00014197
Cu	6.35890897	3.71039514	10.00287029	2	-0.00004383	-0.00054657	0.00445642
Cu	2.53508088	5.92061985	10.02005417	2	0.00042993	0.00119569	0.00765649
Cu	5.07226617	5.92076199	10.03030763	2	-0.00118210	0.00071289	0.00353787
Cu	7.62794642	5.91254695	9.99109956	2	0.00099648	0.00088450	0.00753330
Cu	-0.00112964	0.04502292	12.10195835	1	0.01202171	0.00283452	-0.14756338
Cu	2.55061099	0.03474971	12.06807001	1	-0.00471888	-0.00231409	-0.10277291
Cu	5.06391580	0.03312602	12.02868335	1	-0.01414616	-0.00206340	-0.13000752
Cu	1.28154798	2.26247923	12.05909158	1	-0.00320795	-0.00335907	-0.10659016
Cu	3.81883421	2.24500636	12.08403385	1	0.00291794	-0.00360448	-0.10893635
Cu	6.35624146	2.26691176	12.04747162	1	-0.00202339	-0.00235186	-0.10946287
Cu	2.53778938	4.45526541	12.07110936	1	0.00009136	0.01062169	-0.09856477
Cu	5.09720499	4.45102634	12.06828366	1	-0.00620113	0.00610496	-0.10213345
Cu	7.65020723	4.45834102	12.07360084	1	0.00437467	0.00233683	-0.10583409
H	0.16391214	-0.40039049	14.74314502	0	0.00277855	-0.00409440	-0.03868298
O	-0.40965825	0.30429475	14.34689726	0	0.00878783	-0.00349301	-0.02575223
H	-1.29775355	-0.13884311	14.27865373	0	-0.00505541	-0.00633853	-0.01671901

Cu(100)

30

Lattice="7.65796644025031 0.0 0.0 3.828983220125155 6.6319934785854535 0.0 0.0 0.0 21.968468943549098" Properties=species:S:1:pos:R:3:tags:I:1:forces:R:3

Cu	1.23660153	0.74265532	7.96044825	3	-0.00000765	0.00110009	0.11540190
Cu	3.78747625	0.74647292	7.94395401	3	0.00012951	-0.00052465	0.11947958
Cu	6.34262209	0.74451857	7.95424619	3	0.00094803	0.00048742	0.11683886
Cu	2.51462936	2.95536186	7.95445871	3	-0.00064998	0.00143344	0.11757325
Cu	5.06092013	2.95509907	7.95011954	3	0.00124028	0.00131394	0.11889599
Cu	7.61895433	2.95529302	7.95614258	3	0.00143384	-0.00074771	0.11617535
Cu	3.78713882	5.16326738	7.95823747	3	0.00027645	0.00066565	0.11582697
Cu	6.33878600	5.16822576	7.95164754	3	-0.00008002	0.00072842	0.11717589
Cu	8.89728338	5.16573221	7.94867634	3	0.00175194	0.00019568	0.11696107
Cu	-0.02254619	1.49020969	10.02380322	2	-0.00024162	-0.00197014	0.00477550
Cu	2.53078496	1.49387788	10.01215634	2	-0.00450552	0.00102781	0.00064568
Cu	5.07868205	1.49611815	9.99983922	2	0.00362849	-0.00306640	0.00737946
Cu	1.25158545	3.70936690	10.00505118	2	0.00135354	-0.00233366	0.00256417
Cu	3.80361355	3.70369633	10.01723673	2	-0.00109690	0.00516442	0.00014197
Cu	6.35890897	3.71039514	10.00287029	2	-0.00004383	-0.00054657	0.00445642

Cu	2.53508088	5.92061985	10.02005417	2	0.00042993	0.00119569	0.00765649
Cu	5.07226617	5.92076199	10.03030763	2	-0.00118210	0.00071289	0.00353787
Cu	7.62794642	5.91254695	9.99109956	2	0.00099648	0.00088450	0.00753330
Cu	-0.00112964	0.04502292	12.10195835	1	0.01202171	0.00283452	-0.14756338
Cu	2.55061099	0.03474971	12.06807001	1	-0.00471888	-0.00231409	-0.10277291
Cu	5.06391580	0.03312602	12.02868335	1	-0.01414616	-0.00206340	-0.13000752
Cu	1.28154798	2.26247923	12.05909158	1	-0.00320795	-0.00335907	-0.10659016
Cu	3.81883421	2.24500636	12.08403385	1	0.00291794	-0.00360448	-0.10893635
Cu	6.35624146	2.26691176	12.04747162	1	-0.00202339	-0.00235186	-0.10946287
Cu	2.53778938	4.45526541	12.07110936	1	0.00009136	0.01062169	-0.09856477
Cu	5.09720499	4.45102634	12.06828366	1	-0.00620113	0.00610496	-0.10213345
Cu	7.65020723	4.45834102	12.07360084	1	0.00437467	0.00233683	-0.10583409
H	0.16391214	-0.40039049	14.74314502	0	0.00277855	-0.00409440	-0.03868298
O	-0.40965825	0.30429475	14.34689726	0	0.00878783	-0.00349301	-0.02575223
H	-1.29775355	-0.13884311	14.27865373	0	-0.00505541	-0.00633853	-0.01671901

Cu(211)

30

Lattice="7.65796644025031 0.0 0.0 3.828983220125155 6.6319934785854535 0.0 0.0 0.0 21.968468943549098" Properties=species:S:1:pos:R:3:tags:I:1:forces:R:3

Cu	1.23660153	0.74265532	7.96044825	3	-0.00000765	0.00110009	0.11540190
Cu	3.78747625	0.74647292	7.94395401	3	0.00012951	-0.00052465	0.11947958
Cu	6.34262209	0.74451857	7.95424619	3	0.00094803	0.00048742	0.11683886
Cu	2.51462936	2.95536186	7.95445871	3	-0.00064998	0.00143344	0.11757325
Cu	5.06092013	2.95509907	7.95011954	3	0.00124028	0.00131394	0.11889599
Cu	7.61895433	2.95529302	7.95614258	3	0.00143384	-0.00074771	0.11617535
Cu	3.78713882	5.16326738	7.95823747	3	0.00027645	0.00066565	0.11582697
Cu	6.33878600	5.16822576	7.95164754	3	-0.00008002	0.00072842	0.11717589
Cu	8.89728338	5.16573221	7.94867634	3	0.00175194	0.00019568	0.11696107
Cu	-0.02254619	1.49020969	10.02380322	2	-0.00024162	-0.00197014	0.00477550
Cu	2.53078496	1.49387788	10.01215634	2	-0.00450552	0.00102781	0.00064568
Cu	5.07868205	1.49611815	9.99983922	2	0.00362849	-0.00306640	0.00737946
Cu	1.25158545	3.70936690	10.00505118	2	0.00135354	-0.00233366	0.00256417
Cu	3.80361355	3.70369633	10.01723673	2	-0.00109690	0.00516442	0.00014197
Cu	6.35890897	3.71039514	10.00287029	2	-0.00004383	-0.00054657	0.00445642
Cu	2.53508088	5.92061985	10.02005417	2	0.00042993	0.00119569	0.00765649
Cu	5.07226617	5.92076199	10.03030763	2	-0.00118210	0.00071289	0.00353787
Cu	7.62794642	5.91254695	9.99109956	2	0.00099648	0.00088450	0.00753330
Cu	-0.00112964	0.04502292	12.10195835	1	0.01202171	0.00283452	-0.14756338
Cu	2.55061099	0.03474971	12.06807001	1	-0.00471888	-0.00231409	-0.10277291
Cu	5.06391580	0.03312602	12.02868335	1	-0.01414616	-0.00206340	-0.13000752
Cu	1.28154798	2.26247923	12.05909158	1	-0.00320795	-0.00335907	-0.10659016
Cu	3.81883421	2.24500636	12.08403385	1	0.00291794	-0.00360448	-0.10893635
Cu	6.35624146	2.26691176	12.04747162	1	-0.00202339	-0.00235186	-0.10946287
Cu	2.53778938	4.45526541	12.07110936	1	0.00009136	0.01062169	-0.09856477

Cu	5.09720499	4.45102634	12.06828366	1	-0.00620113	0.00610496	-0.10213345
Cu	7.65020723	4.45834102	12.07360084	1	0.00437467	0.00233683	-0.10583409
H	0.16391214	-0.40039049	14.74314502	0	0.00277855	-0.00409440	-0.03868298
O	-0.40965825	0.30429475	14.34689726	0	0.00878783	-0.00349301	-0.02575223
H	-1.29775355	-0.13884311	14.27865373	0	-0.00505541	-0.00633853	-0.01671901

Cu_{20}

23

Lattice="22.372573646 0.0 0.0 0.0 23.661354253 0.0 0.0 0.0 25.67727445" Properties=species:S:1:pos:R:3:forces:R:3

Cu	8.79393109	11.80430995	10.48389474	-0.01615472	-0.01056855	0.01330979
Cu	12.80480183	14.30222799	11.86044622	-0.00531409	0.00920623	0.00670264
Cu	10.74141132	13.14934941	11.14725740	-0.00583583	0.00105666	-0.00163588
Cu	12.20397450	11.76480629	9.41768602	0.00405486	0.00429495	0.00209494
Cu	14.17881086	12.99816856	10.21618676	0.01195826	-0.00764729	-0.00495753
Cu	10.73409250	10.49136070	11.22968322	0.00115913	0.00506276	0.00412522
Cu	11.46506138	13.14129617	13.49613937	0.00939924	0.01731640	-0.00334402
Cu	10.18183608	13.00012936	8.75182050	-0.00200029	0.01198222	0.00656018
Cu	14.09770847	15.38439132	10.16481029	0.01076268	0.01278371	-0.00727612
Cu	9.44580913	11.87571537	12.82534646	-0.00316536	0.00522180	-0.00321737
Cu	12.90480652	11.84178081	11.98316833	0.00397849	0.00432793	-0.00788581
Cu	12.13992467	14.23085821	9.43384014	-0.01179563	0.00270743	-0.00641452
Cu	11.45464423	10.64294288	13.57719089	0.01476035	0.00563123	0.00126040
Cu	12.79628221	9.38029236	12.01295652	-0.00497948	-0.00184440	0.01178991
Cu	10.16205113	11.95887792	15.12717893	0.01251637	0.01886967	0.02429600
Cu	14.18345258	10.58894560	10.29497548	0.00563217	0.00627487	-0.00216515
Cu	12.14340209	9.31094326	9.58651167	-0.01381012	-0.00332190	-0.01020488
Cu	10.17705911	10.49455199	8.83362291	0.00384473	-0.00715841	0.00320306
Cu	14.10652380	8.20761595	10.39143957	-0.00687945	-0.02132121	0.00006610
Cu	8.25639222	11.73240922	8.14625203	-0.01317221	-0.00652343	0.00005075
O	10.22622440	12.00402387	17.23297296	0.02882688	0.01066990	-0.00749653
H	10.87994389	11.31391389	17.49336887	-0.01556727	0.03965093	0.02027361
H	9.41537437	11.77436325	17.74029354	-0.03480961	-0.02271636	-0.01893153

Cu_{20} (a)

23

Lattice="22.380743814 0.0 0.0 0.0 23.382528273 0.0 0.0 0.0 23.150124264" Properties=species:S:1:pos:R:3:forces:R:3

Cu	8.64172367	12.02324394	10.40299750	0.00432830	-0.00456984	-0.01739598
Cu	12.71875244	13.71143961	12.59857650	0.01624135	0.00901498	-0.01104021
Cu	10.59410760	13.02859679	11.51110757	0.00685544	-0.00380449	-0.00340410
Cu	12.10103914	12.01048665	9.55358537	0.00172819	-0.00314855	-0.00327313
Cu	14.13309435	12.79569554	10.73247112	-0.00288044	0.01292581	-0.01343468
Cu	10.46420374	10.44218011	10.85400921	0.00731021	-0.00148967	-0.00207786
Cu	11.22726138	12.26467033	13.79614540	-0.01758679	-0.00739801	0.00402591
Cu	10.23322058	13.55089980	9.13629563	-0.00116620	-0.01049762	-0.01828726
Cu	14.20471129	15.10882239	11.36486970	0.00696443	-0.01015455	-0.01966724

Cu	9.14939604	11.39972010	12.68897910	-0.00266113	-0.01079855	-0.00030633
Cu	12.63965168	11.32179315	12.01900864	-0.01089303	0.00783788	0.00277543
Cu	12.23738822	14.36290484	10.22498719	0.01248818	0.00941882	-0.00572757
Cu	11.05234865	9.83705127	13.18160655	-0.01291458	-0.00323518	0.00350100
Cu	12.36949325	8.95996993	11.36256265	0.01413993	-0.00243127	-0.00619459
Cu	9.72843287	10.80227339	14.93350649	-0.01362157	-0.00853852	-0.01158889
Cu	13.94743846	10.47081527	10.10745474	-0.00370242	-0.01802191	0.00996766
Cu	11.86538498	9.61559302	8.99080823	0.01010909	-0.01359063	0.00685621
Cu	10.04396427	11.12304970	8.52061788	0.00151032	-0.00087200	-0.00321926
Cu	13.69364532	8.15951011	9.54349819	-0.00102874	0.00838535	0.00007393
Cu	8.25198358	12.66686660	8.13311837	0.00988703	-0.01574688	-0.00830230
O	9.36815189	15.00383319	12.40891140	0.03171407	-0.01197758	-0.01769007
H	10.28035567	15.37335001	12.46002179	0.02667061	0.02536265	0.03008265
H	9.34094148	14.40053163	13.19050151	0.00199380	0.04926420	0.00634528

Cu_{20} (b)

23

Lattice="23.973969331 0.0 0.0 0.0 23.460647651000002 0.0 0.0 0.0 22.843847656999998" Properties=species:S:1:pos:R:3:forces:R:3

Cu	10.20529892	11.28909016	10.78849713	-0.02900072	0.00041889	0.01223708
Cu	14.10065276	14.30844835	10.34416874	0.00198115	-0.00246379	0.00586346
Cu	12.12674381	12.84414979	10.55238988	-0.01428813	-0.00206704	-0.00716067
Cu	13.14302301	11.32463668	8.62887861	0.00380497	-0.00087791	-0.00857200
Cu	15.06919481	12.85944678	8.52931291	0.00330217	-0.00137732	0.00323435
Cu	12.51117392	10.30032173	10.99777590	-0.01110924	0.00301323	-0.00870237
Cu	13.57163085	13.35589361	12.49859701	-0.00217320	0.00640137	-0.00707190
Cu	10.85796500	12.24137540	8.54474605	0.01028825	0.00403441	-0.00881284
Cu	14.63052787	15.18529389	8.18444764	0.00596558	-0.01062261	0.00390118
Cu	11.63012961	11.79532183	12.71680555	-0.00901106	0.00460403	-0.01175137
Cu	14.60377366	11.93210499	10.76593232	-0.00073823	-0.00339830	0.00143275
Cu	12.72370276	13.74359256	8.32809126	0.00913294	-0.00443814	-0.00847097
Cu	13.96067372	10.90587780	12.91645467	-0.00192380	-0.00148777	-0.00519803
Cu	14.85512849	9.51394115	11.16716273	-0.00318154	0.00025245	0.00780112
Cu	13.01187486	12.33002617	14.59067538	-0.00479630	-0.00008067	-0.02205052
Cu	15.44705640	10.50912880	8.93841302	0.00018353	0.00410234	-0.00366364
Cu	13.48590427	8.93934116	9.16524127	-0.00648016	0.00441479	-0.01303761
Cu	11.23562087	9.79888810	8.99218454	0.00721090	0.00464555	-0.01425003
Cu	15.75860032	8.18765843	9.39418136	-0.02428784	0.00951508	0.00302598
Cu	9.05606257	10.69561806	8.71083517	0.01954277	0.01226550	0.00433197
O	8.26295265	11.14549806	11.98102338	0.00398801	-0.00999344	0.03628984
H	7.95223163	10.25084194	11.71312632	-0.01430617	0.00290015	0.04745890
H	8.72674072	10.98632099	12.83589109	0.00088381	-0.01199717	0.04320230

Cu_{38}

41

Lattice="25.377528597 0.0 0.0 0.0 24.190014341999998 0.0 0.0 0.0 24.089874281" Properties=species:S:1:pos:R:3:forces:R:3

Cu	9.97742680	10.44981967	13.63112407	0.00551758	0.01649002	-0.01040445
Cu	9.35605481	11.84663462	11.64161395	-0.02098335	0.00280828	-0.01303291
Cu	10.58469016	12.53587480	14.91916627	-0.01617032	0.00324415	0.01450640
Cu	9.88777697	13.89809501	12.95376409	-0.00894146	-0.01304190	0.00264066
Cu	11.74264291	8.69477985	13.35305991	-0.00742229	0.00459392	0.00477246
Cu	12.77456279	8.28778268	11.12352053	-0.00467775	0.00307208	0.01028134
Cu	13.98764896	8.97223169	14.38257561	-0.00249021	0.00290974	-0.00620546
Cu	10.38975871	11.44734201	9.44865914	-0.00150371	0.00738033	-0.00178768
Cu	12.10367811	9.65859445	9.18830691	-0.00015966	0.00507955	0.00892790
Cu	10.97515867	9.98251003	11.37949705	-0.00768512	0.00492017	-0.00741517
Cu	11.57391227	12.10339561	12.69702700	0.00194704	0.00580735	-0.00394296
Cu	13.31815873	10.34103348	12.41740043	0.00060482	0.00527865	-0.00382277
Cu	12.62984920	11.73578183	10.44977087	0.00742896	0.00384281	0.00062520
Cu	12.22257628	10.68998005	14.74080223	0.00087497	0.00372704	0.00473689
Cu	12.81532251	12.81900580	15.91200834	-0.01218243	-0.00584831	-0.00357729
Cu	14.51246841	11.01926343	15.64310195	0.00301544	0.00534112	-0.00267354
Cu	13.84735092	12.42457974	13.70492863	0.00121427	-0.00527501	-0.00828774
Cu	11.96362843	13.13582908	8.51344173	0.00480196	-0.00007277	0.00010805
Cu	10.82222338	13.57728387	10.67278749	-0.00390030	-0.00598701	0.00096641
Cu	11.43267581	15.57794486	12.01648590	-0.00043130	-0.01388346	-0.00120734
Cu	13.15176052	13.80902419	11.74526349	-0.00092941	-0.01170720	-0.00204064
Cu	12.49333529	15.18186182	9.79147322	0.00272177	-0.00418667	0.00127865
Cu	12.08834767	14.28899946	14.03668711	-0.00741962	-0.01271943	-0.00210405
Cu	14.38504367	14.50441751	14.95070807	-0.00972276	-0.01341533	0.00263695
Cu	13.70508796	15.86747644	13.00803144	-0.00675056	-0.01445036	0.00062626
Cu	15.02973660	8.57776450	12.14910751	0.00871140	-0.00319671	-0.00489930
Cu	13.67896510	11.34280321	8.24622350	0.00340611	-0.00105510	-0.00533077
Cu	14.39408847	9.87097214	10.11746835	0.00434612	0.00215896	0.00102833
Cu	14.88800635	12.02599135	11.47015335	0.01301774	0.00462396	-0.00173203
Cu	16.58975252	10.23999807	11.21201216	0.00928449	-0.00200455	-0.00110717
Cu	15.91597957	11.62717293	9.25328918	0.00162844	-0.00165081	-0.00627615
Cu	15.65182020	10.58261086	13.48399238	0.00374305	-0.00227847	-0.00318268
Cu	16.09149015	12.70472233	14.70021866	0.00746091	-0.00188770	-0.00300369
Cu	17.12598066	12.31333396	12.49148616	0.01387051	-0.00338884	0.00193900
Cu	14.25136481	13.46054371	9.41362653	0.00522598	-0.00032356	-0.00255984
Cu	14.75697543	15.44996756	10.79256772	0.00353218	-0.00873174	0.00712378
Cu	16.46977520	13.69481733	10.52451516	0.01470773	0.00461090	-0.00366827
Cu	15.51754080	14.17608876	12.77285441	-0.00008372	-0.00755176	0.00460080
O	8.21886899	9.59495675	14.56645011	0.01095947	0.03552248	0.03266320
H	8.53431534	8.83006606	15.10123523	0.00975093	0.00917354	0.01458290
H	7.93190485	10.25498640	15.24024381	0.00753689	-0.03811218	-0.01121061

Cu_{38} (a)

41

Lattice="24.22356561 0.0 0.0 0.0 24.301338491 0.0 0.0 0.0 23.992099623" Properties=species:S:1:pos:R:3:forces:R:3

Cu	8.80835810	10.60479440	13.32200589	0.00152373	0.00107976	-0.00212650
Cu	8.31934074	11.86821997	11.19817589	0.00206476	-0.00167185	0.00621399
Cu	9.22028350	12.74589969	14.50562081	-0.00159574	0.00059510	-0.00006732
Cu	8.72768358	14.00285182	12.40807151	0.00003628	0.00062334	0.00021591
Cu	10.55194444	8.82177160	13.32489521	-0.00215152	0.00704137	0.00151408
Cu	11.81044267	8.30410381	11.20471724	-0.00115620	0.00609980	0.00993396
Cu	12.69715240	9.19150795	14.51215762	0.00232153	0.00409620	-0.00051244
Cu	9.56373741	11.34523899	9.13053097	0.00236241	-0.00381010	0.00916598
Cu	11.31457508	9.55893761	9.13429505	-0.00038539	-0.00225353	0.00782126
Cu	9.95626057	9.97964451	11.20125823	-0.01840093	-0.01852785	-0.00420045
Cu	10.44465546	12.20962668	12.41046321	0.00074957	-0.00175345	0.00080394
Cu	12.18986112	10.42516188	12.41403719	-0.00364543	0.00298348	0.00237432
Cu	11.70161774	11.69185764	10.31752822	-0.00214057	-0.00230395	0.00643834
Cu	10.89875144	10.90991016	14.61311746	-0.00125087	0.00298511	0.00438800
Cu	11.35181200	13.10086899	15.68895833	0.00106648	0.00440793	0.00092562
Cu	13.09266805	11.31731992	15.69502593	0.00297729	0.00436669	-0.00253185
Cu	12.60601890	12.58277629	13.61674700	0.00087196	0.00131468	-0.00024055
Cu	11.22845605	12.97507831	8.24656634	0.00005997	-0.00241668	0.00427596
Cu	9.88257347	13.53828614	10.25397328	-0.00124101	-0.00110024	0.00229017
Cu	10.37173691	15.61251198	11.52188932	0.00211902	-0.00092435	0.00045360
Cu	12.11501156	13.84830198	11.51828688	-0.00003434	-0.00075751	0.00248889
Cu	11.63520839	15.09965515	9.43658303	0.00073180	-0.00573946	0.00017458
Cu	10.81620014	14.44609666	13.66783327	-0.00107956	0.00121683	0.00227229
Cu	13.00727487	14.72697704	14.79001091	0.00656814	-0.00132784	-0.00236549
Cu	12.52679088	15.97599002	12.71667067	0.00290013	-0.00305447	0.00347724
Cu	13.94835975	8.67191570	12.41642986	0.00301425	0.00641075	0.00337977
Cu	12.97268710	11.19186724	8.24676476	-0.00134010	-0.00072751	0.00758822
Cu	13.50976263	9.83472525	10.25972420	0.00070837	-0.00224930	0.00229855
Cu	13.86094578	12.06323118	11.52412516	-0.00175218	-0.00118735	0.00068436
Cu	15.58982516	10.28676829	11.53269719	0.00170156	0.00400386	-0.00055224
Cu	15.09980565	11.55486841	9.44293731	0.00142973	0.00072719	0.00174712
Cu	14.42802797	10.75278349	13.67707887	0.00678977	0.00391696	0.00160258
Cu	14.74862430	12.94226952	14.79958037	0.00554668	0.00487931	-0.00390536
Cu	15.99153137	12.43217360	12.73298453	0.00203434	0.00435453	-0.00225184
Cu	13.41839828	13.37514454	9.33616266	0.00266453	-0.00048324	0.00100800
Cu	13.78994112	15.45820454	10.63864722	-0.00121876	-0.00079781	0.00015253
Cu	15.50735564	13.69760732	10.64519088	0.00064293	0.00110490	0.00030449
Cu	14.36124492	14.30331325	12.75629047	0.00319090	0.00018810	-0.00441904
O	8.28853151	8.33320908	10.59549090	-0.01354640	-0.03426856	-0.02051899
H	8.95672773	7.95125512	9.97559671	-0.03536983	-0.01209615	-0.00348499
H	7.86985653	9.04115582	10.04883095	-0.02624351	-0.00823094	-0.02351653

Cu_{55}

58

Lattice="28.52 0.0 0.0 0.0 26.57 0.0 0.0 0.0 28.76" Properties=species:S:1:pos:R:3:forces:R:3

Cu	-0.27791622	-0.02442955	0.44051620	0.00050098	-0.00000947	0.00068035
Cu	1.99129918	-0.02725302	-0.31181090	-0.17423546	-0.00058098	0.05667412
Cu	1.42161451	-0.04396066	2.15010836	-0.12292351	0.00237532	-0.12286209
Cu	-1.96384649	-0.01724415	-1.26583655	0.12879279	-0.00061047	0.13092822
Cu	-2.56038667	-0.01718992	1.17724104	0.17462887	-0.00077500	-0.05696720
Cu	-1.50230739	2.01758274	0.14552597	0.09324446	-0.15660583	0.02272699
Cu	0.94215205	2.00711823	0.73898747	-0.09451139	-0.15545773	-0.02404669
Cu	-1.51491864	-2.05651903	0.13136286	0.09499988	0.15570923	0.02312807
Cu	0.93063640	-2.06799959	0.71714540	-0.09336147	0.15641607	-0.02151495
Cu	-0.76908539	-1.28807682	2.40375760	0.03635372	0.09679625	-0.15156066
Cu	-0.75766977	1.23015768	2.41499383	0.03503749	-0.09552720	-0.15252008
Cu	0.19193673	-1.27961205	-1.54787507	-0.03656939	0.09619260	0.15225879
Cu	0.19898904	1.24106428	-1.53569424	-0.03689434	-0.09716462	0.15164746
Cu	4.23520055	-0.02962910	-1.04153074	-0.16454802	0.00020180	0.05489679
Cu	3.76703335	-0.03878685	1.40934762	-0.11389826	0.00194059	-0.01937837
Cu	3.09089451	-0.06811021	3.82954314	-0.14040664	-0.00709210	-0.15261256
Cu	-3.62257960	-0.00553418	-2.94201225	0.12223432	-0.00069160	0.12386630
Cu	-4.33945250	-0.01910056	-0.54632080	0.11475280	-0.00051923	0.02807975
Cu	-4.80526157	-0.01363163	1.91001064	0.16463796	-0.00046811	-0.05391733
Cu	-2.70383693	4.02839595	-0.13984471	0.08807713	-0.14772953	0.02096571
Cu	-0.27225787	4.15042173	0.45006313	-0.00031698	-0.11765749	-0.00059576
Cu	2.15676020	4.00565788	1.05057018	-0.08866561	-0.14794660	-0.02111429
Cu	-2.72708413	-4.06006260	-0.16552940	0.08926302	0.14720946	0.02196295
Cu	-0.30003475	-4.20022912	0.42109153	0.00070014	0.11809095	0.00049006
Cu	2.12939349	-4.08137061	1.00051804	-0.08779590	0.14839472	-0.02048499
Cu	-1.24436998	-2.52835465	4.35183155	0.03600426	0.09218542	-0.14315346
Cu	-1.26407043	-0.03302518	4.48555615	0.02941213	0.00062112	-0.11491853
Cu	-1.22374663	2.45938447	4.37334175	0.03526690	-0.09127522	-0.14366165
Cu	0.66033214	-2.51303371	-3.50468625	-0.03439100	0.09057055	0.14383536
Cu	0.69375446	-0.01219985	-3.61676738	-0.02781506	-0.00051700	0.11420261
Cu	0.67265660	2.48607019	-3.48170455	-0.03504511	-0.09183602	0.14318833
Cu	0.94240195	-1.33381698	4.18914094	-0.02506670	0.04195583	-0.10178280
Cu	0.95734916	1.23662561	4.20348843	-0.02146087	-0.03981260	-0.10116477
Cu	2.53275363	-1.30865617	-2.36119019	-0.07959253	0.03654372	0.07922891
Cu	2.54167058	1.27002995	-2.34693533	-0.08000601	-0.03694232	0.07903335
Cu	-3.11045113	-1.30613565	3.21811888	0.08019427	0.03659813	-0.07890181
Cu	-3.10223251	1.26998136	3.22633579	0.07992441	-0.03668785	-0.07889004
Cu	-1.51970113	-1.30063990	-3.33885173	0.03527712	0.03613122	0.10701255
Cu	-1.51353882	1.28320426	-3.32583044	0.03493669	-0.03694470	0.10645370
Cu	3.30794033	2.05522917	-0.00913151	-0.10137364	-0.05892666	0.01387925
Cu	2.69005728	2.03608893	2.49283991	-0.07794686	-0.06383421	-0.04768729
Cu	-3.25706634	2.07294533	-1.60057094	0.08399566	-0.05917097	0.05743310
Cu	-3.86516405	2.07150816	0.89926071	0.10154879	-0.05959357	-0.01335326

Cu	3.29187040	-2.12145150	-0.03997570	-0.10054351	0.05931636	0.01401887
Cu	2.68217229	-2.13480662	2.45921945	-0.08156439	0.06371047	-0.05226091
Cu	-3.26447794	-2.10056362	-1.62235963	0.08459319	0.05862416	0.05851245
Cu	-3.87871227	-2.09916967	0.88254131	0.10134978	0.05875557	-0.01292341
Cu	-2.01586294	3.35209745	2.16939969	0.04903310	-0.09532381	-0.04902322
Cu	0.48868513	3.34009981	2.78262869	-0.02092507	-0.09692726	-0.06614863
Cu	-2.04964387	-3.39451600	2.14734226	0.04992356	0.09584599	-0.04861772
Cu	0.46148677	-3.41256125	2.74544997	-0.02031228	0.09612570	-0.06500523
Cu	-1.03954427	3.36176292	-1.88298452	0.02091003	-0.09607711	0.06551636
Cu	1.47130890	3.35119215	-1.27016209	-0.04935876	-0.09547028	0.04866876
Cu	-1.05445747	-3.39504524	-1.90521015	0.02152221	0.09479837	0.06608182
Cu	1.44885673	-3.40477507	-1.30487968	-0.04922943	0.09594019	0.04976359
O	4.55608043	-0.02822464	5.39531418	-0.01614976	-0.00048896	-0.01719322
H	4.01847348	-0.10233951	6.22269525	-0.00978455	-0.00047125	0.00190170
H	4.86577316	0.91060333	5.40126349	-0.00242316	0.00808596	-0.00477534

Cu_{55} (a)

58

Lattice="31.12 0.0 0.0 0.0 26.57 0.0 0.0 0.0 26.05" Properties=species:S:1:pos:R:3:forces:R:3

Cu	-0.36174234	0.01369608	0.61948862	-0.00050488	0.00015506	0.00020367
Cu	1.93168679	-0.02047536	-0.09101767	-0.16919177	0.00233699	0.05703051
Cu	1.32454305	0.00957832	2.32522736	-0.12232313	0.00078658	-0.13006168
Cu	-2.03381002	0.01702064	-1.09422727	0.12704677	-0.00149977	0.13195431
Cu	-2.64535917	0.05047809	1.34629968	0.17459295	-0.00265976	-0.05587251
Cu	-1.55896147	2.06295055	0.29704627	0.09277149	-0.15634416	0.02482125
Cu	0.88802835	2.04185922	0.90892273	-0.09322308	-0.15649762	-0.02159315
Cu	-1.61116732	-2.01287856	0.33385226	0.09631472	0.15422197	0.02284828
Cu	0.83660833	-2.03914777	0.93857092	-0.08593705	0.16043860	-0.02148713
Cu	-0.87028403	-1.22039912	2.60493148	0.03905664	0.09569331	-0.15109348
Cu	-0.83463240	1.29872602	2.58454828	0.03494879	-0.09819877	-0.15071153
Cu	0.12196571	-1.27460993	-1.34029032	-0.03639136	0.09810221	0.15019268
Cu	0.15219102	1.24934226	-1.36225225	-0.04041893	-0.09561140	0.15072728
Cu	4.15812944	-0.05504159	-0.85047721	-0.15489942	-0.00111639	0.06625168
Cu	3.72390270	0.01412772	1.64697668	-0.15723591	0.01357073	-0.03922952
Cu	2.92579358	-0.00559188	4.05100026	-0.10622627	-0.00384137	-0.13095849
Cu	-3.67829530	0.01760039	-2.78587955	0.12048957	0.00000448	0.12360407
Cu	-4.40468699	0.04328348	-0.38853765	0.11439635	-0.00084634	0.02872865
Cu	-4.89330857	0.08648821	2.06864545	0.16474429	-0.00275013	-0.05230115
Cu	-2.73947273	4.08161381	-0.02213005	0.08659301	-0.14812851	0.02395489
Cu	-0.30843476	4.18450469	0.57301158	-0.00132445	-0.11791860	0.00135338
Cu	2.11157105	4.03747846	1.19695785	-0.08887756	-0.14747354	-0.01997461
Cu	-2.84368626	-4.00904310	0.06288349	0.08980342	0.14611141	0.02054536
Cu	-0.41892939	-4.15878736	0.67620673	0.00130075	0.11779974	-0.00137865
Cu	2.01945939	-4.05196205	1.26538494	-0.08461010	0.14945652	-0.02339746
Cu	-1.38184832	-2.43826656	4.56181036	0.03672513	0.08871670	-0.14410436

Cu	-1.37191718	0.06733736	4.65943135	0.03062183	-0.00145081	-0.11574853
Cu	-1.31325547	2.56764080	4.51680237	0.03485445	-0.09281898	-0.14222979
Cu	0.58007878	-2.54770123	-3.27266849	-0.03477399	0.09244817	0.14191728
Cu	0.64704193	-0.04214811	-3.41901008	-0.02714178	0.00144941	0.11609446
Cu	0.64582000	2.46561548	-3.32013322	-0.03654264	-0.08869560	0.14430493
Cu	0.81999218	-1.26437425	4.40610276	-0.03192718	0.03576109	-0.10781430
Cu	0.85062681	1.32197394	4.39326102	-0.03285145	-0.03715024	-0.10712366
Cu	2.46158174	-1.34872028	-2.13307030	-0.07828507	0.03842755	0.07855691
Cu	2.49233150	1.23723193	-2.16606986	-0.07959286	-0.03483099	0.07972340
Cu	-3.22215096	-1.19703028	3.40753670	0.08109508	0.03493118	-0.07901309
Cu	-3.18793423	1.37738652	3.36996363	0.08013974	-0.03863478	-0.07787691
Cu	-1.58370548	-1.30405224	-3.14082799	0.03475039	0.03772149	0.10630548
Cu	-1.55584273	1.28481805	-3.16344221	0.03381933	-0.03586594	0.10732041
Cu	3.24540531	2.06136376	0.16443075	-0.09457204	-0.06230054	0.01615442
Cu	2.60715164	2.09275913	2.69048128	-0.07624706	-0.06364908	-0.05756935
Cu	-3.30931929	2.11210785	-1.45800086	0.08347873	-0.05904765	0.05903994
Cu	-3.92869508	2.15255717	1.03563046	0.10153437	-0.06051651	-0.01122553
Cu	3.20932134	-2.11722779	0.20740588	-0.08609490	0.06632715	0.01954375
Cu	2.58056943	-2.08513486	2.69837894	-0.06671670	0.06494321	-0.05933966
Cu	-3.34293464	-2.06621924	-1.43112289	0.08509272	0.05847060	0.05795846
Cu	-3.97720938	-2.02460920	1.07707550	0.10240127	0.05737579	-0.01269990
Cu	-2.07435721	3.43544833	2.29787410	0.04888822	-0.09700626	-0.04745688
Cu	0.41554139	3.41642518	2.92600129	-0.02184410	-0.09717069	-0.06551343
Cu	-2.17517306	-3.31622412	2.37036869	0.05156037	0.09475462	-0.04968533
Cu	0.33525641	-3.35170643	2.98488031	-0.01927133	0.09604120	-0.06706021
Cu	-1.06835823	3.37341669	-1.74537321	0.02009546	-0.09497159	0.06734351
Cu	1.44025099	3.35518515	-1.12288126	-0.05042938	-0.09531251	0.05003591
Cu	-1.13914236	-3.39217857	-1.67022999	0.02195696	0.09589071	0.06489890
Cu	1.35468020	-3.41370064	-1.05835530	-0.04834086	0.09822884	0.04766708
O	5.90284149	-0.06452222	2.22033595	-0.02674278	0.00142428	-0.00724138
H	5.76279419	-0.70643087	2.96307886	-0.02063396	-0.00478038	0.00400693
H	6.16139683	-0.65083625	1.46488263	-0.01590083	-0.00450067	-0.01332612

Cu(111)*Cu

31

Lattice="7.65796644025031 0.0 0.0 3.828983220125155 6.6319934785854535 0.0 0.0 0.0 21.968468943549098" Properties=species:S:1:pos:R:3:tags:I:1:forces:R:3

Cu	1.25548872	0.72092222	7.96762137	3	0.00109830	0.00306411	0.11428721
Cu	3.80233549	0.72058813	7.91663110	3	-0.00153970	-0.00069527	0.12172414
Cu	6.37266602	0.72418304	7.91756310	3	0.00308424	-0.00062106	0.12200926
Cu	2.53226323	2.93289428	7.95258739	3	-0.00317924	-0.00083602	0.11702852
Cu	5.09052008	2.94263136	7.91984559	3	0.00077090	0.00302785	0.12182934
Cu	7.64032308	2.93535046	7.96611414	3	0.00320188	0.00102242	0.11419338
Cu	3.81962699	5.14848761	7.94925328	3	0.00095146	0.00057387	0.11797322
Cu	6.35786442	5.14427638	7.96333903	3	0.00243292	-0.00011825	0.11534241

Cu	8.91325777	5.14400523	7.94589482	3	-0.00163147	-0.00250835	0.11842814
Cu	-0.01079922	1.46817250	10.02866033	2	-0.00064890	0.00045668	0.00960782
Cu	2.54774762	1.46544115	10.00781436	2	0.00381730	0.00121058	0.01430942
Cu	5.10652185	1.47311270	9.95713220	2	0.00024752	0.00015943	0.03173454
Cu	1.27701156	3.68369301	10.02371792	2	-0.00499234	-0.00464177	-0.00056511
Cu	3.83498325	3.68752037	9.99902687	2	0.00136166	-0.00542883	0.01766348
Cu	6.37194473	3.67456670	10.02857656	2	0.00020683	-0.00143409	0.01010878
Cu	2.56417812	5.89231208	9.98703682	2	-0.00444755	0.00492172	0.02042491
Cu	5.10755865	5.89524319	10.05816651	2	0.00043936	0.00080735	-0.00127332
Cu	7.64713662	5.88906545	9.99597704	2	0.00408844	0.00385218	0.01741486
Cu	0.01675892	0.00642337	12.11993431	1	0.02221489	-0.01046537	-0.12211288
Cu	2.58285013	0.01345748	12.12976325	1	-0.02463145	-0.01304247	-0.12692957
Cu	5.12720241	-0.04596319	11.98820942	1	-0.00017178	-0.00935998	-0.10839127
Cu	1.28520916	2.21580819	12.08274510	1	0.00388592	-0.00098816	-0.09681324
Cu	3.81608450	2.24070301	12.02004519	1	-0.00746148	0.00390011	-0.10728216
Cu	6.39676273	2.21822763	12.10725385	1	0.00433137	0.00244953	-0.10774254
Cu	2.57388474	4.42682727	12.07562303	1	-0.00102729	0.00115951	-0.09501045
Cu	5.12289956	4.43295188	12.12000749	1	0.00085202	0.01933118	-0.11846187
Cu	7.66674169	4.41938998	12.07973873	1	0.00177256	0.00490644	-0.09696894
Cu	4.96892048	1.32899083	13.87272134	0	-0.01147186	-0.00928063	-0.18142453
O	4.97423044	1.35497050	15.96486474	0	-0.00048152	-0.00060808	-0.02616339
H	5.92825243	1.31631269	16.22551688	0	0.00982988	-0.00039252	0.00261507
H	4.70648826	2.27558086	16.21475536	0	-0.00290284	0.00957790	0.00244477

Cu(100)*Cu

31

Lattice="7.65796644025031 0.0 0.0 0.0 7.65796644025031 0.0 0.0 0.0 21.91" Properties=species:S:1:pos:R:3:tags:I:1:forces:R:3

Cu	-0.05140145	-0.01675204	8.07473323	3	-0.00137132	-0.00323109	0.00053740
Cu	2.51182106	-0.01087661	8.09202644	3	-0.00152438	-0.00346024	0.00052527
Cu	5.05512124	-0.00867163	8.09432140	3	-0.00299281	-0.00351186	-0.00019869
Cu	-0.05152672	2.54609084	8.08235413	3	-0.00119846	-0.00490245	0.00205266
Cu	2.51266014	2.53463722	8.09782195	3	-0.00154747	-0.00460464	0.00206868
Cu	5.05208877	2.53615757	8.09654288	3	-0.00311273	-0.00482741	0.00169806
Cu	-0.05026367	5.10065385	8.09003255	3	-0.00104206	-0.00406493	0.00138397
Cu	2.50427739	5.09532687	8.07807962	3	-0.00202068	-0.00420101	0.00221153
Cu	5.06456946	5.09719611	8.07848567	3	-0.00317930	-0.00431711	0.00161629
Cu	1.22208550	1.25831755	9.82101557	2	0.00001360	-0.00446638	0.00258394
Cu	3.78150351	1.25575183	9.84966342	2	-0.00298106	-0.00302901	0.00245580
Cu	6.33389446	1.26386246	9.82921535	2	-0.00292426	-0.00426236	0.00357417
Cu	1.22476321	3.82461446	9.81670428	2	-0.00132410	-0.00466171	0.00007678
Cu	3.78516174	3.82109828	9.82749604	2	-0.00297746	-0.00493665	0.00143966
Cu	6.33421428	3.82289056	9.82578363	2	-0.00164134	-0.00472388	0.00122045
Cu	1.23055668	6.36709611	9.82547701	2	0.00136502	-0.00265627	0.00573711
Cu	3.78334773	6.35890622	9.82770535	2	-0.00312921	-0.00216715	0.00463193

Cu	6.33134643	6.37149542	9.83303406	2	-0.00375207	-0.00245372	0.00574474
Cu	-0.05091692	-0.01210423	11.57594761	1	-0.00171794	-0.00371309	0.00599153
Cu	2.47456945	-0.06587963	11.58296489	1	-0.00096979	-0.00367777	0.00517276
Cu	5.07714635	-0.04999744	11.59865481	1	-0.00277264	-0.00139199	0.00652127
Cu	-0.05006725	2.55677103	11.57909714	1	-0.00278702	-0.00542657	0.00271031
Cu	2.45797428	2.56788794	11.54722468	1	-0.00473998	-0.00254808	-0.00567611
Cu	5.09157817	2.56786116	11.57657814	1	0.00079394	-0.00161980	-0.00153951
Cu	-0.04218083	5.09932031	11.59295351	1	-0.00192842	-0.00517987	0.00494138
Cu	2.50455731	5.07555613	11.59589391	1	-0.00088769	-0.00386660	0.00500386
Cu	5.06555944	5.08642492	11.59077693	1	-0.00270150	-0.00253327	0.00570761
Cu	3.75356168	1.25423874	13.11158740	0	0.00078510	-0.00629443	-0.00116975
O	3.44905835	2.65697295	14.68746262	0	-0.01218406	0.04268053	-0.02781588
H	4.34750714	3.00721632	14.91537916	0	0.01985137	0.05989633	-0.04752522
H	3.09529497	3.35621171	14.06110986	0	-0.01357453	0.00496425	-0.03064713

Cu(211)*Cu

31

Lattice="6.252703415323648 0.0 0.0 0.0 7.65796644025031 0.0 0.0 0.0 23.695105314298182" Properties=species:S:1:pos:R:3:forces:R:3

Cu	4.18940761	1.25024902	13.73465851	0.00927387	0.00038609	-0.11990047
Cu	4.17378601	3.82899221	13.74942772	0.00885138	-0.00130147	-0.11853187
Cu	4.15569023	6.37109590	13.74911995	-0.00881561	0.00162238	-0.11739371
Cu	2.09149457	0.00470798	13.19326756	-0.03403752	-0.02494073	-0.13236515
Cu	2.07169561	2.54713386	13.07115858	-0.03646618	-0.00030548	-0.11550791
Cu	2.08839677	5.08989375	13.18884664	-0.03331658	0.02492987	-0.13112668
Cu	0.05311377	1.26111688	12.48410830	0.02758017	0.01085341	-0.08956010
Cu	0.04649897	3.82973497	12.49144129	0.02891372	-0.01092616	-0.09128868
Cu	0.05569792	6.37666633	12.51262309	0.03386484	-0.00112110	-0.10623812
Cu	4.24742945	-0.00943845	11.64563466	0.01113149	0.00339019	-0.03588233
Cu	4.25562988	2.54558625	11.61881141	0.00815908	0.00007838	-0.02795114
Cu	4.24633087	5.09355241	11.65180727	0.01189661	-0.00240169	-0.03648995
Cu	2.17019058	1.25906132	10.96585347	0.00046337	0.00525737	0.01781008
Cu	2.16781235	3.83072335	10.96668008	0.00110572	-0.00446575	0.01695533
Cu	2.15854824	6.37461620	11.02004226	0.00463523	-0.00020384	0.00258830
Cu	0.07376541	-0.01726031	10.30615773	-0.01230839	0.00150927	0.05714525
Cu	0.08832817	2.54547892	10.30997158	-0.01692173	-0.00036605	0.05601480
Cu	0.07230839	5.10554240	10.30916924	-0.01189201	-0.00198905	0.05592860
Cu	4.27036969	1.26188192	9.44721296	-0.03116005	-0.00027018	0.11907743
Cu	4.26943261	3.82718801	9.44770168	-0.03079922	0.00053273	0.11885505
Cu	4.26126308	6.37225753	9.46521062	-0.02275788	0.00004553	0.11448836
Cu	2.22068620	-0.01900764	8.82072994	0.02846822	-0.00047126	0.11936900
Cu	2.23439872	2.54477713	8.80497703	0.02559251	0.00016538	0.12206675
Cu	2.21987746	5.10563627	8.82094889	0.02909564	0.00032385	0.11931835
Cu	0.17336746	1.26865779	8.21499047	0.00138839	0.00004766	0.13947803
Cu	0.17241345	3.81990782	8.21489143	0.00159209	-0.00008738	0.13914384

Cu	0.15334760	6.37288982	8.20388870	0.00452824	-0.00002842	0.13801294
Cu	2.82684368	2.50885717	15.27405899	-0.02408456	-0.00200542	-0.17666983
O	3.51197063	2.47991277	17.31291929	-0.00020745	-0.00025389	-0.02355887
H	4.27067221	1.86211329	17.14569212	0.01510290	-0.00714814	-0.00920829
H	3.95943890	3.36466377	17.35393008	0.01112372	0.00914390	-0.00457902

References

63. Raju, R. K., Rodriguez, P. & Johnston, R. L. Can a Single Valence Electron Alter the Electrocatalytic Activity and Selectivity for CO2 Reduction on the Subnanometer Scale? *J. Phys. Chem. C* **123**, 14591–14609 (2019).

88. Calle-Vallejo, F. (2023). The ABC of generalized coordination numbers and their use as a descriptor in electrocatalysis. *Advanced Science*, *10*(20), 2207644.

100. Martínez, L., Andrade, R., Birgin, E. G., & Martínez, J. M. (2009). PACKMOL: A package for building initial configurations for molecular dynamics simulations. *Journal of computational chemistry*, *30*(13), 2157-2164.

101. Spicher, S., & Grimme, S. (2020). Robust atomistic modeling of materials, organometallic, and biochemical systems. *Angewandte Chemie International Edition*, *59*(36), 15665-15673.

102. Bannwarth, C., Caldeweyher, E., Ehlert, S., Hansen, A., Pracht, P., Seibert, J., Spicher, S., & Grimme, S. (2021). Extended tight-binding quantum chemistry methods. *Wiley Interdisciplinary Reviews: Computational Molecular Science*, *11*(2), e1493.

109. Ohnishi, Y. Y., Nakao, Y., Sato, H., Nakao, Y., Hiyama, T., & Sakaki, S. (2009). A theoretical study of nickel (0)-catalyzed phenylcyanation of alkynes. Reaction mechanism and regioselectivity. *Organometallics*, *28*(8), 2583-2594.

110. Marenich, A. V., Kelly, C. P., Thompson, J. D., Hawkins, G. D., Chambers, C. C., Giesen, D. J., ... & Truhlar, D. G. (2020). Minnesota solvation database (MNSOL) version 2012.

111. Guba, M. & Höltzl, T. Stability and Electronic Structure of Nitrogen-Doped Graphene-Supported Cun (n = 1–5) Clusters in Vacuum and under Electrochemical Conditions: Toward Sensor and Catalyst Design. J. Phys. Chem. C 128, 4677–4686 (2024).

112. Johnson, E. R., Keinan, S., Mori-Sánchez, P., Contreras-García, J., Cohen, A. J., & Yang, W. (2010). Revealing noncovalent interactions. *Journal of the American Chemical Society*, *132*(18), 6498-6506.

113. Cheng, T., Xiao, H., & Goddard III, W. A. (2016). Reaction mechanisms for the electrochemical reduction of CO2 to CO and formate on the Cu (100) surface at 298 K from quantum mechanics free energy calculations with explicit water. *Journal of the American Chemical Society*, *138*(42), 13802-13805.

114. Hou, X., Qi, L., Li, W., Zhao, J. & Liu, S. Theoretical study on water behavior on the copper surfaces. *J. Mol. Model.* 27, 149 (2021).
